# Abatacept Pharmacokinetics and Exposure Response in Patients Hospitalized With COVID-19: A Secondary Analysis of the ACTIV-1 IM Randomized Clinical Trial

**DOI:** 10.1001/jamanetworkopen.2024.7615

**Published:** 2024-04-25

**Authors:** Stephen J. Balevic, Daniel K. Benjamin, William G. Powderly, P. Brian Smith, Daniel Gonzalez, Matthew W. McCarthy, Linda K. Shaw, Christopher J. Lindsell, Sam Bozzette, Daphne Williams, Benjamin P. Linas, John Blamoun, Heta Javeri, Christoph P. Hornik

**Affiliations:** 1 Duke Clinical Research Institute, Durham, North Carolina; 2 Department of Pediatrics, Duke University School of Medicine, Durham, North Carolina; 3 Division of Infectious Diseases, Department of Medicine, Washington University in St Louis, St Louis, Missouri; 4 Department of Medicine, Weill Cornell Medicine, New York, New York; 5 National Center for Advancing Translational Sciences, Bethesda, Maryland; 6 Bristol Myers Squibb, Philadelphia, Pennsylvania; 7 Section of Infectious Diseases, Department of Medicine, Boston Medical Center, Boston University School of Medicine, Boston, Massachusetts; 8 Department of Critical Care, MyMichigan Health, Midland; 9 Division of Infectious Diseases, Department of Medicine, University of Texas Health Science Center, San Antonio

## Abstract

**Importance:**

The pharmacokinetics of abatacept and the association between abatacept exposure and outcomes in patients with severe COVID-19 are unknown.

**Objective:**

To characterize abatacept pharmacokinetics, relate drug exposure with clinical outcomes, and evaluate the need for dosage adjustments.

**Design, Setting, and Participants:**

This study is a secondary analysis of data from the ACTIV-1 (Accelerating COVID-19 Therapeutic Interventions and Vaccines) Immune Modulator (IM) randomized clinical trial conducted between October 16, 2020, and December 31, 2021. The trial included hospitalized adults who received abatacept in addition to standard of care for treatment of COVID-19 pneumonia. Data analysis was performed between September 2022 and February 2024.

**Exposure:**

Single intravenous infusion of abatacept (10 mg/kg with a maximum dose of 1000 mg).

**Main Outcomes and Measures:**

Mortality at day 28 was the primary outcome of interest, and time to recovery at day 28 was the secondary outcome. Drug exposure was assessed using the projected area under the serum concentration time curve over 28 days (AUC_0-28_). Logistic regression modeling was used to analyze the association between drug exposure and 28-day mortality, adjusted for age, sex, and disease severity. The association between time to recovery and abatacept exposure was examined using Fine-Gray modeling with death as a competing risk, and was adjusted for age, sex, and disease severity.

**Results:**

Of the 509 patients who received abatacept, 395 patients with 848 serum samples were included in the population pharmacokinetic analysis. Their median age was 55 (range, 19-89) years and most (250 [63.3%]) were men. Abatacept clearance increased with body weight and more severe disease activity at baseline. Drug exposure was higher in patients who survived vs those who died, with a median AUC_0-28_ of 21 428 (range, 8462-43 378) mg × h/L vs 18 262 (range, 9628-27 507) mg × h/L (*P* < .001). Controlling for age, sex, and disease severity, an increase of 5000 units in AUC_0-28_ was associated with lower odds of mortality at day 28 (OR, 0.52 [95% CI, 0.35-0.79]; *P* = .002). For an AUC_0-28_ of 19 400 mg × h/L or less, there was a higher probability of recovery at day 28 (hazard ratio, 2.63 [95% CI, 1.70-4.08] for every 5000-unit increase; *P* < .001). Controlling for age, sex, and disease severity, every 5000-unit increase in AUC_0-28_ was also associated with lower odds of a composite safety event at 28 days (OR, 0.46 [95% CI, 0.33-0.63]; *P* < .001). Using the dosing regimen studied in the ACTIV-1 IM trial, 121 of the 395 patients (30.6%) would not achieve an abatacept exposure of at least 19 400 mg × h/L, particularly at the extremes of body weight. Using a modified, higher-dose regimen, only 12 patients (3.0%) would not achieve the hypothesized target abatacept exposure.

**Conclusions and Relevance:**

In this study, patients who were hospitalized with severe COVID-19 and achieved higher projected abatacept exposure had reduced mortality and a higher probability of recovery with fewer safety events. However, abatacept clearance was high in this population, and the current abatacept dosing (10 mg/kg intravenously with a maximum of 1000 mg) may not achieve optimal exposure in all patients.

**Trial Registration:**

ClinicalTrials.gov Identifier: NCT04593940

## Key Points

**Question:**

Is abatacept exposure associated with clinical outcomes in patients with severe COVID-19?

**Findings:**

In this secondary analysis of abatacept pharmacokinetics and exposure-response data for 395 hospitalized patients in the ACTIV-1 IM randomized clinical trial, those who achieved higher projected abatacept exposure had significantly reduced mortality, a higher probability of recovery, and fewer composite safety events. Abatacept clearance and exposure were related to total body weight and baseline disease severity.

**Meaning:**

In this study, abatacept was shown to be efficacious in patients hospitalized with severe COVID-19, although some patients may require higher dosing.

## Teaser

This secondary analysis of a randomized clinical trial examines the pharmacokinetics of abatacept and the association between abatacept exposure and outcomes in patients hospitalized with severe COVID-19.

## Introduction

Infection with COVID-19 can result in a clinical spectrum ranging from asymptomatic illness to hospitalization and even death.^1^ Mortality in patients with severe COVID-19 often occurs secondary to a heightened systemic inflammatory response known as a cytokine storm.^2,3^ This cytokine storm is characterized by significant elevations of multiple inflammatory cytokines.^2,3^ Due to the strong link between the dysregulated immune system and outcomes in COVID-19, multiple immunomodulatory drugs have been studied in the treatment of severe COVID-19.^4,5^

Abatacept (Orencia; Bristol Myers Squibb) is a recombinant fusion protein that inhibits T-cell activation, thereby reducing multiple inflammatory cytokines, including interleukin 6 and tumor necrosis factor α, that are part of the COVID-19 cytokine storm.^6,7^ In the ACTIV-1 (Accelerating COVID-19 Therapeutic Interventions and Vaccines) Immune Modulator (IM) multicenter randomized clinical trial, abatacept, combined with standard of care that often included remdesivir and corticosteroids, decreased mortality in patients hospitalized with moderate to severe COVID-19, but the primary end point of time to recovery was not met.^8^ However, the pharmacokinetics of abatacept and optimal dosing in this patient population are unknown. Because increased body weight is a risk factor for severe COVID-19 and is associated with increased abatacept clearance (CL),^9^ it is possible that the pharmacokinetics of abatacept may be different in hospitalized patients with COVID-19. Accordingly, we conducted a planned secondary analysis of the ACTIV-1 IM trial with the goals to (1) characterize abatacept pharmacokinetics, (2) relate exposure with clinical outcomes, and (3) determine the need for dosage adjustments to reach target drug exposure for COVID-19.

## Methods

### Study Design

This is a secondary analysis of abatacept pharmacokinetics and exposure-response data collected from the ACTIV-1 IM randomized clinical trial. The ACTIV-1 IM methods and full eligibility criteria were previously published.^8^ Briefly, the ACTIV-1 IM multinational trial was conducted between October 16, 2020, and December 31, 2021, using the ACTIV-1 IM master protocol (Supplement 1) that randomized adults hospitalized with moderate to severe COVID-19 to 1 of 3 immune modulators or placebo plus standard of care. All patients also received remdesivir if eligible, and most received corticosteroids. Patients self-reported race and ethnicity, which were considered important covariates due to potential genetic polymorphisms that could affect drug pharmacokinetics and outcomes. Race was reported as American Indian or Alaska Native, Asian, Black or African American (hereinafter, Black), White, other race (further categorization was not available in the pharmacokinetics dataset), or unknown race; ethnicity was reported as Hispanic or Latino (hereinafter, Hispanic), not Hispanic or Latino, or unknown ethnicity. The protocol was approved by institutional review boards at each site or a centralized institutional review board, and informed consent was obtained from all participants or their authorized representative. This study followed the Consolidated Standards of Reporting Trials (CONSORT) reporting guideline.

Patients were eligible for this pharmacokinetics and exposure-response analysis if they were enrolled in the ACTIV-1 IM trial, received abatacept, and had pharmacokinetic samples with detectable concentration from venous blood. eFigure 1 in Supplement 2 presents the overall CONSORT diagram.

Abatacept was administered on day 1 as a single 10-mg/kg intravenous infusion over approximately 30 minutes, with a maximum dose of 1000 mg. Pharmacokinetic sample collection and assay methods are presented in the eMethods in Supplement 2.

### Pharmacokinetic Model Development and Simulations

Abatacept serum pharmacokinetic data were analyzed using nonlinear mixed-effects modeling with Phoenix NLME software, version 8.4 (Certara). We employed a standardized population pharmacokinetics approach as outlined in the eMethods in Supplement 2.

Once a final pharmacokinetic model was selected, we used the following equations to derive individual, model-projected pharmacokinetic parameters and simulate a concentration every 0.5 hour for 28 days:

CL = tvCL × (WT/70 kg)^dClwt^ × exp(ηCL)

V_1_ = tvV_1_ × (WT/70 kg)^dVwt^

V_2_ = tvV_2_

Q = tvQ

where CL is the clearance from the central compartment, tv is the typical population value of a parameter, WT is total body weight (in kilograms), dClwt is the exponent describing the power relationship between weight and CL, η is the deviation from the average population pharmacokinetic parameter value, V_1_ is the volume of distribution in the central compartment, dVwt is the exponent describing the power relationship between weight and V_1_, V_2_ is the volume of distribution in the peripheral compartment, and Q is the intercompartmental CL.

Using this rich time vs concentration profile, we then conducted a noncompartmental analysis to derive each patient’s simulated area under the serum concentration time curve over 28 days (AUC_0-28_) and maximum and minimum concentrations (C_max_ and C_min_) over 28 days.

### Exposure-Response Analysis

Based on results from the ACTIV-1 IM trial, we used mortality at day 28 as our primary outcome of interest and time to recovery at day 28 as our secondary outcome. Recovery was defined as the first day a participant attained category 6, 7, or 8 on the 8-point ordinal scale of disease severity, generally meaning not requiring continuous oxygen and ongoing medical care (a score of 1 indicates death, whereas a score of 8 indicates not hospitalized and no limitations on activities). We used AUC_0-28_ as our primary exposure of interest, because this represents the total amount of drug in the body over the study period. Additionally, we explored C_max_ and C_min_ as exposure metrics. We also evaluated the association between AUC_0-28_ and the composite safety outcome at day 28, defined as the occurrence of death, a serious adverse event, or a grade 3 or 4 adverse event within 28 days.

### Dosage Simulations

The optimal abatacept exposure was determined based on the observed data for the primary and secondary outcomes. Using the final population pharmacokinetic model estimates and each patient’s actual covariates and estimated interindividual variability (IIV), we then conducted dosing simulations (1 replicate) to determine the number of patients projected to achieve the target abatacept exposure derived from the exposure-response analysis (eMethods in Supplement 2).

### Statistical Analysis

We used descriptive statistics to summarize baseline demographics, clinical characteristics, medication usage, and outcomes. Differences in abatacept exposure (AUC_0-28_, C_max_, and C_min_) across mortality status and recovery groups were compared using the Kruskal-Wallis test. Additionally, differences in abatacept AUC_0-28_ across the composite safety outcome were analyzed using the Kruskal-Wallis test.

We used adjusted and unadjusted logistic regression modeling to analyze the association between abatacept exposure (AUC_0-28_, C_max_, and C_min_) and 28-day mortality. The logistic regression model was adjusted for age, sex, and disease severity; additional modeling assumptions and sensitivity analyses are noted in the eMethods in Supplement 2.

The association between time to recovery and abatacept exposure (AUC_0-28_, C_max_, and C_min_) was examined using Fine-Gray modeling^10^ with death as a competing risk and was adjusted for age, sex, and disease severity. Linearity of the exposure variables was tested prior to inclusion in all models, and splines or linear transformations were used when linearity was violated.^11^

**Figure 1.**
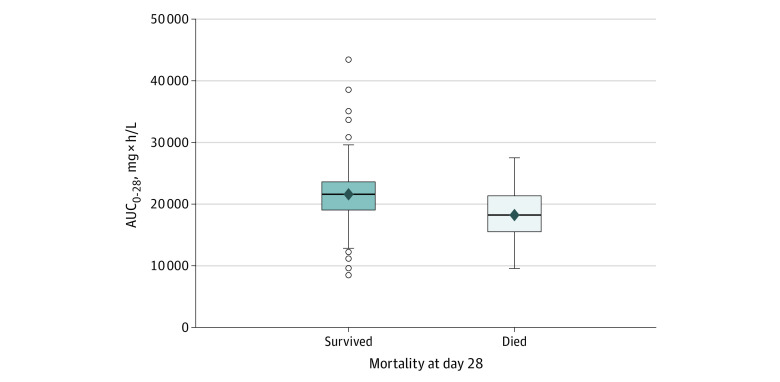
Abatacept Exposure and Mortality at 28 Days The solid line in each box plot represents the median; the diamond represents the mean. Open circles represent data beyond 1.5 times the IQR; *P* < .001. AUC_0-28_ indicates the area under the serum concentration time curve over 28 days. Not all outlier data points can be seen owing to overlapping symbols.

**Figure 2.**
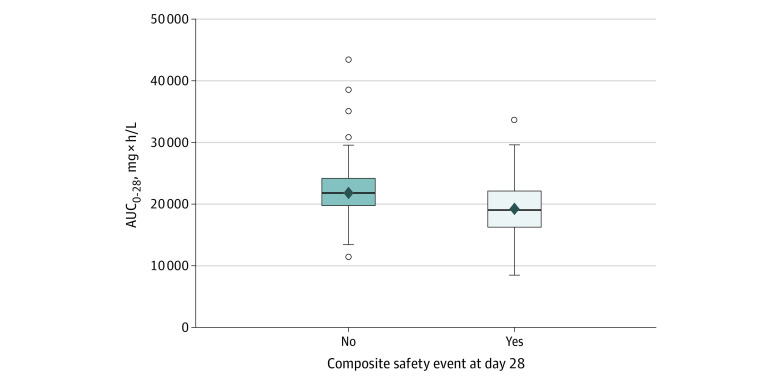
Abatacept Exposure and Safety The solid line in each box plot represents the median; the diamond represents the mean. Open circles represent data beyond 1.5 times the IQR; *P* < .001. AUC_0-28_ indicates the area under the serum concentration time curve over 28 days. Not all outlier data points can be seen owing to overlapping symbols.

All statistical procedures were conducted in SAS, version 9.4 TS1M7 (SAS Institute Inc); R, version 4.1.1 (R Project for Statistical Computing); and RStudio, version 1.4.1717 (RStudio Inc). Figures 1 and 2 were generated using the Box and Whisker function and the exclusive quartile calculation in Microsoft Excel 2016 (Microsoft Corp). All tests were 2 tailed and statistical significance was declared at α < .05 unless otherwise noted. Data analysis was performed between September 2022 and February 2024.

## Results

### Baseline Demographics, Clinical Characteristics, and Samples

Altogether, 509 participants received abatacept in the ACTIV-1 IM trial. A total of 414 participants had pharmacokinetic samples collected, but 1 patient had only pharmacokinetic samples from the extracorporeal membrane oxygenation (ECMO) circuit and was excluded from the analysis. Of the remaining 413 patients, there were a total of 897 pharmacokinetic samples from venous blood. The ECMO samples were not used in the analysis. We excluded 49 samples (5.5%) as outlined in eFigure 1 in Supplement 2, resulting in a final population of 395 patients with 848 serum samples. Their median age was 55 (range, 19-89) years; 145 (36.7%) were women and 250 (63.3%) were men. Patients reported being American Indian or Alaska Native (5 [1.3%]), Asian (15 [3.8%]), Black (48 [12.2%]), White (239 [60.5%]), or of other race (64 [16.2%]); race was unknown for 24 (6.1%). Patients also reported being Hispanic (163 [41.3%]) or not Hispanic or Latino (216 [54.7%]); ethnicity was unknown for 16 (4.1%). Additional demographic and clinical characteristics for the 395 patients are noted in Table 1. The median number of samples per patient was 2 (range, 1-4). Patients received a single abatacept infusion at a median weight-based dose of 10 (range, 4.1-12.9) mg/kg and an absolute dose of 910 (range, 386-1000) mg.

**Table 1.** Baseline Demographics and Clinical Characteristics^a^

Characteristic	Values (N = 395)
Sex	
Female	145 (36.7)
Male	250 (63.3)
Race	
American Indian or Alaska Native	5 (1.3)
Asian	15 (3.8)
Black or African American	48 (12.2)
White	239 (60.5)
Other^b^	64 (16.2)
Unknown	24 (6.1)
Ethnicity	
Hispanic or Latino	163 (41.3)
Not Hispanic or Latino	216 (54.7)
Unknown	16 (4.1)
Age, median (range), y	55 (19-89)
Weight, median (range), kg	91 (38.6-243.6)
Creatinine, median (range), mg/dL (n = 391)^c^	0.8 (0.25-13)
BMI, median (range) (n = 391)^c^	31.1 (14.6-75.8)
Obesity at baseline (n = 391)^c^	226 (57.8)
Hypertension at baseline	160 (40.5)
Disease severity at baseline (8-point ordinal scale)	
Death (1)	0
Hospitalized, invasive ventilation or ECMO (2)	34 (8.6)
Hospitalized, noninvasive ventilation or high-flow oxygen devices (3)	131 (33.2)
Hospitalized, requiring supplemental oxygen (4)	212 (53.7)
Hospitalized, not requiring oxygen, requiring ongoing medical care (5)	18 (4.6)
Hospitalized, not requiring oxygen, not requiring ongoing medical care (6)	0
Not hospitalized, limitations in activity or requiring home oxygen (7)	0
Not hospitalized, no limitations on activities (8)	0
Any tocilizumab use	11 (2.8)
Any baricitinib use	5 (1.3)
Any dexamethasone use	340 (86.1)
ECMO (ever)	8 (2.0)

Abbreviations: BMI, body mass index (calculated as weight in kilograms divided by height in meters squared); ECMO, extracorporeal membrane oxygenation.

SI conversion factor: To convert creatinine to μmol/L, multiply by 88.4.

^a^ Unless indicated otherwise, values are presented as the No. (%) of participants.

^b^ Further categorization was not available in the dataset.

^c^ Data were missing for 4 patients.

### Population Pharmacokinetic Model Development

The base model that best characterized the observed data was a 2-compartment structural model with linear elimination, multiplicative error, and estimates of IIV on abatacept CL (eTable in Supplement 2). After covariate selection, the best statistical model included an effect of baseline disease severity (ordinal), weight, and body mass index (BMI; calculated as weight in kilograms divided by height in meters squared) on CL and weight and BMI on V_1_. In this model, post hoc empirical bayesian estimates of pharmacokinetic parameters suggested that patients who ever received ECMO had higher abatacept CL, with a median of 0.07 (range, 0.02-0.08) L/h compared with 0.04 (range, 0.01-0.08) L/h. However, this model had less physiologic plausibility because the effect of disease severity was not consistent across categories, and there was collinearity between weight and BMI. Moreover, a reduced model (including only weight on CL and V_1_) had overall similar performance, which allows for direct comparison with parameter estimates from published abatacept pharmacokinetic models^9^ and is easier for clinicians to interpret. Accordingly, we selected the reduced model as the final population pharmacokinetic model. A sensitivity analysis comparing these models is presented in the eResults in Supplement 2.

### Final Population Pharmacokinetic Model and Model Evaluation

The final pharmacokinetic model was a 2-compartment model with linear elimination and multiplicative error, IIV on CL, and a power relationship for weight normalized to a 70-kg adult on V_1_ and CL. Parameter estimates are presented in Table 2, and diagnostic plots and the prediction-corrected visual predictive check for the final pharmacokinetic model are presented in eFigures 2 and 3 in Supplement 2. Overall, the final model had good parameter precision with no obvious model misspecification, and the majority of observed concentrations fell within the 90% projection interval. Abatacept CL appeared higher in patients with more severe disease activity at baseline (eFigure 4 in Supplement 2).

**Table 2.** Parameters for the Final Abatacept Pharmacokinetic Model

Parameter	Estimate	RSE, %	2.5th percentile	Bootstrap median	97.5th percentile
V_1_, L/70 kg	4.40	3.70	4.05	4.35	4.66
V_2_, L	4.49	8.46	4.01	4.58	5.55
CL, L/h/70 kg	0.031	3.76	0.028	0.031	0.033
Q, L/h	0.031	32.17	0.021	0.032	0.054
Exponential scaling of weight/70 kg on CL	0.62	13.21	0.45	0.62	0.79
Exponential scaling of weight/70 kg on V_1_	0.50	26.38	0.24	0.52	0.75
Interindividual variability (CV%)					
CL	32.08	20.01	NC	NC	NC
Residual error					
Multiplicative error (%)	27.66	7.23	23.67	27.17	31.10

Abbreviations: CL, clearance from the central compartment; CV, coefficient of variation; NC, not calculated; Q, intercompartmental clearance; RSE, relative standard error; V_1_, volume of distribution in the central compartment; V_2_, volume of distribution in the peripheral compartment.

### Noncompartmental Analysis

Using simulated time vs concentration profiles from the final population pharmacokinetic model, a noncompartmental analysis was conducted to derive individual patient exposures_._ Across all patients, the median AUC_0-28_ was 21 185 (IQR, 18 662-23 534) mg × h/L, C_max_ was 172.9 (IQR, 161.5-181.3) mg/L, and C_min_ was 6.9 (IQR, 5.2-8.9) mg/L (Table 3).

**Table 3.** Projected and Target Exposures From Dosing Simulations^a^

Dosing	Projected exposure			Optimal exposure		
	AUC_0-28_, mg × h/L	C_min_, mg/L	C_max_, mg/L	AUC_0-28_, mg × h/L	C_min_, mg/L	C_max_, mg/L
ACTIV-1 IM trial (10 mg/kg, maximum 1000 mg)	21 185 (18 662-23 534)	6.9 (5.2-8.9)	172.9 (161.5-181.3)	19 400-21 428^b^	7.1^c^	Efficacy: not established; safety: 175-427^d^
High dose (<60 kg = 1000 mg; 60-100 kg = 1250 mg; >100 kg = 1500 mg)	32 285 (28 500-35 869)	10.7 (7.7-13.6)	259.4 (247.5-273.0)			

Abbreviations: ACTIV-1 IM, Accelerating COVID-19 Therapeutic Interventions and Vaccines Immune Modulator; AUC_0-28_, area under the serum concentration time curve over 28 days; C_max_, maximum concentration; C_min_, minimum concentration.

^a^ Data represent the median (25th-75th percentiles) derived from Phoenix NLME software, version 8.4 (Certara).

^b^ Lower end of target derived from the inflection point of the time-to-event analysis and higher end derived from median exposure in the survival group.

^c^ Target derived from median exposure in the survival group.

^d^ Range of exposure observed in healthy volunteers receiving a single dose of abatacept at 10 mg/kg.

### Exposure Response

Overall, 349 patients (88.4%) were alive and 46 (11.6%) had died by day 28. The AUC_0-28_ was significantly higher in patients who survived vs those who died (Figure 1), with a median of 21 428 (range, 8462-43 378) mg × h/L vs 18 262 (range, 9628-27 507) mg × h/L (*P* < .001). Similarly, C_min_ was significantly higher in those who survived, with a median of 7.1 (range, 1.2-38.7) mg/L vs 4.8 (range, 0.7-11.5) mg/L (*P* < .001), whereas there was no significant difference with C_max_.

In logistic regression modeling, no linearity violations were observed for abatacept exposure variables and 28-day mortality. Controlling for age, sex, and disease severity, a 5000-unit increase in AUC_0-28_ was associated with lower odds of mortality at day 28 (OR, 0.52 [95% CI, 0.35-0.79]; *P* = .002). Similarly, there was an association between C_min_ and 28-day mortality with an odds ratio of 0.80 (95% CI, 0.70-0.92; *P* = .002); however, there was no association with C_max_ and mortality. Besides abatacept AUC_0-28_ and C_min_, the only other statistically significant covariates in the multivariable model of survival at day 28 were age and disease severity at baseline. In a sensitivity analysis, addition of concomitant medications to the model did not significantly alter the association between AUC_0-28_ and 28-day mortality. There was no significant interaction of disease severity with drug exposure and mortality.

In time-to-recovery analyses, 320 patients (81.0%) recovered over the 28-day period, 32 (8.1%) did not recover, and 43 (10.9%) died. Three patients initially recovered but later died; these patients were treated as recovered in the time-to-recovery analysis and counted as deceased in the mortality analysis. The unadjusted association between AUC_0-28_ and outcome violated the linearity assumption and was best characterized using 2 linear pieces with an inflection point at 19 400 mg × h/L. Controlling for age, sex, and disease severity, every 5000-unit increase in an AUC_0-28_ of 19 400 mg × h/L or less was associated with a higher probability of recovery at day 28, with a hazard ratio of 2.63 (95% CI, 1.70-4.08; *P* < .001). In the adjusted setting, AUC values greater than 19 400 mg × h/L did not increase the likelihood of recovery (hazard ratio, 1.10 [95% CI, 0.90-1.33]; *P* = .36). The probability of 28-day recovery across AUC_0-28_ levels is displayed in eFigure 5 in Supplement 2. Additionally, higher C_min_ was associated with a higher probability of recovery at day 28, but the association was nonlinear. There was no association with C_max_.

### Exposure Safety

Altogether, 128 of 395 patients (32.4%) experienced a composite safety event through day 28. Of these 128 patients, 46 (35.9%) died. Abatacept AUC_0-28_ was significantly higher in patients who did not have a safety event through day 28 (median [range], 21 838 [11 505-43 378] vs 19 167 [8462-33 625] mg × h/L; *P* < .001; Figure 2). In logistic regression modeling and controlling for age, sex, and disease severity, every 5000-unit increase in AUC_0-28_ was associated with lower odds of a composite safety event at 28 days (OR, 0.46 [95% CI, 0.33-0.63]; *P* < .001). When the 46 patients who died were excluded, the AUC_0-28_ remained associated with lower odds of the composite safety event (odds ratio, 0.51 [95% CI, 0.36-0.72]; *P* < .001), suggesting the association was not entirely driven by a reduction in mortality.

### Dosage Simulations

The proposed minimum therapeutic target for abatacept AUC_0-28_ was determined to be approximately 19 400 to 21 428 mg × h/L based on the median AUC_0-28_ in the survival group as well as the inflection point observed in the time-to-recovery analysis. Using the dosing regimen studied in the ACTIV-1 IM trial, 121 of 395 patients (30.6%) would not achieve an abatacept exposure of at least 19 400 mg × h/L, particularly at the extremes of body weight (eFigure 6A in Supplement 2). Using the modified rheumatoid arthritis regimen, only 12 patients (3.0%) would not achieve the proposed target abatacept AUC_0-28_ (eFigure 6B in Supplement 2). In addition, the individual projected C_max_ for the modified rheumatoid arthritis regimen approximated that observed in abatacept phase 1 clinical trials (eFigures 7 and 8 in Supplement 2).^12^ A summary of exposures for the dosing simulations is provided in Table 3.

## Discussion

In this study, we observed that patients hospitalized with moderate to severe COVID-19 who achieved higher abatacept exposure had improved outcomes with fewer safety events. Additionally, we found that the current abatacept dosing (10 mg/kg intravenously with a maximum of 1000 mg) may not achieve the proposed optimal exposure in this population, particularly those at the extremes for body weight or who are critically ill. These results highlight that drug pharmacokinetics and dosing cannot simply be extrapolated from one population (eg, patients with rheumatoid arthritis) to another.

The US Food and Drug Administration has highlighted that exposure-response data derived from well-controlled studies can contribute substantial evidence of effectiveness and support dosing.^13^ Accordingly, our analyses add to the preponderance of evidence supporting abatacept efficacy in hospitalized patients with severe COVID-19. Our results broadly support the current National Institutes of Health guidelines for the treatment of COVID-19, which recommend abatacept administration for hospitalized patients who require oxygen, including noninvasive ventilation.^14^ However, we found that the exposure-response relationship for mortality was not dependent on baseline disease severity, suggesting that mechanically ventilated patients may also benefit from abatacept. Although time to recovery was not statistically significant compared with placebo in the overall trial, our analysis used a different exposure metric whereby we quantified drug concentrations only in the group receiving abatacept. Because approximately a third of patients who received abatacept in the ACTIV-1 IM trial may have had suboptimal exposure, it is possible that too few patients achieved sufficient abatacept concentrations compared with placebo to detect a difference in the primary study’s time-to-recovery analysis.

We made several important observations regarding abatacept pharmacokinetics in this population. Overall, CL appeared to be higher in patients hospitalized with severe COVID-19 compared with other populations, resulting in lower abatacept concentrations. For example, after administration of a single abatacept dose (10 mg/kg intravenously), the mean C_max_ was 292 (range, 175-427) mg/L in 13 healthy volunteers and the mean (SD) C_max_ was 202 (7.7) mg/L in patients with hematologic malignancies in a previous study compared with a mean (range) of 170 (94-215) mg/L in the ACTIV-1 IM trial.^12,15^ Additionally, the systemic CL of abatacept in patients with rheumatoid arthritis was approximately half (15.4 vs 31 mL/h/70 kg) of the CL observed in our study.^12^ The mechanism for higher CL in this population is unclear but could be due to higher body weights, with more than half of our population being obese, or to the underlying inflammatory state and hospitalization leading to increased protein catabolism or target-mediated drug disposition. Because we observed higher abatacept CL in patients receiving ECMO or mechanical ventilation at baseline, it is also possible that the lack of mortality benefit observed in the overall clinical trial in this subgroup^8^ was attributable to subtherapeutic exposure.

Due to the higher CL of abatacept in patients hospitalized with severe COVID-19, we conducted dosing simulations and found that a higher-dose abatacept regimen would be necessary for most patients to achieve the exposure that resulted in optimal benefit derived from this ACTIV-1 IM cohort. Additionally, we showed that most patients would not experience maximum abatacept concentrations exceeding those of healthy volunteers. This finding, combined with the observation that safety events occurred more frequently at lower (not higher) abatacept exposures, and the linear pharmacokinetics for abatacept^9^ all provide reassurance that higher doses could be studied in future clinical trials. However, it is important to note that these target abatacept exposures may not be representative in other patient populations.

We observed that drug exposure was higher in patients who did not experience the composite safety events. The reason for higher drug exposure in this subgroup is not entirely clear but may be partially due to the reduction in mortality observed in patients with higher drug exposure. Additionally, patients with low drug exposures were more likely to have critical illness, obesity, or both, which may be independent risk factors for adverse events.

This study was conducted between October 2020 and December 2021, before the predominant Omicron variant and subvariants. Although mortality has decreased with new variants,^16^ cytokine storms are believed to be a final common pathway caused by a variety of disorders^2^ for which abatacept may improve mortality.^17^ Accordingly, we would expect similar efficacy of abatacept when COVID-19 results in a hyperinflammatory state, despite continued evolution of the virus.

### Limitations

This study has some limitations. Because of the sparse pharmacokinetic sampling in the trial, we were unable to compute drug exposures using actual (observed) drug concentrations and instead derived individual projected concentrations. Accordingly, the data rely heavily on the performance of the pharmacokinetic model. Due to approximately 30% residual variability in the model and high IIV in abatacept CL, it is expected that actual drug concentrations may vary from those projected by our model. Additionally, the range of abatacept exposures was limited by a dosing cap of 1000 mg and both the safety and efficacy of higher doses of abatacept require confirmation. In addition, mortality in patients with COVID-19 pneumonia is often the result of complex interactions among underlying comorbidities, heterogeneity in the disease process, and treatment effects; and therapeutic abatacept concentrations alone do not guarantee certain outcomes. Finally, the efficacy of abatacept as monotherapy cannot be determined from this analysis.

## Conclusions

In this secondary analysis of the ACTIV-1 IM multinational randomized clinical trial, patients who were hospitalized with severe COVID-19 and achieved higher projected abatacept exposure had reduced mortality and a higher probability of recovery with fewer safety events. However, abatacept CL was high in this population, and the current abatacept dosing (10 mg/kg intravenously with a maximum of 1000 mg) may not achieve optimal exposure in all patients, particularly those at the extremes for body weight or those who are critically ill. Clinical trials in future pandemics could be optimized by evaluating exposure-response relationships during the study and leveraging an adaptive design to adjust dosing.

## Notes

Supplement 1.
Trial Protocol

Supplement 2.
eMethods.

eResults.
**eTable.** Pharmacokinetic Parameters for the Base Model **eFigure 1.** CONSORT Diagram **eFigure 2.** Diagnostic Plots for the Final Abatacept Model **eFigure 3.** Prediction-Corrected Visual Prediction Check for the Final Population Pharmacokinetic Model **eFigure 4.** Abatacept Clearance and Baseline Disease Severity **eFigure 5.** Abatacept Exposure and Probability of Recovery at 28 Days **eFigure 6.** Target Abatacept Attainment From Dosage Simulations **eFigure 7.** Sensitivity Analysis for Maximum Abatacept Concentration Using IPRED **eFigure 8.** Maximum Abatacept Concentration Using DV
eReferences.

Supplement 3.
Nonauthor Collaborators

Supplement 4.
Data Sharing Statement
