# Supplementary material for: Abatacept Pharmacokinetics and Exposure Response in Patients Hospitalized With COVID-19: A Secondary Analysis of the ACTIV-1 IM Randomized Clinical Trial
Source: JAMA Netw Open. 2024 Apr 25;7(4):e247615. doi: 10.1001/jamanetworkopen.2024.7615 (PMC11046337; doi:10.1001/jamanetworkopen.2024.7615)
Supplement: Supplement 1. — Trial Protocol [file jamanetwopen-e247615-s001.pdf]

Randomized Master Protocol for Immune Modulators for Treating COVID-19

Short Title: ACTIV-1 IM

Protocol ID: ACTIV-1 IM

Sponsor: Daniel K. Benjamin, MD, MPH, PhD
Kiser-Arena Distinguished Professor,
Duke Clinical Research Institute

Version Number: 1.0

08 September 2020

Sponsor Contact information:

Daniel K. Benjamin, MD, MPH, PhD
Kiser-Arena Distinguished Professor, Duke University Pediatrics
Duke Clinical Research Institute
300 W. Morgan Street, Suite 800
Durham Center
Durham, NC 27701
Tel: 919-668-7081
Fax: 919-681-9457
E-mail: [redacted]

STATEMENT OF COMPLIANCE

Each institution engaged in this research will hold a current Federalwide Assurance (FWA) issued by the U.S. Office of Human Research Protection (OHRP) for federally funded research. The Institutional Review Board (IRB)/Independent or Institutional Ethics Committee (IEC) must be registered with OHRP as applicable to the research.

The study will be carried out in accordance with the following as applicable:

- All National and Local Regulations and Guidance applicable at each site
- The International Council for Harmonization of Technical Requirements for Registration of Pharmaceuticals for Human Use (ICH) E6(R2) Good Clinical Practice, and the Belmont Report: Ethical Principles and Guidelines for the Protection of Human Subjects of Research, Report of the National Commission for the Protection of Human Subjects of Biomedical and Behavioral Research
- United States (US) Code of Federal Regulations (CFR) 45 CFR Part 46: Protection of Human Subjects
- US Food and Drug Administration (FDA) Regulations: 21 CFR Part 50 (Protection of Human Subjects), 21 CFR Part 54 (Financial Disclosure by Clinical Investigators), 21 CFR Part 56 (IRBs), 21 CFR Part 11, and 21 CFR Part 312 (Investigational New Drug Application), and/or 21 CFR 812 (Investigational Device Exemptions)

The signature below provides the necessary assurance that this study will be conducted according to all stipulations of the protocol including statements regarding confidentiality, and according to local legal and regulatory requirements, US federal regulations, and ICH E6(R2) GCP guidelines.

Site Investigator Signature:

Signed: ____ Date: ____________________
Name and Title

Table of Contents

STATEMENT OF COMPLIANCE 2
1. PROTOCOL SUMMARY 10
1.1. ACTIV-1 IM – Synopsis 10
1.1.1. *Study Overview* 10
1.1.2. *Enrollment Period* 10
1.1.3. *General* 10
1.1.4. *Study Population* 11
1.1.5. *Inclusion Criteria* 11
1.1.6. *Exclusion Criteria at Screening* 11
1.1.7. *Study Intervention* 12
1.1.8. *Schedule of Assessments* 13
2. INTRODUCTION 15
2.1. Background 15
2.1.1. *ACTIV-1 IM – Immune Modulators & Remdesivir Multi-arm Trial* 15
2.2. Risk/Benefit Assessment 15
2.2.1. *Known Potential Risks of Study Participation* 15
2.2.1.1. *Risks to Privacy* 15
2.2.2. *SoC - Remdesivir* 16
2.2.2.1. *Potential Risks of Remdesivir* 16
2.2.2.2. *Potential Benefits of Remdesivir* 16
2.2.2.3. *Assessment of Potential Risks and Benefits of Remdesivir* 16
2.2.3. *Therapeutic Agents* 17
3. OBJECTIVES AND ENDPOINTS 17
4. STUDY DESIGN 19
4.1. Overall Design 19
4.2. Scientific Rationale for Study Design 19
4.3. Justification for Dose 20
4.3.1. *Justification for Dose of Remdesivir* 20
4.3.2. *Justification for Dose of Investigational Agents* 20
5. STUDY POPULATION 20
5.1. Inclusion Criteria 20
5.2. Exclusion Criteria at Screening 20
5.3. Exclusion of Specific Populations 21
5.4. Inclusion of Vulnerable Subjects 21
5.5. Lifestyle Considerations 22
5.6. Screen Failures 22
5.7. Strategies for Recruitment and Retention 22
5.7.1. *Recruitment* 22
5.7.2. *Retention* 22
5.8. Compensation Plan for Subjects 23
5.9. Costs 23
6. STUDY PRODUCT 23
6.1. Study Product(s) and Administration 23

6.1.1. *Study Product Description*......23
6.1.2. *Dosing and Administration*......23
6.1.3. *Dose Escalation*......24
6.1.4. *Dose Modifications*......24
6.1.5. *Overdosage*......24
6.1.6. *Preparation/Handling/Storage/Accountability*......24
6.1.6.1. *Acquisition and Accountability*......24
6.1.6.2. *Formulation, Appearance, Packaging, and Labeling*......25
6.1.6.3. *Product Storage and Stability*......25
6.1.6.4. *Preparation*......25
6.2. Measures to Minimize Bias: Randomization and Blinding......26
6.3. Study Intervention Compliance......26
6.4. Concomitant Therapy......26
6.4.1. *Permitted Concomitant Therapy and Procedures*......26
6.4.2. *Prohibited Concomitant Therapy*......27
6.4.3. *Rescue Medicine*......27
6.4.4. *Non-Research Standard of Care*......28
7. STUDY INTERVENTION DISCONTINUATION AND SUBJECT DISCONTINUATION/WITHDRAWAL......28
7.1. Halting Criteria and Discontinuation of Study Intervention......28
7.1.1. *Individual Study Product Halting*......28
7.1.2. *Study Halting*......28
7.2. Withdrawal from the Study......28
7.3. Lost to Follow-Up......28
7.4. Readmission......29
8. STUDY ASSESSMENTS AND PROCEDURES......29
8.1. Screening and Efficacy Assessments......29
8.1.1. *Screening Procedures*......29
8.1.2. *Efficacy Assessments*......30
8.1.3. *Exploratory Assessments*......32
8.2. Safety and Other Assessments......33
8.2.1. *Procedures to be Followed in the Event of Abnormal Laboratory Test Values or Abnormal Clinical Findings*......35
8.2.2. *Unscheduled Visits*......35
8.3. Adverse Events and Serious Adverse Events......35
8.3.1. *Definition of Adverse Event (AE)*......35
8.3.2. *Definition of Serious Adverse Event (SAE)*......35
8.3.3. *Suspected Unexpected Serious Adverse Reactions (SUSAR)*......36
8.3.4. *Classification of an Adverse Event*......36
8.3.5. *Time Period and Frequency for Event Assessment and Follow-Up*......37
8.3.6. *Serious Adverse Event Reporting*......37
8.3.7. *Reporting of Pregnancy*......38
8.4. Unanticipated Problems......38
8.4.1. *Definition of Unanticipated Problems*......38

8.4.2. *Unanticipated Problem Reporting* 38
9. STATISTICAL CONSIDERATIONS 38
9.1. Statistical Hypotheses 39
9.2. Sample Size Determination 39
9.3. Populations for Analyses 41
9.4. Statistical Analyses 41
9.4.1. *General Approach* 41
9.4.2. *Analysis of the Primary Efficacy Endpoint* 41
9.4.3. *Analysis of the Secondary Endpoint(s)* 42
9.4.4. *Safety Analyses* 42
9.4.5. *Baseline Descriptive Statistics* 42
9.4.6. *Planned Early and Interim Analyses* 42
9.4.7. *Sub-Group Analyses* 44
9.4.8. *Exploratory Analyses* 44
10. SUPPORTING DOCUMENTATION AND OPERATIONAL CONSIDERATIONS – ALL STAGES 44
APPENDIX 1 – SUB-STUDY 1 – INFLIXIMAB (REMICADE) 45
1. SUB-STUDY SUMMARY 45
1.1. Sub-study overview 45
1.2. Enrollment Period 45
1.3. General 45
1.4. Study Population 45
1.5. Inclusion Criteria 45
1.6. Agent Specific Exclusion Criteria at Randomization 45
1.7. Study Intervention 45
1.8. Schedule of Assessments 46
2. JUSTIFICATION FOR SELECTION 46
3. INTRODUCTION 46
3.1. Risk/Benefit Assessment 46
3.1.1. *Remicade* 46
3.1.1.1. *Potential Risks of Remicade* 46
3.1.1.2. *Potential Benefits of Remicade* 47
3.1.1.3. *Assessment of Potential Risks and Benefits of Remicade* 47
4. OBJECTIVES AND ENDPOINTS 48
5. SUB-STUDY DESIGN 48
5.1. Justification for Dose 48
5.1.1. *Justification for Dose of Remicade* 48
6. STUDY POPULATION 48
6.1. Inclusion Criteria 48
6.2. Sub-Study Specific Exclusion Criteria 48
6.3. Exclusion of Specific Populations 49
7. STUDY PRODUCT 49
7.1. Study Product(s) and Administration 49
7.1.1. *Study Product Description* 49

7.1.2. *Dosing and Administration* 49
7.1.3. *Dose Escalation* 49
7.1.4. *Dose Modifications* 50
7.1.5. *Overdosage* 50
7.1.6. *Preparation/Handling/Storage/Accountability* 50
7.1.6.1. *Acquisition and Accountability* 50
7.1.6.2. *Formulation, Appearance, Packaging, and Labeling* 50
7.1.6.3. *Product Storage and Stability* 51
7.1.6.4. *Preparation and Administration* 51
7.2. Measures to Minimize Bias: Randomization and Blinding 51
7.3. Study Intervention Compliance 51
7.4. Concomitant Therapy 51
7.4.1. *Permitted Concomitant Therapy and Procedures* 51
7.4.2. *Prohibited Concomitant Therapy* 51
7.4.3. *Rescue Medicine* 52
7.4.4. *Non-Research Standard of Care* 52
8. STUDY INTERVENTION DISCONTINUATION AND SUBJECT DISCONTINUATION/WITHDRAWAL 52
8.1. Halting Criteria and Discontinuation of Study Intervention 52
8.1.1. *Individual Study Product Halting* 52
8.1.2. *Study Halting* 52
8.2. Withdrawal from the Study 52
8.3. Lost to Follow-Up 52
8.4. Readmission 52
9. STUDY ASSESSMENTS AND PROCEDURES 52
10. STATISTICAL CONSIDERATIONS 52
APPENDIX 2 – SUB-STUDY 2 – ABATACEPT (ORENCIA) 53
1. SUB-STUDY SUMMARY 53
1.1. Sub-study overview 53
1.2. Enrollment Period 53
1.3. General 53
1.4. Study Population 53
1.5. Inclusion Criteria 53
1.6. Agent Specific Exclusion Criteria 53
1.7. Study Intervention 53
1.8. Schedule of Assessments 54
2. JUSTIFICATION FOR SELECTION 54
3. INTRODUCTION 56
3.1. Risk/Benefit Assessment 56
3.1.1. *Abatacept* 56
3.1.1.1. *Potential Risks of Abatacept* 56
3.1.1.2. *Potential Benefits of Abatacept* 58
3.1.1.3. *Assessment of Potential Risks and Benefits of Abatacept* 58
4. OBJECTIVES AND ENDPOINTS 58

5. STUDY DESIGN 59
5.1. Justification for Dose 59
5.1.1. *Justification for Dose of Abatacept* 59
6. STUDY POPULATION 59
6.1. Inclusion Criteria 59
6.2. Sub-Study Specific Exclusion Criteria 59
6.3. Exclusion of Specific Populations 59
7. STUDY PRODUCT 60
7.1. Study Product(s) and Administration 60
7.1.1. *Study Product Description* 60
7.1.2. *Dosing and Administration* 60
7.1.3. *Dose Escalation* 60
7.1.4. *Dose Modifications* 60
7.1.5. *Overdosage* 61
7.1.6. *Preparation/Handling/Storage/Accountability* 61
7.1.6.1. *Acquisition and Accountability* 61
7.1.6.2. *Formulation, Appearance, Packaging, and Labeling* 62
7.1.6.3. *Product Storage and Stability* 62
7.1.6.4. *Preparation* 62
7.2. Measures to Minimize Bias: Randomization and Blinding 63
7.3. Study Intervention Compliance 63
7.4. Concomitant Therapy 63
7.4.1. *Permitted Concomitant Therapy and Procedures* 63
7.4.2. *Prohibited Concomitant Therapy* 63
7.4.3. *Rescue Medicine* 63
7.4.4. *Non-Research Standard of Care* 63
8. STUDY INTERVENTION DISCONTINUATION AND SUBJECT DISCONTINUATION/WITHDRAWAL 64
8.1. Halting Criteria and Discontinuation of Study Intervention 64
8.1.1. *Individual Study Product Halting* 64
8.1.2. *Study Halting* 64
8.2. Withdrawal from the Study 64
8.3. Lost to Follow-Up 64
8.4. Readmission 64
9. STUDY ASSESSMENTS AND PROCEDURES 64
10. STATISTICAL CONSIDERATIONS 64
APPENDIX 3 – SUB-STUDY 3 – CENICRIVIROC (CVC) 65
1. SUB-STUDY SUMMARY 65
1.1. Sub-study overview 65
1.2. Enrollment Period 65
1.3. General 65
1.4. Study Population 65
1.5. Inclusion Criteria 65
1.6. Agent Specific Exclusion Criteria 65

1.7. Study Intervention 65
1.8. Schedule of Assessments 65
2. JUSTIFICATION FOR SELECTION 66
3. INTRODUCTION 68
3.1. Risk/Benefit Assessment 68
3.1.1. *Cenicriviroc* 68
3.1.1.1. *Potential Risks of Cenicriviroc* 68
3.1.1.2. *Potential Benefits of Cenicriviroc* 69
3.1.1.3. *Assessment of Potential Risks and Benefits of Cenicriviroc* 69
4. OBJECTIVES AND ENDPOINTS 70
5. STUDY DESIGN 70
5.1. Justification for Dose 70
5.1.1. *Justification for Dose of Cenicriviroc* 70
6. STUDY POPULATION 73
6.1. Inclusion Criteria 73
6.2. Sub-Study Specific Exclusion Criteria 73
6.3. Exclusion of Specific Populations 73
7. STUDY PRODUCT 73
7.1. Study Product(s) and Administration 73
7.1.1. *Study Product Description* 73
7.1.2. *Dosing and Administration* 74
7.1.3. *Dose Escalation* 75
7.1.4. *Dose Modifications* 75
7.1.4.1. *Dose Modifications for Adverse Events* 75
7.1.5. *Overdosage* 76
7.1.6. *Preparation/Handling/Storage/Accountability* 76
7.1.6.1. *Acquisition and Accountability* 76
7.1.6.2. *Formulation, Appearance, Packaging, and Labeling* 77
7.1.6.3. *Product Storage and Stability* 77
7.1.6.4. *Preparation* 77
7.2. Measures to Minimize Bias: Randomization and Blinding 77
7.3. Study Intervention Compliance 78
7.4. Concomitant Therapy 78
7.4.1. *Permitted Concomitant Therapy and Procedures* 78
7.4.2. *Prohibited Concomitant Therapy* 78
7.4.3. *Rescue Medicine* 80
7.4.4. *Non-Research Standard of Care* 80
8. STUDY INTERVENTION DISCONTINUATION AND SUBJECT DISCONTINUATION/WITHDRAWAL 80
8.1. Halting Criteria and Discontinuation of Study Intervention 80
8.1.1. *Individual Study Product Halting* 80
8.1.2. *Study Halting* 80
8.2. Withdrawal from the Study 80
8.3. Lost to Follow-Up 80

8.4. Readmission 81
9. STUDY ASSESSMENTS AND PROCEDURES 81
10. STATISTICAL CONSIDERATIONS 81
11. REFERENCES 82

ACTIV-1 IM: RANDOMIZED MASTER PROTOCOL FOR IMMUNE MODULATORS FOR TREATING COVID-19

1. PROTOCOL SUMMARY

1.1. ACTIV-1 IM – Synopsis

1.1.1. *Study Overview*

ACTIV-1 IM is a master protocol designed to evaluate multiple investigational agents for the treatment of moderately or severely ill patients infected with severe acute respiratory syndrome coronavirus 2 (SARS-CoV-2). The research objectives are to evaluate each agent with respect to speed of recovery, mortality, illness severity, and hospital resource utilization. Each agent will be evaluated as add-on therapy to the standard of care (SoC) in use at the local clinics, including remdesivir (provided). The SoC may change during the course of the study based on other research findings. Comparisons of the agents among themselves is not a research objective.

The study population corresponds to moderately and severely ill patients infected with the coronavirus disease 2019 (COVID-19) virus. Recruitment will target patients already hospitalized for treatment of COVID-19 infection as well as patients being treated for COVID-19 infection in Emergency Departments while waiting to be admitted to the hospital. Patients both in and out of the ICU are included in the study population.

Enrollment is planned to begin when 2-3 agents have been selected for initial evaluation and are available for testing. Up to 5 agents may be evaluated in ACTIV-1 IM.

The study period is 29 days, with assessments on each day of the hospital stay. Patients will be followed after hospital discharge with periodic follow-up assessments through Day 29. There will also be a safety and clinical status assessment at 60 days. Treatment periods may vary by agent.

The trial is adaptive in that interim analyses are planned to assess the futility of each agent, with the goal of discontinuing those with lower probabilities of success to more effectively utilize trial resources for the remaining agents. Additionally, interim analyses are planned for early stopping for efficacy. Alpha spending functions are used to appropriately control the probability of making an erroneous conclusion at the interim and final analyses.

Safety monitoring will be performed throughout the trial, and formal stopping rules for each agent will be adopted. The Data and Safety Monitoring Board (DSMB) established for ACTIV-1 IM will have oversight responsibility for the study.

1.1.2. *Enrollment Period*

Enrollment is expected to begin in September 2020. It is anticipated the enrollment may be completed in 4-6 months.

1.1.3. *General*

ACTIV-1 IM is a master protocol designed to evaluate immune modulators for the treatment of moderately or severely ill hospitalized patients infected with COVID-19. Trial participants will be assessed daily while hospitalized. If the participants are discharged from the hospital prior to Day 29, they will have follow-up study visits at Days 8, 11, 15, 22, and 29. For discharged participants, it is preferred that the Day 8, 11, 15, and 29 visits are in person to obtain safety laboratory tests and blood (serum/plasma) samples for secondary research as well as clinical outcome data. However, infection control or other restrictions may limit the ability of the participant to return to the clinic. In this case, these visits may be conducted by phone, and only clinical data will be obtained. The Day 22 visit does not have laboratory tests or collection of samples and is conducted by phone. The Day 60 assessment will be conducted by phone.

The effectiveness of each therapeutic agent as add-on therapy to SoC plus remdesivir (provided) will be evaluated based on the primary endpoint of time to recovery by Day 29. The sample size requirements are based on the ability to detect a moderate improvement in time to recovery (3-4 fewer days) for each agent (see Section 9.2). A total of 788 recoveries are required for each comparison to provide approximately 85% power to detect a recovery rate ratio of 1.25. Assuming 73% of participants achieve recovery in 28 days, consistent with the ACTT-1 results, the total sample size to evaluate 1, 2, and 3 agents in ACTIV-1 IM is approximately 1080, 1620, and 2160, respectively. Because each agent is being compared to SoC with no between-agent comparisons, no multiplicity adjustments for multiple agents are planned.

1.1.4. *Study Population*

Hospitalized adults (≥18 years old) with COVID-19, including patients both in and out of the ICU. Patients seeking care for COVID-19 in an Emergency Department (ED) and waiting to be admitted to the hospital are included.

1.1.5. *Inclusion Criteria*

1. Admitted to a hospital or awaiting admission in the ED with symptoms suggestive of COVID-19.
2. Subject (or legally authorized representative) provides informed consent prior to initiation of any study procedures.
3. Subject (or legally authorized representative) understands and agrees to comply with planned study procedures.
4. Male or non-pregnant female adults ≥18 years of age at time of enrollment.
5. Has laboratory-confirmed (within 14 days prior to enrollment) SARS-CoV-2 infection as determined by PCR or other commercial or public health assay in any specimen.
6. Ongoing illness of any duration, and at least one of the following:
    - Radiographic infiltrates by imaging (chest x-ray, CT scan, etc.), OR
    - Blood oxygen saturation (SpO_2) ≤ 94% on room air, OR
    - Requiring supplemental oxygen, OR
    - Requiring mechanical ventilation or ECMO.
7. Women of childbearing potential must agree to either abstinence or use at least one primary form of contraception not including hormonal contraception from the time of screening through Day 60.
8. Agrees to not participate in another interventional trial for the treatment of COVID-19 through Day 60.

1.1.6. *Exclusion Criteria at Screening*

1. ALT or AST >5 times the upper limit of normal.
2. Estimated glomerular filtration rate (eGFR) <30 mL/min (including patients receiving hemodialysis or hemofiltration).
3. Neutropenia (absolute neutrophil count <1000 cells/μL) (<1.0 x 10^3/μL or <1.0 GI/L).
4. Lymphopenia (absolute lymphocyte count <200 cells/μL) (<0.20 x 10^3/μL or <0.20 GI/L)
5. Pregnancy or breast feeding.
6. Anticipated discharge from the hospital or transfer to another hospital which is not a study site within 72 hours.
7. Known allergy to any study medication.
8. Received cytotoxic or biologic treatments (such as anti-interleukin-1 [IL-1], anti-IL-6 [tocilizumab or sarilumab], anti-IL-17, or T-cell or B-cell targeted therapies (e.g., rituximab), tyrosine kinase inhibitors including baricitinib, TNF inhibitors, or interferon within 4 weeks or 5 half-lives prior to screening. Steroid dependency defined as need for prednisone at a dose >10 mg (or equivalent) for >1 month within 2 weeks of screening is exclusionary. Note 1: Dexamethasone (at a dose of 6 mg per day for up to 10 days) is permitted for the treatment of COVID-19 in patients who are already mechanically ventilated and in patients who require supplemental oxygen at screening, but who are not mechanically ventilated in accordance with national guidelines. Note 2: Infusion of convalescent plasma is also allowed.
9. Based on medical history and concomitant therapies that would suggest infection, have suspected clinical diagnosis of current active tuberculosis (TB) or, if known, latent TB treated for less than 4 weeks with appropriate anti-tuberculosis therapy per local guidelines (by history only, no screening required).

10. Based on medical history and concomitant therapies that would suggest infection, suspected serious, active bacterial, fungal, viral (including, but not limited to, active HBV, HCV, or HIV/AIDS).
11. Have received any live vaccine (that is, live attenuated) within 3 months before screening, or intend to receive a live vaccine (or live attenuated) during the study. Note: Use of non-live (inactivated) vaccinations is allowed for all participants.
12. Severe hepatic impairment (defined as liver cirrhosis Child stage C).
13. Current severe heart failure (New York Heart Association [NYHA] III-IV).
14. In the Investigator's judgment, the patient has any advanced organ dysfunction that would not make participation appropriate.

1.1.7. *Study Intervention*

Therapeutic agents will be evaluated as an add-on therapy to SoC. Participants will be randomly assigned to receive either SoC plus one of the test agents or SoC plus placebo. Randomization will proceed in two stages. At the first stage, each participant will be assigned to one of the sub-studies with equal probability. At the second stage, each participant will be assigned to either the test agent or its matching placebo in an n:1 ratio, where n = the number of agents currently active in the master protocol and for which the patient is eligible to receive. With three agents and SoC, for example, this procedure results in a randomization ratio of 1:1:1:1 if the patient is eligible to receive all three agents (see Section 6.2).

Inclusion of a placebo for each agent enables masking of study participants and clinical personnel to treatment assignment at the second stage. Data from patients receiving SoC plus placebo will be pooled across agents for comparative analyses and hypothesis testing.

If there are supply limitations on any product, the sub-study containing that product, or its matching placebo will be temporarily closed to enrollment.

The initial SoC will be remdesivir, based on the results of the ACTT-1 study. Participants will receive SoC as follows:

- Remdesivir will be administered as a 200 mg intravenous (IV) loading dose on Day 1, followed by a 100 mg once-daily IV maintenance dose for the duration of the hospitalization up to a 10-day total course.

Duration of SoC therapy:

- Remdesivir IV component – 5 to 10 days while hospitalized (see Section 6.1.2).

1.1.8. *Schedule of Assessments*

In addition to the table below, please consult the appropriate appendix for each sub-study (Section 1.8 of Appendices 1, 2, and 3) for additional agent- and sub-study-specific assessments.

Table 1-1. Schedule of Assessments (SOA)

	Screen	*Baseline*	*Study Intervention Period*	*Follow-up Visits*			
**Day +/- Window**	**−1 or 1**	**1**	**Daily until hospital discharge***	**15[7] ± 2**	**22[7] ± 3**	**29[7] ± 3**	**60[12] ± 5**
**ELIGIBILITY**							
Informed consent	X						
Demographics & Medical History	X						
Review SARS-CoV-2 results	X						
**STUDY INTERVENTION**							
Randomization		X					
Administration of investigational agent		• Remdesivir+placebo: daily infusion until Day 10 or discharge.					
**STUDY PROCEDURES**							
Targeted physical exam	X						
Vital signs including SpO_2 [1]		X[4]	Daily until discharge[8,9]	X[9]		X[9]	
Clinical data collection [2]		X[4]	Daily until discharge[8,9]	X[9]	X[9]	X[9]	X[12]
Adverse event evaluation		X[4]	Daily until discharge[8,9]	X[9]	X[9]	X[9]	X[12]
Concomitant medication review		X[4]	Daily from Day -7 to Day 15 or until discharge[9]	X[9]			
**SAFETY LABORATORY**							
Safety hematology, chemistry and liver tests	X[3]	X[4,5,6,7]	Day 3, 5, 8, 11 (all ± 1 day) if hospitalized[6,7,9]	X[9]		X[9]	
Pregnancy test for females of childbearing potential	X[3]						
**PHARMACOKINETICS**							
Blood sample for pharmacokinetics[11]		X[11]	Day 8			X[11]	
**RESEARCH LABORATORY**							
Blood for serum and plasma (future secondary research)		X[4,10]	Day 3, Day 8 (all ± 1 day) if hospitalized	X[9]		X[9]	

Notes:

* *If participant is discharged from hospital a telehealth visit will be performed on Day 8 and 11 where applicable (without required blood draws) with assessment of clinical and vital status using the 8-point scale*

[1] *Vital signs include temperature, systolic blood pressure, heart rate, respiratory rate, O_2 saturation and level of consciousness. Vital signs collected as part of standard care may be used.*

[2] *Refer to Section 8.1.2 and 8.1.3 of the protocol for details of clinical data to be collected including ordinal score, NEWS, oxygen requirement, mechanical ventilator requirement, extrapulmonary manifestations, etc.*

[3] *Screening laboratory tests include: CBC with differential (including absolute neutrophil count and absolute lymphocyte count), ALT, AST, ALP, total bilirubin, direct bilirubin, creatinine (and calculate an estimated glomerular filtration rate (eGFR); the formula used is determined by the sites, but should be consistent throughout the study at each site), and*

pregnancy test. Laboratory tests performed in the 48 hours prior to enrollment will be accepted for determination of eligibility.

[4] *Baseline assessments should be performed within 24 hours prior to first study product administration. Results of Day 1 (baseline) laboratory assessment do not need to be reviewed to determine if initial study product should be given.*

[5] *Laboratory tests performed as part of routine clinical care in the 24 hours prior to first dose will be accepted for the baseline safety laboratory tests. Baseline may be the same as the screening laboratory tests.*

[6] *Safety laboratory tests include WBC with differential, hemoglobin, platelets, creatinine (with calculated eGFR), glucose, total bilirubin, direct bilirubin, ALP, ALT, AST, CPK-MB, Troponin, PT/INR, d-dimer, serum ferritin, and C-reactive protein.*

[7] *Any laboratory tests performed as part of routine clinical care within the specified visit window can be used for safety laboratory testing. Window during hospitalization is ±1 day.*

[8] *Daily until hospital discharge or end of study, whichever comes first.*

[9] *In-person visits are preferred but recognizing quarantine and other factors may limit the participant's ability to return to the site for the visit. In this case, the visit may be performed by phone. If participant is still hospitalized during the follow-up period, they should get Day 8, 11, 15, 22, and 29 assessments along with the daily clinical data collection.*

- *If still hospitalized at Day 8, 11, 15, and 29 or returns to the site for an in-person visit: collect clinical data, vital signs, safety laboratory tests, and research laboratory samples (serum/plasma) as able. Concomitant medications will only be assessed through Day 15.*
- *If phone call only on Days 8, 11, 15, and 29 and all Day 22 visits: assess adverse events, clinical status (ordinal scale), readmission to a hospital, and mortality only. Concomitant medications will only be assessed through Day 15.*

[10] *Serum and plasma collected will be stored for future assessment of biomarkers of immune function including cytokines, and chemokines that may be associated with inflammation, anti-viral response, cytokine response syndrome (CRS) and secondary hemophagocytic lymphohistiocytosis (sHLH) during SARS-CoV-2 infection and genetic markers predicting disease progression and/or response to specific therapy. Final decision about the specific biomarkers will be determined later.*

[11] *Pharmacokinetic samples will be collected on select days, see* ***Table 8-2****, Section 8.2 of Master Protocol including for details on timing of sample collection. If the patient is discharged prior to the day of PK sample collection, a sample should be collected just prior to discharge.*

[12] *Telephone assessment of clinical and vital status using the 8-point ordinal scale, of re-hospitalization, and of AEs.*

Study Schema

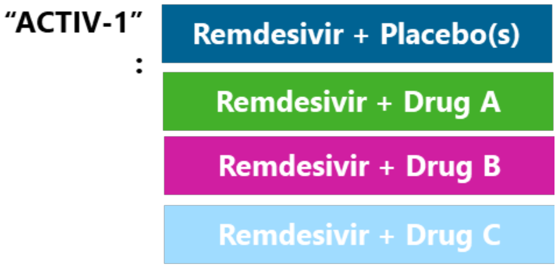

2. INTRODUCTION

2.1. Background

2.1.1. *ACTIV-1 IM – Immune Modulators & Remdesivir Multi-arm Trial*

A preliminary review of data from ACTT-1 occurring after 606 recoveries and 103 deaths (approximately 67% of the 1,063 subjects enrolled) demonstrated that subjects that received remdesivir had a 31% faster time to recovery (11 vs 15 days, recovery rate ratio 1.312 (1.119, 1.541), $p<0.001$), and a decrease in mortality (8.0% vs 11.6%, $p = 0.059$). The DSMB asked that the sponsor be unblinded early given the public health implications of the results as well as the implications for the design and conduct of ACTT-2. Based on the ACTT-1 results and subsequent Emergency Use Authorization (EUA) from FDA, remdesivir is now considered the standard of care for hospitalized COVID-19 patients. Note, however, that while remdesivir has demonstrated efficacy in the treatment of COVID-19, the mortality rate is still high. Infection by pathogenic coronaviruses (e.g. SARS and SARS-CoV–2) often results in excessive cytokine and chemokine action with the development of acute respiratory distress syndrome (ARDS) (33-35). It is postulated that this dysregulated inflammatory immune response is contributing to the excessive mortality and targeting this response will further improve outcomes.

2.2. Risk/Benefit Assessment

2.2.1. *Known Potential Risks of Study Participation*

Potential risks of participating in this trial are those associated with having blood drawn, the IV catheterization, possible reactions to the study interventions (as noted in Section 2.2.2.1), and breach of confidentiality.

Drawing blood may cause transient discomfort and fainting. Fainting is usually transient and managed by having the participant lie down and elevate his/her legs. Bruising at the blood collection sites may occur but can be prevented or lessened by applying pressure to the blood draw site for a few minutes after the blood is taken. IV catheterization may cause insertion site pain, phlebitis, hematoma formation, and infusate extravasation; less frequent but significant complications include bloodstream and local infections. The use of aseptic (sterile) technique will make infection at the site where blood will be drawn or at catheter site less likely.

2.2.1.1. *Risks to Privacy*

Participants will be asked to provide personal health information (PHI). All attempts will be made to keep this PHI confidential within the limits of the law. However, there is a chance that unauthorized persons will see the participant's PHI. All study records will be kept in a locked file cabinet or maintained in a locked room at the participating clinical site. Electronic files will be password protected. Only people who are involved in the conduct, oversight, monitoring, or auditing of this trial will be allowed access to the PHI that is collected. Any publication from this trial will not use information that will identify participants. Organizations that may inspect and/or copy research records maintained at the participating site for quality assurance and data analysis include groups such as the IRB, NCATS, and applicable regulatory agencies (e.g., FDA).

2.2.2. *SoC - Remdesivir*

2.2.2.1. *Potential Risks of Remdesivir*

Remdesivir is an investigational therapeutic agent. 138 healthy adults have been dosed with remdesivir in four Phase 1 clinical trials. Few subjects to date experienced constipation, heartburn, itching, unusual feelings in the ear, dizziness, loss of appetite, nausea, vomiting, shaking of the leg and arm, headache, loose stool, or upset stomach. These AEs were temporary, lasting only a few days, and none were serious. In clinical studies, transient elevations in ALT and AST have been observed with single doses of remdesivir up to 225 mg and multiple once daily doses of remdesivir 150 mg for up to 14 days. Mild (Grade 1) reversible PT prolongation was also noted in some subjects but without any clinically significant change in INR or other evidence of hepatic effects. The mechanism of these elevations is currently unknown. Based on these clinical observations, patients with ALT or AST >5 times the upper limit of normal will not be eligible for study enrollment. Regular laboratory assessments will be performed in order to monitor hepatic function and PT. Any observed liver function-related laboratory abnormalities or possibly related AEs will be treated appropriately and followed to resolution.

In nonclinical animal studies, toxicity studies found dose-dependent and reversible kidney injury and dysfunction. In clinical studies, no evidence of nephrotoxicity has been observed with single doses of remdesivir up to 225 mg or multiple once daily doses of remdesivir 150 mg for up to 14 days. A 150-mg dose of the solution and lyophilized formulations of remdesivir contains 9 g and 4.5 g, respectively, of sulfobutylether-beta-cyclodextrin (SBECD), for which the maximum daily recommended daily dose (based on a European Medicines Agency (EMA) safety review) is approximately 250 mg/kg. Because SBECD is renally cleared, participants with moderate or severe renal impairment may have SBECD exposures greater than those with less severe renal impairment or normal renal function. Based on this information, patients with an estimated glomerular filtration rate (eGFR) of less than 30 mL/min (including patients requiring hemodialysis or hemofiltration) will not be eligible for study enrollment.

Remdesivir is a substrate for CYP2C8, CYP2D6, and CYP3A4. However, coadministration with inhibitors of these CYP isoforms is unlikely to markedly increase remdesivir levels, as its metabolism is likely to be predominantly mediated by hydrolase activity. See IB for full discussion of clinical experience and risks.

There is the potential of the SARS-CoV-2 developing resistance to remdesivir, which could result in decreased efficacy. The clinical impact of the development of resistance is not clear at this time.

In vitro induction studies have demonstrated that a clinically relevant interaction with contraceptive steroids is considered to be of limited clinical significance. Therefore, the use of hormonal contraception with remdesivir is not recommended as the sole method for preventing pregnancy.

2.2.2.2. *Potential Benefits of Remdesivir*

A preliminary review of data from ACTT-1 occurring after 606 recoveries and 103 deaths (approximately 67% of the 1063 subjects enrolled) demonstrated that subjects that received remdesivir had a 31% faster time to recovery (11 vs 15 days, recovery rate ratio 1.312 (1.119, 1.541), p<0.001), and a decrease in mortality (8.0% vs 11.6%, p = 0.059). The DSMB asked that the sponsor be unblinded early given the public health implications of the results and implications for the design and conduct of ACTT-2. Based on the ACTT-1 results, remdesivir is now considered the standard of care for Covid-19 infected hospitalized patients. As a result, all subjects in ACTIV-1 IM will be given remdesivir as SoC, and the new therapeutic agents will be evaluated as add-on therapies to remdesivir.

This is a benefit to participation, though at some point, the new therapeutic agents evaluated under this master protocol may be available outside of clinical trials. In addition, society may benefit from their participation in this study resulting from insights gained about the efficacy of remdesivir combined with the new agents. Determining if additional clinical benefit can be realized by combining an antiviral with one of the new therapeutic agents being evaluated in ACTIV-1 IM for the treatment of COVID-19 may benefit society during this global COVID-19 pandemic.

2.2.2.3. *Assessment of Potential Risks and Benefits of Remdesivir*

Remdesivir is generally a well-tolerated medication. There are liver toxicities that have been observed in prior studies. These have been self-limited and resolved after cessation of the medication. There is the potential for renal

toxicities as observed in pre-clinical data. By excluding those with elevated liver transaminases and decreased kidney function (eGFR <30 mL/min or requires hemodialysis or hemofiltration), and appropriate monitoring during the study, we can minimize the risk to participants. While there may not be benefits for an individual participant, there may be benefits to society if a safe, efficacious therapeutic agent can be identified during this global COVID-19 pandemic. The potential risks therefore are thought to be acceptable given the potential benefits.

2.2.3. *Therapeutic Agents*

For each of the therapeutic agents under investigation, findings from the preclinical and clinical studies are briefly described in the agent-specific appendices, including a summary of the findings described in the Investigator Brochures (IBs). The potential risks and benefits of the therapeutic agents are also summarized in the appendices.

3. OBJECTIVES AND ENDPOINTS

The overall objective of the study is to evaluate the clinical efficacy and safety of different investigational therapeutic agents relative to the control arm among hospitalized adults who have COVID-19.

Table 3-1. Trial objectives and measured endpoints

OBJECTIVES	ENDPOINTS (OUTCOME MEASURES)
Primary	
To evaluate the clinical efficacy of different investigational therapeutics relative to the control arm in adults hospitalized with COVID-19 with respect to time to recovery by Day 29.	Day of recovery is defined as the first day on which the participant satisfies one of the following three categories from the ordinal scale:<br>• Hospitalized, not requiring supplemental oxygen – no longer requires ongoing medical in-patient care;<br>• Not hospitalized, limitation on activities and/or requiring home oxygen;<br>• Not hospitalized, no limitations on activities.<br>Recovery is evaluated up until Day 29.
Key Secondary	
• To evaluate the clinical efficacy of different investigational therapeutics relative to the control arm in adults hospitalized with COVID-19 according to:<br>• Clinical status (8-point ordinal scale) assessed on Day 15 and Day 29 for the previous day	1. Death;<br>2. Hospitalized, on invasive mechanical ventilation or ECMO;<br>3. Hospitalized, on non-invasive ventilation or high flow oxygen devices;<br>4. Hospitalized, requiring supplemental oxygen;<br>5. Hospitalized, not requiring supplemental oxygen – requiring ongoing in-patient medical care (COVID-19 related or otherwise);<br>6. Hospitalized, not requiring supplemental oxygen – no longer requires ongoing in-patient medical care (i.e., in hospital for social reasons, infection control, etc.);<br>7. Not hospitalized, limitation on activities and/or requiring home oxygen;<br>8. Not hospitalized, no limitations on activities.
• Mortality<br>o 14-day mortality	• Date and cause of death (if applicable)

OBJECTIVES	ENDPOINTS<br>(OUTCOME MEASURES)
○ 28-day mortality	
Additional Secondary	
To evaluate the clinical efficacy of different investigational therapeutics relative to the control arm as assessed by:<br>• **Clinical Severity**<br>○ Ordinal scale:<br>▪ Time to an improvement of one category and two categories from Day 1 (baseline) using an 8-point ordinal scale.<br>▪ Subject clinical status using 8-point ordinal scale at Days 3, 5, 8, 11, 15, 22, and 29.<br>▪ Mean change in the 8-point ordinal scale from Day 1 to Days 3, 5, 8, 11, 15, 22, and 29.<br>▪ Total severity score (TSS): 8-point ordinal scale summarized as a daily score (for days collected) averaged over time from Day 1 through Day 29	• Clinical outcome assessed using ordinal scale daily while hospitalized and on Days 15, 22, and 29.
○ Oxygenation:<br>▪ Oxygenation use up to Day 29.<br>▪ Incidence and duration of new oxygen use during the study.	• Days alive and free of supplemental oxygen (if applicable) up to Day 29
○ Non-invasive ventilation/high flow oxygen:<br>▪ Non-invasive ventilation/high flow oxygen use up to Day 29.<br>▪ Incidence and duration of new non-invasive ventilation or high flow oxygen use during the study.	• Days alive and free of non-invasive ventilation/high flow oxygen (if applicable) up to Day 29
○ Invasive Mechanical Ventilation / extracorporeal membrane oxygenation (ECMO):<br>▪ Ventilator / ECMO use up to Day 29.<br>▪ Incidence and duration of new mechanical ventilation or ECMO use during the study.	• Days alive and free of invasive mechanical ventilation/ECMO (if applicable) up to Day 29
• **Hospitalization**<br>○ Duration of hospitalization (days).	• Days alive and out of hospitalization up to Day 29
To evaluate clinical Status at Day 60	• 8-point Ordinal Scale
To evaluate the safety of different investigational therapeutics relative to the control arm as assessed by:<br>• Cumulative incidence of SAEs through Day 29.<br>• Cumulative incidence of Grade 3 and 4 clinical and/or laboratory AEs through Day 29.<br>• Discontinuation or temporary suspension of study product administrations (for any reason)	• SAEs<br>• Grade 3 and 4 AEs<br>• WBC with differential, hemoglobin, platelets, creatinine, glucose, total bilirubin, direct bilirubin, ALP, ALT, AST, CPK-MB, PT/INR, d-dimer,

OBJECTIVES	ENDPOINTS (OUTCOME MEASURES)
• Changes in white blood cell (WBC) count with differential, hemoglobin, platelets, creatinine, glucose, total bilirubin, alanine aminotransferase (ALT), aspartate aminotransferase (AST), prothrombin time (PT reported as INR), d-dimer, and C-reactive protein (CRP) over time (analysis of lab values in addition to AEs noted above).	troponin, serum ferritin, and CRP on Day 1; Days 3, 5, 8, and 11 (while hospitalized); and Days 15 and 29 (if attends in-person visit or still hospitalized).
To evaluate the impact of different investigational therapeutics relative to the control arm of extrapulmonary manifestations of COVID-19.	Incidence of individual and "any" specified manifestations at Day 29.
Exploratory	
o National Early Warning Score (NEWS):<br>▪ Time to discharge or to a NEWS of ≤ 2 and maintained for 24 hours, whichever occurs first.<br>▪ Change from Day 1 to Days 3, 5, 8, 11, 15, and 29 in NEWS.	NEWS assessed daily while hospitalized and on Days 15 and 29, if feasible.
Collect blood samples for future research.	Blood draws on Days 1, 3, 8, 15, and 29.

4. STUDY DESIGN

4.1. Overall Design

ACTIV-1 IM is a master protocol designed to evaluate multiple therapeutic agents for the treatment of moderately or severely ill hospitalized patients infected with COVID-19. Participants will be assessed daily while hospitalized. If the participants are discharged from the hospital, they will have a study visit at Days 8, 11, 15, 22, and 29. For discharged participants, it is preferred that the Day 8, 11, 15, and 29 visits are in person to obtain safety laboratory tests(Day 29) and blood (serum and plasma) samples for secondary research as well as clinical outcome data. However, infection control or other restrictions may limit the ability of the participant to return to the clinic. In this case, these visits may be conducted by phone, and only clinical data will be obtained. The Day 22 visit does not have laboratory tests or collection of samples and is conducted by phone. The Day 60 assessment will be conducted by phone. The primary outcome is time to recovery by Day 29 (see **Table 3-1** for definition of recovery based on the 8-point ordinal scale). Key secondary outcomes include treatment-related improvements in the 8-point ordinal scale at Day 15, evaluation of safety, and 14-day and 28-day mortality.

4.2. Scientific Rationale for Study Design

ACTIV-1 IM utilizes a master protocol to evaluate multiple therapeutic agents for the treatment of COVID-19 infection in hospitalized patients. Approximately 400 agents were identified and screened for clinical readiness by a panel of experts, and 170 agents were selected for further evaluation based on mechanism of action and need for further clinical testing (i.e., not previously studied or plan for study in other trials or trial networks). From this triaging exercise, three agents were initially selected for study in the ACTIV-1 IM master protocol. All three agents are immune modulators with substantial clinical experience available. Details about the initial set of selected agents are provided elsewhere (see Sections 2 and 4 and Appendices 1-3); additional agents may be entered into the ACTIV-1 IM master protocol after the study begins, depending on availability and network capacity. In that case, agent-specific information will be appended to the protocol for any new agents entering the study, and the randomization algorithm will be adjusted accordingly.

ACTIV-1 IM builds upon findings and the model used for ACTT-1 and -2. Including multiple therapeutic agents under a single protocol avoids duplication of effort in terms of infrastructure, trial governance, information systems

(EDC, web-based randomization, etc.) and other aspects of study management. Implementation of the master protocol facilitates discontinuation of less promising agents and addition of possibly newly emergent agents that become available after the study begins, without stopping and starting the study itself for extended pauses.

All test agents are evaluated as add-on therapies to the local SoC at each clinic. The master protocol design allows for the efficacy and safety of each agent to be determined based on comparisons with a pooled control group, consisting of patients receiving SoC plus placebo. Sharing control patients across all test agents substantially reduces the sample size requirements for the study.

4.3. Justification for Dose

4.3.1. *Justification for Dose of Remdesivir*

The dose of remdesivir used in this study will be the same dose that was used in the ACTT-1 and ACTT-2 trials.

4.3.2. *Justification for Dose of Investigational Agents*

The dose of each investigational agent is provided in the agent-specific appendices to this master protocol.

5. STUDY POPULATION

Male and non-pregnant female adults ≥18 years of age or older with COVID-19 and who meet all eligibility criteria will be enrolled at up to approximately 60 clinical trial sites globally. The target population should reflect the community at large. The estimated time from screening (Day -1 or Day 1) to end of study for an individual participant is approximately 60 days.

Subject Inclusion and Exclusion Criteria must be confirmed by any clinician named on the delegation log. If there is any uncertainty, the PI should make the decision on whether a potential participant is eligible for study enrollment. There is no exclusion for receipt of SARS-CoV-2 vaccine (experimental or licensed).

Following are the inclusion and exclusion criteria for the master protocol population. These criteria apply to all patients enrolled, regardless of therapeutic agent to be assigned for treatment. Any agent-specific exclusion criteria that are required will be applied during recruitment in addition to these common criteria and are described in the appendices corresponding to each agent under study.

5.1. Inclusion Criteria

1. Admitted to a hospital or awaiting admission in the ED with symptoms suggestive of COVID-19.
2. Subject (or legally authorized representative) provides informed consent prior to initiation of any study procedures.
3. Subject (or legally authorized representative) understands and agrees to comply with planned study procedures.
4. Male or non-pregnant female adults ≥18 years of age at time of enrollment.
5. Has laboratory-confirmed (within 14 days prior to enrollment) SARS-CoV-2 infection as determined by PCR or other commercial or public health assay in any specimen.
6. Ongoing illness of any duration, and at least one of the following:
   - Radiographic infiltrates by imaging (chest x-ray, CT scan, etc.), OR
   - Blood oxygen saturation (SpO_2) ≤94% on room air, OR
   - Requiring supplemental oxygen, OR
   - Requiring mechanical ventilation or ECMO.
7. Women of childbearing potential must agree to either abstinence or use at least one primary form of contraception not including hormonal contraception from the time of screening through Day 60.
8. Agrees to not participate in another intervention trial for the treatment of COVID-19 through Day 60.

5.2. Exclusion Criteria at Screening

1. ALT or AST >5 times the upper limit of normal.

2. Estimated glomerular filtration rate (eGFR) <30 mL/min (including patients receiving hemodialysis or hemofiltration).
3. Neutropenia (absolute neutrophil count <1000 cells/μL) (<1.0 x 10^3/μL or <1.0 GI/L).
4. Lymphopenia (absolute lymphocyte count <200 cells/μL) (<0.20 x 10^3/μL or <0.20 GI/L)
5. Pregnancy or breast feeding.
6. Anticipated discharge from the hospital or transfer to another hospital which is not a study site within 72 hours.
7. Known Allergy to any study medication.
8. Received cytotoxic or biologic treatments (such as anti-interleukin-1 [IL-1], anti-IL-6 [tocilizumab or sarilumab], IL-17, or T-cell or B-cell targeted therapies (e.g., rituximab), tyrosine kinase inhibitors including baricitinib, TNF inhibitors, or interferon within 4 weeks or 5 half-lives prior to screening. Steroid dependency defined as need for prednisone at a dose >10 mg (or equivalent) for >1 month within 2 weeks of screening is exclusionary. Note 1: Dexamethasone (at a dose of 6 mg per day for up to 10 days) is permitted for the treatment of COVID-19 in patients who are already mechanically ventilated and in patients who require supplemental oxygen at screening, but who are not mechanically ventilated in accordance with national guidelines. Note 2: Infusion of convalescent plasma is also allowed.
9. Based on medical history and concomitant therapies that would suggest infection, have suspected clinical diagnosis of current active tuberculosis (TB) or, if known, latent TB treated for less than 4 weeks with appropriate anti-tuberculosis therapy per local guidelines (by history only, no screening required).
10. Based on medical history and concomitant therapies that would suggest infection, suspected serious, active bacterial, fungal, viral (including, but not limited to, active HBV, HCV, or HIV/AIDS).
11. Have received any live vaccine (that is, live attenuated) within 3 months before screening, or intend to receive a live vaccine (or live attenuated) during the study. Note: Use of non-live (inactivated) vaccinations is allowed for all participants.
12. Severe hepatic impairment (defined as liver cirrhosis Child stage C).
13. Current severe heart failure (New York Heart Association [NYHA] III-IV).
14. In the Investigator's judgment, the patient has any advanced organ dysfunction that would not make participation appropriate.

5.3. Exclusion of Specific Populations

Children and adolescents will not be included in this trial. The SoC, remdesivir, has only been used in a small number of pediatric patients. Initial information about the epidemiology of COVID-19 indicates that the overwhelming burden of severe disease occurs among older adults, especially those with comorbidities. Given significant gaps in knowledge in this population, and a low incidence of severe morbidity/ mortality in children, and that none of the agents initially selected for study or likely to be selected in the future have had or will have demonstrated efficacy in COVID-19, this research is not known to have the prospect of direct benefit to individual child participants, and the risk/benefits do not warrant inclusion of this population into this trial at this time.

In nonclinical reproductive toxicity studies, the SoC, remdesivir, demonstrated no adverse effect on embryo-fetal development when administered to pregnant animals. Embryonic toxicity was seen when remdesivir was initiated in female animals prior to mating and conception, but only at a systemically toxic dose. Remdesivir has not been studied in pregnant women. Information on nonclinical reproductive toxicity studies for the therapeutic agents selected for study is provided in the agent-specific appendices.

In animal studies, remdesivir metabolites have been detected in the nursing pups of mothers given remdesivir. It is not known whether remdesivir is secreted in human milk. Because the effects of the SoC, remdesivir, on the breastfeeding infant is not known, and because the effects of one or more therapeutic agents selected for evaluation in this study may also be unknown, women who are breast feeding are not be eligible for the trial.

5.4. Inclusion of Vulnerable Subjects

Certain human subjects are categorized as vulnerable populations and require special treatment with respect to safeguards of their well-being. For this clinical trial, examples include cognitively impaired or mentally disabled persons and intubated individuals who are sedated. When it is determined that a potential research subject is cognitively impaired, federal and institutional regulations permit researchers to obtain consent from a legally

authorized representative (LAR). The study team will obtain consent from these vulnerable participants using an IRB-approved protocol- specific process for consent using a LAR.

For participants for whom a LAR gave consent, during the course of the study, if the subject regains the capacity to consent, informed consent must be obtained from the subject and the subject offered the ability to leave the study if desired.

5.5. Lifestyle Considerations

During this study, participants are asked to:

- Avoid getting pregnant during the study from Day 1 through Day 60.
- Agree not to participate in another interventional trial for the treatment of COVID-19 or SARS-CoV-2 through Day 60. This includes interventional trials that evaluate treatment of SARS-CoV-2 infection as well as the disease pathogenesis (e.g., treatment trials for the COVID-19-related thrombo-occlusive disease, respiratory complications, and dysregulated immune response to the virus). Co-enrollment for natural history studies of COVID-19 or SARS-CoV-2 infection is permitted; however, participation in both ACTIV-1 IM and natural history studies can only occur if the recommended blood collection volumes are not exceeded.

5.6. Screen Failures

Following consent, after the screening evaluations have been completed, the investigator or designee is to review the inclusion/exclusion criteria and determine the participant's eligibility for the study. If there is any uncertainty, the PI should make the decision on whether a potential participant is eligible for study enrollment.

Only basic demographic information and the reason(s) for ineligibility will be collected on screen failures. Prospective participants who are found to be ineligible will be told the reason(s) for ineligibility.

Individuals who do not meet the criteria for participation in this study (screen failure) because of an abnormal laboratory finding may be rescreened once.

5.7. Strategies for Recruitment and Retention

5.7.1. *Recruitment*

It is anticipated that patients with COVID-19 will present to participating hospitals, and that no external recruitment efforts towards potential participants are needed. Recruitment efforts may also include dissemination of information about this trial to other medical professionals / hospitals.

The hospitals that constitute the ACTIV-1 IM network span the United States and South America. As the pandemic migrates to different locations during the coming months, we anticipate being able to effectively follow the migration and focus recruitment efforts at hot spots through our network hospitals in different locales.

The IRB will approve the recruitment process and all materials provided prior to any recruitment to prospective participants directly.

Screening will begin with a brief discussion with study staff. Some will be excluded based on demographic data and medical history (i.e., pregnant, <18 years of age, renal failure, etc.). Information about the study will be presented to potential participants (or legally authorized representative) and questions will be asked to determine potential eligibility. Screening procedures can begin only after informed consent is obtained.

5.7.2. *Retention*

Retention of participants in this trial is very important for determining the primary endpoint of time to recovery by Day 29. As such, after hospital discharge, participants will be reminded of subsequent study visits and every effort will be made to accommodate the participant's schedule to facilitate follow-up within the specified visit window.

Additionally, there are many circumstances that influence the ability to obtain outcome information after discharge. Follow-up visits may be conducted by phone if in-person visits are not feasible.

5.8. Compensation Plan for Subjects

Compensation, if any, will be determined locally and in accordance with local IRB requirements, and subject to local IRB approval.

5.9. Costs

There is no cost to participants for the research tests, procedures/evaluations and study product while taking part in this trial. Procedures and treatment for clinical care including costs associated with hospital stay may be billed to the participant, participant's insurance, or third party.

6. STUDY PRODUCT

6.1. Study Product(s) and Administration

Participants will first be randomized to one of the agents currently active in the study, and then to either the agent or its matching placebo. The dosing regimen and administration of each agent and matching placebo are described in the agent-specific appendices.

If there are supply limitations on any product, the arms containing that product will be temporarily closed to enrollment and the corresponding placebo is not needed. Currently, the trial team anticipates no difficulty in obtaining remdesivir for the SoC for this study; however, if remdesivir supply should become limited for the trial, the treating physician should enroll the patient without remdesivir using local SoC.

6.1.1. *Study Product Description*

The SoC, remdesivir, is a single diastereomer monophosphoramidate prodrug designed for the intracellular delivery of a modified adenine nucleoside analog GS-441524. In addition to the active ingredient, the lyophilized formulation of remdesivir contains the following inactive ingredients: water for injection, SBECD, and hydrochloric acid and/or sodium hydroxide.

Descriptions of the therapeutic agents selected for evaluation in the study are provided in the agent-specific appendices.

6.1.2. *Dosing and Administration*

For the SoC, remdesivir all participants will receive remdesivir as a 200 mg intravenous (IV) loading dose on Day 1, followed by a 100 mg once-daily IV maintenance dose for 5 days during hospitalization for patients not requiring invasive mechanical ventilation and/or extracorporeal membrane oxygenation (ECMO). If a patient does not demonstrate clinical improvement, treatment may be extended for up to 5 additional days for a total treatment duration of up to 10 days. For patients requiring invasive mechanical ventilation and/or ECMO, the recommended total treatment duration is 10 days.

Dosing of the SoC and investigational agents should not need to occur at the same time. In fact, no physical biochemical compatibility studies have been conducted to evaluate the co-administration of the remdesivir with other investigational agents. Therefore, it should not be infused concomitantly in the same intravenous line with other agents; therefore, if infused together, separate lines should be used to alleviate confounding of attribution of possible infusion reactions or hypersensitivity events.

Any dose that is delayed may be given later that calendar day. Any dose that is missed (not given that calendar day) is not made up. The treatment course continues as described above even if the participant becomes PCR negative.

Dosing and duration of therapy of the therapeutic agents selected for study are described in the agent-specific appendices.

6.1.3. *Dose Escalation*

Not Applicable.

6.1.4. *Dose Modifications*

SoC (remdesivir):
If the eGFR decreases to an eGFR <25 mL/min, the remdesivir infusion should not be given on that day. The infusion may be resumed on the next day if the eGFR returns to ≥30 mL/min. If the participant's renal function worsens to the point that they require hemodialysis or hemofiltration, SoC will be discontinued.

If the ALT and/or AST increases to >5 times upper limits of normal, the infusion should be held and not be restarted until the ALT and AST ≤5 times upper limits of normal.

If any of the therapeutic agents selected for study require dose modifications based on biomarkers or safety signals, these adjustments are described in the agent-specific appendices

6.1.5. *Overdosage*

There is no known antidote for the SoC, remdesivir. In the case of overdose, the participant should receive supportive therapy based on the participant´s signs and symptoms. Availability of antidotes for study agents is discussed in the agent-specific appendices.

6.1.6. *Preparation/Handling/Storage/Accountability*

6.1.6.1. *Acquisition and Accountability*

Investigational products (IP) will be shipped to the site either directly from participating companies, from the Sponsor, or from other regional or local drug repositories. SoC (remdesivir) will be provided to the sponsor's designated distributor through an understanding with the manufacturer, and the sponsor's distributor will provide the SoC agent to the sites. All other supplies should be provided by the site. Multiple lots of each IP may be supplied.

Study products received at the sites will be open label and not kit specific, unless specified in the protocol-specific Manual of Procedures (MOP) or pharmacy manual. For IV infusion agents, drug preparation will be performed by the participating site's unblinded research pharmacist on the same day of administration to the participant. For oral agents, the investigational agent and the placebo will be shipped to the site ready to administer. The participating site's unblinded research pharmacist will provide either the investigational agent or the placebo pill to the treating physician at the time of assignment to the sub-study and randomization to the appropriate arm. See the MOP Appendices for detailed information on the preparation, labeling, storage, and administration of investigational products.

Accountability:

The site PI is responsible for study product distribution and disposition and has ultimate responsibility for study product accountability. The site PI may delegate to the participating site's research pharmacist responsibility for study product accountability. The participating site's research pharmacist will be responsible for maintaining complete records and documentation of study product receipt, accountability, dispensation, storage conditions, and final disposition of the study product(s). Time of study drug administration to the participant will be recorded on the appropriate data collection form (CRF). All study product(s), whether administered or not, must be documented on the appropriate study product accountability record or dispensing log. The Sponsor's monitoring staff will verify the participating site's study product accountability records and dispensing logs per the site monitoring plan. Refer to the protocol-specific MOP for details on storing study medications.

Destruction:

After the study treatment period has ended or as appropriate over the course of the study after study product accountability has been performed, used active and placebo product can be destroyed on-site following applicable site procedures with a second staff member observing and verifying the destruction.

Unused product at the end of the study for any of the agents selected for study should be saved until instructed by sponsor.

6.1.6.2. *Formulation, Appearance, Packaging, and Labeling*

SoC (remdesivir):

The lyophilized formulation of remdesivir is a preservative-free, white to off-white or yellow, lyophilized solid containing 150 mg or 100 mg of remdesivir to be reconstituted with 29 mL or 19 mL (respectively) of sterile water for injection respectively and diluted into IV infusion fluids prior to IV infusion. Following reconstitution, each vial contains a 5 mg/mL remdesivir concentrated solution with sufficient volume to allow withdrawal of 30 mL (150 mg of remdesivir) or 20 mL (100 mg of remdesivir).

It is supplied as a sterile product in a single-use, Type 1 clear glass vial. In addition to the active ingredient, the lyophilized formulation of remdesivir contains the following inactive ingredients: water for injection, SBECD, hydrochloric acid, and/or sodium hydroxide. For more information, refer to the MOP.

Remdesivir will be labeled according to manufacturer specifications and include the statement "Caution: New Drug Limited by Federal (USA) Law to Investigational Use."

Interventional Agents:

Formulation, appearance, packaging, and labeling for each of the agents selected for study are described in the agent-specific appendices.

6.1.6.3. *Product Storage and Stability*

SoC (remdesivir):

Ambient vials of the lyophilized formulation of remdesivir should be stored below 30°C. The lyophilized formulation needs to be reconstituted and then diluted into IV infusion fluids before use. After reconstitution, the total storage time before completion of administration (including any time before or after dilution) should not exceed 4 hours at room temperature (20°C to 25°C) or 24 hours at refrigerated temperature (2°C to 8°C). See MOP for additional information.

Interventional Agents:

Product storage and stability for each of the agents selected for study are described in the agent-specific appendices.

6.1.6.4. *Preparation*

Refer to the protocol-specific MOP for details about preparation.

SoC (remdesivir):

Remdesivir does not meet the criteria for a hazardous compound as defined by NISOH and ASHP hazard classification systems. The SoC may be prepared in a clean room but do not need to be prepared or handled in a fume hood.

Interventional Agents:

Preparation of each of the agents selected for study are described in the agent-specific appendices.

Measures that minimize drug contact with the body should always be considered during handling, preparation, and disposal procedures as indicated in the IB.

6.2. Measures to Minimize Bias: Randomization and Blinding

Randomization will be stratified by:

- Geographic Region
- Severity of illness at enrollment (by ordinal scale)
    - Severe disease:
        - Hospitalized, on invasive mechanical ventilation or ECMO, or
        - Hospitalized, on non-invasive ventilation or high flow oxygen devices.
    - Moderate disease:
        - Hospitalized, requiring supplemental oxygen, or
        - Hospitalized, not requiring supplemental oxygen.

Randomization will proceed in two steps, with stratification at each step. At the first step, each participant will be assigned with equal probability to one of the agents available at the time the patient is enrolled and for which the patient is eligible to receive, after applying any agent-specific safety exclusions. At the second step, each participant will be assigned to either the test agent or its matching placebo in an n:1 ratio, where n = the number of agents currently active in the master protocol and for which the patient is eligible to receive. With SoC and three agents active simultaneously, and the participant meets the criteria to receive all three agents, this procedure results in the randomization ratio of 1:1:1:1. If 3 agents are available, but a patient is only eligible to receive 2 of them, the allocation ratio at the first step will be 1:1, and at the second step will be 2:1 (agent vs placebo).Inclusion of a matching placebo for each agent enables masking of study participants and clinical personnel to treatment assignment at the second stage. Data from patients receiving SoC plus placebo will be pooled across agents for comparative analyses and hypothesis testing. Comparative analyses of a particular agent will include the subset of pooled placebo patients who enrolled concurrently with the new agent. That is, patients enrolled to control arm prior to a new agent entering the trial will not be included in comparative analyses of that newly added investigation agent.

Additional details of the randomization procedure will be described in the MOP.

6.3. Study Intervention Compliance

Each dose of study product will be administered by a member of the clinical research team who is qualified and licensed to administer the study product OR trained and qualified hospital personnel under the direction of the site investigator. Administration, including date and time, will be entered into the case report form (CRF).

6.4. Concomitant Therapy

6.4.1. *Permitted Concomitant Therapy and Procedures*

Therapy prior to enrollment with any other experimental treatment or off-label use of marketed medications that are intended as specific treatment for COVID-19 or the SARS-CoV-2 infection (i.e., post-exposure prophylaxis [PEP]) are permitted (except as detailed in the inclusion/exclusion criteria) but must be discontinued on enrollment. Another example: hydroxychloroquine is allowed prior to screening but must be discontinued. There is no waiting period between discontinuation of these treatments and administration of study products. However, these prior treatments and their end date should be documented on the Concomitant Medication (CCM) form.

Participants who are taking another immunosuppressive drugs for other medical conditions except as noted below as prohibited medications, may continue with the treatment. Agent specific appendices should be referenced for further prohibitions on concomitant therapies.

Local standard of care per written policies or guidelines for treatment for supportive care of COVID-19 or SARS-CoV-2 infection (i.e., not just an individual clinician decision) are permitted. This includes the evolving SoC with anticoagulant therapies. Dexamethasone (at a dose of 6 mg per day for up to 10 days) is permitted for the treatment of COVID-19 in patients who are mechanically ventilated and in patients who require supplemental oxygen but who are not mechanically ventilated in accordance with national guidelines. Infusion of convalescent plasma is also allowed if it is standard of care at the treating site.

VTE prophylaxis is recommended for all patients unless there is a major contraindication such as active bleeding events or history of heparin-induced thrombosis.

6.4.2. *Prohibited Concomitant Therapy*

A participant cannot participate in another clinical trial for the treatment of COVID-19 until after Day 60 (see exclusion criteria).

If there are NO written policies or guidelines for local standard of care, concomitant use of any other experimental treatment or off-label use of marketed medications intended as specific treatment for COVID-19 or SARS-CoV-2 infection are prohibited. This includes medications that target the host immune response.

Any biologic therapies are prohibited. This includes, but is not limited to, TNF inhibitor, interleukin-1[IL-1], IL-6 [tocilizumab or sarilumab], IL-17, or T-cell or B-cell targeted therapies, JAK inhibitor(s), interferon, or plasma or immunoglobulin (IgG) for COVID-19.

Dexamethasone, at a dose of 6 mg per day for up to 10 days, or other steroids in equivalent doses are permitted for the treatment of COVID-19 in patients who are mechanically ventilated and in patients who require supplemental oxygen but who are not mechanically ventilated in accordance with national guidelines. The use of corticosteroids at doses >10 mg/day outside of this indication is prohibited.

The use of chloroquine or hydroxychloroquine is prohibited.

The use of any biologics will be assessed for 4 weeks prior to screening to determine eligibility. Concomitant medications will be assessed only from 7 days prior to enrollment to Day 15 or upon discharge, whichever comes first. Report all prescription medications taken during this time period. Record medications once regardless of the number of times it was given during the time period. For example, vasopressors or insulin should be recorded when first started (the start date) and end date if ended before Day 15 or discharge. Record all antipyretics and other medications given for symptomatic care, if they are administered while an inpatient. However, record these medications only once, even if given multiple times, as needed during hospital course. Do not report medications that the participant did not actually receive during study (e.g., prn medications that were never given).

Any medication or vaccine (including over the counter, prescription medicines, vitamins, herbal supplements, and/or cannabis or other specific categories of interest) that the participant is receiving at the time of screening, has received in the 30 days prior to screening, or is anticipated to receive during the study must be recorded along with:

- Reason for use
- Dates of administration including start and end dates
- Dosage information including dose and frequency

The Sponsor's Medical Safety Physician or equivalent representative should be contacted if there are any questions regarding concomitant or prior therapy.

Due to potential interactions the additional medications maybe disallowed for specific agents on the trial as detailed in the sub-study appendices. If there is a medical need to utilize any of the medications disallowed, then the Investigator should discuss appropriate steps with the Sponsor.

6.4.3. *Rescue Medicine*

Not Applicable.

6.4.4. *Non-Research Standard of Care*

Not Applicable.

7. STUDY INTERVENTION DISCONTINUATION AND SUBJECT DISCONTINUATION/WITHDRAWAL

7.1. Halting Criteria and Discontinuation of Study Intervention

7.1.1. *Individual Study Product Halting*

Study product administration for any given participant may be stopped for SAEs, clinically significant adverse events, severe laboratory abnormalities, or any other medical conditions that indicate to the Investigator that continued dosing is not in the best interest of the patient.

In addition, a participant in this clinical study may discontinue study drug at their request for any reason. Every effort should be made to encourage participants to remain in the study for the duration of their planned outcome assessments. Participants should be educated on the continued scientific importance of their data, even if they discontinue study drug.

Unless the participant withdraws consent, those who discontinue study drug early will remain in the study. The reason for participant discontinuation of study drug should be documented in the case report form.

See Section 6.1.4. for information about dosing modifications due to laboratory abnormalities.

7.1.2. *Study Halting*

There will be close oversight by the protocol team and frequent DSMB reviews of the safety data. The DSMB may recommend halting one or more of the therapeutic agents due to safety, futility, or efficacy issues observed during the study, or they may recommend halting the study as a whole, if a safety issue or issue relating to the COVID-19 pandemic that negatively impacts study continuation arises.

A sub-study will be paused for new enrollment/study drug administration and will be reviewed by the DSMB if the following rule is met:

- After a sub-study has enrolled at least 10% of its planned study population, the proportion of study participants who die within 14 days of study drug administration or first dose administration in the same sub-study exceeds 24% (based on the 11.9% death rate in the ACTT-1 placebo arm; Beigel et al. 2020).

7.2. Withdrawal from the Study

Participants are free to withdraw from participation in the study at any time upon request, without any consequence. Participants should be listed as having withdrawn consent only when they no longer wish to participate in the study and no longer authorize the Investigators to make efforts to continue to obtain their outcome data.

Participants who withdraw from this study or are lost to follow-up after signing the informed consent form (ICF) and administration of the study product, will not be replaced. The reason for participant withdrawal from the study will be recorded on the appropriate CRF.

7.3. Lost to Follow-Up

A participant will be considered lost to follow-up if he or she fails to appear for all follow-up assessments. In lost to follow-up cases, attempts to contact the participant should be made and these efforts should be documented in the participant's records.

7.4. Readmission

If a participant is discharged from the hospital and then readmitted prior to Day 14, they may be given the remainder of the study product (i.e., remdesivir for up to a total of 10 days). If the participant did not withdrawal his/her consent to participate in the study, there is no need to reconsent upon readmission to the study hospital. However, the site will need to inform them that since he/she was readmitted, study product administration will resume and confirm that they still agree to receive study product. If the participant is re-admitted with diminished mental capacity, the site should discuss continued study participation with a LAR.

The site should not complete the Discontinuation of Treatment form since the participant came back to the study hospital to be readmitted. For all data collection procedures required for those readmitted, please see the MOP.

8. STUDY ASSESSMENTS AND PROCEDURES

8.1. Screening and Efficacy Assessments

8.1.1. *Screening Procedures*

Screening procedures may be done over one to two calendar days (from Day -1 to Day 1). However, in many cases all the screening assessments can be done in less than 24 hours. If that is the case, Day 1 pre-study product administration baseline assessments, specimen collection and the initial study product administration can occur on the same calendar day as the screening procedures.

After the informed consent, the following assessments are performed to determine eligibility and obtain baseline data:

- Confirm the positive SARS-CoV-2 test result (per inclusion criteria).
- Take a focused medical history, including the following information:
  - Day of onset of COVID-19 signs and symptoms.
  - History of vaccinations within 4 weeks before screening and planned vaccinations.
    - Exclusionary vaccine history includes:
      - Has received any live vaccine (that is, live attenuated) within 4 weeks before screening, or intend to receive a live vaccine (or live attenuated) during the study. Note: Use of non-live (inactivated) vaccinations is allowed for all participants.
  - History of chronic medical conditions including chronic oxygen requirement prior to onset of COVID-19. See conditions included in exclusion criteria (Section 5.2) and on the Medical History (CMX) data collection form.
    - Exclusionary medical history includes:
      - Has diagnosis of current active tuberculosis (TB) or, if known, latent TB treated for less than 4 weeks with appropriate anti-tuberculosis therapy per local guidelines (by history only, no screening required).
      - Suspected serious, active bacterial, fungal, viral, or other infection (besides COVID-19) that in the opinion of the investigator could constitute a risk when taking investigational product.
  - History of medication allergies.
  - Medications and therapies for this current illness taken in the 7 days prior to Day 1 and history of any medication listed in the exclusion criteria.
    - Exclusionary medication use includes:
      - Received cytotoxic or biologic treatments (such as anti-interleukin-1 [IL-1], anti-IL-6 [tocilizumab or sarilumab], or T-cell or B-cell targeted therapies (e.g., rituximab), tyrosine kinase inhibitors including baricitinib, or interferon within 4 weeks or 5 half-lives prior to screening.
      - Received TNF inhibitors within 4 weeks prior to screening.

- Currently receiving corticosteroids at high doses (i.e., prednisone >10 mg per day or equivalent) for >1 month within 2 weeks of screening. Dexamethasone (at a dose of 6 mg per day for up to 10 days) is permitted for the treatment of COVID-19 in patients who are mechanically ventilated and in patients who require supplemental oxygen but who are not mechanically ventilated in accordance with national guidelines.
- Ask if they are participating in another interventional trial or plan to enroll in another clinical trial in the next 60 days.

- Women of childbearing potential should be counseled to either practice abstinence or use at least one primary form of contraception not including hormonal contraception from the time of screening through Day 60. Women should be confirmed to not be breastfeeding.
  - Note: If a woman is either postmenopausal (i.e., has had ≥12 months of spontaneous amenorrhea) or surgically sterile (i.e., has had a hysterectomy, bilateral ovariectomy (oophorectomy), or bilateral tubal ligation), she is not considered to be of childbearing potential.
- Height and weight (height can be self-reported; weight can be self-reported if not feasible to assess).
- Results of recent radiographic imaging (x-ray or CT scan).
- SpO_2.
- Blood for screening laboratory evaluations if not done as part of routine clinical care in the preceding 48 hours:
  - CBC with differential
    - Evaluate if neutropenic (absolute neutrophil count <1000 cells/µL) (<1.0 x 10^3/µL or <1.0 GI/L) and/or lymphopenic (absolute lymphocyte count <200 cells/µL) (<0.20 x 10^3/µL or <0.20 GI/L)
  - ALT and AST.
    - Assess if ALT or AST >5 times the upper limit of normal.
  - Creatinine (and calculate eGFR).
    - Determine if eGFR <30 mL/min or receiving hemodialysis or hemofiltration.
    - Any automated calculation by the clinical laboratory or published formula for this calculation is acceptable. The site should select a formula to be used for all participants enrolled at the site for the duration of the study.
- Urine or serum pregnancy test (in women of childbearing potential).
- Clinical screening laboratory evaluations will be performed locally by the site laboratory. The volume of venous blood to be collected is presented in **Table 8-2**.
- A targeted physical examination will be performed at screening (Day -1 or Day 1) prior to initial study product administration on Day 1. No routine physical exam is needed for study visits after Day 1.
  - Study staff at some sites are not allowed into the participants' rooms due to a limited supply of PPE and the need for strict respiratory isolation measures for COVID-19 patients. Because of limited access to participants, physical exams can be performed by any licensed provider at the study hospital even if they are not study staff listed on the 1572. The study team can extract information from the hospital chart or EMR.

The overall eligibility of the participant to participate in the study will be assessed once all screening values are available. The screening process can be suspended prior to complete assessment at any time if exclusions are identified by the study team.

8.1.2. *Efficacy Assessments*

For all baseline assessments and follow-up visits, refer to the Schedule of Assessments (SOA) for procedure to be done, and details below for each assessment.

Measures of clinical support, limitations and infection control

The participant's clinical status will be captured on each study day while hospitalized through Day 29. If a participant is discharged prior to Day 15, clinical status is collected on Day 8, Day 11, Day 15, and Day 29 if the participant returns for an in-person clinic visit or by phone if an in-person visit is not possible. Clinical status will also be captured on Day 22 during a phone visit. Clinical status is largely measured by the 8-point ordinal scale and the NEWS. Unlike the NEWS, the ordinal scale can also be evaluated over the phone if the discharged participant is unable to return for visits on Day 8, 11, 15, or 29 as well as on Day 22.

Except for on Day 1, when the ordinal scale and the components of NEWS are is captured at the time of randomization, a site should try to complete the ordinal scale and the components of NEWS at approximately the same time each day. Ideally, complete the ordinal scale concurrently with components of NEWS just prior to study product administration, as time permits. The following measures are recorded for the ordinal scale:

- Hospitalization.
- Oxygen requirement.
- Non-invasive mechanical ventilation (via mask) requirement.
- High flow oxygen requirement.
- Invasive mechanical ventilation (via endotracheal tube or tracheostomy tube) requirement.
- ECMO requirement.
- Ongoing medical in-patient care preventing hospital discharge (COVID-19 related or other medical conditions).
- Limitations of physical activity (self-assessed).
- Isolated for infection control purposes.

Ordinal Scale

The ordinal scale is the basis for the primary and key secondary clinical outcomes in the study, namely, time to recovery, improvement in disease severity, and mortality.

The scale used in this study is as follows (from worst to best):

- Death;
- Hospitalized, on invasive mechanical ventilation or ECMO;
- Hospitalized, on non-invasive ventilation or high flow oxygen devices;
- Hospitalized, requiring supplemental oxygen;
- Hospitalized, not requiring supplemental oxygen – requiring ongoing medical in-patient care (COVID-19 related or otherwise);
- Hospitalized, not requiring supplemental oxygen – no longer requires ongoing medical in-patient care;
    - This would include those kept in hospital for quarantine/infection control/social reasons, awaiting bed in rehabilitation facility or homecare, etc.
- Not hospitalized, limitation on activities and/or requiring home oxygen;
- Not hospitalized, no limitations on activities

To determine a participant's clinical status using the ordinal scale: On Day 1, report their clinical status at randomization. After Day 1, collect the ordinal scale daily while hospitalized from Day 2 through Day 29 by providing the worst clinical assessment for the previous day (i.e., midnight to midnight; 00:00 – 23:59 [24-hr clock]). For those who are discharged prior to Day 15, collect ordinal scale on follow-up Days 8, 11, 15, 22, and 29 by providing the worst clinical assessment for the previous day (i.e., midnight to midnight; 00:00 – 23:59 [24-hr clock]). For example, on study Day 3 when completing the form, the worst clinical outcome measure of Day 2 is captured with the worst being death followed by ECMO, mechanical ventilation, etc. The Day 2 measurement is assessed as occurring anytime in that 24-hour period (00:00 to 23:59). For more information about the data collected for the ordinal scale, see the MOP.

Extrapulmonary manifestations

The presence of the extrapulmonary manifestation of disease during the course of hospitalization will be assessed and reported at discharge. Specifically, clinical organ failure defined by development of any one or more of the following clinical events (see Protocol Information Manual) for criteria for what constitutes each of these conditions):

a. Respiratory dysfunction:
   1. Respiratory failure defined as receipt of high flow nasal oxygen, noninvasive ventilation, invasive mechanical ventilation or ECMO

b. Cardiac and vascular dysfunction:
   1. Myocardial infarction
   2. Myocarditis or pericarditis
   3. Congestive heart failure: new onset NYHA class III or IV, or worsening to class III or IV
   4. Hypotension requiring institution of vasopressor therapy

c. Renal dysfunction:
   1. New requirement for renal replacement therapy

d. Hepatic dysfunction:
   1. Hepatic decompensation

e. Neurological dysfunction
   1. Acute delirium
   2. Cerebrovascular event (stroke, cerebrovascular accident [CVA])
   3. Transient ischemic events (i.e., CVA symptomatology resolving <24 hrs)
   4. Encephalitis, meningitis or myelitis

f. Hematological dysfunction:
   1. Disseminated intravascular coagulation
   2. New arterial or venous thromboembolic events, including pulmonary embolism and deep vein thrombosis
   3. Major bleeding events (>2 units of blood within 24 hours, bleeding at a critical site (intracranial, intraspinal, intraocular, pericardial, intraarticular, intramuscular with compartment syndrome, or retroperitoneal), or fatal bleeding).

g. Serious infection:
   1. Intercurrent, at least probable, documented serious disease caused by an infection other than SARS-CoV2, requiring antimicrobial administration and care.

Longer term follow-up

Information on vital and clinical status and occurrence and duration of rehospitalization will be captured by phone on Day 60.

8.1.3. *Exploratory Assessments*

Blood for serum and plasma (for secondary research)

- If the samples can be processed and but not sent to the repository, the samples may be stored locally.

National Early Warning Score (NEWS)

Vital signs and other clinical assessments are collected for the calculation of the NEWS, and include temperature, systolic blood pressure, heart rate, respiratory rate, O_2 saturation and level of consciousness. Vital signs collected per standard of care can be used. NEWS has demonstrated an ability to discriminate subjects at risk of poor outcomes. (Smith, 2016). This score is based on 7 clinical parameters (see **Table 8-1**). The NEWS is being used as

an exploratory efficacy measure. The components of NEWS should be evaluated daily while hospitalized. It can be performed concurrently with the Ordinal Scale. This should be evaluated at a consistent time for each study day and prior to administration of study product. The 7 parameters can be obtained from the hospital chart or electronic medical record (EMR) using the last measurement prior to the time of assessment (including parameters collected prior to the time of consent) and a numeric score is given for each parameter (e.g., a RR of 9 breaths per minute is one point, oxygen saturation of 92% is two points). This is recorded for the day obtained (i.e., on Day 3, the vital signs and other parameters from Day 3 will be used to obtain NEWS for Day 3). ECMO and mechanically ventilated participants should be assigned a score of 3 for RR (RR ≤8 breaths per minute) regardless of the ventilator setting. Participants on ECMO should get a score of 3 for heart rate since they are on cardiopulmonary bypass.

Table 8-1. National Early Warning Score (NEWS)

PHYSIOLOGICAL PARAMETERS	3	2	1	0	1	2	3
Respiration Rate (breaths per minute)	≤8 [a]		9 – 11	12 – 20		21 – 24	≥25
Oxygen Saturation (%)	≤91	92 – 93	94 – 95	≥96			
Any Supplemental Oxygen		Yes		No			
Temperature (°C)	≤35.0		35.1 – 36.0	36.1 – 38.0	38.1 – 39.0	≥39.1	
Systolic Blood Pressure (mm Hg)	≤90	91 – 100	101 – 110	111 – 219			≥220
Heart Rate (beats per minute)	≤40 [b]		41 – 50	51 – 90	91 – 110	111 – 130	≥131
Level of Consciousness				A			V, P, U

Level of consciousness = alert (A), and non-alert and arousable only to voice (V) or pain (P), and unresponsive (U).
[a] *If the participant is on ECMO or invasive mechanical ventilation, they will be given a score of 3 (≤8 breaths per minute) for respiratory rate regardless of ventilator setting.*
[b] *Participants on ECMO will also receive a score of 3 (≤40 beats per minute) for heart rate.*

8.2. Safety and Other Assessments

Study procedures are specified in the SOA. A study physician licensed to make medical diagnoses and listed on the 1572 will be responsible for all trial-related medical decisions.

Clinical laboratory evaluations:

- Fasting is not required before collection of laboratory samples.
- Blood will be collected at the time points indicated in the SOA.
    - Clinical safety laboratory tests include WBC, differential, Hgb, PLT, creatinine, glucose, total bilirubin, direct bilirubin, AST, ALT, ALP, PT/INR, d-dimer, CPK-MB, cardiac troponin, serum

ferritin, and C-reactive protein. Sites that do not have access to a test for PT will be allowed to report an international normalized ratio (INR).
- Day 1 clinical laboratory evaluations are drawn prior to initial study product administration as a baseline and results do not need to be reviewed to determine if initial study product administration should be given. Note that Day 1 PK assessments need to be drawn after 1st dose of study drug, so will need to be drawn separately from Baseline lab assessments.

- Clinical laboratory testing will be performed at each clinical trial site in real time. PK samples will be analyzed at a central facility at a future date.

Table 8-2. Venipuncture Volumes[1]

	Screen	***Baseline***						
**Day +/- Window**	**−1 to 1**	**1 ± 1**	**3 ± 1**	**5 ±1**	**8 ± 1**	**11 ± 1**	**15 ± 2**	**29 ± 3**
Safety hematology, chemistry and liver tests	X 10mL[2]	X 10mL[2]	X 10mL[2]	X 10mL[2]	X 10mL[2]	X 10mL[2]	X[3] 10mL[2]	X[3] 10mL[2]
Blood for Serum and Plasma		X 18mL	X 18mL		X 18mL		X 18mL	X 18mL
PK Assessments [6]		X[4] 4mL			X[5] 4mL			X[5] 4mL
Total daily volume	10mL	32mL	28mL	10mL	32mL	10mL	28mL	32mL
Total all study days [7]								~182 mL[8]

PK = pharmacokinetic
1. See SOA in Section 1.1.8 for specific tests to be performed.
2. Total volume calculated assumes no further routine clinical laboratory done within 48 hours of screening that can be used for determining eligibility and no routine clinical laboratory tests were done within the window for that visit (±24 hours of Day 1, 3, 5, 8 and 11 and ±48 hours for Day 15 and ±72 hours for Day 29 if still hospitalized).
3. Safety laboratory tests will be collected on Days 8, 11, 15, and 29 if the participant is still hospitalized at these time points or if they return for an in-person outpatient visit and the site has the capacity to collect blood in the outpatient setting.
4. Single blood draw, separate from Day 1 Baseline labs, 3 hours post-dose (post-start of infusion or post-ingestion of oral drug), +/- 30 min. In patients that received an infusion, sample should be collected from the arm contralateral to that used for IV infusion.
5. a) If hospital discharge occurs prior to visit, then the PK blood sample should be collected at discharge; and, b) this sample may be drawn at the time of routine phlebotomy.
6. See specific footnotes for each blood draw in this row. Participants on ECMO may also have up to 4 additional PK samples (16 mL) taken from the ECMO circuit at each PK time point.
7. Total blood volumes may be lower if the participant is discharged early.
8. Participants on ECMO may provide a higher total volume over the course of the study (see footnote 6).

Additional PK blood collections from participants on ECMO:

ECMO has been repeatedly shown to substantially and unpredictably alter drug disposition (Wildschut et al. 2010; Watt et al. 2011). For ACTIV-1 IM participants supported with ECMO, the PK sampling scheme depicted in **Table 8-2** will be augmented with paired samples collected from the participant and the ECMO circuit at the same time.

If possible, and as clinically indicated, the following sections of the ECMO circuit would be sampled:

- Pre-oxygenator / pre-dialysis membrane or hemofilter
- Post-oxygenator / post-dialysis membrane or hemofilter
- Pre-oxygenator / post-dialysis membrane or hemofilter
- Post-oxygenator / pre-dialysis membrane or hemofilter

8.2.1. *Procedures to be Followed in the Event of Abnormal Laboratory Test Values or Abnormal Clinical Findings*

If a physiologic parameter (e.g., vital signs, or laboratory value) is outside of the protocol-specified range, then the measurement may be repeated once if, in the judgment of the investigator, the abnormality is the result of an acute, short-term, rapidly reversible condition or was an error. A physiologic parameter may also be repeated if there is a technical problem with the measurement caused by malfunctioning or an inappropriate measuring device (i.e., inappropriate-sized BP cuff).

8.2.2. *Unscheduled Visits*

If clinical considerations require the participant to be contacted or seen prior to the next schedule assessment to assure the participant's well-being, it is permissible in this protocol. However, no research data is collected at this visit.

8.3. Adverse Events and Serious Adverse Events

8.3.1. *Definition of Adverse Event (AE)*

AE means any untoward medical occurrence associated with the use of an intervention in humans, whether or not considered intervention related. An AE can therefore be any unfavorable and unintended sign (including an abnormal laboratory finding), symptom, or disease temporally associated with the use of medicinal (investigational) product. If multiple abnormalities are part of the same clinical syndrome, they can be reported together as one AE under a unifying clinical diagnosis.

Any medical condition that is present at the time that the participant is screened will be considered as baseline and not reported as an AE. However, if the severity of any pre-existing (baseline) medical condition increases above baseline to severity grade 3 or 4, it should be recorded as an AE.

Given the nature of severity of the underlying illness, participants will have many symptoms and abnormalities in vital signs and laboratory values. All Grade 3 and 4 AEs will be reported in this trial. In addition, the following AEs will be reported:

- Any Grade 2 suspected drug-related hypersensitivity reactions associated with study product administration will be reported as an AE.
- Any infection (other than COVID-19) at any time during the study.
- Any AEs leading to dose modification
- Any AEs leading to discontinuation from the study.

Intermittent abnormal laboratory values or vital sign measurements common in the severely ill populations (such as electrolyte abnormalities, low blood pressure, hyperglycemia, etc.) that are part of the same clinical diagnosis (e.g., uncontrolled diabetic) can be recorded once with the worst grade for each adverse event (grade 3 and 4 only for this trial), with the start and stops dates of the intermittent syndrome. If there is clear resolution of the event, and then recurrence, it should be treated as a separate adverse event. Resolution is defined as return to baseline (either normal if was normal at Day 1, or baseline (Day 1) grade if already an abnormality on the toxicity table at Day 1) for > 48 hours.

8.3.2. *Definition of Serious Adverse Event (SAE)*

An AE or suspected adverse reaction is considered serious (i.e., is an SAE) if, in the view of either the investigator or the Sponsor, it results in any of the following outcomes:

- Death;
- A life-threatening AE;
- Inpatient hospitalization or prolongation of existing hospitalization;

- A persistent or significant incapacity or substantial disruption of the ability to conduct normal life functions; or
- A congenital anomaly/birth defect.

Grade 4 AEs (potentially life-threatening events) are not always SAEs unless they are imminently life threatening.

Important medical events that may not meet the above criteria may be considered serious when, based upon appropriate medical judgment, they may jeopardize the participant and may require medical or surgical intervention to prevent one of the outcomes listed in this definition. Examples of such medical events include allergic bronchospasm requiring intensive treatment in an emergency room or at home, blood dyscrasias, convulsions that do not result in inpatient hospitalization, or the development of drug dependency or drug abuse.

"Life-threatening" refers to an AE that at occurrence represents an immediate risk of death to a participant. An event that may cause death if it occurs in a more severe form is not considered life-threatening. Similarly, a hospital admission for an elective procedure is not considered a SAE.

All SAEs, as with any AE, will be assessed for severity and relationship to study intervention.

All SAEs will be recorded on the AE CRF.

SAEs will only be reported to the designated pharmacovigilance group (see Section 8.3.6 for reporting procedure) if they are considered related to the study product (serious adverse reaction [SAR]).

All SAEs will be followed through resolution or stabilization by a licensed study physician (for IND studies, a physician listed on the Form FDA 1572 as the site PI or Sub-Investigator).

All SAEs will be reviewed and evaluated by the IND sponsor and will be sent to the DSMB (for periodic review), and the IRB/IEC. All secondary infections are regarded as adverse events of special interest (AESI) and will be reported to the DSMB.

8.3.3. *Suspected Unexpected Serious Adverse Reactions (SUSAR)*

A SUSAR is any SAE where a causal relationship with the study product is at least reasonably possible but is not listed in the Investigator Brochure (IB), Package Insert, and/or Summary of Product Characteristics.

8.3.4. *Classification of an Adverse Event*

The determination of seriousness, severity, and causality will be made by an on-site investigator who is qualified (licensed) to diagnose AE information, provide a medical evaluation of AEs, and classify AEs based upon medical judgment. This includes but is not limited to physicians, physician assistants, and nurse practitioners.

Severity of Adverse Events

All AEs and SAEs will be assessed for severity using the DAIDS Table for Grading the Severity of Adult and Pediatric Adverse Events, version 2.1 (July 2017).

For AEs not included in the Table, the following guidelines will be used to describe severity. In addition, all deaths related to an AE are to be classified as grade 5 according to the DAIDS Table.

- Moderate (Grade 2): Events that are usually alleviated with additional specific therapeutic intervention. The event interferes with usual activities of daily living and causes discomfort but poses no significant or permanent risk of harm to the research participant.
- Severe (Grade 3): Events that interrupt usual activities of daily living, or significantly affect clinical status, or may require intensive therapeutic intervention. Severe events are usually incapacitating.
- Potentially life-threatening event (Grade 4): Events that are potentially life threatening.
- Deaths (Grade 5): All deaths related to an AE are classified as grade 5 (per DAIDS Table).

Relationship to Study Intervention

For each reported adverse reaction, the PI or designee must assess the relationship of the event to the study product using the following guideline:

- Related – There is a temporal relationship between the study intervention and event, and the AE is known to occur with the study intervention or there is a reasonable possibility that the study intervention caused the AE. Reasonable possibility means that there is evidence to suggest a causal relationship between the study intervention and the AE.
- Not Related – There is not a reasonable possibility that the administration of the study intervention caused the event, there is no temporal relationship between the study intervention and event onset, or an alternate etiology has been established.

8.3.5. *Time Period and Frequency for Event Assessment and Follow-Up*

For this study, all Grade 3 and 4 AEs and all SAEs occurring from the time the informed consent is signed through the Day 60 ± 5-day telehealth visit will be documented, recorded, and reported. In addition, patients will be contacted at the end of the safety follow-up period required for each study agent (see appendices for details) to assess AEs and SAEs occurring after drug withdrawal.

Investigator Reporting of AEs

Information on AEs will be recorded on the appropriate CRF. All clearly related signs, symptoms, and results of diagnostic procedures performed because of an AE should be grouped together and recorded as a single diagnosis. Secondary infections will be recorded on the appropriate CRF. If the AE is a laboratory abnormality that is part of a clinical condition or syndrome, it should be recorded as the syndrome or diagnosis rather than the individual laboratory abnormality. Each AE will also be described in terms of duration (start and stop date), severity, association with the study product, action(s) taken, and outcome.

8.3.6. *Serious Adverse Event Reporting*

Investigator Reporting of SAEs

Any AE that meets a protocol-defined criterion as a treatment related SAE (SAR) must be submitted within 24 hours of site awareness via an SAE form to the Technical Resources, International (TRI) Pharmacovigilance Group, at the following address: TRISafetyOffice@tech-res.com.

Other supporting documentation of the event may be requested by the TRI Pharmacovigilance Group and should be provided as soon as possible. The TRI Medical Monitor will review and assess the SAE for regulatory reporting and potential impact on study participant safety and protocol conduct.

At any time after completion of the study, if the site PI or appropriate sub-investigator becomes aware of an SAE that occurred during the participant's participation in the study, the site PI or appropriate sub-investigator will report the event to the TRI Pharmacovigilance Group.

Regulatory Reporting of SAEs

Any event that requires expedited reporting to Regulatory Authorities (i.e, Serious Unexpected Suspected Adverse Reactions [SUSARs]) based on applicable national regulations will be forwarded to the IND sponsor in time to meet reporting requirements (e.g. 7 days for fatal and life-threatening initial reports, with follow up reports within another 8 days, 15 days for all other SUSARs). The IND sponsor or its in-country representative as detailed in the Transfer of Regulatory Obligations (TORO) will submit safety reports (e.g. IND safety reports) to the regulatory agencies as necessary, and will inform the investigators of such regulatory reports. Site investigators must submit safety reports as required by their Institutional Review Board (IRB)/Research Ethics Board (REB). Documentation of the submission and receipt by the IRB/REB must be retained for each expedited safety report.

SAEs that are not SUSARs will be reported to the FDA at least annually in a summary format which includes all SAEs.

Sites may have additional local reporting requirements (to the IRB and/or national regulatory authority).

8.3.7. *Reporting of Pregnancy*

Pregnancy is not an AE. However, any pregnancy that occurs during study participation should be reported to the Sponsor on the appropriate CRF. Pregnancies should also be reported to the IRB. The Sponsor will report pregnancies to the DSMB. Pregnancy should be followed to outcome.

8.4. Unanticipated Problems

8.4.1. *Definition of Unanticipated Problems*

An Unanticipated Problem (UP) is any event, incident, experience, or outcome that meets the following criteria:

- Unexpected in terms of nature, severity, or frequency given (a) the research procedures that are described in the protocol-related documents, such as the IRB-approved research protocol and informed consent document; and (b) the characteristics of the subject population being studied;
- Related to participation in the research (meaning there is a reasonable possibility that the incident, experience, or outcome may have been caused by the procedures involved in the research); and
- Suggests that the research places participants or others at a greater risk of harm (including physical, psychological, economic, or social harm) than was previously known or recognized.

8.4.2. *Unanticipated Problem Reporting*

To satisfy the requirement for prompt reporting, all UPs will be reported using the following timeline:

- UPs that are SAEs will be reported to the IRB and to the Statistical and Data Coordinating Center (SDCC)/study Sponsor within 24 hours of the investigator becoming aware of the event per the above described SAE reporting process.
- Any other UP will be reported to the IRB and to the SDCC/study Sponsor within 3 days of the investigator becoming aware of the problem.

9. STATISTICAL CONSIDERATIONS

The ACTIV-1 IM master protocol utilizes an adaptive platform trial design that allows for four types of adaptations: (1) early stopping of agents for futility, to allow more speedy accrual to other agents remaining in the trial; (2) early stopping for strong evidence of efficacy of a particular agent; (3) substitution of one of the test agents as standard of care for the remainder of the trial, should early evidence of superiority over SoC support such a change, and (4) addition of newly emergent agents, if they become available after study start. A brief summary is provided here. Details will be described in the statistical analysis plan (SAP).

The total sample size for ACTIV-1 IM is based on the Kaplan-Meier estimate of the probability of recovery from ACTT-1. Preliminary data indicate this estimate is approximately 73% based on over 1,000 patients enrolled.

Sample size requirements are based on the number of agents being evaluated and the ability to pool control patients for analysis. Initial sample size estimates are derived assuming three agents are ready for testing at study start and will remain in the study for evaluation at the final analysis stage. If newly emergent therapies are entered into the master protocol after study start, sample size requirements will be adjusted accordingly.

The statistical analysis strategy calls for sharing of control patients across all agents available for study when enrollment begins. For agents added to the trial after study start, comparative analyses will be limited to those control patients enrolled concurrently with the new agent. That is, control patients enrolled prior to an agent entering the trial will not be included in comparative analyses of that agent. This approach was used in the recent PALM study in patients with Ebola virus disease (Mulangu et al. 2019). Although sharing of non-concurrently randomized control patients has been used successfully in studies of chronic diseases (DIAN-TU;

https://clinicaltrials.gov/ct2/show/NCT01760005), it is not recommended here due to the evolving nature of the COVID-19 infection and treatment thereof. Exploratory analyses of the similarity between control patients enrolled early and late in the study, however, may be useful in informing the design of future master protocols in COVID-19.

If early evidence of superiority of any one of the test agents relative to SoC (plus placebo) is observed during the trial, consideration will be given to replacing the SoC with the test agent for the remainder of the trial. Such a change is dependent on the safety of combining the remaining test agents with this new SoC and will complicate the final data analyses somewhat. Comparative analyses will need to account for this change in SoC through stratification or other model-based methods. Details will be provided in the statistical analysis plan.

Preliminary reports of the completed RECOVERY Trial show that dexamethasone may reduce mortality in some hospitalized patients with COVID-19 (RECOVERY Collaborative Group, 2020). If dexamethasone or steroid, or other agents, become part of standard of care (SoC) or are used in a substantial number of patients, the observed potential benefit of immunomodulators may be altered. Nonetheless, ACTIV-1 IM is designed with high power to detect even a modest benefit of 25% for the primary outcome; attempting to detect smaller effects would cause a prohibitively large increase in sample size. Addition of dexamethasone or other agents to SoC may also affect the key secondary endpoint of mortality and alter the proportions of people in different cells of another key secondary endpoint, the 8-point ordinal scale. Accordingly, a blinded sample size re-estimation may be conducted at the 2nd interim analysis, after approximately one-half of the total number of expected recoveries has occurred. The purpose is to use the overall proportions of people in the different categories of the key secondary endpoint of the ordinal scale to recalculate power for that endpoint. If power for this key secondary endpoint is below 70%, the sample size might be increased, but by at most 25%. The sample size would not be reduced based on this potential re-estimation. More details will be provided in the SAP.

9.1. Statistical Hypotheses

The primary null hypothesis being tested is that time-to-recovery does not differ between each test agent and the pooled control group, consisting of patients receiving SoC plus placebo. The alternative hypothesis is that the test agent and pooled control group differ in time-to-recovery. If Drug A, B, and C denote the initial set of agents to be tested in ACTIV-1 IM, and S denotes patients receiving SoC plus placebo, then the primary hypotheses are:

H_{01}: $TR_A = TR_S$ vs H_{A1}: $TR_A \neq TR_S$

H_{02}: $TR_B = TR_S$ vs H_{A2}: $TR_B \neq TR_S$

H_{03}: $TR_C = TR_S$ vs H_{A3}: $TR_C \neq TR_S$

There are two key secondary endpoints that will be evaluated as supportive evidence:

1. The distribution of the 8-point ordinal scale at Day 15 and Day 29. For this, the parameter of interest is the "common odds ratio," which quantifies the shift in the severity distribution resulting from treatment. For an efficacious treatment, an odds ratio greater than 1 quantifies an improvement in disease severity; a value of 2 indicates a bigger improvement than a value of 1.25. The null hypothesis to be tested is that the odds of improvement on the ordinal scale is the same for the placebo and experimental treatment arms (i.e., the common odds ratio is 1). It is worth noting that, for large sample sizes, the test based on the proportional odds model is nearly the same as the Wilcoxon rank sum test.
2. Mortality will be evaluated with standard survival analysis techniques. Kaplan Meier curves and associated log-rank statistics will be generated to compare mortality between each test agent and SoC. Mortality will also be evaluated at Days 15 and 29.

9.2. Sample Size Determination

Primary endpoint: The Fine-Gray approach will be used to compare each test agent with SoC with respect to the cumulative incidence of recovery, accounting for the competing risk of mortality (Fine and Gray, 1999). The

approach is similar to using a log-rank test on time to recovery, retaining in the risk set people who die. The two key determinants of power are the total number of events (i.e., recoveries) E and the treatment-to-control ratio of the rate of recovery, RRR. Without accounting for futility monitoring, the number of events required for power $1 - \beta$ to detect a recovery rate ratio of θ using a two-tailed test at alpha=0.05 is approximately

$$E = \frac{4\left(1.96 + z_\beta\right)^2}{\{\ln(RRR)\}^2},$$

where z_β is the $100(1 - \beta)$th percentile of the standard normal distribution. The number of events must be increased to account for futility monitoring. For 85% power, the $1.96 + z_\beta$ in the above equation is replaced by 3.1312.

Table 9-1 provides the numbers of recoveries and of patients required to provide 85% power for a single pairwise comparison of test drug versus control assuming a 73% recovery rate and various recovery rate ratios (RRRs). Note that the rate of recovery is the analogue of the hazard for each test agent or control treatment, and the RRR is the analogue of the hazard ratio for a test agent relative to control in this setting. As can be seen from the table, approximately 347 recoveries are required to detect a 40% increase in the rate of recovery ($\theta = 1.40$) from control. An RRR of 1.40 is similar to, but slightly higher than the figure of 1.31 reported in Cao et al. (2020) for a lopinavir/ritonavir trial that used time to improvement by 2 categories as primary endpoint. A total of 436 recoveries is needed for an RRR of 1.35 with 85% power. Sample size requirements for ACTT-1 assumed an RRR of 1.35, but the study was over-enrolled (1,063 patients were enrolled, compared to N=572 needed) due to more rapid than anticipated accrual. Preliminary results from ACTT-1 show an RRR of 1.32 ([CI = [1.12,1.55]) for remdesivir vs placebo.

Table 9-1. Number of recoveries and number of patients needed for 85% power assuming a type I error rate of 5% for various recovery ratios

Recovery rate ratio (θ)	Number of recoveries needed for 85% power	Sample Size assuming 73% recovery rate (1 test agent vs SoC)
1.25	**788**	**1,080**
1.30	570	781
1.35	436	598
1.40	347	476

The objective of ACTIV-1 IM is to provide evidence of efficacy and safety of selected agents that would support regulatory approval of their use in treating moderately to severely ill hospitalized Covid-19 patients. It was decided that a modest effect as low as a 25% improvement in recovery rate attributable to any one of the test agents in the study would be of interest to detect. Consequently, the sample size requirements are based on the ability to detect an RRR of 1.25 for each agent relative to SoC.

A total of 788 recoveries is required for each comparison to provide approximately 85% power to detect a recovery rate ratio of 1.25 for the therapeutic agent relative to control, accounting for the interim analyses. Assuming 73% of participants achieve recovery in 28 days, consistent with the ACTT-1 results, the total sample size to compare 1, 2, or 3 agents to control in ACTIV-1 IM is approximately 1,080, 1,620, and 2,160, respectively. Because each agent is being compared to SoC with no between-agent comparisons currently planned, no multiplicity adjustments will be made, provided the number of agents assessed in ACTIV-1 IM is three or fewer. If more than three agents are evaluated, a minor multiplicity adjustment will be considered to aid in interpreting the results of the study.

Key secondary endpoints: A sample size can be computed using an (assumed) ordinal scale distribution for the control and the odds ratio representing clinical improvement. The odds ratio represents the odds of improvement in the ordinal scale for treatment relative to control [Whitehead, 1993]. The sample size to detect a given odds ratio for 1:1 randomization using a 2-tailed test at level α is given by

$$\frac{12\left(z_{\alpha/2}+z_{\beta}\right)^2}{\lambda^2\left(1-\sum_{i=1}^{K}p_i^3\right)},$$

where λ is the log odds ratio, p_i is the overall probability (combined over both arms) of being in the ith category of the K ordinal outcomes, and $z_{\alpha/2}$ and z_{β} are the $1-\alpha/2$ and $1-\beta$ quantiles of the standard normal distribution.

A sample size of 1,080 for an (active, control) pair gives approximately 95% or 85% power to detect an odds ratio of 1.50 (the observed odds ratio in the ACTT-1 trial) or 1.40 using a 2-tailed test at level $\alpha = 0.05$.

The 28-day mortality probability in the remdesivir arm of ACTT-1 was approximately 12%. Power for comparing 28-day mortality is 90% only for a 50% relative reduction (from 12% to 6%).

9.3. Populations for Analyses

The primary analysis will be based on an intention-to-treat population, including all participants randomized. Similarly, safety analyses will be based on a modified intention-to-treat population consisting of all participants who received at least one dose or injection of each drug administered in the randomization arm (e.g., test agent plus SoC). The primary analysis will be based on those participants enrolled in order to achieve 788 recoveries for each pairwise comparison as noted in section 9.1. Subsequent analysis will be performed on all enrolled participants.

9.4. Statistical Analyses

9.4.1. *General Approach*

This is a randomized, controlled, double-blinded trial testing a superiority hypothesis with respect to each therapeutic agent plus SoC versus SoC plus placebo with a two-sided Type I error probability of 5% for each agent. This is a randomized, controlled, double-blinded trial testing a superiority hypothesis with respect to each therapeutic agent plus SoC versus SoC plus placebo with a two-sided Type I error probability of 5% for each agent. Secondary hypotheses have been ordered according to relative importance, with key secondary hypotheses identified (corresponding to the odds ratio based on the 8-point clinical severity scale, and time to death). These will be described according to the appropriate summary statistics (e.g., proportions for categorical data, median for time-to-event data).

A statistical analysis plan will be developed and filed with the FDA prior to database lock and unblinding of treatment assignments.

Unblinding of study data for final analysis will occur for each test agent independently of other agents, consistent with the master protocol design. That is, once the planned number of recoveries required for a particular agent's comparative analysis are observed, study close-out procedures for that agent are applied for all study visits and data elements associated with the participants receiving the test agent as well as participants receiving SoC plus placebo during the randomization period for that agent (i.e., concurrently controlled participants). The study visits are monitored, data edits are completed, queries are resolved, database is locked, and treatment assignments are unmasked for that comparative analysis only. Note that, because control participant data are shared across agents, procedures for unmasking data for one agent's final analysis will be established to protect the ability to continue the ongoing study in a double-blinded manner for the remaining agents. It may be that recovery rates are similar enough across all agents in the study to enable a single study close-out with standard procedures. Any necessary modifications or updates to the statistical analysis should be made prior to the study unblinding.

9.4.2. *Analysis of the Primary Efficacy Endpoint*

The primary efficacy analysis for the comparison of each test agent plus SoC versus SoC plus placebo is a stratified test based on the Fine-Gray proportional hazards approach, where stratification is according to region and baseline disease severity (i.e. protocol defined mild/moderate vs severe disease). The method provides an estimate of the cumulative incidence of recovery while accounting for the competing risk of death. The hazard ratio can also be computed. With no censoring, the hazard rate in each arm can be thought of as the hazard for recovery, treating deaths as never having recovered (see section 2 of Fine and Gray, 1999). Every attempt will be made to complete

final clinic visits for patients dropping out of the study prematurely. For those unable to complete the final visit, data collection will be attempted via telephone interview. At a minimum, vital status will be obtained.

9.4.3. *Analysis of the Secondary Endpoint(s)*

1) The ordinal scale will be used to estimate a proportional odds model by disease strata. We will perform a stratified test to evaluate whether the common odds ratio for treatment is equal to one. The distribution of severity results will be summarized by treatment arm as percentages. Efforts to minimize loss-to-follow-up will be considerable. However, small amounts of missing data may occur. In such cases, participants without final outcome data will be excluded from the analysis. Sensitivity analyses will evaluate the impact of making different assumptions about missing observations. These analyses will be defined in the SAP.
2) When death is not a competing risk (for example, the endpoint includes death in the composite), differences in time-to-event endpoints by treatment will be summarized with Kaplan-Meier curves and 95% confidence bounds. When death is a competing risk (for example, time to at least a one-category improvement in ordinal scale, and time to at least a two-category improvement), the same competing risk approach will be used as for the primary analysis.
3) Change in ordinal scale at specific time points will be summarized by proportions (e.g., proportion who have a 1-, 2-, 3-, or 4-point improvement or 1-, 2-, 3-, 4-point worsening).
4) Differences in total severity score (TSS) over time will assessed by fitting a longitudinal mixed model to the 8-point clinical scale value on each day of assessment (in hospital plus Days 15, 22, 29). The average difference across days in study (analogous to the area under the severity curve) will be estimated with 95% confidence limits in the context of the longitudinal model.
5) Duration of event (e.g., duration of mechanical ventilation) will be summarized according to median days with quartiles.
6) Binary data (e.g., incidence of new oxygen use) will be summarized as a percent with 95% confidence intervals. Comparisons between arms will be presented as differences in proportions with 95% confidence intervals.
7) Categorical data (e.g., 28-day mortality or ordinal scale by day) may be summarized according to proportions by category and/or odds ratios with confidence intervals.

Procedures for handling missing data, including informative censoring (e.g., a missing duration of oxygen use endpoint due to a death), will be described in the statistical analysis plan.

9.4.4. *Safety Analyses*

Safety endpoints include death through Day 28 (as assessed at Day 29), SAEs, and Grade 3 and 4 AEs. These events will be analyzed univariately and as a composite endpoint. Time-to-event methods will be used for death and the composite endpoint. Each AE will be counted once for a given participant and graded by severity and relationship to COVID-19 or study intervention. AEs will be coded using the current version of the Medical Dictionary for Regulatory Activities (MedDRA). AEs will be presented by system organ class, duration (in days), start- and stop-date. Adverse events leading to premature discontinuation from the study intervention and serious AEs will be presented either in a table or a listing.

9.4.5. *Baseline Descriptive Statistics*

Baseline characteristics will be summarized by test agent versus control for each agent. For continuous measures the mean and standard deviation will be summarized. Categorical variables will be described by the proportion in each category (with the corresponding sample size numbers).

9.4.6. *Planned Early and Interim Analyses*

Data analyses will be conducted to monitor enrollment and follow-up rates and to summarize baseline characteristics throughout the course of the study. These early analyses will be conducted by the study team masked to treatment assignment. Summaries will be generated for each test agent separately. For agents entering the study at staggered

times, data summaries (pooling test agent and control data in blinded fashion) will incorporate data from concurrently randomized control participants only.

Unblinded interim analyses are planned to (i) assess the futility of each agent, with the goal of discontinuing those with lower probabilities of success to more effectively utilize trial resources for the remaining agents and (iii) review comparative analyses for each test agent to assess early stopping for efficacy. Alpha spending functions will be used to appropriately control the probability of making an erroneous conclusion across interim and final analyses at α = 0.05 (two-sided) for each agent.

A Data and Safety Monitoring Board (DSMB) will monitor ongoing results to ensure participant well-being and safety as well as study integrity. The DSMB will be asked to recommend early termination due to futility and early stopping for efficacy, only when stopping rules and conditions are clearly met. More details about the interim analyses are described in section 9.4.6.1 and 9.4.6.2 below as well as a separate guidance document for the DSMB.

Interim Safety Analyses

Safety analyses will evaluate Grade 3 and 4 AE and SAEs by treatment arm. Safety monitoring will be ongoing. The unblinded statistical team will prepare these reports for review by the DSMB.

Interim Analyses for Futility and Efficacy Review

Interim analyses for futility and efficacy are planned at three times during the study corresponding to the availability of approximately 25%, 50%, and 75% of total information. The planned randomization algorithm described above (Section 6.2) ensures equal allocation to each test agent or control (e.g., 1:1:1:1 for 3 test agents and control). It is anticipated that the interim analyses for each agent will occur at the same time, and the DSMB will make recommendations for all agents at their scheduled meetings. If recovery rates vary substantially by agent, however, it may be necessary to let the interim analysis times also vary by agent. Because Type I error probabilities are controlled for each, independently of the other agents, the need for additional DSMB reviews due to differential information accrual across agents should not pose any issues other than logistical ones.

The Lan-DeMets spending function analog of the O'Brien-Fleming boundaries will be used to monitor the primary endpoint as a guide for the DSMB. This spending function is conservative in that priority is given to preserving power for the final analysis with the use of stringent stopping rules early in the study.

In contrast, moderately aggressive stopping rules for futility will be implemented to promote early discontinuation of agents with low probabilities for success. The futility stopping rules will be considered non-binding by the DSMB in their review of interim data. The futility boundaries are computed based on the gamma family of spending functions $\alpha\{1-\exp(-\gamma t)\}/\{1-\exp(-\gamma)\}$ (Hwang et al. 1990). Figure 1 below illustrates the efficacy stopping boundaries based on the Lan-DeMets spending function (in blue shading) and the futility boundaries based on a Gamma (-2) spending function (in pink shading).

Figure 1: **Stopping Boundaries for Efficacy (blue shading) and Futility (pink shading).**

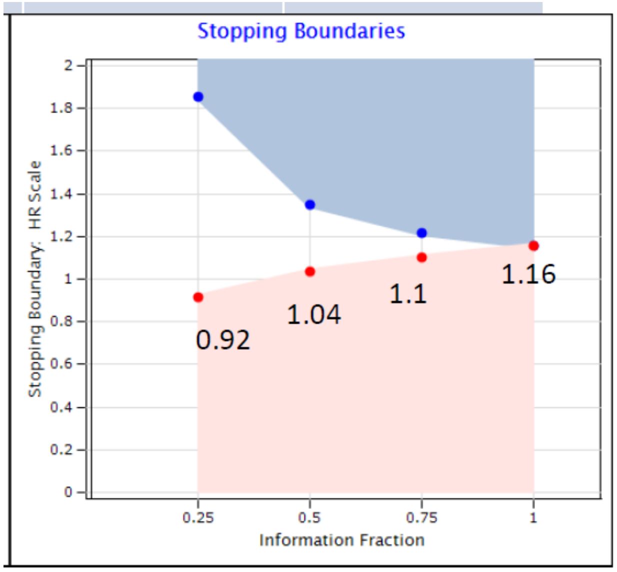

As can be seen from the figure, an RRR showing no more than a small amount of improvement in recovery time (RRR ≤ 1.04) will result in early discontinuation of the agent mid-way through information accrual in the study. When the null hypothesis is true (RRR = 1), the probability of discontinuing an agent at this point is 66%, but under the alternative, this probability is 4%. In simulations, the average number of recoveries under these efficacy and futility boundaries is 587, and the maximum sample size is approximately 1,080 participants.

The unblinded statistical team will prepare closed reports for each DSMB review meeting. Analyses will be presented with blinded codes for treatment arms to protect against the possibility that the DSMB report may fall into the wrong hands. Unblinding codes will be provided to DSMB members who wish to be unblinded. A DSMB charter will further describe procedures and membership. An additional document on statistical issues related to monitoring will be provided to the DSMB prior to the first interim analysis.

9.4.7. *Sub-Group Analyses*

Subgroup analyses for the primary outcomes will evaluate treatment effects across the following subgroups: geographic region, duration of symptoms prior to enrollment, baseline disease severity (stratification variable of mild/moderate and severe, as well as ordinal scale of 4/5 vs 6/7) age, race, sex and comorbidities. A forest plot will display confidence intervals across subgroups. Interaction tests will be conducted to determine whether the effect of treatment varies by subgroup. These subgroup analyses will be carried out separately for each test agent.

9.4.8. *Exploratory Analyses*

An exploratory analysis will compare treatment efficacy estimates for each agent according to the various scales outlined in section 8.1.3. Specifically, the probability of falling into category “i” or better will be compared between arms for each i. Exploratory analyses will also compare the difference between each test agent plus SoC and SoC plus placebo in TSS at each study day for which the clinical scale is administered (in-hospital and at Days 15, 22, and 29).

10. SUPPORTING DOCUMENTATION AND OPERATIONAL CONSIDERATIONS – ALL STAGES

All supporting documentation and operational considerations are applicable to the entire platform trial and are not unique to the individual stages. These are therefore covered in the main protocol document.

APPENDIX 1 – SUB-STUDY 1 – INFLIXIMAB (REMICADE)

1. SUB-STUDY SUMMARY

1.1. Sub-study overview

The sub-study population corresponds to moderately and severely ill patients infected with the COVID-19 virus. Recruitment will target patients already hospitalized for treatment of COVID-19 infection as well as patients being treated for COVID-19 infection in Emergency Departments while waiting to be admitted to the hospital. Patients both in and out of the ICU are included in the study population.

Enrollment will begin as soon as all elements are met for the sub-study to activate.

The study period is 29 days, with assessments on each day of the hospital stay. Patients will be followed after hospital discharge with periodic follow-up assessments through Day 29 and at Day 60.

1.2. Enrollment Period

Enrollment is expected to begin in September 2020. It is anticipated the enrollment may be completed in 4-6 months.

1.3. General

This sub-study is designed to evaluate Remicade for the treatment of moderately or severely ill hospitalized patients infected with COVID-19. This sub-study will have the same assessments and procedures as the main protocol.

The effectiveness of Remicade as add-on therapy to SoC will be evaluated based on the primary endpoint of time to recovery after 28 days. The sample size requirements are based on the ability to detect a moderate improvement in time to recovery (3-4 fewer days).

1.4. Study Population

Hospitalized adults (≥18 years old) with COVID-19, including patients both in and out of the ICU. Patients seeking care for COVID-19 in an Emergency Department (ED) and waiting to be admitted to the hospital are included.

1.5. Inclusion Criteria

Inclusion criteria are outlined in the main body of the ACTIV-1 IM protocol.

1.6. Agent Specific Exclusion Criteria at Randomization

1. Allergy to other monoclonal antibodies, or to any murine proteins.
2. History of HSTL or other lymphoma within 5 years before screening; history of or current diagnosis of multiple sclerosis (MS) or other significant demyelinating condition (e.g., optic neuritis).

1.7. Study Intervention

Therapeutic agents will be evaluated as an add-on therapy to SoC. The study intervention is as outlined in the main body of the ACTIV-1 IM protocol.

For the Remicade component, participants will receive active product as follows:

- Remicade will be administered as a single dose of 5 mg/kg (IV) on Day 1 in addition to SoC. The administration window will be within 24 hours of randomization. Infusion of Remicade and remdesivir should not be given concurrently in order not to confound attribution of possible infusion reactions or hypersensitivity events.

Duration of therapy:

- Remicade IV component – single dose on Day 1.

1.8. Schedule of Assessments

Assessments will be performed according to **Table 1-1** in 1.1.8 in the main body of the ACTIV-1 IM master protocol.

In addition, TB and HBV testing should be performed on Day 1 to rule out latent TB and/or HBV infection. Results do not need to be available for the participant to initiate treatment with study medication on Day 1. Once results are communicated, if the participant tests positive for TB or HBV, consultation with an infectious disease specialist and treatment for latent TB or hepatitis B should be considered.

2. JUSTIFICATION FOR SELECTION

Respiratory failure from acute respiratory distress syndrome (ARDS) is the leading cause of mortality associated with COVID-19 infection (Ruan et al. 2020).

Learnings from severe acute respiratory syndrome coronavirus 2 (SARS-CoV-2), initial COVID-19 data (Conti, Ronconi et al. 2020; Prompetchara et al. 2020) and preclinical mouse model data indicate that many cytokines including tumor necrosis factor α (TNFα), interleukin (IL)-1, and IL-6, may be key drivers of the acute lung injury (ALI) and ARDS observed in COVID-19. Severe COVID-19 patients from an initial study in China had elevated levels of TNFα. (Huang, Wang et al. 2020) SARS-CoV, a virus closely related to SARS-CoV-2, has been directly implicated in stimulating TNFα generation. The virus' spike protein has been shown to interact with angiotensin-converting enzyme 2 (ACE2) with consequent activation of TNFα converting enzyme (TACE), in a process that facilitates both viral entry into host cells and TNFα production (Haga et al. 2008).

Excess TNFα has been associated with multiple inflammatory diseases and may be an initiating or contributing factor to disease pathology and progression (Braun and Sieper 2003). TNFα has been reported to be an amplifier of inflammation, and suppressing it has the potential to suppress multiple downstream cytokines such as IL-1 and IL-6, as well as decrease capillary leak, and leukocyte trafficking as has been shown in rheumatoid arthritis (RA) patients (Feldmann et al. 2020).

Remicade binds to both soluble and transmembrane forms of TNFα and inhibits its functional activity. TNF antagonist therapy does not appear to be a risk factor for severe COVID-19 (Brenner et al. 2020).

No efficacy data are available on the use of Remicade to treat COVID-19 induced ARDS. Remicade has been approved across the globe for the treatment of Crohn's disease (adult and pediatric), RA, UC (adult and pediatric), psoriasis, PsA, and AS, although not all indications have been approved in all countries. Remicade has also been approved in Japan for use in some orphan indications including variants of psoriasis (psoriatic erythroderma and pustular psoriasis), Behçet's disease (entero-, neuro-, vasculo-, and uveitis), and acute Kawasaki disease.

Safety data from clinical studies are available from 6,122 Remicade-treated subjects including 2,363 with RA, 1,427 with Crohn's disease, 275 with AS, 191 with PsA, 1,373 with plaque psoriasis, and 493 with UC. Infusion-related reactions (e.g., dyspnea, flushing, headache and rash) were among the most common causes for discontinuation, except in UC, pediatric Crohn's disease, and PsA. Remicade has been mainly studied in chronic use, and approximately 3 million patients have been exposed to Remicade since its launch in August 1998.

3. INTRODUCTION

3.1. Risk/Benefit Assessment

3.1.1. *Remicade*

3.1.1.1. *Potential Risks of Remicade*

Risks that have been observed in patients with chronic use of Remicade but may also have relevance for single dose administration are the occurrence of serious infusion reactions, hypersensitivity, serious infections, liver enzymes increase, leukopenia or pancytopenia, and worsening of heart failure in those with NYHA Functional Class III/IV

heart failure. Although there is a theoretical risk of cancer with the use of Remicade, this is felt to be of less concern with single dose administration. Of these, the most significant risks relate to the complications associated with immunosuppression, essentially mycobacterial, fungal, and viral infections.

The following risk mitigations are in place:

The inclusion/exclusion criteria prevent patients with pre-existing comorbidities (including non-COVID-19-related active infections, steroid dependency, being on renal replacement therapy, severe hepatic inflammation, history of HSTL or other lymphoma within 5 years before screening, current NYHA Functional Class III/IV heart failure) from participating.

Remicade administered at a dose of 10 mg/kg at 0, 2 and 6 weeks post randomization has been associated with increased combined risk of death from any cause or hospitalization for heart failure (hazard ratio 2.84, 95% confidence interval 1.01 to 7.97; nominal p=0.043) in the ATTACH Trial, a randomized study evaluating the use of Remicade in participants with NYHA Functional Class III/IV heart failure. Remicade at a dose of 5 mg/kg did not increase risk of mortality or worsening of heart failure compared to placebo (Chung et al. 2003).

Remicade may have a risk of reactivation of latent TB and chronic hepatitis B. Therefore, participants will be screened for both TB and hepatitis B. Results of these tests do not need to be available for the participant to initiate study medication. If the participant tests positive for TB or hepatitis B, consultation with an infectious disease specialist and treatment for latent TB or hepatitis B should be considered.

To further protect the participants from the immunomodulatory effects of Remicade that may predispose them to infections, the investigators are referred to the local guidelines for the concomitant use of antibacterial and/or antiviral agents that are intended for management of community acquired pneumonia or secondary bacterial infections, and to inhibit SARS CoV-2 viral activity.

3.1.1.2. *Potential Benefits of Remicade*

The potential benefit of Remicade is to fulfill an urgent medical need for patients by shortening the period of hospitalization.

3.1.1.3. *Assessment of Potential Risks and Benefits of Remicade*

The potential benefits of treatment with Remicade are considered to outweigh any associated risks of treatment. This is based upon the following:

The proposed target patient population consists of individuals who have hypoxemia and who are at risk of ARDS. These are the patients with the most urgent medical need who are at the greatest risk of dying. The current mortality due to COVID-19-associated pulmonary complications for hospitalized patients with hypoxemia and pulmonary involvement ranges from approximately 20-50% depending upon the age of the individual and pre-existing comorbidities.

Access of patients to investigational therapeutics to mitigate the disease is limited in this pandemic setting, and this study will offer the potential to access a therapeutic in the context of a clinical study.

Remicade has been approved for more than 20 years and has accrued safety data from estimated marketing exposure of over 3 million patients encompassing over 7 million patient years of exposure. Remicade is contraindicated for CHF at a dose higher than 5 mg/kg.

By excluding those with elevated liver transaminases, class 3 or 4 congestive heart failure or with concomitant infections, and with appropriate monitoring during the study as well as inclusion of an Independent Data Monitoring Committee (IDMC), risk to participants can be minimized. While there may not be benefits for an individual participant, there may be benefits to society if a safe, efficacious therapeutic agent can be identified during this global COVID-19 pandemic. The potential risks therefore are thought to be acceptable given the potential benefits.

4. OBJECTIVES AND ENDPOINTS

The overall objective of the study is to evaluate the clinical efficacy and safety of different investigational therapeutics relative to the control arm among hospitalized adults who have COVID-19. All the endpoints for the overall trial are defined in the body of the main ACTIV-1 IM protocol.

5. SUB-STUDY DESIGN

5.1. Justification for Dose

5.1.1. *Justification for Dose of Remicade*

Based on the findings reported, blood TNFα levels are significantly elevated in severe and critical COVID-19 patients (Chen et al. 2020; Wu et al. 2020). In one study, blood TNFα levels were found to correlate with disease severity with a substantial proportion of severe and critical COVID 19 patients having TNFα levels exceeding 11 pg/mL (Komatsu et al. 2001). Of note, median serum TNFα levels in patients with active inflammatory bowel disease (IBD; UC and CD) ranged between 9.5 to 14.0 pg/mL (Komatsu et al. 2001). Recent emerging data has suggested that levels of TNFα may reach roughly 100 pg/mL in critically ill COVID-19 patients (Huang et al. 2020). At these higher levels of TNFα, the Remicade 5 mg/kg dose provides substantial molar excess. Remicade exposure after a single dose of 5 mg/kg IV is predicted to yield roughly 100,000-fold molar excess at T_{max} and 20,000 fold at 14 days post-dose to plasma TNFα, assuming a ~100 pg/mL concentration of TNFα.Therefore, the Remicade dose proposed to be evaluated in severe or critical COVID-19 patients is 5 mg/kg administered as a single IV infusion.

In the approved labeling, with the exception of patients with RA who start with a dose of 3 mg/kg Remicade in conjunction with methotrexate and have the option of increasing the Remicade dose up to 10 mg/kg, the standard recommended starting dose of Remicade is 5 mg/kg for all other indications. The safety and efficacy of the 5 mg/kg dose regimen of Remicade is well established in many immune-mediated disease populations such as patients with RA, Crohn’s disease, UC, and psoriasis.

In a short-term study of patients with CD where single doses of 5 mg/kg, 10 mg/kg, or 20 mg/kg Remicade were evaluated, no apparent dose-response was observed for this range of doses with respect to either magnitude or duration of clinical response or CRP reductions. Accordingly, it is not anticipated that doses greater than 5 mg/kg would be needed for acute treatment of COVID-19.

With respect to treatment duration, because the symptomatic phase of the disease lasts about 2 weeks (Zhou et al. 2020), it is reasonable to use a single dose of 5 mg/kg Remicade which is expected to provide adequate exposure to suppress elevated TNFα levels for the duration of the symptomatic phase of COVID-19 infection.

In summary, Remicade 5 mg/kg administered as a single IV infusion is expected to be effective with acceptable risks in rapidly suppressing the elevated TNFα levels in participants with confirmed severe or critical COVID-19.

6. STUDY POPULATION

The study population will adhere to the master protocol population, except for the noted additional exclusions and considerations below.

6.1. Inclusion Criteria

The same as the master protocol.

6.2. Sub-Study Specific Exclusion Criteria

1. Allergy to other monoclonal antibodies, or to any murine proteins.
2. History of HSTL or other lymphoma within 5 years before screening; history of or current diagnosis of MS or other significant demyelinating condition (e.g., optic neuritis).

6.3. Exclusion of Specific Populations

Since Remicade does not cross-react with TNFα in species other than humans and chimpanzees, animal reproduction studies have not been conducted with Remicade. No evidence of maternal toxicity, embryotoxicity, or teratogenicity was observed in a developmental toxicity study conducted in mice using an analogous antibody that selectively inhibits the functional activity of mouse TNFα. Doses of 10 mg/kg to 15 mg/kg in PD animal models with the anti-TNF analogous antibody produced maximal pharmacologic effectiveness. Doses up to 40 mg/kg were shown to produce no adverse effects in animal reproduction studies. It is not known whether Remicade can cause fetal harm when administered to a pregnant woman or can affect reproductive capacity. Remicade should be given to a pregnant woman only if considered that the benefits outweigh the risks.

As with other IgG antibodies, Remicade crosses the placenta. Remicade has been detected in the serum of infants up to 6 months following birth.

After in utero exposure to Remicade, infants may be at increased risk of infection, including disseminated infection that can become fatal. It is recommended that therapeutic infectious agents are not given concurrently with Remicade.

It is not known whether Remicade is excreted in human milk or absorbed systemically after ingestion. Because many drugs and immunoglobulins are excreted in human milk, and because of the potential for ADRs in nursing infants from REMICADE, a decision should be made whether to discontinue nursing or to discontinue the drug, taking into account the importance of the drug to the mother.

7. STUDY PRODUCT

7.1. Study Product(s) and Administration

Participants will be randomized according to the procedure described in the master protocol.

If there are supply limitations on any product, the arms containing that product will be temporarily closed to enrollment and the corresponding placebo is not needed.

7.1.1. *Study Product Description*

Remicade is a chimeric human-murine IgG1γ monoclonal antibody that specifically binds to human TNFα. In addition to the active ingredient, the lyophilized formulation of Remicade contains the following inactive ingredients: water for injection, sucrose, polysorbate 80, monobasic sodium phosphate monohydrate, and dibasic sodium phosphate dihydrate.

7.1.2. *Dosing and Administration*

For the Remicade component, participants will receive either active product or placebo as follows:

- Remicade will be administered as a single dose of 5 mg/kg (IV) on Day 1 in addition to SoC.
- A placebo of IV saline will be given at an equal volume at the same schedule in addition to SoC.

The administration window will be within 24 hours of randomization. Any dose that is delayed may be given later that calendar day. Any dose that is missed (not given that calendar day) is not made up. The treatment course continues as described above even if the participant becomes PCR negative.

Duration of therapy:

- Remicade IV component or saline placebo – single dose on Day 1.

7.1.3. *Dose Escalation*

Not Applicable

7.1.4. *Dose Modifications*

For mild to moderate infusion reactions, the patient may improve following slowing or suspension of the infusion, and upon resolution of the reaction, re-initiation at a lower infusion rate and/or therapeutic administration of antihistamines, acetaminophen, and/or corticosteroids. For participants who do not tolerate the infusion following these interventions, Remicade should be discontinued.

Participants who have severe infusion-related hypersensitivity reactions should be discontinued from further Remicade treatment. The management of severe infusion reactions should be dictated by the signs and symptoms of the reaction. Appropriate personnel and medication should be available to treat anaphylaxis if it occurs.

7.1.5. *Overdosage*

There is no known specific antidote for Remicade overdose. In the event of an overdose, the participant should be monitored for any signs or symptoms of adverse effects and institute appropriate symptomatic treatment immediately.

7.1.6. *Preparation/Handling/Storage/Accountability*

7.1.6.1. *Acquisition and Accountability*

Investigational products (IP) will be shipped to the site either directly from participating companies, from the Sponsor, or from other regional or local drug repositories. All other supplies should be provided by the site. Multiple lots of each IP may be supplied.

Study products received at the sites will be open label and not kit specific, unless specified in the protocol-specific Manual of Procedures (MOP) or pharmacy manual. Drug preparation will be performed by the participating site's unblinded research pharmacist on the same day of administration to the participant. See the MOP Appendices for detailed information on the preparation, labeling, storage, and administration of investigational products.

Accountability:

The site PI is responsible for study product distribution and disposition and has ultimate responsibility for study product accountability. The site PI may delegate to the participating site's research pharmacist responsibility for study product accountability. The participating site's research pharmacist will be responsible for maintaining complete records and documentation of study product receipt, accountability, dispensation, storage conditions, and final disposition of the study product(s). Time of study drug administration to the participant will be recorded on the appropriate data collection form (CRF). All study product(s), whether administered or not, must be documented on the appropriate study product accountability record or dispensing log. The Sponsor's monitoring staff will verify the participating site's study product accountability records and dispensing logs per the site monitoring plan. Refer to the protocol-specific MOP for details on storing study medications.

Destruction:

After the study treatment period has ended or as appropriate over the course of the study after study product accountability has been performed, used active and placebo product can be destroyed on-site following applicable site procedures with a second staff member observing and verifying the destruction.

Unused product at the end of the study should be saved until instructed by the Sponsor.

7.1.6.2. *Formulation, Appearance, Packaging, and Labeling*

Remicade component: The lyophilized formulation of Remicade is a preservative-free, white, lyophilized solid containing 100 mg of Remicade to be reconstituted with 10 mL of sterile water for injection and diluted into IV infusion fluids prior to IV infusion. Following reconstitution, each vial contains a 10 mg/mL Remicade concentrated solution.

It is supplied as a sterile product in a single-use, Type 1 clear glass vial. In addition to the active ingredient, the lyophilized formulation of Remicade contains the following inactive ingredients: water for injection, sucrose, polysorbate 80, monobasic sodium phosphate monohydrate, and dibasic sodium phosphate dihydrate. For more information, refer to the MOP.

Injectable placebo component:

Injectable placebo will be IV normal saline (0.9% NaCl) made up unblinded by the resident pharmacist and blinded to the study team. Note that the type of infusion bag used for placebo should be identical to that used for the preparation of the Remicade infusion in order to ensure the blind in the study.

7.1.6.3. *Product Storage and Stability*

Study intervention must be stored at controlled temperatures as indicated on the product label and should not be used beyond the expiration date.

Refer to the MOP for additional guidance on study intervention storage.

7.1.6.4. *Preparation and Administration*

Refer to the protocol-specific MOP for details about preparation.

Remicade 5 mg/kg will be administered to the participant via an IV infusion at a rate of not more than 100 mL/h over a time period of not less than 2 hours using a total volume of 250 mL. Placebo participants will receive the IV infusion of 250 mL normal saline at the same rate. Aseptic procedures must be used during the study agent infusion.

Remicade must not be infused with other agents in the same infusion line. Generally, lines should be flushed before and after use if to be used for administration of other medications. In addition, the infusion of Remicade should not be given concurrently with the infusion of Remdisivir in order not to confound attribution of possible infusion reactions or hypersensitivity events.

Refer to the MOP for additional guidance on study intervention preparation and handling.

7.2. Measures to Minimize Bias: Randomization and Blinding

Randomization will be performed as specified in the Master Protocol

7.3. Study Intervention Compliance

Each dose of study product will be administered by a member of the clinical research team who is qualified and licensed to administer the study product. Administration date and infusion start/stop times will be entered into the case report form (CRF).

7.4. Concomitant Therapy

7.4.1. *Permitted Concomitant Therapy and Procedures*

All concomitant therapies permitted in the master protocol will be allowed for this sub-study.

Of further note in regard to co-administration of corticosteroids, Remicade in combination with corticosteroids, including dexamethasone, has been used in clinical trials and 20+ years of post-marketing experience in several immuno-mediated inflammatory diseases with an overall positive benefit-risk profile. This profile was established in the context of long-term use of Remicade in patients without active infection at the time of initiation of treatment.

7.4.2. *Prohibited Concomitant Therapy*

All concomitant therapies prohibited in the master protocol will also be prohibited for this sub-study.

Medications to be used with caution as their exposure could be modified by the study medication

The formation of CYP450 enzymes may be suppressed by increased levels of cytokines (e.g., TNFα, IL-1, IL-6, IL-10, IFN) during chronic inflammation. Therefore, it is expected that for a molecule that antagonizes cytokine activity, such as Remicade, the formation of CYP450 enzymes could be normalized. Upon initiation of Remicade in patients being treated with CYP450 substrates with a narrow therapeutic index, monitoring of the effect (e.g., warfarin) or drug concentration (e.g., cyclosporine or theophylline) is recommended and the individual dose of the drug product may be adjusted as needed.

7.4.3. *Rescue Medicine*

Not Applicable.

7.4.4. *Non-Research Standard of Care*

Not Applicable.

8. STUDY INTERVENTION DISCONTINUATION AND SUBJECT DISCONTINUATION/WITHDRAWAL

8.1. Halting Criteria and Discontinuation of Study Intervention

8.1.1. *Individual Study Product Halting*

Individual study product halting will be managed as specified in the Master Protocol.

Remicade Halting

For mild to moderate infusion reactions, the patient may improve following slowing or suspension of the infusion, and upon resolution of the reaction, re-initiation at a lower infusion rate and/or therapeutic administration of antihistamines, acetaminophen, and/or corticosteroids. For patients that do not tolerate the infusion following these interventions, Remicade should be discontinued.

Patients who have severe infusion-related hypersensitivity reactions should be discontinued from further Remicade treatment. The management of severe infusion reactions should be dictated by the signs and symptoms of the reaction. Appropriate personnel and medication should be available to treat anaphylaxis if it occurs.

8.1.2. *Study Halting*

Study halting will be managed as specified in the Master Protocol.

8.2. Withdrawal from the Study

Sub-study will have the same requirements as the main study.

8.3. Lost to Follow-Up

Sub-study will have the same requirements as the main study.

8.4. Readmission

Sub-study will have the same requirements as the main study.

9. STUDY ASSESSMENTS AND PROCEDURES

Sub-study will have the same assessments and procedures as the main protocol.

10. STATISTICAL CONSIDERATIONS

Statistical analysis will be performed as described within the main body of the ACTIV-1 IM master protocol.

APPENDIX 2 – SUB-STUDY 2 – ABATACEPT (ORENCIA)

1. SUB-STUDY SUMMARY

1.1. Sub-study overview

The sub-study population corresponds to moderately and severely ill patients infected with the COVID-19 virus. Recruitment will target patients already hospitalized for treatment of COVID-19 infection as well as patients being treated for COVID-19 infection in Emergency Departments while waiting to be admitted to the hospital. Patients both in and out of the ICU are included in the study population.

Enrollment will begin as soon as all elements are met for the sub-study to activate.

The study period is 60 days, with assessments on each day of the hospital stay. Patients will be followed after hospital discharge with periodic follow-up assessments through Day 29 and at Day 60.

1.2. Enrollment Period

Enrollment is expected to begin in September 2020. It is anticipated the enrollment may be completed in 4-6 months.

1.3. General

This sub-study is designed to evaluate abatacept for the treatment of moderately or severely ill hospitalized patients infected with COVID-19. Sub-study will have the same assessments and procedures as the main protocol.

The effectiveness of abatacept as add-on therapy to SoC will be evaluated based on the primary endpoint of time to recovery after 28 days. The sample size requirements are based on the ability to detect a moderate improvement in time to recovery (3-4 fewer days).

1.4. Study Population

Hospitalized adults (≥18 years old) with COVID-19, including patients both in and out of the ICU. Patients seeking care for COVID-19 in an Emergency Department (ED) and waiting to be admitted to the hospital are included.

1.5. Inclusion Criteria

Inclusion criteria are outlined in the main body of the ACTIV-1 IM protocol.

1.6. Agent Specific Exclusion Criteria

There are no agent specific exclusion criteria for this sub-study.

1.7. Study Intervention

Therapeutic agents will be evaluated as an add-on therapy to SoC. The study intervention is as outlined in the main body of the ACTIV-1 IM protocol.

For the abatacept component, the dose of abatacept will be 10 mg/kg with a maximum dose of 1000 mg. Study medication will be administered in a fixed volume of 100 mL at a constant infusion rate over approximately 30 minutes. The IV line must be flushed with 25 mL of D5W or NS solution at the end of the infusion. The administration window will be within 24 hours of randomization.

Infusion of abatacept and remdesivir should not be given concurrently in order not to confound attribution of possible infusion reactions or hypersensitivity events.

Duration of therapy:

- Abatacept IV component – single infusion on Day 1.

1.8. Schedule of Assessments

Assessments will be performed according to **Table 1-1** in 1.1.8 in the main body of the ACTIV-1 IM master protocol.

2. JUSTIFICATION FOR SELECTION

It is hypothesized that abatacept therapy can modulate the ongoing/emerging dysregulated immune response considered to be driving the progression of disease severity in COVID-19. Abatacept is not a therapy for neutralizing cytokines but for preventing further cellular activation (e.g., CD4+ T cells, B cells, macrophages) and production of cytokines and interrupting the feedforward pathological process.

Appendix 2 Figure 1: Cellular interactions affected by abatacept

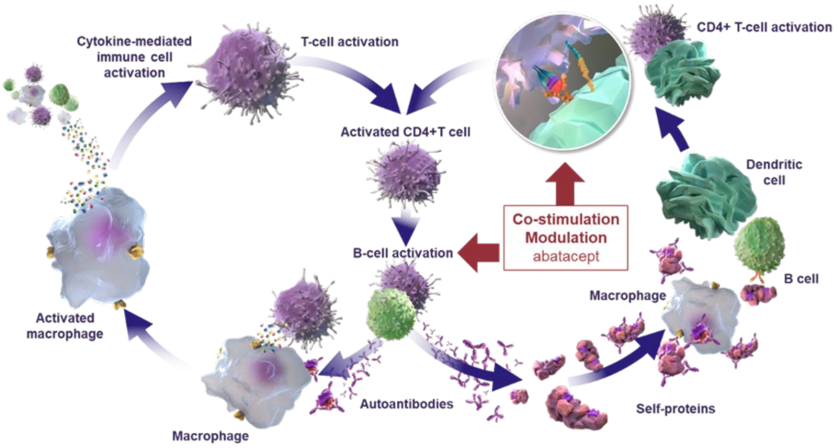

Abatacept was first studied in the setting of a pathological response to a viral infection in a murine model of influenza (Teijaro et al. 2009). In this study, abatacept could prevent development of severe lung injury while not inhibiting an effective memory antiviral response in the setting of secondary infection. This study, along with others, have continued to evolve the understanding of the mechanism of action (MOA) of abatacept which has proven to be much more complex than initially envisioned. Although CTLA-4 is central to the regulation of T cell activation, CTLA-4Ig clearly has effect on other cell types. Since CTLA-4 regulates interactions with T cells and professional antigen presenting cells (APC), it is not surprising that effects on APC populations (i.e., dendritic cells, macrophages, B cells) can also be seen, in vitro and clinically (Chambers and Allison 1999). Indeed, in vitro work has demonstrated reverse signaling via CD80 or CD86 in APCs although the full impact of this is not fully understood (Davis et al. 2008).

Treatment with abatacept has also been shown to have broad effects on cytokines in RA patients. Abatacept shows an impact within 24 hours in human in vitro mixed-lymphocyte reactions (MLR) on IL-2, TNF-α, and IFN-γ (Davis, Nadler et al. 2008). In vivo, abatacept reduces the levels of multiple cytokines in RA patients including TNF-α, sIL-2R and IL-6 (Weisman et al. 2006). In addition, abatacept suppresses multiple biomarkers (cytokines and chemokines) in the synovium from RA patients (Buch et al. 2009). What these studies don't show is which cell type(s) was most impacted.

The best evidence for abatacept's direct effects on APCs comes from reports on both in vitro and in vivo effects on macrophages and B cells. Abatacept inhibits pro-inflammatory cytokine (TNF-a, IL-12, INF-g) secretion by in vitro activated human macrophages (Wenink et al. 2012). Abatacept can also downregulate production of IL-6, TNFα, IL1-β and TGFβ from activated synovial macrophages from RA patients (Cutolo et al. 2009). B-cell populations are

notably decreased in RA synovium of abatacept-treated patients (Buch et al. 2009). Direct effects of abatacept on plasmablasts has also been proposed (Carvajal Alegria et al. 2016). Together, all of this data suggests that abatacept's impact on the immune system is not limited to T cells. Activations of macrophages and B cells are also likely impacted by abatacept therapy.

Background

Abatacept is a recombinant fusion protein (MW 92 kDa) consisting of the extracellular domain of human CTLA4 and a fragment (hinge - CH2 - CH3 domains) of the Fc domain of human immunoglobulin (Ig) GI that has been modified to prevent complement fixation and antibody-dependent cellular cytotoxicity. Abatacept is a selective costimulation modulator that inhibits T-cell activation by binding to CD80 and CD86 on antigen presenting cells, thereby blocking the interaction with CD28 on T-cells that provides a costimulatory signal necessary for full activation of T-cells. By inhibiting CD28 mediated T-cell activation upstream of inflammatory cytokines, such as TNF, abatacept utilizes a unique mechanism of action that offers significant therapeutic benefit to patients with a variety autoimmune-mediated diseases (Linsley and Nadler 2009). Abatacept, 250 mg for intravenous (IV) infusion, is indicated to treat participants with rheumatoid arthritis (RA) age 18 and older and participants with polyarticular juvenile idiopathic arthritis (pJIA) age 6 to 17.

IV-administered abatacept was first approved in the US for the treatment of moderate-to-severe RA in adults in December 2005. Since then, IV abatacept has received marketing approval for the treatment of adult RA in many other countries, including the EU, Canada, Australia, and Japan. IV abatacept was also approved in the US for the treatment of moderately-to-severely active juvenile idiopathic arthritis (JIA) in pediatric patients 6 years of age or older in April 2008. In addition, IV abatacept has received marketing approval for the treatment of JIA in several other countries, including the EU, Canada, and Australia. In 2017, abatacept was approved for adult use in psoriatic arthritis (PsA) in the US and has also received marketing approval for the treatment of PsA in several other countries, including the EU, Canada, and Australia. A subcutaneous (SC) formulation of abatacept in a prefilled syringe and autoinjector has been approved for adult RA and PsA patients in the US, EU, and several other countries; it is also approved in the US and EU for use in JIA.

A detailed description of the chemistry, pharmacology, efficacy, and safety of abatacept is provided in the Investigator's Brochure (IB) (Company, B.M.S. 2020a; Company, B.M.S. 2020b).

Clinical Evidence for Abatacept Suppression of Cytokine Release Syndrome

There is some clinical data to inform the impact on cytokine release syndrome (CRS). Patients with active systemic JIA, some with current macrophage activation syndrome (MAS), who were heavily treated with multiple agents, including high dose corticosteroids and anakinra, responded to the addition of abatacept. The time course for the benefit of abatacept is not well described but the ability to decrease doses or suspend some concomitant therapy is reported.

A subject with active immune checkpoint inhibitor-associated myocarditis did demonstrate clinical and biochemical improvement within a day of initiating abatacept therapy (Salem et al. 2019). This clinical course has been seen in other similar patients treated with abatacept (personal communication of unpublished data).

The strongest evidence for rapid onset of abatacept and its effect on CRS is from prevention, not treatment. Use of peripheral blood stem cell (PBSC) graft for post-transplantation cyclophosphamide (PTCy)-based haploidentical hematopoietic cell transplantation (HCT) is associated with early CRS in over 92% of patients (14% severe CRS) (Jaiswal and Chakrabarti 2020). Treatment with abatacept 1 day before the infusion of the PBSC graft (followed dosing on day +5, +20, +35 and every four weeks after) decreased the rate of CRS to 6% (none severe). The continued use of abatacept is because of ongoing donor lymphocyte infusions, which are therapeutic in this context and also believed to be the source of the CRS process.

In a related clinical setting, abatacept has been studied for acute graft-versus-host disease (aGVHD) prevention during unrelated-donor hematopoietic cell transplantation (HCT), in both HLA matched (8/8) and unmatched (7/8) patients

(Koura et al. 2013; Watkins et al. 2017). Patients who receive unrelated-donor HCT are at elevated risk of severe aGVHD and death. Abatacept treatment at days -1, +5, +14, and +28 almost completely suppresses severe (Grade III-IV) aGVHD without negative impact on relapse and without significant impact on patient safety. CMV reactivation, but not EBV, was seen more frequently (47% vs 33%, p=0.16) but uncontrolled infections were not a major problem.

The relevance of these reports is that they illustrate the rapid effects of abatacept on feedforward mechanisms that produce CRS. They also suggest that the onset of abatacept's therapeutic effects can be sufficiently rapid as to be clinically relevant in the setting of COVID-19.

3. INTRODUCTION

3.1. Risk/Benefit Assessment

3.1.1. *Abatacept*

3.1.1.1. *Potential Risks of Abatacept*

The safety profile of abatacept is well established based on the safety data collected from a number of clinical studies and 14-year post-marketing experience during the treatment of autoimmune/inflammatory diseases; abatacept has been well-tolerated and shown a favorable benefit-risk profile in the approved disease population (i.e., RA, PsA, pJIA).

Infections and infusion related reactions (for IV formulation) are identified risks for abatacept, the majority of these events are non-serious and do not impact benefit/risk profile of the product. For infections, during the double-blind, placebo-controlled period of RA studies (9 IV studies and 2 SC studies in the integrated safety database), the incidence rate of overall infections was comparable between abatacept group (total of 2,653 subjects with mean exposure duration of 10.8 months) and placebo group (total of 1,485 subjects with mean exposure duration of 10.3 months): 93.21 versus 93.02 per 100 patient-years; a small increase for IR of overall serious infections in the abatacept group compared to placebo group (3.0 vs 2.25 per 100 patient-years). Majority of the infections were caused by common pathogens; the most commonly reported PTs of infections were upper respiratory tract infection (IR 14.3 per 100 patient-years) and nasopharyngitis (IR 14.3 per 100 patient-years); the most frequently reported serious infection was pneumonia (IR 0.68 per 100 patient-years) in abatacept group, which was comparable to that in placebo group (IR 0.72 per 100 patient-years). The IR of opportunistic infections appeared to be numerically lower in abatacept group compared to placebo (0.17 vs 0.56 per 100 patient years). For the infusion related reactions occurred in IV abatacept studies, most of the peri-infusional events that occurred within 24 hours following the start of infusion were non-serious dizziness and nausea; serious events of hypersensitivity/anaphylactic reactions were rare. Safety experience from clinical studies in PsA and pJIA patients was similar to that in RA studies. Ten-year post-marketing epidemiology studies in RA population did not reveal any new safety finding relevant to these two identified risks.

Malignancies and autoimmune disorders have been reported with abatacept use; however, the clinical and post-marketing data do not show an increased risk of these events associated with abatacept use, therefore, the potential role of abatacept in the development of these diseases cannot be concluded based on the available evidence.

The potential deleterious impact of abatacept on viral clearance is certainly a concern that we recognize. The previously discussed murine model of influenza infection-induced immunopathology clearly demonstrates the risk of blunting anti-viral responses in abatacept-treated mice prior to viral exposure (Teijaro et al. 2009). These concerns are not new. Throughout the abatacept clinical development program, concerns over the impact of therapy on infectious agents has been paramount.

During pre-clinical abatacept development, host resistance models of infection were explored (Linsley and Nadler 2009). Of note, negative impacts on immune responses were only noted for herpes simplex infections. The observation that abatacept partially inhibits the generation of CD8 cytotoxic T cell (CTL) responses but does not inhibit CD8 T cell cytotoxic activity has been reported, which could also predict deleterious impact on viral responses (Van Gool et al. 1996; Whitmire and Ahmed 2000).

In spite of these pre-clinical observations, clinical observations, both from controlled trials and post marketing surveillance, have been of less concern. A review of safety data of viral infections from an integrated safety database of RA clinical studies (includes 7044 participants receiving IV or SC abatacept for a total exposure of 21994.84 patient years during the cumulative period (controlled plus long-term open label)), reveals the following:

1. During the randomized placebo-controlled double-blind short-term period of the studies, the most frequently (>1.0%) reported PTs of viral infections and infections that could be caused by virus in the ABA group vs. placebo group, respectively, were upper respiratory tract infection (11.9% vs 12.1%), nasopharyngitis (11.8% vs 10%), bronchitis (6.5% vs 5.8%), influenza (5.8% vs 5.9%), pharyngitis (3.5% vs 3.3%), rhinitis (2.4% vs 1.3%), conjunctivitis (2.1% vs 1.5%), gastroenteritis (2.0% vs 3.0%), oral herpes (1.8% vs 1.3%), herpes zoster (1.5% vs 1.4%), pneumonia (1.8% vs 1.1%). Majority of these events were mild or moderate in severity and abatacept use did not cause increased severity of infections compared to placebo.
2. During the DB ST period of the studies, the most frequently reported PTs (≥ 2 cases) of serious viral infections and infections that could be caused by virus in ABA group and placebo group were pneumonia (0.6% vs 0.6%), bronchitis (0.2% vs 0), gastroenteritis (0.1% vs 0.1%), herpes zoster (0.1% vs 0). Among these events, death outcomes were reported for pneumonia and the IRs for fatal pneumonia were 0.08 vs 0.16 per 100 patient-years for abatacept and placebo, respectively.
3. There were no increases for the incident rates of serious infections reported during cumulative period compared to those reported in short-term period.

Of note is the absence of an imbalance in viral pneumonias, specifically influenza. Also, the median event duration for influenza was similar between abatacept treated patients and placebo group. Antibody responses to the conventional seasonal influenza vaccine in abatacept treated RA patients were also studied (Alten et al. 2016). The study demonstrated that most participants on abatacept were able to mount an appropriate immune response. While this study did not demonstrate impact on actual influenza rates, it does support the extensive clinical observation that abatacept therapy may not influence anti-influenza responses in a clinically meaningful way.

Review of the post-marketing safety experience (estimated exposure at 763,109 p-y as of 30-Sep-2019 in > 13 years) identified 21 cases of coronavirus infection by 13-Apr-2020, events of confirmed COVID-19 or suspected COVID-19 were reported in 13 of these cases. The majority of these cases contained minimal information and robust assessment of the impact of abatacept on COVID-19 is not possible. One literature report from Japan reported possible COVID-19 in a 70-year-old male received abatacept and tacrolimus to treat RA and interstitial lung disease (ILD). The patient developed respiratory compromise and ultimately required ventilatory support. Testing of sputum but no other sources was positive for SAR-CoV-2. A recent published letter describes 8 patients being treated with targeted therapies for chronic arthritis in Italy. Among them were 2 patients receiving therapy with abatacept, 1 with confirmed and one with suspected SARS-CoV-2 infection. Only 1 of the patients required hospitalization, and then only briefly for oxygen therapy and all recovered without incident (Monti et al. 2020). Belatacept is a related drug to abatacept with a similar mechanism of action. In a recent report (Marx et al. 2020), a 58 year old kidney transplant patient on belatacept and MMF became infected with SARS-CoV-2 and had a mild clinical course with rapid recovery. The authors hypothesized that ongoing immunotherapy with belatacept may protect from severe clinical course of COVID-19 by limiting cytokines/chemokines production.

The abatacept label has a warning for risk of serious infections, including fatal sepsis and pneumonia. The clear risk for abatacept use is in bacterial infections, mostly of the respiratory and urinary tract. This includes a higher rate of complications in patients with COPD. The abatacept label also warns about treatment initiation in patients with an active infection until the infection is controlled. Of note, extensive experience in the use of abatacept to treat RA in elderly patients with co-morbidities has been reported (Monti et al. 2017). This suggests that among biologic DMARD therapies, abatacept may have less risk of infections (Curtis et al. 2014; Kang et al. 2019; Chen et al. 2020).

Based on the review of safety data relevant to viral infections from clinical studies and post marketing experience, there is no evidence suggesting increased risk of severe respiratory illnesses associated with abatacept use. Current evidence suggests that once COVID-19 patients develop symptoms of respiratory compromise, their anti-viral responses, including adaptive immune responses, are well underway but the pathological process leading to CRS-like scenario has only just begun. It is proposed that abatacept may interrupt this process without greatly interfering with the viral clearance and that any study to be attempted should be aware of these goals and concerns.

3.1.1.2. *Potential Benefits of Abatacept*

It is hypothesized that abatacept therapy can modulate the ongoing/emerging dysregulated immune response considered to be driving the progression of disease severity in the setting of SARS-CoV-2 infection. Abatacept is not a therapy for neutralizing cytokines but for preventing further cellular activation (e.g. CD4+ T cells, B cells, macrophages) and production of cytokines and interrupting the feedforward pathological process. The sponsor's hypothesis is that once patients with COVID-19 develop symptoms of respiratory compromise, their anti-viral responses, including adaptive immune responses, are well underway but the pathological process leading to the CRS-like scenario has not yet fully developed. We hypothesize that abatacept may interrupt this process without negatively impacting viral clearance.

Abatacept has demonstrated the ability to modulate the maladaptive immune response in multiple clinical models of Cytokine Release Syndrome (CRS) and in clinical conditions characterized by CRS. The most relevant data for the use of abatacept in the setting of COVID-19 comes from a mouse model of influenza pneumonitis. In this model, treatment with CTLA4-Ig decoupled the protective and immunopathological memory T cell responses following secondary infection without effecting viral clearance and reduced the development of the immunopathology and enhanced recovery. The use of CTLA4-IG in mouse models of both Toxic Shock and Staph B induced cytokine storm resulted in significant reductions in morbidity (including lung damage and edema) and mortality, and lower levels of both Il-6 and Ifn-ϒ (Whitfield et al. 2017).

3.1.1.3. *Assessment of Potential Risks and Benefits of Abatacept*

Current evidence indicates that once COVID-19 patients develop symptoms of respiratory compromise, their anti-viral responses, including adaptive immune responses, are well underway but the pathological process leading to a CRS-like scenario has only just begun. The sponsor proposes that abatacept may interrupt this process without greatly interfering with the viral clearance.

Preclinical work indicates that abatacept can suppress or control cytokine production, including clinical scenarios consistent with CRS. Clinical work in multiple complex disease states also suggests abatacept therapy can suppress or help manage excessive cytokine production.

Abatacept has been studied in human clinical trials and marketed for over 15 years. Most patients treated have chronic, immune-mediated diseases with use of concomitant immunosuppressant therapies and co-morbid conditions. The most notable risk for abatacept use is an increase in the rate of bacterial infections, particularly of the respiratory and urinary tract. Based on the review of safety data relevant to viral infections from clinical studies and post-marketing experience, there is no evidence suggesting an increased risk of viral respiratory illnesses, severe or otherwise, associated with abatacept use.

The study of the therapeutic potential of abatacept in symptomatic patients with COVID-19 at high risk for progression to respiratory failure is warranted. Concerns over the negative impact on viral clearance or secondary bacterial infections are appropriate. These concerns are mitigated by the relatively short course of treatment in the setting of hospitalized monitoring and care, monitoring of super-infections during the study and an oversight study committee.

More detailed information about the known and expected benefits and risks and reasonably anticipated adverse events of abatacept may be found in the Investigator's Brochure (Company, B.M.S. 2020a; Company, B.M.S. 2020b).

4. OBJECTIVES AND ENDPOINTS

The overall objective of the study is to evaluate the clinical efficacy and safety of different investigational therapeutics relative to the control arm among hospitalized adults who have COVID-19. All the endpoints for the overall trial are defined in the body of the main ACTIV-1 IM protocol.

5. STUDY DESIGN

5.1. Justification for Dose

5.1.1. *Justification for Dose of Abatacept*

Abatacept is available in 2 formulations, lyophilized for intravenous infusion and pre-filled syringe for subcutaneous injection. Pharmacokinetic evaluation over 2 decades of clinical development has shown that the most relevant PK parameter is trough (Cmin) levels which are best at predicting maximal efficacy and are driven by weight-based dosing. Superior efficacy is observed with 10 mg/kg dosing in RA patients. Dosing frequency of Day 1, 15, 28 and every 28 days afterwards was established as an efficacious dosing regimen for reaching target Cmin levels. Evidence based on pharmacokinetic, pharmacodynamic and clinical efficacy data suggest that higher doses of abatacept would not be more effective in RA patients. Higher doses of abatacept at 30 mg/kg have been tested in BMS sponsored trials. In patients with PsA, higher doses did not produce better efficacy but also did not result in any increase in safety signal. In inflammatory bowel disease, no clinical efficacy was seen. In lupus nephritis, higher doses were thought needed due to urinary losses of abatacept due to the severe proteinuria unique to this disease. None of these studies suggested higher abatacept doses were associated with increased safety concerns. Based on the experiences in other systemic inflammatory diseases, which we believe are somewhat informative as to what we will see in COVID-19 related disease, we believe a one time IV dose of abatacept would be sufficient to provide continuous exposure above the target Cmin and have no reason to believe a higher dose or higher frequency would be needed.

6. STUDY POPULATION

The study population will adhere to the master protocol population, except for the noted additional exclusions and considerations below.

6.1. Inclusion Criteria

The same as the master protocol.

6.2. Sub-Study Specific Exclusion Criteria

The same as the master protocol.

6.3. Exclusion of Specific Populations

Children and adolescents will not be included in this trial. Abatacept has been used extensively in pediatric patients.

The data with abatacept use in pregnant women are insufficient to inform on drug-associated risk. In reproductive toxicology studies in rats and rabbits, no fetal malformations were observed with intravenous administration of abatacept during organogenesis at doses that produced exposures approximately 29 times the exposure at the maximum recommended human dose (MRHD) of 10 mg/kg/month on an AUC basis. However, in a pre- and postnatal development study in rats, ORENCIA altered immune function in female rats at 11 times the MRHD on an AUC basis. There are no adequate and well-controlled studies of abatacept use in pregnant women. The data with abatacept use in pregnant women are insufficient to inform on drug-associated risk.

Intravenous administration of abatacept during organogenesis to mice (10, 55, or 300 mg/kg/day), rats (10, 45, or 200 mg/kg/day), and rabbits (10, 45, or 200 mg/kg every 3 days) produced exposures in rats and rabbits that were approximately 29 times the MRHD on an AUC basis (at maternal doses of 200 mg/kg/day in rats and rabbits), and no embryotoxicity or fetal malformations were observed in any species

In a study of pre- and postnatal development in rats (10, 45, or 200 mg/kg every 3 days from gestation day 6 through lactation day 21), alterations in immune function in female offspring, consisting of a 9-fold increase in T-cell-dependent antibody response relative to controls on postnatal day (PND) 56 and thyroiditis in a single female pup on PND 112, occurred at approximately 11 times the MRHD on an AUC basis (at a maternal dose of 200 mg/kg). No adverse effects were observed at approximately 3 times the MRHD (a maternal dose of 45 mg/kg). It is not known if

immunologic perturbations in rats relevant indicators of a risk for development of autoimmune diseases in humans are exposed in utero to abatacept. Exposure to abatacept in the juvenile rat, which may be more representative of the fetal immune system state in the human, resulted in immune system abnormalities including inflammation of the thyroid and pancreas [see Nonclinical Toxicology.

There is no information regarding the presence of abatacept in human milk, the effects on the breastfed infant, or the effects on milk production. However, abatacept was present in the milk of lactating rats dosed with abatacept.

7. STUDY PRODUCT

7.1. Study Product(s) and Administration

Participants will be randomized according to the procedure described in the master protocol.

If there are supply limitations on any product, the arms containing that product will be temporarily closed to enrollment and the corresponding placebo is not needed.

7.1.1. *Study Product Description*

Abatacept for Injection is a lyophilized powder for intravenous infusion. ORENCIA for Injection is supplied as a sterile, white, preservative-free, lyophilized powder for reconstitution and dilution prior to intravenous administration. Following reconstitution of the lyophilized powder with 10 mL of Sterile Water for Injection, USP, the solution of abatacept is clear, colorless to pale yellow, with a pH range of 7.2 to 7.8. Each single-use vial of abatacept for Injection provides 250 mg abatacept, maltose (500 mg), monobasic sodium phosphate (17.2 mg), and sodium chloride (14.6 mg) for administration.

7.1.2. *Dosing and Administration*

The dose of abatacept will be 10 mg/kg with a maximum dose of 1000 mg. Study medication will be administered in a fixed volume of 100 mL at a constant infusion rate over approximately 30 minutes. The IV line must be flushed with 25 mL of D5W or NS solution at the end of the infusion. The administration window will be within 24 hours of randomization.

Infusion of abatacept and remdesivir should not be given concurrently in order not to confound attribution of possible infusion reactions or hypersensitivity events.

Duration of therapy:

- Abatacept IV component – single infusion on day 1

7.1.3. *Dose Escalation*

Not Applicable

7.1.4. *Dose Modifications*

In the absence of adverse events, participants will complete their scheduled infusion as prescribed by protocol. If there is evidence of toxicity (e.g., infusion reaction) that, in the judgment of the Investigator, could place the participant at increased risk, study drug administration should be discontinued.

Management of Possible Acute Hypersensitivity Reactions to Abatacept

Hypersensitivity or acute allergic reactions may occur as a result of the protein nature of abatacept. Should any of these reactions occur during the course of the study, they need to be reported as specified in 8.3.6 (Adverse Events). In this study, participants' vital signs will be monitored before and following study drug administration. Appropriate emergency equipment and qualified personnel should be available where the participants are treated in the event of a serious anaphylactic reaction.

The following information is provided to assist in the recognition of hypersensitivity reactions and in the management of those reactions should they occur during or after the administration of abatacept. Care should be taken to treat any acute toxicities expeditiously, should they occur. When IV dosing of abatacept is conducted, equipment such as a portable tank or wall-source of oxygen, endotracheal intubation set, oral airway, mask, ambu-bag, syringes, injectable epinephrine, injectable antihistamine, and injectable corticosteroids should be kept in the vicinity where the participant is treated.

Signs and management of potential acute hypersensitivity reactions include:

a) Symptomatic Hypotension should be managed by discontinuing the infusion of study medication, placing the participant in the Trendelenburg position and administering intravenous fluid. Additional medical intervention may also include the use of epinephrine, corticosteroids, antihistamines and pressor agents.
b) Dyspnea should be managed by discontinuing the infusion of study medication and observing the participant for worsening of the event and for the appearance of additional signs and symptoms of anaphylaxis. Antihistamines, epinephrine and corticosteroids may be administered as indicated.
c) Acute pain in the chest, back or extremities may also be a sign of anaphylaxis and may be treated as described above for dyspnea
d) Chills, fever, urticaria or generalized erythema may all be signs of an allergic reaction to protein products. Such signs and symptoms may be treated with acetaminophen and antihistamines.

The decision of whether to complete the infusion of study medication if symptoms improve or have resolved will be left to the medical judgment of the Investigator.

7.1.5. *Overdosage*

Doses of Abatacept up to 50 mg/kg have been administered intravenously without apparent toxic effect. In the case of overdosage, it is recommended that patients should be monitored for any signs or symptoms of adverse reactions and have the appropriate symptomatic treatment instituted immediately.

7.1.6. *Preparation/Handling/Storage/Accountability*

7.1.6.1. *Acquisition and Accountability*

Investigational products (IP) will be shipped to the site either directly from participating companies, from the Sponsor, or from other regional or local drug repositories. All other supplies should be provided by the site. Multiple lots of each IP may be supplied.

Study products received at the sites will be open label and not kit specific, unless specified in the protocol-specific Manual of Procedures (MOP) or pharmacy manual. Drug preparation will be performed by the participating site’s unblinded research pharmacist on the same day of administration to the participant. See the MOP Appendices for detailed information on the preparation, labeling, storage, and administration of investigational products.

Accountability:

The site PI is responsible for study product distribution and disposition and has ultimate responsibility for study product accountability. The site PI may delegate to the participating site’s research pharmacist responsibility for study product accountability. The participating site’s research pharmacist will be responsible for maintaining complete records and documentation of study product receipt, accountability, dispensation, storage conditions, and final disposition of the study product(s). Time of study drug administration to the participant will be recorded on the appropriate data collection form (CRF). All study product(s), whether administered or not, must be documented on the appropriate study product accountability record or dispensing log. The Sponsor’s monitoring staff will verify the participating site’s study product accountability records and dispensing logs per the site monitoring plan. Refer to the protocol-specific MOP for details on storing study medications.

Destruction:

After the study treatment period has ended or as appropriate over the course of the study after study product accountability has been performed, used active and placebo product can be destroyed on-site following applicable site procedures with a second staff member observing and verifying the destruction.

Unused product at the end of the study should be saved until instructed by the Sponsor.

7.1.6.2. *Formulation, Appearance, Packaging, and Labeling*

Abatacept component:

Abatacept for Injection is a lyophilized powder for intravenous infusion. ORENCIA for Injection is supplied as a sterile, white, preservative-free, lyophilized powder for reconstitution and dilution prior to intravenous administration. Following reconstitution of the lyophilized powder with 10 mL of Sterile Water for Injection, USP, the solution of abatacept is clear, colorless to pale yellow, with a pH range of 7.2 to 7.8. Each single-use vial of abatacept for Injection provides 250 mg abatacept, maltose (500 mg), monobasic sodium phosphate (17.2 mg), and sodium chloride (14.6 mg) for administration. It is supplied as an individually packaged, single-use vial with a silicone-free disposable syringe, providing 250 mg of abatacept in a 15-mL vial.

Injectable placebo component:
Injectable placebo will be normal IV saline (0.9% NaCl)made up unblinded by the resident pharmacist and blinded to the study team. Note that the type of infusion bag used for placebo should be identical to that used for the preparation of the abatacept infusion in order to ensure the blind in the study.

7.1.6.3. *Product Storage and Stability*

Abatacept should be stored at 2°C-8°C and protected from light.

7.1.6.4. *Preparation*

Refer to the protocol-specific MOP for details about preparation.

Use aseptic technique.
abatacept for Injection is provided as a lyophilized powder in preservative-free, single-use vials. Each ORENCIA vial provides 250 mg of abatacept for administration. The abatacept powder in each vial must be reconstituted with 10 mL of Sterile Water for Injection, USP, using *only the silicone-free disposable syringe provided with each vial* and an 18- to 21-gauge needle. After reconstitution, the concentration of abatacept in the vial will be 25 mg/mL. If the abatacept powder is accidentally reconstituted using a siliconized syringe, the solution may develop a few translucent particles. Discard any solutions prepared using siliconized syringes.

If the *silicone-free disposable syringe* is dropped or becomes contaminated, use a new *silicone-free disposable syringe* from inventory.

1) Use 10 mL of Sterile Water for Injection, USP to reconstitute the abatacept powder. To reconstitute the abatacept powder, remove the flip-top from the vial and wipe the top with an alcohol swab. Insert the syringe needle into the vial through the center of the rubber stopper and direct the stream of Sterile Water for Injection, USP, to the glass wall of the vial. Do not use the vial if the vacuum is not present. Rotate the vial with gentle swirling to minimize foam formation, until the contents are completely dissolved. Do not shake. Avoid prolonged or vigorous agitation.
2) Upon complete dissolution of the lyophilized powder, the vial should be vented with a needle to dissipate any foam that may be present. After reconstitution, each milliliter will contain 25 mg (250 mg/10 mL). The solution should be clear and colorless to pale yellow. Do not use if opaque particles, discoloration, or other foreign particles are present.
3) The reconstituted abatacept solution must be further diluted to 100 mL as follows. From a 100 mL infusion bag or bottle, withdraw a volume of 0.9% Sodium Chloride Injection, USP, equal to the volume of the

reconstituted abatacept solution required for the patient's dose. Slowly add the reconstituted abatacept solution into the infusion bag or bottle using the same *silicone-free disposable syringe provided with each vial*. Gently mix. *Do not shake the bag or bottle*. The final concentration of abatacept in the bag or bottle will depend upon the amount of drug added but will be no more than 10 mg/mL. Any unused portions in the abatacept vial must be immediately discarded.

4) Prior to administration, the abatacept solution should be inspected visually for particulate matter and discoloration. Discard the solution if any particulate matter or discoloration is observed.
5) The entire, fully diluted abatacept solution should be administered over a period of 30 minutes and must be administered with an infusion set and a *sterile, non-pyrogenic, low-protein-binding filter* (pore size of 0.2 µm to 1.2 µm).
6) The infusion of the fully diluted abatacept solution must be completed within 24 hours of reconstitution of the ORENCIA vials. The fully diluted abatacept solution may be stored at room temperature or refrigerated at 2ºC to 8ºC (36ºF to 46ºF) before use. Discard the fully diluted solution if not administered within 24 hours.
7) Abatacept should not be infused concomitantly in the same intravenous line with other agents. No physical or biochemical compatibility studies have been conducted to evaluate the coadministration of abatacept with other agents.

7.2. Measures to Minimize Bias: Randomization and Blinding

Randomization will be conducted as specified in the Master Protocol.

7.3. Study Intervention Compliance

Each dose of study product will be administered by a member of the clinical research team who is qualified and licensed to administer the study product. Administration and date, and time, will be entered into the case report form (CRF).

7.4. Concomitant Therapy

7.4.1. *Permitted Concomitant Therapy and Procedures*

All concomitant therapies permitted in the master protocol will be allowed for this sub-study.

Of specific note to cotreatment with corticosteroids, Orencia is routinely co-administered with low dose corticosteroids (CS, prednisone or equivalent ≤ 10 mg/day) in its labeled indications for the treatment of Rheumatoid Arthritis, Psoriatic Arthritis and Juvenile Idiopathic Arthritis and is not associated with increased risk of adverse outcomes. Concomitant use of abatacept with higher doses of CS has been studied in other disease areas, e.g. lupus nephritis. Based on clinical trial data, use of high dose CS with sustained abatacept treatment may be associated with some increased risk for serious bacterial infections.

7.4.2. *Prohibited Concomitant Therapy*

All concomitant therapies prohibited in the master protocol will also be prohibited for this sub-study.

7.4.3. *Rescue Medicine*

Not Applicable.

7.4.4. *Non-Research Standard of Care*

Not Applicable.

8. STUDY INTERVENTION DISCONTINUATION AND SUBJECT DISCONTINUATION/WITHDRAWAL

8.1. Halting Criteria and Discontinuation of Study Intervention

8.1.1. *Individual Study Product Halting*

Individual study product halting will be managed according to the Master Protocol.

Abatacept Halting

If there is evidence of toxicity (e.g., infusion reaction) that, in the judgment of the Investigator, could place the participant at increased risk, study drug administration should be discontinued.

8.1.2. *Study Halting*

Study halting will be managed as specified by the Master Protocol.

8.2. Withdrawal from the Study

Sub-study will have the same requirements as the main study.

8.3. Lost to Follow-Up

Sub-study will have the same requirements as the main study.

8.4. Readmission

Sub-study will have the same requirements as the main study.

9. STUDY ASSESSMENTS AND PROCEDURES

Sub-study will have the same assessments and procedures as the main protocol.

10. STATISTICAL CONSIDERATIONS

Statistical analysis will be performed as described within the main body of the ACTIV-1 IM master protocol.

APPENDIX 3 – SUB-STUDY 3 – CENICRIVIROC (CVC)

1. SUB-STUDY SUMMARY

1.1. Sub-study overview

The sub-study population corresponds to moderately and severely ill patients infected with the COVID-19 virus. Recruitment will target patients already hospitalized for treatment of COVID-19 infection as well as patients being treated for COVID-19 infection in Emergency Departments while waiting to be admitted to the hospital. Patients both in and out of the ICU are included in the study population.

Enrollment will begin as soon as all elements are met for the sub-study to activate.

The time on drug period is 28 days (i.e., up to Day 29 of the study), with assessments on each day of the hospital stay. Patients will be followed after hospital discharge and after last dose of drug, with periodic follow-up assessments through Day 60 (i.e., approximately 30 days after last dose of drug).

1.2. Enrollment Period

Enrollment is expected to begin in August 2020. It is anticipated the enrollment may be completed in 4-6 months.

1.3. General

This sub-study is designed to evaluate Cenicriviroc for the treatment of moderately or severely ill hospitalized patients infected with COVID-19. Sub-study will have the same assessments and procedures as the main protocol.

The effectiveness of Cenicriviroc as add-on therapy to SoC will be evaluated based on the primary endpoint of time to recovery after 28 days. The sample size requirements are based on the ability to detect a moderate improvement in time to recovery (3-4 fewer days).

1.4. Study Population

Hospitalized adults (≥18 years old) with COVID-19, including patients both in and out of the ICU. Patients seeking care for COVID-19 in an Emergency Department (ED) and waiting to be admitted to the hospital are included.

1.5. Inclusion Criteria

Inclusion criteria are outlined in the main body of the ACTIV-1 IM protocol.

1.6. Agent Specific Exclusion Criteria

Use of medications that are contraindicated with CVC and that could not be replaced or stopped during the study period (See **Appendix 3 Table 3** for Disallowed Medications).

1.7. Study Intervention

Therapeutic agents will be evaluated as an add-on therapy to SoC. The study intervention is as outlined in the main body of the ACTIV-1 IM protocol.

For the cenicriviroc (CVC) component, CVC will be administered with food as a 450 mg oral loading dose on Day 1, followed by a 300 mg (150 mg BID) maintenance dose for the duration of the study (through Day 29). The administration window of the loading dose will be within 24 hours of randomization.

Duration of therapy:

- Cenicriviroc Oral component – 28 days, including while hospitalized and post-discharge.

1.8. Schedule of Assessments

Assessments will be performed according to **Table 1-1** in 1.1.8 in the main body of the ACTIV master protocol.

2. JUSTIFICATION FOR SELECTION

Severe pneumonia and respiratory-tree infection caused by coronaviruses such as SARS-CoV and MERS-CoV are often associated with massive inflammatory cell infiltration and elevated pro-inflammatory cytokine and chemokine responses -- resulting in injury to the respiratory tree and an acute respiratory distress syndrome (ARDS). Patients with this cytokine storm have been observed to have high levels of CCL2 (MCP1) and CCL5 (RANTES), likely caused by feedback from SARS-CoV upregulating CCR2 and CCR5 (Law et al. 2009). Elevated CCL5 may lead to severe lung inflammation, ARDS, and death in patients with SARS (Ng et al. 2007) demonstrated that the RANTES-28 G allele, which is associated with high levels of CCL5, was a risk factor for poor outcomes and death in Hong Kong and Beijing Chinese SARS patients. This study noted that these chemokines, released from activated immune cells, not only take part in the anti-viral immune response but are causative of cell damage and organ dysfunction. Further demonstrated was the significant induction of CCR-1, CCR-3 and CCR-5 mRNA expression in SARS-CoV infected dendritic cells (DCs), suggesting the possibility of an autocrine loop facilitating DC trafficking. Moreover, presence of the CCL2 G-2518A allele, which leads to high CCL2 levels, made patients more susceptible to infection with SARS-CoV (Tu et al. 2015). Thus, clinical data demonstrate that both CCL5 and CCL2 play key roles in the inflammatory sequelae and poor clinical outcomes of SARS-CoV infection.

In patients with ARDS, CCL2 is upregulated, inducing the migration of circulating CCR2+ inflammatory cells into alveoli (Zemans and Matthay 2017). Patients with ARDS have elevated levels of CCL2 in BAL fluid compared with controls and greater neutrophil recruitment (Williams et al. 2017).

Rationale for dual targeting of CCR2 and CCR5

Both CCR2 and CCR5 play roles in ARDS and the consequences of SARS-CoV. Rationale of dual inhibition includes: redundancy among these pathways and dual inhibition preventing a spill over that may occur with blocking only one; and, these two routes have complementary roles in infection-associated inflammation and tissue damage. For example, CCR2 targeted action is more critical for inhibition of circulating immune cells (e.g., monocytes, memory T cells), their trafficking, and migration into tissue; CCR5 action is more related to inhibiting tissue-based immune cells, and therefore a somewhat later time window (e.g., macrophages, activated T effector cells, dendritic cells). Both CCR2/5 paths have been shown to be operative, and effects off-set by attenuation of CCR2/5 has been shown to be operative in: CoV infection; ARDS; and influenza-caused lung inflammation, tissue destruction, and fibrosis. Moreover, specifically with CVC, dual CCR2/5 has been shown to be an effective mechanism in pre-clinical models of liver inflammation/fibrosis and viral encephalitis.

Importantly, shown in a murine model of SARS-CoV infection, there are 2 time windows: 1) before the onset or worsening of pneumonitis when the administration of a CCR2 inhibitor has the highest likelihood of being beneficial to patients with COVID-19 and 2) during the pneumonitis when both CCR2 and CCR5 inhibition may be beneficial, reinforcing the potential role for dual targeting of CCR2/5 (**Appendix 3 Figure 1**) (Chen et al. 2010).

Appendix 3 Figure 1: ***Pathogenesis of SARS-CoV Infection in Senescent Mice***

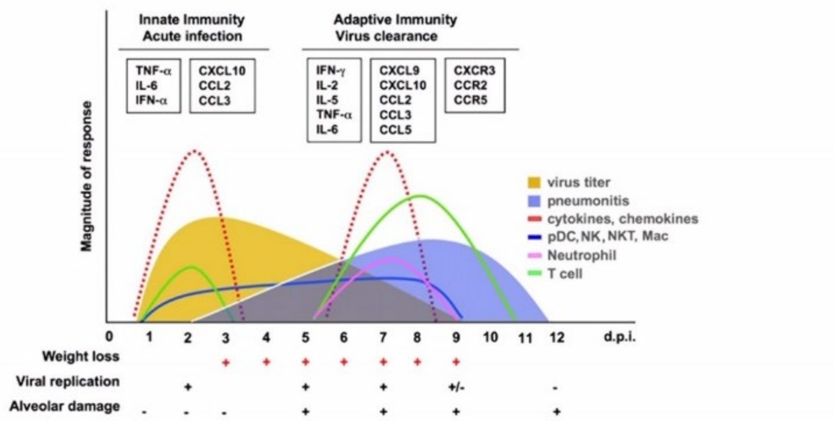

FIG. 7. Time course of host responses to primary SARS-CoV infection in senescent mice. A biphasic expression of inflammatory mediators associated with cellular infiltration into the lungs of infected mice, coincident with peaks in viral replication and clearance, respectively, is seen. Clinical illness such as weight loss was observed coincident with pulmonary viral replication, while lung pathology (pneumonitis) was associated with T-cell infiltration when virus was cleared from the lung.

Cenicriviroc (CVC) is a novel small-molecule, orally active, well-tolerated, potent and selective antagonist of CCR2 and CCR5 with anti-inflammatory and anti-fibrotic activity. Data from animal and clinical studies support a favorable action of CVC in inflammation associated with severe infection. In a mouse model of acute liver injury, administration of CVC significantly decreased the numbers of monocyte-derived macrophages and associated inflammation and tissue damage. CVC also reduced the numbers of Kupffer cells or tissue-based macrophages with a KC-like phenotype peri-injury. Other immune cell populations such as neutrophils or lymphoid cells were not affected (Puengel, Krenkel et al. 2017). In a 2nd mouse model of liver injury, CCR2 inhibition with CVC reduced the influx of proinflammatory monocytes into the liver, which significantly attenuated the early phase of tissue injury and later necrosis (Mossanen et al. 2016). Of note, lung disease models demonstrating infiltrating monocyte-derived interstitial macrophages have a similar inflammatory phenotype, including CCR2/5 based processes, as in the liver disease models, and alveolar macrophages share multiple features with Kupffer cells in the liver. Moreover, immunomodulatory action from CVC has been shown to result in reductions of inflammation and in tissue preservation in murine models of viral encephalitis.

In an ex-vivo study of monocytes derived from HIV-infected patients, dual CCR2/5 inhibition with CVC was more effective in decreasing trans-endothelial migration of monocytes than single CCR2 or CCR5 blockade. Moreover, CVC was also associated with decreased expression of E-selectin on endothelial cells, a major receptor for monocyte recruitment (D'Antoni et al. 2018).

Appendix 3 Figure 2: ***Mechanism of Action for Cenicriviroc (CVC)***

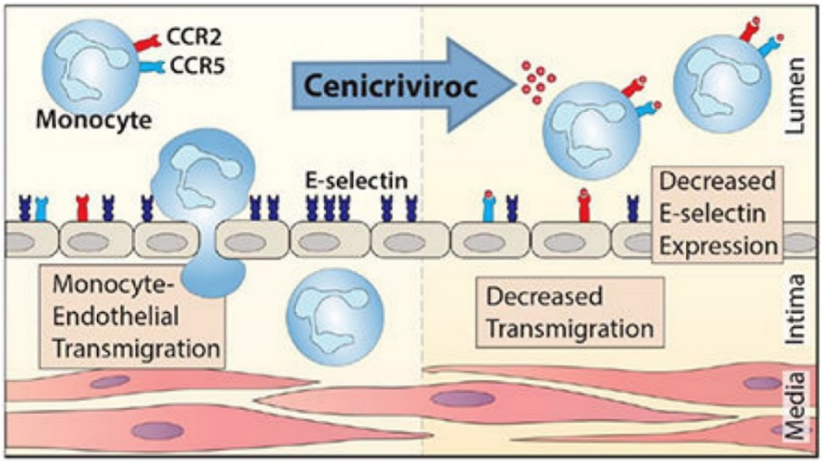

We hypothesize that CVC, by blocking CCR2 and CCR5, will decrease recruitment of infiltrating monocyte-derived interstitial macrophages and other immune cells, known to be operative in respiratory tissues in coronavirus infections, thus preventing the cytokine storm, respiratory injury, and ARDS with COVID-19 (Mossanen et al. 2016; Puengel et al. 2017). Also, we hypothesize that the reduction of trans-endothelial vascular migration of monocytes could play a role in attenuating vascular-based injury. Therefore, we believe that treatment with CVC could prevent the cytokine storm that cause the lung injury and is the cause of acute respiratory distress syndrome (ARDS) in patients with COVID-19.

We propose the investigation of administration of CVC in patients hospitalized due to COVID-19, in those who have not yet developed severe respiratory disease or are early in a course of respiratory compromise, and in those with substantial respiratory compromise, with the objective of reducing the risk of evolution to severe lung injury. The documented safety profile allows the potentially broad treatment of patients, e.g. at several stages of COVID-19.

3. INTRODUCTION

3.1. Risk/Benefit Assessment

3.1.1. *Cenicriviroc*

3.1.1.1. *Potential Risks of Cenicriviroc*

Cenicriviroc (CVC) is contraindicated in participants with clinically significant hypersensitivity to any components of its formulation.

Warnings and Precautions

Treatment-emergent elevations in liver transaminases have been observed in participants receiving CVC treatment. Most elevations were transient, mild to moderate in severity, and resolved upon continued treatment with CVC. Some elevations were severe in intensity and resulted in dose interruption or permanent discontinuation of CVC. CVC has been administered to >2000 patients, including patients with non-alcoholic fatty liver disease (NASH) and a separately to those with HIV, in clinical trials. In NASH patients with pre-existing liver disease, 2 cases of possible autoimmune hepatitis (1 participant in the CVC arm and 1 participant in the placebo arm) have been observed.

Hepatic and Renal Impairment

Based on clinical data (studies 652-1-121 and 3152-102-002), CVC exposures did not increase in participants with mild hepatic impairment (Child-Pugh A), increased in participants with moderate (Child-Pugh B) (AUC0-tau 55%),

and significantly increased in severe (Child-Pugh C) (AUC0-t 40%) hepatic impairment relative to corresponding matched healthy participants. These clinical studies allowed inclusion for ALT or AST abnormalities up to 5x ULN. Of note: based on the PK and safety results for these studies, no CVC dose adjustment will be required in patients with hepatic impairment. Based on results from the human ADME study (Study 3152-103-002), renal excretion of CVC and its metabolites was negligible (< 2% of dose). Consistent with this observation, the renal excretion of CVC (non-radiolabel study) was observed to be < 0.00011% (Study 01-03-TL-652-001). The impact of end stage renal disease on the PK of CVC is under evaluation (Study 3152-104-002).

Drug Interactions

CVC is a substrate of CYP3A4, CYP2C8 and P-gp. CVC is also a weak inhibitor of CYP3A4 and an inhibitor of BCRP and P-gp. The following classes of medications are disallowed at any time during CVC treatment:

- Potent CYP3A4 inhibitors and CYP3A4 inducers will be excluded
- Potent CYP2C8 inhibitors will be excluded
- Drugs with narrow therapeutic windows that are sensitive CYP3A4 substrates will be excluded (i.e., drugs which should not be co-administered with weak CYP3A4 inhibitors such as CVC)
- Appropriate dose adjustments of BCRP substrates are recommended

Co-administration of CVC and remdesivir is allowed.

Co-administration of CVC and glucocorticoids is allowed.

When required, acid-reducing agents should be administered at least 2 hours after the CVC (4 hours for fast-acting antacids). It is recommended to use an H2 receptor antagonist (except cimetidine) or antacids rather than a proton pump inhibitor. It is recommended to start with the lowest dose of these agents and titrate according to clinical response. If lipid lowering medications are used, clinical monitoring and dose titration are recommended to achieve the desired clinical response.

See **Appendix 3 Table 3** and **Appendix 3 Table 4** for fuller instructions regarding allowed concomitant drugs.

In summary, the risks of CVC are low and similar to placebo.

3.1.1.2. *Potential Benefits of Cenicriviroc*

CVC may or may not improve clinical outcome of an individual trial participant with COVID-19. However, there is potential benefit to society from their participation in this study resulting from insights gained about the CVC under study as well as the natural history of the disease. While there may not be benefits for an individual participant, there may be benefits to society if a safe, efficacious therapeutic agent can be identified during this global COVID-19 outbreak.

3.1.1.3. *Assessment of Potential Risks and Benefits of Cenicriviroc*

CVC is generally a well-tolerated medication, which has been administered to participants up to 2 years at different doses. It is well tolerated in a wide dose range. There are no significant toxicities that have been observed in prior studies. An increase of nasopharyngitis in the second year of administration without an increase in upper respiratory tract infections has been reported from a long-term exposure trial (2 years). Since the drug is administered for only 28 days we do not expect any of those side effects. The loading dose of 450 mg on day one will be split into two doses (300 mg (AM) and 150 mg (PM)); note: participants enrolled in the study in the latter portion of the day may receive only the 300 mg component of the Day 1 loading dose (see **Appendix 3 Table 2** for specific dosing scenarios). Additional side effects are not expected. Clinical and pharmacologic assessments support this dose, and supportive data exist for significantly higher doses.

4. OBJECTIVES AND ENDPOINTS

The overall objective of the study is to evaluate the clinical efficacy and safety of different investigational therapeutics relative to the control arm among hospitalized adults who have COVID-19. All the endpoints for the overall trial are defined in the body of the main ACTIV-1 IM protocol.

5. STUDY DESIGN

5.1. Justification for Dose

5.1.1. *Justification for Dose of Cenicriviroc*

To ensure rapid onset of action of CVC, a loading dose of 450 mg will be administered on Day 1 (300 mg first dose in the morning and 150 mg 12 hours later); patients enrolled in the trial in the latter portion of the day may receive only the 300 mg component of the Day 1 loading dose (see **Appendix 3 Table 2** for details on dosing scenarios). Starting Day 2, patients will receive 150 mg of CVC twice per day (total dose of 300 mg per day) until Day 29.

Prior clinical data, clinical pharmacological data, and PK/PD modelling have indicated that CVC inhibited CCR2 (measured as MCP-1 increase) and CCR5 (measured as MIP-1b increase) with an IC_{50} of 25 ng/mL and 43 ng/mL, respectively (3152-102-002: ACCP poster, 2019). This model predicts maximal increase in MCP-1 (~6-fold) and MIP-1b (~2-fold) achieved at steady state by once daily dosing of 150 mg CVC, which reflects complete receptor occupancy. In order to achieve complete receptor occupancy at an earlier time, a loading dose of 300 mg CVC in the morning and 150 mg CVC after 12 hours will be administered: patients enrolled in the trial in the latter portion of the day may receive only the 300 mg component of the Day 1 loading dose (see **Appendix 3 Table 2** for details on dosing scenarios). This dosing regimen predicts achievement of ~90% receptor occupancy by end of Day 1. Thereafter, continued dosing of 150 mg CVC twice daily is expected to maintain effective receptor occupancy for the duration of the treatment.

Moreover, the total dose of 450 mg CVC on Day 1 (split into 300 mg and 150 mg; see **Appendix 3 Table 2** for details on dosing scenarios) is justified based on clinical trials in which multiple doses of CVC up to 900 mg QD for 7 days were administered to healthy volunteers with no safety findings observed. Furthermore, other safety margin data also give support for this dosing regimen. In GLP toxicology studies, the NOAEL was determined to be 2000 mg/kg/day in mice, 100 mg/kg/day in rats, and 1000 mg/kg/day in monkeys. Based on the most sensitive NOAEL dose of 100 mg/kg/day in rats, a HED of 960 mg was estimated. The most commonly observed AEs (≥ 2%) with CVC in multiple-dose studies evaluating daily doses up to 900 mg for ≥ 7 days were headache and constipation.

Furthermore, CVC has been shown to be safe and well-tolerated following multiple-dose administration of 450 mg and 900 mg once daily for 7 days (Study 3152-107-002). In study 3152-107-002, steady state C_{max} of CVC at doses of 450 mg and 900 mg was 2977 ng/mL and 4025 ng/mL, respectively, and the corresponding $AUC_{0-\tau}$ values were 37724 and 72905 ng.h/mL, respectively. Since the Day 1 dose of 450 mg in the current study is being split (as 300 mg in the morning and 150 mg to be administered after 12 hours), and from day 2 onwards the dose of 300 mg is split (as 150 mg BID), the maximal CVC concentrations (Cmax) as well as steady state AUC (AUC_t) in this study are expected to be less than those observed in earlier clinical studies (3152-107-002 and 3152-106-002). Note: patients enrolled in the trial in the latter portion of the day may receive only the 300 mg component of the Day 1 loading dose (see **Appendix 3 Table 2** for details on dosing scenarios).

Since food has been shown to have significant effect on CVC exposure, all doses are recommended to be administered within 30 minutes of consumption of a meal (or feeds), which is also how CVC has been administered in other clinical trials.

Pharmacokinetic Modelling Assessments for Dose Rationale

CVC is a dual antagonist of CCR2 and CCR5 receptors. Increase in serum concentrations of ligands of CCR2 (MCP-1) and CCR5 (MIP-1β) is used as a surrogate marker (biomarker response) to determine CVC receptor occupancy in clinical studies. In an earlier Phase 1 clinical study (Study 3152-105-002), exposure response relationship between plasma CVC concentrations and biomarker response (MCP-1 or MIP-1β) were explored using a sequential PK/PD model (**Appendix 3 Figure 3**). The structural PK model was a two-compartment model with

first-order absorption and with a lag time. The exposure response for each biomarker was modelled by an indirect pharmacodynamic response model. The kinetic parameter estimates from the indirect response model are summarized in **Appendix 3 Table 1**.

Appendix 3 Figure 3: ***Sequential approach of population PK and Indirect response model utilized to determine exposure response***

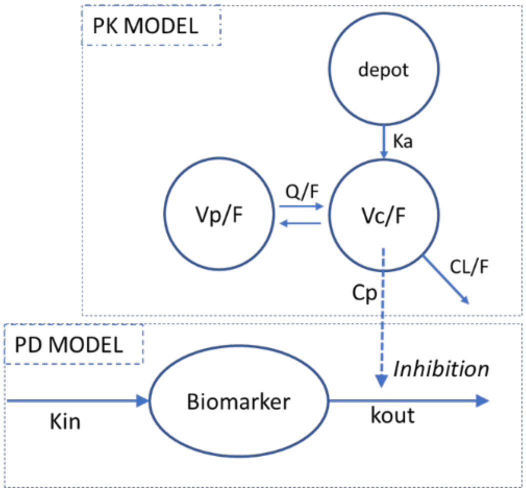

Appendix 3 Table 1: ***Parameter Estimates for Indirect Response Model***

	MCP-1 [pg/mL]			MIP-1B [pg/mL]		
	Typical Value	RSE(%)	IIV	Typical Value	RSE(%)	IIV
Baseline [pg/mL]	339	3.8	18%	98	7.8	38%
kout [1/h]	0.60	fixed	n.e.	0.055	9.8	n.e.
Imax [-]	0.90	fixed	n.e.	0.62	fixed	n.e.
IC50 [ng/mL]	25	63	78%	43	31	117%
Proportional Residual Error	19%	12	n.e.	12%	5.7	n.e.

RSE – relative standard error for estimated typical value; IIV – inter-individual variability; n.e. – not estimated; Kin=Baseline*kout; %inhibition of kout = Cp*Imax/(Cp+IC50); "fixed" - to aid a stable estimation, some parameter values were fixed in final model based on values obtained in iterative model runs and evaluated through numerical and visual goodness-of-fit.

Based on these parameters, the biomarker response (% change from baseline of MCP-1 or MIP-1β) was predicted following once daily dosing of 150 mg CVC (**Appendix 3 Figure 4)**. These simulations suggest maximal change in MCP-1 levels (~5.5-fold increase) and MIP-1β (~2-fold) upon once daily dosing of CVC for at least 5 days and 10 days, respectively.

Appendix 3 Figure 4: ***Predicted biomarker response following once daily dosing of 150 mg CVC***

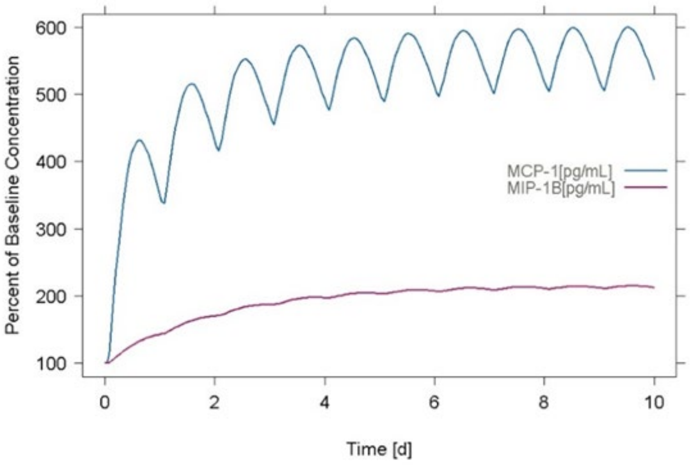

Since treatment of fibrosis in NASH patients require chronic administration of CVC (AURORA study duration: 5 years of once daily treatment of 150 mg CVC) and the earliest change in endpoint (reduction in fibrosis) is expected after once daily treatment for 1 year, achieving maximal biomarker change after 5 to 10 days of once daily treatment is acceptable in NASH indication. However, COVID-19 is a severe acute infectious disease that requires rapid onset of action and relatively short duration of treatment (planned duration of treatment 28 days). Therefore, dosing regimen containing a total loading dose of 450 mg CVC (divided as 300 mg in the morning + 150 mg in the evening) was proposed on Day 1 of this study followed by dose of 300 mg/day (divided as 150 mg in the morning plus 150 mg in the evening). For the proposed dosing regimen, the biomarker response (% change from baseline of MCP-1 or MIP-1β) was predicted as shown (**Appendix 3 Figure 5**). As per simulations, loading dose is expected to increase MCP-1 levels by 6-fold and MIP-1β levels by 1.5-fold on Day 1. Following twice-daily administration of 150 mg CVC, maximal increase in MCP-1 (~7-fold) and MIP-1β (~2-fold) were sustained during the duration of treatment beginning Day 2. These simulations suggest effective blockade of activation of CCR2 and CCR5 receptors by respective ligands could be achieved rapidly and sustained by following the proposed dosing regimen.

Appendix 3 Figure 5: ***Predicted biomarker response following proposed dosing regimen of CVC (Day 1: 300 mg in the morning + 150 mg in the evening, Day 2 to Day 29: 150 mg twice daily)***

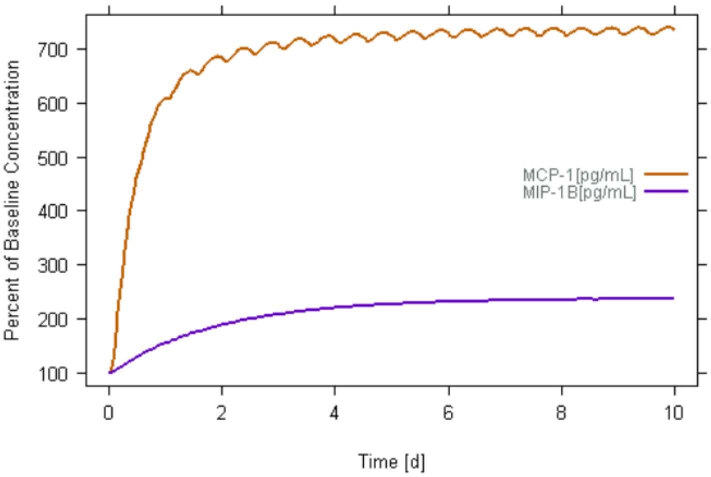

As of January 2020, approximately 2000 participants have been exposed to either single or multiple doses of CVC in completed and ongoing clinical studies. CVC doses explored in these clinical studies range from 25 mg to 900 mg

across all CVC studies. There was no apparent dose- or exposure-relationship for safety observed. Furthermore, splitting the daily dose is expected to limit the plasma peak concentrations of CVC corresponding to 300 mg dose on Day 1 and 150 mg dose from Day 2 onwards. In addition, food effect on the bioavailability of CVC formulation is significant (up to 5.2-fold increase with standard breakfast). The CVC dose can be administered to an intubated and severely ill COVID-19 patient via G-tube and with liquid food. In such a case, if there were to be reduced enteral absorption, then any lowered plasma concentrations/exposure of CVC may be partially offset by the twice-daily dosing of CVC.

In summary, these data provide support and rationale for the proposed dosing being well within appropriate parameters and safety. The sponsor believes the proposed loading dose (450 mg on Day 1) and twice daily administration of 150 mg CVC are clearly justified based on the following: safe use of CVC doses up to 900 mg in prior clinical studies; immediate need for optimal plasma exposure of CVC in patients with COVID-19 infection, ensure full antagonistic activity against CCR2 and CCR5 receptors in target tissues; and maintenance of such exposure throughout the duration of treatment without compromising patient safety.

6. STUDY POPULATION

The study population will adhere to the master protocol population, except for the noted additional exclusions and considerations below.

6.1. Inclusion Criteria

The same as the master protocol.

6.2. Sub-Study Specific Exclusion Criteria

Use of medications that are contraindicated with CVC and that could not be replaced or stopped during the study period (See **Appendix 3 Table 3** for Disallowed Medications).

6.3. Exclusion of Specific Populations

Children and adolescents will not be included in this trial.

No data currently exist for the effects of CVC on reproduction and development in humans. Pregnancy should be avoided in women receiving this compound. Women of childbearing potential should undergo pregnancy testing prior to initiation of CVC and periodically during treatment. In addition, adequate contraceptive methods should be consistently used by males and females during and for 1 month after treatment with CVC.

CVC is contraindicated in nursing mothers. CVC and its metabolites were detected in milk secreted from rats administered CVC. CVC milk to plasma ratios ranged from 0.7 to 0.8 in dams indicating that milk represents a potential route of exposure to CVC in rat pups. The extent to which CVC or any of its metabolites are excreted in human breast milk is not known.

7. STUDY PRODUCT

7.1. Study Product(s) and Administration

Participants will be randomized according to the procedure described in the master protocol.

If there are supply limitations on any product, the arms containing that product will be temporarily closed to enrollment and the corresponding placebo is not needed.

7.1.1. *Study Product Description*

The CVC drug product is a solid oral dosage (tablet form) containing 150 mg of the active ingredient as a free base equivalent. The CVC 150 mg tablets are capsule-shaped, 15.7 × 7.9 mm in diameter and plain on both sides. The

inactive ingredients of CVC tablets include fumaric acid, microcrystalline cellulose, croscarmellose sodium, colloidal silicon dioxide and magnesium stearate, which is film coated with an Opadry II yellow, a polyethylene glycol (PEG) and partially hydrolyzed polyvinyl alcohol (PVA) non-functional film coat.

7.1.2. *Dosing and Administration*

For the CVC component, treatment will be administered to participants while hospitalized as in-patients and will continue whether or not the patient is discharged from the hospital for a total 28-day course. CVC should be administered twice daily at approximately 12-hour intervals in fed condition and at approximately the same time each day (±2 hours). Patients may receive a larger dose (450 mg total) for their first day of treatment as a loading dose administered as a morning dose of 300 mg and an evening dose of 150 mg. The administration window will be within 24 hours of randomization. See **Appendix 3 Table 2** for further details.

Doses should be administered with food (or tube-feeds). For participants requiring mechanical ventilation, Investigators will need to determine the most appropriate means for providing the drug product with food (i.e., crushed tablet by feeding tube with adequate flush); preparation instructions for administration of CVC using a G-tube or other enteral feeding tube are in the Pharmacy Manual. If a feeding tube is not available for >24 hours, the participant will discontinue study drug intervention, but will remain in the study for endpoints and safety observations.

Appendix 3 Table 2: Dosing of CVC Depending on Time of Day of Randomization (Scenarios)

	**Scenario 1**		**Scenario 2**		**Scenario 3**	
	Participant is randomized and entered into the system for first-dose of Day 1 with breakfast		Participant is randomized and entered into the system for first-dose of Day 1 with lunch or early afternoon snack/meal		Participant is randomized and entered into the system for first-dose of Day 1 with dinner; 2nd dose of Day 1 is N/A	
BID Dose	*Dose 1*	*Dose 2*	*Dose 1*	*Dose 2*	*Dose 1*	*Dose 2*
**Day 1 (Loading Dose, mg)**	*300*	*150*	*300*	*150*	*300*	N/A
Time of dosing	8:00 AM	8:00 PM	11:30 AM	11:30 PM	6:00 PM	N/A
Acceptable Time Range	6 – 10 AM	6 – 10 PM	9:30 AM – 1:30 PM	9:30 PM – 1:30 AM	4 – 8 PM	N/A
**Days 2-28 (Dose, mg)**	*150*	*150*	*150*	*150*	*150*	*150*
Time of dosing	8:00 AM	8:00 PM	8:00 AM	8:00 PM	8:00 AM	8:00 PM
Acceptable Time Range	6 – 10 AM	6 – 10 PM	6 – 10 AM	6 – 10 PM	6 – 10 AM	6 – 10 PM

Any dose that is delayed may be given later that calendar day. Any dose that is missed (not given that calendar day) is not made up. The treatment course continues as described above even if the participant becomes PCR negative.

Duration of therapy:

- Cenicriviroc (Oral) – 28 days (i.e., up to Day 29 of study), including while hospitalized and post-discharge.

7.1.3. *Dose Escalation*

Not Applicable

7.1.4. *Dose Modifications*

See Table in section 7.4.2, Allowed Medications Requiring Dose Adjustments.

7.1.4.1. *Dose Modifications for Adverse Events*

If there is evidence of toxicity that, in the judgment of the Investigator, could place the participant at increased risk, study drug administration should be interrupted. Resumption of dosing should be discussed with the Sponsor.

Any potential dose modifications of CVC need to be discussed with the Sponsor.

Liver Assessment: Suggested Actions and Follow-up

Close monitoring should be initiated for the following participants:

- Participants enrolled with a normal baseline serum aminotransferases (AT) who develop an increase of serum AT≥ 3 × ULN, or
- Participants enrolled with elevated baseline AT who develop an increase of serum AT >2 × the baseline value

The participant should be evaluated for liver biochemistry elevation potentially meeting Hy's law criteria (see below)as soon as possible, preferably within 24 to 48 hours from the time the investigator becomes aware of the abnormal results. Evaluation should typically include repeat testing of all 4 of the usual serum biochemical measures (ALT, AST, ALP, and TBL) to confirm the abnormalities and to determine if they are increasing or decreasing. Testing should be repeated until the levels decrease or stabilize.

If it is difficult for the participant to return to the study site promptly, the participant should be retested locally, but normal laboratory ranges should be recorded, results should be made available to sponsor's study physician and the investigator immediately, and the data should be included in the eCRF. If repeat testing within this time frame is not possible, the study intervention should be discontinued.

It is critical to initiate close monitoring immediately upon detection and confirmation of signals of liver biochemistry elevation potentially meeting Hy's law criteria as early as possible and not to wait until the next scheduled visit or monitoring interval. Close monitoring of the participant should be initiated in conjunction with the sponsor and consideration given to the following:

- Obtain a more detailed history of symptoms and prior or concurrent diseases.
- Obtain a history of concomitant drug use, including nonprescription medications, herbal products and dietary supplements, alcohol and recreational drug use, and special diets.
- Obtain a history of exposure to environmental chemical agents.
- Initiation of appropriate evaluations including applicable laboratory tests (e.g., direct bilirubin, INR), physical assessments, and other assessments (e.g., imaging)
    - Rule out other potential causes of biochemical abnormalities, e.g., acute viral hepatitis types A, B, C, D, and E; autoimmune or alcoholic hepatitis; NASH; hypoxic/ischemic hepatopathy; and biliary tract disease.
- Consider gastroenterology or hepatology consultations.

If any of the following criteria are met, discontinuation of study intervention should be considered (if indicated, prior to receipt of confirming retest biochemistry laboratory test results) and the sponsor notified of the discontinuation:

- ALT or AST ≥3 × ULN and the participant is symptomatic with the appearance of fatigue, nausea, vomiting, right upper quadrant pain or tenderness, fever, rash, or eosinophilia (>5%)
- ALT or AST ≥3 × ULN and total bilirubin >2 × ULN or INR >1.5
- ALT or AST ≥5 × ULN for more than 2 weeks
- ALT or AST ≥8 × ULN

All participants showing liver biochemistry elevation meeting potential Hy's law criteria should be followed until all abnormalities return to normal or to the baseline state.

Reporting of Potential Hy's Law Cases

Potential Hy's law cases are defined by biochemical test results of hepatocellular injury and impaired hepatic function. They should be evaluated and followed further (i.e., close monitoring initiated) to determine whether these laboratory abnormalities are indicative of liver biochemistry elevation meeting potential Hy's law criteria. As indicated above, discontinuation of study intervention should also be considered. Criteria that identify a potential Hy's law case are as follows:

- ALT or AST ≥3 × ULN AND
- Total bilirubin ≥2 × ULN AND
- Alkaline phosphatase <2 × ULN

Sites must report every participant who meets the Hy's law criteria if this occurs within the time the participant signs the ICF until 30 days after the last dose of study intervention.

A laboratory alert for a liver biochemistry elevation potentially meeting Hy's law criteria case will be sent immediately to the sponsor and investigators when the above criteria have been met, even if no clinical symptoms have been experienced. An Adverse Event of Interest Abnormal Liver Function Reporting Form should be completed as soon as possible (within 24 hours of notification) for liver biochemistry elevations potentially meeting Hy's law criteria cases and submitted to the Sponsor. The eCRF pages associated with the potential Hy's law cases must be completed within 7 calendar days. Potential Hy's law cases will be evaluated by a hepatologist with expertise in drug-induced liver injury (DILI) and reviewed by the DSMB.

7.1.5. *Overdosage*

No specific information is available on the treatment of overdose of CVC. In the event of overdose, the participant should be observed closely for signs of toxicity. Appropriate supportive treatment should be provided if clinically indicated.

An overdose of CVC, regardless of the presence of an associated SAE, is considered an ECI and must be documented and reported.

Additionally, an SAE associated with an overdose of CVC must be documented and reported according to the requirements for SAEs.

7.1.6. *Preparation/Handling/Storage/Accountability*

7.1.6.1. *Acquisition and Accountability*

Investigational products (IP) will be shipped to the site either directly from participating companies, from the Sponsor, or from other regional or local drug repositories. All other supplies should be provided by the site. Multiple lots of each IP may be supplied.

Study products received at the sites will be open label and not kit specific, unless specified in the protocol-specific Manual of Procedures (MOP) or pharmacy manual. Drug preparation will be performed by the participating site's unblinded research pharmacist on the same day of administration to the participant. See the MOP Appendices for detailed information on the preparation, labeling, storage, and administration of investigational products.

Accountability:

The site PI is responsible for study product distribution and disposition and has ultimate responsibility for study product accountability. The site PI may delegate to the participating site's research pharmacist responsibility for study product accountability. The participating site's research pharmacist will be responsible for maintaining complete records and documentation of study product receipt, accountability, dispensation, storage conditions, and final disposition of the study product(s). Time of study drug administration to the participant will be recorded on the appropriate data collection form (CRF). All study product(s), whether administered or not, must be documented on the appropriate study product accountability record or dispensing log. The Sponsor's monitoring staff will verify the participating site's study product accountability records and dispensing logs per the site monitoring plan. Refer to the protocol-specific MOP for details on storing study medications.

Destruction:

After the study treatment period has ended or as appropriate over the course of the study after study product accountability has been performed, used active and placebo product can be destroyed on-site following applicable site procedures with a second staff member observing and verifying the destruction.

Unused product at the end of the study should be saved until instructed by the Sponsor.

7.1.6.2. *Formulation, Appearance, Packaging, and Labeling*

CVC Component:

The Cenicriviroc mesylate drug product formulation used for clinical trials is DP-7A, it is supplied as an immediate-release tablet for oral administration containing 150 mg of Cenicriviroc as Cenicriviroc mesylate (CVC) drug substance. The 150 mg tablets are yellow-coated. The formulated tablet contains the following excipients: fumaric acid (solubility enhancer), microcrystalline cellulose (filler), croscarmellose sodium (disintegrant), colloidal silicon dioxide (glidant), magnesium stearate (lubricant), and Opadry® II yellow (film coating).

Study drug will be provided in a HDPE bottle containing desiccant, and induction sealed.

Oral placebo component:

Placebo tablets match the active product in appearance. Placebo tablets contain excipients of microcrystalline cellulose, colloidal silicon dioxide, croscarmellose sodium, magnesium stearate, Opadry® II yellow, lactose monohydrate, and FD&C Yellow #5 Aluminum Lake.

7.1.6.3. *Product Storage and Stability*

CVC study drug is to be stored at 15°C to 30°C (59°F to 86°F) with transient excursions permitted to -20°C (-4°F) to 60°C (140°F).

7.1.6.4. *Preparation*

Refer to the protocol-specific MOP for details about preparation.

No agent preparation is required for CVC oral dosing. Preparation instructions for use of CVC with a feeding tube can be found in the Pharmacy Manual.

7.2. Measures to Minimize Bias: Randomization and Blinding

Randomization will as specified in the Master Protocol.

7.3. Study Intervention Compliance

Each dose of study product will be administered by a member of the clinical research team who is qualified and licensed to administer the study product. Administration and date, and time, will be entered into the case report form (CRF).

7.4. Concomitant Therapy

7.4.1. *Permitted Concomitant Therapy and Procedures*

All concomitant therapies permitted in the master protocol will be allowed for this sub-study, except lopinavir/ritonavir, as indicated below.

7.4.2. *Prohibited Concomitant Therapy*

All concomitant therapies prohibited in the master protocol will also be prohibited for this sub-study. Additional prohibited therapies are detailed below.

<u>**CVC Component:**</u>
Caution should always be exercised when administering concomitant medications based on the individual medication profile and clinical risk-benefit assessment. A complete list of prohibited medications is provided in **Appendix 3 Table 3**. Concomitant therapy of particular note include:

- Antiretroviral agents (See the table below [**Appendix 3 Table 3**] for other antiretroviral agents)
  - CVC can be used in combination with remdesivir.
  - Dolutegravir, Tenofovir each has no significant impact on CVC exposure and would be acceptable to co-administer with study intervention in this study.
  - Ritonavir or any combination of a protease inhibitor with ritonavir should not be co-administered with drug product in this study due to a significant drug interaction between ritonavir and CVC.

Appendix 3 Table 3: For CVC, Disallowed Medications

**Disallowed Medications**	
**Antibacterials**	rifampin, nafcillin, clarithromycin, erythromycin, telithromycin
**Anticonvulsants**	carbamazepin, phenytoin
**Antidepressants**	Nefazodone
**Antifungals**	voriconazole, itraconazole, ketoconazole, posaconazole
**Antihistamines**	Astemizole
**Anti-inflammatory drugs**	Sulfasalazine
**Antimetabolite drugs**	Methotrexate
**Antipsychotics**	Pimozide
**Antivirals**	Efavirenz, etravirine, boceprevir, dasabuvir/ombitasvir/paritaprevir/ritonavir, indinavir, lopinavir/ritonavir, nelfinavir, ombitasvir/paritaprevir/ritonavir, ritonavir, saquinavir, telaprevir, glecaprevir/pibrentasvir
**Ade Ergot Alkaloids**	dihydroergotamine, ergonovine, ergotamine, methylergonovine
**Lipid-lowering agents**	Gemfibrozil
**Other**	Cisapride

Appendix 3 Table 4: Allowed Medications Requiring Dose Adjustments

Allowed Medications Requiring Dose Adjustments	
**Opioids:**<br>**Fentanyl, Alfentanil**	The preference for opioid use for analgesia is to employ a non-fentanyl-based strategy (e.g., dilaudid). **Note: fentanyl and alfentanil are CYP3A4 substrates, thus use of either with CVC may increase fentanyl and alfentanil exposure.**<br><br>If needed, fentanyl/alfentanil should be administered with close surveillance and a dose-titration strategy:<br>• If feasible, it is recommended that the initial dose be decreased by 50% and up titrated to the desired effect.<br>• If a customary starting-dose is needed in the Investigator's judgment, should be down-titrated after initiation
**Sedative/hypnotics: midazolam, triazolam**	The preference for sedation is to employ a non-midazolam-based strategy (e.g., propofol). **Note: the exposure of midazolam (a substrate of CYP3A4) can be increased 1.84-fold when co-administered with CVC.**<br><br>If needed, midazolam or triazolam should be administered with close surveillance and a dose-titration strategy:<br>• If feasible, it is recommended that the initial dose be decreased by 50% and up titrated to the desired effect.<br>• If a customary starting-dose is needed in the investigator's judgement, should be down-titrated after initiation
**Immunosuppressants: cyclosporin, tacrolimus**	In patients actively receiving cyclosporine or tacrolimus, CVC should be given as 150 mg QD instead of 150 mg BID.
**Gastric acid-reducing agents (H2 receptor antagonists, antacids, proton-pump inhibitors [PPIs])**	Gastric acid-reducing agents should be administered at least 2 hours after CVC dosing (4 hours after dosing is ideal for fast-acting antacids). When possible, an H2 receptor antagonist (except cimetidine) or antacid is preferred over a proton-pump inhibitor (PPI). It is recommended to start with the lowest dose of these agents and titrate appropriately.<br>• H2 receptor antagonists (e.g., famotidine or ranitidine) should not exceed a dose comparable to famotidine 40 mg daily.<br>• Antacids (e.g., aluminum hydroxide, calcium carbonate, magnesium carbonate, magnesium hydroxide, or bismuth subsalicylate) should be given at least 4 hours after administration of study drug.<br>• PPIs (e.g., omeprazole, lansoprazole, esomeprazole, pantoprazole, rabeprazole, dexlansoprazole) are not recommended, however if needed, administer approximately 3 hours after study drug at a dose that does not exceed doses comparable to omeprazole 20 mg daily. Note: due to the prolonged acid-reducing effect of PPIs (~16-24 hours), it is advised to follow these recommendations to reduce their potential impact on absorption of the subsequent CVC dose.

Allowed Medications Requiring Dose Adjustments	
**Lipid-lowering agents: atorvastatin, simvastatin, lovastatin, pravastatin, rosuvastatin**	The maximum recommended daily doses are as follows:<br>• atorvastatin 40 mg,<br>• simvastatin 20 mg,<br>• lovastatin 40 mg,<br>• pravastatin 40 mg, and<br>• rosuvastatin 20 mg;<br>• pitavastatin use is allowed without dose restriction<br><br>The medical monitor or equivalent representative must be consulted prior to use of higher doses of statins than those recommended above.
**PDE5 enzyme inhibitors: sildenafil, tadalafil, vardenafil**	The recommended starting doses for these medications are as follows:<br>• sildenafil 25 mg,<br>• tadalafil 2.5 mg,<br>• vardenafil 2.5 mg
**Anticoagulants**	The recommended instructions for these medications are as follows:<br>• rivaroxaban - if required, do not exceed 10 mg<br>• apixaban and edoxaban – may increase CVC exposure, use with reduced doses and close clinical monitoring

7.4.3. *Rescue Medicine*

Not Applicable.

7.4.4. *Non-Research Standard of Care*

Not Applicable.

8. STUDY INTERVENTION DISCONTINUATION AND SUBJECT DISCONTINUATION/WITHDRAWAL

8.1. Halting Criteria and Discontinuation of Study Intervention

8.1.1. *Individual Study Product Halting*

Individual study product halting will be managed as specified in the Master Protocol.

Cenicriviroc Halting

See Section 7.1.4.1 for information about dosing modifications and potential discontinuation of CVC due to laboratory abnormalities.

8.1.2. *Study Halting*

Study halting will be managed as specified in the Master Protocol.

8.2. Withdrawal from the Study

Sub-study will have the same requirements as the main study.

8.3. Lost to Follow-Up

Sub-study will have the same requirements as the main study.

8.4. Readmission

Sub-study will have the same requirements as the main study.

9. STUDY ASSESSMENTS AND PROCEDURES

Sub-study will have the same assessments and procedures as the main protocol.

Day 8 and Day 29 PK blood samples are optimally drawn 3-6 hours post dosing of drug.

In addition, the CVC assessments will include the following PK blood collections (4 mL each):

- If participant requires an enteral tube for feeding: Three days post-feeding-tube placement, for patients receiving CVC through a feeding tube (two PK blood draws: pre-dose; and 3-6 hours post-dose). (Total blood collected: 8 mL)
- If participant requires an ECMO: One blood draw upon initiation of ECMO, and two blood draws on the third day of ECMO (pre-dose; and 3-6 hours post-dose). (Total blood collected: 12 mL)

10. STATISTICAL CONSIDERATIONS

Statistical analysis will be performed as described within the main body of the ACTIV-1 IM master protocol.

11. REFERENCES

Abatacept US Prescribing Information.

Alten, R., C.O. Bingham, 3rd, S.B. Cohen, J.R. Curtis, S. Kelly, D. Wong and M.C. Genovese (2016). "Antibody response to pneumococcal and influenza vaccination in patients with rheumatoid arthritis receiving abatacept." BMC Musculoskelet Disord **17**: 231.

Beigel, J., K.M. Tomashek, L.E. Dodd, A.K. Mehta, B.S. Zingman, A.C. Kalil, E. Hohmann, H.Y. Chu, A. Luetkemeyer, S.Kline, D. Lopez de Castilla, R.W. Finberg and ACTT-1 Study Group Members (2020). "Remdesivir for the Treatment of Covid-19 – Preliminary Report." N Engl J Med NEJMoa2007764.

Braun, J. and J. Sieper (2003). "Role of novel biological therapies in psoriatic arthritis: effects on joints and skin." BioDrugs **17**(3): 187-199.

Brenner, E.J., R.C. Ungaro, R.B. Gearry, G.G. Kaplan, M. Kissous-Hunt, J.D. Lewis, S.C. Ng, J.F. Rahier, W. Reinisch, F.M. Ruemmele, F. Steinwurz, F.E. Underwood, X. Zhang, J.F. Colombel and M.D. Kappelman (2020). "Corticosteroids, but not TNF Antagonists, are Associated with Adverse COVID-19 Outcomes in Patients With Inflammatory Bowel Diseases: Results from an International Registry." Gastroenterology **159**(2): 481-491.e3.

Buch, M.H., D.L. Boyle, S. Rosengren, B. Saleem, R.J. Reece, L.A. Rhodes, A. Radjenovic, A. English, H. Tang, G. Vratsanos, P. O'Connor, G.S. Firestein and P. Emery (2009). "Mode of action of abatacept in rheumatoid arthritis patients having failed tumour necrosis factor blockade: a histological, gene expression and dynamic magnetic resonance imaging pilot study." Ann Rheum Dis **68**(7): 1220-1227.

Carvajal Alegria, G., P. Pochard, J.O. Pers and D. Cornec (2016). "Could abatacept directly target expanded plasmablasts in IgG4-related disease?" Ann Rheum Dis **75**(11): e73.

Chambers, C.A. and J.P. Allison (1999). "CTLA-4--the costimulatory molecule that doesn't: regulation of T-cell responses by inhibition." Cold Spring Harb Symp Quant Biol **64**: 303-312.

Chen, J., Y.F. Lau, E.W. Lamirande, C.D. Paddock, J.H. Bartlett, S.R. Zaki and K. Subbarao (2010). "Cellular immune responses to severe acute respiratory syndrome coronavirus (SARS-CoV) infection in senescent BALB/c mice: CD4+ T cells are important in control of SARS-CoV infection." J Virol **84**(3): 1289-1301.

Chen, S.K., K.P. Liao, J. Liu and S.C. Kim (2020). "Risk of Hospitalized Infection and Initiation of Abatacept Versus Tumor Necrosis Factor Inhibitors Among Patients With Rheumatoid Arthritis: A Propensity Score-Matched Cohort Study." Arthritis Care Res (Hoboken) **72**(1): 9-17.

Chen, X., B. Zhao, Y. Qu, Y. Chen, J. Xiong, Y. Feng, D. Men, Q. Huang, Y. Liu, B. Yang, J. Ding and F. Li (2020). "Detectable serum SARS-CoV-2 viral load (RNAaemia) is closely correlated with drastically elevated interleukin 6 (IL-6) level in critically ill COVID-19 patients." Clin Infect Dis ciaa449.

Chung, E. S., M. Packer, K.H. Lo, A.A. Fasanmade and J.T. Willerson (2003). "Randomized, double-blind, placebo-controlled, pilot trial of infliximab, a chimeric monoclonal antibody to tumor necrosis factor-alpha, in patients with moderate-to-severe heart failure: results of the anti-TNF Therapy Against Congestive Heart Failure (ATTACH) trial." Circulation **107**(25): 3133-3140.

Company, B.M.S. (2020a). Abatacept (BMS-188667) Investigator Brochure Version 23.

Company, B.M.S. (2020b). Abatacept (BMS-188667) Investigator Brochure Addendum No. 1.

Conti, P., G. Ronconi, A. Caraffa, C.E. Gallenga, R. Ross, I. Frydas and S.K. Kritas (2020). "Induction of pro-inflammatory cytokines (IL-1 and IL-6) and lung inflammation by Coronavirus-19 (COVI-19 or SARS-CoV-2): anti-inflammatory strategies." J Biol Regul Homeost Agents **34**(2): 327-331.

Curtis, J.R., S. Yang, N.M. Patkar, L. Chen, J.A. Singh, G.W. Cannon, T.R. Mikuls, E. Delzell, K.G. Saag, M.M. Safford, S. DuVall, K. Alexander, P. Napalkov, K.L. Winthrop, M.J. Burton, A. Kamauu and J.W. Baddley (2014). "Risk of hospitalized bacterial infections associated with biologic treatment among US veterans with rheumatoid arthritis." Arthritis Care Res (Hoboken) **66**(7): 990-997.

Cutolo, M., S. Soldano, P. Montagna, A. Sulli, B. Seriolo, B. Villaggio, P. Triolo, P. Clerico, L. Felli and R. Brizzolara (2009). "CTLA4-Ig interacts with cultured synovial macrophages from rheumatoid arthritis patients and downregulates cytokine production." Arthritis Res Ther **11**(6): R176.

D'Antoni, M. L., B.I. Mitchell, S. McCurdy, M.M. Byron, D. Ogata-Arakaki, D. Chow, N.N. Mehta, W.A. Boisvert, E. Lefebvre, C.M. Shikuma, L.C. Ndhlovu and Y. Baumer (2018). "Cenicriviroc inhibits trans-endothelial passage of monocytes and is associated with impaired E-selectin expression." J Leukoc Biol **104**(6): 1241-1252.

Davis, P. M., S.G. Nadler, D.K. Stetsko and S.J. Suchard (2008). "Abatacept modulates human dendritic cell-stimulated T-cell proliferation and effector function independent of IDO induction." Clin Immunol **126**(1): 38-47.

Delmonte, O.M., R. Castagnoli, E. Calzoni and L.D. Notarangelo (2019). "Inborn Errors of Immunity With Immune Dysregulation: From Bench to Bedside." Front Pediatr **7**: 353.

Feldmann, M., R.N. Maini, J.N. Woody, S.T. Holgate, G. Winter, M. Rowland, D. Richards and T. Hussell (2020). "Trials of anti-tumour necrosis factor therapy for COVID-19 are urgently needed." Lancet **395**(10234): 1407-1409.

Haga, S., N. Yamamoto, C. Nakai-Murakami, Y. Osawa, K. Tokunaga, T. Sata, N. Yamamoto, T. Sasazuki and Y. Ishizaka (2008). "Modulation of TNF-alpha-converting enzyme by the spike protein of SARS-CoV and ACE2 induces TNF-alpha production and facilitates viral entry." Proc Natl Acad Sci U S A **105**(22): 7809-7814.

Huang, C., Y. Wang, X. Li, L. Ren, J. Zhao, Y. Hu, L. Zhang, G. Fan, J. Xu, X. Gu, Z. Cheng, T. Yu, J. Xia, Y. Wei, W. Wu, X. Xie, W. Yin, H. Li, M. Liu, Y. Xiao, H. Gao, L. Guo, J. Xie, G. Wang, R. Jiang, Z. Gao, Q. Jin, J. Wang and B. Cao (2020). "Clinical features of patients infected with 2019 novel coronavirus in Wuhan, China." Lancet **395**(10223): 497-506.

Hwang, I.K., W.J. Shih and J.S. De Cani (1990). "Group sequential designs using a family of type I error probability spending functions." Stat Med **9**(12): 1439-1445.

Jaiswal, S.R. and S. Chakrabarti (2020). "CTLA4Ig Limits Both Incidence and Severity of Early Cytokine Release Syndrome following Haploidentical Peripheral Blood Stem Cell Transplantation." Biol Blood Marrow Transplant **26**(4): e86-e87.

Kang, E.H., Y. Jin, R.J. Desai, J. Liu, J.A. Sparks and S.C. Kim (2019). "Risk of exacerbation of pulmonary comorbidities in patients with rheumatoid arthritis after initiation of abatacept versus TNF inhibitors: A cohort study." Semin Arthritis Rheum **50**(3): 401-408.

Komatsu, M., D. Kobayashi, K. Saito, D. Furuya, A. Yagihashi, H. Araake, N. Tsuji, S. Sakamaki, Y. Niitsu and N. Watanabe (2001). "Tumor necrosis factor-alpha in serum of patients with inflammatory bowel disease as measured by a highly sensitive immuno-PCR." Clin Chem **47**(7): 1297-1301.

Koura, D. T., J.T. Horan, A.A. Langston, M. Qayed, A. Mehta, H.J. Khoury, R.D. Harvey, Y. Suessmuth, C. Couture, J. Carr, A. Grizzle, H.R. Johnson, J.A. Cheeseman, J.A. Conger, J. Robertson, L. Stempora, B.E. Johnson, A. Garrett, A.D. Kirk, C.P. Larsen, E.K. Waller and L.S. Kean (2013). "In vivo T cell costimulation blockade with abatacept for acute graft-versus-host disease prevention: a first-in-disease trial." Biol Blood Marrow Transplant **19**(11): 1638-1649.

Law, H.K., C.Y. Cheung, S.F. Sia, Y.O. Chan, J.S. Peiris and Y.L. Lau (2009). "Toll-like receptors, chemokine receptors and death receptor ligands responses in SARS coronavirus infected human monocyte derived dendritic cells." BMC Immunol **10**: 35.

Linsley, P.S. and S.G. Nadler (2009). "The clinical utility of inhibiting CD28-mediated costimulation." Immunol Rev **229**(1): 307-321.

Lo, B., K. Zhang, W. Lu, L. Zheng, Q. Zhang, C. Kanellopoulou, Y. Zhang, Z. Liu, J. M. Fritz, R. Marsh, A. Husami, D. Kissell, S. Nortman, V. Chaturvedi, H. Haines, L. R. Young, J. Mo, A.H. Filipovich, J.J. Bleesing, P. Mustillo, M. Stephens, C.M. Rueda, C.A. Chougnet, K. Hoebe, J. McElwee, J. D. Hughes, E. Karakoc-Aydiner, H. F. Matthews, S. Price, H.C. Su, V.K. Rao, M.J. Lenardo and M.B. Jordan (2015). "AUTOIMMUNE DISEASE. Patients with LRBA deficiency show CTLA4 loss and immune dysregulation responsive to abatacept therapy." Science **349**(6246): 436-440.

Marx, D., B. Moulin, S. Fafi-Kremer, I. Benotmane, G. Gautier, P. Perrin and S. Caillard (2020). "First case of COVID-19 in a kidney transplant recipient treated with belatacept." Am J Transplant **20**(7): 1944-1946.

Monti, S., S. Balduzzi, P. Delvino, E. Bellis, V.S. Quadrelli and C. Montecucco (2020). "Clinical course of COVID-19 in a series of patients with chronic arthritis treated with immunosuppressive targeted therapies." Ann Rheum Dis **79**(5): 667-668.

Monti, S., C. Klersy, R. Gorla, P. Sarzi-Puttini, F. Atzeni, R. Pellerito, E. Fusaro, G. Paolazzi, P.A. Rocchetta, E.G. Favalli, A. Marchesoni and R. Caporali (2017). "Factors influencing the choice of first- and second-line biologic therapy for the treatment of rheumatoid arthritis: real-life data from the Italian LORHEN Registry." Clin Rheumatol **36**(4): 753-761.

Mossanen, J.C., O. Krenkel, C. Ergen, O. Govaere, A. Liepelt, T. Puengel, F. Heymann, S. Kalthoff, E. Lefebvre, D. Eulberg, T. Luedde, G. Marx, C.P. Strassburg, T. Roskams, C. Trautwein and F. Tacke (2016). "Chemokine (C-C motif) receptor 2-positive monocytes aggravate the early phase of acetaminophen-induced acute liver injury." Hepatology **64**(5): 1667-1682.

Mulangu, S., L.E. Dodd, R.T. Davey, Jr., O. Tshiani Mbaya, M. Proschan, D. Mukadi, M. Lusakibanza Manzo, D. Nzolo, A. Tshomba Oloma, A. Ibanda, R. Ali, S. Coulibaly, A.C. Levine, R. Grais, J. Diaz, H.C. Lane, J.J. Muyembe-Tamfum, P.W. Group, B. Sivahera, M. Camara, R. Kojan, R. Walker, B. Dighero-Kemp, H. Cao, P. Mukumbayi, P. Mbala-Kingebeni, S. Ahuka, S. Albert, T. Bonnett, I. Crozier, M. Duvenhage, C. Proffitt, M. Teitelbaum, T. Moench, J. Aboulhab, K. Barrett, K. Cahill, K. Cone, R. Eckes, L. Hensley, B. Herpin, E. Higgs, J. Ledgerwood, J. Pierson, M. Smolskis, Y. Sow, J. Tierney, S. Sivapalasingam, W. Holman, N. Gettinger, D. Vallee, J. Nordwall and P.C.S. Team (2019). "A Randomized, Controlled Trial of Ebola Virus Disease Therapeutics." N Engl J Med **381**(24): 2293-2303.

Ng, M.W., G. Zhou, W. P. Chong, L.W. Lee, H.K. Law, H. Zhang, W.H. Wong, S.F. Fok, Y. Zhai, R.W. Yung, E. Y. Chow, K.L. Au, E.Y. Chan, W. Lim, J.S. Peiris, F. He and Y.L. Lau (2007). "The association of RANTES polymorphism with severe acute respiratory syndrome in Hong Kong and Beijing Chinese." BMC Infect Dis **7**: 50.

Prompetchara, E., C. Ketloy and T. Palaga (2020). "Immune responses in COVID-19 and potential vaccines: Lessons learned from SARS and MERS epidemic." Asian Pac J Allergy Immunol **38**(1): 1-9.

Puengel, T., O. Krenkel, M. Kohlhepp, E. Lefebvre, T. Luedde, C. Trautwein and F. Tacke (2017). "Differential impact of the dual CCR2/CCR5 inhibitor cenicriviroc on migration of monocyte and lymphocyte subsets in acute liver injury." PLoS One **12**(9): e0184694.

Record, J.L., T. Beukelman and R.Q. Cron (2011). "Combination therapy of abatacept and anakinra in children with refractory systemic juvenile idiopathic arthritis: a retrospective case series." J Rheumatol **38**(1): 180-181.

RECOVERY Collaborative Group (2020). "Dexamethasone in Hospitalized Patients with Covid-19 – Preliminary Report." N Engl J Med NEJMoa2021436.

Ruan, Q., K. Yang, W. Wang, L. Jiang and J. Song (2020). "Clinical predictors of mortality due to COVID-19 based on an analysis of data of 150 patients from Wuhan, China." Intensive Care Med **46**(5): 846-848.

Salem, J.E., Y. Allenbach, A. Vozy, N. Brechot, D.B. Johnson, J.J. Moslehi and M. Kerneis (2019). "Abatacept for Severe Immune Checkpoint Inhibitor-Associated Myocarditis." N Engl J Med **380**(24): 2377-2379.

Teijaro, J.R., M. N. Njau, D. Verhoeven, S. Chandran, S.G. Nadler, J. Hasday and D.L. Farber (2009). "Costimulation modulation uncouples protection from immunopathology in memory T cell responses to influenza virus." J Immunol **182**(11): 6834-6843.

Tesch, V.K., H. Abolhassani, B. Shadur, J. Zobel, Y. Mareika, S. Sharapova, E. Karakoc-Aydiner, J.G. Rivière, M. Garcia-Prat, N. Moes, F. Haerynck, L.I. Gonzales-Granado, J.L. Santos Pérez, A. Mukhina, A. Shcherbina, A. Aghamohammadi, L. Hammarström, F. Dogu, S. Haskologlu, A.I. İkincioğulları, S. Köstel Bal, S. Baris, S.S. Kilic, N.E. Karaca, N. Kutukculer, H. Girschick, A. Kolios, S. Keles, V. Uygun, P. Stepensky, A. Worth, J.M. van Montfrans, A.M.J. Peters, I. Meyts, M. Adeli, A. Marzollo, N. Padem, A.M. Khojah, Z. Chavoshzadeh, M. Avbelj Stefanija, S. Bakhtiar, B. Florkin, M. Meeths, L. Gamez, B. Grimbacher, M.R.J. Seppänen, A. Lankester, A.R.

Gennery and M.G. Seidel (2020). "Long-term outcome of LRBA deficiency in 76 patients after various treatment modalities as evaluated by the immune deficiency and dysregulation activity (IDDA) score." J Allergy Clin Immunol **145**(5): 1452-1463.

Tu, X., W.P. Chong, Y. Zhai, H. Zhang, F. Zhang, S. Wang, W. Liu, M. Wei, N. H. Siu, H. Yang, W. Yang, W. Cao, Y. L. Lau, F. He and G. Zhou (2015). "Functional polymorphisms of the CCL2 and MBL genes cumulatively increase susceptibility to severe acute respiratory syndrome coronavirus infection." J Infect **71**(1): 101-109.

Van Gool, S.W., Y. Zhang, A. Kasran, M. de Boer and J.L. Ceuppens (1996). "T helper-independent activation of human CD8+ cells: the role of CD28 costimulation." Scand J Immunol **44**(1): 21-29.

Watkins, B., M. Qayed, B. Bratrude, K. Betz, M. Brown, J. Rhodes, S. Sinclair, Y. Suessmuth, A. Yu, K. Hebert, M. C. Pasquini, S.W. Choi, J. Davis, C. Duncan, R. Giller, M. Grimley, A. C. Harris, D.A. Jacobsohn, N. Lalefar, M. Norkin, M.A. Pulsipher, S. Shenoy, B. Blazar, A. Langston, J.T. Horan and L. Kean (2017). "T Cell Costimulation Blockade with Abatacept Nearly Eliminates Early Severe Acute Graft Versus Host Disease after HLA-Mismatched (7/8 HLA Matched) Unrelated Donor Transplant, with a Favorable Impact on Disease-Free and Overall Survival." Blood **130**(Supplement 1): 212-212.

Watt, K., J.S. Li, D.K. Benjamin Jr. and M. Cohen-Wolkowiez (2011). "Pediatric cardiovascular drug dosing in critically ill children and extracorporeal membrane oxygenation." J Cardiovasc Pharmacol **58**(2): 126-132.

Wildschut, E.D., M.J. Ahsman, K. Allegaert, R.A. Mathot, and D. Tibboel (2010). "Determinants of drug absorption in different ECMO circuits." Intensive Care Med **36**(12): 2109-2116.

Weisman, M.H., P. Durez, D. Hallegua, R. Aranda, J. C. Becker, I. Nuamah, G. Vratsanos, Y. Zhou and L.W. Moreland (2006). "Reduction of inflammatory biomarker response by abatacept in treatment of rheumatoid arthritis." J Rheumatol **33**(11): 2162-2166.

Wenink, M.H., K. C. Santegoets, A.M. Platt, W.B. van den Berg, P.L. van Riel, P. Garside, T.R. Radstake and I.B. McInnes (2012). "Abatacept modulates proinflammatory macrophage responses upon cytokine-activated T cell and Toll-like receptor ligand stimulation." Ann Rheum Dis **71**(1): 80-83.

Whitfield, S.J. C., C. Taylor, J.E. Risdall, G.D. Griffiths, J.T.A. Jones, E.D. Williamson, S. Rijpkema, L. Saraiva, S. Vessillier, A.C. Green and A.J. Carter (2017). "Interference of the T Cell and Antigen-Presenting Cell Costimulatory Pathway Using CTLA4-Ig (Abatacept) Prevents Staphylococcal Enterotoxin B Pathology." J Immunol **198**(10): 3989-3998.

Whitmire, J.K. and R. Ahmed (2000). "Costimulation in antiviral immunity: differential requirements for CD4(+) and CD8(+) T cell responses." Curr Opin Immunol **12**(4): 448-455.

Williams, A.E., R.J. José, P.F. Mercer, D. Brealey, D. Parekh, D.R. Thickett, C. O'Kane, D.F. McAuley and R.C. Chambers (2017). "Evidence for chemokine synergy during neutrophil migration in ARDS." Thorax **72**(1): 66-73.

Wu, F., S. Zhao, B. Yu, Y.M. Chen, W. Wang, Z. G. Song, Y. Hu, Z.W. Tao, J.H. Tian, Y.Y. Pei, M.L. Yuan, Y.L. Zhang, F.H. Dai, Y. Liu, Q.M. Wang, J.J. Zheng, L. Xu, E.C. Holmes and Y.Z. Zhang (2020). "A new coronavirus associated with human respiratory disease in China." Nature **579**(7798): 265-269.

Zemans, R.L. and M.A. Matthay (2017). "What drives neutrophils to the alveoli in ARDS?" Thorax **72**(1): 1-3.

Zhou, F., T. Yu, R. Du, G. Fan, Y. Liu, Z. Liu, J. Xiang, Y. Wang, B. Song, X. Gu, L. Guan, Y. Wei, H. Li, X. Wu, J. Xu, S. Tu, Y. Zhang, H. Chen and B. Cao (2020). "Clinical course and risk factors for mortality of adult inpatients with COVID-19 in Wuhan, China: a retrospective cohort study." Lancet **395**(10229): 1054-1062.

Randomized Master Protocol for Immune Modulators for Treating COVID-19

Short Title: ACTIV-1 IM

Protocol ID: ACTIV-1 IM

Version Number: 2.0

02 December 2020

Sponsor:

Daniel K. Benjamin, MD, MPH, PhD
Kiser-Arena Distinguished Professor, Duke University Pediatrics
Duke Clinical Research Institute
300 W. Morgan Street, Suite 800
Durham Center
Durham, NC 27701
Tel: 919-668-7081
Fax: 919-681-9457
E-mail: danny.benjamin@duke.edu

Supported by:

The National Center for Advancing Translational Sciences (NCATS) with funding from the Biomedical Advanced Research and Development Authority (BARDA) of the U.S. Department of Health and Human Services Office of the Assistant Secretary for Preparedness and Response

Protocol Chair:

William G. Powderly, MD
J. William Campbell Professor of Medicine
Larry J. Shapiro Director, Institute for Public Health
Director, Institute of Clinical and Translational Sciences
Co-Director, Division of Infectious Diseases
Washington University in St. Louis

STATEMENT OF COMPLIANCE

Each institution engaged in this research will hold a current Federalwide Assurance (FWA) issued by the U.S. Office of Human Research Protection (OHRP) for federally funded research. The Institutional Review Board (IRB)/Independent or Institutional Ethics Committee (IEC) must be registered with OHRP as applicable to the research.

The study will be carried out in accordance with the following as applicable:

- All National and Local Regulations and Guidance applicable at each site
- The International Council for Harmonization of Technical Requirements for Registration of Pharmaceuticals for Human Use (ICH) E6(R2) Good Clinical Practice, and the Belmont Report: Ethical Principles and Guidelines for the Protection of Human Subjects of Research, Report of the National Commission for the Protection of Human Subjects of Biomedical and Behavioral Research
- United States (US) Code of Federal Regulations (CFR) 45 CFR Part 46: Protection of Human Subjects
- US Food and Drug Administration (FDA) Regulations: 21 CFR Part 50 (Protection of Human Subjects), 21 CFR Part 54 (Financial Disclosure by Clinical Investigators), 21 CFR Part 56 (IRBs), 21 CFR Part 11, and 21 CFR Part 312 (Investigational New Drug Application), and/or 21 CFR 812 (Investigational Device Exemptions)

The signature below provides the necessary assurance that this study will be conducted according to all stipulations of the protocol including statements regarding confidentiality, and according to local legal and regulatory requirements, US federal regulations, and ICH E6(R2) GCP guidelines.

Site Investigator Signature:

Signed: ______________________________ Date: ______________
Name and Title

Table of Contents

STATEMENT OF COMPLIANCE 2
1. PROTOCOL SUMMARY 10
1.1. ACTIV-1 IM – Synopsis 10
1.1.1. *Study Overview* 10
1.1.2. *Enrollment Period* 10
1.1.3. *General* 10
1.1.4. *Study Population* 11
1.1.5. *Inclusion Criteria* 11
1.1.6. *Exclusion Criteria at Screening* 11
1.1.7. *Study Intervention* 12
1.1.8. *Schedule of Assessments* 14
2. INTRODUCTION 17
2.1. Background 17
2.1.1. *ACTIV-1 IM – Immune Modulators & Remdesivir Multi-arm Trial* 17
2.2. Risk/Benefit Assessment 17
2.2.1. *Known Potential Risks of Study Participation* 17
2.2.1.1. *Risks to Privacy* 17
2.2.2. *SoC - Remdesivir* 18
2.2.2.1. *Potential Risks of Remdesivir* 18
2.2.2.2. *Potential Benefits of Remdesivir* 18
2.2.2.3. *Assessment of Potential Risks and Benefits of Remdesivir* 19
2.2.3. *Therapeutic Agents* 19
3. OBJECTIVES AND ENDPOINTS 19
4. STUDY DESIGN 21
4.1. Overall Design 21
4.2. Scientific Rationale for Study Design 21
4.3. Justification for Dose 22
4.3.1. *Justification for Dose of Remdesivir* 22
4.3.2. *Justification for Dose of Investigational Agents* 22
5. STUDY POPULATION 22
5.1. Inclusion Criteria 22
5.2. Exclusion Criteria at Screening 23
5.3. Exclusion of Specific Populations 24
5.4. Inclusion of Vulnerable Subjects 24
5.5. Lifestyle Considerations 24
5.6. Screen Failures 24
5.7. Strategies for Recruitment and Retention 25
5.7.1. *Recruitment* 25
5.7.2. *Retention* 25
5.8. Compensation Plan for Subjects 25
5.9. Costs 25
6. STUDY PRODUCT 25
6.1. Study Product(s) and Administration 25

6.1.1. *Study Product Description* 26
6.1.2. *Dosing and Administration* 26
6.1.3. *Dose Escalation* 26
6.1.4. *Dose Modifications* 26
6.1.5. *Overdosage* 27
6.1.6. *Preparation/Handling/Storage/Accountability* 27
6.1.6.1. *Acquisition and Accountability* 27
6.1.6.2. *Formulation, Appearance, Packaging, and Labeling* 27
6.1.6.3. *Product Storage and Stability* 28
6.1.6.4. *Preparation* 28
6.2. Measures to Minimize Bias: Randomization and Blinding 28
6.3. Study Intervention Compliance 29
6.4. Concomitant Therapy 29
6.4.1. *Permitted Concomitant Therapy and Procedures* 29
6.4.2. *Prohibited Concomitant Therapy* 29
6.4.3. *Rescue Medicine* 30
6.4.4. *Non-Research Standard of Care* 30
7. STUDY INTERVENTION DISCONTINUATION AND SUBJECT DISCONTINUATION/WITHDRAWAL 30
7.1. Halting Criteria and Discontinuation of Study Intervention 30
7.1.1. *Individual Study Product Halting* 30
7.1.2. *Study Halting* 31
7.2. Withdrawal from the Study 31
7.3. Lost to Follow-Up 31
7.4. Readmission 31
8. STUDY ASSESSMENTS AND PROCEDURES 31
8.1. Screening and Efficacy Assessments 31
8.1.1. *Screening Procedures* 31
8.1.2. *Efficacy Assessments* 33
8.1.3. *Exploratory Assessments* 35
8.2. Safety and Other Assessments 36
8.2.1. *Procedures to be Followed in the Event of Abnormal Laboratory Test Values or Abnormal Clinical Findings* 38
8.2.2. *Unscheduled Visits* 38
8.3. Adverse Events and Serious Adverse Events 38
8.3.1. *Definition of Adverse Event (AE)* 38
8.3.2. *Definition of Serious Adverse Event (SAE)* 39
8.3.3. *Suspected Unexpected Serious Adverse Reactions (SUSAR)* 39
8.3.4. *Classification of an Adverse Event* 40
8.3.5. *Time Period and Frequency for Event Assessment and Follow-Up* 40
8.3.6. *Serious Adverse Event Reporting* 41
8.3.7. *Reporting of Pregnancy* 41
8.4. Unanticipated Problems 41
8.4.1. *Definition of Unanticipated Problems* 41

8.4.2. *Unanticipated Problem Reporting* 41
9. STATISTICAL CONSIDERATIONS 42
9.1. Statistical Hypotheses 43
9.2. Sample Size Determination 43
9.3. Populations for Analyses 44
9.4. Statistical Analyses 45
9.4.1. *General Approach* 45
9.4.2. *Analysis of the Primary Efficacy Endpoint* 45
9.4.3. *Analysis of the Secondary Endpoint(s)* 45
9.4.4. *Safety Analyses* 46
9.4.5. *Baseline Descriptive Statistics* 46
9.4.6. *Planned Early and Interim Analyses* 46
9.4.7. *Sub-Group Analyses* 47
9.4.8. *Exploratory Analyses* 48
10. SUPPORTING DOCUMENTATION AND OPERATIONAL CONSIDERATIONS – ALL STAGES 48
APPENDIX 1 – SUB-STUDY 1 – INFLIXIMAB (REMICADE) 49
1. SUB-STUDY SUMMARY 49
1.1. Sub-study overview 49
1.2. Enrollment Period 49
1.3. General 49
1.4. Study Population 49
1.5. Inclusion Criteria 49
1.6. Agent Specific Exclusion Criteria at Randomization 49
1.7. Study Intervention 49
1.8. Schedule of Assessments 50
2. JUSTIFICATION FOR SELECTION 50
3. INTRODUCTION 51
3.1. Risk/Benefit Assessment 51
3.1.1. *Remicade* 51
3.1.1.1. *Potential Risks of Remicade* 51
3.1.1.2. *Potential Benefits of Remicade* 51
3.1.1.3. *Assessment of Potential Risks and Benefits of Remicade* 51
4. OBJECTIVES AND ENDPOINTS 52
5. SUB-STUDY DESIGN 52
5.1. Justification for Dose 52
5.1.1. *Justification for Dose of Remicade* 52
6. STUDY POPULATION 52
6.1. Inclusion Criteria 53
6.2. Sub-Study Specific Exclusion Criteria 53
6.3. Exclusion of Specific Populations 53
7. STUDY PRODUCT 53
7.1. Study Product(s) and Administration 53
7.1.1. *Study Product Description* 53

7.1.2. *Dosing and Administration* 53
7.1.3. *Dose Escalation* 54
7.1.4. *Dose Modifications* 54
7.1.5. *Overdosage* 54
7.1.6. *Preparation/Handling/Storage/Accountability* 54
7.1.6.1. *Acquisition and Accountability* 54
7.1.6.2. *Formulation, Appearance, Packaging, and Labeling* 55
7.1.6.3. *Product Storage and Stability* 55
7.1.6.4. *Preparation and Administration* 55
7.2. Measures to Minimize Bias: Randomization and Blinding 55
7.3. Study Intervention Compliance 55
7.4. Concomitant Therapy 55
7.4.1. *Permitted Concomitant Therapy and Procedures* 55
7.4.2. *Prohibited Concomitant Therapy* 56
7.4.3. *Rescue Medicine* 56
7.4.4. *Non-Research Standard of Care* 56
8. STUDY INTERVENTION DISCONTINUATION AND SUBJECT DISCONTINUATION/WITHDRAWAL 56
8.1. Halting Criteria and Discontinuation of Study Intervention 56
8.1.1. *Individual Study Product Halting* 56
8.1.2. *Study Halting* 56
8.2. Withdrawal from the Study 56
8.3. Lost to Follow-Up 56
8.4. Readmission 56
9. STUDY ASSESSMENTS AND PROCEDURES 57
10. STATISTICAL CONSIDERATIONS 57
APPENDIX 2 – SUB-STUDY 2 – ABATACEPT (ORENCIA) 58
1. SUB-STUDY SUMMARY 58
1.1. Sub-study overview 58
1.2. Enrollment Period 58
1.3. General 58
1.4. Study Population 58
1.5. Inclusion Criteria 58
1.6. Agent Specific Exclusion Criteria 58
1.7. Study Intervention 58
1.8. Schedule of Assessments 59
2. JUSTIFICATION FOR SELECTION 59
3. INTRODUCTION 61
3.1. Risk/Benefit Assessment 61
3.1.1. *Abatacept* 61
3.1.1.1. *Potential Risks of Abatacept* 61
3.1.1.2. *Potential Benefits of Abatacept* 63
3.1.1.3. *Assessment of Potential Risks and Benefits of Abatacept* 63
4. OBJECTIVES AND ENDPOINTS 63

5. STUDY DESIGN 64
5.1. Justification for Dose 64
5.1.1. *Justification for Dose of Abatacept* 64
6. STUDY POPULATION 64
6.1. Inclusion Criteria 64
6.2. Sub-Study Specific Exclusion Criteria 64
6.3. Exclusion of Specific Populations 64
7. STUDY PRODUCT 65
7.1. Study Product(s) and Administration 65
7.1.1. *Study Product Description* 65
7.1.2. *Dosing and Administration* 65
7.1.3. *Dose Escalation* 65
7.1.4. *Dose Modifications* 65
7.1.5. *Overdosage* 66
7.1.6. *Preparation/Handling/Storage/Accountability* 66
7.1.6.1. *Acquisition and Accountability* 66
7.1.6.2. *Formulation, Appearance, Packaging, and Labeling* 67
7.1.6.3. *Product Storage and Stability* 67
7.1.6.4. *Preparation* 67
7.2. Measures to Minimize Bias: Randomization and Blinding 68
7.3. Study Intervention Compliance 68
7.4. Concomitant Therapy 68
7.4.1. *Permitted Concomitant Therapy and Procedures* 68
7.4.2. *Prohibited Concomitant Therapy* 68
7.4.3. *Rescue Medicine* 68
7.4.4. *Non-Research Standard of Care* 68
8. STUDY INTERVENTION DISCONTINUATION AND SUBJECT DISCONTINUATION/WITHDRAWAL 68
8.1. Halting Criteria and Discontinuation of Study Intervention 68
8.1.1. *Individual Study Product Halting* 68
8.1.2. *Study Halting* 69
8.2. Withdrawal from the Study 69
8.3. Lost to Follow-Up 69
8.4. Readmission 69
9. STUDY ASSESSMENTS AND PROCEDURES 69
10. STATISTICAL CONSIDERATIONS 69
APPENDIX 3 – SUB-STUDY 3 – CENICRIVIROC (CVC) 70
1. SUB-STUDY SUMMARY 70
1.1. Sub-study overview 70
1.2. Enrollment Period 70
1.3. General 70
1.4. Study Population 70
1.5. Inclusion Criteria 70
1.6. Agent Specific Exclusion Criteria 70

1.7. Study Intervention 70
1.8. Schedule of Assessments 71
2. JUSTIFICATION FOR SELECTION 71
3. INTRODUCTION 73
3.1. Risk/Benefit Assessment 73
3.1.1. *Cenicriviroc* 73
3.1.1.1. *Potential Risks of Cenicriviroc* 73
3.1.1.2. *Potential Benefits of Cenicriviroc* 74
3.1.1.3. *Assessment of Potential Risks and Benefits of Cenicriviroc* 74
4. OBJECTIVES AND ENDPOINTS 75
5. STUDY DESIGN 75
5.1. Justification for Dose 75
5.1.1. *Justification for Dose of Cenicriviroc* 75
6. STUDY POPULATION 78
6.1. Inclusion Criteria 78
6.2. Sub-Study Specific Exclusion Criteria 78
6.3. Exclusion of Specific Populations 78
7. STUDY PRODUCT 78
7.1. Study Product(s) and Administration 78
7.1.1. *Study Product Description* 79
7.1.2. *Dosing and Administration* 79
7.1.3. *Dose Escalation* 80
7.1.4. *Dose Modifications* 80
7.1.4.1. *Dose Modifications for Adverse Events* 80
7.1.5. *Overdosage* 81
7.1.6. *Preparation/Handling/Storage/Accountability* 82
7.1.6.1. *Acquisition and Accountability* 82
7.1.6.2. *Formulation, Appearance, Packaging, and Labeling* 82
7.1.6.3. *Product Storage and Stability* 82
7.1.6.4. *Preparation* 82
7.2. Measures to Minimize Bias: Randomization and Blinding 83
7.3. Study Intervention Compliance 83
7.4. Concomitant Therapy 83
7.4.1. *Permitted Concomitant Therapy and Procedures* 83
7.4.2. *Prohibited Concomitant Therapy* 83
7.4.3. *Rescue Medicine* 85
7.4.4. *Non-Research Standard of Care* 85
8. STUDY INTERVENTION DISCONTINUATION AND SUBJECT DISCONTINUATION/WITHDRAWAL 85
8.1. Halting Criteria and Discontinuation of Study Intervention 85
8.1.1. *Individual Study Product Halting* 85
8.1.2. *Study Halting* 85
8.2. Withdrawal from the Study 85
8.3. Lost to Follow-Up 85

8.4. Readmission....................86
9. STUDY ASSESSMENTS AND PROCEDURES....................86
10. STATISTICAL CONSIDERATIONS....................86
APPENDIX 4 – ACTIV-1 IM PROTOCOL TEAM ROSTER....................87
11. REFERENCES....................91

ACTIV-1 IM:
RANDOMIZED MASTER PROTOCOL FOR IMMUNE MODULATORS FOR TREATING COVID-19

1. PROTOCOL SUMMARY

1.1. ACTIV-1 IM – Synopsis

1.1.1. *Study Overview*

ACTIV-1 IM is a master protocol designed to evaluate multiple investigational agents for the treatment of moderately or severely ill patients infected with severe acute respiratory syndrome coronavirus 2 (SARS-CoV-2). The research objectives are to evaluate each agent with respect to speed of recovery, mortality, illness severity, and hospital resource utilization. Each agent will be evaluated as add-on therapy to the standard of care (SoC) in accordance with national or local guidelines, which may include remdesivir. The SoC may change during the course of the study based on other research findings. Comparisons of the agents among themselves is not a research objective.

The study population corresponds to moderately and severely ill patients infected with the coronavirus disease 2019 (COVID-19) virus. Recruitment will target patients already hospitalized for treatment of COVID-19 infection as well as patients being treated for COVID-19 infection in Emergency Departments while waiting to be admitted to the hospital. Patients both in and out of the ICU are included in the study population.

Enrollment began when 3 agents were selected for initial evaluation and available for testing. Up to 5 agents may be evaluated in ACTIV-1 IM. Appendices to this protocol describe each agent and any sub-study-specific procedures and considerations for that agent.

The study period is 29 days, with assessments on each day of the hospital stay. Discharged patients will be followed with periodic follow-up assessments through Day 29. There will also be a safety and clinical status assessment at 60 days. Treatment periods may vary by agent.

The trial is adaptive in that interim analyses are planned to assess the futility of each agent, with the goal of discontinuing those with lower probabilities of success to more effectively utilize trial resources for the remaining agents. Additionally, interim analyses are planned for early stopping for efficacy. Alpha spending functions are used to appropriately control the probability of making an erroneous conclusion at the interim and final analyses.

Safety monitoring will be performed throughout the trial, and formal stopping rules for each agent will be adopted. The Data and Safety Monitoring Board (DSMB) selected for ACTIV-1 IM has oversight responsibility for the study.

1.1.2. *Enrollment Period*

Enrollment began in October 2020. It is anticipated that enrollment will be completed in 4-6 months.

1.1.3. *General*

ACTIV-1 IM is a master protocol designed to evaluate immune modulators for the treatment of moderately or severely ill hospitalized patients infected with COVID-19. Trial participants will be assessed daily while hospitalized. If the participants are discharged from the hospital prior to Day 29, they will have follow-up study visits at Days 8, 11, 15, 22, and 29. For discharged participants, it is preferred that the Day 8, 11, 15, and 29 visits are in person to obtain safety laboratory tests and blood (serum/plasma) samples for secondary research as well as clinical outcome data. However, infection control or other restrictions may limit the ability of the participant to return to the clinic. In this case, these visits may be conducted by phone, and only clinical data will be obtained. The Day 22 visit does not have laboratory tests or collection of samples and is conducted by phone. The Day 60 assessment will be conducted by phone.

The effectiveness of each therapeutic agent as add-on therapy to SoC which may include remdesivir (provided) will be evaluated based on the primary endpoint of time to recovery by Day 29. The sample size requirements are based on the ability to detect a moderate improvement in time to recovery (3-4 fewer days) for each agent (see Section 9.2). A total of 788 recoveries are required for each comparison to provide approximately 85% power to detect a recovery rate ratio of 1.25. Assuming 73% of participants achieve recovery in 28 days, consistent with the ACTT-1 results, the total sample size to evaluate 1, 2, and 3 agents in ACTIV-1 IM is approximately 1080, 1620, and 2160, respectively. Because each agent is being compared to SoC with no between-agent comparisons, no multiplicity adjustments for multiple agents are planned.

1.1.4. *Study Population*

Hospitalized adults (≥18 years old) with COVID-19, including patients both in and out of the ICU. Patients seeking care for COVID-19 in an Emergency Department (ED) and waiting to be admitted to the hospital are included.

1.1.5. *Inclusion Criteria*

1. Admitted to a hospital or awaiting admission in the ED with symptoms suggestive of COVID-19.
2. Subject (or legally authorized representative) provides informed consent prior to initiation of any study procedures.
3. Subject (or legally authorized representative) understands and agrees to comply with planned study procedures.
4. Male or non-pregnant female adults ≥18 years of age at time of enrollment.
5. Has laboratory-confirmed (within 14 days prior to enrollment) SARS-CoV-2 infection as determined by PCR or other commercial or public health assay in any specimen.
6. Ongoing illness of any duration, and at least one of the following:
    - Radiographic infiltrates by imaging (chest x-ray, CT scan, etc.), OR
    - Blood oxygen saturation (SpO_2) ≤ 94% on room air, OR
    - Requiring supplemental oxygen, OR
    - Requiring mechanical ventilation or ECMO.
7. Women of childbearing potential must agree to either abstinence or use of at least one primary form of contraception not including hormonal contraception from the time of screening through Day 60.
8. Agrees not to participate in another interventional trial for the treatment of COVID-19 through Day 60.
   Exception 1: Participant may co-enroll in ACTIV-4 (ACTIV-4A and ACTIV-4C).
   Exception 2: Participants in ACTIV-2 who have been hospitalized may be enrolled in ACTIV-1 as long as ACTIV-2 study therapy has been discontinued. They will remain in ACTIV-2 follow-up.
   Exception 3: If participant is already participating in a COVID-19 vaccine trial but develops COVID-19 disease that requires hospitalization, participant is eligible for this study, assuming all other inclusion/exclusion criteria are met.

1.1.6. *Exclusion Criteria at Screening*

1. ALT or AST >10 times the upper limit of normal.
2. Estimated glomerular filtration rate (eGFR) <30 mL/min (including patients receiving hemodialysis or hemofiltration).
   Exception: Participants with an eGFR <30 mL/min may enroll as long as their renal insufficiency has been stable without renal replacement therapy for ≥1 month and they are not current candidates for renal replacement therapy. **These participants will not receive remdesivir.**
3. Neutropenia (absolute neutrophil count <1000 cells/µL) (<1.0 x 10^3/µL or <1.0 GI/L).
4. Lymphopenia (absolute lymphocyte count <200 cells/µL) (<0.20 x 10^3/µL or <0.20 GI/L)
5. Pregnancy or breast feeding.
6. Anticipated discharge from the hospital or transfer to another hospital which is not a study site within 72 hours.
7. Known allergy to any study medication.
8. Received targeted immune-modulator treatments (such as anti-interleukin-1 [IL-1], anti-IL-6 [tocilizumab or sarilumab], anti-IL-17, T-cell or B-cell targeted therapies [e.g., rituximab], JAK inhibitors [including

baricitinib], TNF inhibitors, or interferon) within 4 weeks or 5 half-lives prior to screening, whichever is longer. Steroid dependency, defined as need for prednisone at a dose >10 mg (or equivalent) for >1 month within 2 weeks of screening, is exclusionary.
Exception 1: Dexamethasone is permitted for the treatment of COVID-19 in patients who are already mechanically ventilated and in patients who require supplemental oxygen at screening, but who are not mechanically ventilated in accordance with national guidelines.
Exception 2: Infusion of convalescent plasma given for treatment of COVID-19 while on-study is also allowed.
Exception 3: Monoclonal antibody therapy given for COVID-19 treatment at any time prior to enrollment is also allowed.

9. Known or suspected history of untreated tuberculosis (TB). TB diagnosis may be suspected based on medical history and concomitant therapies that would suggest TB infection. Participants are also excluded if they have known, latent TB treated for less than 4 weeks with appropriate anti-tuberculosis therapy per local guidelines (by history only, no screening required).
10. Known or suspected serious, active bacterial, fungal, or viral infection (excepting SARS-CoV-2 and including, but not limited to, active HBV, HCV, or HIV with the latter defined as a CD4 count <200 or an unsuppressed HIV viral load), or other infection (besides COVID-19) that in the opinion of the investigator could constitute a risk when taking investigational product.
Note: Broad-spectrum empiric antibiotic usage does not exclude participation.
11. Have received any live vaccine (or live attenuated) within 3 months before screening, or intend to receive a live vaccine (or live attenuated) during the study.
Exception: Use of prior non-live (inactivated) vaccinations is allowed for all participants, including any vaccine for COVID-19.
12. Severe hepatic impairment (defined as liver cirrhosis Child stage C).
13. Known severe heart failure (New York Heart Association [NYHA] III-IV) or new-onset left-systolic or global cardiac dysfunction in the setting of COVID-19.
Exception: Right-sided heart dysfunction or pulmonary hypertension thought related to COVID-19 is permitted.
14. In the Investigator’s judgment, the patient has any advanced organ dysfunction that would not make participation appropriate.

1.1.7. *Study Intervention*

Immune-modulating agents will be evaluated as an add-on therapy to SoC. Participants will be randomly assigned to receive either SoC (which may include remdesivir) plus one of the test agents or SoC (which may include remdesivir) plus placebo. Randomization will proceed in two stages via a web-based central randomization system. At the first stage (open label), each participant will be assigned to one of the sub-studies with equal probability. At the second stage (blinded), each participant will be assigned to either the test agent or its matching placebo in an n:1 ratio, where n = the number of agents currently active in the master protocol and for which the patient is eligible to receive. With three agents and SoC, for example, this procedure results in a randomization ratio of 1:1:1:1 if the participant is eligible to receive all three agents (see Section 6.2).

Inclusion of a placebo for each agent enables masking of study participants and clinical personnel to treatment assignment at the second stage. Data from participants receiving SoC plus placebo will be pooled across agents for comparative analyses and hypothesis testing.

If there are supply limitations on any test agent, the sub-study containing that product, or its matching placebo, will be temporarily closed to enrollment.

The initial SoC may include remdesivir in accordance with local or national guidelines, based on the results of the ACTT-1 study. Participants will receive remdesivir according to the FDA-approved package insert as follows:

- For participants not already receiving remdesivir, remdesivir will be administered as a 200 mg intravenous (IV) loading dose on Day 1, followed by a 100 mg once-daily IV maintenance dose.

- Participants who have already been receiving remdesivir as SoC prior to Day 1 will continue to receive a 100 mg once-daily IV maintenance dose up to a total of 10 days (inclusive of the days of treatment prior to Day 1).

Duration of SoC therapy:

- Remdesivir IV component – up to 10 days while hospitalized (see Section 6.1.2), per FDA package insert.
- Other SoC as determined by the treating site in accordance with local or national guidelines

1.1.8. *Schedule of Assessments*

In addition to the table below, please consult the appropriate appendix for each sub-study (Section 1.8 of Appendices 1, 2, and 3) for additional agent- and sub-study-specific assessments. Also see Table 8-2 for a schedule of blood collections.

Participants may be discharged from the hospital early, but should be present in the clinic for in-person (outpatient) follow-up visits according to the schedule in **Table 1-1** below. These should occur on Day 8, Day 11, Day 15, and Day 29 and every effort should be made to encourage participants to return to the study site for these visits. (A telephone follow-up is scheduled for Day 22.) Participants who are discharged at any time are considered to have met the study endpoint and will continue to be followed via the scheduled outpatient visits and/or telephone calls. Participants who are transferred to another hospital that is not a study site will be followed by telephone calls; if the participant is enrolled in a sub-study involving self-administered medication, the participant will be given a supply of that medication and instructed to continue taking it. It is anticipated that transfer to another hospital will be an extremely rare occurrence.

Day 29 assessments should not be performed earlier than Day 29 (to assess Day 28 endpoints), with a visit window of +3 days; therefore, the Day 29 visit may be conducted on Day 29 through Day 32.

Table 1-1. Schedule of Assessments (SOA)

	Screen	*Baseline* [1]	*Study Intervention Period (while hospitalized)*	*Follow-up Visits After Discharge* [2]					
**Day (± Window)**	**−1 or 1**	**1** [1]		**8 (± 1)**	**11 (± 1)**	**15 (± 2)**	22 [3] (± 3)	**29 (+ 3)**	60 [4] (± 5)
**ELIGIBILITY**									
Informed consent	X								
Demographics & Medical History	X								
Review SARS-CoV-2 results	X								
**STUDY INTERVENTION**									
Randomization		X							
Administration of investigational agents		• Remdesivir: if included as SoC, given as daily infusion up to a total of 10 days or discharge.<br>• Immune-modulator: *See corresponding sub-study for dosing schedule.*							
**STUDY PROCEDURES**									
Targeted physical exam	X								
Vital signs including SpO_2 [5]		X	Daily through hospital discharge or Day 29 [6]	X	X	X		X	
Clinical data collection [7]		X	Daily through hospital discharge or Day 29 [6]	X	X	X	X	X	X
Adverse event evaluation (including AESI) [8]		X	Daily through hospital discharge or Day 29 [6]	X	X	X	X	X	X
Prior/concomitant medications review	X (from Day –7)	X	Daily through hospital discharge or Day 29 [6]	X	X	X	X	X	
**SAFETY LABORATORY**									
Safety hematology, chemistry and liver function tests	X [9]	X	Day 3, 5, 8, 11, 15 (all ±1 day) if hospitalized [10]	X [10,11]	X [11]	X [11]		X [11]	
Pregnancy test and counseling for females of childbearing potential	X								
**PHARMACOKINETICS**									
Blood sample for serum <u>or</u> plasma (pharmacokinetics)		X	Day 8 (±1 day) if hospitalized [10]	X [10]				X [10]	

	Screen	*Baseline* [1]	*Study Intervention Period (while hospitalized)*	*Follow-up Visits After Discharge* [2]					
**Day (± Window)**	**−1 or 1**	**1** [1]		**8 (± 1)**	**11 (± 1)**	**15 (± 2)**	22 [3] (± 3)	**29 (+ 3)**	60 [4] (± 5)
**RESEARCH LABORATORY**									
Blood for serum and plasma (future secondary research) [12]		X	Day 3, 8, 15 (all ± 1 day) if hospitalized	X		X		X	
Whole blood for genetic testing [12]		X							

Notes:

[1] Baseline assessments should be performed within 24 hours prior to first study product administration. Results of Day 1 (baseline) laboratory assessment do not need to be reviewed to determine if initial study product should be given.

[2] In-person follow-up visits for Days 8, 11, 15, and 29 are strongly preferred and should be encouraged by any means. If a participant cannot return to the site, a telephone follow-up visit (see footnote 3) will assess adverse events, clinical status (ordinal scale), readmission to a hospital, concomitant medications, and mortality only. If the participant is still hospitalized on any of these days, then the inpatient "daily until discharge" assessments (including concomitant medications) will be performed.

[3] Day 22 telephone-only follow-up will assess adverse events, clinical status (ordinal scale only), readmission to a hospital, concomitant medications, and mortality in participants who have been discharged from the hospital. If the participant is still hospitalized on Day 22, then the inpatient "daily until discharge" assessments (including concomitant medications) will be performed.

[4] Day 60 telephone-only follow-up will assess adverse events, clinical status (ordinal scale only), readmission to a hospital, and mortality.

[5] Vital signs include temperature, systolic blood pressure, heart rate, respiratory rate, O_2 saturation, and level of consciousness. Vital signs collected as part of standard care may be used. Height/weight will be assessed at baseline only.

[6] Daily until hospital discharge or through Day 29, whichever comes first.

[7] Refer to Sections 8.1.2 and 8.1.3 of the protocol for details of clinical data to be collected including ordinal scale, NEWS, oxygen requirement, mechanical ventilator requirement, extrapulmonary manifestations, etc. During telephone-only follow-ups, only ordinal scale will be collected.

[8] Secondary infections/superinfections are considered AEs of special interest (AESI) and are captured on the appropriate case report form. See Section 8.3.2.

[9] Laboratory tests performed in the 48 hours prior to enrollment will be accepted for determination of eligibility. See Section 8.2 for the complete list of safety laboratory parameters to be assessed at each time point.

[10] If discharged before Day 8 or Day 29, blood for the corresponding PK assessments may be drawn shortly before discharge. PK sample may be drawn at time of routine phlebotomy. All efforts will be made to collect the PK samples. If the samples are not collected due to complication or collected outside the window, this will not be considered a deviation.

[11] For outpatient visits (returning to clinic), any laboratory tests performed as part of routine clinical care within the specified visit window can be used for safety laboratory testing.

[12] Blood samples for future research, including genetic testing, are mandatory at all CTSA sites, and optional at all other US sites. These samples will not be collected at non-US sites.

Study Schema

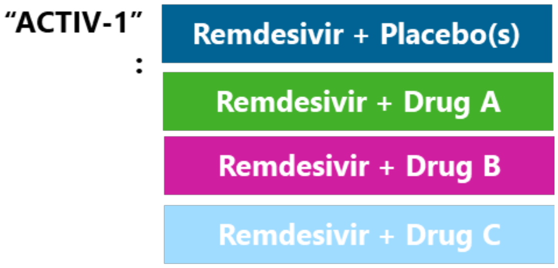

2. INTRODUCTION

2.1. Background

2.1.1. *ACTIV-1 IM – Immune Modulators & Remdesivir Multi-arm Trial*

A preliminary review of data from ACTT-1 occurring after 606 recoveries and 103 deaths (approximately 67% of the 1,063 subjects enrolled) demonstrated that subjects that received remdesivir had a 31% faster time to recovery (11 vs 15 days, recovery rate ratio 1.312 (1.119, 1.541), p<0.001), and a decrease in mortality (8.0% vs 11.6%, p = 0.059). The DSMB asked that the sponsor be unblinded early given the public health implications of the results as well as the implications for the design and conduct of ACTT-2. Based on the ACTT-1 results, the Emergency Use Authorization (EUA) from FDA, and the subsequent (as of 22 October 2020) FDA approval for COVID-19 treatment in hospitalized adults, remdesivir is now considered the SoC in the US for hospitalized COVID-19 patients. Note, however, that while remdesivir has demonstrated efficacy in the treatment of COVID-19, the mortality rate is still high. Infection by pathogenic coronaviruses (e.g. SARS and SARS-CoV–2) often results in excessive cytokine and chemokine action with the development of acute respiratory distress syndrome (ARDS) (33-35). It is postulated that this dysregulated inflammatory immune response is contributing to the excessive mortality and targeting this response will further improve outcomes.

2.2. Risk/Benefit Assessment

2.2.1. *Known Potential Risks of Study Participation*

Potential risks of participating in this trial are those associated with having blood drawn, the IV catheterization, possible reactions to the study interventions (as noted in Section 2.2.2.1), and breach of confidentiality.

Drawing blood may cause transient discomfort and fainting. Fainting is usually transient and managed by having the participant lie down and elevate his/her legs. Bruising at the blood collection sites may occur but can be prevented or lessened by applying pressure to the blood draw site for a few minutes after the blood is taken. IV catheterization may cause insertion site pain, phlebitis, hematoma formation, and infusate extravasation; less frequent but significant complications include bloodstream and local infections. The use of aseptic (sterile) technique will make infection at the site where blood will be drawn or at catheter site less likely.

2.2.1.1. *Risks to Privacy*

Participants will be asked to provide personal health information (PHI). All attempts will be made to keep this PHI confidential within the limits of the law. However, there is a chance that unauthorized persons will see the participant's PHI. All study records will be kept in a locked file cabinet or maintained in a locked room at the participating clinical site. Electronic files will be password protected. Only people who are involved in the conduct, oversight, monitoring, or auditing of this trial will be allowed access to the PHI that is collected. Any publication from this trial will not use information that will identify participants. Organizations that may inspect and/or copy research records maintained at the participating site for quality assurance and data analysis include groups such as the IRB/EC, NCATS, and applicable regulatory agencies (e.g., FDA).

2.2.2. *SoC - Remdesivir*

2.2.2.1. *Potential Risks of Remdesivir*

Remdesivir has been approved in the US for the treatment of COVID-19 in hospitalized adults and pediatric patients ≥12 years of age and weighing ≥40 kg. It is still considered an investigational therapeutic in other regions including South America. Of 138 healthy adults dosed with remdesivir in four Phase 1 clinical trials, few subjects to date experienced constipation, heartburn, itching, unusual feelings in the ear, dizziness, loss of appetite, nausea, vomiting, shaking of the leg and arm, headache, loose stool, or upset stomach. These AEs were temporary, lasting only a few days, and none were serious. In clinical studies, transient elevations in ALT and AST have been observed with single doses of remdesivir up to 225 mg and multiple once daily doses of remdesivir 150 mg for up to 14 days. Mild (Grade 1) reversible PT prolongation was also noted in some subjects but without any clinically significant change in INR or other evidence of hepatic effects. The mechanism of these elevations is currently unknown. Based on these clinical observations, patients with ALT or AST >10 times the upper limit of normal will not be eligible for study enrollment. Regular laboratory assessments will be performed in order to monitor hepatic function and PT. Any observed liver function-related laboratory abnormalities or possibly related AEs will be treated appropriately and followed to resolution.

In nonclinical animal studies, toxicity studies found dose-dependent and reversible kidney injury and dysfunction. In clinical studies, no evidence of nephrotoxicity has been observed with single doses of remdesivir up to 225 mg or multiple once daily doses of remdesivir 150 mg for up to 14 days. A 150-mg dose of the solution and lyophilized formulations of remdesivir contains 9 g and 4.5 g, respectively, of sulfobutylether-beta-cyclodextrin (SBECD), for which the maximum daily recommended daily dose (based on a European Medicines Agency (EMA) safety review) is approximately 250 mg/kg. Because SBECD is renally cleared, participants with moderate or severe renal impairment may have SBECD exposures greater than those with less severe renal impairment or normal renal function. Based on this information, patients with an estimated glomerular filtration rate (eGFR) of less than 30 mL/min and requiring hemodialysis or hemofiltration will not be eligible for study enrollment. Patients with eGFR <30 mL/min may enroll to the trial as long as their renal insufficiency has remained stable for at least 1 month and they are not on hemodialysis/hemofiltration, though these participants will **not** be treated with remdesivir.

Remdesivir is a substrate for CYP2C8, CYP2D6, and CYP3A4. However, coadministration with inhibitors of these CYP isoforms is unlikely to markedly increase remdesivir levels, as its metabolism is likely to be predominantly mediated by hydrolase activity. Drug-drug interaction trials of remdesivir and other concomitant medications have not been conducted in humans. See the FDA product label and the IB for full discussions of clinical experience and risks.

There is the potential of the SARS-CoV-2 developing resistance to remdesivir, which could result in decreased efficacy. The clinical impact of the development of resistance is not clear at this time.

2.2.2.2. *Potential Benefits of Remdesivir*

A preliminary review of data from ACTT-1 occurring after 606 recoveries and 103 deaths (approximately 67% of the 1063 subjects enrolled) demonstrated that subjects that received remdesivir had a 31% faster time to recovery (11 vs 15 days, recovery rate ratio 1.312 (1.119, 1.541), p<0.001), and a decrease in mortality (8.0% vs 11.6%, p = 0.059). The DSMB asked that the sponsor be unblinded early given the public health implications of the results and implications for the design and conduct of ACTT-2. Based on the ACTT-1 results, remdesivir is now considered the standard of care for Covid-19 infected hospitalized patients. As a result, all subjects in ACTIV-1 IM will be given remdesivir as part of SoC, and the new therapeutic agents will be evaluated as add-on therapies to SoC including remdesivir.

This is a benefit to participation as remdesivir will be provided to all participants without contraindications as part of the study. In addition, society may benefit from their participation in this study resulting from insights gained about the efficacy of remdesivir combined with the new agents. Determining if additional clinical benefit can be realized by combining the antiviral with one of the new therapeutic agents being evaluated in ACTIV-1 IM for the treatment of COVID-19 may benefit society during this global COVID-19 pandemic.

2.2.2.3. *Assessment of Potential Risks and Benefits of Remdesivir*

Remdesivir is generally a well-tolerated medication. There are liver toxicities and minor (grade 1-2) increases in prothrombin time (PT) that have been observed in prior studies. These have been self-limited and resolved after cessation of the medication. There is the potential for renal toxicities as observed in pre-clinical data. By excluding from remdesivir treatment those with elevated liver transaminases and decreased kidney function (eGFR <30 mL/min or requires hemodialysis or hemofiltration), and appropriate monitoring during the study (including assessment of PT/INR), we can minimize the risk to participants. While there may not be benefits for an individual participant, there may be benefits to society if a safe, efficacious therapeutic agent can be identified during this global COVID-19 pandemic. The potential risks therefore are thought to be acceptable given the potential benefits.

2.2.3. *Therapeutic Agents*

For each of the therapeutic agents under investigation, findings from the preclinical and clinical studies are briefly described in the agent-specific appendices, including a summary of the findings described in the Investigator Brochures (IBs). The potential risks and benefits of the therapeutic agents are also summarized in the appendices.

3. OBJECTIVES AND ENDPOINTS

The overall objective of the study is to evaluate the clinical efficacy and safety of different investigational therapeutic agents relative to the control arm among hospitalized adults who have COVID-19.

Table 3-1. Trial objectives and measured endpoints

OBJECTIVES	ENDPOINTS (OUTCOME MEASURES)
Primary	
To evaluate the clinical efficacy of different investigational therapeutics relative to the control arm in adults hospitalized with COVID-19 with respect to time to recovery by Day 29.	Day of recovery is defined as the first day on which the participant satisfies one of the following three categories from the ordinal scale:<br>• Hospitalized, not requiring supplemental oxygen – no longer requires ongoing medical in-patient care;<br>• Not hospitalized, limitation on activities and/or requiring home oxygen;<br>• Not hospitalized, no limitations on activities.<br>Recovery is evaluated up until Day 29.
Key Secondary	
• To evaluate the clinical efficacy of different investigational therapeutics relative to the control arm in adults hospitalized with COVID-19 according to:<br>• Clinical status (8-point ordinal scale) assessed on Day 15 and Day 29 for the previous day	1. Death;<br>2. Hospitalized, on invasive mechanical ventilation or ECMO;<br>3. Hospitalized, on non-invasive ventilation or high flow oxygen devices;<br>4. Hospitalized, requiring supplemental oxygen;<br>5. Hospitalized, not requiring supplemental oxygen – requiring ongoing in-patient medical care (COVID-19 related or otherwise);<br>6. Hospitalized, not requiring supplemental oxygen – no longer requires ongoing in-patient medical care (i.e., in hospital for social reasons, infection control, etc.);<br>7. Not hospitalized, limitation on activities and/or requiring home oxygen;<br>8. Not hospitalized, no limitations on activities.

OBJECTIVES	ENDPOINTS (OUTCOME MEASURES)
• Mortality<br>o 14-day mortality<br>o 28-day mortality	• Date and cause of death (if applicable)
Additional Secondary	
To evaluate the clinical efficacy of different investigational therapeutics relative to the control arm as assessed by:<br>• **Clinical Severity**<br>o Ordinal scale:<br>▪ Time to an improvement of one category and two categories from Day 1 (baseline) using an 8-point ordinal scale.<br>▪ Subject clinical status using 8-point ordinal scale at Days 3, 5, 8, 11, 15, 22, and 29.<br>▪ Mean change in the 8-point ordinal scale from Day 1 to Days 3, 5, 8, 11, 15, 22, and 29.<br>▪ Total severity score (TSS): 8-point ordinal scale summarized as a daily score (for days collected) averaged over time from Day 1 through Day 29	• Clinical outcome assessed using ordinal scale daily while hospitalized and on Days 15, 22, and 29.
o Oxygenation:<br>▪ Oxygenation use up to Day 29.<br>▪ Incidence and duration of new oxygen use during the study.	• Days alive and free of supplemental oxygen (if applicable) up to Day 29
o Non-invasive ventilation/high flow oxygen:<br>▪ Non-invasive ventilation/high flow oxygen use up to Day 29.<br>▪ Incidence and duration of new non-invasive ventilation or high flow oxygen use during the study.	• Days alive and free of non-invasive ventilation/high flow oxygen (if applicable) up to Day 29
o Invasive Mechanical Ventilation / extracorporeal membrane oxygenation (ECMO):<br>▪ Ventilator / ECMO use up to Day 29.<br>▪ Incidence and duration of new mechanical ventilation or ECMO use during the study.	• Days alive and free of invasive mechanical ventilation/ECMO (if applicable) up to Day 29
• **Hospitalization**<br>o Duration of hospitalization (days).	• Days alive and out of hospitalization up to Day 29
To evaluate clinical Status at Day 60	• 8-point Ordinal Scale
To evaluate the safety of different investigational therapeutics relative to the control arm as assessed by:<br>• Cumulative incidence of SAEs through Day 29.<br>• Cumulative incidence of Grade 3 and 4 clinical and/or laboratory AEs through Day 29.<br>• Discontinuation or temporary suspension of study product administrations (for any reason)	• SAEs<br>• Grade 3 and 4 AEs<br>• WBC with differential, hemoglobin, platelets, creatinine, glucose, total bilirubin, ALP, ALT, and AST on Days 1, 3, 5, 8, 11, 15, and 29.

OBJECTIVES	ENDPOINTS (OUTCOME MEASURES)
• Changes in white blood cell (WBC) count with differential, hemoglobin, platelets, creatinine, glucose, total bilirubin, alanine aminotransferase (ALT), aspartate aminotransferase (AST), prothrombin time (PT reported as INR), d-dimer, and C-reactive protein (CRP) over time (analysis of lab values in addition to AEs noted above).	• PT/INR, d-dimer, troponin, and CRP on Days 1 and 8.
To evaluate the impact of different investigational therapeutics relative to the control arm of extrapulmonary manifestations of COVID-19.	Incidence of individual and "any" specified manifestations at Day 29.
Exploratory	
o National Early Warning Score (NEWS):<br>▪ Time to discharge or to a NEWS of ≤ 2 and maintained for 24 hours, whichever occurs first.<br>▪ Change from Day 1 to Days 3, 5, 8, 11, 15, and 29 in NEWS.	NEWS assessed daily while hospitalized and on Days 15 and 29, if feasible.
Collect blood samples for future research.	Blood draws on Days 1, 3, 8, 15, and 29.

4. STUDY DESIGN

4.1. Overall Design

ACTIV-1 IM is a master protocol designed to evaluate multiple therapeutic agents for the treatment of moderately or severely ill hospitalized patients infected with COVID-19. Participants will be assessed daily while hospitalized. If the participants are discharged from the hospital, they will have a study visit at Days 8, 11, 15, 22, and 29. For discharged participants, it is preferred that the Day 8, 11, 15, and 29 visits are in person to obtain safety laboratory tests and blood (serum and plasma) samples for secondary research as well as clinical outcome data. However, infection control or other restrictions may limit the ability of the participant to return to the clinic. In this case, these visits may be conducted by phone, and only clinical data will be obtained. The Day 22 visit does not have laboratory tests or collection of samples and is conducted by phone. The Day 60 assessment will be conducted by phone. The primary outcome is time to recovery by Day 29 (see **Table 3-1** for definition of recovery based on the 8-point ordinal scale). Key secondary outcomes include treatment-related improvements in the 8-point ordinal scale at Day 15, evaluation of safety, and 14-day and 28-day mortality.

4.2. Scientific Rationale for Study Design

ACTIV-1 IM utilizes a master protocol to evaluate multiple therapeutic agents for the treatment of COVID-19 infection in hospitalized patients. Approximately 400 agents were identified and screened for clinical readiness by a panel of experts, and 170 agents were selected for further evaluation based on mechanism of action and need for further clinical testing (i.e., not previously studied or plan for study in other trials or trial networks). From this triaging exercise, three agents were initially selected for study in the ACTIV-1 IM master protocol. All three agents are immune modulators with substantial clinical experience available. Details about the initial set of selected agents are provided elsewhere (see Sections 2 and 4 and Appendices 1-3); additional agents may be entered into the ACTIV-1 IM master protocol after the study begins, depending on availability and network capacity. In that case, agent-specific information will be appended to the protocol for any new agents entering the study, and the randomization algorithm will be adjusted accordingly.

ACTIV-1 IM builds upon findings and the model used for ACTT-1 and -2. Including multiple therapeutic agents under a single protocol avoids duplication of effort in terms of infrastructure, trial governance, information systems (EDC, web-based randomization, etc.) and other aspects of study management. Implementation of the master

protocol facilitates discontinuation of less promising agents and addition of possibly newly emergent agents that become available after the study begins, without stopping and starting the study itself for extended pauses.

All test agents are evaluated as add-on therapies to the local SoC at each clinic. The master protocol design allows for the efficacy and safety of each agent to be determined based on comparisons with a pooled control group, consisting of patients receiving SoC plus placebo. Sharing control patients across all test agents substantially reduces the sample size requirements for the study.

4.3. Justification for Dose

4.3.1. *Justification for Dose of Remdesivir*

The dose of remdesivir used in this study is the dose specified on its FDA-approved product label, and the same dose that was used in the ACTT-1 and ACTT-2 trials.

4.3.2. *Justification for Dose of Investigational Agents*

The dose of each investigational agent is provided in the agent-specific appendices to this master protocol.

5. STUDY POPULATION

Male and non-pregnant female adults ≥18 years of age or older with COVID-19 and who meet all eligibility criteria will be enrolled at up to approximately 90 clinical trial sites globally. The target population should reflect the community at large. The estimated time from screening (Day -1 or Day 1) to end of study for an individual participant is approximately 60 days.

Subject Inclusion and Exclusion Criteria must be confirmed by any clinician named on the delegation log. If there is any uncertainty, the PI should make the decision on whether a potential participant is eligible for study enrollment. There is no exclusion for receipt of SARS-CoV-2 vaccine (experimental or licensed).

Following are the inclusion and exclusion criteria for the master protocol population. These criteria apply to all patients enrolled, regardless of therapeutic agent to be assigned for treatment. Any agent-specific exclusion criteria that are required will be applied during recruitment in addition to these common criteria and are described in the appendices corresponding to each agent under study.

5.1. Inclusion Criteria

1. Admitted to a hospital or awaiting admission in the ED with symptoms suggestive of COVID-19.
2. Subject (or legally authorized representative) provides informed consent prior to initiation of any study procedures.
3. Subject (or legally authorized representative) understands and agrees to comply with planned study procedures.
4. Male or non-pregnant female adults ≥18 years of age at time of enrollment.
5. Has laboratory-confirmed (within 14 days prior to enrollment) SARS-CoV-2 infection as determined by PCR or other commercial or public health assay in any specimen.
6. Ongoing illness of any duration, and at least one of the following:
   - Radiographic infiltrates by imaging (chest x-ray, CT scan, etc.), OR
   - Blood oxygen saturation (SpO_2) ≤ 94% on room air, OR
   - Requiring supplemental oxygen, OR
   - Requiring mechanical ventilation or ECMO.
7. Women of childbearing potential must agree to either abstinence or use of at least one primary form of contraception not including hormonal contraception from the time of screening through Day 60.
8. Agrees not to participate in another interventional trial for the treatment of COVID-19 through Day 60.
   Exception 1: Participant may co-enroll in ACTIV-4 (ACTIV-4A and ACTIV-4C).
   Exception 2: Participants in ACTIV-2 who have been hospitalized may be enrolled in ACTIV-1 as long as ACTIV-2 study therapy has been discontinued. They will remain in ACTIV-2 follow-up.

Exception 3: If participant is already participating in a COVID-19 vaccine trial but develops severe COVID-19, participant is eligible for this study, assuming all other inclusion/exclusion criteria are met.

5.2. Exclusion Criteria at Screening

1. ALT or AST >10 times the upper limit of normal.
2. Estimated glomerular filtration rate (eGFR) <30 mL/min (including patients receiving hemodialysis or hemofiltration).
   Exception: Participants with an eGFR <30 mL/min may enroll as long as their renal insufficiency has been stable without renal replacement therapy for ≥1 month and they are not current candidates for renal replacement therapy. **These participants will not receive remdesivir.**
3. Neutropenia (absolute neutrophil count <1000 cells/μL) (<1.0 x 10^3/μL or <1.0 GI/L).
4. Lymphopenia (absolute lymphocyte count <200 cells/μL) (<0.20 x 10^3/μL or <0.20 GI/L)
5. Pregnancy or breast feeding.
6. Anticipated discharge from the hospital or transfer to another hospital which is not a study site within 72 hours.
7. Known allergy to any study medication.
8. Received targeted immune-modulator treatments (such as anti-interleukin-1 [IL-1], anti-IL-6 [tocilizumab or sarilumab], anti-IL-17, T-cell or B-cell targeted therapies [e.g., rituximab], JAK inhibitors [including baricitinib], TNF inhibitors, or interferon) within 4 weeks or 5 half-lives prior to screening, whichever is longer. Steroid dependency, defined as need for prednisone at a dose >10 mg (or equivalent) for >1 month within 2 weeks of screening, is exclusionary.
   Exception 1: Dexamethasone is permitted for the treatment of COVID-19 in patients who are already mechanically ventilated and in patients who require supplemental oxygen at screening, but who are not mechanically ventilated in accordance with national guidelines.
   Exception 2: Infusion of convalescent plasma given for treatment of COVID-19 while on-study is also allowed.
   Exception 3: Monoclonal antibody therapy given for COVID-19 treatment at any time prior to enrollment is also allowed.
9. Known or suspected history of untreated tuberculosis (TB). TB diagnosis may be suspected based on medical history and concomitant therapies that would suggest TB infection. Participants are also excluded if they have known, latent TB treated for less than 4 weeks with appropriate anti-tuberculosis therapy per local guidelines (by history only, no screening required).
10. Known or suspected serious, active bacterial, fungal, or viral infection (excepting SARS-CoV-2 and including, but not limited to, active HBV, HCV, or HIV with the latter defined as a CD4 count <200 or an unsuppressed HIV viral load), or other infection (besides COVID-19) that in the opinion of the investigator could constitute a risk when taking investigational product.
    Note: Broad-spectrum empiric antibiotic usage does not exclude participation.
11. Have received any live vaccine (or live attenuated) within 3 months before screening, or intend to receive a live vaccine (or live attenuated) during the study.
    Exception: Use of prior non-live (inactivated) vaccinations is allowed for all participants, including any vaccine for COVID-19.
12. Severe hepatic impairment (defined as liver cirrhosis Child stage C).
13. Known severe heart failure (New York Heart Association [NYHA] III-IV) or new-onset left-systolic or global cardiac dysfunction in the setting of COVID-19.
    Exception: Right-sided heart dysfunction or pulmonary hypertension thought related to COVID-19 is permitted.
14. In the Investigator's judgment, the patient has any advanced organ dysfunction that would not make participation appropriate.

5.3. Exclusion of Specific Populations

Children and adolescents will not be included in this trial. The SoC, remdesivir, has only been used in a small number of pediatric patients, and does not have FDA approval for use in any patients under 12 years of age or in patients 12 years of age or older who weigh less than 40 kg. Initial information about the epidemiology of COVID-19 indicates that the overwhelming burden of severe disease occurs among older adults, especially those with comorbidities. Given significant gaps in knowledge in this population, and a low incidence of severe morbidity/ mortality in children, and that none of the agents initially selected for study or likely to be selected in the future have had or will have demonstrated efficacy in COVID-19, this research is not known to have the prospect of direct benefit to individual child participants, and the risk/benefits do not warrant inclusion of this population into this trial at this time.

In nonclinical reproductive toxicity studies, the SoC, remdesivir, demonstrated no adverse effect on embryo-fetal development when administered to pregnant animals. Embryonic toxicity was seen when remdesivir was initiated in female animals prior to mating and conception, but only at a systemically toxic dose. Remdesivir has not been studied in pregnant women. Information on nonclinical reproductive toxicity studies for the therapeutic agents selected for study is provided in the agent-specific appendices.

In animal studies, remdesivir metabolites have been detected in the nursing pups of mothers given remdesivir. It is not known whether remdesivir is secreted in human milk. Because the effects of the SoC, remdesivir, on the breastfeeding infant is not known, and because the effects of one or more therapeutic agents selected for evaluation in this study may also be unknown, women who are breast feeding are not be eligible for the trial.

5.4. Inclusion of Vulnerable Subjects

Certain human subjects are categorized as vulnerable populations and require special treatment with respect to safeguards of their well-being. For this clinical trial, examples include cognitively impaired or mentally disabled persons and intubated individuals who are sedated. When it is determined that a potential research participant is cognitively impaired, federal and institutional regulations permit researchers to obtain consent from a legally authorized representative (LAR). The study team will obtain consent from these vulnerable participants using an IRB-approved protocol- specific process for consent using a LAR.

For participants for whom a LAR gave consent, during the course of the study, if the participant regains the capacity to consent, informed consent must be obtained from the participant and the participant offered the ability to leave the study if desired.

5.5. Lifestyle Considerations

During this study, participants are asked to:

- Avoid getting pregnant, breastfeeding, or impregnating their partner during the study from Day 1 through Day 60.
- Agree not to participate in another interventional trial for the treatment of COVID-19 or SARS-CoV-2 through Day 60, with the exception of ACTIV-4 (ACTIV-4A and ACTIV-4C). This includes interventional trials that evaluate treatment of SARS-CoV-2 infection as well as the disease pathogenesis (e.g., treatment trials for the COVID-19-related thrombo-occlusive disease, respiratory complications, and dysregulated immune response to the virus). Co-enrollment for natural history studies of COVID-19 or SARS-CoV-2 infection and follow-up from ACTIV-2 and COVID-19 vaccine studies is permitted; however, participation in both ACTIV-1 IM and other studies can only occur if the total recommended blood collection volumes are not exceeded.

5.6. Screen Failures

Following consent, after the screening evaluations have been completed, the investigator or designee is to review the inclusion/exclusion criteria and determine the participant's eligibility for the study. If there is any uncertainty, the PI should make the decision on whether a potential participant is eligible for study enrollment.

Only basic demographic information and the reason(s) for ineligibility will be collected on screen failures. Prospective participants who are found to be ineligible will be told the reason(s) for ineligibility.

Individuals who do not meet the criteria for participation in this study (screen failure) because of an abnormal laboratory finding may be rescreened once.

5.7. Strategies for Recruitment and Retention

5.7.1. *Recruitment*

It is anticipated that patients with COVID-19 will present to participating hospitals, and that no external recruitment efforts towards potential participants are needed. Recruitment efforts may also include dissemination of information about this trial to other medical professionals / hospitals.

The hospitals that will constitute the ACTIV-1 IM network span the United States and South America. As the pandemic migrates to different locations during the coming months, we anticipate being able to effectively follow the migration and focus recruitment efforts at hot spots through our network hospitals in different locales.

The IRB/EC will approve the recruitment process and all materials provided prior to any recruitment to prospective participants directly.

Screening will begin with a brief discussion with study staff. Some patients will be excluded based on demographic data and medical history (i.e., pregnant, <18 years of age, renal failure, etc.). Information about the study will be presented to potential participants (or legally authorized representative) and questions will be asked to determine potential eligibility. Screening procedures can begin only after informed consent is obtained.

5.7.2. *Retention*

Retention of participants in this trial is very important for determining the primary endpoint of time to recovery by Day 29. As such, after hospital discharge, participants will be reminded of subsequent study visits and every effort will be made to accommodate the participant's schedule to facilitate follow-up within the specified visit window. Additionally, there are many circumstances that influence the ability to obtain outcome information after discharge. Follow-up visits may be conducted by phone if in-person visits are not feasible.

5.8. Compensation Plan for Subjects

Compensation, if any, will be determined locally and in accordance with local IRB/EC requirements, and subject to local IRB/EC approval.

5.9. Costs

There is no cost to participants for the research tests, procedures/evaluations, and study product (including remdesivir) while taking part in this trial. Procedures and treatment for clinical care including costs associated with hospital stay may be billed to the participant, participant's insurance, or third party.

6. STUDY PRODUCT

6.1. Study Product(s) and Administration

Participants will first be randomized to one of the agents currently active in the study, and then to either the agent or its matching placebo. The dosing regimen and administration of each agent and matching placebo are described in the agent-specific appendices.

If there are supply limitations on any product, the arms containing that product and the corresponding placebo will be temporarily closed to enrollment. Currently, the trial team anticipates no difficulty in obtaining remdesivir for

the SoC for this study; however, if remdesivir supply should become limited for the trial, the treating physician should enroll the patient without remdesivir using local SoC.

6.1.1. *Study Product Description*

The SoC, remdesivir, is a single diastereomer monophosphoramidate prodrug designed for the intracellular delivery of a modified adenine nucleoside analog GS-441524. In addition to the active ingredient, the sterile, preservative-free lyophilized formulation of remdesivir contains the following inactive ingredients: SBECD, and hydrochloric acid and/or sodium hydroxide. Use of remdesivir in this study is investigational.

Descriptions of the therapeutic agents selected for evaluation in the study are provided in the agent-specific appendices.

6.1.2. *Dosing and Administration*

Participants who were enrolled with baseline eGFR <30 mL/min **will not receive remdesivir**.

Participants who have not yet received remdesivir as SoC prior to enrollment will receive remdesivir as a 200 mg intravenous (IV) loading dose on Day 1, followed by a 100 mg once-daily IV maintenance dose for 5 days during hospitalization for patients not requiring invasive mechanical ventilation and/or extracorporeal membrane oxygenation (ECMO). If a patient does not demonstrate clinical improvement, treatment may be extended for up to 5 additional days for a total treatment duration of up to 10 days. For patients requiring invasive mechanical ventilation and/or ECMO, the recommended total treatment duration is 10 days.

If a participant has been receiving remdesivir as SoC prior to enrollment, their first on-study dose will be the 100 mg (maintenance) dose to a maximum of 10 days total, as determined by the PI. Patients who have completed 5 days of remdesivir may, at the treating physician's discretion, receive no more remdesivir or continue to a total of 10 days. Patients who have completed 10 days of remdesivir should not receive any more doses.

Dosing of the SoC and investigational agents should not occur at the same time. In fact, no physical biochemical compatibility studies have been conducted to evaluate the co-administration of the remdesivir with other investigational agents.

Any dose that is delayed may be given later that calendar day. Any dose that is missed (not given that calendar day) is not made up. The treatment course continues as described above even if the participant becomes SARS-CoV-2 PCR negative.

Dosing and duration of therapy of the therapeutic agents selected for study are described in the agent-specific appendices.

6.1.3. *Dose Escalation*

Not Applicable.

6.1.4. *Dose Modifications*

SoC (remdesivir):
If the eGFR decreases to an eGFR <25 mL/min, the remdesivir infusion should not be given on that day. The infusion may be resumed on the next day if the eGFR returns to ≥30 mL/min. If the participant's renal function worsens to the point that they require hemodialysis or hemofiltration, remdesivir will be discontinued.

If the ALT increases to >10 times upper limits of normal, discontinue remdesivir. If elevated ALT (>5 times upper limit of normal) is accompanied by signs or symptoms of liver inflammation, discontinue remdesivir without the option to restart.

If any of the therapeutic agents selected for study require dose modifications based on biomarkers or safety signals, these adjustments are described in the agent-specific appendices.

6.1.5. *Overdosage*

There is no known antidote for an overdose of remdesivir. In the case of overdose, the participant should receive supportive therapy based on the participant´s signs and symptoms. Availability of antidotes for study agents is discussed in the agent-specific appendices.

6.1.6. *Preparation/Handling/Storage/Accountability*

6.1.6.1. *Acquisition and Accountability*

Investigational products (IP) will be shipped to the site either directly from participating companies, or from other regional or local drug repositories. SoC (remdesivir) will be provided to the sponsor's designated distributor and the sponsor's distributor will provide the SoC agent to the sites. All other supplies should be provided by the site. Multiple lots of each IP may be supplied.

Study products received at the sites will be open label and not kit specific, unless specified in the protocol-specific Manual of Procedures (MOP) or pharmacy manual. For IV infusion agents, drug preparation will be performed by the participating site's unblinded research pharmacist on the same day of administration to the participant. For oral agents, the investigational agent and the placebo will be shipped to the site ready to administer. The participating site's unblinded research pharmacist will provide either the investigational agent or the placebo pill to the treating physician at the time of assignment to the sub-study and randomization to the appropriate arm. See the MOP Appendices for detailed information on the preparation, labeling, storage, and administration of investigational products.

Accountability:

The site PI is responsible for study product distribution and disposition and has ultimate responsibility for study product accountability. The site PI may delegate to the participating site's research pharmacist responsibility for study product accountability. The participating site's research pharmacist will be responsible for maintaining complete records and documentation of study product receipt, accountability, dispensation, storage conditions, and final disposition of the study product(s). Time of study drug administration to the participant will be recorded on the appropriate data collection form (CRF). All study product(s), whether administered or not, must be documented on the appropriate study product accountability record or dispensing log. The Sponsor's monitoring staff will verify the participating site's study product accountability records and dispensing logs per the site monitoring plan. Refer to the protocol-specific MOP for details on storing study medications.

Destruction:

After the study treatment period has ended or as appropriate over the course of the study after study product accountability has been performed, used active and placebo product can be destroyed on-site following applicable site procedures with a second staff member observing and verifying the destruction.

Unused product at the end of the study for any of the agents selected for study should be saved until instructed by sponsor.

6.1.6.2. *Formulation, Appearance, Packaging, and Labeling*

SoC (remdesivir):

The lyophilized 100 mg single-dose vial formulation of remdesivir is a preservative-free, white to off-white to yellow, lyophilized powder to be reconstituted with 19 mL of sterile water for injection and immediately diluted into either a 100 mL or 250 mL 0.9% sodium chloride (NaCl) infusion bag prior to IV infusion. A clear solution should result. Following reconstitution, each vial contains a 5 mg/mL remdesivir concentrated solution with sufficient volume to allow withdrawal of 20 mL (100 mg of remdesivir).

It is supplied as a sterile product in a single-use, Type 1 clear glass vial. In addition to the active ingredient, the lyophilized formulation of remdesivir contains the following inactive ingredients: SBECD, hydrochloric acid, and/or sodium hydroxide. For more information, refer to the MOP.

Remdesivir sourced for this study is labeled with the EUA label and will include the statement “Caution: New Drug Limited by Federal (USA) Law to Investigational Use.”

Interventional Agents:

Formulation, appearance, packaging, and labeling for each of the agents selected for study are described in the agent-specific appendices.

6.1.6.3. *Product Storage and Stability*

SoC (remdesivir):

Ambient vials of the lyophilized formulation of remdesivir should be stored below 30°C (below 86°F). After reconstitution, use the vials immediately to prepare the diluted solution. The diluted solution in the infusion bags should not exceed 4 hours at room temperature (20°C to 25°C or 68°F to 77°F) prior to administration or 24 hours at refrigerated temperature (2°C to 8°C or 36°F to 46°F). See MOP for additional information.

Interventional Agents:

Product storage and stability for each of the agents selected for study are described in the agent-specific appendices.

6.1.6.4. *Preparation*

Refer to the protocol-specific MOP for details about preparation.

SoC (remdesivir):

Remdesivir does not meet the criteria for a hazardous compound as defined by NISOH and ASHP hazard classification systems. The SoC may be prepared in a clean room but do not need to be prepared or handled in a fume hood.

Interventional Agents:

Preparation of each of the agents selected for study are described in the agent-specific appendices.

Measures that minimize drug contact with the body should always be considered during handling, preparation, and disposal procedures as indicated in the IB.

6.2. Measures to Minimize Bias: Randomization and Blinding

Randomization will be stratified by:

- Geographic Region (regions will be identified in the Statistical Analysis Plan)
- Severity of illness at enrollment (by ordinal scale)
    - Severe disease:
        - Hospitalized, on invasive mechanical ventilation or ECMO, or
        - Hospitalized, on non-invasive ventilation or high flow oxygen devices.
    - Moderate disease:
        - Hospitalized, requiring supplemental oxygen, or
        - Hospitalized, not requiring supplemental oxygen.

Randomization will proceed in two steps, with stratification at each step. At the first step, each participant will be assigned with equal probability to one of the agents available at the time the patient is enrolled and for which the patient is eligible to receive, after applying any agent-specific safety exclusions. At the second step, each participant will be assigned to either the test agent or its matching placebo in an n:1 ratio, where n = the number of agents

currently active in the master protocol and for which the patient is eligible to receive. With SoC and three agents active simultaneously, and if the participant meets the criteria to receive all three agents, the allocation ratio at the first step will be 1:1:1 and the second step 3:1 (agent vs placebo) or overall randomization ratio of 1:1:1:1. If 3 agents are available, but a patient is only eligible to receive 2 of them, the allocation ratio at the first step will be 1:1, and at the second step will be 2:1 (agent vs placebo) or overall randomization of 1:1:1. Inclusion of a matching placebo for each agent enables masking of study participants and clinical personnel to treatment assignment at the second stage. Data from patients receiving SoC plus placebo will be pooled across agents for comparative analyses and hypothesis testing. Comparative analyses of a particular agent will include the subset of pooled placebo patients who enrolled concurrently with the new agent. That is, patients enrolled to control arm prior to a new agent entering the trial will not be included in comparative analyses of that newly added investigation agent.

Additional details of the randomization procedure will be described in the MOP.

6.3. Study Intervention Compliance

Each dose of study product will be administered by a member of the clinical research team who is qualified and licensed to administer the study product OR trained and qualified hospital personnel under the direction of the site investigator. Administration, including date and time, will be entered into the case report form (CRF).

6.4. Concomitant Therapy

6.4.1. *Permitted Concomitant Therapy and Procedures*

Therapy prior to enrollment with any other experimental treatment or off-label use of marketed medications that are intended as specific treatment for COVID-19 or the SARS-CoV-2 infection (i.e., post-exposure prophylaxis [PEP]) are permitted (except as detailed in the inclusion/exclusion criteria) but must be discontinued on enrollment. For example: anti-SARS-CoV-2 monoclonal antibody allowed prior to screening but must be discontinued. Patients who were enrolled on ACTIV-2 and had their study treatment discontinued may enroll to ACTIV-1 IM while remaining in ACTIV-2 follow-up. There is no waiting period between discontinuation of these treatments and administration of study products. However, these prior treatments and their end date should be documented on the Concomitant Medication (CCM) form.

Participants who are taking other immunosuppressive or immunomodulatory drugs for other medical conditions except as noted below as prohibited medications, may continue with the treatment. Agent specific appendices should be referenced for further prohibitions on concomitant therapies.

Local standard of care per written policies or guidelines for treatment for supportive care of COVID-19 or SARS-CoV-2 infection (i.e., not just an individual clinician decision) are permitted. This includes the evolving SoC with anticoagulant therapies as well as co-enrollment to ACTIV-4 (ACTIV-4A and ACTIV-4C). Dexamethasone is permitted for the treatment of COVID-19 in patients who are mechanically ventilated and in patients who require supplemental oxygen but who are not mechanically ventilated in accordance with national guidelines. Infusion of convalescent plasma is also allowed if it is standard of care at the treating site.

Venous thromboembolism (VTE) prophylaxis is recommended for all patients unless there is a major contraindication such as active bleeding events or history of heparin-induced thrombosis.

6.4.2. *Prohibited Concomitant Therapy*

See Section 5.5 (Lifestyle Considerations) for details on allowable participation on other clinical trials.

If there are NO written policies or guidelines for local standard of care, concomitant use of any other experimental treatment or off-label use of marketed medications intended as specific treatment for COVID-19 or SARS-CoV-2 infection are prohibited. This includes medications that target the host immune response.

Any immune-modulating therapies are prohibited. This includes, but is not limited to, TNF inhibitors, anti-interleukin-1 [IL-1], anti-IL-6 [tocilizumab or sarilumab], anti-IL-17, T-cell or B-cell targeted therapies, JAK inhibitor(s) (including baricitinib), and interferon within 4 weeks or 5 half-lives prior to screening, whichever is

longer. Anti-SARS-CoV-2 monoclonal antibody therapy given for COVID-19 treatment prior to enrollment is allowed. The use of any biologics prior to screening will be used to determine eligibility.

Dexamethasone or other steroids are permitted for the treatment of COVID-19 in patients who are mechanically ventilated and in patients who require supplemental oxygen but who are not mechanically ventilated in accordance with national guidelines. The use of corticosteroids outside of this indication is prohibited.

The use of chloroquine or hydroxychloroquine for treatment of COVID-19 is prohibited.

Other concomitant medications will be assessed only from 7 days prior to enrollment to Day 29 or upon discharge, whichever comes first. Report all prescription medications and vaccines (as well as over the counter, vitamins, herbal supplements, and/or cannabis or other specific categories of interest) taken during this time period. Record medications once regardless of the number of times it was given during the time period. For example, vasopressors or insulin should be recorded when first started (the start date) and end date if ended before Day 29 or discharge. Record all antipyretics and other medications given for symptomatic care, if they are administered while an inpatient. However, record these medications only once, even if given multiple times, as needed during hospital course. Do not report medications that the participant did not actually receive during study (e.g., prn medications that were never given).

Medications must be recorded along with:

- Reason for use
- Dates of administration including start and end dates
- Dosage information including dose and frequency

The Sponsor's Medical Safety Physician or equivalent representative should be contacted if there are any questions regarding concomitant or prior therapy.

Due to potential interactions the additional medications may be disallowed for specific agents on the trial as detailed in the sub-study appendices. If there is a medical need to utilize any of the medications disallowed, then the Investigator should discuss appropriate steps with the Sponsor.

6.4.3. *Rescue Medicine*

Not Applicable.

6.4.4. *Non-Research Standard of Care*

Not Applicable.

7. STUDY INTERVENTION DISCONTINUATION AND SUBJECT DISCONTINUATION/WITHDRAWAL

7.1. Halting Criteria and Discontinuation of Study Intervention

7.1.1. *Individual Study Product Halting*

Study product administration for any given participant may be stopped for SAEs, clinically significant adverse events, severe laboratory abnormalities, or any other medical conditions that indicate to the Investigator that continued dosing is not in the best interest of the patient.

In addition, a participant in this clinical study may discontinue study drug at their request for any reason. Every effort should be made to encourage participants to remain in the study for the duration of their planned outcome assessments. Participants should be educated on the continued scientific importance of their data, even if they discontinue study drug.

Unless the participant withdraws consent, those who discontinue study drug early will remain in the study. The reason for participant discontinuation of study drug should be documented in the case report form.

See Section 6.1.4 for information about dosing modifications of remdesivir due to laboratory abnormalities.

7.1.2. *Study Halting*

There will be close oversight by the protocol team and frequent DSMB reviews of the safety data. The DSMB may recommend halting one or more of the therapeutic agents due to safety, futility, or efficacy issues observed during the study, or they may recommend halting the study as a whole, if a safety issue or issue relating to the COVID-19 pandemic that negatively impacts study continuation arises.

A sub-study will be paused for new enrollment/study drug administration and will be reviewed by the DSMB if the following rule is met:

- After a sub-study has enrolled at least 10% of its planned study population, the proportion of study participants who die within 14 days of study drug administration or first dose administration in the same sub-study exceeds 24% (based on the 11.9% death rate in the ACTT-1 placebo arm; Beigel et al. 2020).

7.2. Withdrawal from the Study

Participants are free to withdraw from participation in the study at any time upon request, without any consequence. Participants should be listed as having withdrawn consent only when they no longer wish to participate in the study and no longer authorize the Investigators to make efforts to continue to obtain their outcome data.

Participants who withdraw from this study or are lost to follow-up after signing the informed consent form (ICF) and administration of the study product, will not be replaced. The reason for participant withdrawal from the study will be recorded on the appropriate CRF.

7.3. Lost to Follow-Up

A participant will be considered lost to follow-up if he or she fails to appear for all follow-up assessments. In lost to follow-up cases, attempts to contact the participant should be made and these efforts should be documented in the participant's records.

7.4. Readmission

If a participant is discharged from the hospital and then readmitted prior to Day 14, they may be given the remainder of the study product (i.e., remdesivir for up to a total of 10 days). If the participant did not withdrawal his/her consent to participate in the study, there is no need to reconsent upon readmission to the study hospital. However, the site will need to inform them that since he/she was readmitted, study product administration will resume and confirm that they still agree to receive study product. If the participant is re-admitted with diminished mental capacity, the site should discuss continued study participation with a LAR.

The site should not complete the Discontinuation of Treatment form since the participant came back to the study hospital to be readmitted. For all data collection procedures required for those readmitted, please see the MOP.

8. STUDY ASSESSMENTS AND PROCEDURES

8.1. Screening and Efficacy Assessments

8.1.1. *Screening Procedures*

Screening procedures may be done over one to two calendar days (from Day -1 to Day 1). However, in many cases all the screening assessments can be done in less than 24 hours. If that is the case, Day 1 pre-study product administration baseline assessments, specimen collection and the initial study product administration can occur on the same calendar day as the screening procedures.

ACTIV-1 IM: Randomized Master Protocol for Immune Modulators for Treating COVID-19

After the informed consent, the following assessments are performed to determine eligibility and obtain baseline data:

- Confirm the positive SARS-CoV-2 test result (per inclusion criteria).
- Take a focused medical history, including the following information:
  - Day of onset of COVID-19 signs and symptoms.
  - History of vaccinations within 4 weeks before screening and planned vaccinations.
    - **Exception:** COVID-19 vaccine should **always** be captured even if administered more than 4 weeks before screening. Participants on COVID-19 vaccine trials are eligible for this trial if they develop severe COVID-19.
    - Exclusionary vaccine history includes:
      - Has received any live vaccine (or live attenuated) within 4 weeks before screening, or intend to receive a live vaccine (or live attenuated) during the study. Note: Use of non-live (inactivated) vaccinations is allowed for all participants.
  - History of chronic medical conditions including chronic oxygen requirement prior to onset of COVID-19. See conditions included in exclusion criteria (Section 5.2) and on the Medical History (CMX) data collection form.
    - Exclusionary medical history includes:
      - Has diagnosis of current active tuberculosis (TB) or, if known, latent TB treated for less than 4 weeks with appropriate anti-tuberculosis therapy per local guidelines (by history only, no screening required).
      - Suspected serious, active bacterial, fungal, or viral (excepting SARS-CoV-2 and including, but not limited to, active HBV, HCV, or HIV with the latter defined as a CD4 count <200 or an unsuppressed HIV viral load) infection, or other infection (besides COVID-19) that in the opinion of the investigator could constitute a risk when taking investigational product.
  - History of medication allergies.
  - Medications and therapies for this current illness taken in the 7 days prior to Day 1, and history of any medication listed in the exclusion criteria within the timelines described below (also see Section 1.1.6, and Section 5.2).
    - Exclusionary medication use includes:
      - Received targeted immune-modulating treatments (such as TNF inhibitors, anti-interleukin-1 [IL-1], anti-IL-6 [tocilizumab or sarilumab], anti-IL-17, T-cell or B-cell targeted therapies (e.g., rituximab), JAK inhibitors including baricitinib, or interferon within 4 weeks or 5 half-lives prior to screening, whichever is longer.
      - Currently receiving corticosteroids at high doses (i.e., prednisone >10 mg per day or equivalent) for >1 month within 2 weeks of screening. Dexamethasone is permitted for the treatment of COVID-19 in patients who are mechanically ventilated and in patients who require supplemental oxygen but who are not mechanically ventilated in accordance with national guidelines.
  - Ask if they are participating in another interventional trial or plan to enroll in another clinical trial in the next 60 days.
- Women of childbearing potential should be counseled to either practice abstinence or use at least one primary form of contraception not including hormonal contraception from the time of screening through Day 60. Women should be confirmed to not be breastfeeding.
  - Note: If a woman is either postmenopausal (i.e., has had ≥12 months of spontaneous amenorrhea) or surgically sterile (i.e., has had a hysterectomy, bilateral ovariectomy (oophorectomy), or bilateral tubal ligation), she is not considered to be of childbearing potential.
- Height and weight (height can be self-reported; weight can be self-reported if not feasible to assess).
- Results of recent radiographic imaging specific to chest (x-ray or CT scan).

- SpO_2, including whether patient is on room air or receiving supplemental oxygen.
- Blood for screening laboratory evaluations if not done as part of routine clinical care in the preceding 48 hours prior to enrollment:
  - CBC with differential
    - Evaluate if neutropenic (absolute neutrophil count <1000 cells/µL) (<1.0 x 10^3/µL or <1.0 GI/L) and/or lymphopenic (absolute lymphocyte count <200 cells/µL) (<0.20 x 10^3/µL or <0.20 GI/L)
  - ALT and AST
    - Assess if ALT or AST >10 times the upper limit of normal.
  - Creatinine (and calculated eGFR)
    - Determine if eGFR <30 mL/min or receiving hemodialysis or hemofiltration.
    - Any automated calculation by the clinical laboratory or published formula for this calculation is acceptable. The site should select a formula to be used for all participants enrolled at the site for the duration of the study.
    - Patients with eGFR <30 mL/min may still enroll in this study (but **not** receive remdesivir) as long as their renal insufficiency has remained stable for at least 1 month without renal replacement therapy (and are not current candidates for renal replacement therapy).
- Urine or serum pregnancy test (in women of childbearing potential).
- Clinical screening laboratory evaluations will be performed locally by the site laboratory. The volume of venous blood to be collected is presented in **Table 8-2**.
- A targeted physical examination will be performed at screening (Day -1 or Day 1) prior to initial study product administration on Day 1. No routine physical exam is needed for study visits after Day 1.
    - Study staff at some sites are not allowed into the participants' rooms due to a limited supply of PPE and the need for strict respiratory isolation measures for COVID-19 patients. Because of limited access to participants, physical exams can be performed by any licensed provider at the study hospital even if they are not study staff listed on the FDA form 1572. The study team can extract information from the hospital chart or EMR.

The overall eligibility of the participant to participate in the study will be assessed once all screening values are available. The screening process can be suspended prior to complete assessment at any time if exclusions are identified by the study team.

8.1.2. *Efficacy Assessments*

For all baseline assessments and follow-up visits, refer to the Schedule of Assessments (SOA) for procedure to be done, and details below for each assessment.

Measures of clinical support, limitations and infection control

The participant's clinical status will be captured on each study day while hospitalized through Day 29. If a participant is discharged early, clinical status is collected on Day 8, Day 11, Day 15, and Day 29 if the participant returns for an in-person clinic visit or by phone if an in-person visit is not possible. Clinical status will also be captured on Day 22 during a phone visit. Clinical status is largely measured by the 8-point ordinal scale and the NEWS. Unlike the NEWS, the ordinal scale can also be evaluated over the phone if the discharged participant is unable to return for visits on Days 8, 11, 15, or 29 as well as on Day 22.

The following measures are recorded for the ordinal scale:

- Hospitalization.
- Oxygen requirement.
- Non-invasive mechanical ventilation (via mask) requirement.
- High flow oxygen requirement.
- Invasive mechanical ventilation (via endotracheal tube or tracheostomy tube) requirement.

- ECMO requirement.
- Ongoing medical in-patient care preventing hospital discharge (COVID-19 related or other medical conditions).
- Limitations of physical activity (self-assessed).
- Isolated for infection control purposes.

Ordinal Scale

The ordinal scale is the basis for the primary and key secondary clinical outcomes in the study, namely, time to recovery, improvement in disease severity, and mortality.

The scale used in this study is as follows (from worst to best):

- Death;
- Hospitalized, on invasive mechanical ventilation or ECMO;
- Hospitalized, on non-invasive ventilation or high flow oxygen devices;
- Hospitalized, requiring supplemental oxygen;
- Hospitalized, not requiring supplemental oxygen – requiring ongoing medical in-patient care (COVID-19 related or otherwise);
- Hospitalized, not requiring supplemental oxygen – no longer requires ongoing medical in-patient care;
  - o This would include those kept in hospital for quarantine/infection control/social reasons, awaiting bed in rehabilitation facility or homecare, etc.
- Not hospitalized, limitation on activities and/or requiring home oxygen;
- Not hospitalized, no limitations on activities.

To determine a participant's clinical status using the ordinal scale: On Day 1, report their clinical status at randomization. After Day 1, collect the ordinal scale daily while hospitalized from Day 2 through Day 29 by providing the worst clinical assessment for the previous day (i.e., midnight to midnight; 00:00 – 23:59 [24-hr clock]). For those who are discharged early, collect ordinal scale on follow-up Days 8, 11, 15, 22, and 29 by providing the worst clinical assessment for the previous day (i.e., midnight to midnight; 00:00 – 23:59 [24-hr clock]). For example, on study Day 3 when completing the form, the worst clinical outcome measure of Day 2 is captured with the worst being death followed by ECMO, mechanical ventilation, etc. The Day 2 measurement is assessed as occurring anytime in that 24-hour period (00:00 to 23:59). For more information about the data collected for the ordinal scale, see the MOP.

Extrapulmonary manifestations

The presence of the extrapulmonary manifestation of disease during the course of hospitalization will be assessed and conditions present at any time during hospitalization will be reported at discharge. Specifically, clinical organ failure defined by development of any one or more of the following clinical events (see Protocol Information Manual) for criteria for what constitutes each of these conditions):

a. Respiratory dysfunction:
   1. Respiratory failure defined as receipt of high flow nasal oxygen, noninvasive ventilation, invasive mechanical ventilation or ECMO

b. Cardiac and vascular dysfunction:
   1. Myocardial infarction
   2. Myocarditis or pericarditis
   3. Congestive heart failure: new onset NYHA class III or IV, or worsening to class III or IV
   4. Hypotension requiring institution of vasopressor therapy

c. Renal dysfunction:
   1. New requirement for renal replacement therapy
   2. New occurrence of eGFR <30 mL/min (or, for participants admitted with baseline eGFR <30 mL/min, an increase in serum creatinine to >1.5 times the baseline value)

d. Hepatic dysfunction:
    1. Hepatic decompensation

e. Neurological dysfunction
    1. Acute delirium
    2. Cerebrovascular event (stroke, cerebrovascular accident [CVA])
    3. Transient ischemic events (i.e., CVA symptomatology resolving <24 hrs)
    4. Encephalitis, meningitis or myelitis

f. Hematological dysfunction:
    1. Disseminated intravascular coagulation
    2. New arterial or venous thromboembolic events, including pulmonary embolism and deep vein thrombosis
    3. Major bleeding events (>2 units of blood within 24 hours, bleeding at a critical site (intracranial, intraspinal, intraocular, pericardial, intraarticular, intramuscular with compartment syndrome, or retroperitoneal), or fatal bleeding).

g. Secondary infection/superinfection:
    1. New or worsening (post-randomization) intercurrent, at least probable, documented serious disease caused by an infection other than SARS-CoV2, requiring antimicrobial administration and care.
    2. A list of secondary infections/superinfections is provided in the study Manual of Operating Procedures (MOP). These are considered adverse events of special interest (AESI).

Longer term follow-up

Information on vital and clinical status and occurrence and duration of re-hospitalization will be captured by phone on Day 60.

8.1.3. *Exploratory Assessments*

Blood for serum, plasma, and whole blood (for future secondary research)
Serum and plasma collected will be stored for future assessment of biomarkers of immune function including cytokines, and chemokines that may be associated with inflammation, anti-viral response, cytokine response syndrome (CRS) and secondary hemophagocytic lymphohistiocytosis (sHLH) during SARS-CoV-2 infection and genetic markers predicting disease progression and/or response to specific therapy. The final decisions about the specific biomarkers will be determined later.

An additional sample of whole blood will be collected at baseline for future genetic testing.

- If the samples can be processed and stored, but not sent to the repository, the samples may be stored locally.
- These samples will be collected at Clinical and Translational Science Awards (CTSA) Program sites. At all other US sites, this collection is optional. Samples will not be collected at non-US sites.
- Samples may be collected at the time of routine phlebotomy.
- See Section 8.2, Table 8-2 (Venipuncture Volumes) for the schedule of blood collections.

National Early Warning Score (NEWS)
Vital signs and other clinical assessments are collected for the calculation of the NEWS, and include temperature, systolic blood pressure, heart rate, respiratory rate, O_2 saturation and level of consciousness. Vital signs collected per standard of care can be used. NEWS has demonstrated an ability to discriminate subjects at risk of poor outcomes. (Smith, 2016). This score is based on 7 clinical parameters (see **Table 8-1**). The NEWS is being used as an exploratory efficacy measure. The components of NEWS should be evaluated daily while hospitalized. It can be performed concurrently with the Ordinal Scale. This should be evaluated at a consistent time for each study day and

prior to administration of study product. The 7 parameters can be obtained from the hospital chart or electronic medical record (EMR) using the last measurement prior to the time of assessment (including parameters collected prior to the time of consent) and a numeric score is given for each parameter (e.g., a RR of 9 breaths per minute is one point, oxygen saturation of 92% is two points). This is recorded for the day obtained (i.e., on Day 3, the vital signs and other parameters from Day 3 will be used to obtain NEWS for Day 3). ECMO and mechanically ventilated participants should be assigned a score of 3 for RR (RR ≤8 breaths per minute) regardless of the ventilator setting. Participants on ECMO should get a score of 3 for heart rate since they are on cardiopulmonary bypass.

Table 8-1. National Early Warning Score (NEWS)

PHYSIOLOGICAL PARAMETERS	3	2	1	0	1	2	3
Respiration Rate (breaths per minute)	≤8 [a]		9 – 11	12 – 20		21 – 24	≥25
Oxygen Saturation (%)	≤91	92 – 93	94 – 95	≥96			
Any Supplemental Oxygen		Yes		No			
Temperature (°C)	≤35.0		35.1 – 36.0	36.1 – 38.0	38.1 – 39.0	≥39.1	
Systolic Blood Pressure (mm Hg)	≤90	91 – 100	101 – 110	111 – 219			≥220
Heart Rate (beats per minute)	≤40 [b]		41 – 50	51 – 90	91 – 110	111 – 130	≥131
Level of Consciousness				A			V, P, U

Level of consciousness = alert (A), and non-alert and arousable only to voice (V) or pain (P), and unresponsive (U).
[a] If the participant is on ECMO or invasive mechanical ventilation, they will be given a score of 3 (≤8 breaths per minute) for respiratory rate regardless of ventilator setting.
[b] Participants on ECMO will also receive a score of 3 (≤40 beats per minute) for heart rate.

8.2. Safety and Other Assessments

Study procedures are specified in the SOA. A study physician licensed to make medical diagnoses and listed on the FDA form 1572 will be responsible for all trial-related medical decisions.

Clinical laboratory evaluations:

- Fasting is not required before collection of laboratory samples.
- Blood will be collected at the time points indicated in the SOA and in Table 8-2.
- Clinical safety laboratory tests will evaluate the following parameters at the following time points:
    - Screening: CBC with differential (including absolute neutrophil count and absolute lymphocyte count), ALT, AST, ALP, total bilirubin, and creatinine (with calculated eGFR).

    - Baseline: CBC with differential (including absolute neutrophil count and absolute lymphocyte count), ALT, AST, ALP, total bilirubin, creatinine (with calculated eGFR), glucose, PT/INR, troponin, d-dimer, and C-reactive protein. Sites that do not have access to a test for PT will be allowed to report an international normalized ratio (INR).
    - Days 3, 5, 8, 11, 15, and 29: WBC with differential, hemoglobin, platelets, ALT, AST, ALP, total bilirubin, creatinine (with calculated eGFR), and glucose.
        - On Day 8 (or on day of discharge if prior to Day 8), the following **additional** parameters will be assessed: PT/INR, troponin, d-dimer, and C-reactive protein. Sites that do not have access to a test for PT will be allowed to report an international normalized ratio (INR).
- Routine standard-of-care clinical laboratory bloodwork may be accepted for Screening if performed within the 48 hours prior to enrollment.
- Baseline (Day 1) clinical laboratory evaluations are drawn prior to initial study product administration as a baseline and results do not need to be reviewed to determine if initial study product administration should be given.
    - Laboratory tests performed as part of routine clinical care in the 48 hours prior to baseline visit will be accepted for the baseline safety laboratory tests.
    - Note that Day 1 PK assessments need to be drawn after 1st dose of study drug, so will need to be drawn separately from Baseline lab assessments.
- Clinical laboratory testing will be performed at each clinical trial site in real time. PK samples will be analyzed at a central facility at a future date.

Table 8-2. Venipuncture Volumes

	Screen	***Baseline***						
**Day (± Window)**	**−1 to 1**	**1 (± 1)**	**3 (± 1)**	**5 (± 1)**	**8 (± 1)**	**11 (± 1)**	**15 (± 2)**	**29 (+ 3)**
Safety hematology, chemistry and liver tests [1]	X 10mL	X 10mL	X 10mL	X 10mL	X [2] 10mL	X [2] 10mL	X [2] 10mL	X [2] 10mL
Blood for Serum and Plasma (future and secondary research) [3]		X [3] 18mL	X [3] 18mL		X [3] 18mL		X [3] 18mL	X [3] 18mL
Blood for Whole Blood (genetic testing) [3]		X [3] 8mL						
PK Assessments [4]		X [5] 4mL			X [6] 4mL			X [6] 4mL
Total daily volume [7]	10mL	40mL (14mL)	28mL (10mL)	10mL	32mL (14mL)	10mL	28mL (10mL)	32mL (14mL)
Total all study days [7]								190 mL (92mL)

PK = pharmacokinetic

1. Routine clinical laboratory bloodwork may substitute for any post-baseline blood draws if drawn within the given window for the study days. At Screening and Baseline, routine clinical laboratory bloodwork may be accepted if performed within 48 hours prior to enrollment (Screening) or baseline visit (Baseline).

2. Safety laboratory tests will be collected on Days 8, 11, 15, and 29 if the participant is still hospitalized at these time points or if they return for an in-person outpatient visit and the site has the capacity to collect blood in the outpatient setting.

3. Blood collection for future secondary research, including genetic testing, is to be performed at CTSA sites; collection is optional at all other US sites. Ex-US sites will not collect blood for future secondary research.
4. All efforts will be made to collect the PK samples. If the samples are not collected due to complication or collected outside the window, this will not be considered a deviation. See study Manual of Operating Procedures (MOP) and sub-study specific appendices for specific PK blood collection procedures. Participants on ECMO may have an additional blood draw at the given PK timepoint; please consult the MOP.
5. Single blood draw, separate from Day 1 Baseline labs, within 24 hours post-dose (post-start of infusion or post-ingestion of oral drug), with timing of drug administration and PK collection recorded. In patients that received an infusion, sample should be collected from the arm contralateral to that used for IV infusion.
6. If hospital discharge occurs prior to visit, then the PK blood sample should be collected at discharge; this sample may be drawn at the time of routine phlebotomy.
*7. Values in parentheses indicate total volume of blood if blood for future secondary research (including genetic testing) is **not** collected.*

Additional PK blood collections from participants on ECMO:

ECMO has been repeatedly shown to substantially and unpredictably alter drug disposition (Wildschut et al. 2010; Watt et al. 2011). For ACTIV-1 IM participants supported with ECMO, the PK sampling scheme depicted in **Table 8-2** will be augmented with a paired sample collected from the ECMO circuit. Please consult the study Manual of Operating Procedures (MOP).

8.2.1. *Procedures to be Followed in the Event of Abnormal Laboratory Test Values or Abnormal Clinical Findings*

If a physiologic parameter (e.g., vital signs, or laboratory value) is outside of the protocol-specified range, then the measurement may be repeated once if, in the judgment of the investigator, the abnormality is the result of an acute, short-term, rapidly reversible condition or was an error. A physiologic parameter may also be repeated if there is a technical problem with the measurement caused by malfunctioning or an inappropriate measuring device (i.e., inappropriate-sized BP cuff).

8.2.2. *Unscheduled Visits*

If clinical considerations require the participant to be contacted or seen prior to the next schedule assessment to assure the participant's well-being, it is permissible in this protocol. However, no research data is collected at this visit.

8.3. Adverse Events and Serious Adverse Events

8.3.1. *Definition of Adverse Event (AE)*

AE means any untoward medical occurrence associated with the use of an intervention in humans, whether or not considered intervention related. An AE can therefore be any unfavorable and unintended sign (including an abnormal laboratory finding), symptom, or disease temporally associated with the use of medicinal (investigational) product. If multiple abnormalities are part of the same clinical syndrome, they can be reported together as one AE under a unifying clinical diagnosis.

Any medical condition that is present at the time that the participant is screened will be considered as baseline and not reported as an AE. However, if the severity of any pre-existing (baseline) medical condition increases above baseline to severity grade 3 or 4, it should be recorded as an AE.

Given the nature of severity of the underlying illness, participants will have many symptoms and abnormalities in vital signs and laboratory values. All Grade 3 and 4 AEs will be reported in this trial. In addition, the following AEs will be reported:

- Any Grade 2 suspected drug-related hypersensitivity reactions associated with study product administration will be reported as an AE.
- Any newly diagnosed infection (other than COVID-19) at any time during the study.
- Any AEs leading to dose modification
- Any AEs leading to discontinuation from the study.

Intermittent abnormal laboratory values or vital sign measurements common in the severely ill populations (such as electrolyte abnormalities, low blood pressure, hyperglycemia, etc.) that are part of the same clinical diagnosis (e.g., uncontrolled diabetic) can be recorded once with the worst grade for each adverse event (grade 3 and 4 only for this trial), with the start and stops dates of the intermittent syndrome. If there is clear resolution of the event, and then recurrence, it should be treated as a separate adverse event. Resolution is defined as return to baseline (either normal if was normal at Day 1, or baseline (Day 1) grade if already an abnormality on the toxicity table at Day 1) for > 48 hours.

Serious or acute syndromes that arise during the course of the study should be treated and referred as necessary according to the standard of care prevailing at the local treatment site.

8.3.2. *Definition of Serious Adverse Event (SAE)*

An AE or suspected adverse reaction is considered serious (i.e., is an SAE) if, in the view of either the investigator or the Sponsor, it results in any of the following outcomes:

- Death;
- A life-threatening AE;
- Inpatient hospitalization or prolongation of existing hospitalization;
- A persistent or significant incapacity or substantial disruption of the ability to conduct normal life functions; or
- A congenital anomaly/birth defect.

Grade 4 AEs (potentially life-threatening events) are not always SAEs unless they are imminently life threatening.

Important medical events that may not meet the above criteria may be considered serious when, based upon appropriate medical judgment, they may jeopardize the participant and may require medical or surgical intervention to prevent one of the outcomes listed in this definition. Examples of such medical events include allergic bronchospasm requiring intensive treatment in an emergency room or at home, blood dyscrasias, convulsions that do not result in inpatient hospitalization, or the development of drug dependency or drug abuse.

“Life-threatening” refers to an AE that at occurrence represents an immediate risk of death to a participant. An event that may cause death if it occurs in a more severe form is not considered life-threatening. Similarly, a hospital admission for an elective procedure is not considered a SAE.

All SAEs, as with any AE, will be assessed for severity and relationship to study intervention.

All SAEs will be recorded on the AE CRF.

SAEs will only be reported to the designated pharmacovigilance group (see Section 8.3.6 for reporting procedure) if they are considered related to the study product (serious adverse reaction [SAR]).

All SAEs will be followed through resolution or stabilization by a licensed study physician (for IND studies, a physician listed on the Form FDA 1572 as the site PI or Sub-Investigator).

All SAEs will be reviewed and evaluated by the IND sponsor and will be sent to the DSMB (for periodic review), and the IRB/IEC. All newly-diagnosed secondary infections/superinfections are regarded as adverse events of special interest (AESI) and will be reported to the DSMB.

8.3.3. *Suspected Unexpected Serious Adverse Reactions (SUSAR)*

A SUSAR is any SAE where a causal relationship with the study product is at least reasonably possible but is not listed in the Investigator Brochure (IB), Package Insert, and/or Summary of Product Characteristics.

8.3.4. *Classification of an Adverse Event*

The determination of seriousness, severity, and causality will be made by an on-site investigator who is qualified (licensed) to diagnose AE information, provide a medical evaluation of AEs, and classify AEs based upon medical judgment. This includes but is not limited to physicians, physician assistants, and nurse practitioners.

Severity of Adverse Events

All AEs and SAEs will be assessed for severity using the DAIDS Table for Grading the Severity of Adult and Pediatric Adverse Events, version 2.1 (July 2017).

For AEs not included in the Table, the following guidelines will be used to describe severity. In addition, all deaths related to an AE are to be classified as grade 5 according to the DAIDS Table.

- Moderate (Grade 2): Events that are usually alleviated with additional specific therapeutic intervention. The event interferes with usual activities of daily living and causes discomfort but poses no significant or permanent risk of harm to the research participant.
- Severe (Grade 3): Events that interrupt usual activities of daily living, or significantly affect clinical status, or may require intensive therapeutic intervention. Severe events are usually incapacitating.
- Potentially life-threatening event (Grade 4): Events that are potentially life threatening.
- Deaths (Grade 5): All deaths related to an AE are classified as grade 5 (per DAIDS Table).

Relationship to Study Intervention

For each reported adverse reaction, the PI or designee must assess the relationship of the event to the study product using the following guideline:

- Related – There is a temporal relationship between the study intervention and event, and the AE is known to occur with the study intervention or there is a reasonable possibility that the study intervention caused the AE. Reasonable possibility means that there is evidence to suggest a causal relationship between the study intervention and the AE.
- Not Related – There is not a reasonable possibility that the administration of the study intervention caused the event, there is no temporal relationship between the study intervention and event onset, or an alternate etiology has been established.

8.3.5. *Time Period and Frequency for Event Assessment and Follow-Up*

For this study, all Grade 3 and 4 AEs and all SAEs occurring from the time the informed consent is signed through the Day 60 ± 5-day telehealth visit will be documented, recorded, and reported. In addition, patients will be contacted at the end of the safety follow-up period required for each study agent (see appendices for details) to assess AEs and SAEs occurring after drug withdrawal.

Investigator Reporting of AEs

Information on AEs will be recorded on the appropriate CRF. All clearly related signs, symptoms, and results of diagnostic procedures performed because of an AE should be grouped together and recorded as a single diagnosis. New secondary infections/superinfections will be recorded on the appropriate CRF. If the AE is a laboratory abnormality that is part of a clinical condition or syndrome, it should be recorded as the syndrome or diagnosis rather than the individual laboratory abnormality. Each AE will also be described in terms of duration (start and stop date), severity, association with the study product, action(s) taken, and outcome.

8.3.6. *Serious Adverse Event Reporting*

Investigator Reporting of SAEs

Any AE that meets a protocol-defined criterion as a treatment related SAE (SAR) must be submitted within 24 hours of site awareness via an SAE form to the Technical Resources, International (TRI) Pharmacovigilance Group, at the following address: TRISafetyOffice@tech-res.com.

Other supporting documentation of the event may be requested by the TRI Pharmacovigilance Group and should be provided as soon as possible. The TRI Medical Monitor will review and assess the SAE for regulatory reporting and potential impact on study participant safety and protocol conduct.

At any time after completion of the study, if the site PI or appropriate sub-investigator becomes aware of an SAE that occurred during the participant's participation in the study, the site PI or appropriate sub-investigator will report the event to the TRI Pharmacovigilance Group.

Regulatory Reporting of SAEs

Any event that requires expedited reporting to Regulatory Authorities (i.e, Serious Unexpected Suspected Adverse Reactions [SUSARs]) based on applicable national regulations will be forwarded to the IND sponsor in time to meet reporting requirements (e.g. 7 days for fatal and life-threatening initial reports, with follow up reports within another 8 days, 15 days for all other SUSARs). The IND sponsor or its in-country representative as detailed in the Transfer of Regulatory Obligations (TORO) will submit safety reports (e.g. IND safety reports) to the regulatory agencies as necessary, and will inform the investigators of such regulatory reports. Site investigators must submit safety reports as required by their Institutional Review Board (IRB)/Ethics Committee (EC). Documentation of the submission and receipt by the IRB/REB must be retained for each expedited safety report.

SAEs that are not SUSARs will be reported to the FDA at least annually in a summary format which includes all SAEs.

Sites may have additional local reporting requirements (to the IRB/EC and/or national regulatory authority).

8.3.7. *Reporting of Pregnancy*

Pregnancy is not an AE. However, any pregnancy that occurs in a study subject during study participation should be reported to the Sponsor on the appropriate CRF. Pregnancies should also be reported to the IRB/EC. The Sponsor will report pregnancies to the DSMB. Pregnancy should be followed to outcome.

Pregnancy should also be reported in a partner of a male participant, though information may not be collected from the pregnant partner.

8.4. Unanticipated Problems

8.4.1. *Definition of Unanticipated Problems*

An Unanticipated Problem (UP) is any event, incident, experience, or outcome that meets the following criteria:

- Unexpected in terms of nature, severity, or frequency given (a) the research procedures that are described in the protocol-related documents, such as the IRB-approved research protocol and informed consent document; and (b) the characteristics of the subject population being studied;
- Related to participation in the research (meaning there is a reasonable possibility that the incident, experience, or outcome may have been caused by the procedures involved in the research); and
- Suggests that the research places participants or others at a greater risk of harm (including physical, psychological, economic, or social harm) than was previously known or recognized.

8.4.2. *Unanticipated Problem Reporting*

To satisfy the requirement for prompt reporting, all UPs will be reported using the following timeline:

- UPs that are SAEs will be reported to the IRB/EC and to the Statistical and Data Coordinating Center (SDCC)/study Sponsor within 24 hours of the investigator becoming aware of the event per the above described SAE reporting process.
- Any other UP will be reported to the IRB/EC and to the SDCC/study Sponsor within 3 days of the investigator becoming aware of the problem.

9. STATISTICAL CONSIDERATIONS

The ACTIV-1 IM master protocol utilizes an adaptive platform trial design that allows for four types of adaptations: (1) early stopping of agents for futility, to allow more speedy accrual to other agents remaining in the trial; (2) early stopping for strong evidence of efficacy of a particular agent; (3) substitution of one of the test agents as standard of care for the remainder of the trial, should early evidence of superiority over SoC support such a change, and (4) addition of newly emergent agents, if they become available after study start. A brief summary is provided here. Details will be described in the statistical analysis plan (SAP).

The total sample size for ACTIV-1 IM is based on the Kaplan-Meier estimate of the probability of recovery from ACTT-1. Preliminary data indicate this estimate is approximately 73% based on over 1,000 patients enrolled.

Sample size requirements are based on the number of agents being evaluated and the ability to pool control patients for analysis. Initial sample size estimates are derived assuming three agents are ready for testing at study start and will remain in the study for evaluation at the final analysis stage. If newly emergent therapies are entered into the master protocol after study start, sample size requirements will be adjusted accordingly.

The statistical analysis strategy calls for sharing of control participants across all agents available for study when enrollment begins. For agents added to the trial after study start, comparative analyses will be limited to those control patients enrolled concurrently with the new agent. That is, control participants enrolled prior to an agent entering the trial will not be included in comparative analyses of that agent. This approach was used in the recent PALM study in patients with Ebola virus disease (Mulangu et al. 2019). Although sharing of non-concurrently randomized control patients has been used successfully in studies of chronic diseases (DIAN-TU; https://clinicaltrials.gov/ct2/show/NCT01760005), it is not recommended here due to the evolving nature of the COVID-19 infection and treatment thereof. Exploratory analyses of the similarity between control participants enrolled early and late in the study, however, may be useful in informing the design of future master protocols in COVID-19.

If early evidence of superiority of any one of the test agents relative to SoC (plus placebo) is observed during this trial, consideration will be given to replacing the SoC with the test agent for the remainder of the trial. Such a change is dependent on the safety of combining the remaining test agents with this new SoC and will complicate the final data analyses somewhat. Comparative analyses will need to account for this change in SoC through stratification or other model-based methods. Details will be provided in the statistical analysis plan.

Preliminary reports of the completed RECOVERY Trial show that dexamethasone may reduce mortality in some hospitalized patients with COVID-19 (RECOVERY Collaborative Group, 2020). If dexamethasone or steroid, or other agents, become part of standard of care (SoC) or are used in a substantial number of patients, the observed potential benefit of immunomodulators may be altered. Nonetheless, ACTIV-1 IM is designed with high power to detect even a modest benefit of 25% for the primary outcome; attempting to detect smaller effects would cause a prohibitively large increase in sample size. Addition of dexamethasone or other agents to SoC may also affect the key secondary endpoint of mortality and alter the proportions of people in different cells of another key secondary endpoint, the 8-point ordinal scale. Accordingly, a blinded sample size re-estimation may be conducted at the 2nd interim analysis, after approximately one-half of the total number of expected recoveries has occurred. The purpose is to use the overall proportions of people in the different categories of the key secondary endpoint of the ordinal scale to recalculate power for that endpoint. If power for this key secondary endpoint is below 70%, the sample size

might be increased, but by at most 25%. The sample size would not be reduced based on this potential re-estimation. More details will be provided in the SAP.

9.1. Statistical Hypotheses

The primary null hypothesis being tested is that time-to-recovery does not differ between each test agent and the pooled control group, consisting of patients receiving SoC plus placebo. The alternative hypothesis is that the test agent and pooled control group differ in time-to-recovery. If Drug A, B, and C denote the initial set of agents to be tested in ACTIV-1 IM, and S denotes patients receiving SoC plus placebo, then the primary hypotheses are:

H_{01}: $TR_A = TR_S$ vs H_{A1}: $TR_A \neq TR_S$

H_{02}: $TR_B = TR_S$ vs H_{A2}: $TR_B \neq TR_S$

H_{03}: $TR_C = TR_S$ vs H_{A3}: $TR_C \neq TR_S$

There are two key secondary endpoints that will be evaluated as supportive evidence:

1. The distribution of the 8-point ordinal scale at Day 14 and Day 28 (assessed on Days 15 and 29 for the previous day). For this, the parameter of interest is the "common odds ratio," which quantifies the shift in the severity distribution resulting from treatment. For an efficacious treatment, an odds ratio greater than 1 quantifies an improvement in disease severity; a value of 2 indicates a bigger improvement than a value of 1.25. The null hypothesis to be tested is that the odds of improvement on the ordinal scale is the same for the placebo and experimental treatment arms (i.e., the common odds ratio is 1). It is worth noting that, for large sample sizes, the test based on the proportional odds model is nearly the same as the Wilcoxon rank sum test.
2. Mortality will be evaluated with standard survival analysis techniques. Kaplan Meier curves and associated log-rank statistics will be generated to compare mortality between each test agent and SoC. Mortality (14-day and 28-day) will also be evaluated at Days 15 and 29.

9.2. Sample Size Determination

Primary endpoint: The Fine-Gray approach will be used to compare each test agent with SoC with respect to the cumulative incidence of recovery, accounting for the competing risk of mortality (Fine and Gray, 1999). The approach is similar to using a log-rank test on time to recovery, retaining in the risk set people who die. The two key determinants of power are the total number of events (i.e., recoveries) E and the treatment-to-control ratio of the rate of recovery, RRR. Without accounting for futility monitoring, the number of events required for power $1-\beta$ to detect a recovery rate ratio of θ using a two-tailed test at alpha=0.05 is approximately

$$E = \frac{4\left(1.96 + z_\beta\right)^2}{\{\ln(RRR)\}^2},$$

where z_β is the $100(1-\beta)$th percentile of the standard normal distribution. The number of events must be increased to account for futility monitoring. For 85% power, the $1.96 + z_\beta$ in the above equation is replaced by 3.1312.

Table 9-1 provides the numbers of recoveries and of patients required to provide 85% power for a single pairwise comparison of test drug versus control assuming a 73% recovery rate and various recovery rate ratios (RRRs). Note that the rate of recovery is the analogue of the hazard for each test agent or control treatment, and the RRR is the analogue of the hazard ratio for a test agent relative to control in this setting. As can be seen from the table, approximately 347 recoveries are required to detect a 40% increase in the rate of recovery ($\theta = 1.40$) from control. An RRR of 1.40 is similar to, but slightly higher than the figure of 1.31 reported in Cao et al. (2020) for a lopinavir/ritonavir trial that used time to improvement by 2 categories as primary endpoint. A total of 436 recoveries is needed for an RRR of 1.35 with 85% power. Sample size requirements for ACTT-1 assumed an RRR of 1.35, but

the study was over-enrolled (1,063 patients were enrolled, compared to N=572 needed) due to more rapid than anticipated accrual. Preliminary results from ACTT-1 show an RRR of 1.32 ([CI = [1.12,1.55]) for remdesivir vs placebo.

Table 9-1. Number of recoveries and number of patients needed for 85% power assuming a type I error rate of 5% for various recovery ratios

Recovery rate ratio (θ)	Number of recoveries needed for 85% power	Sample Size assuming 73% recovery rate (1 test agent vs SoC)
1.25	**788**	**1,080**
1.30	570	781
1.35	436	598
1.40	347	476

The objective of ACTIV-1 IM is to provide evidence of efficacy and safety of selected agents that would support regulatory approval of their use in treating moderately to severely ill hospitalized Covid-19 patients. It was decided that a modest effect as low as a 25% improvement in recovery rate attributable to any one of the test agents in the study would be of interest to detect. Consequently, the sample size requirements are based on the ability to detect an RRR of 1.25 for each agent relative to SoC.

A total of 788 recoveries is required for each comparison to provide approximately 85% power to detect a recovery rate ratio of 1.25 for the therapeutic agent relative to control, accounting for the interim analyses. Assuming 73% of participants achieve recovery in 28 days, consistent with the ACTT-1 results, the total sample size to compare 1, 2, or 3 agents to control in ACTIV-1 IM is approximately 1,080, 1,620, and 2,160, respectively. Because each agent is being compared to SoC with no between-agent comparisons currently planned, no multiplicity adjustments will be made, provided the number of agents assessed in ACTIV-1 IM is three or fewer. If more than three agents are evaluated, a minor multiplicity adjustment will be considered to aid in interpreting the results of the study.

Key secondary endpoints: A sample size can be computed using an (assumed) ordinal scale distribution for the control and the odds ratio representing clinical improvement. The odds ratio represents the odds of improvement in the ordinal scale for treatment relative to control [Whitehead, 1993]. The sample size to detect a given odds ratio for 1:1 randomization using a 2-tailed test at level α is given by

$$\frac{12\left(z_{\alpha/2}+z_{\beta}\right)^2}{\lambda^2\left(1-\sum_{i=1}^{K} p_i^3\right)},$$

where λ is the log odds ratio, p_i is the overall probability (combined over both arms) of being in the ith category of the K ordinal outcomes, and $z_{\alpha/2}$ and z_{β} are the $1-\alpha/2$ and $1-\beta$ quantiles of the standard normal distribution.

A sample size of 1,080 for an (active, control) pair gives approximately 95% or 85% power to detect an odds ratio of 1.50 (the observed odds ratio in the ACTT-1 trial) or 1.40 using a 2-tailed test at level $\alpha = 0.05$.

The 28-day mortality probability in the remdesivir arm of ACTT-1 was approximately 12%. Power for comparing 28-day mortality is 90% only for a 50% relative reduction (from 12% to 6%).

9.3. Populations for Analyses

The primary analysis will be based on an intention-to-treat population, including all participants randomized. Similarly, safety analyses will be based on a modified intention-to-treat population consisting of all participants who received at least one dose or injection of each drug administered in the randomization arm (e.g., test agent plus SoC). The primary analysis will be based on those participants enrolled in order to achieve 788 recoveries for each pairwise comparison as noted in section 9.1. Subsequent analysis will be performed on all enrolled participants.

9.4. Statistical Analyses

9.4.1. *General Approach*

This is a randomized, controlled, double-blinded trial testing a superiority hypothesis with respect to each therapeutic agent plus SoC versus SoC plus placebo with a two-sided Type I error probability of 5% for each agent. This is a randomized, controlled, double-blinded trial testing a superiority hypothesis with respect to each therapeutic agent plus SoC versus SoC plus placebo with a two-sided Type I error probability of 5% for each agent. Secondary hypotheses have been ordered according to relative importance, with key secondary hypotheses identified (corresponding to the odds ratio based on the 8-point clinical severity scale, and time to death). These will be described according to the appropriate summary statistics (e.g., proportions for categorical data, median for time-to-event data).

A statistical analysis plan will be developed and filed with the FDA prior to the first unblinded interim analysis.

Unblinding of study data for final analysis will occur for each test agent independently of other agents, consistent with the master protocol design. That is, once the planned number of recoveries required for a particular agent's comparative analysis are observed, study close-out procedures for that agent are applied for all study visits and data elements associated with the participants receiving the test agent as well as participants receiving SoC plus placebo during the randomization period for that agent (i.e., concurrently controlled participants). The study visits are monitored, data edits are completed, queries are resolved, database is locked, and treatment assignments are unmasked for that comparative analysis only. Note that, because control participant data are shared across agents, procedures for unmasking data for one agent's final analysis will be established to protect the ability to continue the ongoing study in a double-blinded manner for the remaining agents. It may be that recovery rates are similar enough across all agents in the study to enable a single study close-out with standard procedures. Any necessary modifications or updates to the statistical analysis should be made prior to the study unblinding.

9.4.2. *Analysis of the Primary Efficacy Endpoint*

The primary efficacy analysis for the comparison of each test agent plus SoC versus SoC plus placebo is a stratified test based on the Fine-Gray proportional hazards approach, where stratification is according to region and baseline disease severity (i.e. protocol defined mild/moderate vs severe disease). The method provides an estimate of the cumulative incidence of recovery while accounting for the competing risk of death. The hazard ratio can also be computed. With no censoring, the hazard rate in each arm can be thought of as the hazard for recovery, treating deaths as never having recovered (see section 2 of Fine and Gray, 1999). Every attempt will be made to complete final clinic visits for patients dropping out of the study prematurely. For those unable to complete the final visit, data collection will be attempted via telephone interview. At a minimum, vital status will be obtained.

9.4.3. *Analysis of the Secondary Endpoint(s)*

1) The ordinal scale will be used to estimate a proportional odds model by disease strata. We will perform a stratified test to evaluate whether the common odds ratio for treatment is equal to one. The distribution of severity results will be summarized by treatment arm as percentages. Efforts to minimize loss-to-follow-up will be considerable. However, small amounts of missing data may occur. In such cases, participants without final outcome data will be excluded from the analysis. Sensitivity analyses will evaluate the impact of making different assumptions about missing observations. These analyses will be defined in the SAP.

2) When death is not a competing risk (for example, the endpoint includes death in the composite), differences in time-to-event endpoints by treatment will be summarized with Kaplan-Meier curves and 95% confidence bounds. When death is a competing risk (for example, time to at least a one-category improvement in ordinal scale, and time to at least a two-category improvement), the same competing risk approach will be used as for the primary analysis.

3) Change in ordinal scale at specific time points will be summarized by proportions (e.g., proportion who have a 1-, 2-, 3-, or 4-point improvement or 1-, 2-, 3-, 4-point worsening).

4) Differences in total severity score (TSS) over time will assessed by fitting a longitudinal mixed model to the 8-point clinical scale value on each day of assessment (in hospital plus Days 15, 22, 29). The average

difference across days in study (analogous to the area under the severity curve) will be estimated with 95% confidence limits in the context of the longitudinal model.

5) Duration of event (e.g., duration of mechanical ventilation) will be summarized according to median days with quartiles.

6) Binary data (e.g., incidence of new oxygen use) will be summarized as a percent with 95% confidence intervals. Comparisons between arms will be presented as differences in proportions with 95% confidence intervals.

7) Categorical data (e.g., 28-day mortality or ordinal scale by day) may be summarized according to proportions by category and/or odds ratios with confidence intervals.

Procedures for handling missing data, including informative censoring (e.g., a missing duration of oxygen use endpoint due to a death), will be described in the statistical analysis plan.

9.4.4. *Safety Analyses*

Safety endpoints include death through Day 28 (as assessed at Day 29), SAEs, and Grade 3 and 4 AEs. These events will be analyzed univariately and as a composite endpoint. Time-to-event methods will be used for death and the composite endpoint. Each AE will be counted once for a given participant and graded by severity and relationship to COVID-19 or study intervention. AEs will be coded using the current version of the Medical Dictionary for Regulatory Activities (MedDRA). AEs will be presented by system organ class, duration (in days), start- and stop-date. Adverse events leading to premature discontinuation from the study intervention and serious AEs will be presented either in a table or a listing.

9.4.5. *Baseline Descriptive Statistics*

Baseline characteristics will be summarized by test agent versus control for each agent. For continuous measures the mean and standard deviation will be summarized. Categorical variables will be described by the proportion in each category (with the corresponding sample size numbers).

9.4.6. *Planned Early and Interim Analyses*

Data analyses will be conducted to monitor enrollment and follow-up rates and to summarize baseline characteristics throughout the course of the study. These early analyses will be conducted by the study team masked to treatment assignment. Summaries will be generated for each test agent separately. For agents entering the study at staggered times, data summaries (pooling test agent and control data in blinded fashion) will incorporate data from concurrently randomized control participants only.

Unblinded interim analyses are planned to (i) assess the futility of each agent, with the goal of discontinuing those with lower probabilities of success to more effectively utilize trial resources for the remaining agents and (iii) review comparative analyses for each test agent to assess early stopping for efficacy. Alpha spending functions will be used to appropriately control the probability of making an erroneous conclusion across interim and final analyses at $\alpha = 0.05$ (two-sided) for each agent.

A Data and Safety Monitoring Board (DSMB) will monitor ongoing results to ensure participant well-being and safety as well as study integrity. The DSMB will be asked to recommend early termination due to futility and early stopping for efficacy, only when stopping rules and conditions are clearly met. More details about the interim analyses are described in section 9.4.6.1 and 9.4.6.2 below as well as a separate guidance document for the DSMB.

Interim Safety Analyses

Safety analyses will evaluate Grade 3 and 4 AE and SAEs by treatment arm. Safety monitoring will be ongoing. The unblinded statistical team will prepare these reports for review by the DSMB.

Interim Analyses for Futility and Efficacy Review

Interim analyses for futility and efficacy are planned at three times during the study corresponding to the availability of approximately 25%, 50%, and 75% of total information. The planned randomization algorithm described above

(Section 6.2) ensures equal allocation to each test agent or control (e.g., 1:1:1:1 for 3 test agents and control). It is anticipated that the interim analyses for each agent will occur at the same time, and the DSMB will make recommendations for all agents at their scheduled meetings. If recovery rates vary substantially by agent, however, it may be necessary to let the interim analysis times also vary by agent. Because Type I error probabilities are controlled for each, independently of the other agents, the need for additional DSMB reviews due to differential information accrual across agents should not pose any issues other than logistical ones.

The Lan-DeMets spending function analog of the O'Brien-Fleming boundaries will be used to monitor the primary endpoint as a guide for the DSMB. This spending function is conservative in that priority is given to preserving power for the final analysis with the use of stringent stopping rules early in the study.

In contrast, moderately aggressive stopping rules for futility will be implemented to promote early discontinuation of agents with low probabilities for success. The futility stopping rules will be considered non-binding by the DSMB in their review of interim data. The futility boundaries are computed based on the gamma family of spending functions $\alpha\{1-\exp(-\gamma t)\}/\{1-\exp(-\gamma)\}$ (Hwang et al. 1990). Figure 1 below illustrates the efficacy stopping boundaries based on the Lan-DeMets spending function (in blue shading) and the futility boundaries based on a Gamma (-2) spending function (in pink shading).

Figure 1: **Stopping Boundaries for Efficacy (blue shading) and Futility (pink shading).**

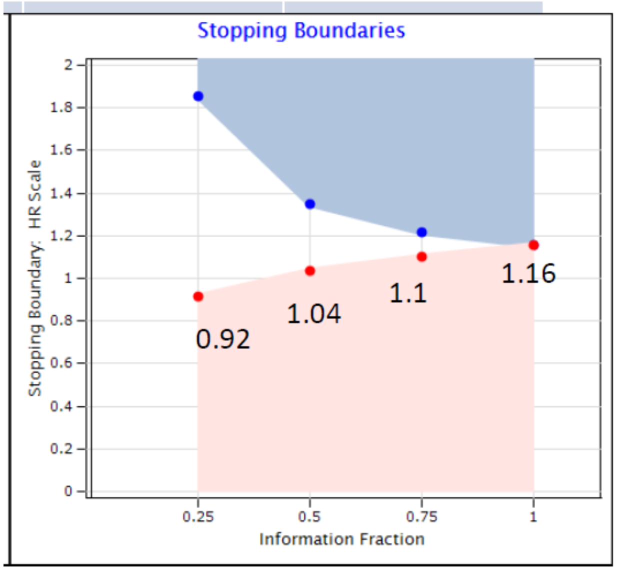

As can be seen from the figure, an RRR showing no more than a small amount of improvement in recovery time (RRR ≤ 1.04) will result in early discontinuation of the agent mid-way through information accrual in the study. When the null hypothesis is true (RRR = 1), the probability of discontinuing an agent at this point is 66%, but under the alternative, this probability is 4%. In simulations, the average number of recoveries under these efficacy and futility boundaries is 587, and the maximum sample size is approximately 1,080 participants.

The unblinded statistical team will prepare closed reports for each DSMB review meeting. Analyses will be presented with blinded codes for treatment arms to protect against the possibility that the DSMB report may fall into the wrong hands. Unblinding codes will be provided to DSMB members who wish to be unblinded. A DSMB charter will further describe procedures and membership. An additional document on statistical issues related to monitoring will be provided to the DSMB prior to the first interim analysis.

9.4.7. *Sub-Group Analyses*

Subgroup analyses for the primary outcomes will evaluate treatment effects across the following subgroups: geographic region, duration of symptoms prior to enrollment, baseline disease severity (stratification variable of mild/moderate and severe, as well as ordinal scale of 4/5 vs 6/7) age, race, sex and comorbidities. A forest plot will

display confidence intervals across subgroups. Interaction tests will be conducted to determine whether the effect of treatment varies by subgroup. These subgroup analyses will be carried out separately for each test agent.

9.4.8. *Exploratory Analyses*

An exploratory analysis will compare treatment efficacy estimates for each agent according to the various scales outlined in section 8.1.3. Specifically, the probability of falling into category "i" or better will be compared between arms for each i. Exploratory analyses will also compare the difference between each test agent plus SoC and SoC plus placebo in TSS at each study day for which the clinical scale is administered (in-hospital and at Days 15, 22, and 29).

10. SUPPORTING DOCUMENTATION AND OPERATIONAL CONSIDERATIONS – ALL STAGES

All supporting documentation and operational considerations are applicable to the entire platform trial and are not unique to the individual stages. These are therefore covered in the main protocol document.

APPENDIX 1 – SUB-STUDY 1 – INFLIXIMAB (REMICADE)

1. SUB-STUDY SUMMARY

1.1. Sub-study overview

The sub-study population corresponds to moderately and severely ill patients infected with the COVID-19 virus. Recruitment will target patients already hospitalized for treatment of COVID-19 infection as well as patients being treated for COVID-19 infection in Emergency Departments while waiting to be admitted to the hospital. Patients both in and out of the ICU are included in the study population.

Enrollment will begin as soon as all elements are met for the sub-study to activate.

The study period is 29 days, with assessments on each day of the hospital stay. Patients will be followed after hospital discharge with periodic follow-up assessments through Day 29 and at Day 60.

1.2. Enrollment Period

Enrollment began in October 2020. It is anticipated that enrollment will be completed in 4-6 months.

1.3. General

This sub-study is designed to evaluate Remicade for the treatment of moderately or severely ill hospitalized patients infected with COVID-19. This sub-study will have the same assessments and procedures as the main protocol.

The effectiveness of Remicade as add-on therapy to SoC will be evaluated based on the primary endpoint of time to recovery after 28 days. The sample size requirements are based on the ability to detect a moderate improvement in time to recovery (3-4 fewer days).

1.4. Study Population

Hospitalized adults (≥18 years old) with COVID-19, including patients both in and out of the ICU. Patients seeking care for COVID-19 in an Emergency Department (ED) and waiting to be admitted to the hospital are included.

1.5. Inclusion Criteria

Inclusion criteria are outlined in the main body of the ACTIV-1 IM protocol.

1.6. Agent Specific Exclusion Criteria at Randomization

1. Allergy to other monoclonal antibodies, or to any murine proteins.
2. History of HSTL or other lymphoma within 5 years before screening; history of or current diagnosis of multiple sclerosis (MS) or other significant demyelinating condition (e.g., optic neuritis).

1.7. Study Intervention

Therapeutic agents will be evaluated as an add-on therapy to SoC. The study intervention is as outlined in the main body of the ACTIV-1 IM protocol.

For the Remicade component, participants will receive active product as follows:

- Remicade will be administered as a single dose of 5 mg/kg (IV) on Day 1 in addition to SoC. The administration window will be within 24 hours of randomization. Infusion of Remicade and remdesivir should not be given concurrently in order not to confound attribution of possible infusion reactions or hypersensitivity events.

Duration of therapy:

- Remicade IV component – single dose on Day 1.

1.8. Schedule of Assessments

Assessments will be performed according to **Table 1-1** in 1.1.8 in the main body of the ACTIV-1 IM master protocol.

In addition, TB and HBV testing should be performed on Day 1 to rule out latent TB and/or HBV infection. Results do not need to be available for the participant to initiate treatment with study medication on Day 1. Once results are communicated, if the participant tests positive for TB or HBV, consultation with an infectious disease specialist and treatment for latent TB or hepatitis B should be considered.

2. JUSTIFICATION FOR SELECTION

Respiratory failure from acute respiratory distress syndrome (ARDS) is the leading cause of mortality associated with COVID-19 infection (Ruan et al. 2020).

Learnings from severe acute respiratory syndrome coronavirus 2 (SARS-CoV-2), initial COVID-19 data (Conti, Ronconi et al. 2020; Prompetchara et al. 2020) and preclinical mouse model data indicate that many cytokines including tumor necrosis factor α (TNFα), interleukin (IL)-1, and IL-6, may be key drivers of the acute lung injury (ALI) and ARDS observed in COVID-19. Severe COVID-19 patients from an initial study in China had elevated levels of TNFα. (Huang, Wang et al. 2020) SARS-CoV, a virus closely related to SARS-CoV-2, has been directly implicated in stimulating TNFα generation. The virus' spike protein has been shown to interact with angiotensin-converting enzyme 2 (ACE2) with consequent activation of TNFα converting enzyme (TACE), in a process that facilitates both viral entry into host cells and TNFα production (Haga et al. 2008).

Excess TNFα has been associated with multiple inflammatory diseases and may be an initiating or contributing factor to disease pathology and progression (Braun and Sieper 2003). TNFα has been reported to be an amplifier of inflammation, and suppressing it has the potential to suppress multiple downstream cytokines such as IL-1 and IL-6, as well as decrease capillary leak, and leukocyte trafficking as has been shown in rheumatoid arthritis (RA) patients (Feldmann et al. 2020).

Remicade binds to both soluble and transmembrane forms of TNFα and inhibits its functional activity. TNF antagonist therapy does not appear to be a risk factor for severe COVID-19 (Brenner et al. 2020).

No efficacy data are available on the use of Remicade to treat COVID-19 induced ARDS. Remicade has been approved across the globe for the treatment of Crohn's disease (adult and pediatric), RA, UC (adult and pediatric), psoriasis, PsA, and AS, although not all indications have been approved in all countries. Remicade has also been approved in Japan for use in some orphan indications including variants of psoriasis (psoriatic erythroderma and pustular psoriasis), Behçet's disease (entero-, neuro-, vasculo-, and uveitis), and acute Kawasaki disease.

Safety data from clinical studies are available from 6,122 Remicade-treated subjects including 2,363 with RA, 1,427 with Crohn's disease, 275 with AS, 191 with PsA, 1,373 with plaque psoriasis, and 493 with UC. Infusion-related reactions (e.g., dyspnea, flushing, headache and rash) were among the most common causes for discontinuation, except in UC, pediatric Crohn's disease, and PsA. Remicade has been mainly studied in chronic use, and approximately 3 million patients have been exposed to Remicade since its launch in August 1998.

3. INTRODUCTION

3.1. Risk/Benefit Assessment

3.1.1. *Remicade*

3.1.1.1. *Potential Risks of Remicade*

Risks that have been observed in patients with chronic use of Remicade but may also have relevance for single dose administration are the occurrence of serious infusion reactions, hypersensitivity, serious infections, liver enzymes increase, leukopenia or pancytopenia, and worsening of heart failure in those with NYHA Functional Class III/IV heart failure. Although there is a theoretical risk of cancer with the use of Remicade, this is felt to be of less concern with single dose administration. Of these, the most significant risks relate to the complications associated with immunosuppression, essentially mycobacterial, fungal, and viral infections.

The following risk mitigations are in place:

The inclusion/exclusion criteria prevent patients with pre-existing comorbidities (including non-COVID-19-related active infections, steroid dependency, being on renal replacement therapy, severe hepatic inflammation, history of HSTL or other lymphoma within 5 years before screening, current NYHA Functional Class III/IV heart failure) from participating.

Remicade administered at a dose of 10 mg/kg at 0, 2 and 6 weeks post randomization has been associated with increased combined risk of death from any cause or hospitalization for heart failure (hazard ratio 2.84, 95% confidence interval 1.01 to 7.97; nominal p=0.043) in the ATTACH Trial, a randomized study evaluating the use of Remicade in participants with NYHA Functional Class III/IV heart failure. Remicade at a dose of 5 mg/kg did not increase risk of mortality or worsening of heart failure compared to placebo (Chung et al. 2003).

Remicade may have a risk of reactivation of latent TB and chronic hepatitis B. Therefore, participants will be screened for both TB and hepatitis B. Results of these tests do not need to be available for the participant to initiate study medication. If the participant tests positive for TB or hepatitis B, consultation with an infectious disease specialist and treatment for latent TB or hepatitis B should be considered.

To further protect the participants from the immunomodulatory effects of Remicade that may predispose them to infections, the investigators are referred to the local guidelines for the concomitant use of antibacterial and/or antiviral agents that are intended for management of community acquired pneumonia or secondary bacterial infections, and to inhibit SARS CoV-2 viral activity.

3.1.1.2. *Potential Benefits of Remicade*

The potential benefit of Remicade is to fulfill an urgent medical need for patients by shortening the period of hospitalization.

3.1.1.3. *Assessment of Potential Risks and Benefits of Remicade*

The potential benefits of treatment with Remicade are considered to outweigh any associated risks of treatment. This is based upon the following:

The proposed target patient population consists of individuals who have hypoxemia and who are at risk of ARDS. These are the patients with the most urgent medical need who are at the greatest risk of dying. The current mortality due to COVID-19-associated pulmonary complications for hospitalized patients with hypoxemia and pulmonary involvement ranges from approximately 20-50% depending upon the age of the individual and pre-existing comorbidities.

Access of patients to investigational therapeutics to mitigate the disease is limited in this pandemic setting, and this study will offer the potential to access a therapeutic in the context of a clinical study.

Remicade has been approved for more than 20 years and has accrued safety data from estimated marketing exposure of over 3 million patients encompassing over 7 million patient years of exposure. Remicade is contraindicated for CHF at a dose higher than 5 mg/kg.

By excluding those with elevated liver transaminases, class 3 or 4 congestive heart failure or with concomitant infections, and with appropriate monitoring during the study as well as inclusion of an Independent Data Monitoring Committee (IDMC), risk to participants can be minimized. While there may not be benefits for an individual participant, there may be benefits to society if a safe, efficacious therapeutic agent can be identified during this global COVID-19 pandemic. The potential risks therefore are thought to be acceptable given the potential benefits.

4. OBJECTIVES AND ENDPOINTS

The overall objective of the study is to evaluate the clinical efficacy and safety of different investigational therapeutics relative to the control arm among hospitalized adults who have COVID-19. All the endpoints for the overall trial are defined in the body of the main ACTIV-1 IM protocol.

5. SUB-STUDY DESIGN

5.1. Justification for Dose

5.1.1. *Justification for Dose of Remicade*

Based on the findings reported, blood TNFα levels are significantly elevated in severe and critical COVID-19 patients (Chen et al. 2020; Wu et al. 2020). In one study, blood TNFα levels were found to correlate with disease severity with a substantial proportion of severe and critical COVID 19 patients having TNFα levels exceeding 11 pg/mL (Komatsu et al. 2001). Of note, median serum TNFα levels in patients with active inflammatory bowel disease (IBD; UC and CD) ranged between 9.5 to 14.0 pg/mL (Komatsu et al. 2001). Recent emerging data has suggested that levels of TNFα may reach roughly 100 pg/mL in critically ill COVID-19 patients (Huang et al. 2020). At these higher levels of TNFα, the Remicade 5 mg/kg dose provides substantial molar excess. Remicade exposure after a single dose of 5 mg/kg IV is predicted to yield roughly 100,000-fold molar excess at T_{max} and 20,000 fold at 14 days post-dose to plasma TNFα, assuming a ~100 pg/mL concentration of TNFα.Therefore, the Remicade dose proposed to be evaluated in severe or critical COVID-19 patients is 5 mg/kg administered as a single IV infusion.

In the approved labeling, with the exception of patients with RA who start with a dose of 3 mg/kg Remicade in conjunction with methotrexate and have the option of increasing the Remicade dose up to 10 mg/kg, the standard recommended starting dose of Remicade is 5 mg/kg for all other indications. The safety and efficacy of the 5 mg/kg dose regimen of Remicade is well established in many immune-mediated disease populations such as patients with RA, Crohn's disease, UC, and psoriasis.

In a short-term study of patients with CD where single doses of 5 mg/kg, 10 mg/kg, or 20 mg/kg Remicade were evaluated, no apparent dose-response was observed for this range of doses with respect to either magnitude or duration of clinical response or CRP reductions. Accordingly, it is not anticipated that doses greater than 5 mg/kg would be needed for acute treatment of COVID-19.

With respect to treatment duration, because the symptomatic phase of the disease lasts about 2 weeks (Zhou et al. 2020), it is reasonable to use a single dose of 5 mg/kg Remicade which is expected to provide adequate exposure to suppress elevated TNFα levels for the duration of the symptomatic phase of COVID-19 infection.

In summary, Remicade 5 mg/kg administered as a single IV infusion is expected to be effective with acceptable risks in rapidly suppressing the elevated TNFα levels in participants with confirmed severe or critical COVID-19.

6. STUDY POPULATION

The study population will adhere to the master protocol population, except for the noted additional exclusions and considerations below.

6.1. Inclusion Criteria

The same as the master protocol.

6.2. Sub-Study Specific Exclusion Criteria

1. Allergy to other monoclonal antibodies, or to any murine proteins.
2. History of HSTL or other lymphoma within 5 years before screening; history of or current diagnosis of MS or other significant demyelinating condition (e.g., optic neuritis).

6.3. Exclusion of Specific Populations

Since Remicade does not cross-react with TNFα in species other than humans and chimpanzees, animal reproduction studies have not been conducted with Remicade. No evidence of maternal toxicity, embryotoxicity, or teratogenicity was observed in a developmental toxicity study conducted in mice using an analogous antibody that selectively inhibits the functional activity of mouse TNFα. Doses of 10 mg/kg to 15 mg/kg in PD animal models with the anti-TNF analogous antibody produced maximal pharmacologic effectiveness. Doses up to 40 mg/kg were shown to produce no adverse effects in animal reproduction studies. It is not known whether Remicade can cause fetal harm when administered to a pregnant woman or can affect reproductive capacity. Remicade should be given to a pregnant woman only if considered that the benefits outweigh the risks.

As with other IgG antibodies, Remicade crosses the placenta. Remicade has been detected in the serum of infants up to 6 months following birth.

After in utero exposure to Remicade, infants may be at increased risk of infection, including disseminated infection that can become fatal. It is recommended that therapeutic infectious agents are not given concurrently with Remicade.

It is not known whether Remicade is excreted in human milk or absorbed systemically after ingestion. Because many drugs and immunoglobulins are excreted in human milk, and because of the potential for ADRs in nursing infants from REMICADE, a decision should be made whether to discontinue nursing or to discontinue the drug, taking into account the importance of the drug to the mother.

7. STUDY PRODUCT

7.1. Study Product(s) and Administration

Participants will be randomized according to the procedure described in the master protocol.

If there are supply limitations on any product, the arms containing that product will be temporarily closed to enrollment and the corresponding placebo is not needed.

7.1.1. *Study Product Description*

Remicade is a chimeric human-murine IgG1γ monoclonal antibody that specifically binds to human TNFα. In addition to the active ingredient, the lyophilized formulation of Remicade contains the following inactive ingredients: water for injection, sucrose, polysorbate 80, monobasic sodium phosphate monohydrate, and dibasic sodium phosphate dihydrate.

7.1.2. *Dosing and Administration*

For the Remicade component, participants will receive either active product or placebo as follows:

- Remicade will be administered as a single dose of 5 mg/kg (IV) on Day 1 in addition to SoC.
- A placebo of IV saline will be given at an equal volume at the same schedule in addition to SoC.

The administration window will be within 24 hours of randomization. Any dose that is delayed may be given later that calendar day. Any dose that is missed (not given that calendar day) is not made up. The treatment course continues as described above even if the participant becomes PCR negative.

Duration of therapy:

- Remicade IV component or saline placebo – single dose on Day 1.

7.1.3. *Dose Escalation*

Not Applicable

7.1.4. *Dose Modifications*

For mild to moderate infusion reactions, the patient may improve following slowing or suspension of the infusion, and upon resolution of the reaction, re-initiation at a lower infusion rate and/or therapeutic administration of antihistamines, acetaminophen, and/or corticosteroids. For participants who do not tolerate the infusion following these interventions, Remicade should be discontinued.

Participants who have severe infusion-related hypersensitivity reactions should be discontinued from further Remicade treatment. The management of severe infusion reactions should be dictated by the signs and symptoms of the reaction. Appropriate personnel and medication should be available to treat anaphylaxis if it occurs.

7.1.5. *Overdosage*

There is no known specific antidote for Remicade overdose. In the event of an overdose, the participant should be monitored for any signs or symptoms of adverse effects and institute appropriate symptomatic treatment immediately.

7.1.6. *Preparation/Handling/Storage/Accountability*

7.1.6.1. *Acquisition and Accountability*

Investigational products (IP) will be shipped to the site either directly from participating companies, from the Sponsor, or from other regional or local drug repositories. All other supplies should be provided by the site. Multiple lots of each IP may be supplied.

Study products received at the sites will be open label and not kit specific, unless specified in the protocol-specific Manual of Procedures (MOP) or pharmacy manual. Drug preparation will be performed by the participating site's unblinded research pharmacist on the same day of administration to the participant. See the MOP Appendices for detailed information on the preparation, labeling, storage, and administration of investigational products.

Accountability:

The site PI is responsible for study product distribution and disposition and has ultimate responsibility for study product accountability. The site PI may delegate to the participating site's research pharmacist responsibility for study product accountability. The participating site's research pharmacist will be responsible for maintaining complete records and documentation of study product receipt, accountability, dispensation, storage conditions, and final disposition of the study product(s). Time of study drug administration to the participant will be recorded on the appropriate data collection form (CRF). All study product(s), whether administered or not, must be documented on the appropriate study product accountability record or dispensing log. The Sponsor's monitoring staff will verify the participating site's study product accountability records and dispensing logs per the site monitoring plan. Refer to the protocol-specific MOP for details on storing study medications.

Destruction:

After the study treatment period has ended or as appropriate over the course of the study after study product accountability has been performed, used active and placebo product can be destroyed on-site following applicable site procedures with a second staff member observing and verifying the destruction.

Unused product at the end of the study should be saved until instructed by the Sponsor.

7.1.6.2. *Formulation, Appearance, Packaging, and Labeling*

Remicade component: The lyophilized formulation of Remicade is a preservative-free, white, lyophilized solid containing 100 mg of Remicade to be reconstituted with 10 mL of sterile water for injection and diluted into IV infusion fluids prior to IV infusion. Following reconstitution, each vial contains a 10 mg/mL Remicade concentrated solution.

It is supplied as a sterile product in a single-use, Type 1 clear glass vial. In addition to the active ingredient, the lyophilized formulation of Remicade contains the following inactive ingredients: water for injection, sucrose, polysorbate 80, monobasic sodium phosphate monohydrate, and dibasic sodium phosphate dihydrate. For more information, refer to the MOP.

Injectable placebo component:

Injectable placebo will be IV normal saline (0.9% NaCl) made up unblinded by the resident pharmacist and blinded to the study team. Note that the type of infusion bag used for placebo should be identical to that used for the preparation of the Remicade infusion in order to ensure the blind in the study.

7.1.6.3. *Product Storage and Stability*

Study intervention must be stored at controlled temperatures as indicated on the product label and should not be used beyond the expiration date.

Refer to the MOP for additional guidance on study intervention storage.

7.1.6.4. *Preparation and Administration*

Refer to the protocol-specific MOP for details about preparation.

Remicade 5 mg/kg will be administered to the participant via an IV infusion at a rate of not more than 100 mL/h over a time period of not less than 2 hours using a total volume of 250 mL. Placebo participants will receive the IV infusion of 250 mL normal saline at the same rate. Aseptic procedures must be used during the study agent infusion.

Remicade must not be infused with other agents in the same infusion line. Generally, lines should be flushed before and after use if to be used for administration of other medications. In addition, the infusion of Remicade should not be given concurrently with the infusion of remdesivir in order not to confound attribution of possible infusion reactions or hypersensitivity events.

Refer to the MOP for additional guidance on study intervention preparation and handling.

7.2. Measures to Minimize Bias: Randomization and Blinding

Randomization will be performed as specified in the Master Protocol

7.3. Study Intervention Compliance

Each dose of study product will be administered by a member of the clinical research team who is qualified and licensed to administer the study product. Administration date and infusion start/stop times will be entered into the case report form (CRF).

7.4. Concomitant Therapy

7.4.1. *Permitted Concomitant Therapy and Procedures*

All concomitant therapies permitted in the master protocol will be allowed for this sub-study.

Of further note in regard to co-administration of corticosteroids, Remicade in combination with corticosteroids, including dexamethasone, has been used in clinical trials and 20+ years of post-marketing experience in several

immuno-mediated inflammatory diseases with an overall positive benefit-risk profile. This profile was established in the context of long-term use of Remicade in patients without active infection at the time of initiation of treatment.

7.4.2. *Prohibited Concomitant Therapy*

All concomitant therapies prohibited in the master protocol will also be prohibited for this sub-study.

Medications to be used with caution as their exposure could be modified by the study medication

The formation of CYP450 enzymes may be suppressed by increased levels of cytokines (e.g., TNFα, IL-1, IL-6, IL-10, IFN) during chronic inflammation. Therefore, it is expected that for a molecule that antagonizes cytokine activity, such as Remicade, the formation of CYP450 enzymes could be normalized. Upon initiation of Remicade in patients being treated with CYP450 substrates with a narrow therapeutic index, monitoring of the effect (e.g., warfarin) or drug concentration (e.g., cyclosporine or theophylline) is recommended and the individual dose of the drug product may be adjusted as needed.

7.4.3. *Rescue Medicine*

Not Applicable.

7.4.4. *Non-Research Standard of Care*

Not Applicable.

8. STUDY INTERVENTION DISCONTINUATION AND SUBJECT DISCONTINUATION/WITHDRAWAL

8.1. Halting Criteria and Discontinuation of Study Intervention

8.1.1. *Individual Study Product Halting*

Individual study product halting will be managed as specified in the Master Protocol.

Remicade Halting

For mild to moderate infusion reactions, the patient may improve following slowing or suspension of the infusion, and upon resolution of the reaction, re-initiation at a lower infusion rate and/or therapeutic administration of antihistamines, acetaminophen, and/or corticosteroids. For patients that do not tolerate the infusion following these interventions, Remicade should be discontinued.

Patients who have severe infusion-related hypersensitivity reactions should be discontinued from further Remicade treatment. The management of severe infusion reactions should be dictated by the signs and symptoms of the reaction. Appropriate personnel and medication should be available to treat anaphylaxis if it occurs.

8.1.2. *Study Halting*

Study halting will be managed as specified in the Master Protocol.

8.2. Withdrawal from the Study

Sub-study will have the same requirements as the main study.

8.3. Lost to Follow-Up

Sub-study will have the same requirements as the main study.

8.4. Readmission

Sub-study will have the same requirements as the main study.

9. STUDY ASSESSMENTS AND PROCEDURES

Sub-study will have the same assessments and procedures as the main protocol.

10. STATISTICAL CONSIDERATIONS

Statistical analysis will be performed as described within the main body of the ACTIV-1 IM master protocol.

APPENDIX 2 – SUB-STUDY 2 – ABATACEPT (ORENCIA)

1. SUB-STUDY SUMMARY

1.1. Sub-study overview

The sub-study population corresponds to moderately and severely ill patients infected with the COVID-19 virus. Recruitment will target patients already hospitalized for treatment of COVID-19 infection as well as patients being treated for COVID-19 infection in Emergency Departments while waiting to be admitted to the hospital. Patients both in and out of the ICU are included in the study population.

Enrollment will begin as soon as all elements are met for the sub-study to activate.

The study period is 60 days, with assessments on each day of the hospital stay. Patients will be followed after hospital discharge with periodic follow-up assessments through Day 29 and at Day 60.

1.2. Enrollment Period

Enrollment began in October 2020. It is anticipated that enrollment will be completed in 4-6 months.

1.3. General

This sub-study is designed to evaluate abatacept for the treatment of moderately or severely ill hospitalized patients infected with COVID-19. Sub-study will have the same assessments and procedures as the main protocol.

The effectiveness of abatacept as add-on therapy to SoC will be evaluated based on the primary endpoint of time to recovery after 28 days. The sample size requirements are based on the ability to detect a moderate improvement in time to recovery (3-4 fewer days).

1.4. Study Population

Hospitalized adults (≥18 years old) with COVID-19, including patients both in and out of the ICU. Patients seeking care for COVID-19 in an Emergency Department (ED) and waiting to be admitted to the hospital are included.

1.5. Inclusion Criteria

Inclusion criteria are outlined in the main body of the ACTIV-1 IM protocol.

1.6. Agent Specific Exclusion Criteria

There are no agent specific exclusion criteria for this sub-study.

1.7. Study Intervention

Therapeutic agents will be evaluated as an add-on therapy to SoC. The study intervention is as outlined in the main body of the ACTIV-1 IM protocol.

For the abatacept component, the dose of abatacept will be 10 mg/kg with a maximum dose of 1000 mg. Study medication will be administered in a fixed volume of 100 mL at a constant infusion rate over approximately 30 minutes. The IV line must be flushed with 25 mL of NS solution at the end of the infusion. The administration window will be within 24 hours of randomization.

Infusion of abatacept and remdesivir should not be given concurrently in order not to confound attribution of possible infusion reactions or hypersensitivity events.

Duration of therapy:

- Abatacept IV component – single infusion on Day 1.

1.8. Schedule of Assessments

Assessments will be performed according to **Table 1-1** in 1.1.8 in the main body of the ACTIV-1 IM master protocol.

2. JUSTIFICATION FOR SELECTION

It is hypothesized that abatacept therapy can modulate the ongoing/emerging dysregulated immune response considered to be driving the progression of disease severity in COVID-19. Abatacept is not a therapy for neutralizing cytokines but for preventing further cellular activation (e.g., CD4+ T cells, B cells, macrophages) and production of cytokines and interrupting the feedforward pathological process.

Appendix 2 Figure 1: Cellular interactions affected by abatacept

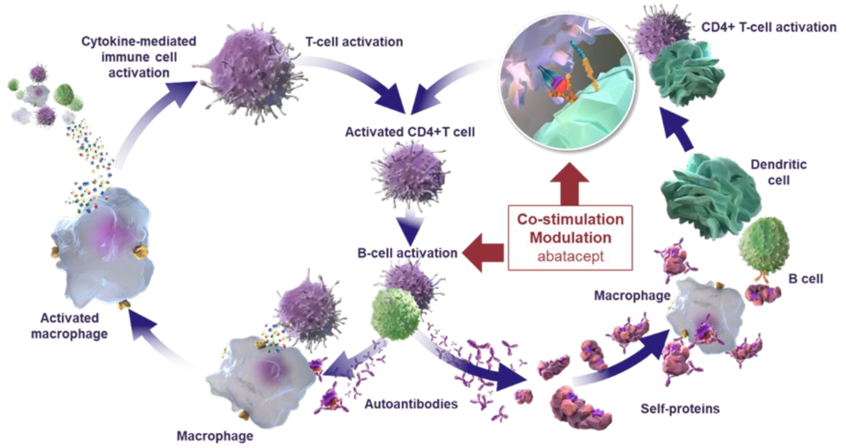

In vitro evidence of abatacept impact on cytokine production by multiple cell types

Abatacept was first studied in the setting of a pathological response to a viral infection in a murine model of influenza (Teijaro et al. 2009). In this study, abatacept could prevent development of severe lung injury while not inhibiting an effective memory antiviral response in the setting of secondary infection. This study, along with others, have continued to evolve the understanding of the mechanism of action (MOA) of abatacept which has proven to be much more complex than initially envisioned. Although CTLA-4 is central to the regulation of T cell activation, CTLA-4Ig clearly has effect on other cell types. Since CTLA-4 regulates interactions with T cells and professional antigen presenting cells (APC), it is not surprising that effects on APC populations (i.e., dendritic cells, macrophages, B cells) can also be seen, in vitro and clinically (Chambers and Allison 1999). Indeed, in vitro work has demonstrated reverse signaling via CD80 or CD86 in APCs although the full impact of this is not fully understood (Davis et al. 2008).

Treatment with abatacept has also been shown to have broad effects on cytokines in RA patients. Abatacept shows an impact within 24 hours in human in vitro mixed-lymphocyte reactions (MLR) on IL-2, TNF-α, and IFN-γ (Davis, Nadler et al. 2008). In vivo, abatacept reduces the levels of multiple cytokines in RA patients including TNF-α, sIL-2R and IL-6 (Weisman et al. 2006). In addition, abatacept suppresses multiple biomarkers (cytokines and chemokines) in the synovium from RA patients (Buch et al. 2009). What these studies don't show is which cell type(s) was most impacted.

The best evidence for abatacept's direct effects on APCs comes from reports on both in vitro and in vivo effects on macrophages and B cells. Abatacept inhibits pro-inflammatory cytokine (TNF-a, IL-12, INF-g) secretion by in vitro activated human macrophages (Wenink et al. 2012). Abatacept can also downregulate production of IL-6, TNFα, IL1-β and TGFβ from activated synovial macrophages from RA patients (Cutolo et al. 2009). B-cell populations are

notably decreased in RA synovium of abatacept-treated patients (Buch et al. 2009). Direct effects of abatacept on plasmablasts has also been proposed (Carvajal Alegria et al. 2016). Together, all of this data suggests that abatacept's impact on the immune system is not limited to T cells. Activations of macrophages and B cells are also likely impacted by abatacept therapy.

Background

Abatacept is a recombinant fusion protein (MW 92 kDa) consisting of the extracellular domain of human CTLA4 and a fragment (hinge - CH2 - CH3 domains) of the Fc domain of human immunoglobulin (Ig) GI that has been modified to prevent complement fixation and antibody-dependent cellular cytotoxicity. Abatacept is a selective costimulation modulator that inhibits T-cell activation by binding to CD80 and CD86 on antigen presenting cells, thereby blocking the interaction with CD28 on T-cells that provides a costimulatory signal necessary for full activation of T-cells. By inhibiting CD28 mediated T-cell activation upstream of inflammatory cytokines, such as TNF, abatacept utilizes a unique mechanism of action that offers significant therapeutic benefit to patients with a variety autoimmune-mediated diseases (Linsley and Nadler 2009). Abatacept, 250 mg for intravenous (IV) infusion, is indicated to treat participants with rheumatoid arthritis (RA) age 18 and older and participants with polyarticular juvenile idiopathic arthritis (pJIA) age 6 to 17.

IV-administered abatacept was first approved in the US for the treatment of moderate-to-severe RA in adults in December 2005. Since then, IV abatacept has received marketing approval for the treatment of adult RA in many other countries, including the EU, Canada, Australia, and Japan. IV abatacept was also approved in the US for the treatment of moderately-to-severely active juvenile idiopathic arthritis (JIA) in pediatric patients 6 years of age or older in April 2008. In addition, IV abatacept has received marketing approval for the treatment of JIA in several other countries, including the EU, Canada, and Australia. In 2017, abatacept was approved for adult use in psoriatic arthritis (PsA) in the US and has also received marketing approval for the treatment of PsA in several other countries, including the EU, Canada, and Australia. A subcutaneous (SC) formulation of abatacept in a prefilled syringe and autoinjector has been approved for adult RA and PsA patients in the US, EU, and several other countries; it is also approved in the US and EU for use in JIA.

A detailed description of the chemistry, pharmacology, efficacy, and safety of abatacept is provided in the Investigator's Brochure (IB) (Company, B.M.S. 2020a; Company, B.M.S. 2020b).

Clinical Evidence for Abatacept Suppression of Cytokine Release Syndrome

There is some clinical data to inform the impact on cytokine release syndrome (CRS). Patients with active systemic JIA, some with current macrophage activation syndrome (MAS), who were heavily treated with multiple agents, including high dose corticosteroids and anakinra, responded to the addition of abatacept. The time course for the benefit of abatacept is not well described but the ability to decrease doses or suspend some concomitant therapy is reported.

A subject with active immune checkpoint inhibitor-associated myocarditis did demonstrate clinical and biochemical improvement within a day of initiating abatacept therapy (Salem et al. 2019). This clinical course has been seen in other similar patients treated with abatacept (personal communication of unpublished data).

The strongest evidence for rapid onset of abatacept and its effect on CRS is from prevention, not treatment. Use of peripheral blood stem cell (PBSC) graft for post-transplantation cyclophosphamide (PTCy)-based haploidentical hematopoietic cell transplantation (HCT) is associated with early CRS in over 92% of patients (14% severe CRS) (Jaiswal and Chakrabarti 2020). Treatment with abatacept 1 day before the infusion of the PBSC graft (followed dosing on day +5, +20, +35 and every four weeks after) decreased the rate of CRS to 6% (none severe). The continued use of abatacept is because of ongoing donor lymphocyte infusions, which are therapeutic in this context and also believed to be the source of the CRS process.

In a related clinical setting, abatacept has been studied for acute graft-versus-host disease (aGVHD) prevention during unrelated-donor hematopoietic cell transplantation (HCT), in both HLA matched (8/8) and unmatched (7/8) patients

(Koura et al. 2013; Watkins et al. 2017). Patients who receive unrelated-donor HCT are at elevated risk of severe aGVHD and death. Abatacept treatment at days -1, +5, +14, and +28 almost completely suppresses severe (Grade III-IV) aGVHD without negative impact on relapse and without significant impact on patient safety. CMV reactivation, but not EBV, was seen more frequently (47% vs 33%, p=0.16) but uncontrolled infections were not a major problem.

The relevance of these reports is that they illustrate the rapid effects of abatacept on feedforward mechanisms that produce CRS. They also suggest that the onset of abatacept's therapeutic effects can be sufficiently rapid as to be clinically relevant in the setting of COVID-19.

3. INTRODUCTION

3.1. Risk/Benefit Assessment

3.1.1. *Abatacept*

3.1.1.1. *Potential Risks of Abatacept*

The safety profile of abatacept is well established based on the safety data collected from a number of clinical studies and 14-year post-marketing experience during the treatment of autoimmune/inflammatory diseases; abatacept has been well-tolerated and shown a favorable benefit-risk profile in the approved disease population (i.e., RA, PsA, pJIA).

Infections and infusion related reactions (for IV formulation) are identified risks for abatacept, the majority of these events are non-serious and do not impact benefit/risk profile of the product. For infections, during the double-blind, placebo-controlled period of RA studies (9 IV studies and 2 SC studies in the integrated safety database), the incidence rate of overall infections was comparable between abatacept group (total of 2,653 subjects with mean exposure duration of 10.8 months) and placebo group (total of 1,485 subjects with mean exposure duration of 10.3 months): 93.21 versus 93.02 per 100 patient-years; a small increase for IR of overall serious infections in the abatacept group compared to placebo group (3.0 vs 2.25 per 100 patient-years). Majority of the infections were caused by common pathogens; the most commonly reported PTs of infections were upper respiratory tract infection (IR 14.3 per 100 patient-years) and nasopharyngitis (IR 14.3 per 100 patient-years); the most frequently reported serious infection was pneumonia (IR 0.68 per 100 patient-years) in abatacept group, which was comparable to that in placebo group (IR 0.72 per 100 patient-years). The IR of opportunistic infections appeared to be numerically lower in abatacept group compared to placebo (0.17 vs 0.56 per 100 patient years). For the infusion related reactions occurred in IV abatacept studies, most of the peri-infusional events that occurred within 24 hours following the start of infusion were non-serious dizziness and nausea; serious events of hypersensitivity/anaphylactic reactions were rare. Safety experience from clinical studies in PsA and pJIA patients was similar to that in RA studies. Ten-year post-marketing epidemiology studies in RA population did not reveal any new safety finding relevant to these two identified risks.

Malignancies and autoimmune disorders have been reported with abatacept use; however, the clinical and post-marketing data do not show an increased risk of these events associated with abatacept use, therefore, the potential role of abatacept in the development of these diseases cannot be concluded based on the available evidence.

The potential deleterious impact of abatacept on viral clearance is certainly a concern that we recognize. The previously discussed murine model of influenza infection-induced immunopathology clearly demonstrates the risk of blunting anti-viral responses in abatacept-treated mice prior to viral exposure (Teijaro et al. 2009). These concerns are not new. Throughout the abatacept clinical development program, concerns over the impact of therapy on infectious agents has been paramount.

During pre-clinical abatacept development, host resistance models of infection were explored (Linsley and Nadler 2009). Of note, negative impacts on immune responses were only noted for herpes simplex infections. The observation that abatacept partially inhibits the generation of CD8 cytotoxic T cell (CTL) responses but does not inhibit CD8 T cell cytotoxic activity has been reported, which could also predict deleterious impact on viral responses (Van Gool et al. 1996; Whitmire and Ahmed 2000).

In spite of these pre-clinical observations, clinical observations, both from controlled trials and post marketing surveillance, have been of less concern. A review of safety data of viral infections from an integrated safety database of RA clinical studies (includes 7044 participants receiving IV or SC abatacept for a total exposure of 21994.84 patient years during the cumulative period (controlled plus long-term open label)), reveals the following:

1. During the randomized placebo-controlled double-blind short-term period of the studies, the most frequently (>1.0%) reported PTs of viral infections and infections that could be caused by virus in the ABA group vs. placebo group, respectively, were upper respiratory tract infection (11.9% vs 12.1%), nasopharyngitis (11.8% vs 10%), bronchitis (6.5% vs 5.8%), influenza (5.8% vs 5.9%), pharyngitis (3.5% vs 3.3%), rhinitis (2.4% vs 1.3%), conjunctivitis (2.1% vs 1.5%), gastroenteritis (2.0% vs 3.0%), oral herpes (1.8% vs 1.3%), herpes zoster (1.5% vs 1.4%), pneumonia (1.8% vs 1.1%). Majority of these events were mild or moderate in severity and abatacept use did not cause increased severity of infections compared to placebo.
2. During the DB ST period of the studies, the most frequently reported PTs (≥ 2 cases) of serious viral infections and infections that could be caused by virus in ABA group and placebo group were pneumonia (0.6% vs 0.6%), bronchitis (0.2% vs 0), gastroenteritis (0.1% vs 0.1%), herpes zoster (0.1% vs 0). Among these events, death outcomes were reported for pneumonia and the IRs for fatal pneumonia were 0.08 vs 0.16 per 100 patient-years for abatacept and placebo, respectively.
3. There were no increases for the incident rates of serious infections reported during cumulative period compared to those reported in short-term period.

Of note is the absence of an imbalance in viral pneumonias, specifically influenza. Also, the median event duration for influenza was similar between abatacept treated patients and placebo group. Antibody responses to the conventional seasonal influenza vaccine in abatacept treated RA patients were also studied (Alten et al. 2016). The study demonstrated that most participants on abatacept were able to mount an appropriate immune response. While this study did not demonstrate impact on actual influenza rates, it does support the extensive clinical observation that abatacept therapy may not influence anti-influenza responses in a clinically meaningful way.

Review of the post-marketing safety experience (estimated exposure at 763,109 p-y as of 30-Sep-2019 in > 13 years) identified 21 cases of coronavirus infection by 13-Apr-2020, events of confirmed COVID-19 or suspected COVID-19 were reported in 13 of these cases. The majority of these cases contained minimal information and robust assessment of the impact of abatacept on COVID-19 is not possible. One literature report from Japan reported possible COVID-19 in a 70-year-old male received abatacept and tacrolimus to treat RA and interstitial lung disease (ILD). The patient developed respiratory compromise and ultimately required ventilatory support. Testing of sputum but no other sources was positive for SAR-CoV-2. A recent published letter describes 8 patients being treated with targeted therapies for chronic arthritis in Italy. Among them were 2 patients receiving therapy with abatacept, 1 with confirmed and one with suspected SARS-CoV-2 infection. Only 1 of the patients required hospitalization, and then only briefly for oxygen therapy and all recovered without incident (Monti et al. 2020). Belatacept is a related drug to abatacept with a similar mechanism of action. In a recent report (Marx et al. 2020), a 58 year old kidney transplant patient on belatacept and MMF became infected with SARS-CoV-2 and had a mild clinical course with rapid recovery. The authors hypothesized that ongoing immunotherapy with belatacept may protect from severe clinical course of COVID-19 by limiting cytokines/chemokines production.

The abatacept label has a warning for risk of serious infections, including fatal sepsis and pneumonia. The clear risk for abatacept use is in bacterial infections, mostly of the respiratory and urinary tract. This includes a higher rate of complications in patients with COPD. The abatacept label also warns about treatment initiation in patients with an active infection until the infection is controlled. Of note, extensive experience in the use of abatacept to treat RA in elderly patients with co-morbidities has been reported (Monti et al. 2017). This suggests that among biologic DMARD therapies, abatacept may have less risk of infections (Curtis et al. 2014; Kang et al. 2019; Chen et al. 2020).

Based on the review of safety data relevant to viral infections from clinical studies and post marketing experience, there is no evidence suggesting increased risk of severe respiratory illnesses associated with abatacept use. Current evidence suggests that once COVID-19 patients develop symptoms of respiratory compromise, their anti-viral responses, including adaptive immune responses, are well underway but the pathological process leading to CRS-like scenario has only just begun. It is proposed that abatacept may interrupt this process without greatly interfering with the viral clearance and that any study to be attempted should be aware of these goals and concerns.

3.1.1.2. *Potential Benefits of Abatacept*

It is hypothesized that abatacept therapy can modulate the ongoing/emerging dysregulated immune response considered to be driving the progression of disease severity in the setting of SARS-CoV-2 infection. Abatacept is not a therapy for neutralizing cytokines but for preventing further cellular activation (e.g. CD4+ T cells, B cells, macrophages) and production of cytokines and interrupting the feedforward pathological process. The sponsor's hypothesis is that once patients with COVID-19 develop symptoms of respiratory compromise, their anti-viral responses, including adaptive immune responses, are well underway but the pathological process leading to the CRS-like scenario has not yet fully developed. We hypothesize that abatacept may interrupt this process without negatively impacting viral clearance.

Abatacept has demonstrated the ability to modulate the maladaptive immune response in multiple clinical models of Cytokine Release Syndrome (CRS) and in clinical conditions characterized by CRS. The most relevant data for the use of abatacept in the setting of COVID-19 comes from a mouse model of influenza pneumonitis. In this model, treatment with CTLA4-Ig decoupled the protective and immunopathological memory T cell responses following secondary infection without effecting viral clearance and reduced the development of the immunopathology and enhanced recovery. The use of CTLA4-IG in mouse models of both Toxic Shock and Staph B induced cytokine storm resulted in significant reductions in morbidity (including lung damage and edema) and mortality, and lower levels of both Il-6 and Ifn-ϒ (Whitfield et al. 2017).

3.1.1.3. *Assessment of Potential Risks and Benefits of Abatacept*

Current evidence indicates that once COVID-19 patients develop symptoms of respiratory compromise, their anti-viral responses, including adaptive immune responses, are well underway but the pathological process leading to a CRS-like scenario has only just begun. The sponsor proposes that abatacept may interrupt this process without greatly interfering with the viral clearance.

Preclinical work indicates that abatacept can suppress or control cytokine production, including clinical scenarios consistent with CRS. Clinical work in multiple complex disease states also suggests abatacept therapy can suppress or help manage excessive cytokine production.

Abatacept has been studied in human clinical trials and marketed for over 15 years. Most patients treated have chronic, immune-mediated diseases with use of concomitant immunosuppressant therapies and co-morbid conditions. The most notable risk for abatacept use is an increase in the rate of bacterial infections, particularly of the respiratory and urinary tract. Based on the review of safety data relevant to viral infections from clinical studies and post-marketing experience, there is no evidence suggesting an increased risk of viral respiratory illnesses, severe or otherwise, associated with abatacept use.

The study of the therapeutic potential of abatacept in symptomatic patients with COVID-19 at high risk for progression to respiratory failure is warranted. Concerns over the negative impact on viral clearance or secondary bacterial infections are appropriate. These concerns are mitigated by the relatively short course of treatment in the setting of hospitalized monitoring and care, monitoring of super-infections during the study and an oversight study committee.

More detailed information about the known and expected benefits and risks and reasonably anticipated adverse events of abatacept may be found in the Investigator's Brochure (Company, B.M.S. 2020a; Company, B.M.S. 2020b).

4. OBJECTIVES AND ENDPOINTS

The overall objective of the study is to evaluate the clinical efficacy and safety of different investigational therapeutics relative to the control arm among hospitalized adults who have COVID-19. All the endpoints for the overall trial are defined in the body of the main ACTIV-1 IM protocol.

5. STUDY DESIGN

5.1. Justification for Dose

5.1.1. *Justification for Dose of Abatacept*

Abatacept is available in 2 formulations, lyophilized for intravenous infusion and pre-filled syringe for subcutaneous injection. Pharmacokinetic evaluation over 2 decades of clinical development has shown that the most relevant PK parameter is trough (Cmin) levels which are best at predicting maximal efficacy and are driven by weight-based dosing. Superior efficacy is observed with 10 mg/kg dosing in RA patients. Dosing frequency of Day 1, 15, 28 and every 28 days afterwards was established as an efficacious dosing regimen for reaching target Cmin levels. Evidence based on pharmacokinetic, pharmacodynamic and clinical efficacy data suggest that higher doses of abatacept would not be more effective in RA patients. Higher doses of abatacept at 30 mg/kg have been tested in BMS sponsored trials. In patients with PsA, higher doses did not produce better efficacy but also did not result in any increase in safety signal. In inflammatory bowel disease, no clinical efficacy was seen. In lupus nephritis, higher doses were thought needed due to urinary losses of abatacept due to the severe proteinuria unique to this disease. None of these studies suggested higher abatacept doses were associated with increased safety concerns. Based on the experiences in other systemic inflammatory diseases, which we believe are somewhat informative as to what we will see in COVID-19 related disease, we believe a one time IV dose of abatacept would be sufficient to provide continuous exposure above the target Cmin and have no reason to believe a higher dose or higher frequency would be needed.

6. STUDY POPULATION

The study population will adhere to the master protocol population, except for the noted additional exclusions and considerations below.

6.1. Inclusion Criteria

The same as the master protocol.

6.2. Sub-Study Specific Exclusion Criteria

The same as the master protocol.

6.3. Exclusion of Specific Populations

Children and adolescents will not be included in this trial. Abatacept has been used extensively in pediatric patients.

The data with abatacept use in pregnant women are insufficient to inform on drug-associated risk. In reproductive toxicology studies in rats and rabbits, no fetal malformations were observed with intravenous administration of abatacept during organogenesis at doses that produced exposures approximately 29 times the exposure at the maximum recommended human dose (MRHD) of 10 mg/kg/month on an AUC basis. However, in a pre- and postnatal development study in rats, ORENCIA altered immune function in female rats at 11 times the MRHD on an AUC basis. There are no adequate and well-controlled studies of abatacept use in pregnant women. The data with abatacept use in pregnant women are insufficient to inform on drug-associated risk.

Intravenous administration of abatacept during organogenesis to mice (10, 55, or 300 mg/kg/day), rats (10, 45, or 200 mg/kg/day), and rabbits (10, 45, or 200 mg/kg every 3 days) produced exposures in rats and rabbits that were approximately 29 times the MRHD on an AUC basis (at maternal doses of 200 mg/kg/day in rats and rabbits), and no embryotoxicity or fetal malformations were observed in any species

In a study of pre- and postnatal development in rats (10, 45, or 200 mg/kg every 3 days from gestation day 6 through lactation day 21), alterations in immune function in female offspring, consisting of a 9-fold increase in T-cell-dependent antibody response relative to controls on postnatal day (PND) 56 and thyroiditis in a single female pup on PND 112, occurred at approximately 11 times the MRHD on an AUC basis (at a maternal dose of 200 mg/kg). No adverse effects were observed at approximately 3 times the MRHD (a maternal dose of 45 mg/kg). It is not known if

immunologic perturbations in rats relevant indicators of a risk for development of autoimmune diseases in humans are exposed in utero to abatacept. Exposure to abatacept in the juvenile rat, which may be more representative of the fetal immune system state in the human, resulted in immune system abnormalities including inflammation of the thyroid and pancreas [see Nonclinical Toxicology.

There is no information regarding the presence of abatacept in human milk, the effects on the breastfed infant, or the effects on milk production. However, abatacept was present in the milk of lactating rats dosed with abatacept.

7. STUDY PRODUCT

7.1. Study Product(s) and Administration

Participants will be randomized according to the procedure described in the master protocol.

If there are supply limitations on any product, the arms containing that product will be temporarily closed to enrollment and the corresponding placebo is not needed.

7.1.1. *Study Product Description*

Abatacept for Injection is a lyophilized powder for intravenous infusion. ORENCIA for Injection is supplied as a sterile, white, preservative-free, lyophilized powder for reconstitution and dilution prior to intravenous administration. Following reconstitution of the lyophilized powder with 10 mL of Sterile Water for Injection, USP, the solution of abatacept is clear, colorless to pale yellow, with a pH range of 7.2 to 7.8. Each single-use vial of abatacept for Injection provides 250 mg abatacept, maltose (500 mg), monobasic sodium phosphate (17.2 mg), and sodium chloride (14.6 mg) for administration.

7.1.2. *Dosing and Administration*

The dose of abatacept will be 10 mg/kg with a maximum dose of 1000 mg. Study medication will be administered in a fixed volume of 100 mL at a constant infusion rate over approximately 30 minutes. The IV line must be flushed with 25 mL of NS solution at the end of the infusion. The administration window will be within 24 hours of randomization.

Infusion of abatacept and remdesivir should not be given concurrently in order not to confound attribution of possible infusion reactions or hypersensitivity events.

Duration of therapy:

- Abatacept IV component – single infusion on Day 1.

7.1.3. *Dose Escalation*

Not Applicable

7.1.4. *Dose Modifications*

In the absence of adverse events, participants will complete their scheduled infusion as prescribed by protocol. If there is evidence of toxicity (e.g., infusion reaction) that, in the judgment of the Investigator, could place the participant at increased risk, study drug administration should be discontinued.

Management of Possible Acute Hypersensitivity Reactions to Abatacept

Hypersensitivity or acute allergic reactions may occur as a result of the protein nature of abatacept. Should any of these reactions occur during the course of the study, they need to be reported as specified in 8.3.6 (Adverse Events). In this study, participants' vital signs will be monitored before and following study drug administration. Appropriate emergency equipment and qualified personnel should be available where the participants are treated in the event of a serious anaphylactic reaction.

The following information is provided to assist in the recognition of hypersensitivity reactions and in the management of those reactions should they occur during or after the administration of abatacept. Care should be taken to treat any acute toxicities expeditiously, should they occur. When IV dosing of abatacept is conducted, equipment such as a portable tank or wall-source of oxygen, endotracheal intubation set, oral airway, mask, ambu-bag, syringes, injectable epinephrine, injectable antihistamine, and injectable corticosteroids should be kept in the vicinity where the participant is treated.

Signs and management of potential acute hypersensitivity reactions include:

a) Symptomatic Hypotension should be managed by discontinuing the infusion of study medication, placing the participant in the Trendelenburg position and administering intravenous fluid. Additional medical intervention may also include the use of epinephrine, corticosteroids, antihistamines and pressor agents.
b) Dyspnea should be managed by discontinuing the infusion of study medication and observing the participant for worsening of the event and for the appearance of additional signs and symptoms of anaphylaxis. Antihistamines, epinephrine and corticosteroids may be administered as indicated.
c) Acute pain in the chest, back or extremities may also be a sign of anaphylaxis and may be treated as described above for dyspnea
d) Chills, fever, urticaria or generalized erythema may all be signs of an allergic reaction to protein products. Such signs and symptoms may be treated with acetaminophen and antihistamines.

The decision of whether to complete the infusion of study medication if symptoms improve or have resolved will be left to the medical judgment of the Investigator.

7.1.5. *Overdosage*

Doses of Abatacept up to 50 mg/kg have been administered intravenously without apparent toxic effect. In the case of overdosage, it is recommended that patients should be monitored for any signs or symptoms of adverse reactions and have the appropriate symptomatic treatment instituted immediately.

7.1.6. *Preparation/Handling/Storage/Accountability*

7.1.6.1. *Acquisition and Accountability*

Investigational products (IP) will be shipped to the site either directly from participating companies, from the Sponsor, or from other regional or local drug repositories. All other supplies should be provided by the site. Multiple lots of each IP may be supplied.

Study products received at the sites will be open label and not kit specific, unless specified in the protocol-specific Manual of Procedures (MOP) or pharmacy manual. Drug preparation will be performed by the participating site's unblinded research pharmacist on the same day of administration to the participant. See the MOP Appendices for detailed information on the preparation, labeling, storage, and administration of investigational products.

Accountability:

The site PI is responsible for study product distribution and disposition and has ultimate responsibility for study product accountability. The site PI may delegate to the participating site's research pharmacist responsibility for study product accountability. The participating site's research pharmacist will be responsible for maintaining complete records and documentation of study product receipt, accountability, dispensation, storage conditions, and final disposition of the study product(s). Time of study drug administration to the participant will be recorded on the appropriate data collection form (CRF). All study product(s), whether administered or not, must be documented on the appropriate study product accountability record or dispensing log. The Sponsor's monitoring staff will verify the participating site's study product accountability records and dispensing logs per the site monitoring plan. Refer to the protocol-specific MOP for details on storing study medications.

Destruction:

After the study treatment period has ended or as appropriate over the course of the study after study product accountability has been performed, used active and placebo product can be destroyed on-site following applicable site procedures with a second staff member observing and verifying the destruction.

Unused product at the end of the study should be saved until instructed by the Sponsor.

7.1.6.2. *Formulation, Appearance, Packaging, and Labeling*

Abatacept component:
Abatacept for Injection is a lyophilized powder for intravenous infusion. ORENCIA for Injection is supplied as a sterile, white, preservative-free, lyophilized powder for reconstitution and dilution prior to intravenous administration. Following reconstitution of the lyophilized powder with 10 mL of Sterile Water for Injection, USP, the solution of abatacept is clear, colorless to pale yellow, with a pH range of 7.2 to 7.8. Each single-use vial of abatacept for Injection provides 250 mg abatacept, maltose (500 mg), monobasic sodium phosphate (17.2 mg), and sodium chloride (14.6 mg) for administration. It is supplied as an individually packaged, single-use vial with a silicone-free disposable syringe, providing 250 mg of abatacept in a 15-mL vial.

Injectable placebo component:
Injectable placebo will be normal IV saline (0.9% NaCl) made up unblinded by the resident pharmacist and blinded to the study team. Note that the type of infusion bag used for placebo should be identical to that used for the preparation of the abatacept infusion in order to ensure the blind in the study.

7.1.6.3. *Product Storage and Stability*

Abatacept should be stored at 2ºC-8ºC and protected from light.

7.1.6.4. *Preparation*

Refer to the protocol-specific MOP for details about preparation.

Use aseptic technique.
abatacept for Injection is provided as a lyophilized powder in preservative-free, single-use vials. Each ORENCIA vial provides 250 mg of abatacept for administration. The abatacept powder in each vial must be reconstituted with 10 mL of Sterile Water for Injection, USP, using *only the silicone-free disposable syringe provided with each vial* and an 18- to 21-gauge needle. After reconstitution, the concentration of abatacept in the vial will be 25 mg/mL. If the abatacept powder is accidentally reconstituted using a siliconized syringe, the solution may develop a few translucent particles. Discard any solutions prepared using siliconized syringes.

If the *silicone-free disposable syringe* is dropped or becomes contaminated, use a new *silicone-free disposable syringe* from inventory.

1) Use 10 mL of Sterile Water for Injection, USP to reconstitute the abatacept powder. To reconstitute the abatacept powder, remove the flip-top from the vial and wipe the top with an alcohol swab. Insert the syringe needle into the vial through the center of the rubber stopper and direct the stream of Sterile Water for Injection, USP, to the glass wall of the vial. Do not use the vial if the vacuum is not present. Rotate the vial with gentle swirling to minimize foam formation, until the contents are completely dissolved. Do not shake. Avoid prolonged or vigorous agitation.
2) Upon complete dissolution of the lyophilized powder, the vial should be vented with a needle to dissipate any foam that may be present. After reconstitution, each milliliter will contain 25 mg (250 mg/10 mL). The solution should be clear and colorless to pale yellow. Do not use if opaque particles, discoloration, or other foreign particles are present.
3) The reconstituted abatacept solution must be further diluted to 100 mL as follows. From a 100 mL infusion bag or bottle, withdraw a volume of 0.9% Sodium Chloride Injection, USP, equal to the volume of the reconstituted abatacept solution required for the patient’s dose. Slowly add the reconstituted abatacept solution into the infusion bag or bottle using the same *silicone-free disposable syringe provided with each vial*. Gently mix. *Do not shake the bag or bottle*. The final concentration of abatacept in the bag or bottle will depend upon the amount of drug added but will be no more than 10 mg/mL. Any unused portions in the abatacept vial must be immediately discarded.
4) Prior to administration, the abatacept solution should be inspected visually for particulate matter and discoloration. Discard the solution if any particulate matter or discoloration is observed.

5) The entire, fully diluted abatacept solution should be administered over a period of 30 minutes and must be administered with an infusion set and a *sterile, non-pyrogenic, low-protein-binding filter* (pore size of 0.2 µm to 1.2 µm).
6) The infusion of the fully diluted abatacept solution must be completed within 24 hours of reconstitution of the ORENCIA vials. The fully diluted abatacept solution may be stored at room temperature or refrigerated at 2ºC to 8ºC (36ºF to 46ºF) before use. Discard the fully diluted solution if not administered within 24 hours.
7) Abatacept should not be infused concomitantly in the same intravenous line with other agents. No physical or biochemical compatibility studies have been conducted to evaluate the coadministration of abatacept with other agents.

7.2. Measures to Minimize Bias: Randomization and Blinding

Randomization will be conducted as specified in the Master Protocol.

7.3. Study Intervention Compliance

Each dose of study product will be administered by a member of the clinical research team who is qualified and licensed to administer the study product. Administration and date, and time, will be entered into the case report form (CRF).

7.4. Concomitant Therapy

7.4.1. *Permitted Concomitant Therapy and Procedures*

All concomitant therapies permitted in the master protocol will be allowed for this sub-study.

Of specific note to cotreatment with corticosteroids, Orencia is routinely co-administered with low dose corticosteroids (CS, prednisone or equivalent ≤ 10 mg/day) in its labeled indications for the treatment of Rheumatoid Arthritis, Psoriatic Arthritis and Juvenile Idiopathic Arthritis and is not associated with increased risk of adverse outcomes. Concomitant use of abatacept with higher doses of CS has been studied in other disease areas, e.g. lupus nephritis. Based on clinical trial data, use of high dose CS with sustained abatacept treatment may be associated with some increased risk for serious bacterial infections.

7.4.2. *Prohibited Concomitant Therapy*

All concomitant therapies prohibited in the master protocol will also be prohibited for this sub-study.

7.4.3. *Rescue Medicine*

Not Applicable.

7.4.4. *Non-Research Standard of Care*

Not Applicable.

8. STUDY INTERVENTION DISCONTINUATION AND SUBJECT DISCONTINUATION/WITHDRAWAL

8.1. Halting Criteria and Discontinuation of Study Intervention

8.1.1. *Individual Study Product Halting*

Individual study product halting will be managed according to the Master Protocol.

Abatacept Halting

If there is evidence of toxicity (e.g., infusion reaction) that, in the judgment of the Investigator, could place the participant at increased risk, study drug administration should be discontinued.

8.1.2. *Study Halting*

Study halting will be managed as specified by the Master Protocol.

8.2. Withdrawal from the Study

Sub-study will have the same requirements as the main study.

8.3. Lost to Follow-Up

Sub-study will have the same requirements as the main study.

8.4. Readmission

Sub-study will have the same requirements as the main study.

9. STUDY ASSESSMENTS AND PROCEDURES

Sub-study will have the same assessments and procedures as the main protocol.

10. STATISTICAL CONSIDERATIONS

Statistical analysis will be performed as described within the main body of the ACTIV-1 IM master protocol.

APPENDIX 3 – SUB-STUDY 3 – CENICRIVIROC (CVC)

1. SUB-STUDY SUMMARY

1.1. Sub-study overview

The sub-study population corresponds to moderately and severely ill patients infected with the COVID-19 virus. Recruitment will target patients already hospitalized for treatment of COVID-19 infection as well as patients being treated for COVID-19 infection in Emergency Departments while waiting to be admitted to the hospital. Patients both in and out of the ICU are included in the study population.

Enrollment will begin as soon as all elements are met for the sub-study to activate.

The time on drug period is 28 days (i.e., up to Day 28 of the study), with assessments on each day of the hospital stay. Patients will be followed after hospital discharge and after last dose of drug, with periodic follow-up assessments through Day 60 (i.e., approximately 30 days after last dose of drug).

1.2. Enrollment Period

Enrollment began in October 2020. It is anticipated that enrollment will be completed in 4-6 months.

1.3. General

This sub-study is designed to evaluate cenicriviroc (CVC) for the treatment of moderately or severely ill hospitalized patients infected with COVID-19. Sub-study will have the same assessments and procedures as the main protocol.

The effectiveness of CVC as add-on therapy to SoC will be evaluated based on the primary endpoint of time to recovery after 28 days. The sample size requirements are based on the ability to detect a moderate improvement in time to recovery (3-4 fewer days).

1.4. Study Population

Hospitalized adults (≥18 years old) with COVID-19, including patients both in and out of the ICU. Patients seeking care for COVID-19 in an Emergency Department (ED) and waiting to be admitted to the hospital are included.

1.5. Inclusion Criteria

Inclusion criteria are outlined in the main body of the ACTIV-1 IM protocol.

1.6. Agent Specific Exclusion Criteria

Use of medications that are contraindicated with CVC and that could not be replaced or stopped during the study period (See **Appendix 3 Table 3** for Disallowed Medications).

1.7. Study Intervention

Therapeutic agents will be evaluated as an add-on therapy to SoC. The study intervention is as outlined in the main body of the ACTIV-1 IM protocol.

For the cenicriviroc (CVC) component, CVC will be administered with food as a 450 mg oral loading dose on Day 1, followed by a 300 mg (150 mg BID) maintenance dose for the duration of the study (through Day 28). The administration window of the loading dose will be within 24 hours of randomization.

Duration of therapy:

- Cenicriviroc Oral component – 28 days, including while hospitalized and post-discharge.

1.8. Schedule of Assessments

Assessments will be performed according to **Table 1-1** in 1.1.8 in the main body of the ACTIV master protocol.

2. JUSTIFICATION FOR SELECTION

Severe pneumonia and respiratory-tree infection caused by coronaviruses such as SARS-CoV and MERS-CoV are often associated with massive inflammatory cell infiltration and elevated pro-inflammatory cytokine and chemokine responses -- resulting in injury to the respiratory tree and an acute respiratory distress syndrome (ARDS). Patients with this cytokine storm have been observed to have high levels of CCL2 (MCP1) and CCL5 (RANTES), likely caused by feedback from SARS-CoV upregulating CCR2 and CCR5 (Law et al. 2009). Elevated CCL5 may lead to severe lung inflammation, ARDS, and death in patients with SARS (Ng et al. 2007) demonstrated that the RANTES-28 G allele, which is associated with high levels of CCL5, was a risk factor for poor outcomes and death in Hong Kong and Beijing Chinese SARS patients. This study noted that these chemokines, released from activated immune cells, not only take part in the anti-viral immune response but are causative of cell damage and organ dysfunction. Further demonstrated was the significant induction of CCR-1, CCR-3 and CCR-5 mRNA expression in SARS-CoV infected dendritic cells (DCs), suggesting the possibility of an autocrine loop facilitating DC trafficking. Moreover, presence of the CCL2 G-2518A allele, which leads to high CCL2 levels, made patients more susceptible to infection with SARS-CoV (Tu et al. 2015). Thus, clinical data demonstrate that both CCL5 and CCL2 play key roles in the inflammatory sequelae and poor clinical outcomes of SARS-CoV infection.

In patients with ARDS, CCL2 is upregulated, inducing the migration of circulating CCR2+ inflammatory cells into alveoli (Zemans and Matthay 2017). Patients with ARDS have elevated levels of CCL2 in BAL fluid compared with controls and greater neutrophil recruitment (Williams et al. 2017).

Rationale for dual targeting of CCR2 and CCR5

Both CCR2 and CCR5 play roles in ARDS and the consequences of SARS-CoV. Rationale of dual inhibition includes: redundancy among these pathways and dual inhibition preventing a spill over that may occur with blocking only one; and, these two routes have complementary roles in infection-associated inflammation and tissue damage. For example, CCR2 targeted action is more critical for inhibition of circulating immune cells (e.g., monocytes, memory T cells), their trafficking, and migration into tissue; CCR5 action is more related to inhibiting tissue-based immune cells, and therefore a somewhat later time window (e.g., macrophages, activated T effector cells, dendritic cells). Both CCR2/5 paths have been shown to be operative, and effects off-set by attenuation of CCR2/5 has been shown to be operative in: CoV infection; ARDS; and influenza-caused lung inflammation, tissue destruction, and fibrosis. Moreover, specifically with CVC, dual CCR2/5 has been shown to be an effective mechanism in pre-clinical models of liver inflammation/fibrosis and viral encephalitis.

Importantly, shown in a murine model of SARS-CoV infection, there are 2 time windows: 1) before the onset or worsening of pneumonitis when the administration of a CCR2 inhibitor has the highest likelihood of being beneficial to patients with COVID-19 and 2) during the pneumonitis when both CCR2 and CCR5 inhibition may be beneficial, reinforcing the potential role for dual targeting of CCR2/5 (**Appendix 3 Figure 1**) (Chen et al. 2010).

Appendix 3 Figure 1: ***Pathogenesis of SARS-CoV Infection in Senescent Mice***

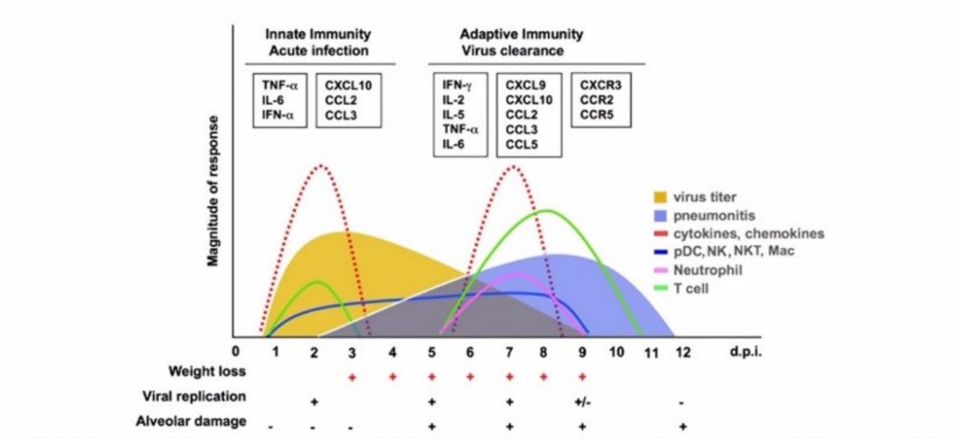

FIG. 7. Time course of host responses to primary SARS-CoV infection in senescent mice. A biphasic expression of inflammatory mediators associated with cellular infiltration into the lungs of infected mice, coincident with peaks in viral replication and clearance, respectively, is seen. Clinical illness such as weight loss was observed coincident with pulmonary viral replication, while lung pathology (pneumonitis) was associated with T-cell infiltration when virus was cleared from the lung.

Cenicriviroc (CVC) is a novel small-molecule, orally active, well-tolerated, potent and selective antagonist of CCR2 and CCR5 with anti-inflammatory and anti-fibrotic activity. Data from animal and clinical studies support a favorable action of CVC in inflammation associated with severe infection. In a mouse model of acute liver injury, administration of CVC significantly decreased the numbers of monocyte-derived macrophages and associated inflammation and tissue damage. CVC also reduced the numbers of Kupffer cells or tissue-based macrophages with a KC-like phenotype peri-injury. Other immune cell populations such as neutrophils or lymphoid cells were not affected (Puengel, Krenkel et al. 2017). In a 2nd mouse model of liver injury, CCR2 inhibition with CVC reduced the influx of proinflammatory monocytes into the liver, which significantly attenuated the early phase of tissue injury and later necrosis (Mossanen et al. 2016). Of note, lung disease models demonstrating infiltrating monocyte-derived interstitial macrophages have a similar inflammatory phenotype, including CCR2/5 based processes, as in the liver disease models, and alveolar macrophages share multiple features with Kupffer cells in the liver. Moreover, immunomodulatory action from CVC has been shown to result in reductions of inflammation and in tissue preservation in murine models of viral encephalitis.

In an ex-vivo study of monocytes derived from HIV-infected patients, dual CCR2/5 inhibition with CVC was more effective in decreasing trans-endothelial migration of monocytes than single CCR2 or CCR5 blockade. Moreover, CVC was also associated with decreased expression of E-selectin on endothelial cells, a major receptor for monocyte recruitment (D'Antoni et al. 2018).

Appendix 3 Figure 2: ***Mechanism of Action for Cenicriviroc (CVC)***

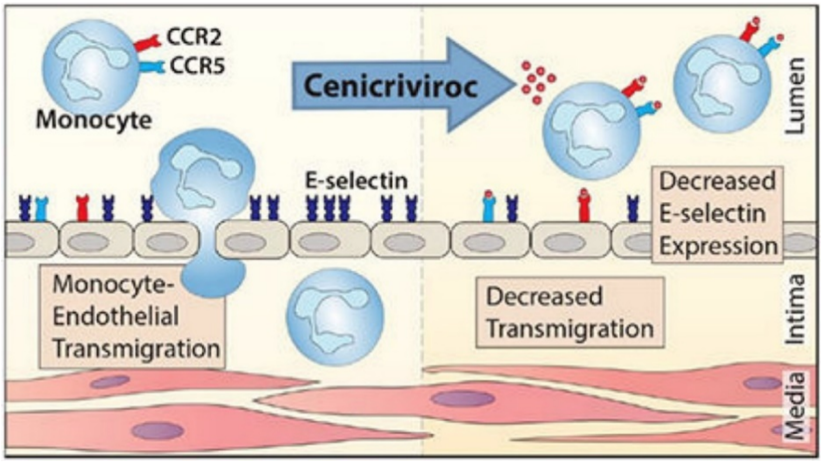

We hypothesize that CVC, by blocking CCR2 and CCR5, will decrease recruitment of infiltrating monocyte-derived interstitial macrophages and other immune cells, known to be operative in respiratory tissues in coronavirus infections, thus preventing the cytokine storm, respiratory injury, and ARDS with COVID-19 (Mossanen et al. 2016; Puengel et al. 2017). Also, we hypothesize that the reduction of trans-endothelial vascular migration of monocytes could play a role in attenuating vascular-based injury. Therefore, we believe that treatment with CVC could prevent the cytokine storm that cause the lung injury and is the cause of acute respiratory distress syndrome (ARDS) in patients with COVID-19.

We propose the investigation of administration of CVC in patients hospitalized due to COVID-19, in those who have not yet developed severe respiratory disease or are early in a course of respiratory compromise, and in those with substantial respiratory compromise, with the objective of reducing the risk of evolution to severe lung injury. The documented safety profile allows the potentially broad treatment of patients, e.g. at several stages of COVID-19.

3. INTRODUCTION

3.1. Risk/Benefit Assessment

3.1.1. *Cenicriviroc*

3.1.1.1. *Potential Risks of Cenicriviroc*

Cenicriviroc (CVC) is contraindicated in participants with clinically significant hypersensitivity to any components of its formulation.

Warnings and Precautions

Treatment-emergent elevations in liver transaminases have been observed in participants receiving CVC treatment. Most elevations were transient, mild to moderate in severity, and resolved upon continued treatment with CVC. Some elevations were severe in intensity and resulted in dose interruption or permanent discontinuation of CVC. CVC has been administered to >2000 patients, including patients with non-alcoholic fatty liver disease (NASH) and a separately to those with HIV, in clinical trials. In NASH patients with pre-existing liver disease, 2 cases of possible autoimmune hepatitis (1 participant in the CVC arm and 1 participant in the placebo arm) have been observed.

Hepatic and Renal Impairment

Based on clinical data (studies 652-1-121 and 3152-102-002), CVC exposures did not increase in participants with mild hepatic impairment (Child-Pugh A), increased in participants with moderate (Child-Pugh B) (AUC0-tau 55%), and significantly increased in severe (Child-Pugh C) (AUC0-t 40%) hepatic impairment relative to corresponding matched healthy participants. These clinical studies allowed inclusion for ALT or AST abnormalities up to 5x ULN. Of note: based on the PK and safety results for these studies, no CVC dose adjustment will be required in patients with hepatic impairment. Based on results from the human ADME study (Study 3152-103-002), renal excretion of CVC and its metabolites was negligible (< 2% of dose). Consistent with this observation, the renal excretion of CVC (non-radiolabel study) was observed to be < 0.00011% (Study 01-03-TL-652-001). The impact of end stage renal disease on the PK of CVC is under evaluation (Study 3152-104-002).

Drug Interactions

CVC is a substrate of CYP3A4, CYP2C8 and P-gp. CVC is also a weak inhibitor of CYP3A4 and an inhibitor of BCRP and P-gp. The following classes of medications are disallowed at any time during CVC treatment:

- Potent CYP3A4 inhibitors and CYP3A4 inducers will be excluded
- Potent CYP2C8 inhibitors will be excluded
- Drugs with narrow therapeutic windows that are sensitive CYP3A4 substrates will be excluded (i.e., drugs which should not be co-administered with weak CYP3A4 inhibitors such as CVC)
- Appropriate dose adjustments of BCRP substrates are recommended

Co-administration of CVC and remdesivir is allowed.

Co-administration of CVC and glucocorticoids is allowed.

When required, acid-reducing agents should be administered at least 2 hours after the CVC (4 hours for fast-acting antacids). It is recommended to use an H2 receptor antagonist (except cimetidine) or antacids rather than a proton pump inhibitor. It is recommended to start with the lowest dose of these agents and titrate according to clinical response. If lipid lowering medications are used, clinical monitoring and dose titration are recommended to achieve the desired clinical response.

See **Appendix 3 Table 3** and **Appendix 3 Table 4** for fuller instructions regarding allowed concomitant drugs.

In summary, the risks of CVC are low and similar to placebo.

3.1.1.2. *Potential Benefits of Cenicriviroc*

CVC may or may not improve clinical outcome of an individual trial participant with COVID-19. However, there is potential benefit to society from their participation in this study resulting from insights gained about the CVC under study as well as the natural history of the disease. While there may not be benefits for an individual participant, there may be benefits to society if a safe, efficacious therapeutic agent can be identified during this global COVID-19 outbreak.

3.1.1.3. *Assessment of Potential Risks and Benefits of Cenicriviroc*

CVC is generally a well-tolerated medication, which has been administered to participants up to 2 years at different doses. It is well tolerated in a wide dose range. There are no significant toxicities that have been observed in prior studies. An increase of nasopharyngitis in the second year of administration without an increase in upper respiratory tract infections has been reported from a long-term exposure trial (2 years). Since the drug is administered for only 28 days we do not expect any of those side effects. The loading dose of 450 mg on day one will be split into two doses (300 mg (AM) and 150 mg (PM)); note: participants enrolled in the study in the latter portion of the day may receive only the 300 mg component of the Day 1 loading dose (see **Appendix 3 Table 2** for specific dosing scenarios). Additional side effects are not expected. Clinical and pharmacologic assessments support this dose, and supportive data exist for significantly higher doses.

4. OBJECTIVES AND ENDPOINTS

The overall objective of the study is to evaluate the clinical efficacy and safety of different investigational therapeutics relative to the control arm among hospitalized adults who have COVID-19. All the endpoints for the overall trial are defined in the body of the main ACTIV-1 IM protocol.

5. STUDY DESIGN

5.1. Justification for Dose

5.1.1. *Justification for Dose of Cenicriviroc*

To ensure rapid onset of action of CVC, a loading dose of 450 mg will be administered on Day 1 (300 mg first dose in the morning and 150 mg 12 hours later); patients enrolled in the trial in the latter portion of the day may receive only the 300 mg component of the Day 1 loading dose (see **Appendix 3 Table 2** for details on dosing scenarios). Starting Day 2, patients will receive 150 mg of CVC twice per day (total dose of 300 mg per day) through Day 28.

Prior clinical data, clinical pharmacological data, and PK/PD modelling have indicated that CVC inhibited CCR2 (measured as MCP-1 increase) and CCR5 (measured as MIP-1b increase) with an IC_{50} of 25 ng/mL and 43 ng/mL, respectively (3152-102-002: ACCP poster, 2019). This model predicts maximal increase in MCP-1 (~6-fold) and MIP-1b (~2-fold) achieved at steady state by once daily dosing of 150 mg CVC, which reflects complete receptor occupancy. In order to achieve complete receptor occupancy at an earlier time, a loading dose of 300 mg CVC in the morning and 150 mg CVC after 12 hours will be administered: patients enrolled in the trial in the latter portion of the day may receive only the 300 mg component of the Day 1 loading dose (see **Appendix 3 Table 2** for details on dosing scenarios). This dosing regimen predicts achievement of ~90% receptor occupancy by end of Day 1. Thereafter, continued dosing of 150 mg CVC twice daily is expected to maintain effective receptor occupancy for the duration of the treatment.

Moreover, the total dose of 450 mg CVC on Day 1 (split into 300 mg and 150 mg; see **Appendix 3 Table 2** for details on dosing scenarios) is justified based on clinical trials in which multiple doses of CVC up to 900 mg QD for 7 days were administered to healthy volunteers with no safety findings observed. Furthermore, other safety margin data also give support for this dosing regimen. In GLP toxicology studies, the NOAEL was determined to be 2000 mg/kg/day in mice, 100 mg/kg/day in rats, and 1000 mg/kg/day in monkeys. Based on the most sensitive NOAEL dose of 100 mg/kg/day in rats, a HED of 960 mg was estimated. The most commonly observed AEs (≥ 2%) with CVC in multiple-dose studies evaluating daily doses up to 900 mg for ≥ 7 days were headache and constipation.

Furthermore, CVC has been shown to be safe and well-tolerated following multiple-dose administration of 450 mg and 900 mg once daily for 7 days (Study 3152-107-002). In study 3152-107-002, steady state C_{max} of CVC at doses of 450 mg and 900 mg was 2977 ng/mL and 4025 ng/mL, respectively, and the corresponding $AUC_{0-\tau}$ values were 37724 and 72905 ng.h/mL, respectively. Since the Day 1 dose of 450 mg in the current study is being split (as 300 mg in the morning and 150 mg to be administered after 12 hours), and from day 2 onwards the dose of 300 mg is split (as 150 mg BID), the maximal CVC concentrations (Cmax) as well as steady state AUC (AUC_t) in this study are expected to be less than those observed in earlier clinical studies (3152-107-002 and 3152-106-002). Note: patients enrolled in the trial in the latter portion of the day may receive only the 300 mg component of the Day 1 loading dose (see **Appendix 3 Table 2** for details on dosing scenarios).

Since food has been shown to have significant effect on CVC exposure, all doses are recommended to be administered within 30 minutes of consumption of a meal (or feeds), which is also how CVC has been administered in other clinical trials.

Pharmacokinetic Modelling Assessments for Dose Rationale

CVC is a dual antagonist of CCR2 and CCR5 receptors. Increase in serum concentrations of ligands of CCR2 (MCP-1) and CCR5 (MIP-1β) is used as a surrogate marker (biomarker response) to determine CVC receptor occupancy in clinical studies. In an earlier Phase 1 clinical study (Study 3152-105-002), exposure response relationship between plasma CVC concentrations and biomarker response (MCP-1 or MIP-1β) were explored using

a sequential PK/PD model (**Appendix 3 Figure 3**). The structural PK model was a two-compartment model with first-order absorption and with a lag time. The exposure response for each biomarker was modelled by an indirect pharmacodynamic response model. The kinetic parameter estimates from the indirect response model are summarized in **Appendix 3 Table 1**.

Appendix 3 Figure 3: ***Sequential approach of population PK and Indirect response model utilized to determine exposure response***

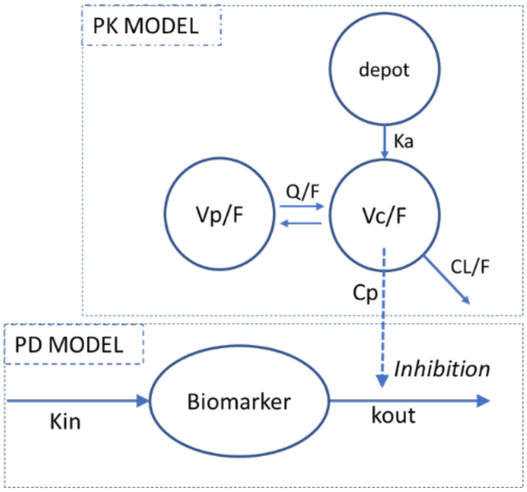

Appendix 3 Table 1: ***Parameter Estimates for Indirect Response Model***

	MCP-1 [pg/mL]			MIP-1B [pg/mL]		
	Typical Value	RSE(%)	IIV	Typical Value	RSE(%)	IIV
Baseline [pg/mL]	339	3.8	18%	98	7.8	38%
kout [1/h]	0.60	fixed	n.e.	0.055	9.8	n.e.
Imax [-]	0.90	fixed	n.e.	0.62	fixed	n.e.
IC50 [ng/mL]	25	63	78%	43	31	117%
Proportional Residual Error	19%	12	n.e.	12%	5.7	n.e.

RSE – relative standard error for estimated typical value; IIV – inter-individual variability; n.e. – not estimated; Kin=Baseline*kout; %inhibition of kout = Cp*Imax/(Cp+IC50); "fixed" - to aid a stable estimation, some parameter values were fixed in final model based on values obtained in iterative model runs and evaluated through numerical and visual goodness-of-fit.

Based on these parameters, the biomarker response (% change from baseline of MCP-1 or MIP-1β) was predicted following once daily dosing of 150 mg CVC (**Appendix 3 Figure 4)**. These simulations suggest maximal change in MCP-1 levels (~5.5-fold increase) and MIP-1β (~2-fold) upon once daily dosing of CVC for at least 5 days and 10 days, respectively.

Appendix 3 Figure 4: ***Predicted biomarker response following once daily dosing of 150 mg CVC***

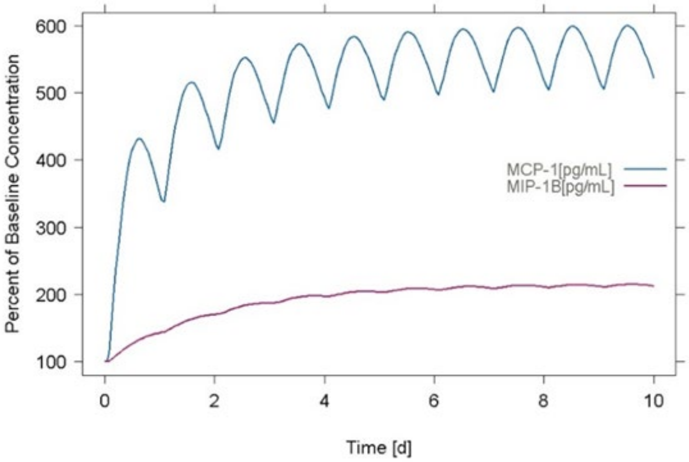

Since treatment of fibrosis in NASH patients require chronic administration of CVC (AURORA study duration: 5 years of once daily treatment of 150 mg CVC) and the earliest change in endpoint (reduction in fibrosis) is expected after once daily treatment for 1 year, achieving maximal biomarker change after 5 to 10 days of once daily treatment is acceptable in NASH indication. However, COVID-19 is a severe acute infectious disease that requires rapid onset of action and relatively short duration of treatment (planned duration of treatment 28 days). Therefore, dosing regimen containing a total loading dose of 450 mg CVC (divided as 300 mg in the morning + 150 mg in the evening) was proposed on Day 1 of this study followed by dose of 300 mg/day (divided as 150 mg in the morning plus 150 mg in the evening). For the proposed dosing regimen, the biomarker response (% change from baseline of MCP-1 or MIP-1β) was predicted as shown (**Appendix 3 Figure 5**). As per simulations, loading dose is expected to increase MCP-1 levels by 6-fold and MIP-1β levels by 1.5-fold on Day 1. Following twice-daily administration of 150 mg CVC, maximal increase in MCP-1 (~7-fold) and MIP-1β (~2-fold) were sustained during the duration of treatment beginning Day 2. These simulations suggest effective blockade of activation of CCR2 and CCR5 receptors by respective ligands could be achieved rapidly and sustained by following the proposed dosing regimen.

Appendix 3 Figure 5: ***Predicted biomarker response following proposed dosing regimen of CVC (Day 1: 300 mg in the morning + 150 mg in the evening, Day 2 to Day 29: 150 mg twice daily)***

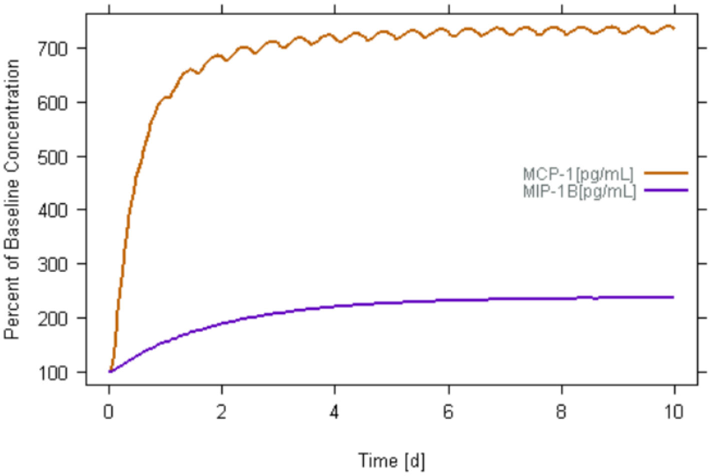

As of January 2020, approximately 2000 participants have been exposed to either single or multiple doses of CVC in completed and ongoing clinical studies. CVC doses explored in these clinical studies range from 25 mg to 900 mg

across all CVC studies. There was no apparent dose- or exposure-relationship for safety observed. Furthermore, splitting the daily dose is expected to limit the plasma peak concentrations of CVC corresponding to 300 mg dose on Day 1 and 150 mg dose from Day 2 onwards. In addition, food effect on the bioavailability of CVC formulation is significant (up to 5.2-fold increase with standard breakfast). The CVC dose can be administered to an intubated and severely ill COVID-19 patient via G-tube and with liquid food. In such a case, if there were to be reduced enteral absorption, then any lowered plasma concentrations/exposure of CVC may be partially offset by the twice-daily dosing of CVC.

In summary, these data provide support and rationale for the proposed dosing being well within appropriate parameters and safety. The sponsor believes the proposed loading dose (450 mg on Day 1) and twice daily administration of 150 mg CVC are clearly justified based on the following: safe use of CVC doses up to 900 mg in prior clinical studies; immediate need for optimal plasma exposure of CVC in patients with COVID-19 infection, ensure full antagonistic activity against CCR2 and CCR5 receptors in target tissues; and maintenance of such exposure throughout the duration of treatment without compromising patient safety.

6. STUDY POPULATION

The study population will adhere to the master protocol population, except for the noted additional exclusions and considerations below.

6.1. Inclusion Criteria

The same as the master protocol.

6.2. Sub-Study Specific Exclusion Criteria

Use of medications that are contraindicated with CVC and that could not be replaced or stopped during the study period (See **Appendix 3 Table 3** for Disallowed Medications).

6.3. Exclusion of Specific Populations

Children and adolescents will not be included in this trial.

No data currently exist for the effects of CVC on reproduction and development in humans. Pregnancy should be avoided in women receiving this compound. Women of childbearing potential should undergo pregnancy testing prior to initiation of CVC and periodically during treatment. In addition, adequate contraceptive methods should be consistently used by males and females during and for 1 month after treatment with CVC. Pregnancies in both female participants and in partners of male participants should be reported to the PI.

CVC is contraindicated in nursing mothers. CVC and its metabolites were detected in milk secreted from rats administered CVC. CVC milk to plasma ratios ranged from 0.7 to 0.8 in dams indicating that milk represents a potential route of exposure to CVC in rat pups. The extent to which CVC or any of its metabolites are excreted in human breast milk is not known.

7. STUDY PRODUCT

7.1. Study Product(s) and Administration

Participants will be randomized according to the procedure described in the master protocol.

If there are supply limitations on any product, the arms containing that product will be temporarily closed to enrollment and the corresponding placebo is not needed.

7.1.1. *Study Product Description*

The CVC drug product is a solid oral dosage (tablet form) containing 150 mg of the active ingredient as a free base equivalent. The CVC 150 mg tablets are capsule-shaped, 15.7 × 7.9 mm in diameter and plain on both sides. The inactive ingredients of CVC tablets include fumaric acid, microcrystalline cellulose, croscarmellose sodium, colloidal silicon dioxide and magnesium stearate, which is film coated with an Opadry II yellow, a polyethylene glycol (PEG) and partially hydrolyzed polyvinyl alcohol (PVA) non-functional film coat.

7.1.2. *Dosing and Administration*

For the CVC component, treatment will be administered to participants while hospitalized as in-patients and will continue whether or not the patient is discharged from the hospital for a total 28-day course. CVC should be administered twice daily at approximately 12-hour intervals in fed condition and at approximately the same time each day (±2 hours). Patients may receive a larger dose (450 mg total) for their first day of treatment as a loading dose administered as a morning dose of 300 mg and an evening dose of 150 mg. The administration window will be within 24 hours of randomization. See **Appendix 3 Table 2** for further details.

Doses should be administered with food (or tube-feeds). For participants requiring mechanical ventilation, Investigators will need to determine the most appropriate means for providing the drug product with food (i.e., crushed tablet by feeding tube with adequate flush); preparation instructions for administration of CVC using a G-tube or other enteral feeding tube are in the Pharmacy Manual. If a feeding tube is not available for >24 hours, the participant will discontinue study drug intervention, but will remain in the study for endpoints and safety observations.

Appendix 3 Table 2: Dosing of CVC Depending on Time of Day of Randomization (Scenarios)

	**Scenario 1**		**Scenario 2**		**Scenario 3**	
	Participant is randomized and entered into the system for first-dose of Day 1 with breakfast		Participant is randomized and entered into the system for first-dose of Day 1 with lunch or early afternoon snack/meal		Participant is randomized and entered into the system for first-dose of Day 1 with dinner; 2nd dose of Day 1 is N/A	
BID Dose	*Dose 1*	*Dose 2*	*Dose 1*	*Dose 2*	*Dose 1*	*Dose 2*
**Day 1 (Loading Dose, mg)**	*300*	*150*	*300*	*150*	*300*	N/A
Time of dosing	8:00 AM	8:00 PM	11:30 AM	11:30 PM	6:00 PM	N/A
Acceptable Time Range	6 – 10 AM	6 – 10 PM	9:30 AM – 1:30 PM	9:30 PM – 1:30 AM	4 – 8 PM	N/A
**Days 2-28 (Dose, mg)**	*150*	*150*	*150*	*150*	*150*	*150*
Time of dosing	8:00 AM	8:00 PM	8:00 AM	8:00 PM	8:00 AM	8:00 PM
Acceptable Time Range	6 – 10 AM	6 – 10 PM	6 – 10 AM	6 – 10 PM	6 – 10 AM	6 – 10 PM

Any dose that is delayed may be given later that calendar day. Any dose that is missed (not given that calendar day) is not made up. The treatment course continues as described above even if the participant becomes PCR negative.

Duration of therapy:

- Cenicriviroc (Oral) – 28 days (i.e., up to Day 28 of study), including while hospitalized and post-discharge.

7.1.3. *Dose Escalation*

Not Applicable

7.1.4. *Dose Modifications*

See Table in section 7.4.2, Allowed Medications Requiring Dose Adjustments.

7.1.4.1. *Dose Modifications for Adverse Events*

If there is evidence of toxicity that, in the judgment of the Investigator, could place the participant at increased risk, study drug administration should be interrupted. Resumption of dosing should be discussed with the Sponsor.

Any potential dose modifications of CVC need to be discussed with the Sponsor.

Liver Assessment: Suggested Actions and Follow-up

Close monitoring should be initiated for the following participants:

- Participants enrolled with a normal baseline serum aminotransferases (AT) who develop an increase of serum AT ≥3 × ULN, or
- Participants enrolled with elevated baseline AT who develop an increase of serum AT >2 × the baseline value

The participant should be evaluated for liver biochemistry elevation potentially meeting Hy's law criteria (see below) as soon as possible, preferably within 24 to 48 hours from the time the investigator becomes aware of the abnormal results. Evaluation should typically include repeat testing of all 4 of the usual serum biochemical measures (ALT, AST, ALP, and TBL) to confirm the abnormalities and to determine if they are increasing or decreasing. Testing should be repeated until the levels decrease or stabilize.

If it is difficult for the participant to return to the study site promptly, the participant should be retested locally, but normal laboratory ranges should be recorded, results should be made available to sponsor's study physician and the investigator immediately, and the data should be included in the eCRF. If repeat testing within this time frame is not possible, the study intervention should be discontinued.

It is critical to initiate close monitoring immediately upon detection and confirmation of signals of liver biochemistry elevation potentially meeting Hy's law criteria as early as possible and not to wait until the next scheduled visit or monitoring interval. Close monitoring of the participant should be initiated in conjunction with the sponsor and consideration given to the following:

- Obtain a more detailed history of symptoms and prior or concurrent diseases.
- Obtain a history of concomitant drug use, including nonprescription medications, herbal products and dietary supplements, alcohol and recreational drug use, and special diets.
- Obtain a history of exposure to environmental chemical agents.
- Initiation of appropriate evaluations including applicable laboratory tests (e.g., direct bilirubin, INR), physical assessments, and other assessments (e.g., imaging)

    - Rule out other potential causes of biochemical abnormalities, e.g., acute viral hepatitis types A, B, C, D, and E; autoimmune or alcoholic hepatitis; NASH; hypoxic/ischemic hepatopathy; and biliary tract disease.
- Consider gastroenterology or hepatology consultations.

If any of the following criteria are met, discontinuation of study intervention should be considered (if indicated, prior to receipt of confirming retest biochemistry laboratory test results) and the sponsor notified of the discontinuation:

- ALT or AST ≥3 × ULN and the participant is symptomatic with the appearance of fatigue, nausea, vomiting, right upper quadrant pain or tenderness, fever, rash, or eosinophilia (>5%)
- ALT or AST ≥3 × ULN and total bilirubin >2 × ULN or INR >1.5
- ALT or AST ≥5 × ULN for more than 2 weeks
- ALT or AST ≥8 × ULN

All participants showing liver biochemistry elevation meeting potential Hy's law criteria should be followed until all abnormalities return to normal or to the baseline state.

Reporting of Potential Hy's Law Cases

Potential Hy's law cases are defined by biochemical test results of hepatocellular injury and impaired hepatic function. They should be evaluated and followed further (i.e., close monitoring initiated) to determine whether these laboratory abnormalities are indicative of liver biochemistry elevation meeting potential Hy's law criteria. As indicated above, discontinuation of study intervention should also be considered. Criteria that identify a potential Hy's law case are as follows:

- ALT or AST ≥3 × ULN AND
- Total bilirubin ≥2 × ULN AND
- Alkaline phosphatase <2 × ULN

Sites must report every participant who meets the Hy's law criteria if this occurs within the time the participant signs the ICF until 30 days after the last dose of study intervention.

A laboratory alert for a liver biochemistry elevation potentially meeting Hy's law criteria case will be sent immediately to the sponsor and investigators when the above criteria have been met, even if no clinical symptoms have been experienced. An Adverse Event of Interest Abnormal Liver Function Reporting Form should be completed as soon as possible (within 24 hours of notification) for liver biochemistry elevations potentially meeting Hy's law criteria cases and submitted to the Sponsor. The eCRF pages associated with the potential Hy's law cases must be completed within 7 calendar days. Potential Hy's law cases will be evaluated by a hepatologist with expertise in drug-induced liver injury (DILI) and reviewed by the DSMB.

7.1.5. *Overdosage*

No specific information is available on the treatment of overdose of CVC. In the event of overdose, the participant should be observed closely for signs of toxicity. Appropriate supportive treatment should be provided if clinically indicated.

An overdose of CVC, regardless of the presence of an associated SAE, is considered an ECI and must be documented and reported.

Additionally, an SAE associated with an overdose of CVC must be documented and reported according to the requirements for SAEs.

7.1.6. *Preparation/Handling/Storage/Accountability*

7.1.6.1. *Acquisition and Accountability*

Investigational products (IP) will be shipped to the site either directly from participating companies, from the Sponsor, or from other regional or local drug repositories. All other supplies should be provided by the site. Multiple lots of each IP may be supplied.

Study products received at the sites will be open label and not kit specific, unless specified in the protocol-specific Manual of Procedures (MOP) or pharmacy manual. Drug preparation will be performed by the participating site's unblinded research pharmacist on the same day of administration to the participant. See the MOP Appendices for detailed information on the preparation, labeling, storage, and administration of investigational products.

Accountability:

The site PI is responsible for study product distribution and disposition and has ultimate responsibility for study product accountability. The site PI may delegate to the participating site's research pharmacist responsibility for study product accountability. The participating site's research pharmacist will be responsible for maintaining complete records and documentation of study product receipt, accountability, dispensation, storage conditions, and final disposition of the study product(s). Time of study drug administration to the participant will be recorded on the appropriate data collection form (CRF). All study product(s), whether administered or not, must be documented on the appropriate study product accountability record or dispensing log. The Sponsor's monitoring staff will verify the participating site's study product accountability records and dispensing logs per the site monitoring plan. Refer to the protocol-specific MOP for details on storing study medications.

Destruction:

After the study treatment period has ended or as appropriate over the course of the study after study product accountability has been performed, used active and placebo product can be destroyed on-site following applicable site procedures with a second staff member observing and verifying the destruction.

Unused product at the end of the study should be saved until instructed by the Sponsor.

7.1.6.2. *Formulation, Appearance, Packaging, and Labeling*

CVC Component:

The Cenicriviroc mesylate drug product formulation used for clinical trials is DP-7A, it is supplied as an immediate-release tablet for oral administration containing 150 mg of Cenicriviroc as Cenicriviroc mesylate (CVC) drug substance. The 150 mg tablets are yellow-coated. The formulated tablet contains the following excipients: fumaric acid (solubility enhancer), microcrystalline cellulose (filler), croscarmellose sodium (disintegrant), colloidal silicon dioxide (glidant), magnesium stearate (lubricant), and Opadry® II yellow (film coating).

Study drug will be provided in a HDPE bottle containing desiccant, and induction sealed.

Oral placebo component:

Placebo tablets match the active product in appearance. Placebo tablets contain excipients of microcrystalline cellulose, colloidal silicon dioxide, croscarmellose sodium, magnesium stearate, Opadry® II yellow, lactose monohydrate, and FD&C Yellow #5 Aluminum Lake.

7.1.6.3. *Product Storage and Stability*

CVC study drug is to be stored at 15°C to 30°C (59°F to 86°F) with transient excursions permitted to -20°C (-4°F) to 60°C (140°F).

7.1.6.4. *Preparation*

Refer to the protocol-specific MOP for details about preparation.

No agent preparation is required for CVC oral dosing. Preparation instructions for use of CVC with a feeding tube can be found in the Pharmacy Manual.

7.2. Measures to Minimize Bias: Randomization and Blinding

Randomization will as specified in the Master Protocol.

7.3. Study Intervention Compliance

Each dose of study product will be administered by a member of the clinical research team who is qualified and licensed to administer the study product. Administration and date, and time, will be entered into the case report form (CRF).

7.4. Concomitant Therapy

7.4.1. *Permitted Concomitant Therapy and Procedures*

All concomitant therapies permitted in the master protocol will be allowed for this sub-study, except lopinavir/ritonavir, as indicated below.

7.4.2. *Prohibited Concomitant Therapy*

All concomitant therapies prohibited in the master protocol will also be prohibited for this sub-study. Additional prohibited therapies are detailed below.

CVC Component:
Caution should always be exercised when administering concomitant medications based on the individual medication profile and clinical risk-benefit assessment. A complete list of prohibited medications is provided in **Appendix 3 Table 3**. Concomitant therapies of particular note include:

- Antiretroviral agents (See the table below [**Appendix 3 Table 3**] for other antiretroviral agents)
  - CVC can be used in combination with remdesivir.
  - Dolutegravir, Tenofovir each has no significant impact on CVC exposure and would be acceptable to co-administer with study intervention in this study.
  - Ritonavir or any combination of a protease inhibitor with ritonavir should not be co-administered with drug product in this study due to a significant drug interaction between ritonavir and CVC.

Appendix 3 Table 3: For CVC, Disallowed Medications

**Disallowed Medications**	
**Antibacterials**	rifampin, nafcillin, clarithromycin, erythromycin, telithromycin
**Anticonvulsants**	carbamazepin, phenytoin
**Antidepressants**	Nefazodone
**Antifungals**	voriconazole, itraconazole, ketoconazole, posaconazole
**Antihistamines**	Astemizole
**Anti-inflammatory drugs**	Sulfasalazine
**Antimetabolite drugs**	Methotrexate
**Antipsychotics**	Pimozide
**Antivirals**	Efavirenz, etravirine, boceprevir, dasabuvir/ombitasvir/paritaprevir/ritonavir, indinavir, lopinavir/ritonavir, nelfinavir, ombitasvir/paritaprevir/ritonavir, ritonavir, saquinavir, telaprevir, glecaprevir/pibrentasvir
**Ade Ergot Alkaloids**	dihydroergotamine, ergonovine, ergotamine, methylergonovine

**Lipid-lowering agents**	Gemfibrozil
**Other**	Cisapride

Appendix 3 Table 4: Allowed Medications Requiring Dose Adjustments

**Allowed Medications Requiring Dose Adjustments**	
**Opioids:**<br>**Fentanyl, Alfentanil**	The preference for opioid use for analgesia is to employ a non-fentanyl-based strategy (e.g., dilaudid). **Note: fentanyl and alfentanil are CYP3A4 substrates, thus use of either with CVC may increase fentanyl and alfentanil exposure.**<br><br>If needed, fentanyl/alfentanil should be administered with close surveillance and a dose-titration strategy:<br>• If feasible, it is recommended that the initial dose be decreased by 50% and up titrated to the desired effect.<br>• If a customary starting-dose is needed in the Investigator's judgment, should be down-titrated after initiation
**Sedative/hypnotics: midazolam, triazolam**	The preference for sedation is to employ a non-midazolam-based strategy (e.g., propofol). **Note: the exposure of midazolam (a substrate of CYP3A4) can be increased 1.84-fold when co-administered with CVC.**<br><br>If needed, midazolam or triazolam should be administered with close surveillance and a dose-titration strategy:<br>• If feasible, it is recommended that the initial dose be decreased by 50% and up titrated to the desired effect.<br>• If a customary starting-dose is needed in the investigator's judgement, should be down-titrated after initiation
**Immunosuppressants: cyclosporin, tacrolimus**	In patients actively receiving cyclosporine or tacrolimus, CVC should be given as 150 mg QD instead of 150 mg BID.
**Gastric acid-reducing agents (H2 receptor antagonists, antacids, proton-pump inhibitors [PPIs])**	Gastric acid-reducing agents should be administered at least 2 hours after CVC dosing (4 hours after dosing is ideal for fast-acting antacids). When possible, an H2 receptor antagonist (except cimetidine) or antacid is preferred over a proton-pump inhibitor (PPI). It is recommended to start with the lowest dose of these agents and titrate appropriately.<br>• H2 receptor antagonists (e.g., famotidine or ranitidine) should not exceed a dose comparable to famotidine 40 mg daily.<br>• Antacids (e.g., aluminum hydroxide, calcium carbonate, magnesium carbonate, magnesium hydroxide, or bismuth subsalicylate) should be given at least 4 hours after administration of study drug.<br>• PPIs (e.g., omeprazole, lansoprazole, esomeprazole, pantoprazole, rabeprazole, dexlansoprazole) are not recommended, however if needed, administer approximately 3 hours after study drug at a dose that does not exceed doses comparable to omeprazole 20 mg daily. Note: due to the prolonged acid-reducing effect of PPIs (~16-24 hours), it is advised to follow these recommendations to reduce their potential impact on absorption of the subsequent CVC dose.

Allowed Medications Requiring Dose Adjustments	
**Lipid-lowering agents: atorvastatin, simvastatin, lovastatin, pravastatin, rosuvastatin**	The maximum recommended daily doses are as follows:<br>• atorvastatin 40 mg,<br>• simvastatin 20 mg,<br>• lovastatin 40 mg,<br>• pravastatin 40 mg, and<br>• rosuvastatin 20 mg;<br>• pitavastatin use is allowed without dose restriction<br><br>The medical monitor or equivalent representative must be consulted prior to use of higher doses of statins than those recommended above.
**PDE5 enzyme inhibitors: sildenafil, tadalafil, vardenafil**	The recommended starting doses for these medications are as follows:<br>• sildenafil 25 mg,<br>• tadalafil 2.5 mg,<br>• vardenafil 2.5 mg
**Anticoagulants**	The recommended instructions for these medications are as follows:<br>• rivaroxaban - if required, do not exceed 10 mg<br>• apixaban and edoxaban – may increase CVC exposure, use with reduced doses and close clinical monitoring

7.4.3. *Rescue Medicine*

Not Applicable.

7.4.4. *Non-Research Standard of Care*

Not Applicable.

8. STUDY INTERVENTION DISCONTINUATION AND SUBJECT DISCONTINUATION/WITHDRAWAL

8.1. Halting Criteria and Discontinuation of Study Intervention

8.1.1. *Individual Study Product Halting*

Individual study product halting will be managed as specified in the Master Protocol.

Cenicriviroc Halting

See Section 7.1.4.1 for information about dosing modifications and potential discontinuation of CVC due to laboratory abnormalities.

8.1.2. *Study Halting*

Study halting will be managed as specified in the Master Protocol.

8.2. Withdrawal from the Study

Sub-study will have the same requirements as the main study.

8.3. Lost to Follow-Up

Sub-study will have the same requirements as the main study.

8.4. Readmission

Sub-study will have the same requirements as the main study.

9. STUDY ASSESSMENTS AND PROCEDURES

Sub-study will have the same assessments and procedures as the main protocol.

10. STATISTICAL CONSIDERATIONS

Statistical analysis will be performed as described within the main body of the ACTIV-1 IM master protocol.

APPENDIX 4 – ACTIV-1 IM PROTOCOL TEAM ROSTER

Role	Contact
**Study Chair**	**William G. Powderly, MD**<br>J. William Campbell Professor of Medicine<br>Larry J. Shapiro Director, Institute for Public Health<br>Director, Institute of Clinical and Translational Sciences<br>Co-Director, Division of Infectious Diseases<br>Washington University School of Medicine in St. Louis<br>[redacted]
**NCATS Representatives**	**Jane C. Atkinson, DDS**<br>Director, Trial Innovation Network<br>[redacted]<br><br>**Cynthia Boucher, MS**<br>Health Science Policy Analyst<br>[redacted]<br><br>**Soju Chang, MD, MPH**<br>Medical Officer<br>[redacted]<br><br>**Sam Bozzette, MD, PhD**<br>Chief Medical Officer<br>[redacted]
**OWS Representative**	**Thomas Stock, DO**<br>Clinical Advisor, OWS Therapeutics<br>Advanced Decision Vectors, Contractor Supporting OS/ASPR<br>[redacted]
**BARDA Representative**	**Anna O'Rourke, MS**<br>Senior Advisor, Division of Clinical Development<br>Biomedical Advanced Research and Development Authority<br>[redacted]
**ACTIV Clinical TX WG/NIAID Representative**	**Sarah Read, MD, MHS**<br>ACTIV Clinical Therapeutics Working Group Co-Chair<br>Deputy Director, Division of AIDS, NIAID<br>[redacted]
**Study Statisticians**	Blinded:<br>**Lisa LaVange, PhD**<br>Professor and Interim Co-Chair, Department of Biostatistics<br>University of North Carolina Gillings School of Health<br>[redacted]<br><br>Unblinded:<br>**Michael Proschan, PhD**<br>Mathematical Statistician, Biometrics Research Branch, NIAID<br>[redacted]

ACTIV-1 IM: Randomized Master Protocol for Immune Modulators for Treating COVID-19

Role	Contact
**DSMB Statisticians**	Blinded:<br>**Kevin Anstrom, PhD**<br>Director of Biostatistics/Professor of Biostatistics and Bioinformatics<br>Duke Clinical Research Institute<br>[redacted]<br><br>**Sean O'Brien, PhD**<br>Associate Professor of Biostatistics and Bioinformatics<br>Duke Clinical Research Institute<br>[redacted]<br><br>Unblinded:<br>**Hussein Al-Khalidi, PhD**<br>Professor of Biostatistics and Bioinformatics<br>Duke Clinical Research Institute<br>[redacted]<br><br>**Susan Halabi, PhD**<br>Professor of Biostatistics and Bioinformatics<br>Duke Clinical Research Institute<br>[redacted]
**Site Investigator Representatives**	**Emily R. Ko, MD, PhD**<br>Assistant Professor, Department of Medicine<br>Duke University<br>[redacted]<br>**Anne Lachiewicz, MD, MPH, FIDSA**<br>Associate Professor of Medicine<br>Division of Infectious Diseases<br>University of North Carolina<br>[redacted]<br>**Jane O'Halloran, MD, PhD**<br>Assistant Professor of Medicine<br>Washington University, St. Louis<br>[redacted]
**Site Coordinator Representative**	**Michael Klebert, PhD**<br>Washington University<br>[redacted]
**Community Representative – Chair of Community Advisory Board**	TBD

ACTIV-1 IM: Randomized Master Protocol for Immune Modulators for Treating COVID-19

**Role**	**Contact**
**Coordinating Center Representative(s)**	**Theresa Jasion, MSIS**<br>Project Lead, Trial Innovation Center<br>Duke Clinical Research Institute<br>[redacted]<br><br>**Neta Nelson, MPH**<br>Associate Director, Clinical Operations<br>Technical Resources International, Inc.<br>[redacted]<br><br>**Sandra Mendez Giraldo, MD**<br>Project Manager I, General Medicine<br>Syneos Health<br>[redacted]
**U.S. IND Sponsor**	**Daniel K. Benjamin, MD, MPH, PhD**<br>Kiser-Arena Distinguished Professor<br>Duke Clinical Research Institute<br>[redacted]
**Ex-U.S. HA Sponsor**	**Daniel Molina, PharmD, MBA**<br>VP, Strategic Planning<br>Technical Resources International, Inc.<br>[redacted]
**Industry Representatives**	Allergan/AbbVie:<br>**David B. Bharucha**, MD, PhD, FACC<br>Vice President, Clinical Development, R&D<br>[redacted]<br><br>Bristol Myers Squibb:<br>**Brian J. Gavin, PhD**<br>Development Lead, Orencia & Nulojix<br>Immunology & Fibrosis Development<br>[redacted]<br><br>Gilead Sciences:<br>**Huyen Cao, MD**<br>Sr. Director<br>[redacted]<br><br>Janssen Pharmaceuticals/Johnson & Johnson:<br>**Maria Beumont-Mauviel, MD**<br>Senior Director, Hepatitis Franchise Medical Lead<br>ID&V Medical Department<br>[redacted]
**FNIH Representative**	**Stacey J. Adam, PhD**<br>Director, Cancer<br>Foundation for the National Institutes of Health<br>[redacted]

Role	Contact
**Study Pharmacy Representative**	TBD

11. REFERENCES

Abatacept US Prescribing Information.

Alten, R., C.O. Bingham, 3rd, S.B. Cohen, J.R. Curtis, S. Kelly, D. Wong and M.C. Genovese (2016). "Antibody response to pneumococcal and influenza vaccination in patients with rheumatoid arthritis receiving abatacept." BMC Musculoskelet Disord **17**: 231.

Beigel, J., K.M. Tomashek, L.E. Dodd, A.K. Mehta, B.S. Zingman, A.C. Kalil, E. Hohmann, H.Y. Chu, A. Luetkemeyer, S.Kline, D. Lopez de Castilla, R.W. Finberg and ACTT-1 Study Group Members (2020). "Remdesivir for the Treatment of Covid-19 – Preliminary Report." N Engl J Med NEJMoa2007764.

Braun, J. and J. Sieper (2003). "Role of novel biological therapies in psoriatic arthritis: effects on joints and skin." BioDrugs **17**(3): 187-199.

Brenner, E.J., R.C. Ungaro, R.B. Gearry, G.G. Kaplan, M. Kissous-Hunt, J.D. Lewis, S.C. Ng, J.F. Rahier, W. Reinisch, F.M. Ruemmele, F. Steinwurz, F.E. Underwood, X. Zhang, J.F. Colombel and M.D. Kappelman (2020). "Corticosteroids, but not TNF Antagonists, are Associated with Adverse COVID-19 Outcomes in Patients With Inflammatory Bowel Diseases: Results from an International Registry." Gastroenterology **159**(2): 481-491.e3.

Buch, M.H., D.L. Boyle, S. Rosengren, B. Saleem, R.J. Reece, L.A. Rhodes, A. Radjenovic, A. English, H. Tang, G. Vratsanos, P. O'Connor, G.S. Firestein and P. Emery (2009). "Mode of action of abatacept in rheumatoid arthritis patients having failed tumour necrosis factor blockade: a histological, gene expression and dynamic magnetic resonance imaging pilot study." Ann Rheum Dis **68**(7): 1220-1227.

Carvajal Alegria, G., P. Pochard, J.O. Pers and D. Cornec (2016). "Could abatacept directly target expanded plasmablasts in IgG4-related disease?" Ann Rheum Dis **75**(11): e73.

Chambers, C.A. and J.P. Allison (1999). "CTLA-4--the costimulatory molecule that doesn't: regulation of T-cell responses by inhibition." Cold Spring Harb Symp Quant Biol **64**: 303-312.

Chen, J., Y.F. Lau, E.W. Lamirande, C.D. Paddock, J.H. Bartlett, S.R. Zaki and K. Subbarao (2010). "Cellular immune responses to severe acute respiratory syndrome coronavirus (SARS-CoV) infection in senescent BALB/c mice: CD4+ T cells are important in control of SARS-CoV infection." J Virol **84**(3): 1289-1301.

Chen, S.K., K.P. Liao, J. Liu and S.C. Kim (2020). "Risk of Hospitalized Infection and Initiation of Abatacept Versus Tumor Necrosis Factor Inhibitors Among Patients With Rheumatoid Arthritis: A Propensity Score-Matched Cohort Study." Arthritis Care Res (Hoboken) **72**(1): 9-17.

Chen, X., B. Zhao, Y. Qu, Y. Chen, J. Xiong, Y. Feng, D. Men, Q. Huang, Y. Liu, B. Yang, J. Ding and F. Li (2020). "Detectable serum SARS-CoV-2 viral load (RNAaemia) is closely correlated with drastically elevated interleukin 6 (IL-6) level in critically ill COVID-19 patients." Clin Infect Dis ciaa449.

Chung, E. S., M. Packer, K.H. Lo, A.A. Fasanmade and J.T. Willerson (2003). "Randomized, double-blind, placebo-controlled, pilot trial of infliximab, a chimeric monoclonal antibody to tumor necrosis factor-alpha, in patients with moderate-to-severe heart failure: results of the anti-TNF Therapy Against Congestive Heart Failure (ATTACH) trial." Circulation **107**(25): 3133-3140.

Company, B.M.S. (2020a). Abatacept (BMS-188667) Investigator Brochure Version 23.

Company, B.M.S. (2020b). Abatacept (BMS-188667) Investigator Brochure Addendum No. 1.

Conti, P., G. Ronconi, A. Caraffa, C.E. Gallenga, R. Ross, I. Frydas and S.K. Kritas (2020). "Induction of pro-inflammatory cytokines (IL-1 and IL-6) and lung inflammation by Coronavirus-19 (COVI-19 or SARS-CoV-2): anti-inflammatory strategies." J Biol Regul Homeost Agents **34**(2): 327-331.

Curtis, J.R., S. Yang, N.M. Patkar, L. Chen, J.A. Singh, G.W. Cannon, T.R. Mikuls, E. Delzell, K.G. Saag, M.M. Safford, S. DuVall, K. Alexander, P. Napalkov, K.L. Winthrop, M.J. Burton, A. Kamauu and J.W. Baddley (2014). "Risk of hospitalized bacterial infections associated with biologic treatment among US veterans with rheumatoid arthritis." Arthritis Care Res (Hoboken) **66**(7): 990-997.

Cutolo, M., S. Soldano, P. Montagna, A. Sulli, B. Seriolo, B. Villaggio, P. Triolo, P. Clerico, L. Felli and R. Brizzolara (2009). "CTLA4-Ig interacts with cultured synovial macrophages from rheumatoid arthritis patients and downregulates cytokine production." Arthritis Res Ther **11**(6): R176.

D'Antoni, M. L., B.I. Mitchell, S. McCurdy, M.M. Byron, D. Ogata-Arakaki, D. Chow, N.N. Mehta, W.A. Boisvert, E. Lefebvre, C.M. Shikuma, L.C. Ndhlovu and Y. Baumer (2018). "Cenicriviroc inhibits trans-endothelial passage of monocytes and is associated with impaired E-selectin expression." J Leukoc Biol **104**(6): 1241-1252.

Davis, P. M., S.G. Nadler, D.K. Stetsko and S.J. Suchard (2008). "Abatacept modulates human dendritic cell-stimulated T-cell proliferation and effector function independent of IDO induction." Clin Immunol **126**(1): 38-47.

Delmonte, O.M., R. Castagnoli, E. Calzoni and L.D. Notarangelo (2019). "Inborn Errors of Immunity With Immune Dysregulation: From Bench to Bedside." Front Pediatr **7**: 353.

Feldmann, M., R.N. Maini, J.N. Woody, S.T. Holgate, G. Winter, M. Rowland, D. Richards and T. Hussell (2020). "Trials of anti-tumour necrosis factor therapy for COVID-19 are urgently needed." Lancet **395**(10234): 1407-1409.

Fine, J.P., and R.J. Gray (1999), “A Proportional Hazards Model for the Subdistribution of a Competing Risk.” J Am Stat Assoc **94**(446): 496-509.

Haga, S., N. Yamamoto, C. Nakai-Murakami, Y. Osawa, K. Tokunaga, T. Sata, N. Yamamoto, T. Sasazuki and Y. Ishizaka (2008). "Modulation of TNF-alpha-converting enzyme by the spike protein of SARS-CoV and ACE2 induces TNF-alpha production and facilitates viral entry." Proc Natl Acad Sci U S A **105**(22): 7809-7814.

Huang, C., Y. Wang, X. Li, L. Ren, J. Zhao, Y. Hu, L. Zhang, G. Fan, J. Xu, X. Gu, Z. Cheng, T. Yu, J. Xia, Y. Wei, W. Wu, X. Xie, W. Yin, H. Li, M. Liu, Y. Xiao, H. Gao, L. Guo, J. Xie, G. Wang, R. Jiang, Z. Gao, Q. Jin, J. Wang and B. Cao (2020). "Clinical features of patients infected with 2019 novel coronavirus in Wuhan, China." Lancet **395**(10223): 497-506.

Hwang, I.K., W.J. Shih and J.S. De Cani (1990). "Group sequential designs using a family of type I error probability spending functions." Stat Med **9**(12): 1439-1445.

Jaiswal, S.R. and S. Chakrabarti (2020). "CTLA4Ig Limits Both Incidence and Severity of Early Cytokine Release Syndrome following Haploidentical Peripheral Blood Stem Cell Transplantation." Biol Blood Marrow Transplant **26**(4): e86-e87.

Kang, E.H., Y. Jin, R.J. Desai, J. Liu, J.A. Sparks and S.C. Kim (2019). "Risk of exacerbation of pulmonary comorbidities in patients with rheumatoid arthritis after initiation of abatacept versus TNF inhibitors: A cohort study." Semin Arthritis Rheum **50**(3): 401-408.

Komatsu, M., D. Kobayashi, K. Saito, D. Furuya, A. Yagihashi, H. Araake, N. Tsuji, S. Sakamaki, Y. Niitsu and N. Watanabe (2001). "Tumor necrosis factor-alpha in serum of patients with inflammatory bowel disease as measured by a highly sensitive immuno-PCR." Clin Chem **47**(7): 1297-1301.

Koura, D. T., J.T. Horan, A.A. Langston, M. Qayed, A. Mehta, H.J. Khoury, R.D. Harvey, Y. Suessmuth, C. Couture, J. Carr, A. Grizzle, H.R. Johnson, J.A. Cheeseman, J.A. Conger, J. Robertson, L. Stempora, B.E. Johnson, A. Garrett, A.D. Kirk, C.P. Larsen, E.K. Waller and L.S. Kean (2013). "In vivo T cell costimulation blockade with abatacept for acute graft-versus-host disease prevention: a first-in-disease trial." Biol Blood Marrow Transplant **19**(11): 1638-1649.

Law, H.K., C.Y. Cheung, S.F. Sia, Y.O. Chan, J.S. Peiris and Y.L. Lau (2009). "Toll-like receptors, chemokine receptors and death receptor ligands responses in SARS coronavirus infected human monocyte derived dendritic cells." BMC Immunol **10**: 35.

Linsley, P.S. and S.G. Nadler (2009). "The clinical utility of inhibiting CD28-mediated costimulation." Immunol Rev **229**(1): 307-321.

Lo, B., K. Zhang, W. Lu, L. Zheng, Q. Zhang, C. Kanellopoulou, Y. Zhang, Z. Liu, J. M. Fritz, R. Marsh, A. Husami, D. Kissell, S. Nortman, V. Chaturvedi, H. Haines, L. R. Young, J. Mo, A.H. Filipovich, J.J. Bleesing, P. Mustillo, M. Stephens, C.M. Rueda, C.A. Chougnet, K. Hoebe, J. McElwee, J. D. Hughes, E. Karakoc-Aydiner, H.

F. Matthews, S. Price, H.C. Su, V.K. Rao, M.J. Lenardo and M.B. Jordan (2015). "AUTOIMMUNE DISEASE. Patients with LRBA deficiency show CTLA4 loss and immune dysregulation responsive to abatacept therapy." Science **349**(6246): 436-440.

Marx, D., B. Moulin, S. Fafi-Kremer, I. Benotmane, G. Gautier, P. Perrin and S. Caillard (2020). "First case of COVID-19 in a kidney transplant recipient treated with belatacept." Am J Transplant **20**(7): 1944-1946.

Monti, S., S. Balduzzi, P. Delvino, E. Bellis, V.S. Quadrelli and C. Montecucco (2020). "Clinical course of COVID-19 in a series of patients with chronic arthritis treated with immunosuppressive targeted therapies." Ann Rheum Dis **79**(5): 667-668.

Monti, S., C. Klersy, R. Gorla, P. Sarzi-Puttini, F. Atzeni, R. Pellerito, E. Fusaro, G. Paolazzi, P.A. Rocchetta, E.G. Favalli, A. Marchesoni and R. Caporali (2017). "Factors influencing the choice of first- and second-line biologic therapy for the treatment of rheumatoid arthritis: real-life data from the Italian LORHEN Registry." Clin Rheumatol **36**(4): 753-761.

Mossanen, J.C., O. Krenkel, C. Ergen, O. Govaere, A. Liepelt, T. Puengel, F. Heymann, S. Kalthoff, E. Lefebvre, D. Eulberg, T. Luedde, G. Marx, C.P. Strassburg, T. Roskams, C. Trautwein and F. Tacke (2016). "Chemokine (C-C motif) receptor 2-positive monocytes aggravate the early phase of acetaminophen-induced acute liver injury." Hepatology **64**(5): 1667-1682.

Mulangu, S., L.E. Dodd, R.T. Davey, Jr., O. Tshiani Mbaya, M. Proschan, D. Mukadi, M. Lusakibanza Manzo, D. Nzolo, A. Tshomba Oloma, A. Ibanda, R. Ali, S. Coulibaly, A.C. Levine, R. Grais, J. Diaz, H.C. Lane, J.J. Muyembe-Tamfum, P.W. Group, B. Sivahera, M. Camara, R. Kojan, R. Walker, B. Dighero-Kemp, H. Cao, P. Mukumbayi, P. Mbala-Kingebeni, S. Ahuka, S. Albert, T. Bonnett, I. Crozier, M. Duvenhage, C. Proffitt, M. Teitelbaum, T. Moench, J. Aboulhab, K. Barrett, K. Cahill, K. Cone, R. Eckes, L. Hensley, B. Herpin, E. Higgs, J. Ledgerwood, J. Pierson, M. Smolskis, Y. Sow, J. Tierney, S. Sivapalasingam, W. Holman, N. Gettinger, D. Vallee, J. Nordwall and P.C.S. Team (2019). "A Randomized, Controlled Trial of Ebola Virus Disease Therapeutics." N Engl J Med **381**(24): 2293-2303.

Ng, M.W., G. Zhou, W. P. Chong, L.W. Lee, H.K. Law, H. Zhang, W.H. Wong, S.F. Fok, Y. Zhai, R.W. Yung, E. Y. Chow, K.L. Au, E.Y. Chan, W. Lim, J.S. Peiris, F. He and Y.L. Lau (2007). "The association of RANTES polymorphism with severe acute respiratory syndrome in Hong Kong and Beijing Chinese." BMC Infect Dis **7**: 50.

Prompetchara, E., C. Ketloy and T. Palaga (2020). "Immune responses in COVID-19 and potential vaccines: Lessons learned from SARS and MERS epidemic." Asian Pac J Allergy Immunol **38**(1): 1-9.

Puengel, T., O. Krenkel, M. Kohlhepp, E. Lefebvre, T. Luedde, C. Trautwein and F. Tacke (2017). "Differential impact of the dual CCR2/CCR5 inhibitor cenicriviroc on migration of monocyte and lymphocyte subsets in acute liver injury." PLoS One **12**(9): e0184694.

Record, J.L., T. Beukelman and R.Q. Cron (2011). "Combination therapy of abatacept and anakinra in children with refractory systemic juvenile idiopathic arthritis: a retrospective case series." J Rheumatol **38**(1): 180-181.

RECOVERY Collaborative Group (2020). "Dexamethasone in Hospitalized Patients with Covid-19 – Preliminary Report." N Engl J Med NEJMoa2021436.

Remdesivir FDA Product Label.

Ruan, Q., K. Yang, W. Wang, L. Jiang and J. Song (2020). "Clinical predictors of mortality due to COVID-19 based on an analysis of data of 150 patients from Wuhan, China." Intensive Care Med **46**(5): 846-848.

Salem, J.E., Y. Allenbach, A. Vozy, N. Brechot, D.B. Johnson, J.J. Moslehi and M. Kerneis (2019). "Abatacept for Severe Immune Checkpoint Inhibitor-Associated Myocarditis." N Engl J Med **380**(24): 2377-2379.

Teijaro, J.R., M. N. Njau, D. Verhoeven, S. Chandran, S.G. Nadler, J. Hasday and D.L. Farber (2009). "Costimulation modulation uncouples protection from immunopathology in memory T cell responses to influenza virus." J Immunol **182**(11): 6834-6843.

Tesch, V.K., H. Abolhassani, B. Shadur, J. Zobel, Y. Mareika, S. Sharapova, E. Karakoc-Aydiner, J.G. Rivière, M. Garcia-Prat, N. Moes, F. Haerynck, L.I. Gonzales-Granado, J.L. Santos Pérez, A. Mukhina, A. Shcherbina, A. Aghamohammadi, L. Hammarström, F. Dogu, S. Haskologlu, A.I. İkincioğulları, S. Köstel Bal, S. Baris, S.S. Kilic, N.E. Karaca, N. Kutukculer, H. Girschick, A. Kolios, S. Keles, V. Uygun, P. Stepensky, A. Worth, J.M. van Montfrans, A.M.J. Peters, I. Meyts, M. Adeli, A. Marzollo, N. Padem, A.M. Khojah, Z. Chavoshzadeh, M. Avbelj Stefanija, S. Bakhtiar, B. Florkin, M. Meeths, L. Gamez, B. Grimbacher, M.R.J. Seppänen, A. Lankester, A.R. Gennery and M.G. Seidel (2020). "Long-term outcome of LRBA deficiency in 76 patients after various treatment modalities as evaluated by the immune deficiency and dysregulation activity (IDDA) score." J Allergy Clin Immunol **145**(5): 1452-1463.

Tu, X., W.P. Chong, Y. Zhai, H. Zhang, F. Zhang, S. Wang, W. Liu, M. Wei, N. H. Siu, H. Yang, W. Yang, W. Cao, Y. L. Lau, F. He and G. Zhou (2015). "Functional polymorphisms of the CCL2 and MBL genes cumulatively increase susceptibility to severe acute respiratory syndrome coronavirus infection." J Infect **71**(1): 101-109.

Van Gool, S.W., Y. Zhang, A. Kasran, M. de Boer and J.L. Ceuppens (1996). "T helper-independent activation of human CD8+ cells: the role of CD28 costimulation." Scand J Immunol **44**(1): 21-29.

Watkins, B., M. Qayed, B. Bratrude, K. Betz, M. Brown, J. Rhodes, S. Sinclair, Y. Suessmuth, A. Yu, K. Hebert, M. C. Pasquini, S.W. Choi, J. Davis, C. Duncan, R. Giller, M. Grimley, A. C. Harris, D.A. Jacobsohn, N. Lalefar, M. Norkin, M.A. Pulsipher, S. Shenoy, B. Blazar, A. Langston, J.T. Horan and L. Kean (2017). "T Cell Costimulation Blockade with Abatacept Nearly Eliminates Early Severe Acute Graft Versus Host Disease after HLA-Mismatched (7/8 HLA Matched) Unrelated Donor Transplant, with a Favorable Impact on Disease-Free and Overall Survival." Blood **130**(Supplement 1): 212-212.

Watt, K., J.S. Li, D.K. Benjamin Jr. and M. Cohen-Wolkowiez (2011). "Pediatric cardiovascular drug dosing in critically ill children and extracorporeal membrane oxygenation." J Cardiovasc Pharmacol **58**(2): 126-132.

Wildschut, E.D., M.J. Ahsman, K. Allegaert, R.A. Mathot, and D. Tibboel (2010). "Determinants of drug absorption in different ECMO circuits." Intensive Care Med **36**(12): 2109-2116.

Weisman, M.H., P. Durez, D. Hallegua, R. Aranda, J. C. Becker, I. Nuamah, G. Vratsanos, Y. Zhou and L.W. Moreland (2006). "Reduction of inflammatory biomarker response by abatacept in treatment of rheumatoid arthritis." J Rheumatol **33**(11): 2162-2166.

Wenink, M.H., K. C. Santegoets, A.M. Platt, W.B. van den Berg, P.L. van Riel, P. Garside, T.R. Radstake and I.B. McInnes (2012). "Abatacept modulates proinflammatory macrophage responses upon cytokine-activated T cell and Toll-like receptor ligand stimulation." Ann Rheum Dis **71**(1): 80-83.

Whitfield, S.J. C., C. Taylor, J.E. Risdall, G.D. Griffiths, J.T.A. Jones, E.D. Williamson, S. Rijpkema, L. Saraiva, S. Vessillier, A.C. Green and A.J. Carter (2017). "Interference of the T Cell and Antigen-Presenting Cell Costimulatory Pathway Using CTLA4-Ig (Abatacept) Prevents Staphylococcal Enterotoxin B Pathology." J Immunol **198**(10): 3989-3998.

Whitmire, J.K. and R. Ahmed (2000). "Costimulation in antiviral immunity: differential requirements for CD4(+) and CD8(+) T cell responses." Curr Opin Immunol **12**(4): 448-455.

Williams, A.E., R.J. José, P.F. Mercer, D. Brealey, D. Parekh, D.R. Thickett, C. O'Kane, D.F. McAuley and R.C. Chambers (2017). "Evidence for chemokine synergy during neutrophil migration in ARDS." Thorax **72**(1): 66-73.

Wu, F., S. Zhao, B. Yu, Y.M. Chen, W. Wang, Z. G. Song, Y. Hu, Z.W. Tao, J.H. Tian, Y.Y. Pei, M.L. Yuan, Y.L. Zhang, F.H. Dai, Y. Liu, Q.M. Wang, J.J. Zheng, L. Xu, E.C. Holmes and Y.Z. Zhang (2020). "A new coronavirus associated with human respiratory disease in China." Nature **579**(7798): 265-269.

Zemans, R.L. and M.A. Matthay (2017). "What drives neutrophils to the alveoli in ARDS?" Thorax **72**(1): 1-3.

Zhou, F., T. Yu, R. Du, G. Fan, Y. Liu, Z. Liu, J. Xiang, Y. Wang, B. Song, X. Gu, L. Guan, Y. Wei, H. Li, X. Wu, J. Xu, S. Tu, Y. Zhang, H. Chen and B. Cao (2020). "Clinical course and risk factors for mortality of adult inpatients with COVID-19 in Wuhan, China: a retrospective cohort study." Lancet **395**(10229): 1054-1062.

Summary of Changes

ACTIV-1 IM: Randomized Master Protocol for Immune Modulators for Treating COVID-19

Version 1.0 (08 September 2020) to Version 2.0 (02 December 2020)

General:

- Protocol and ICF updated throughout with updated remdesivir risk and eligibility information based on the FDA-approved package insert. Note, however, that preparation and storage of remdesivir will still be according to the instructions in the Investigator's Brochure (see **Section 6.1.6.3, Product Storage and Stability**).
- Clarification throughout that standard-of-care (SoC) **may** include remdesivir, if in accordance with local or national guidelines.
- References to "serious infections" replaced with "secondary infections/superinfections" to be consistent with the CRF.

Inclusion Criteria:

- Participants are allowed to co-enroll in Protocol ACTIV-4 (4A and 4C).
- Participants in ACTIV-2 or a SARS-CoV-2 vaccine trial may immediately proceed to screening for ACTIV-1 IM if they develop COVID-19 requiring hospitalization. Participants in ACTIV-2 will complete ACTIV-2 follow-up while in ACTIV-1 IM.

Exclusion Criteria:

- Potential participants with ALT or AST >10x the institutional upper limit of normal are excluded (previously >5x).
- Potential participants with an eGFR <30 mL may be enrolled as long as their renal insufficiency has been stable for at least 1 month without renal replacement therapy (and are not current candidates for renal replacement therapy).
  - These participants will not receive remdesivir.
  - Also note that for this population, "new renal dysfunction" is defined in **Section 8.1.2** as "an increase in serum creatinine to >1.5 times the baseline value").
- Clarification that prohibited "cytotoxic or biologic" treatments are targeted immune-modulating therapies.
  - These include TNF inhibitors, anti-interleukin-1 (IL-1), anti-IL-6 (tocilizumab or sarilumab), anti-IL-17, T-cell or B-cell targeted therapies (e.g., rituximab), JAK inhibitors including baricitinib, and interferon.
  - These agents are prohibited within 4 weeks or 5 half-lives prior to screening, whichever is longer.
- There is no longer a dose restriction on dexamethasone, which is permitted in the context of treatment of COVID-19 patients who are already mechanically ventilated and in patients who require supplemental oxygen at screening, but who are not mechanically ventilated in accordance with national guidelines. See also **Section 6.4.1**.
- Clarified exclusion criteria #9 and #10 regarding suspicion of TB and other infections:
  - #9. Known or suspected history of untreated tuberculosis (TB). TB diagnosis may be suspected based on medical history and concomitant therapies that would suggest TB

infection. Participants are also excluded if they have known, latent TB treated for less than 4 weeks with appropriate anti-tuberculosis therapy per local guidelines (by history only, no screening required).

  - #10. Known or suspected serious, active bacterial, fungal, or viral infection (excepting SARS-CoV-2 and including, but not limited to, active HBV, HCV, or HIV with the latter defined as a CD4 count <200 <u>or</u> an unsuppressed HIV viral load), or other infection (besides COVID-19) that in the opinion of the investigator could constitute a risk when taking investigational product.
  - **Note:** Broad-spectrum empiric antibiotic usage prior to screening is **not** exclusionary.
- Clarification added to exclusion criterion #13 (known severe heart failure): Right-sided heart dysfunction or pulmonary hypertension <u>thought related to COVID-19</u> is permitted.

<u>Schedule of Assessments</u>:

- Added columns for Study Days 8 and 11; the standalone columns for Days 8, 11, 15, 22, and 29 depict the assessments to be performed if the participant has been discharged prior to the given study day (otherwise, if participant is still hospitalized, the "Daily until hospital discharge" assessments will be performed).
- Streamlined footnotes and moved details to other appropriate sub-sections of the protocol.
- Clarified that participants may receive a maximum of 10 days of remdesivir <u>in total</u>, including any doses of remdesivir administered prior to enrollment.
- Concomitant medications are now assessed daily through Day 29 (formerly Day 15):
  - While hospitalized, daily until Day 29 or discharge, whichever comes first.
  - After discharge, assessed at post-discharge follow-up visits through Day 29 (Days 8, 11, 15, 22, 29).
- Clarification added to PK assessments: "All efforts will be made to collect PK samples. If the samples are not collected due to complication or collected outside the window, this will not be considered a deviation."
- Participants on ECMO will now have a single additional PK blood sample; details to be provided in the study Manual of Operating Procedures (MOP).
- Blood samples for future secondary research (including genetic testing) are to be performed at CTSA sites, and are optional at all other United States (non-CTSA) sites. These samples will not be collected outside of the US.
- Added a new row for blood collection for genetic testing (baseline only, additional 8 mL collection; see also **Table 8-2** in **Section 8-2**).
- The following safety laboratory assessments have changed (see also **Section 8.2**):
  - Direct bilirubin, CPK-MB, and serum ferritin are no longer required to be assessed at any timepoint.
  - PT/INR, d-dimer, troponin, and C-reactive protein are assessed only at baseline and Day 8 (or at time of hospital discharge if prior to Day 8).
  - Routine standard-of-care bloodwork may be accepted for baseline blood collection if performed up to 48 hours prior to the baseline visit (previously 24 hours).

<u>Dosing/Dose Modifications</u>:

- Participants receiving remdesivir prior to enrollment will continue to receive remdesivir at the 100 mg maintenance dose level for up to 10 days total (including the days of administration prior to enrollment on ACTIV-1 IM).
- Discontinue remdesivir if:
    - ALT increases to >10x upper limit of normal (previously >5x the upper limit of normal for either ALT or AST)
    - ALT elevation (>5x upper limit of normal) is accompanied by signs or symptoms of liver inflammation.

Appendix 3 / Sub-study 3 (Cenicriviroc):

- Removed details of potential additional PK blood draws (for patients on enteral feeding or ECMO). Details will be provided in the MOP.

Study Staff Information:

- Added **Appendix 4: Study Team Roster** providing institutional affiliations and contact information (e-mail) for critical study staff.

Master Protocol for Immune Modulators for Treating COVID-19

Protocol: ACTIV-1 IM

STATISTICAL ANALYSIS PLAN (SAP)

Protocol Version 1.0

Protocol Version Date: 08 SEP 2020

SAP Version Number: 1.0

25 NOV 2020

Daniel K. Benjamin, MD, MPH, PhD
Kiser-Arena Distinguished Professor, Duke University Pediatrics
Duke Clinical Research Institute
300 W. Morgan Street, Suite 800
Durham Center
Durham, NC 27701
Tel: 919-668-7081
E-mail: danny.benjamin@duke.edu

TABLE OF CONTENTS

List of Abbreviations 4
1.0 Study and Document Overview 5
2.0 Study Objectives 6
3.0 Statistical Hypotheses and Endpoints 8
3.1 Study Definitions and Derived Variables 11
3.1.1 VISIT DAY 11
3.1.2 Measures of Clinical Support, Limitations, and Infection Control 11
3.1.3 Extrapulmonary Manifestations 11
3.1.4 National Early Warning Score (NEWS) 13
3.2 Power and Sample Size 14
3.3 Randomization Scheme 15
3.4 Interim Analyses and Data Monitoring 16
3.4.1 Interim Safety Analyses 16
3.4.2 Interim Analyses for Futility and Efficacy Review 16
3.4.3 Adaptations 17
3.5 Data Sources 18
3.6 Documentation Convention 18
3.7 Verification of Results 18
3.8 Subject Disposition 18
3.9 Populations for Analyses 19
4.0 Statistical Analyses 20
4.1 General Approach 20
4.2 Analysis of the Primary Efficacy Endpoint 22
4.3 Analysis of the Secondary efficacy Endpoint(s) 23
4.4 Safety Analyses 25
4.4.1 Laboratory Data 25
4.4.2 Vital Signs 26
4.4.3 Prior and Comcomitant Medications 26
4.4.4 Treatment Compliance 26
4.4.5 Pregnancy Tests 26
4.5 Exploratory Analyses 26
4.6 Baseline Descriptive Statistics 27
4.7 Sub-Group Analyses 27
4.8 References 28
4.9 Appendices 29

LIST OF ABBREVIATIONS

ACTIV	Accelerating COVID-19 Therapeutic Interventions and Vaccines
ADaM	Analysis Data Model
AE	Adverse Event
ALT	Alanine transaminase
AST	Aspartate transaminase
ATC	Anatomical Therapeutic Classification
BMI	Body Mass Index
CDISC	Clinical Data Interchange Standards Consortium
CI	Confidence Interval
COVID-19	Coronavirus
CRP	C-Reactive Protein
CTCAE	Common Terminology Criteria for Adverse Events
DAIDS	Division of AIDS
DCRI	Duke Clinical Research Institute
DSMB	Data and Safety Monitoring Board
ECMO	Extracorporeal Membrane Oxygenation
eCRF	Electronic Case Report Form
EOS	End of Study
ET	Early Termination
FU	Follow-up
ITT	Intent-to-Treat
LLN	Lower Limit of Normal
MedDRA	Medical Dictionary of Regulatory Activities
MITT	Modified Intent-to-Treat
NEWS	National Early Warning Score
PT	Preferred Term
Q1	25th Percentile (1st Quartile)
Q3	75th Percentile (3rd Quartile)
RR	Respiratory Rate
RRR	Recovery Rate Ratio
SAE	Serious Adverse Event
SAP	Statistical Analysis Plan
SARS-CoV-2	Severe Acute Respiratory Syndrome Coronavirus 2
SD	Standard Deviation
SDTM	Study Data Tabulation Model
SE	Standard Error
SoC	Standard of Care
ULN	Upper Limit of Normal
WBC	White Blood Cell

1.0 STUDY AND DOCUMENT OVERVIEW

ACTIV-1 IM is a master protocol designed to evaluate multiple investigational agents for the treatment of moderately or severely ill patients infected with severe acute respiratory syndrome coronavirus 2 (SARS-CoV-2). ACTIV-1 IM is a randomized, controlled, double-blinded trial which will evaluate each agent with respect to speed of recovery, mortality, illness severity, and hospital resource utilization. Each agent will be evaluated as add-on therapy to the standard of care (SoC) in use at the local clinics, including remdesivir (provided). The SoC may change during the course of the study based on other research findings. Comparisons of the investigational agents among themselves is not a research objective.

This document describes the planned statistical analyses that will be conducted at the completion of the ACTIV-1 IM study for each of the investigational agents. Once the initial unblinded data review has occurred, only the blinded statistics team members will be allowed to modify the SAP (see Appendix 1 for the list of blinded and unblinded statisticians). The initial unblinded analyses are expected to review data from the first 40 participants with at least 15 days of follow-up. That meeting is planned for January 2021.

The study population corresponds to moderately and severely ill adults (≥18 years old) with the coronavirus disease 2019 (COVID-19) virus. Recruitment will target patients already hospitalized for treatment of COVID-19 infection as well as patients being treated for COVID-19 infection in Emergency Departments while waiting to be admitted to the hospital. Patients both in and out of the intensive care unit (ICU) will be included in the study population.

Enrollment began in October 2020 with 3 agents selected for initial evaluation. Up to 5 agents may be evaluated in ACTIV-1 IM.

The study period is 29 days, with assessments on each day of the hospital stay. If the participants are discharged from the hospital, they will have a study visit at Days 8, 11, 15, 22, and 29. For discharged participants, it is preferred that the Day 8, 11, 15, and 29 visits are in person to obtain safety laboratory tests (Day 29) and blood (serum and plasma) samples for secondary research as well as clinical outcome data. However, infection control or other restrictions may limit the ability of the participant to return to the clinic. In this case, these visits may be conducted by phone, and only clinical data will be obtained. The Day 22 visit does not have laboratory tests or collection of samples and is conducted by phone. There will also be a safety and clinical status assessment at 60 days conducted by phone. Treatment periods may vary by agent.

The trial is adaptive in that interim analyses are planned to assess the futility of each agent, with the goal of discontinuing those with lower probabilities of success to more effectively utilize trial resources for the remaining agents. Additionally, interim analyses are planned for early stopping for efficacy. Alpha spending functions are used to appropriately control the probability of making an erroneous conclusion at the interim and final analyses.

Safety monitoring will be performed throughout the trial, and formal stopping rules for each agent will be adopted. The Data and Safety Monitoring Board (DSMB) established for ACTIV-1 IM will have oversight responsibility for the study.

The effectiveness of each therapeutic agent as add-on therapy to SoC plus remdesivir (provided) will be evaluated based on the primary endpoint of time to recovery by Day 29. The sample size requirements are based on the ability to detect a moderate improvement in time to recovery for each agent. A total of 788 recoveries are required for each comparison to provide approximately 85% power to detect a recovery rate ratio (RRR) of 1.25. Assuming 73% of participants achieve recovery in 28 days, consistent with the ACTT-1 (Beigel, Tomashek et al. 2020) results, the total sample size to evaluate 1, 2, and 3 agents in ACTIV-1 IM is approximately 1080, 1620, and 2160, respectively. Because each agent is being compared to SoC plus placebo with no between-agent comparisons, no multiplicity adjustments for multiple agents are planned.

2.0 STUDY OBJECTIVES

Primary Objective:

- To evaluate the clinical efficacy of different investigational therapeutics relative to the control arm in adults hospitalized with COVID-19 with respect to time to recovery by Day 29 from randomization.

Key Secondary Objectives:

- To evaluate the clinical efficacy of different investigational therapeutics relative to the control arm in adults hospitalized with COVID-19 according to:
  - Clinical status (8-point ordinal scale) assessed on Day 15 and Day 29
  - Mortality
    - 14-day mortality
    - 28-day mortality

Additional Secondary Objectives:

- To evaluate the clinical efficacy of different investigational therapeutics relative to the control arm as assessed by:
  - **Clinical Severity**
    - Ordinal scale:
      - Time to an improvement of one category and two categories from Day 1 (baseline) using an 8-point ordinal scale.
      - Subject clinical status using 8-point ordinal scale at Days 3, 5, 8, 11, 15, 22, and 29.
      - Mean change in the 8-point ordinal scale from Day 1 to Days 3, 5, 8, 11, 15, 22, and 29.
      - Total severity score (TSS): 8-point ordinal scale summarized as a daily score (for days collected) averaged over time from Day 1 through Day 29
    - Oxygenation:
      - Oxygenation use up to Day 29.
      - Incidence and duration of new oxygen use during the study.
    - Non-invasive ventilation/high flow oxygen:
      - Non-invasive ventilation/high flow oxygen use up to Day 29.

      - Incidence and duration of new non-invasive ventilation or high flow oxygen use during the study.
    - Invasive Mechanical Ventilation / extracorporeal membrane oxygenation (ECMO):
      - Ventilator / ECMO use up to Day 29.
      - Incidence and duration of new mechanical ventilation or ECMO use during the study.

  - **Hospitalization**
    - Duration of hospitalization (days) from randomization to discharge.

- To evaluate clinical status at Day 60.
- To evaluate the safety of different investigational therapeutics relative to the control arm as assessed by:
  - Cumulative incidence of SAEs through Day 29.
  - Cumulative incidence of Grade 3 and 4 clinical and/or laboratory AEs through Day 29.
  - Discontinuation or temporary suspension of study product administrations (for any reason).
  - Changes in white blood cell (WBC) count with differential, hemoglobin, platelets, creatinine, glucose, total bilirubin, alanine aminotransferase (ALT), aspartate aminotransferase (AST), alkaline phosphatase (ALP), prothrombin time (PT)/international normalized ratio (INR), d-dimer, cardiac troponin, and C-reactive protein (CRP) over time (analysis of lab values in addition to AEs noted above).
- To evaluate the impact of different investigational therapeutics relative to the control arm of extrapulmonary manifestations of COVID-19.

Exploratory Objectives:

- To assess the National Early Warning Score (NEWS):
    - Time to discharge or to a NEWS of ≤ 2 and maintained for 24 hours, whichever occurs first.
    - Change from Day 1 to Days 3, 5, 8, 11, 15, and 29 in NEWS.
- To collect blood samples for future research.

3.0 STATISTICAL HYPOTHESES AND ENDPOINTS

- Primary: The primary null hypothesis being tested is that time-to-recovery does not differ between each test agent (plus SoC) and the pooled control group, consisting of patients receiving SoC plus placebo. The alternative hypothesis is that the test agent and pooled control group differ in time-to-recovery in that the test agent group has a shorter time-to-recovery than the pooled control group. If Drug A, B, and C denote the initial set of agents to be tested in ACTIV-1 IM plus SoC, and S denotes patients receiving SoC plus placebo, then the primary hypotheses are:

  H_{01}: $TR_A = TR_S$ vs H_{A1}: $TR_A \neq TR_S$

  H_{02}: $TR_B = TR_S$ vs H_{A2}: $TR_B \neq TR_S$

  H_{03}: $TR_C = TR_S$ vs H_{A3}: $TR_C \neq TR_S$

  There are two key secondary endpoints that will be evaluated as supportive evidence:

  1. The distribution of the 8-point ordinal scale (see definition below) at Day 15 and Day 29. For this, the parameter of interest is the "common odds ratio," which quantifies the shift in the severity distribution resulting from treatment. The odds ratio will be defined such that a value greater than 1 implies better outcomes with active drug compared to control and a value less than 1 implies worse outcomes with active drug compared to control. In this case, for an efficacious treatment, an odds ratio greater than 1 quantifies an improvement in disease severity; a value of 2 indicates a bigger improvement than a value of 1.25. The null hypothesis to be tested is that the odds of improvement on the ordinal scale is the same for the placebo and experimental treatment arms (i.e., the common odds ratio is 1). It is worth noting that, for large sample sizes, the test based on the proportional odds model is nearly the same as the Wilcoxon rank sum test.
  2. Mortality will be evaluated with standard survival analysis techniques. Kaplan Meier curves and associated stratified log-rank statistics will be generated to compare mortality between each test agent and SoC. The stratification factors are based on the combination of geographic region and baseline disease severity Mortality is evaluated at Days 15 and 29 to assess treatment effects in 14-day and 28-day mortality.

- 8-Point Ordinal Scale
  - The 8-point ordinal scale is the basis for the primary and key secondary clinical endpoints in the study, namely, time to recovery, improvement in disease severity, and mortality.
  - The scale used in this study is as follows (from worst to best):
    - 1: Death;
    - 2: Hospitalized, on invasive mechanical ventilation or ECMO;

        - 3: Hospitalized, on non-invasive ventilation or high flow oxygen devices;
        - 4: Hospitalized, requiring supplemental oxygen;
        - 5: Hospitalized, not requiring supplemental oxygen – requiring ongoing medical in-patient care (COVID-19 related or otherwise);
        - 6: Hospitalized, not requiring supplemental oxygen – no longer requires ongoing medical in-patient care;
            - This would include those kept in hospital for quarantine/infection control/social reasons, awaiting bed in rehabilitation facility or homecare, etc.
        - 7: Not hospitalized, limitation on activities and/or requiring home oxygen;
        - 8: Not hospitalized, no limitations on activities

- Primary Efficacy Endpoint
    - Time to recovery by Day 29
    - Day of recovery is defined as the first day on which the participant satisfies one of the following three categories from the ordinal scale:
        - Hospitalized, not requiring supplemental oxygen – no longer requires ongoing medical in-patient care;
        - Not hospitalized, limitation on activities and/or requiring home oxygen;
        - Not hospitalized, no limitations on activities.

- Key Secondary Endpoints
    - Clinical status (8-point ordinal scale) assessed on Day 15 and Day 29 for the previous day
    - 14-day and 28-day mortality assessed using date and cause of death (if applicable)

- Additional Secondary Endpoints
    - Clinical severity assessed using ordinal scale daily while hospitalized and on Days 15, 22, and 29
    - Days alive and free of supplemental oxygen (if applicable) up to Day 29
    - Days alive and free of non-invasive ventilation/high oxygen (if applicable) up to Day 29
    - Days alive and free of invasive mechanical ventilation/ECMO (if applicable) up to Day 29
    - Days alive and out of hospital up to Day 29
    - Clinical status (8-point ordinal scale) at Day 60
    - Serious Adverse Events (SAEs)
    - Grade 3 and 4 Adverse Events (AEs)
    - Baseline laboratory tests include CBC with differential (including absolute neutrophil count and absolute lymphocyte count), ALT, AST, ALP, total bilirubin, creatinine (with calculated eGFR), glucose, Troponin, PT/INR, d-dimer, and C-reactive protein. *On Days 3, 5, 8, 11, 15, and 29, safety laboratory tests include WBC with differential, hemoglobin, platelets, ALT, AST, ALP, total bilirubin, creatinine (with calculated*

*eGFR), and glucose. On Day 8 or on day of discharge (if less than 24 hours prior to Day 8), the following **additional** laboratory assessments are performed: Troponin, PT/INR, d-dimer, and C-reactive protein.*

  - Incidence of individual and "any" specified extrapulmonary manifestations at Day 29

- Exploratory Endpoints
  - NEWS assessed daily while hospitalized (Day 1 – discharge) and on Days 15 and 29, if feasible
  - Blood draws on Days 1, 3, 8, 15, and 29

3.1 STUDY DEFINITIONS AND DERIVED VARIABLES

3.1.1 VISIT DAY

The baseline visit is the date of randomization (Day 1). Post-baseline study day visits are documenting the clinical information through the completion of the prior day (e.g. Day 29 is collecting data through of end of Day 28).

3.1.2 MEASURES OF CLINICAL SUPPORT, LIMITATIONS, AND INFECTION CONTROL

The participant's clinical status will be captured on each study day while hospitalized through Day 29. If a participant is discharged prior to Day 15, clinical status is collected on Day 8, Day 11, Day 15, and Day 29, if the participant returns for an in-person clinic visit or by phone if an in-person visit is not possible. Clinical status will also be captured on Day 22 during a phone visit. Clinical status is largely measured by the 8-point ordinal scale and the NEWS. Unlike the NEWS, the ordinal scale can also be evaluated over the phone if the discharged participant is unable to return for visits on Day 8, 11, 15, or 29 as well as on Day 22.

Improvement will be defined as an increase of at least one point on the 8-point scale compared to the baseline value (e.g. from 4 to 5) and the time to improvement will be defined as the elapsed time (in days) from Day 1 to the earliest day of observed improvement.

Any subjects that are lost to follow-up or terminated early prior to an observed improvement will be censored at the day of their last observed assessment. Subjects who complete follow-up but do not experience improvement will be censored at the day of their Day 29 visit.

3.1.3 EXTRAPULMONARY MANIFESTATIONS

The presence of the extrapulmonary manifestation of disease during the course of hospitalization will be assessed and reported at discharge. These tables will be reported with stratification by the participant's baseline clinical status. Specifically, clinical organ failure is defined by development of any one or more of the following clinical events:

a. Respiratory dysfunction:
   1. Respiratory failure defined as receipt of high flow nasal oxygen, noninvasive ventilation, invasive mechanical ventilation or ECMO

b. Cardiac and vascular dysfunction:
   1. Myocardial infarction
   2. Myocarditis or pericarditis
   3. Congestive heart failure: new onset NYHA class III or IV, or worsening to class III or IV
   4. Hypotension requiring institution of vasopressor therapy

c. Renal dysfunction:
   1. New requirement for renal replacement therapy

d. Hepatic dysfunction:
   1. Hepatic decompensation

e. Neurological dysfunction
1. Acute delirium
2. Cerebrovascular event (stroke, cerebrovascular accident [CVA])
3. Transient ischemic events (i.e., CVA symptomatology resolving <24 hrs)
4. Encephalitis, meningitis or myelitis

f. Hematological dysfunction:
1. Disseminated intravascular coagulation
2. New arterial or venous thromboembolic events, including pulmonary embolism and deep vein thrombosis
3. Major bleeding events (>2 units of blood within 24 hours, bleeding at a critical site (intracranial, intraspinal, intraocular, pericardial, intraarticular, intramuscular with compartment syndrome, or retroperitoneal), or fatal bleeding).

g. Secondary infection / superinfection:
1. Intercurrent, at least probable, documented serious disease caused by an infection other than SARS-CoV2, requiring antimicrobial administration and care.
2. A complete list of secondary infections / superinfections is provided in the study manual of procedures.

3.1.4 NATIONAL EARLY WARNING SCORE (NEWS)

Vital signs and other clinical assessments are collected for the calculation of the NEWS, and include temperature, systolic blood pressure, heart rate, respiratory rate, O_2 saturation and level of consciousness. Vital signs collected per standard of care can be used. NEWS has demonstrated an ability to discriminate subjects at risk of poor outcomes. (Smith, Prytherch et al. 2016). This score is based on 7 clinical parameters (see **Table 1**). The NEWS is being used as an exploratory efficacy measure. The components of NEWS should be evaluated daily while hospitalized. It can be performed concurrently with the Ordinal Scale. This should be evaluated at a consistent time for each study day and prior to administration of study product. The 7 parameters can be obtained from the hospital chart or electronic medical record (EMR) using the last measurement prior to the time of assessment (including parameters collected prior to the time of consent) and a numeric score is given for each parameter (e.g., a respiratory rate [RR] of 9 breaths per minute is one point, oxygen saturation of 92% is two points). This is recorded for the day obtained (i.e., on Day 3, the vital signs and other parameters from Day 3 will be used to obtain NEWS for Day 3). ECMO and mechanically ventilated participants should be assigned a score of 3 for RR (RR ≤8 breaths per minute) regardless of the ventilator setting. Participants on ECMO should get a score of 3 for heart rate since they are on cardiopulmonary bypass.

Table 1. National Early Warning Score (NEWS)

PHYSIOLOGICAL PARAMETERS	3	2	1	0	1	2	3
Respiration Rate (breaths per minute)	≤8 [a]		9 – 11	12 – 20		21 – 24	≥25
Oxygen Saturation (%)	≤91	92 – 93	94 – 95	≥96			
Any Supplemental Oxygen		Yes		No			
Temperature (°C)	≤35.0		35.1 – 36.0	36.1 – 38.0	38.1 – 39.0	≥39.1	
Systolic Blood Pressure (mm Hg)	≤90	91 – 100	101 – 110	111 – 219			≥220
Heart Rate (beats per minute)	≤40 [b]		41 – 50	51 – 90	91 – 110	111 – 130	≥131
Level of Consciousness				A			V, P, U

Level of consciousness = alert (A), and non-alert and arousable only to voice (V) or pain (P), and unresponsive (U).

[a] *If the participant is on ECMO or invasive mechanical ventilation, they will be given a score of 3 (≤8 breaths per minute) for respiratory rate regardless of ventilator setting.*

[b] *Participants on ECMO will also receive a score of 3 (≤40 beats per minute) for heart rate.*

3.2 POWER AND SAMPLE SIZE

ACTIV-1 is an event-driven trial based on the number of observed recovery events. The effectiveness of each therapeutic agent as add-on therapy to SoC will be evaluated based on the primary endpoint of time to recovery by Day 29. The Fine-Gray approach will be used to compare each test agent + SoC vs. SoC-alone with respect to the cumulative incidence of recovery, accounting for the competing risk of mortality (Fine and Gray 1999). The approach is similar to using a log-rank test on time to recovery, retaining in the risk set people who die. The two key determinants of power are the total number of events (i.e., recoveries) and the treatment-to-control recovery rate ratio (RRR).

Overall study sample size requirements are based on the number of agents being evaluated and the ability to pool control patients for analysis. Initial sample size estimates are derived assuming three agents are ready for testing at study start and will remain in the study for evaluation at the final analysis stage. If newly emergent therapies are entered into the master protocol after study start, sample size requirements will be adjusted accordingly.

Table 2 provides the numbers of recoveries and of patients required to provide 85% power for a single pairwise comparison of test drug versus control assuming a 73% recovery rate and various RRRs. The assumed 73% recovery rate is based on the Kaplan-Meier recovery rate from ACTT-1. Note that the recovery rate is the analogue of the hazard for each test agent or control treatment, and the RRR is the analogue of the hazard ratio for a test agent relative to control in this setting. Approximately 347 recoveries are required to detect a 40% increase in the rate of recovery ($\theta = 1.40$) from control. An RRR of 1.40 is similar to, but slightly higher than the figure of 1.31 reported in Cao et al. (2020) for a lopinavir/ritonavir trial that used time to improvement by 2 categories as primary endpoint (Cao, Wang et al. 2020). A total of 436 recoveries is needed for an RRR of 1.35 with 85% power.

Table 2. Number of recoveries and number of patients needed for 85% power assuming a type I error rate of 5% for various recovery ratios

Recovery rate ratio (θ)	Number of recoveries needed for 85% power	Sample Size assuming 73% recovery rate (1 test agent vs SoC)
1.25	**788**	**1,080**
1.30	570	781
1.35	436	598
1.40	347	476

The sample size requirements are based on the ability to detect a moderate improvement in time to recovery for each agent. After accounting for the proposed interim efficacy and futility monitoring rules, a total of 788 recoveries are required for each comparison to provide approximately 85% power to detect a RRR of 1.25. Assuming 73% of participants achieve recovery in 28 days, consistent with the ACTT-1 results results (Beigel, Tomashek et al. 2020), the total sample size to evaluate 1, 2, and 3 agents in ACTIV-1 IM is approximately 1080, 1620, and 2160, respectively.

The proposed power and sample size requirements also provide adequate power to detect differences in the key secondary endpoints of clinical improvement and mortality. Based on the formulas from

Whitehead (1993), a sample size of 1,080 (540 participants / group)) gives approximately 95% or 85% power to detect a common odds ratio of 1.50 of clinical improvement (the observed odds ratio in the ACTT-1 trial) or 1.40 using a 2-tailed test at level $\alpha=0.05$. The 28-day mortality probability in the remdesivir arm of ACTT-1 was approximately 12%. The power for comparing 28-day mortality is 90% for a 50% relative reduction (from 12% to 6%) and 80% to detect a difference of 12.0% vs. 6.8%.

3.3 RANDOMIZATION SCHEME

Randomization will be stratified by:

- Geographic Region
  - USA – Northeast
    - Connecticut, Maine, Massachusetts, New Hampshire, New Jersey, New York, Pennsylvania, Rhode Island, Vermont
  - USA - Midwest
    - Illinois, Indiana, Iowa, Kansas, Michigan, Minnesota, Missouri, North Dakota, Nebraska, Ohio, South Dakota, Wisconsin
  - USA – South
    - Alabama, Arkansas, Delaware, District of Columbia, Florida, Georgia, Kentucky, Louisiana, Maryland, Mississippi, North Carolina, Oklahoma, South Carolina, Tennessee, Texas, Virginia, West Virginia
  - USA – West
    - Alaska, Arizona, California, Colorado, Hawaii, Idaho, Montana, New Mexico, Nevada, Oregon, Utah, Wyoming, Washington
  - Argentina
  - Brazil
  - Peru
  - Colombia
  - Additional countries will be treated as separate regions
- Severity of illness at enrollment (by ordinal scale)
  - Severe disease:
    - Hospitalized, on invasive mechanical ventilation or ECMO, or
    - Hospitalized, on non-invasive ventilation or high flow oxygen devices.
  - Moderate disease:
    - Hospitalized, requiring supplemental oxygen, or
    - Hospitalized, not requiring supplemental oxygen.

Conceptually, randomization will proceed in two steps. At the first step, each participant will be assigned (open-label) with equal probability to one of the agents available at the time the patient is enrolled and for which the patient is eligible to receive, after applying any agent-specific safety exclusions. At the second step, each participant will be assigned to either the test agent or its matching placebo in an n:1 ratio, where n = the number of agents currently active in the master protocol and for which the patient is eligible to receive.

With SoC and three agents active simultaneously, if the participant meets each of the agent-specific criteria, this procedure results in the randomization ratio of 1:1:1:1. If 3 agents are available, but a patient is only eligible to receive 2 of them, the allocation ratio at the first step will be 1:1, and at the second step will be 2:1 (agent vs placebo). Inclusion of a matching placebo for each agent enables masking of study participants and clinical personnel to treatment assignment at the second stage. Data from patients receiving SoC plus placebo will be pooled across agents for comparative analyses and hypothesis testing. Comparative analyses of a particular agent will include the subset of pooled placebo patients who enrolled concurrently with the particular agent and would have been eligible to receive the agent. That is, patients enrolled to control arm prior to a new agent entering the trial will not be included in comparative analyses of that newly added investigation agent.

3.4 INTERIM ANALYSES AND DATA MONITORING

A complete description of the interim analyses plans including mock tables, listings, and figures is included in a separate DSMB guidance document. Briefly, unblinded interim analyses are planned to (i) assess the futility of each agent, with the goal of discontinuing those with lower probabilities of success to more effectively utilize trial resources for the remaining agents and (ii) review comparative analyses for each test agent to assess early stopping for efficacy. Alpha spending functions will be used to appropriately control the probability of making an erroneous conclusion across interim and final efficacy analyses at $\alpha = 0.05$ (two-sided) for each agent.

3.4.1 INTERIM SAFETY ANALYSES

Safety analyses will evaluate Grade 3 and 4 AE and SAEs by treatment arm. Safety monitoring will be ongoing. The unblinded statistical team will prepare these reports for review by the DSMB. More details will be provided in a separate DSMB guidance document.

3.4.2 INTERIM ANALYSES FOR FUTILITY AND EFFICACY REVIEW

Interim analyses for futility and efficacy are planned at three times during the study corresponding to the availability of approximately 25%, 50%, and 75% of total information. The planned randomization algorithm ensures approximately equal allocation to each test agent or control assuming that participants are eligible for all available agents. It is anticipated that the interim analyses for each agent will occur at the same time, and the DSMB will make recommendations for all agents at their scheduled meetings. If recovery rates vary substantially by agent; however, it may be necessary to let the interim analysis times also vary by agent. Because Type I error probabilities are controlled for each agent, independently of the other agents, the need for additional DSMB reviews due to differential information accrual across agents should not pose any issues other than logistical ones.

The Lan-DeMets spending function analog with the O'Brien-Fleming boundaries will be used to monitor the primary endpoint as a guide for the DSMB. This spending function is conservative in that priority is given to preserving power for the final analysis with the use of stringent stopping rules early in the study.

In contrast, moderately aggressive stopping rules for futility will be implemented to promote early discontinuation of agents with low probabilities for success. The futility stopping rules will be considered non-binding by the DSMB in their review of interim data. The futility boundaries are computed based on

the gamma family of spending functions (Hwang, Shih, DeCani 1990). **Figure 1** below illustrates the efficacy boundaries based on the Lan-DeMets spending function analog to O'Brien-Fleming and the futility boundaries based on a (Gamma=-2) spending function.

Figure 1: **Stopping Boundaries for Efficacy (blue shading) and Futility (pink shading).**

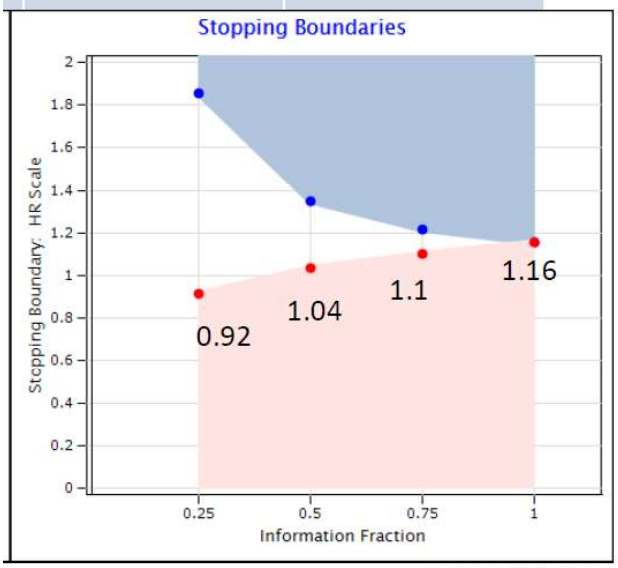

As can be seen from the figure, an RRR showing no more than a small amount of improvement in recovery time (RRR ≤ 1.04) will result in early discontinuation of the agent mid-way through information accrual in the study. When the null hypothesis is true (RRR = 1), the probability of discontinuing an agent at this point is 66%, but under the alternative, this probability is 4%.

3.4.3 ADAPTATIONS

The master protocol design with common data elements, procedures, etc., and adaptive platform design allows for seamless dropping or replacing of an arm by a new treatment. If the pooled control arm is determined inferior to another arm at an interim analysis, the DSMB will be asked to make a recommendation about possible modifications. Only concurrent data will be used for comparisons. Thus, a new arm will be compared to controls enrolled on or after the time the new arm is added and determined to be eligible to receive the agent. It is anticipated that the DSMB will informally incorporate mortality and adverse event information into the decision making regarding stopping for efficacy and futility.

At the time of the second interim analysis, there is a plan to have a blinded sample size re-estimation that allows for sample size increases of as much as 25% but no reduction. The formula of Whitehead (1993) was used to determine the expected power for the 8-point scale. That calculation includes a factor that depends on the marginal distribution of the 8-point scale lumped over the active and placebo arms. Additionally, the recommendation for the sample size adjustment will examine the overall recovery rate by day 29. It is possible that the observed recovery rates will be somewhat different from the ACTT-1 results due to changes in SoC, differences in participant mix, and random variability. Recommendations for the sample size adjustment will be based on trying to maintain ≥ 85% power for the primary endpoint and ≥ 70% power for the key secondary endpoints.

3.5 DATA SOURCES

At each study site, data will be entered on the electronic Case Report Forms (eCRFs) stored in Medidata Rave. Prior to database lock, programmed computer edit checks will be run against the database to identify discrepancies and verify reasonableness of the data. Queries to resolve discrepancies will be generated and resolved by the sites. As needed, DCRI Statistics will be able to directly download the eCRF database from Rave using SAS on Demand as Clinical Data Interchange Standards Consortium Study Data Tabulation Model (CDISC SDTM version 3.2) datasets. CDISC Analysis Data Model (ADaM version 2.1) datasets will be created by DCRI Statistics for production of tables, figures, and listings. All planned reporting will be based off of CDISC datasets, but in the case of emergent safety data, some reporting may occur from the raw eCRF data. All programs written to create analysis datasets and perform analyses will be validated according to SOPs established by the DCRI Statistical Programming group.

3.6 DOCUMENTATION CONVENTION

The statistical analyses described in this SAP, as well as production of tables, listings, and figures will be performed using SAS®, version 9.4 or higher (SAS Institute, Cary, NC). Additional statistical software may be used as needed.

3.7 VERIFICATION OF RESULTS

All tables, listings, and graphs will be verified and reviewed before considered final. The verification process will ensure that the numbers are produced by a statistically valid method and that the execution of the computations is correct. Qualified statisticians or statistical programmers employed by the DCRI who have not been previously involved in the production of the original programming will perform the verification procedures. Methods of verification include independent programming, prior to issuance of the draft statistical report, of all analysis datasets/ADaM and comparison to data listings. Tables, listings, and figures will be reviewed for accuracy, consistency with this analysis plan, consistency within tables/listings/figures, and consistency with corresponding output. Once verification is complete, all documentation of the verification process will be filed in a statistical programming documentation notebook as required by the Statistical Standard Operations Procedures of the DCRI.

3.8 SUBJECT DISPOSITION

The disposition of subjects (number randomized, number who received any amount of the randomly assigned treatment, number completing study drug administration, number who withdrew consent or discontinued from study drug early, and number lost to follow-up, and number who completed the trial) will be summarized by treatment group. The number of subjects screened for inclusion and a breakdown of reasons for exclusion will be summarized. The timing and reasons for early discontinuation of study drug and/or withdrawal from the study will be summarized by treatment group. For the calculation of percentages, subjects who die will not be included in the denominators for visits/assessments beyond their death. Treatment compliance (e.g. number of subjects who had their required infusions halted/slowed and the number of subjects with missed doses) will be summarized by treatment group. A subject listing of analysis population eligibilities will be generated. A listing of all

subjects discontinued from the study after enrollment, broken down by site and treatment group will be provided. The listing will include: reason for discontinuation, treatment group, duration of treatment, and whether or not the blind was broken. Also, for subjects who discontinued from the study after enrollment, a listing of adverse events will be provided. Subject-specific protocol deviations will be summarized by the reason for the deviation, the deviation category, treatment group, disease severity and (separately) site for all subjects. All subject-specific protocol deviations and non-subject specific protocol deviations will be included in listings.

3.9 POPULATIONS FOR ANALYSES

The primary analysis will be based on an intent-to-treat (ITT) population, including all participants randomized. Similarly, safety analyses will be based on a modified intention-to-treat (MITT) population consisting of all participants who received at least one dose or injection of each drug administered in the randomization arm (e.g. investigational agent/placebo plus SoC). Each substudy will define separate ITT and MITT populations due to the shared SoC plus placebo (or control) group. The primary analysis will be based on those participants randomized in order to achieve 788 recoveries for each pairwise comparison. Subsequent analysis will be performed on all randomized participants.

4.0 STATISTICAL ANALYSES

4.1 GENERAL APPROACH

- Statistical significance: Statistical comparisons will be performed using two-sided significance tests. An alpha level of 0.05 will determine significance (unless otherwise noted) for each agent.

- Descriptive statistics:
  - Continuous variables will be presented as n, mean, standard deviation, median, Q1, Q3, and minimum and maximum. For comparisons of treatment groups, we will use the non-parametric Wilcoxon/Mann-Whitney rank-sum test.
  - Categorical variables will be presented as percentage (number). Group comparisons will use the conventional chi-square test.
  - Binary variables will be presented as percentage (number). Group comparisons will use the conventional chi-square test or Fisher's Exact Test.

- When death is not a competing risk (for example, the endpoint includes death in the composite), differences in time-to-event endpoints by treatment will be summarized with Kaplan-Meier curves, log-rank tests, stratified log-rank tests, and Cox proportional hazards models. When death is a competing risk (for example, time to at least a one-category improvement in ordinal scale, and time to at least a two-category improvement), the same competing risk approach will be used as for the primary analysis.

- For hazard ratio and odds ratio estimates, Wald confidence intervals will be used. For other efficacy outcomes, Wilson or Score confidence intervals will be used. For safety outcomes, exact (e.g. Clopper-Pearson) confidence intervals will be used.

- Unblinding:

  Unblinding of study data for final analysis will occur for each test agent independently of other agents, consistent with the master protocol design. That is, once the planned number of recoveries required for a particular agent's comparative analysis are observed, study close-out procedures for that agent are applied for all study visits and data elements associated with the participants receiving the test agent as well as participants receiving SoC plus placebo during the randomization period for that agent (i.e., concurrently controlled participants) subset to those eligible to receive the agent. The study visits are monitored, data edits are completed, queries are resolved, database is locked, and treatment assignments are unmasked for that comparative analysis only. Note that, because control participant data are shared across agents, procedures for unmasking data for one agent's final analysis will be established to protect the ability to continue the ongoing study in a double-blinded manner for the remaining agents. It may be that recovery rates are similar enough across all agents in the study to enable a single study close-out with standard procedures. Any necessary modifications or updates to the statistical analysis should be made prior to the study unblinding.

- Handling of Missing Data:

Efforts to minimize loss-to-follow-up will be considerable. However, small amounts of missing data may occur. The plans for handling missing data follow the approach taken in the ACTT-1 study (Beigel, Tomashek et al. 2020). All attempts will be made to collect all data per protocol. Any data point that appears to be erroneous or inexplicable based on clinical judgment will be investigated as a possible outlier. If data points are identified as outliers, sensitivity analyses may be performed to examine the impact of including or excluding the outliers. Any substantive differences in these analyses will be reported.

For time to event outcomes, participants who are lost to follow-up or terminate the study prior to Day 29 and prior to observing/experiencing the event will be censored at the time of their last observed assessment. Participants who die prior to observing/experiencing the event will be censored at Day 29.

For the analyses of the secondary outcomes described in Section 4.3, the following imputation rules will be used for participants who are lost to follow-up, terminate early from the study, or do not have further outcome data available after discharge for any reason:

- Days of Non-invasive ventilation/high-flow oxygen:
    - If the subject's clinical status scale is 3 at the last observed assessment, then the subject will be considered to be on non-invasive ventilation/high-flow oxygen through Day 29. The endpoint will be total days when assessments are available plus all imputed days following the last observed assessment.
    - If the subject is not on non-invasive ventilation/high-flow oxygen at the last observed assessment, then the subject will be considered to not be on non- invasive ventilation/high-flow oxygen for the remainder of follow-up. Thus, no additional imputed days will be added to the number of days recorded on available assessments.

- Days of ventilation/ECMO:
    - If the subject's clinical status scale is 2 at the last observed assessment, then the subject will be considered to be on ventilation/ECMO through Day 29. The endpoint will be total days when assessments are available plus all imputed days following the last observed assessment.
    - If the subject is not on ventilation/ECMO at the last observed assessment, then the subject will be considered to not be on ventilation/ECMO through Day 29. Thus, no additional imputed days will be added to the number of days recorded on available assessments.

- Days of Oxygen:
    - If the subject's clinical status score is 4, 3, or 2 at the last observed assessment, then the subject will be considered to be on oxygen through Day 29. The endpoint will be total days when assessments are available plus all imputed days following the last observed assessment.
    - If the subject is not on oxygen at the last observed assessment, then the subject will be considered to not be on oxygen through Day 29. Thus, no additional imputed days will be added to the number of days recorded on available assessments.

- Days of Hospitalization

- If the subject is discharged and no further hospitalization data are available, then the subject will be assumed to not have been readmitted. Thus, no additional imputed days will be added to the number of days recorded on available assessments. If a subject dies while hospitalized, the number of days of hospitalization will be imputed as 28 days.

- NEWS
  - There will be no imputation for incomplete components of the NEWS score.

4.2 ANALYSIS OF THE PRIMARY EFFICACY ENDPOINT

The primary efficacy analysis for the comparison of each test agent plus SoC versus SoC plus placebo is a stratified test based on the Fine-Gray proportional hazards approach, where stratification is according to geographic region and baseline disease severity (i.e. protocol defined moderate vs severe disease). The approach is similar to using a stratified log-rank test on time to recovery, providing an estimate of the cumulative incidence of recovery while accounting for the competing risk of death. The primary quantity of interest, or estimand, is the RRR. With no censoring, the hazard rate in each arm can be thought of as the hazard for recovery, treating deaths as never having recovered (Fine and Gray 1999, Gooley, Leisenring et al. 1999).

The median time to event and 95% confidence interval will be summarized by treatment arm. Cumulative incidence curves for each treatment arm will be presented, supplemented with the RRR estimate, p-value, and the number of subjects at risk in each arm at Days 1, 3, 5, 7, 11, 15, 22, and 29.

- Sensitivity Analyses

  The primary analysis will be repeated in the MITT analysis population where subjects who are ineligible at baseline will be censored at enrollment. For all supplemental and sensitivity analyses, p-values will be reported and 95% confidence levels will be used for confidence interval estimates. The tabular and graphical summaries described in the previous section will be replicated for the MITT analysis.

  The primary analysis will also be repeated using the other subgroups defined in Section 4.7. Each subgroup will be considered separately and the tabular and graphical summaries described in the previous section will be replicated for each subgroup. In addition, a forest plot will be generated to display the overall treatment hazard ratio (or RRR) estimate and CI from each of the within-stratum analyses. These analyses will be performed in the ITT and MITT populations.

  Sensitivity analyses will be performed to explore subjects who recover but subsequently report a clinical score < 6. The specific analytic procedures used to explore the subjects will depend on the number of subjects who experience this scenario at the timing of their increase in score. Analyses may include repeating the above analyses, treating such subjects as not recovered or excluding such subjects, in addition to reporting rates of increased score post-recovery.

  Analyses that take into account concomitant medication will be performed. The primary analysis will be repeated, where subjects who take prohibited medications will be treated as treatment failures and will be censored at the time of medication use. Other censoring techniques and additional analyses may be performed.

A very important sensitivity analysis in the ACTT trials treated recoveries followed by death as a failure to recover. Based on the prior ACTT results we expect fewer than 1% of participants to have this outcome during the 29 days of follow-up. Finally, a set of Bayesian analyses will be conducted for the primary endpoint using optimistic, neutral, and skeptical prior as described in Wijeysundera et al. (2009).

4.3 ANALYSIS OF THE SECONDARY EFFICACY ENDPOINT(S)

The ordinal scale will be used to estimate a proportional odds model by disease strata and region. We will perform a covariate adjusted test to evaluate whether the common odds ratio for treatment is equal to one. The distribution of severity results will be summarized by treatment group as percentages. The treatment odds ratio, 95% CI, and p-value estimated from the model will be presented. Efforts to minimize loss-to-follow-up will be considerable.

The number and proportion of subjects along with 95% confidence intervals by category of clinical status will be presented by treatment arm at Days 1, 3, 5, 8, 11, 15 and 29. A figure will present stacked bar charts by day with side by side bars for each treatment arm. Histograms will be generated to display the ordinal scale value distributions over time in each treatment group.

Change in clinical status scale from baseline at specific time points will be summarized by proportions (e.g., proportion who have a 1-, 2-, 3-, or 4-point improvement or 1-, 2-, 3-, 4-point worsening). A table will present the proportion of people on Days 3, 5, 8, 11, 15 and 29 within each category of change by treatment arm along with 95% confidence intervals. The difference in proportions between treatment arms along with 95% confidence interval will also be reported. Additionally, side-by-side histograms (of an agent versus control) of the proportions with various changes will be presented.

The number and percentage of subjects who die within 14 and 28 days following randomization will be presented by treatment arm (denominator for the percentages will be the number of subjects in the MITT population in each treatment arm). The 14- and 28-day mortality rate, which will take into account the amount of follow-up time for each subject will be calculated and presented. Mortality through Day 29 will also be analyzed as a time to event endpoint, similar to the analysis of the primary endpoint. A table will present median time to event along with 95% confidence intervals overall for each treatment arm along with the hazard ratio estimate and log rank p-values. Differences in time-to-event endpoints by treatment will be summarized with Kaplan-Meier curves.

Differences in total severity score (TSS) over time will assessed by fitting a longitudinal mixed model to the 8-point clinical scale value on each day of assessment (in hospital plus Days 15, 22, 29). The average difference across days in study (analogous to the area under the severity curve) will be estimated with 95% confidence limits in the context of the longitudinal model. The longitudinal mixed model will be developed as follows. Differences in total severity score (TSS) over time will be assessed by fitting a longitudinal mixed effects model to the 8-point clinical scale calculated daily from randomization until the day 29 assessment. The average of the 8-point scale over days 1 to 29 (analogous to the area under the severity curve) will be estimated by treatment group with 95% confidence limits in the context of the longitudinal model. The value of the ordinal scale on day t will be assumed to depend on the

patient's prior outcome trajectory only through the prior day's value, treatment group assignment, and an unobserved patient-specific random intercept. The general form of the model will be:

$$\Pr\left(Y_{it} \le k \middle| H_{i,t-1}, Y_{i.t-1} = x, Z_i = z, \boldsymbol{\alpha}, \boldsymbol{\beta}, \boldsymbol{\gamma}, e_i\right) \equiv \pi_{itk|xz},$$

$$\log \frac{\pi_{itk|xz}}{1 - \pi_{itk|xz}} = \alpha_{kx} + t\beta_1 + t^2\beta_2 + z(\gamma_0 + t\gamma_1 + t^2\gamma_2) + e_i,$$

$$e_i \sim N(0, \sigma^2),$$

where Y_{it} is outcome of the i-th patient on day t, $H_{i,t-1} = (Y_{i1}, \dots, Y_{i,t-1})$ is the patient's outcome trajectory through day $t-1$, Z_i is a binary indicator of treatment group assignment (1=active agent + SoC 0=placebo + SoC), e_i is an unobserved patient-specific random intercept, and the symbols $\alpha_{kx}, \beta_1, \beta_2, \gamma_0, \gamma_1\gamma_2, \sigma^2$ represent unknown fixed effect parameters to be estimated from the data. The model assumes that on each day, the odds of an outcome in one of the k lowest categories depends jointly on treatment assignment, days since randomization, and the patient's prior day outcome category. Parameters will be estimated by maximum likelihood using 1 record per patient per day from the first day post-randomization until the earlier of day 28, death or loss to follow-up. Days between death and day 28 do not contribute to the likelihood because death is an absorbing state and hence $\alpha_{11} = \infty$ and $\alpha_{21} = \alpha_{31} \cdots = \alpha_{81} = -\infty$. The estimation procedure will assume that loss to follow-up is conditionally ignorable given treatment assignment and the patient's current observed outcome trajectory. A feature of the trial's outcome assessment schedule is that only patients who are hospitalized have their outcome assessed on days other than 8, 11, 15, 22, and 29. If a patient is not hospitalized, the patient's outcome category will be at least 7 but the exact value (7 versus 8) will only be collected on days 8, 11, 15, 22, and 29. To mitigate potential bias from informatively missing data, categories 7 and 8 will be combined into a single category for this analysis. This modification guarantees that a patient will have non-missing data on all days 1 through 28 if the patient's vital status and hospitalization status is known on each day and the patient's outcome is assessed per protocol on each day that the patient is alive and in-hospital. Additional considerations for this analysis will be detailed in a later version of the Statistical Analysis Plan.

Duration of oxygenation days will be summarized in a table using mean (median) and standard deviations (quartiles) by treatment arm. Point estimates and associated 95% confidence intervals will be obtained using a linear model with treatment group and baseline severity score as a categorical predictor. Duration of hospitalization days, duration of non-invasive ventilation/high flow oxygen days, and duration of invasive Mechanical Ventilation/ECMO days will be analyzed using a similar approach.

The incidence of new oxygen use will be analyzed by treatment arm. This will only include subjects in category 4 at enrollment. New use will be identified by a post-enrollment score of at least 5. The number and percent of subjects reporting new use and 95% confidence interval will be reported. Comparisons between treatment arms will be presented as differences in proportions with 95% confidence intervals.

The incidence of new Non-Invasive Ventilation/High-Flow Oxygen use will be analyzed by treatment arm. This will only include subjects in category 4 or 5 at enrollment. The incidence of new oxygen use will be analyzed by treatment arm. This will only include subjects in category 4 at enrollment. New use will be identified by a post-enrollment score of at least 6. The number and percent of subjects reporting new use and the 95% confidence interval will be reported. Comparisons between treatment arms will be presented as differences in proportions with 95% confidence intervals.

The incidence of new Non-Invasive Ventilation/High-Flow Oxygen use will be analyzed by treatment arm. This will only include subjects in category 4, 5, or 6 at enrollment. The incidence of new oxygen use will be analyzed by treatment arm. This will only include subjects in category 4 at enrollment. New use will be identified by a post-enrollment score of 7. The number and percent of subjects reporting new use and the 95% confidence interval will be reported. Comparisons between treatment arms will be presented as differences in proportions with 95% confidence intervals.

4.4 SAFETY ANALYSES

Safety endpoints include death through Day 28 (as assessed at Day 29), SAEs, and Grade 3 and 4 AEs. These events will be analyzed individually and as a composite endpoint. The time to this composite endpoint will be defined as the elapsed time (in days) from Day 1 to the earliest date of any of the events. Any subjects that are lost to follow-up or terminated early prior to experiencing any of the events will be censored at the day of their last observed assessment. Subjects who complete follow-up but do not experience any of the events will be censored at the day of their Day 29 visit. Time-to-event methods will be used for death and the composite endpoint. A table will present the hazard ratio estimate with 95% confidence intervals and log rank p-values. Differences in time-to-event endpoints by treatment will be summarized with cumulative incidence curves.

Similar analyses will be conducted for the following AEs:

- Any Grade 2 suspected drug-related hypersensitivity reactions associated with study product administration will be reported as an AE.
- Any newly diagnosed infection (other than COVID-19) at any time during the study.
- Any AEs leading to dose modification
- Any AEs leading to discontinuation from the study.

AEs will be coded in accordance with the current version of the Medical Dictionary for Regulatory Activities (MedDRA). AEs will be graded with regard to severity according to criteria defined in the Common Terminology Criteria for Adverse Events version 5.0 (CTCAE) and relationship to COVID-19 or study intervention. Each AE will be counted once only for a given participant. AEs will be presented by system organ class, preferred term (PT), duration (in days), start- and stop-date. AEs leading to premature discontinuation from the study intervention and serious AEs will be presented in a listing.

4.4.1 LABORATORY DATA

Clinical safety laboratory adverse events are collected Day 1, 3, 5, 8, 11 and Day 15 and 29 if able to return to clinic or still hospitalized. Parameters evaluated include white blood cell count, differential, Hgb, PLT, creatinine, glucose, total bilirubin, AST, ALT, ALP, PT/INR, d-dimer, cardiac troponin, and C-reactive protein. Laboratory safety parameters will be graded according to the Division of AIDS (DAIDS) Table for Grading the Severity of Adult and Pediatric Adverse Events, version 2.1 (July 2017). The distribution of abnormal chemistry and hematology laboratory results by maximum severity, time point, and treatment group will be presented. In addition, the distribution of Grade 3 and 4 chemistry and hematology laboratory results by maximum severity, time point, disease severity and treatment group will be presented. Descriptive statistics including mean, median, standard deviation, maximum, and minimum values and change from baseline by time point, for all and each chemistry and hematology laboratory parameter will be summarized by disease severity and treatment arm. Changes in chemistry

and hematology laboratory values will be presented in line graphs over time with mean and SD plotted by disease severity and treatment arm.

Descriptive statistics for baseline and post-baseline time points will be generated for abnormal laboratory data (hematology and chemistry values). Additionally, shift tables will be presented. The shift tables tabulate the number of lab values determined to be “Normal” and “Abnormal” at baseline and post-baseline time points. The classifications on shift tables will categorize lab values as “Normal” when they are in acceptable range, using a combination of clinician judgment specific to each lab test, and the reference ranges. Laboratory values outside the normal range will be graded on the Labs Listing as 1x, 2x, 3x, 5x, or 10x the upper limit (or .5x, 1x the lower limit) of normal value based on appropriate increases or decreases. Laboratory data will also be summarized graphically to show the magnitude and changes in individual subject values over time relative to normal ranges. Details of any abnormalities will be listed.

4.4.2 VITAL SIGNS

Vital sign measurements include pulse, systolic blood pressure, respiratory rate, SpO2 and oral temperature. Vital signs will be presented in a listing.

4.4.3 PRIOR AND COMCOMITANT MEDICATIONS

Concomitant medications will be coded to the Anatomical Therapeutic Classification using the WHO Drug Dictionary. The use of prior, concomitant medications taken, and changes in concomitant medications during the study will be recorded on the CRFs. The use of concomitant medications during the study will be summarized by ATC1, ATC2 code, disease severity, and treatment group for the MITT population. The summaries will be repeated for each test agent.

4.4.4 TREATMENT COMPLIANCE

Treatment compliance will be assessed based upon the administration rules for each of the test agents. Details for each test agent will be provided in an addendum.

4.4.5 PREGNANCY TESTS

Pregnancy tests for females of childbearing potential are performed as part of screening laboratory test on Day -1/Day 1. For any female participants in the MITT population who become pregnant during the study, every attempt will be made to follow these participants to completion of pregnancy to document the outcome, including information regarding any complications with pregnancy and/or delivery. A set of listings of pregnancies and outcomes will be presented.

4.5 EXPLORATORY ANALYSES

An exploratory analysis will compare treatment efficacy estimates for each agent for the primary endpoint and the two key secondary endpoints. Specifically, the probability of falling into category “i” or better will be compared between arms for each baseline severity score category. Exploratory analyses

will also compare the difference between each test agent plus SoC and SoC plus placebo in TSS at each study day for which the clinical scale is administered (in-hospital and at Days 15, 22, and 29).

4.6 BASELINE DESCRIPTIVE STATISTICS

Baseline characteristics will be summarized by test agent versus control for each agent and study population. For continuous measures the mean and standard deviation will be summarized. Categorical variables will be described by the proportion in each category (with the corresponding sample size numbers). Baseline characteristics will include age, sex, race, ethnicity, symptom duration, hypertension, obesity, diabetes, coronary disease, history of heart failure, history of cancer, asthma, COPD, chronic pulmonary disease, tuberculosis, HIV, severe liver disease, severe kidney disease, respiratory support received, days since hospitalization, and baseline 8-point ordinal score.

4.7 SUB-GROUP ANALYSES

The following subgroups have been pre-specified for the primary outcome of time to recovery and key secondary outcomes:

- Geographic region
- Duration of symptoms prior to randomization (<= Median; > Median)
- Duration of symptoms prior to randomization (based on quartiles)
- Hospitalization duration prior to randomization (<= Median, > Median)
- Baseline disease severity based on the 8-point ordinal scale
- Age (<40; 40-64; 65 and older)
- Race (White; Black/African American; Asian; Other)
- Ethnicity (Hispanic, non-Hispanic)
- Sex (Female; Male)
- Comorbidities: Any, 1, 2, 3+
- Baseline use of dexamethasone
- Individual comorbidities: hypertension, obesity, diabetes, coronary disease, history of heart failure, history of cancer, asthma, COPD, chronic pulmonary disease, severe liver disease, severe kidney disease

In addition, Fine-Gray models will be run within each of the disease severity strata to obtain stratum-specific estimates of the treatment hazard ratio. A forest plot will display confidence intervals across subgroups. Interaction tests will be conducted to determine whether the effect of treatment varies by subgroup. These subgroup analyses will be carried out separately for each test agent.

4.8 REFERENCES

Beigel, J. H., K. M. Tomashek, L. E. Dodd, A. K. Mehta, B. S. Zingman, A. C. Kalil, E. Hohmann, H. Y. Chu, A. Luetkemeyer, S. Kline, D. Lopez de Castilla, R. W. Finberg, K. Dierberg, V. Tapson, L. Hsieh, T. F. Patterson, R. Paredes, D. A. Sweeney, W. R. Short, G. Touloumi, D. C. Lye, N. Ohmagari, M. D. Oh, G. M. Ruiz-Palacios, T. Benfield, G. Fätkenheuer, M. G. Kortepeter, R. L. Atmar, C. B. Creech, J. Lundgren, A. G. Babiker, S. Pett, J. D. Neaton, T. H. Burgess, T. Bonnett, M. Green, M. Makowski, A. Osinusi, S. Nayak and H. C. Lane (2020). "Remdesivir for the Treatment of Covid-19 - Preliminary Report." N Engl J Med.

Cao, B., Y. Wang, D. Wen, W. Liu, J. Wang, G. Fan, L. Ruan, B. Song, Y. Cai, M. Wei, X. Li, J. Xia, N. Chen, J. Xiang, T. Yu, T. Bai, X. Xie, L. Zhang, C. Li, Y. Yuan, H. Chen, H. Li, H. Huang, S. Tu, F. Gong, Y. Liu, Y. Wei, C. Dong, F. Zhou, X. Gu, J. Xu, Z. Liu, Y. Zhang, H. Li, L. Shang, K. Wang, K. Li, X. Zhou, X. Dong, Z. Qu, S. Lu, X. Hu, S. Ruan, S. Luo, J. Wu, L. Peng, F. Cheng, L. Pan, J. Zou, C. Jia, J. Wang, X. Liu, S. Wang, X. Wu, Q. Ge, J. He, H. Zhan, F. Qiu, L. Guo, C. Huang, T. Jaki, F. G. Hayden, P. W. Horby, D. Zhang and C. Wang (2020). "A Trial of Lopinavir-Ritonavir in Adults Hospitalized with Severe Covid-19." N Engl J Med 382(19): 1787-1799.

Fine, J. P. and R. J. Gray (1999). "A Proportional Hazards Model for the Subdistribution of a Competing Risk." Journal of the American Statistical Association 94(446): 496-509.

Gooley, T. A., W. Leisenring, J. Crowley and B. E. Storer (1999). "Estimation of failure probabilities in the presence of competing risks: new representations of old estimators." Stat Med 18(6): 695-706.

Huang, C., Y. Wang, X. Li, L. Ren, J. Zhao, Y. Hu, L. Zhang, G. Fan, J. Xu, X. Gu, Z. Cheng, T. Yu, J. Xia, Y. Wei, W. Wu, X. Xie, W. Yin, H. Li, M. Liu, Y. Xiao, H. Gao, L. Guo, J. Xie, G. Wang, R. Jiang, Z. Gao, Q. Jin, J. Wang and B. Cao (2020). "Clinical features of patients infected with 2019 novel coronavirus in Wuhan, China." Lancet 395(10223): 497-506.

Hwang IK, Shih WJ, J S De Cani JS (1990). “Group sequential designs using a family of type I error probability spending functions” Stat Med; 9(12):1439-45.

Smith, G. B., D. R. Prytherch, S. Jarvis, C. Kovacs, P. Meredith, P. E. Schmidt and J. Briggs (2016). "A Comparison of the Ability of the Physiologic Components of Medical Emergency Team Criteria and the U.K. National Early Warning Score to Discriminate Patients at Risk of a Range of Adverse Clinical Outcomes." Crit Care Med 44(12): 2171-2181.

Whitehead J. (1993) “Sample Size Calculations for Ordered Categorical Data.” Stat Med; vol. 12, 2257-2271.

Wijeysundera DN, Austin PC, Hux JE, Beattie WS, Laupacis A (2009). “Bayesian statistical inference enhances the interpretation of contemporary randomized controlled trials.” J Clin Epidemiol; 62: 13-21 e15.

4.9 APPENDICES

APPENDIX 1. ACTIV-1 Roster of Statisticians

Team	Name	Email	Attends DSMB Meetings
Unblinded Statistical Team	Michael Proschan	[redacted]	X
	Tyler Bonnet	[redacted]	X
	Jyotsna Garg	[redacted]	X
	Eric Yow	[redacted]	X
	Zhen Huang	[redacted]	
	Hwasoon Kim	[redacted]	
	Hussein Al-Khalidi	[redacted]	X
	Sean O'Brien	[redacted]	X
Blinded Statistical Team	Lisa LaVange	[redacted]	X
	Steven McNulty	[redacted]	X
	Adam Silverstein	[redacted]	
	Pearl Zakroysky	[redacted]	X
	Susan Halabi	[redacted]	X
	Kevin Anstrom	[redacted]	X

Statistical Analysis Plan ACTIV1_IM_25NOV2020_clean

Final Audit Report 2020-12-04

Created:	2020-12-02
By:	Pearl Zakroysky (pz33@duke.edu)
Status:	Signed
Transaction ID:	CBJCHBCAABAAvlICGJWy11ZJIO7QbAkWAPOjLqEjAL4b

"Statistical Analysis Plan ACTIV1_IM_25NOV2020_clean" History

Document created by Pearl Zakroysky (pz33@duke.edu)
2020-12-02 - 2:21:36 PM GMT- IP address: 152.16.191.126

Document emailed to danny benjamin (danny.benjamin@duke.edu) for signature
2020-12-02 - 2:23:44 PM GMT

Email viewed by danny benjamin (danny.benjamin@duke.edu)
2020-12-02 - 5:26:20 PM GMT- IP address: 99.132.141.156

Document e-signed by danny benjamin (danny.benjamin@duke.edu)
Signature Date: 2020-12-02 - 5:26:55 PM GMT - Time Source: server- IP address: 99.132.141.156

Document emailed to Jane C. Atkinson (jatkinso@mail.nih.gov) for signature
2020-12-02 - 5:26:57 PM GMT

Email viewed by Jane C. Atkinson (jatkinso@mail.nih.gov)
2020-12-02 - 6:24:07 PM GMT- IP address: 128.231.234.6

Document e-signed by Jane C. Atkinson (jatkinso@mail.nih.gov)
Signature Date: 2020-12-02 - 6:26:26 PM GMT - Time Source: server- IP address: 128.231.234.6

Document emailed to Lisa LaVange (lisa.lavange@unc.edu) for signature
2020-12-02 - 6:26:28 PM GMT

Email viewed by Lisa LaVange (lisa.lavange@unc.edu)
2020-12-04 - 1:36:39 AM GMT- IP address: 99.69.18.80

Document e-signed by Lisa LaVange (lisa.lavange@unc.edu)
Signature Date: 2020-12-04 - 1:39:40 AM GMT - Time Source: server- IP address: 99.69.18.80

Document emailed to Michael Proschan (proscham@niaid.nih.gov) for signature

2020-12-04 - 1:39:43 AM GMT

Email viewed by Michael Proschan (proscham@niaid.nih.gov)

2020-12-04 - 2:21:38 PM GMT- IP address: 128.231.234.67

Document e-signed by Michael Proschan (proscham@niaid.nih.gov)

Signature Date: 2020-12-04 - 3:44:48 PM GMT - Time Source: server- IP address: 128.231.234.67

Document emailed to William Powderly (wpowderly@wustl.edu) for signature

2020-12-04 - 3:44:50 PM GMT

Email viewed by William Powderly (wpowderly@wustl.edu)

2020-12-04 - 3:51:31 PM GMT- IP address: 128.252.34.131

Document e-signed by William Powderly (wpowderly@wustl.edu)

Signature Date: 2020-12-04 - 3:53:30 PM GMT - Time Source: server- IP address: 128.252.34.131

Agreement completed.

2020-12-04 - 3:53:30 PM GMT

Adobe Sign

Master Protocol for Immune Modulators for Treating COVID-19

Protocol: ACTIV-1 IM

STATISTICAL ANALYSIS PLAN (SAP)

Protocol Version 2.0

Protocol Version Date: 02 DEC 2020

SAP Version Number: 5.0

11 APR 2022

Daniel K. Benjamin, MD, MPH, PhD

Kiser-Arena Distinguished Professor, Duke University Pediatrics

Duke Clinical Research Institute

300 W. Morgan Street, Suite 800

Durham Center

Durham, NC 27701

Tel: 919-668-7081

E-mail: [redacted]

TABLE OF CONTENTS

List of Abbreviations 5
1.0 Study and Document Overview 7
2.0 Study Objectives and Estimands 8
3.0 Statistical Hypotheses and Endpoints 11
3.1 Study Definitions and Derived Variables 14
3.1.1 Visit Day 14
3.1.2 8-Point Ordinal Scale 14
3.1.3 Measures of Clinical Support, Limitations, and Infection Control 14
3.1.4 Extrapulmonary Manifestations 15
3.1.5 National Early Warning Score (NEWS) 16
3.2 Power and Sample Size 18
3.3 Randomization Scheme 19
3.4 Interim Analyses and Data Monitoring 20
3.4.1 Interim Safety Analyses 21
3.4.2 Interim Analyses for Futility and Efficacy Review 21
3.4.3 Adaptations 23
3.5 Data Sources 25
3.6 Documentation Convention 26
3.7 Verification of Results 26
3.8 Subject Disposition 26
3.9 Populations for Analyses 27
4.0 Statistical Analyses 28
4.1 General Approach 28
4.2 Analysis of the Primary and Key Secondary Efficacy Endpoints 31
4.3 Analysis of the Other Secondary Efficacy Endpoints 36
4.4 Safety Analyses 39
4.4.1 Laboratory Data 40

4.4.2 Vital Signs 41

4.4.3 Prior and Comcomitant Medications 41

4.4.4 Treatment Compliance 41

4.4.5 Pregnancy Tests 41

4.5 Exploratory Analyses 41

4.6 Baseline Descriptive Statistics 42

4.7 Sub-Group Analyses 42

4.8 References 44

4.9 Appendices 46

LIST OF ABBREVIATIONS

ACTIV	Accelerating COVID-19 Therapeutic Interventions and Vaccines
ADaM	Analysis Data Model
AE	Adverse Event
ALT	Alanine transaminase
AST	Aspartate transaminase
ATC	Anatomical Therapeutic Classification
BMI	Body Mass Index
CDISC	Clinical Data Interchange Standards Consortium
CI	Confidence Interval
COVID-19	Coronavirus
CRP	C-Reactive Protein
CTCAE	Common Terminology Criteria for Adverse Events
DAIDS	Division of AIDS
DCRI	Duke Clinical Research Institute
DSMB	Data and Safety Monitoring Board
ECMO	Extracorporeal Membrane Oxygenation
eCRF	Electronic Case Report Form
EOS	End of Study
ET	Early Termination
FU	Follow-up
ITT	Intent-to-Treat
LLN	Lower Limit of Normal
MedDRA	Medical Dictionary of Regulatory Activities
MITT	Modified Intent-to-Treat

NEWS	National Early Warning Score
PT	Preferred Term
Q1	25th Percentile (1st Quartile)
Q3	75th Percentile (3rd Quartile)
RR	Respiratory Rate
RRR	Recovery Rate Ratio
SAE	Serious Adverse Event
SAP	Statistical Analysis Plan
SARS-CoV-2	Severe Acute Respiratory Syndrome Coronavirus 2
SD	Standard Deviation
SDTM	Study Data Tabulation Model
SE	Standard Error
SoC	Standard of Care
ULN	Upper Limit of Normal
WBC	White Blood Cell

1.0 STUDY AND DOCUMENT OVERVIEW

ACTIV-1 IM is a master protocol designed to evaluate multiple investigational agents for the treatment of moderately or severely ill patients infected with severe acute respiratory syndrome coronavirus 2 (SARS-CoV-2). ACTIV-1 IM is a randomized, controlled, double-blinded trial which will evaluate each agent with respect to speed of recovery, mortality, illness severity, and hospital resource utilization. Each agent will be evaluated as add-on therapy to the standard of care (SoC) in use at the local clinics. The SoC may change during the course of the study based on other research findings. Comparisons of the investigational agents among themselves is not a research objective.

This document describes the planned statistical analyses that will be conducted at the completion of the ACTIV-1 IM study for each of the investigational agents. Once the initial unblinded data review has occurred, only the blinded statistics team members will be allowed to modify the SAP (see **Appendix 1** for the list of blinded and unblinded statisticians).

The study population corresponds to moderately and severely ill adults (≥18 years old) with the coronavirus disease 2019 (COVID-19) virus. Recruitment will target patients already hospitalized for treatment of COVID-19 infection as well as patients being treated for COVID-19 infection in Emergency Departments while waiting to be admitted to the hospital. Patients both in and out of the intensive care unit (ICU) will be included in the study population. Enrollment began in October 2020 with 3 agents selected for initial evaluation. Up to 5 agents may be evaluated in ACTIV-1 IM.

The study period is 29 days, with assessments on each day of the hospital stay. If the participants are discharged from the hospital, they will have a study visit at Days 8, 11, 15, 22, and 29. For discharged participants, it is preferred that the Day 8, 11, 15, and 29 visits are in person to obtain safety laboratory tests and blood (serum and plasma) samples for secondary research (Day 29) as well as clinical outcome data. Clinical outcome is assessed for the previous day; for example, study visit Days 8, 11, 22, and 29 represent data for Days 7, 10, 21, and 28. However, infection control or other restrictions may limit the ability of the participant to return to the clinic. In this case, these visits may be conducted by phone, and only clinical data will be obtained. The Day 22 visit does not have laboratory tests or collection of samples and is conducted by phone. There will also be a safety and clinical status assessment at 60 days conducted by phone. Treatment periods may vary by agent.

The trial is adaptive in that interim analyses are planned to assess the futility of each agent, with the goal of discontinuing those with lower probabilities of success to more effectively utilize trial resources for the remaining agents. Additionally, interim analyses are planned for early stopping for efficacy.

Alpha spending functions are used to appropriately control the probability of making an erroneous conclusion at the interim and final analyses. Safety monitoring will be performed throughout the trial, and formal stopping rules for each agent will be adopted. The Data and Safety Monitoring Board (DSMB) established for ACTIV-1 IM will have oversight responsibility for the study.

The effectiveness of each therapeutic agent as add-on therapy to SoC will be evaluated based on the primary endpoint of time to recovery through Day 28. The sample size requirements are based on the ability to detect a moderate improvement in time to recovery for each agent. A total of 788 recoveries are required for each comparison to provide approximately 85% power to detect a recovery rate ratio (RRR) of 1.25. Assuming 73% of participants achieve recovery within 28 days, consistent with the ACTT-1 (Beigel, Tomashek et al. 2020) results, the total sample size to evaluate 1, 2, and 3 agents in ACTIV-1 IM is approximately 1080, 1620, and 2160, respectively. Because each agent is being compared to SoC plus placebo with no between-agent comparisons, no multiplicity adjustments for multiple agents are planned.

2.0 STUDY OBJECTIVES AND ESTIMANDS

Primary Objective:

- To evaluate the clinical efficacy of different investigational therapeutics relative to the control arm in adults hospitalized with COVID-19 with respect to time to recovery through Day 28 from randomization.

Key Secondary Objectives:

- To evaluate the clinical efficacy of different investigational therapeutics relative to the control arm in adults hospitalized with COVID-19 according to:
    - Clinical status (8-point ordinal scale) assessed on Day 14 and Day 28
    - Mortality

Summaries of the questions of interest and descriptions of the estimand attributes are shown in **Table 1**.

Table 1. Study Objectives and Estimands.

**Question of Interest**	**Objective Description / Study Population**	**Endpoint**	**Intercurrent Events**	**Population Summary**
Does drug X reduce the time-to-recovery compared with standard-of-care alone for patients hospitalized with moderate or severe COVID-19?	Primary Objective of the Study / All Randomized Participants	Time to recovery defined as the first day with an 8-point ordinal scale score of ≥ 6.	Death from any cause (a semi-competing event). Once an individual has experienced death, they no longer have the opportunity to 'recover'. All other intercurrent events will be ignored.	Recovery rate ratio (RRR) and its associated confidence interval from a Fine-Gray model. Values greater than 1.0 indicate a benefit for drug X. A value of 2.0 suggests a larger benefit than 1.25. Stratification factors will include region and baseline severity (2, 3, 4, or 5). Other covariates in the model will include age and sex.
Does drug X improve the ordinal response of the 8-point ordinal scale compared with standard-of-care alone?	Key Secondary Objective of the Study / All Randomized Participants*	The 8-point ordinal scale score on Day 14. A supplemental analysis will be completed on the same scale at Day 28.	Deaths are placed as the worst possible score on the ordinal scale. All other intercurrent events will be ignored in these analyses.	Odds ratio estimate based on a proportional odds model. Values above 1.0 suggest benefit from drug X. The model will include baseline severity (2, 3, 4, or 5), region, sex, and age (continuous) as covariates.
Does drug X reduce the rate of death over 28 days compared with standard-of-care alone?	Key Secondary Objective of the Study / All Randomized Participants*	Death observed between randomization through Day 28	All other intercurrent events will be ignored in these analyses.	An odds ratio from a logistic regression model with indicator variables for treatment group,

				8-point ordinal scale (2, 3, 4, or 5) at baseline, region, sex, and age (continuous).

*For the test agent and pooled control group

Additional Secondary Objectives:

- To evaluate the clinical efficacy of different investigational therapeutics relative to the control arm as assessed by clinical severity and hospitalization.
- To evaluate clinical status at Day 60.
- To evaluate the safety of different investigational therapeutics relative to the control arm.
- To evaluate the impact of different investigational therapeutics relative to the control arm of extrapulmonary manifestations of COVID-19.

Exploratory Objectives:

- To assess the National Early Warning Score (NEWS).

3.0 STATISTICAL HYPOTHESES AND ENDPOINTS

- The primary null hypothesis being tested is that time-to-recovery does not differ between each test agent (plus SoC) and the pooled control group, consisting of patients receiving SoC plus placebo. The alternative hypothesis is that the test agent and pooled control group differ in time-to-recovery with the hope that the test agent group has a shorter time-to-recovery than the pooled control group although a two-tailed test will be used. If Drug A, B, and C denote the initial set of agents to be tested in ACTIV-1 IM plus SoC, and S denotes patients receiving SoC plus placebo, then the primary hypotheses are:

H_{01}: $TR_A = TR_S$ vs H_{A1}: $TR_A \neq TR_S$

H_{02}: $TR_B = TR_S$ vs H_{A2}: $TR_B \neq TR_S$

H_{03}: $TR_C = TR_S$ vs H_{A3}: $TR_C \neq TR_S$

- **Primary Efficacy Endpoint -** Time to recovery through Day 28

  Day of recovery is defined as the first day on which the participant satisfies one of the following three categories from the 8-point ordinal scale (defined in Section 3.1.2):
  - Hospitalized, not requiring supplemental oxygen – no longer requires ongoing medical in-patient care;
  - Not hospitalized, limitation on activities and/or requiring home oxygen;
  - Not hospitalized, no limitations on activities.

- There are two key secondary hypotheses that will be evaluated as supportive evidence.

- **Key Secondary Endpoint 1** - Clinical status (8-point ordinal scale) on Day 14

  The null hypothesis to be tested is that the odds of improvement on the 8-point ordinal scale is the same for the placebo and experimental treatment arms (i.e., the common odds ratio is 1) on Day 14. For this comparison, the parameter of interest is the "common odds ratio," which quantifies the shift in the severity distribution resulting from treatment. The odds ratio will be defined such that a value greater than 1 implies better outcomes with active drug compared to placebo control.

- **Key Secondary Endpoint 2** - Mortality

  The null hypothesis to be tested is that there is no difference in mortality rates between treatment groups. Mortality through Day 28 will be analyzed using a logistic regression model with an indicator variable for the treatment group, 8-point ordinal scale (2, 3, 4, or 5) at baseline, region, sex, and age.

- Additional Secondary Endpoints
  - Time to an improvement of one category and two categories from Day 0 (baseline) using the 8-point ordinal scale.
  - Mean change in the 8-point ordinal scale from baseline to Days 2, 4, 7, 10, 14, 21, 28, and 60.
  - Differences in total severity score (TSS): 8-point ordinal scale summarized as a daily score (for days collected) averaged over time from baseline through Day 28.
  - Days alive and free of supplemental oxygen through 28.
  - Incidence and duration of new oxygen use during the study. This includes new supplemental oxygen, non-invasive ventilation/high flow oxygen use, and invasive mechanical ventilation/extracorporeal membrane oxygenation (ECMO).
  - Days alive and free of non-invasive ventilation/high flow oxygen use through Day 28.
  - Incidence and duration of new non-invasive ventilation or high flow oxygen use during the study. This includes new non-invasive ventilation or high flow oxygen use as well as invasive mechanical ventilation/ECMO.
  - Days alive and free of invasive mechanical ventilation/ECMO through Day 28.
  - Incidence and duration of new mechanical ventilation/ECMO use during the study.
  - Days alive and out of hospitalization through Day 28.

- Key Safety Endpoints
  - Serious Adverse Events (SAEs)
  - Grade 3 and 4 Adverse Events (AEs)
  - Baseline laboratory tests include CBC with differential (including absolute neutrophil count and absolute lymphocyte count), ALT, AST, ALP, total bilirubin, creatinine (with calculated eGFR), glucose, Troponin, PT/INR, d-dimer, and C-reactive protein. *On Days 3, 5, 8, 11, 15, and 29, safety laboratory tests include WBC with differential, hemoglobin, platelets, ALT,*

*AST, ALP, total bilirubin, creatinine (with calculated eGFR), and glucose. On Day 8 or on day of discharge (if less than 24 hours prior to Day 8), the following **additional** laboratory assessments are performed: Troponin, PT/INR, d-dimer, and C-reactive protein.*
    - Incidence of individual and “any” specified extrapulmonary manifestations up to Day 29

- Exploratory Endpoints
    - NEWS assessed daily while hospitalized (Day 1 – discharge) and on Days 15 and 29

3.1 STUDY DEFINITIONS AND DERIVED VARIABLES

3.1.1 VISIT DAY

The baseline visit is the date of randomization. Post-baseline study day visits are documenting the clinical information through the completion of the prior day (e.g. Day 2 is collecting the post-randomization data Day 1 and Day 29 is collecting data that encompasses all of Day 28). However, we will present visit day as actual day that is being assessed.

3.1.2 8-POINT ORDINAL SCALE

- The 8-point ordinal scale is the basis for the primary and key secondary clinical endpoints in the study, namely, time to recovery, improvement in disease severity, and mortality.
- The scale used in this study is as follows (from worst to best):
  - 1: Death;
  - 2: Hospitalized, on invasive mechanical ventilation or ECMO;
  - 3: Hospitalized, on non-invasive ventilation or high flow oxygen devices;
  - 4: Hospitalized, requiring supplemental oxygen;
  - 5: Hospitalized, not requiring supplemental oxygen – requiring ongoing medical in-patient care (COVID-19 related or otherwise);
  - 6: Hospitalized, not requiring supplemental oxygen – no longer requires ongoing medical in-patient care;
    - This would include those kept in hospital for quarantine/infection control/social reasons, awaiting bed in rehabilitation facility or homecare, etc.
  - 7: Not hospitalized, limitation on activities and/or requiring home oxygen;
  - 8: Not hospitalized, no limitations on activities

3.1.3 MEASURES OF CLINICAL SUPPORT, LIMITATIONS, AND INFECTION CONTROL

The participant's clinical status will be captured on each study day while hospitalized through Day 29, for the previous day. Clinical data status is measured twice on Day 1, once at the time of randomization and once at the Day 2 visit for the post-randomization time period. If a participant is discharged prior to Day 15, clinical status is collected on Day 8, Day 11, Day 15, and Day 29, if the participant returns for an in-person clinic visit or by phone if an in-person visit is not possible. Clinical status will also be captured on Day 22 during a phone visit. Clinical status is largely measured by the 8-point ordinal scale and the NEWS. Unlike the NEWS, the ordinal scale can also be evaluated over the phone if the discharged participant is unable to return for visits on Day 8, 11, 15, or 29 as well as on Day 22.

Improvement will be defined as an increase of at least one point on the 8-point scale compared to the baseline value (e.g. from 4 to 5) and the time to improvement will be defined as the elapsed time (in days) from baseline to the earliest day of observed improvement.

Any subjects that are lost to follow-up or terminated early prior to an observed improvement will be censored at the day of their last observed assessment.

3.1.4 EXTRAPULMONARY MANIFESTATIONS

The presence of the extrapulmonary manifestation of disease during the course of hospitalization will be assessed and reported at discharge. These tables will be reported with stratification by the participant's baseline clinical status. Specifically, clinical organ failure is defined by development of any one or more of the following clinical events:

a. Respiratory dysfunction:

1. Respiratory failure defined as receipt of high flow nasal oxygen, noninvasive ventilation, invasive mechanical ventilation or ECMO

b. Cardiac and vascular dysfunction:

1. Myocardial infarction
2. Myocarditis or pericarditis
3. Congestive heart failure: new onset NYHA class III or IV, or worsening to class III or IV
4. Hypotension requiring institution of vasopressor therapy

c. Renal dysfunction:

1. New requirement for renal replacement therapy

d. Hepatic dysfunction:

1. Hepatic decompensation

e. Neurological dysfunction

1. Acute delirium
2. Cerebrovascular event (stroke, cerebrovascular accident [CVA])
3. Transient ischemic events (i.e., CVA symptomatology resolving <24 hrs)
4. Encephalitis, meningitis or myelitis

f. Hematological dysfunction:

1. Disseminated intravascular coagulation
2. New arterial or venous thromboembolic events, including pulmonary embolism and deep vein thrombosis
3. Major bleeding events (>2 units of blood within 24 hours, bleeding at a critical site (intracranial, intraspinal, intraocular, pericardial, intraarticular, intramuscular with compartment syndrome, or retroperitoneal), or fatal bleeding).

g. Secondary infection / superinfection:

1. Intercurrent, at least probable, documented serious disease caused by an infection other than SARS-CoV2, requiring antimicrobial administration and care.
2. A complete list of secondary infections / superinfections is provided in the study manual of procedures.

3.1.5 NATIONAL EARLY WARNING SCORE (NEWS)

Vital signs and other clinical assessments are collected for the calculation of the NEWS, and include temperature, systolic blood pressure, heart rate, respiratory rate, O_2 saturation and level of consciousness. Vital signs collected per standard of care can be used. NEWS has demonstrated an ability to discriminate subjects at risk of poor outcomes. (Smith, Prytherch et al. 2016). This score is based on 7 clinical parameters (see **Table 2**). The NEWS is being used as an exploratory efficacy measure. The components of NEWS should be evaluated daily while hospitalized. It can be performed concurrently with the Ordinal Scale. This should be evaluated at a consistent time for each study day and prior to administration of study product. The 7 parameters can be obtained from the hospital chart or electronic medical record (EMR) using the last measurement prior to the time of assessment (including parameters collected prior to the time of consent) and a numeric score is given for each parameter (e.g., a respiratory rate [RR] of 9 breaths per minute is one point, oxygen saturation of 92% is two points). This is recorded for the day obtained (i.e., on Day 3, the vital signs and other parameters from Day 3 will be

used to obtain NEWS for Day 3). ECMO and mechanically ventilated participants should be assigned a score of 3 for RR (RR ≤8 breaths per minute) regardless of the ventilator setting. Participants on ECMO should get a score of 3 for heart rate since they are on cardiopulmonary bypass.

Table 2. National Early Warning Score (NEWS)

PHYSIOLOGICAL PARAMETERS	3	2	1	0	1	2	3
Respiration Rate (breaths per minute)	≤8 [a]		9 – 11	12 – 20		21 – 24	≥25
Oxygen Saturation (%)	≤91	92 – 93	94 – 95	≥96			
Any Supplemental Oxygen		Yes		No			
Temperature (°C)	≤35.0		35.1 – 36.0	36.1 – 38.0	38.1 – 39.0	≥39.1	
Systolic Blood Pressure (mm Hg)	≤90	91 – 100	101 – 110	111 – 219			≥220
Heart Rate (beats per minute)	≤40 [b]		41 – 50	51 – 90	91 – 110	111 – 130	≥131
Level of Consciousness				A			V, P, U

Level of consciousness = alert (A), and non-alert and arousable only to voice (V) or pain (P), and unresponsive (U).

[a] *If the participant is on ECMO or invasive mechanical ventilation, they will be given a score of 3 (≤8 breaths per minute) for respiratory rate regardless of ventilator setting.*

[b] *Participants on ECMO will also receive a score of 3 (≤40 beats per minute) for heart rate.*

3.2 POWER AND SAMPLE SIZE

ACTIV-1 is an event-driven trial based on the number of observed recovery events. The effectiveness of each therapeutic agent as add-on therapy to SoC will be evaluated based on the primary endpoint of time to recovery through Day 28. The Fine-Gray approach will be used to compare each test agent + SoC vs. SoC-alone with respect to the cumulative incidence of recovery, accounting for the semi-competing risk of mortality (Fine and Gray 1999). The approach is similar to using a log-rank test on time to recovery, retaining in the risk set people who die. The two key determinants of power are the total number of events (i.e., recoveries) and the treatment-to-control recovery rate ratio (RRR).

Overall study sample size requirements are based on the number of agents being evaluated and the ability to pool control patients for analysis. Initial sample size estimates are derived assuming three agents are ready for testing at study start and will remain in the study for evaluation at the final analysis stage. If newly emergent therapies are entered into the master protocol after study start, sample size requirements will be adjusted accordingly.

Table 3 provides the numbers of recoveries and of patients required to provide 85% power for a single pairwise comparison of test drug versus control assuming a 73% recovery rate and various RRRs. The assumed 73% recovery rate is based on the Kaplan-Meier recovery rate from ACTT-1. Note that the recovery rate is the analogue of the hazard for each test agent or control treatment, and the RRR is the analogue of the hazard ratio for a test agent relative to control in this setting. Approximately 347 recoveries are required to detect a 40% increase in the rate of recovery ($\theta = 1.40$) from control. An RRR of 1.40 is similar to, but slightly higher than the figure of 1.31 reported in Cao et al. (2020) for a lopinavir/ritonavir trial that used time to improvement by 2 categories as primary endpoint (Cao, Wang et al. 2020). A total of 436 recoveries is needed for an RRR of 1.35 with 85% power.

Table 3. Number of recoveries and number of patients needed for 85% power assuming a type I error rate of 5% for various recovery ratios

Recovery rate ratio (θ)	Number of recoveries needed for 85% power	Sample Size assuming 73% recovery rate (1 test agent vs SoC)
1.25	**788**	**1,080**
1.30	570	781
1.35	436	598
1.40	347	476

The sample size requirements are based on the ability to detect a moderate improvement in time to recovery for each agent. After accounting for the proposed interim efficacy and futility monitoring rules,

a total of 788 recoveries are required for each comparison to provide approximately 85% power to detect a RRR of 1.25. Assuming 73% of participants achieve recovery in 28 days, consistent with the ACTT-1 results (Beigel, Tomashek et al. 2020), the total sample size to evaluate 1, 2, and 3 agents in ACTIV-1 IM is approximately 1080, 1620, and 2160, respectively.

The proposed power and sample size requirements also provide adequate power to detect differences in the key secondary endpoints of clinical improvement and mortality. Based on the formulas from Whitehead (1993), a sample size of 1,080 (540 participants / group)) gives approximately 95% or 85% power to detect a common odds ratio of 1.50 of clinical improvement (the observed odds ratio in the ACTT-1 trial) or 1.40 using a 2-tailed test at level α=0.05. The 28-day mortality probability in the remdesivir arm of ACTT-1 was approximately 12%. The power for comparing 28-day mortality is 90% for a 50% relative reduction (from 12% to 6%) and 80% to detect a difference of 12.0% vs. 6.8%.

3.3 RANDOMIZATION SCHEME

Randomization will be stratified by:

- Geographic Region
    - USA – Northeast
        - Connecticut, Maine, Massachusetts, New Hampshire, New Jersey, New York, Pennsylvania, Rhode Island, Vermont
    - USA - Midwest
        - Illinois, Indiana, Iowa, Kansas, Michigan, Minnesota, Missouri, North Dakota, Nebraska, Ohio, South Dakota, Wisconsin
    - USA – South
        - Alabama, Arkansas, Delaware, District of Columbia, Florida, Georgia, Kentucky, Louisiana, Maryland, Mississippi, North Carolina, Oklahoma, South Carolina, Tennessee, Texas, Virginia, West Virginia
    - USA – West
        - Alaska, Arizona, California, Colorado, Hawaii, Idaho, Montana, New Mexico, Nevada, Oregon, Utah, Wyoming, Washington
    - Argentina
    - Brazil
    - Peru
    - Mexico
    - Additional countries will be treated as separate regions
- Severity of illness at enrollment (by ordinal scale)
    - Severe disease:

    - Hospitalized, on invasive mechanical ventilation or ECMO, or
    - Hospitalized, on non-invasive ventilation or high flow oxygen devices.
  - Moderate disease:
    - Hospitalized, requiring supplemental oxygen, or
    - Hospitalized, not requiring supplemental oxygen.

Conceptually, randomization will proceed in two steps. At the first step, each participant will be assigned (open-label) with equal probability to one of the agents available at the time the patient is enrolled and for which the patient is eligible to receive, after applying any agent-specific safety exclusions. At the second step, each participant will be assigned to either the test agent or its matching placebo in an n:1 ratio, where n = the number of agents currently active in the master protocol and for which the patient is eligible to receive.

With SoC and three agents active simultaneously, if the participant meets each of the agent-specific criteria, this procedure results in the randomization ratio of 1:1:1:1. If 3 agents are available, but a patient is only eligible to receive 2 of them, the allocation ratio at the first step will be 1:1, and at the second step will be 2:1 (agent vs placebo). Inclusion of a matching placebo for each agent enables masking of study participants and clinical personnel to treatment assignment at the second stage. Data from patients receiving SoC plus placebo will be pooled across agents for comparative analyses and hypothesis testing. Comparative analyses of a particular agent will include the subset of pooled placebo patients who enrolled concurrently with the particular agent and would have been eligible to receive the agent. That is, patients enrolled to control arm prior to a new agent entering the trial will not be included in comparative analyses of that newly added investigation agent.

3.4 INTERIM ANALYSES AND DATA MONITORING

A complete description of the interim analyses plans including mock tables, listings, and figures is included in a separate DSMB guidance document. Briefly, unblinded interim analyses are planned to (i) assess the futility of each agent, with the goal of discontinuing those with lower probabilities of success to more effectively utilize trial resources for the remaining agents and (ii) review comparative analyses for each test agent to assess early stopping for efficacy.

Alpha spending functions will be used to appropriately control the probability of making an erroneous conclusion across interim and final efficacy analyses at $\alpha = 0.05$ (two-sided) for each agent. The familywise error rates will be controlled at the two-sided 0.05 level for the primary endpoint and the two key secondary endpoints. Briefly, a gatekeeper approach will be used to test the primary hypothesis. As described in Hung, Wang and O'Neill (2007), the null hypotheses for the two key secondary endpoints are tested only if the null hypothesis is rejected for the primary endpoint. At the second level, the two key secondary endpoints are evaluated using a Pocock-type boundary with a Holm

procedure used to control the type I error for the two secondary endpoints (Tamhane, Mehta, Liu 2010; Holm 1979).

3.4.1 INTERIM SAFETY ANALYSES

Safety analyses will evaluate Grade 3 and 4 AE and SAEs by treatment arm. Safety monitoring will be ongoing. The unblinded statistical team will prepare these reports for review by the DSMB. More details will be provided in a separate DSMB guidance document.

3.4.2 INTERIM ANALYSES FOR FUTILITY AND EFFICACY REVIEW

Interim analyses for futility and efficacy were planned at three times during the study corresponding to the availability of approximately 25%, 50%, and 75% of total information. The planned randomization algorithm ensures approximately equal allocation to each test agent or control assuming that participants are eligible for all available agents. It is anticipated that the interim analyses for each agent will occur at the same time, and the DSMB will make recommendations for all agents at their scheduled meetings. If recovery rates vary substantially by agent; however, it may be necessary to let the interim analysis times also vary by agent. Because Type I error probabilities are controlled for each agent, independently of the other agents, the need for additional DSMB reviews due to differential information accrual across agents should not pose any issues other than logistical ones.

The Lan-DeMets spending function analog with the O'Brien-Fleming boundaries will be used to monitor the primary endpoint as a guide for the DSMB. This spending function is conservative in that priority is given to preserving power for the final analysis with the use of stringent stopping rules early in the study.

In contrast, moderately aggressive stopping rules for futility will be implemented to promote early discontinuation of agents with low probabilities for success. The futility stopping rules will be considered non-binding by the DSMB in their review of interim data. The futility boundaries are computed based on the gamma family of spending functions (Hwang, Shih, DeCani 1990). **Figure 1** below illustrates the efficacy boundaries based on the Lan-DeMets spending function analog to O'Brien-Fleming and the futility boundaries based on a (Gamma=-2) spending function.

Figure 1: Stopping Boundaries for Efficacy (blue shading) and Futility (pink shading).

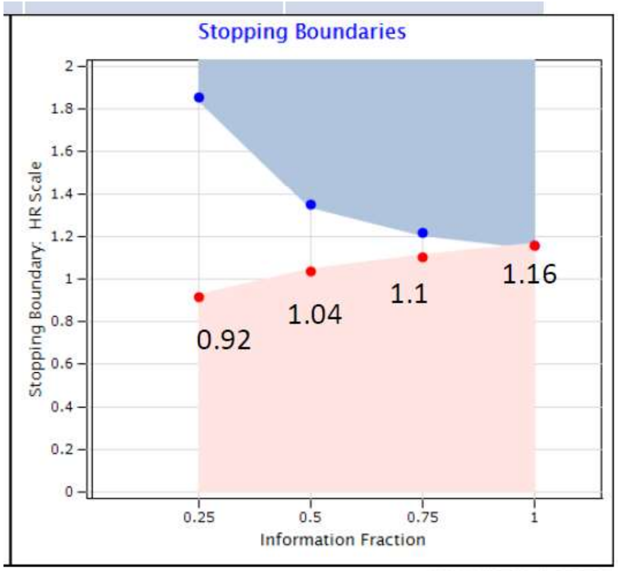

As can be seen from the figure, an RRR showing no more than a small amount of improvement in recovery time (RRR ≤ 1.04) will result in early discontinuation of the agent mid-way through information accrual in the study (**Table 4**). When the null hypothesis is true (RRR = 1), the probability of discontinuing an agent at this point is 66%, but under the alternative, this probability is 4%.

Table 4. Efficacy and futility boundaries expressed in terms of z-scores and recovery rate ratios (RRRs). Conditional power corresponding to futility boundaries is also shown, and is computed under the originally hypothesized RRR of 1.25. For the interim analyses, conditional power will be calculated under additional scenarios including the current trend and the null hypothesis.

	Efficacy Boundary		Futility Boundary		
Information Fraction	Z-score	RRR	Z-score	RRR	Conditional Power
0.25	4.3326	1.8540	-0.5854	0.9200	0.52
0.50	2.9631	1.3479	0.3791	1.0389	0.40
0.75	2.3590	1.2142	1.2187	1.1055	0.36
1.00	2.0141	1.1543	2.0141	1.1543	NA

If the primary endpoint is declared to be statistically significant in the favorable direction, the two key secondary endpoints will be tested according to a Pocock-like boundary with the Holm procedure used to control for the two key secondary endpoint comparisons (Holm 1979). See **Table 5** for the details of these procedures.

Table 5. Efficacy boundaries for the two key secondary endpoints assuming 3 equally spaced interim reviews and a final analysis.

Information Fraction	If the primary endpoint is declared significant, the larger absolute Z-statistic (smaller nominal p-value) is compared against …<br>|Z| (one-sided p-value)	If the primary and other key secondary endpoint is declared statistically significant, the smaller absolute Z-statistic (larger p-value) is compared against …<br>|Z| (one-sided p-value)
0.25	2.62 (0.0043)*	2.36 (0.0091)
0.50	2.62 (0.0043)	2.36 (0.0091)
0.75	2.62 (0.0043)	2.36 (0.0091)
1.00	2.62 (0.0043)	2.36 (0.0091)

*Exact boundaries will be computed using a spending function approach with a Pocock-like bound.

Please note that the DSMB meeting scheduled for the 0.25 information time occurred closer to 0.30 information fraction. Furthermore, the DSMB approval letter dated May 28, 2021 states the following …

"The NIAID DCR Data and Safety Monitoring Board held a teleconference on May 6, 2021. After carefully reviewing the safety data and interim analyses, the DSMB identified no safety concerns and recommended that all sub-studies in ACTIV-1 continue as planned. The DSMB made other recommendations as noted in the summary and recommended that the next DSMB meeting should take placed when ACTIV-1 has reached 60% enrollment."

Following the September 2, 2021 DSMB meeting, the recommendations stated that "the Board recommends that no further interim analysis be done". This meeting was the second DSMB review of the unblinded study data. 1340 participants had been randomized across 70 sites in the U.S. and Latin America at the time of the data cut.

3.4.3 ADAPTATIONS

The master protocol design with common data elements, procedures, etc., and adaptive platform design allows for seamless dropping or replacing of an arm by a new treatment. Only concurrent data will be used for comparisons. Thus, a new arm will be compared to controls enrolled on or after the time the new arm is added and determined to be eligible to receive the agent. It is anticipated that the DSMB will informally incorporate mortality and adverse event information into the decision making regarding stopping for efficacy and futility.

Prior to the second interim analysis, there is a plan to have a blinded sample size re-estimation that allows for sample size increases of as much as 25% but no reduction. This decision would be made by the members of the study team without access to the treatment codes (i.e. active or placebo) or the unblinded results. The formula of Whitehead (1993) was used to determine the expected power for the 8-point scale. That calculation includes a factor that depends on the marginal distribution of the 8-point scale lumped over the active and placebo arms. Additionally, the recommendation for the sample size adjustment will examine the overall recovery rate through Day 28. It is possible that the observed

recovery rates will be somewhat different from the ACTT-1 results due to changes in SoC, differences in participant mix, and random variability. Recommendations for the sample size adjustment will be based on trying to maintain ≥ 85% power for the primary endpoint and ≥ 70% power for the key secondary endpoints.

Approximately 2 weeks prior to the data cut for the second interim analysis, the blinded statistical team will conduct a review of marginal event rates and endpoint distributions. The goals of the blinded review are to assure that there is adequate power for the primary and two key secondary endpoints under various extreme scenarios. Namely, the team would like to maintain 85% power for the primary endpoint and >70% power for the two key secondary endpoints. The blinded statistics team will have access to limited data including the marginal 28-day mortality rate and the 14-day 8-point ordinal scale lumped across the three sub-studies. As noted in **Table 6** below, the power for these endpoints does depend on these marginal distributions.

Table 6. Outline of the Power Calculations for the Primary and Two Key Secondary Endpoints

**Endpoint**	**Assumptions under the Null Hypothesis** (H_0)	**Assumptions under the Alternative Hypothesis** (H_a)	**Notes**
Time-to-recovery	RRR = 1.0	RRR = 1.25	Each sub-study plans to observe >788 recovery events for the primary endpoint, which provides 85% power under the alternative hypothesis.
8-Point Ordinal Score at 14-days	Common Odds Ratio = 1.00	Common Odds Ratio = 1.50	The power for this statistical comparison is highly dependent on the marginal distribution of the 8-ordered categories. For example, if the placebo group distribution includes 12.5% for each category, a common odds ratio of 1.50 with sample sizes of 540/group will yield approximately 97% power with two-sided Type I error of 0.05. By comparison, a placebo distribution with 10% in category 1, 84% in category 8, and 1% each in categories 2 to 7, would yield only 63% power under the same conditions.
Mortality at 28 days (binary endpoint)	Odds Ratio = 1.00	Odds Ratio < 1.00 (e.g. 50% relative reduction)	12% (placebo) vs. 6% (active) with 540 participants per group will provide 88% power with Type 1 error (two-sided) of 0.025. By comparison, 6% (placebo) vs. 3% (active) will yield the only 55% power under the same conditions.

Tables 7 and 8 provide an example of the power calculations that will be conducted to determine whether there is adequate power to detect 50% reductions in 28-day mortality rates. In the example below there would be adequate power (i.e. >70% power) to detect large relative differences. With a much lower pooled mortality rate, the power would decrease.

Table 7. Four scenarios for the treatment mortality rates with marginal mortality rates of 10%.

	**Scenario 1**	**Scenario 2**	**Scenario 3**	**Scenario 4**
	**No effective treatments**	**1 effective treatment**	**2 effective treatments**	**3 effective treatments**
Placebo	10%	11.43%	13.33%	16.00%
Active Drug 1	10%	5.71%	6.67%	8.00%
Active Drug 2	10%	11.43%	6.67%	8.00%
Active Drug 3	10%	11.43%	13.33%	8.00%
Total	10.00%	10.00%	10.00%	10.00%

Table 8. Power calculations to detect 50% relative reductions for the 28-day mortality endpoint.

**Placebo Group Rate**	**Active Group Rate**	**Power with Type I Error of 0.05 (two-sided)**	**Power with Type I Error of 0.025 (two-sided)**
11.43%	5.71%	92%	86%
13.33%	6.67%	95%	92%
16.00%	8.00%	98%	96%

Assuming approximately 540 participants per group

A similar approach will be used with the marginal distribution on the 8-point scale. If these plausible scenarios provide greater than 70% power to detect a common odds ratio of 1.50, the sample size will not be increased.

3.5 DATA SOURCES

At each study site, data will be entered on the electronic Case Report Forms (eCRFs) stored in Medidata Rave. Prior to database lock, programmed computer edit checks will be run against the database to identify discrepancies and verify reasonableness of the data. Queries to resolve discrepancies will be generated and resolved by the sites. As needed, DCRI Statistics will be able to directly download the eCRF database from Rave using SAS on Demand as Clinical Data Interchange Standards Consortium Study Data Tabulation Model (CDISC SDTM version 3.2) datasets. CDISC Analysis Data Model (ADaM version 2.1) datasets will be created by DCRI Statistics for production of final tables, figures, and listings.

All planned reporting will be based off of CDISC datasets, but in the case of emergent safety data, some reporting may occur from the raw eCRF data. All programs written to create analysis datasets and perform analyses will be validated according to SOPs established by the DCRI Statistical Programming group.

3.6 DOCUMENTATION CONVENTION

The statistical analyses described in this SAP, as well as production of tables, listings, and figures will be performed using SAS®, version 9.4 or higher (SAS Institute, Cary, NC). Additional statistical software may be used as needed.

3.7 VERIFICATION OF RESULTS

All tables, listings, and graphs will be verified and reviewed before considered final. The verification process will ensure that the numbers are produced by a statistically valid method and that the execution of the computations is correct. Qualified statisticians or statistical programmers employed by the DCRI who have not been previously involved in the production of the original programming will perform the verification procedures. Methods of verification include independent programming, prior to issuance of the draft statistical report, of all analysis datasets/ADaM and comparison to data listings. Tables, listings, and figures will be reviewed for accuracy, consistency with this analysis plan, consistency within tables/listings/figures, and consistency with corresponding output. Once verification is complete, all documentation of the verification process will be filed as required by the Statistical Standard Operations Procedures of the DCRI.

3.8 SUBJECT DISPOSITION

The disposition of subjects (number randomized, number who received any amount of the randomly assigned treatment, number completing study drug administration, number who withdrew consent or discontinued from study drug early, and number lost to follow-up, and number who completed the trial) will be summarized by treatment group. The number of subjects screened for inclusion and a breakdown of reasons for exclusion will be summarized. The timing and reasons for early discontinuation of study drug and/or withdrawal from the study will be summarized by treatment group. For the calculation of percentages, subjects who die will not be included in the denominators for visits/assessments beyond their death. Treatment compliance (e.g. number of subjects who had their required infusions halted/slowed and the number of subjects with missed doses) will be summarized by

treatment group. A subject listing of analysis population eligibilities will be generated. A listing of all subjects discontinued from the study after enrollment, broken down by site and treatment group will be provided. The listing will include: reason for discontinuation, treatment group, duration of treatment, and whether or not the blind was broken. Also, for subjects who discontinued from the study after enrollment, a listing of adverse events will be provided. Subject-specific protocol deviations will be summarized by the reason for the deviation, the deviation category, treatment group, disease severity and (separately) site for all subjects. All subject-specific protocol deviations and non-subject specific protocol deviations will be included in listings.

3.9 POPULATIONS FOR ANALYSES

The primary analysis will be based on an intent-to-treat (ITT) population, including all participants randomized. Similarly, safety analyses will be based on a modified intention-to-treat (MITT) population consisting of all participants who received at least one dose or injection of each drug administered in the randomization arm (e.g. investigational agent/placebo plus SoC). Each substudy will define separate ITT and MITT populations due to the shared SoC plus placebo (or control) group. The primary analysis will be based on those participants randomized in order to achieve 788 recoveries for each pairwise comparison. Subsequent analysis will be performed on all randomized participants.

4.0 STATISTICAL ANALYSES

4.1 GENERAL APPROACH

- Statistical significance: Statistical comparisons will be performed using two-sided significance tests. An alpha level of 0.05 will determine significance (unless otherwise noted) for each agent. Analyses will be carried out separately for each test agent.

- Descriptive statistics:
  - Continuous variables will be presented as n, mean, standard deviation, median, Q1, Q3, and minimum and maximum. For comparisons of treatment groups, we will use the non-parametric Wilcoxon-Mann-Whitney rank-sum test.
  - Categorical variables will be presented as percentage (number). Group comparisons will use the conventional chi-square test.
  - Binary variables will be presented as percentage (number). Group comparisons will use the conventional chi-square test or Fisher's Exact Test.

- When death is not a competing risk (for example, the endpoint includes death in the composite), differences in time-to-event endpoints by treatment will be summarized with Kaplan-Meier curves, log-rank tests, stratified log-rank tests, and hazard ratios from Cox proportional hazards models. When death is a competing risk (for example, time to at least a one-category improvement in ordinal scale, and time to at least a two-category improvement), the same competing risk approach will be used as for the primary analysis and results will be summarized by the hazard ratio from the Fine-Gray model.

- Covariate adjustment clarification – for some of the models described below, it might be necessary to combine regions and / or disease severity levels to meet modeling assumptions.

- Unblinding for the final analysis of a study agent:

  Unblinding of study data for final analysis will occur for each test agent independently of other agents, consistent with the master protocol design. That is, once the planned number of recoveries required for a particular agent's comparative analysis are observed, study close-out

procedures for that agent are applied for all study visits and data elements associated with the participants receiving the test agent as well as participants receiving SoC plus placebo during the randomization period for that agent (i.e., concurrently controlled participants) subset to those eligible to receive the agent.

Once the study visits are monitored, data edits are completed, queries are resolved, the database is locked, and treatment assignments are unmasked for that comparative analysis only. Note that, because placebo control participant data are shared across agents, procedures for unmasking data for one agent's final analysis will be established to protect the ability to continue the ongoing study in a double-blinded manner for the remaining agents. It may be that recovery rates are similar enough across all agents in the study to enable a single study close-out with standard procedures. Any necessary modifications or updates to the statistical analysis should be made prior to the study unblinding.

- Handling of Missing Data:

Efforts to minimize loss-to-follow-up will be considerable. However, small amounts of missing data may occur. The plans for handling missing data follow the approach taken in the ACTT-1 study (Beigel, Tomashek et al. 2020). All attempts will be made to collect all data per protocol. Any data point that appears to be erroneous or inexplicable based on clinical judgment will be investigated as a possible outlier. If data points are identified as outliers, sensitivity analyses may be performed to examine the impact of including or excluding the outliers. Any substantive differences in these analyses will be reported.

For time to event outcomes, participants who are lost to follow-up or terminate the study prior to Day 28 and prior to observing/experiencing the event will be censored at the time of their last observed assessment.

For the analyses of the secondary outcomes described in Section 4.3, the following imputation rules will be used for participants who are lost to follow-up, terminate early from the study, or do not have further outcome data available after discharge for any reason:

- Days of Non-invasive ventilation/high-flow oxygen:
  - If the subject's clinical status scale is 3 or 2 at the last observed assessment, then the subject will be considered to be on non-invasive ventilation/high-flow oxygen through

Day 28. The endpoint will be total days when assessments are available plus all imputed days following the last observed assessment.

- If the subject is not on non-invasive ventilation/high-flow oxygen at the last observed assessment, then the subject will be considered to not be on non- invasive ventilation/high-flow oxygen for the remainder of follow-up. Thus, no additional imputed days will be added to the number of days recorded on available assessments.

- Days of ventilation/ECMO:
  - If the subject's clinical status scale is 2 at the last observed assessment, then the subject will be considered to be on ventilation/ECMO through Day 28. The endpoint will be total days when assessments are available plus all imputed days following the last observed assessment.
  - If the subject is not on ventilation/ECMO at the last observed assessment, then the subject will be considered to not be on ventilation/ECMO through Day 28. Thus, no additional imputed days will be added to the number of days recorded on available assessments.

- Days of Oxygen:
  - If the subject's clinical status score is 4, 3, or 2 at the last observed assessment, then the subject will be considered to be on oxygen through Day 28. The endpoint will be total days when assessments are available plus all imputed days following the last observed assessment.
  - If the subject is not on oxygen at the last observed assessment, then the subject will be considered to not be on oxygen through Day 28. Thus, no additional imputed days will be added to the number of days recorded on available assessments.

- Days of Hospitalization
  - If the subject is discharged and no further hospitalization data are available, then the subject will be assumed to not have been readmitted. Thus, no additional imputed days will be added to the number of days recorded on available assessments.

- NEWS - There will be no imputation for incomplete components of the NEWS score.

4.2 ANALYSIS OF THE PRIMARY AND KEY SECONDARY EFFICACY ENDPOINTS

- **Primary Endpoint – Time to Recovery through Day 28**

The primary efficacy analysis for the comparison of each test agent plus SoC versus SoC plus placebo is a stratified test based on the Fine-Gray proportional hazards approach, where stratification is according to geographic region and baseline disease severity on the 8-point ordinal scale (i.e. 2 or 3 or 4 or 5). Other covariates in the model will include age, and sex. The approach is similar to using a stratified log-rank test on time to recovery, providing an estimate of the cumulative incidence of recovery while accounting for the competing risk of death. The primary quantity of interest is the RRR. With no censoring, the hazard rate in each arm can be thought of as the hazard for recovery, treating deaths as never having recovered (Fine and Gray 1999, Gooley, Leisenring et al. 1999). The median time to event and 95% confidence interval will be summarized by treatment arm. Cumulative incidence curves for each treatment arm will be presented, supplemented with the RRR estimate, p-value, along with the number of subjects at risk in each arm at Days 0 (baseline), 2, 4, 7, 10, 14, 21, and 28.

- Sensitivity Analyses

Gray's test on time to recovery will be calculated to obtain the p-value with fewer parametric assumptions (Gray 1988).

As a sensitivity analysis, the main analysis of the primary and two key secondary endpoints (described below) will be repeated in the MITT population.

- Supplemental Analyses

A supplemental analysis will examine the Day 28 binary response of 'recovered' or 'not recovered' which aligns with the 8-point ordinal score at Day 28. Approximately, 95% of study participants are expected to have complete information on this endpoint.

For individuals with a recovery event but missing 8-point ordinal score at Day 28 (~3% of the study population), a multiple imputation approach will be applied to impute those missing values. For each missing observation, 10 imputed observations will be randomly drawn from the subset of individuals with complete data who had a recovery event.

For individuals without a recovery event but missing 8-point ordinal score at Day 28 (~2% of the study population), a multiple imputation approach will be applied to impute those missing values. For each missing observation, 10 imputed observations will be randomly drawn from the subset of individuals with complete data and the same baseline 8-point ordinal score (i.e. 2 or 3 or 4 or 5). Results from the 10 imputed datasets will be combined using Rubin's rule to obtain the point estimate, 95% confidence interval, and p-value.

Those individuals in categories 6-8 would be considered as recovered and those in categories 1-5 are 'not recovered'. The point estimate will be based on a difference in the probability estimates for active vs. control and the 95% CI will be constructed (and p-value obtained) using the Wald method.

Additionally, for each participant, a count of the number of days recovered and out-of-hospital will be computed. The treatment effect will be summarized by the mean difference between groups. This outcome will be analyzed using a linear model with adjustment for geographic region and baseline disease severity on the 8-point ordinal scale (i.e. 2 or 3 or 4 or 5) and an indicator for active vs. placebo treatment. An additional supplemental analysis will analyze this outcome variable using a Wilcoxon-Mann-Whitney test.

Analyses that take into account concomitant medication will be performed. The primary analysis will be repeated, where subjects who take prohibited medications will be treated as treatment failures and will be censored at the time of medication use. Other censoring techniques and additional analyses may be performed.

- **Key Secondary Endpoint #1 - Clinical status (8-point ordinal scale) at Day 14**

The ordinal scale will be used to estimate a proportional odds model by disease strata and region. We will perform a covariate adjusted test to evaluate whether the common odds ratio for treatment is equal to one. The model covariates will include baseline severity (2, 3, 4, or 5), region, sex, and age (continuous). The distribution of severity results will be summarized by treatment group as percentages. The treatment odds ratio, 95% CI, and p-value estimated from the model will be presented.

Supplemental analyses will examine the clinical status using the 8-point ordinal scale at Day 28. This endpoint is expected to be bimodal with individuals more likely to be at the extremes of the scale compared to the Day 14 version.

Analyses of the Day 14 and Day 28 clinical status score will be conducted using multiple imputation. The ~2% of participants who recovered prior to Day 14 but have missing data for the 8-point ordinal scale collected at Day 14 will have 10 observations imputed using randomly selected data from other participants who had complete data for their clinical status (8-point ordinal scale) at Day 14 and a recovery event prior to Day 14.

Participants without a recovery and with missing data for the 8-point ordinal scale at Day 14 (~2% of participants) will have 10 imputed values randomly drawn from the subset of individuals with complete data and the same baseline 8-point ordinal scale. A similar approach will be taken for the Day 28 clinical status score. Results from the 10 imputed datasets will be combined using Rubin's rule.

The number and proportion of subjects along with 95% confidence intervals by category of clinical status will be presented by treatment arm at Days 0 (baseline), 2, 4, 7, 10, 14, 21, and 28. A figure will present stacked bar charts by day with side by side bars for each treatment arm. Histograms will be generated to display the ordinal scale value distributions over time in each treatment group.

- ### Key Secondary Endpoint #2 - Mortality through Day 28

Mortality through Day 28 will be analyzed as a binary endpoint, using a logistic regression model with an indicator variable for the treatment group. Other variables in the regression model will include the 8-point ordinal scale at baseline (2, 3, 4, or 5), region, sex, and age (continuous). The odds ratio from this model will be supplemented with event rates at 14 and 28-days along with a difference between treatment groups and 95% confidence intervals (and p-values) obtained using the Wald method. The number and percentage of subjects who die within 14- and 28-days following randomization will be presented by treatment arm (denominator for the percentages will be the number of subjects in the ITT population in each treatment arm).

Approximately, 95% of study participants are expected to have complete information on this endpoint.

For individuals with a recovery event but missing mortality status at Day 28 (~3% of the study population), a multiple imputation approach will be applied to impute those missing values. For each missing observation, 10 imputed observations will be randomly drawn from the subset of individuals with complete data who had a recovery event.

For individuals without a recovery event but missing mortality status at Day 28 (~2% of the study population), a multiple imputation approach will be applied to impute those missing values. For each missing observation, 10 imputed observations will be randomly drawn from the subset of individuals with complete data and the same 8-point ordinal scale baseline score.

Results from the 10 imputed datasets will be combined using Rubin's rule to obtain the point estimate, 95% confidence interval, and p-value.

- ### Tipping Point Sensitivity Analyses for the Primary and Key Secondary Endpoints

Tipping point sensitivity analyses that systematically and comprehensively vary assumptions about the missing outcomes on the two treatment arms will be applied. These analyses will be two-dimensional and will allow assumptions about the missing outcomes on the two arms to vary independently. These scenarios will include those where dropouts on active drug tend to have worse outcomes than dropouts on placebo. The goal is to explore the plausibility of missing data assumptions under which the conclusions change, i.e., under which there is no longer evidence of efficacy.

Among randomized participants, approximately 5% or participants have missing data for the primary or the two key secondary endpoints (**Table 9**). There are two distinct types of missing data for these endpoints:

1) participants with almost no information (n~42) and

2) those with missing data following a recovery event (n~60).

Among the 1308 randomized participants with a recovery event prior to Day 14, the vast majority (~98%) had complete data for the ordinal score at Day 14 and approximately 99% of those individuals had a score of 7 or 8. Similarly, among the 1527 randomized participants with a recovery event prior to Day 28, only 8 (<1%) died prior to Day 28.

Table 9. Summary of Missing Data by Type for the Primary and Two Key Secondary Endpoints (data as of March 28, 2022)

Endpoint	Total Participants with Missing Data (N=1971)	Post-Recovery Missing Data (N=1971)	Missing Data Prior to a Recovery (N=1971)
Primary Endpoint – Time to Recovery through Day 28	42* (2.1%)	n/a	42 (2.1%)
Key Secondary Endpoint #1 - Clinical status (8-point ordinal scale) at Day 14	77 (3.9%)	35 (1.8%)	42 (2.1%)
Key Secondary Endpoint #2 - Mortality through Day 28	102 (5.2%)	60** (3.0%)	42 (2.1%)

* 5 (11.4%) participants had > 3 days of follow-up and 11 (25%) received any of their randomized study drug
**of the 1527 participants who recovered prior to Day 28, 8 (<1%) were determined to have died prior by Day 28.

To create an imputation strategy that maintains consistency between the primary and two key secondary endpoints, we will first impute the 'mortality status at day 28' endpoint, then the '8-point ordinal scale at 14 days', and finally, the 'time-to-recovery through day 28' primary endpoint. This structure will help us avoid illogical scenarios such as having the same participant with the following imputed values …

- Death at Day 6
- Recovery at Day 10
- Day 14 8-point ordinal scale of 2 (hospitalized, on invasive mechanical ventilation or ECMO)

Clearly, a participant who dies on Day 6 cannot have a recovery on Day 10.

An ordinal logistic regression model will be fitted to the entire ACTIV1 cohort of randomized participants. The outcome variable will be the participant's status at Day 28 and will have three levels. The worst value of the outcome is death, the best value of the outcome is recovery prior to Day 28, and the intermediate value is for those participants who are alive without a recovery by Day 28. The logistic regression imputation model will include covariates for region and severity of illness ordinal score at the time of randomization. A parameter, ϴ, will vary from 0.1 to 10 and correspond to an odds ratio parameter for the ordinal logistic model. Values of ϴ less than 1.0 will be associated with better than average outcomes and values great than 1.0 will have worse than average outcomes. Specifically, for each level of the ordered response, the value of log(ϴ) will be added to the logit function with the covariates associated with the participants with missing data. Then a random uniform variable will be

generated and compared to the imputed probability distribution. With the imputed outcome of either 'death', 'recovery', or 'alive without recovery', the time-to-recovery and the Day 14 8-point ordinal scale response will be imputed by selecting a participant at random with the same severity of illness at randomization, region, and corresponding status at Day 28 (e.g. 'death', 'recovery', or 'alive without recovery').

On average, a value of ϴ = 0.1 will correspond to an expected Day 28 mortality of < 1% whereas a value of ϴ = 10 will correspond to an expected Day 28 mortality of ~ 49% (see **Table 10**). As special cases, values are ϴ = 0 and ϴ = ∞ will be used to denote the special cases of 'best possible outcome' and 'worst possible outcome', respectively. As an example, for a participant who withdrew on Day 3, the best possible outcome (i.e. ϴ = 0) would correspond to: Recovery at 3, Day 14 8-point ordinal scale of 8, and Alive at Day 28. Similarly, for a participant who withdrew on Day 3, the worst possible outcome (i.e. ϴ = ∞) would correspond to: Death at Day 3 and Day 14 8-point ordinal scale of 1.

Table 10. Probability, on average, of recovery and death by Day 28 for various parameter settings

Parameter Setting	Recovery through Day 28	Death through Day 28
ϴ = 0 "Best Case"	100%	0%
ϴ = 0.1	98%*	1%
ϴ = 0.2	95%	2%
ϴ = 0.5	90%	6%
ϴ = 1 "Missing at Random"	82%	10%
ϴ = 2	71%	18%
ϴ = 5	51%	34%
ϴ = 10	35%	49%
ϴ = ∞ "Worst Case"	0%	100%

*the exact values will depend on the participant's covariates

For each parameter setting, 10 imputed datasets will be constructed for each individual with missing data for the primary endpoint. For each imputed dataset, the primary and two key secondary analyses will be re-run and the results of the 10 models will be combined using Rubin's rules. The results of the tipping point analysis will allow for a comparison of point estimates and p-values across the various values of ϴ (see **Table 11** for an example).

Table 11 will be repeated for the two key secondary endpoints, and this analysis will be repeated for the comparison of each study drug vs. the corresponding placebo. Separate models will be constructed for participants with incomplete data following a recovery event (e.g. withdrawal of consent after discharge from the hospital).

Table 11. Example summary of the tipping point analysis for the time-to-recovery endpoint

RRR*<br>95% CI<br>p-value						Active				
		ϴ = 0	ϴ = 0.1	ϴ = 0.2	ϴ = 0.5	ϴ = 1.0	ϴ = 2	ϴ = 5	ϴ = 10	ϴ = ∞
	ϴ=0									
	ϴ=0.1									
	ϴ=0.2									
	ϴ=0.5									
Placebo	ϴ=1									
	ϴ=2									
	ϴ=5									
	ϴ=10									
	ϴ=∞									

*the values within each cell will correspond to the estimated RRR, 95% CI, and p-value for the particular parameter setting (e.g. $\theta_{Placebo}$=1, θ_{Active}=10).

The above description of the tipping point analysis has focused on the missing data of the subset of participants with no recovery. For the two key secondary endpoints, it is expected that 2-3% of participants will have some missing data following a recovery event.

For individuals with a recovery event but missing mortality status at Day 28 (~3% of the study population) or the Day 14 clinical status score, a multiple imputation approach will be applied to impute those missing values. For each missing observation, 10 imputed observations will be randomly drawn from the subset of individuals with complete data who had a recovery event, and the imputed datasets will be combined as described above.

4.3 ANALYSIS OF THE OTHER SECONDARY EFFICACY ENDPOINTS

- Time to improvement of one and two categories in ordinal scale

Two separate endpoints are considered here. The first endpoint will examine the time to a one category improvement in the 8-point ordinal scale. The second endpoint will examine the time to a two category improvement in the 8-point ordinal scale. These time-to-event endpoints will be computed through Day 28 will also be analyzed using the same Fine-Gray proportional hazards approach as for the primary efficacy endpoint and stratification using the randomization factors. Proportions of one and two category improvements at 14 and 28-days along with 95% confidence intervals overall for each treatment arm along with a difference between treatment arms and 95% confidence intervals obtained

using the Miettinen-Nurminen method. Calculations of these endpoints will rely on the 8-point ordinal scale that is collected on a daily basis while the participant is hospitalized.

- Mean change in ordinal scale at specific time points

Change in clinical status scale from baseline at specific time points will be summarized by proportions (e.g., proportion who have a 1-, 2-, 3-, or 4+ point improvement or 1-, 2-, 3-, 4-point worsening). A table will present the proportion of people on Days 2, 4, 7, 10, 14, 21, and 28 within each category of change by treatment arm along with 95% confidence intervals. The mean change in the ordinal scale difference between treatment arms along with 95% confidence interval will also be reported. Additionally, side-by-side histograms (of an agent versus control) of the proportions with various changes will be presented.

- Differences in total severity score (TSS) over time

Differences in total severity score (TSS) over time will assessed by fitting a longitudinal Markov model to a modified version of the 8-point clinical scale value on a daily basis between days 1 and 28. The average difference across days in study (analogous to the area under the severity curve) will be estimated with 95% confidence limits in the context of the longitudinal model. The longitudinal mixed model will be developed as follows. The value of the ordinal scale on day t will be assumed to depend on the patient's prior outcome trajectory only through the prior day's value, and treatment group assignment. The general form of the model will be:

$$\Pr\left(Y_{it} \leq k \middle| H_{i,t-1}, Y_{i.t-1} = x, Z_i = z, \boldsymbol{\alpha}, \boldsymbol{\beta}, \boldsymbol{\gamma}, e_i\right) \equiv \pi_{itk|xz},$$

$$\log \frac{\pi_{itk|xz}}{1 - \pi_{itk|xz}} = \alpha_{kx} + t\beta_1 + t^2\beta_2 + z(\gamma_0 + t\gamma_1 + t^2\gamma_2),$$

where Y_{it} is outcome of the i-th patient on day t, $H_{i,t-1} = (Y_{i1}, \ldots, Y_{i,t-1})$ is the patient's outcome trajectory through day $t - 1$, Z_i is a binary indicator of treatment group assignment (1=active agent plus SoC, 0=placebo plus SoC), and the symbols $\alpha_{kx}, \beta_1, \beta_2, \gamma_0, \gamma_1\gamma_2$ represent unknown fixed effect parameters to be estimated from the data. The model assumes that on each day, the odds of an outcome in one of the k lowest categories depends jointly on treatment assignment, days since randomization, and x is the patient's prior day outcome category. Parameters will be estimated by maximum likelihood using 1 record per patient per day from the first day post-randomization until the earlier of day 28, death or loss to follow-up. Days between death and day 28 do not contribute to the likelihood because death is an absorbing state and hence $\alpha_{11} = \infty$ and $\alpha_{21} = \alpha_{31} \cdots = \alpha_{81} = -\infty$. The estimation procedure will assume that loss to follow-up is conditionally ignorable given treatment assignment and the patient's current observed outcome trajectory. A feature of the trial's outcome assessment schedule is that only patients who are hospitalized have their outcome assessed on days

other than 7, 10, 14, 21, and 28. If a patient is not hospitalized, the patient's outcome category will be at least 7 but the exact value (7 versus 8) will only be collected on days 7, 10, 14, 21, and 28. To mitigate potential bias from informatively missing data, categories 7 and 8 will be combined into a single category for this analysis. This modification guarantees that a patient will have non-missing data on all days 1 through 28 if the patient's vital status and hospitalization status is known on each day and the patient's outcome is assessed per protocol on each day that the patient is alive and in-hospital.

- Days alive and free of supplemental oxygen

Days alive and free of supplemental oxygen and its components, duration of oxygenation days and total days alive, will be summarized in a table using mean (median) and standard deviations (quartiles) by treatment arm. Point estimates for the difference between active and placebo for days alive and free of supplemental oxygen and associated 95% confidence intervals will be obtained using a linear model with treatment group and baseline 8-point ordinal score (i.e. 2 or 3 or 4 or 5) as categorical predictors. The duration of oxygenation days will be computed using the 8-point ordinal scale that is collected each day the participant is hospitalized. If a participant is rehospitalized after an initial recovery and the 8-point ordinal scale is not available, the participant will be assumed to have an 8-point ordinal score value of 2 for all days of the rehospitalization.

The incidence of new oxygen use will be analyzed by treatment arm. This will only include subjects in ordinal scale category 5 at enrollment (i.e. 5: Hospitalized, not requiring supplemental oxygen – requiring ongoing medical in-patient care). New use will be identified by a post-enrollment score of 4 or 3 or 2. The number and percent of subjects reporting new use and 95% confidence interval will be reported. Comparisons between treatment arms will be presented as differences in proportions with 95% confidence intervals using the Miettinen-Nurminen method.

- Days alive and free of non-invasive ventilation/high flow oxygen

Days alive and free of non-invasive ventilation/high flow oxygen and its components, duration of non-invasive ventilation/high flow oxygen days will be summarized in a table using mean (median) and standard deviations (quartiles) by treatment arm. Point estimates and associated 95% confidence intervals will be obtained using a linear model with treatment group and baseline 8-point ordinal score (i.e. 2 or 3 or 4 or 5) as categorical predictors. The duration of non-invasive ventilation/high flow oxygen days will be computed using the 8-point ordinal scale that is collected each day the participant is hospitalized. If a participant is rehospitalized after an initial recovery and the 8-point ordinal scale is not available, the participant will be assumed to have an 8-point ordinal score value of 2 for all days of the rehospitalization.

The incidence of new Non-Invasive Ventilation/High Flow Oxygen use will be analyzed by treatment arm. This will only include subjects in category 4 or 5 at enrollment. New use will be identified by a post-

enrollment score of 3 or 2. Comparisons between treatment arms will be presented as differences in proportions with 95% confidence intervals using the Miettinen-Nurminen method.

- Days alive and free of invasive mechanical ventilation/ECMO

Days alive and free of invasive mechanical ventilation / ECMO and its components, total days alive and duration of invasive mechanical ventilation/ECMO days, will be summarized in a table using mean (median) and standard deviations (quartiles) by treatment arm. Point estimates for differences in the days alive and free of invasive mechanical ventilation / ECMO and associated 95% confidence intervals will be obtained using a linear model with treatment group and baseline 8-point ordinal score (i.e. 2 or 3 or 4 or 5) as categorical predictors. The duration of invasive mechanical ventilation/ECMO days will be computed using the 8-point ordinal scale that is collected each day the participant is hospitalized. If a participant is rehospitalized after an initial recovery and the 8-point ordinal scale is not available, the participant will be assumed to have an 8-point ordinal score value of 2 for all days of the rehospitalization.

The incidence of new Invasive Mechanical Ventilation/ECMO use will be analyzed by treatment arm. This will only include subjects in category 5, 4, or 3 at enrollment. New use will be identified by a post-enrollment score of 2. The number and percent of subjects reporting new use and the 95% confidence interval will be reported. Comparisons between treatment arms will be presented as differences in proportions with 95% confidence intervals using the Miettinen-Nurminen method.

- Days alive and out of hospitalization

Days alive and out of hospital along with its components, duration of hospitalizations and total days alive, will be summarized in a table using mean (median) and standard deviations (quartiles) by treatment arm. Point estimates for the difference between active and placebo agents for days alive and out of hospital and associated 95% confidence intervals will be obtained using a linear model with treatment group and baseline 8-point ordinal score (i.e. 2 or 3 or 4 or 5) as categorical predictors.

4.4 SAFETY ANALYSES

Safety endpoints include death through Day 28 (as assessed at Day 29), SAEs, and Grade 3 and 4 AEs. These events will be analyzed individually and as a composite endpoint. The time to this composite endpoint will be defined as the elapsed time (in days) from baseline to the earliest date of any of the events. Any subjects that are lost to follow-up or terminated early prior to experiencing any of the events will be censored at the day of their last observed assessment. Subjects who complete follow-up

but do not experience any of the events will be censored at the day of their Day 29 visit. Time-to-event methods will be used for death and the composite endpoint. A table will present the hazard ratio estimate with 95% confidence intervals and log rank p-values. Differences in time-to-event endpoints by treatment will be summarized with cumulative incidence curves. In addition, risk differences between treatment groups and 95% CIs using the Miettinen-Nurminen method will be estimated for each type of event.

Similar analyses will be conducted for the following AEs:

- Any Grade 2 suspected drug-related hypersensitivity reactions associated with study product administration will be reported as an AE.
- Any newly diagnosed infection (other than COVID-19) at any time during the study.
- Any AEs leading to dose modification
- Any AEs leading to discontinuation from the study.

AEs will be coded in accordance with the current version of the Medical Dictionary for Regulatory Activities (MedDRA). AEs will be graded with regard to severity according to criteria defined in the Common Terminology Criteria for Adverse Events version 5.0 (CTCAE) and relationship to COVID-19 or study intervention. Each AE will be counted once only for a given participant. AEs will be presented by system organ class, preferred term (PT), duration (in days), start- and stop-date. AEs leading to premature discontinuation from the study intervention and serious AEs will be presented in a listing.

4.4.1 LABORATORY DATA

Clinical safety laboratory adverse events are collected Day 1, 3, 5, 8, 11 and Day 15 and 29 if able to return to clinic or still hospitalized. Parameters evaluated include white blood cell count, differential, Hgb, PLT, creatinine, glucose, total bilirubin, AST, ALT, ALP, PT or INR, d-dimer, cardiac troponin, and C-reactive protein.

Descriptive statistics for baseline and post-baseline time points will be generated. Additionally, shift tables will be presented. The shift tables tabulate the number of lab values determined to be “Normal” and “Abnormal” at baseline and post-baseline time points. The classifications on shift tables will categorize lab values as “Normal” when they are in acceptable range. Laboratory values outside the normal range will be graded on the Labs Listing as >1x, >2x, >3x, >5x, or >10x the upper limit (or <0.1x, <0.5x, <1x the lower limit) of normal value based on appropriate increases or decreases. Laboratory data will also be summarized graphically to show the magnitude and changes in individual subject values over time relative to normal ranges. Details of any abnormalities will be listed.

4.4.2 VITAL SIGNS

Vital sign measurements include pulse, systolic blood pressure, respiratory rate, SpO2 and oral temperature.

4.4.3 PRIOR AND COMCOMITANT MEDICATIONS

Concomitant medications will be coded to the Anatomical Therapeutic Classification using the WHO Drug Dictionary. The use of prior, concomitant medications taken, and changes in concomitant medications during the study will be recorded on the CRFs. The use of concomitant medications during the study will be summarized by ATC1, ATC2 code, and treatment group for the ITT population. The summaries will be repeated for each test agent.

4.4.4 TREATMENT COMPLIANCE

Treatment compliance will be assessed based upon the administration rules for each of the test agents. For treatments that are one-time infusions (e.g. infliximab or abatacept), compliance will be summarized by the percentage of participants starting the infusion, the mean duration of the infusion, and the total amount of drug received (mg). For oral medications (e.g. cenicriviroc), compliance will be summarized by the percentage of participants taking the loading dose and starting the maintenance dose, mean number of maintenance doses, and mean duration (days) of the oral medication.

4.4.5 PREGNANCY TESTS

A set of listings of pregnancies and outcomes will be presented.

4.5 EXPLORATORY ANALYSES

The probability of falling into category “i” or better will be compared between arms for each baseline severity score category. Exploratory analyses will also compare the difference between each test agent plus SoC and SoC plus placebo in the 8-point ordinal scale at each study day for which the clinical scale is administered (in-hospital and at Days 14, 21, and 28).

A set of Bayesian analyses will be conducted for the primary endpoint using informative, enthusiastic, and skeptical priors as described in Wijeysundera et al. (2009) and Zampieri et al. (2021). Three different prior distributions will be assumed for the odds ratio parameter for the treatment effect in the proportional odds model. For the informative prior distribution, the log odds ratio (active drug vs. placebo) will have a mean of 0 and a standard deviation of 10 on the log-odds scale. For the skeptical prior, the log odds ratio will have a mean of 0 and a standard deviation for the log-odds ratio that provides 5% probability for a response that is better than the assumed alternative hypothesis (e.g. OR=1.25). For the enthusiastic prior, the assumed log-odds ratio will use the treatment effect assumed for the estimation of the sample size (e.g. OR~1.25) and a standard deviation that provides 5% probability of no benefit (e.g. OR<=1.00). Similar analyses will be conducted for the 28-day mortality binary endpoint. For these analyses, the enthusiastic prior will correspond to an odds ratio < 1.0 indicating treatment benefit for the active drug.

A separate statistical analysis plan will be created for the analyses of biomarkers.

4.6 BASELINE DESCRIPTIVE STATISTICS

Baseline characteristics will be summarized by test agent versus control for each agent and study population. For continuous measures the mean and standard deviation will be summarized. Categorical variables will be described by the proportion in each category (with the corresponding sample size). Baseline characteristics will include age, sex, race, ethnicity, symptom duration, hypertension, obesity, diabetes, coronary disease, history of heart failure, history of cancer, asthma, COPD, chronic pulmonary disease, tuberculosis, HIV, severe liver disease, severe kidney disease, days since hospitalization, and baseline 8-point ordinal score.

4.7 SUB-GROUP ANALYSES

The following subgroups have been pre-specified for the primary outcome and the two key secondary outcomes:

- Geographic region
- Duration of symptoms prior to randomization (<= Median; > Median)
- Duration of symptoms prior to randomization (based on quartiles)
- Hospitalization duration prior to randomization (<= Median, > Median)
- Baseline disease severity based on the 8-point ordinal scale

- Age (<40; 40-64; 65 and older)
- Race (White; Black/African American; Asian; Other)
- Ethnicity (Hispanic, non-Hispanic)
- Sex (Female; Male)
- Comorbidities: 0, 1, 2, 3+
- Baseline use of dexamethasone
- Co-enrollment in any ACTIV trial prior to randomization in ACTIV-1 IM
- Individual comorbidities: hypertension, obesity, diabetes, coronary disease, history of heart failure, history of cancer, asthma, COPD, chronic pulmonary disease, severe liver disease, severe kidney disease

Any subgroup that has a frequency <5% will not be generated. Subgroups with frequencies <10% may also have issues with model convergence that dictate that they cannot be generated.

The primary analysis will also be repeated for the primary endpoint and the two key secondary endpoints using the subgroups defined above. Each subgroup will be considered separately and the tabular and graphical summaries described in the previous section will be replicated for each subgroup. In addition, a forest plot will be generated to display the overall treatment hazard ratio (or RRR) estimate and CI from each of the within-stratum analyses. These analyses will be performed in the ITT and MITT populations.

Interaction tests will be conducted to determine whether the effect of treatment varies by subgroup.

In addition to the subgroup analyses described above, primary and key secondary and safety analyses will be performed within additional subgroups as outlined in **Appendix 2**.

4.8 REFERENCES

Beigel, J. H., K. M. Tomashek, L. E. Dodd, A. K. Mehta, B. S. Zingman, A. C. Kalil, E. Hohmann, H. Y. Chu, A. Luetkemeyer, S. Kline, D. Lopez de Castilla, R. W. Finberg, K. Dierberg, V. Tapson, L. Hsieh, T. F. Patterson, R. Paredes, D. A. Sweeney, W. R. Short, G. Touloumi, D. C. Lye, N. Ohmagari, M. D. Oh, G. M. Ruiz-Palacios, T. Benfield, G. Fätkenheuer, M. G. Kortepeter, R. L. Atmar, C. B. Creech, J. Lundgren, A. G. Babiker, S. Pett, J. D. Neaton, T. H. Burgess, T. Bonnett, M. Green, M. Makowski, A. Osinusi, S. Nayak and H. C. Lane (2020). "Remdesivir for the Treatment of Covid-19 - Preliminary Report." N Engl J Med.

Cao, B., Y. Wang, D. Wen, W. Liu, J. Wang, G. Fan, L. Ruan, B. Song, Y. Cai, M. Wei, X. Li, J. Xia, N. Chen, J. Xiang, T. Yu, T. Bai, X. Xie, L. Zhang, C. Li, Y. Yuan, H. Chen, H. Li, H. Huang, S. Tu, F. Gong, Y. Liu, Y. Wei, C. Dong, F. Zhou, X. Gu, J. Xu, Z. Liu, Y. Zhang, H. Li, L. Shang, K. Wang, K. Li, X. Zhou, X. Dong, Z. Qu, S. Lu, X. Hu, S. Ruan, S. Luo, J. Wu, L. Peng, F. Cheng, L. Pan, J. Zou, C. Jia, J. Wang, X. Liu, S. Wang, X. Wu, Q. Ge, J. He, H. Zhan, F. Qiu, L. Guo, C. Huang, T. Jaki, F. G. Hayden, P. W. Horby, D. Zhang and C. Wang (2020). "A Trial of Lopinavir-Ritonavir in Adults Hospitalized with Severe Covid-19." N Engl J Med 382(19): 1787-1799.

Fine, J. P. and R. J. Gray (1999). "A Proportional Hazards Model for the Subdistribution of a Competing Risk." Journal of the American Statistical Association 94(446): 496-509.

Gooley, T. A., W. Leisenring, J. Crowley and B. E. Storer (1999). "Estimation of failure probabilities in the presence of competing risks: new representations of old estimators." Stat Med 18(6): 695-706.

Gray RJ (1988). “A class of K-sample tests for comparing the cumulative incidence of a competing risk.” Ann Stat 16:1141-1154.

Holm SA. (1979) “A simple sequentially rejective multiple test procedure.” Scandanavian Journal of Statistics; 6: 65-70.

Huang, C., Y. Wang, X. Li, L. Ren, J. Zhao, Y. Hu, L. Zhang, G. Fan, J. Xu, X. Gu, Z. Cheng, T. Yu, J. Xia, Y. Wei, W. Wu, X. Xie, W. Yin, H. Li, M. Liu, Y. Xiao, H. Gao, L. Guo, J. Xie, G. Wang, R. Jiang, Z. Gao, Q. Jin, J. Wang and B. Cao (2020). "Clinical features of patients infected with 2019 novel coronavirus in Wuhan, China." Lancet 395(10223): 497-506.

Hung J, Wang S-J, O'Neill R (2007). “Statistical considerations for testing multiple endpoints in group sequential or adaptive clinical trials.” J Biopharm Stat; 17(6):1201-10.

Hwang IK, Shih WJ, J S De Cani JS (1990). “Group sequential designs using a family of type I error probability spending functions” Stat Med; 9(12):1439-45.

Smith, G. B., D. R. Prytherch, S. Jarvis, C. Kovacs, P. Meredith, P. E. Schmidt and J. Briggs (2016). "A Comparison of the Ability of the Physiologic Components of Medical Emergency Team Criteria and the

U.K. National Early Warning Score to Discriminate Patients at Risk of a Range of Adverse Clinical Outcomes." Crit Care Med 44(12): 2171-2181.

Tamhane AC, Mehta CR, Liu L (2010) "Testing a Primary and a Secondary Endpoint in a Group Sequential Design." Biometrics; 66(4): 1174–1184.

Whitehead J. (1993) "Sample Size Calculations for Ordered Categorical Data." Stat Med; vol. 12, 2257-2271.

Wijeysundera DN, Austin PC, Hux JE, Beattie WS, Laupacis A (2009). "Bayesian statistical inference enhances the interpretation of contemporary randomized controlled trials." J Clin Epidemiol; 62: 13-21 e15.

Zampieri FG, Casey JD, Shankar-Hari M; Harrell Jr. FE, and Harhay MO (2021). "Using Bayesian Methods to Augment the Interpretation of Critical Care Trials: An Overview of Theory and Example Reanalysis of the Alveolar Recruitment Trial." Am J Resp Crit Care Med. Am J Respir Crit Care Med; 203(5): 543-552.

4.9 APPENDICES

APPENDIX 1. ACTIV-1 Roster of Statisticians

Team	Name	Email	Attends DSMB Meetings
Unblinded Statistical Team	Michael Proschan	[redacted]	X
	Tyler Bonnet	[redacted]	X
	Jyotsna Garg	[redacted]	X
	Eric Yow	[redacted]	X
	Zhen Huang	[redacted]	
	Hwasoon Kim	[redacted]	
	Hussein Al-Khalidi	[redacted]	X
	Sean O'Brien	[redacted]	X
Blinded Statistical Team	Lisa LaVange	[redacted]	X
	Steven McNulty	[redacted]	X
	Adam Silverstein	[redacted]	
	Jun Wen	[redacted]	
	Pearl Zakroysky	[redacted]	X
	Susan Halabi	[redacted]	X
	Kevin Anstrom	[redacted]	X

APPENDIX 2. Additional Tables and Listings

Table	Demographic and Baseline Characteristics – US	Intent-to-Treat Population
Table	Demographic and Baseline Characteristics – Latin America	Intent-to-Treat Population
Table	Demographic and Baseline Characteristics by Geographic Region - Argentina	Intent-to-Treat Population
Table	Demographic and Baseline Characteristics by Geographic Region - Brazil	Intent-to-Treat Population
Table	Demographic and Baseline Characteristics by Geographic Region - Mexico	Intent-to-Treat Population
Table	Demographic and Baseline Characteristics by Geographic Region - Peru	Intent-to-Treat Population
Table	Demographic and Baseline Characteristics by Geographic Region – USA – Northeast	Intent-to-Treat Population
Table	Demographic and Baseline Characteristics by Geographic Region – USA – Midwest	Intent-to-Treat Population
Table	Demographic and Baseline Characteristics by Geographic Region – USA – South	Intent-to-Treat Population
Table	Demographic and Baseline Characteristics by Geographic Region – USA – West	Intent-to-Treat Population
Table	Demographic and Baseline Characteristics – Moderate Disease Severity at Baseline	Intent-to-Treat Population
Table	Demographic and Baseline Characteristics – Severe Disease Severity at Baseline	Intent-to-Treat Population
Table	Time to Recovery by US vs. Latin America	Intent-to-Treat Population
Table	Time to Recovery by Disease Severity at Baseline (Moderate vs. Severe)	Intent-to-Treat Population
Table	Time to Recovery by Remdesivir Use at Randomization	Intent-to-Treat Population
Table	Time to Recovery by Steroid Use at Randomization	Intent-to-Treat Population
Table	Time to Recovery by Immune Modulator Use at Randomization	Intent-to-Treat Population
Table	Time to Recovery by Anticoagulant Use at Randomization	Intent-to-Treat Population
Table	Time to Recovery by Baseline CRP (<=75 mg/L; >75 mg/L)	Intent-to-Treat Population
Table	Proportional Odds Model of Clinical Status Score at Day 14 by US vs. Latin America	Intent-to-Treat Population
Table	Proportional Odds Model of Clinical Status Score at Day 14 by Disease Severity at Baseline (Moderate vs. Severe)	Intent-to-Treat Population

Table	Proportional Odds Model of Clinical Status Score at Day 28 by US vs. Latin America	Intent-to-Treat Population
Table	Proportional Odds Model of Clinical Status Score at Day 28 by Geographic Region	Intent-to-Treat Population
Table	Proportional Odds Model of Clinical Status Score at Day 28 by Disease Severity at Baseline (Moderate vs. Severe)	Intent-to-Treat Population
Table	28-Day Mortality by US vs. Latin America	Intent-to-Treat Population
Table	28-Day Mortality by Disease Severity at Baseline (Moderate vs. Severe)	Intent-to-Treat Population
Table	28-Day Mortality by Remdesivir Use at Randomization	Intent-to-Treat Population
Table	28-Day Mortality by Steroid Use at Randomization	Intent-to-Treat Population
Table	28-Day Mortality by Immune Modulator Use at Randomization	Intent-to-Treat Population
Table	28-Day Mortality by Anticoagulant Use at Randomization	Intent-to-Treat Population
Table	28-Day Mortality by Baseline CRP (<=75 mg/L; >75 mg/L)	Intent-to-Treat Population
Table	14-Day and 28-Day Mortality Rates by US vs. Latin America	Intent-to-Treat Population
Table	14-Day and 28-Day Mortality Rates by Geographic Region	Intent-to-Treat Population
Table	14-Day and 28-Day Mortality Rates by Disease Severity at Baseline (Moderate vs. Severe)	Intent-to-Treat Population
Table	14-Day and 28-Day Mortality Rates by Baseline CRP (<=75 mg/L; >75 mg/L)	Intent-to-Treat Population
Table	Overview of Safety Composite, Individual Endpoints, and Adverse Events through Day 28 - US	Modified Intent-to-Treat Population
Table	Overview of Safety Composite, Individual Endpoints, and Adverse Events through Day 28 – Latin America	Modified Intent-to-Treat Population
Table	Overview of Safety Composite, Individual Endpoints, and Adverse Events through Day 28 by Geographic Region - Argentina	Modified Intent-to-Treat Population
Table	Overview of Safety Composite, Individual Endpoints, and Adverse Events through Day 28 by Geographic Region – Brazil	Modified Intent-to-Treat Population
Table	Overview of Safety Composite, Individual Endpoints, and Adverse Events through Day 28 by Geographic Region – Mexico	Modified Intent-to-Treat Population
Table	Overview of Safety Composite, Individual Endpoints, and Adverse Events through Day 28 by Geographic Region – Peru	Modified Intent-to-Treat Population
Table	Overview of Safety Composite, Individual Endpoints, and Adverse Events	Modified Intent-to-Treat

	through Day 28 by Geographic Region – USA – Northeast	Population
Table	Overview of Safety Composite, Individual Endpoints, and Adverse Events through Day 28 by Geographic Region – USA – Midwest	Modified Intent-to-Treat Population
Table	Overview of Safety Composite, Individual Endpoints, and Adverse Events through Day 28 by Geographic Region – USA – South	Modified Intent-to-Treat Population
Table	Overview of Safety Composite, Individual Endpoints, and Adverse Events through Day 28 by Geographic Region – USA – West	Modified Intent-to-Treat Population
Table	Overview of Safety Composite, Individual Endpoints, and Adverse Events through Day 28 – Moderate Disease Severity at Baseline	Modified Intent-to-Treat Population
Table	Overview of Safety Composite, Individual Endpoints, and Adverse Events through Day 28 – Severe Disease Severity at Baseline	Modified Intent-to-Treat Population
Table	Most Common Adverse Events through Day 28 Occurring in >10% of Infliximab Subjects	Modified Intent-to-Treat Population
Table	Most Common Adverse Events through Day 60 Occurring in >10% of Infliximab Subjects	Modified Intent-to-Treat Population
Listing	Prior Medications	Intent-to-Treat Population
Listing	Concomitant Medications	Intent-to-Treat Population

04_SAP_ACTIV1_IM_11APR2022_clean

Final Audit Report 2022-04-12

Created:	2022-04-11
By:	rose beci (beci0001@duke.edu)
Status:	Signed
Transaction ID:	CBJCHBCAABAAV5E68nB7XxM9S6KJ2NYIp5YBkhrJV7J4

"04_SAP_ACTIV1_IM_11APR2022_clean" History

Document created by rose beci (beci0001@duke.edu)

2022-04-11 - 8:41:01 PM GMT- IP address: 136.226.49.3

Document emailed to Danny Benjamin (danny.benjamin@duke.edu) for signature

2022-04-11 - 8:41:56 PM GMT

Email viewed by Danny Benjamin (danny.benjamin@duke.edu)

2022-04-11 - 10:17:02 PM GMT- IP address: 104.28.33.33

Document e-signed by Danny Benjamin (danny.benjamin@duke.edu)

Signature Date: 2022-04-11 - 10:18:13 PM GMT - Time Source: server- IP address: 174.216.2.166

Document emailed to Soju Chang (soju.chang@nih.gov) for signature

2022-04-11 - 10:18:15 PM GMT

Email viewed by Soju Chang (soju.chang@nih.gov)

2022-04-11 - 10:37:14 PM GMT- IP address: 172.225.29.21

Document e-signed by Soju Chang (soju.chang@nih.gov)

Signature Date: 2022-04-12 - 0:01:29 AM GMT - Time Source: server- IP address: 128.231.234.26

Document emailed to Lisa LaVange (lisa.lavange@unc.edu) for signature

2022-04-12 - 0:01:31 AM GMT

Email viewed by Lisa LaVange (lisa.lavange@unc.edu)

2022-04-12 - 1:27:32 PM GMT- IP address: 152.19.206.98

Document e-signed by Lisa LaVange (lisa.lavange@unc.edu)

Signature Date: 2022-04-12 - 1:28:08 PM GMT - Time Source: server- IP address: 152.19.206.98

Document emailed to William Powderly (wpowderly@wustl.edu) for signature

2022-04-12 - 1:28:10 PM GMT

Email viewed by William Powderly (wpowderly@wustl.edu)

2022-04-12 - 1:30:52 PM GMT- IP address: 108.207.150.118

Document e-signed by William Powderly (wpowderly@wustl.edu)

Signature Date: 2022-04-12 - 1:31:22 PM GMT - Time Source: server- IP address: 108.207.150.118

Agreement completed.

2022-04-12 - 1:31:22 PM GMT

Master Protocol for Immune Modulators for Treating COVID-19

Protocol: ACTIV-1 IM

STATISTICAL ANALYSIS PLAN (SAP)

Protocol Version 12.0

Protocol Version Date: 08 SEP02 DEC 2020-

SAP Version Number: 15.0
25 NOV 2020

11 APR 2022

Daniel K. Benjamin, MD, MPH, PhD

Kiser-Arena Distinguished Professor, Duke University Pediatrics

Duke Clinical Research Institute

300 W. Morgan Street, Suite 800

Durham Center

Durham, NC 27701

Tel: 919-668-7081

CONFIDENTIAL

E-mail: [redacted]

CONFIDENTIAL

SAP Approval Page

Study Title: Master Protocol for Immune Modulators for Treating COVID-19

Version: 15.0

Date of Issue: November 25, 202011 APR 2022

Study Sponsor: Daniel K. Benjamin, MD, MPH, PhD

Kiser-Arena Distinguished Professor, Duke University Pediatrics Duke

Clinical Research Institute

300 W. Morgan Street, Suite 800 / Durham CenterCentre / Durham, NC 27701
Durham, NC 27701

We, the undersigned, have read and approve of this SAP and agree on its content.

[redacted] Electronically signed by: danny benjamin
Reason: Approved
Date: Dec 2, 2020 _ 12:26 EST

CONFIDENTIAL

[redacted]

Electronically signed by: Danny Benjamin
Reason: Approved
Date: Apr 11, 2022 18:18 EDT

Sponsor Representative – Daniel K. Benjamin, MD, MPH, PhD

[redacted]

Electronically signed by: Jane C. Atkinson
Reason: Approved
Date: Dec 2, 2020 13:26 EST

[redacted]

Electronically signed by: Soju Chang
Reason: Approved
Date: Apr 11, 2022 20:01 EDT

Sponsor Representative – Jane Atkinson, DDSSoju Chang, MD, MPH

[redacted]

Electronically signed by: Lisa LaVange
Reason: Approved
Date: Dec 3, 2020 20:39 EST

[redacted]

Electronically signed by: Lisa LaVange
Reason: Approved
Date: Apr 12, 2022 09:28 EDT

ACTIV-1 Protocol Statistician – Lisa LaVange, PhD

[redacted]

Electronically signed by: Michael Proschan
Reason: Approved
Date: Dec 4, 2020 10:44 EST

ACTIV-1 Protocol Statistician – Michael Proschan, PhD

[redacted]

Electronically signed by: William Powderly
Reason: Approved
Date: Dec 4, 2020 09:53 CST

[redacted]

Electronically signed by: William Powderly
Reason: Approved
Date: Apr 12, 2022 08:31 CDT

ACTIV-1 Protocol Chair – William G. Powderly, MD

CONFIDENTIAL

TABLE OF CONTENTS

List of Abbreviations 45

1.0 .... Study and Document Overview 5

2.0 Study Objectives 6

3.0 Statistical Hypotheses and Endpoints 8

3.1 Study Definitions and Derived Variables 11

3.1.1 VISIT DAY 11

3.1.2 Measures of Clinical Support, Limitations, and Infection Control 11

3.1.3 Extrapulmonary Manifestations 11

3.1.4 National Early Warning Score (NEWS) 13

3.2 Power and Sample Size 14

3.3 Randomization Scheme 15

3.4 Interim Analyses and Data Monitoring 16

3.4.1 Interim Safety Analyses 16

1.0 Study and Document Overview 7

2.0 Study Objectives and Estimands 8

3.0 Statistical Hypotheses and Endpoints 11

3.1 Study Definitions and Derived Variables 14

3.1.1 Visit Day 14

3.1.2 8-Point Ordinal Scale 14

3.1.3 Measures of Clinical Support, Limitations, and Infection Control 14

3.1.4 Extrapulmonary Manifestations 15

3.1.5 National Early Warning Score (NEWS) 16

3.2 Power and Sample Size 18

3.3 Randomization Scheme 19

3.4.23.4 Interim Analyses for Futility and Efficacy Review ...16Data Monitoring 20

3.4.33.4.1 Adaptations .... 17Interim Safety Analyses 21

3.53.4.2 Data Sources........18Interim Analyses for Futility and Efficacy Review 21

3.63.4.3 Documentation Convention 18Adaptations 23

3.7 Verification of Results 18

CONFIDENTIAL

3.8 Subject Disposition 18
3.9 Populations for Analyses 19
4.0 Statistical Analyses 20
4.1 General Approach 20
3.5 Data Sources 25
3.6 Documentation Convention 26
3.7 Verification of Results 26
3.8 Subject Disposition 26
3.9 Populations for Analyses 27
4.24.0 Analysis of the Primary Efficacy Endpoint .... 22Statistical Analyses 28
4.34.1 Analysis of the Secondary efficacy Endpoint(s) ....23General Approach 28
4.4 Safety Analyses 25Analysis of the Primary and Key Secondary Efficacy Endpoints 31
4.4.1 Laboratory Data 25
4.4.2 Vital Signs 26
4.4.3 Prior and Comcomitant Medications 26
4.4.4 Treatment Compliance 26
4.4.5 Pregnancy Tests 26
4.5 Exploratory Analyses 26
4.6 Baseline Descriptive Statistics 27
4.7 Sub-Group Analyses 27
4.8 References 28
4.9 Appendices 29
4.2
4.3 Analysis of the Other Secondary Efficacy Endpoints 36
4.4 Safety Analyses 39
4.4.1 Laboratory Data 40
4.4.2 Vital Signs 41
4.4.3 Prior and Comcomitant Medications 41
4.4.4 Treatment Compliance 41
4.4.5 Pregnancy Tests 41
4.5 Exploratory Analyses 41

CONFIDENTIAL

4.6 Baseline Descriptive Statistics 42

4.7 Sub-Group Analyses 42

4.8 References 44

4.9 Appendices 46

CONFIDENTIAL

CONFIDENTIAL

LIST OF ABBREVIATIONS

ACTIV	Accelerating COVID-19 Therapeutic Interventions and Vaccines
ADaM	Analysis Data Model
AE	Adverse Event
ALT	Alanine transaminase
AST	Aspartate transaminase
ATC	Anatomical Therapeutic Classification
BMI	Body Mass Index
CDISC	Clinical Data Interchange Standards Consortium
CI	Confidence Interval
COVID-19	Coronavirus
CRP	C-Reactive Protein
CTCAE	Common Terminology Criteria for Adverse Events
DAIDS	Division of AIDS
DCRI	Duke Clinical Research Institute
DSMB	Data and Safety Monitoring Board
ECMO	Extracorporeal Membrane Oxygenation
eCRF	Electronic Case Report Form
EOS	End of Study
ET	Early Termination
FU	Follow-up
ITT	Intent-to-Treat
LLN	Lower Limit of Normal
MedDRA	Medical Dictionary of Regulatory Activities
MITT	Modified Intent-to-Treat

CONFIDENTIAL

NEWS	National Early Warning Score
PT	Preferred Term
Q1	25th Percentile (1st Quartile)
Q3	75th Percentile (3rd Quartile)
RR	Respiratory Rate
RRR	Recovery Rate Ratio
SAE	Serious Adverse Event
SAP	Statistical Analysis Plan
SARS-CoV-2	Severe Acute Respiratory Syndrome Coronavirus 2
SD	Standard Deviation
SDTM	Study Data Tabulation Model
SE	Standard Error
SoC	Standard of Care
ULN	Upper Limit of Normal
WBC	White Blood Cell

CONFIDENTIAL

1.0 STUDY AND DOCUMENT OVERVIEW

ACTIV-1 IM is a master protocol designed to evaluate multiple investigational agents for the treatment of moderately or severely ill patients infected with severe acute respiratory syndrome coronavirus 2 (SARS-CoV-2). ACTIV-1 IM is a randomized, controlled, double-blinded trial which will evaluate each agent with respect to speed of recovery, mortality, illness severity, and hospital resource utilization. Each agent will be evaluated as add-on therapy to the standard of care (SoC) in use at the local clinics, including remdesivir (provided).. The SoC may change during the course of the study based on other research findings. Comparisons of the investigational agents among themselves is not a research objective.

This document describes the planned statistical analyses that will be conducted at the completion of the ACTIV-1 IM study for each of the investigational agents. Once the initial unblinded data review has occurred, only the blinded statistics team members will be allowed to modify the SAP (see **Appendix 1** for the list of blinded and unblinded statisticians). The initial unblinded analyses are expected to review data from the first 40 participants with at least 15 days of follow-up. That meeting is planned for January 2021.

The study population corresponds to moderately and severely ill adults (≥18 years old) with the coronavirus disease 2019 (COVID-19) virus. Recruitment will target patients already hospitalized for treatment of COVID-19 infection as well as patients being treated for COVID-19 infection in Emergency Departments while waiting to be admitted to the hospital. Patients both in and out of the intensive care unit (ICU) will be included in the study population.

Enrollment began in October 2020 with 3 agents selected for initial evaluation. Up to 5 agents may be evaluated in ACTIV-1 IM.

The study period is 29 days, with assessments on each day of the hospital stay. If the participants are discharged from the hospital, they will have a study visit at Days 8, 11, 15, 22, and 29. For discharged participants, it is preferred that the Day 8, 11, 15, and 29 visits are in person to obtain safety laboratory tests (Day 29) and blood (serum and plasma) samples for secondary research (Day 29) as well as clinical outcome data. Clinical outcome is assessed for the previous day; for example, study visit Days 8, 11, 22, and 29 represent data for Days 7, 10, 21, and 28. However, infection control or other restrictions may limit the ability of the participant to return to the clinic. In this case, these visits may be conducted by phone, and only clinical data will be obtained. The Day 22 visit does not have laboratory tests or collection of samples and is conducted by phone.
There will also be a safety and clinical status assessment at 60 days conducted by phone. Treatment periods may vary by agent.

CONFIDENTIAL

The trial is adaptive in that interim analyses are planned to assess the futility of each agent, with the goal of discontinuing those with lower probabilities of success to more effectively utilize trial resources for the remaining agents. Additionally, interim analyses are planned for early stopping for efficacy.

CONFIDENTIAL

Alpha spending functions are used to appropriately control the probability of making an erroneous conclusion at the interim and final analyses.

Safety monitoring will be performed throughout the trial, and formal stopping rules for each agent will be adopted. The Data and Safety Monitoring Board (DSMB) established for ACTIV-1 IM will have oversight responsibility for the study.

CONFIDENTIAL

The effectiveness of each therapeutic agent as add-on therapy to SoC plus remdesivir (provided) will be evaluated based on the primary endpoint of time to recovery bythrough Day 2928. The sample size requirements are based on the ability to detect a moderate improvement in time to recovery for each agent. A total of 788 recoveries are required for each comparison to provide approximately 85% power to detect a recovery rate ratio (RRR) of 1.25. Assuming 73% of participants achieve recovery inwithin 28 days, consistent with the ACTT-1 (Beigel, Tomashek et al. 2020) results, the total sample size to evaluate 1, 2, and 3 agents in ACTIV-1 IM is approximately 1080, 1620, and 2160, respectively. Because each agent is being compared to SoC plus placebo with no between-agent comparisons, no multiplicity adjustments for multiple agents are planned.

2.0 STUDY OBJECTIVES AND ESTIMANDS

2.0

Primary Objective:

- To evaluate the clinical efficacy of different investigational therapeutics relative to the control arm in adults hospitalized with COVID-19 with respect to time to recovery bythrough Day 2928 from randomization.

Key Secondary Objectives:

- To evaluate the clinical efficacy of different investigational therapeutics relative to the control arm in adults hospitalized with COVID-19 according to:
    - Clinical status (8-point ordinal scale) assessed on Day 1514 and Day 2928
    - Mortality
        - 14-day mortality

28-day mortality

Summaries of the questions of interest and descriptions of the estimand attributes are shown in **Table 1**.

CONFIDENTIAL

Table 1. Study Objectives and Estimands.

**Question of Interest**	**Objective Description / Study Population**	**Endpoint**	**Intercurrent Events**	**Population Summary**
Does drug X reduce the time-to-recovery compared with standard-of-care alone for patients hospitalized with moderate or severe COVID-19?	Primary Objective of the Study / All Randomized Participants	Time to recovery defined as the first day with an 8-point ordinal scale score of ≥ 6.	Death from any cause (a semi-competing event). Once an individual has experienced death, they no longer have the opportunity to 'recover'. All other intercurrent events will be ignored.	Recovery rate ratio (RRR) and its associated confidence interval from a Fine-Gray model. Values greater than 1.0 indicate a benefit for drug X. A value of 2.0 suggests a larger benefit than 1.25. Stratification factors will include region and baseline severity (2, 3, 4, or 5). Other covariates in the model will include age and sex.
Does drug X improve the ordinal response of the 8-point ordinal scale compared with standard-of-care alone?	Key Secondary Objective of the Study / All Randomized Participants*	The 8-point ordinal scale score on Day 14. A supplemental analysis will be completed on the same scale at Day 28.	Deaths are placed as the worst possible score on the ordinal scale. All other intercurrent events will be ignored in these analyses.	Odds ratio estimate based on a proportional odds model. Values above 1.0 suggest benefit from drug X. The model will include baseline severity (2, 3, 4, or 5), region, sex, and age (continuous) as covariates.
Does drug X reduce the rate of death over 28 days compared with standard-of-care alone?	Key Secondary Objective of the Study / All Randomized Participants*	Death observed between randomization through Day 28	All other intercurrent events will be ignored in these analyses.	An odds ratio from a logistic regression model with indicator variables for treatment group,

				8-point ordinal scale (2, 3, 4, or 5) at baseline, region, sex, and age (continuous).

*For the test agent and pooled control group

o Additional Secondary Objectives:

- To evaluate the clinical efficacy of different investigational therapeutics relative to the control arm as assessed by clinical severity and hospitalization.

arm as assessed by:

- **Clinical Severity**
  - Ordinal scale:
    - Time to an improvement of one category and two categories from Day 1 (baseline) using an 8-point ordinal scale.
    - Subject clinical status using 8-point ordinal scale at Days 3, 5, 8, 11, 15, 22, and 29.
    - Mean change in the 8-point ordinal scale from Day 1 to Days 3, 5, 8, 11, 15, 22, and 29.
    - Total severity score (TSS): 8-point ordinal scale summarized as a daily score (for days collected) averaged over time from Day 1 through Day 29
  - Oxygenation:
    - Oxygenation use up to Day 29.
    - Incidence and duration of new oxygen use during the study.
  - Non-invasive ventilation/high flow oxygen:
    - Non-invasive ventilation/high flow oxygen use up to Day 29.

CONFIDENTIAL

- Incidence and duration of new non-invasive ventilation or high flow oxygen use during the study.
- Invasive Mechanical Ventilation / extracorporeal membrane oxygenation (ECMO):
  - Ventilator / ECMO use up to Day 29.
  - Incidence and duration of new mechanical ventilation or ECMO use during the study.
- **Hospitalization**
  - Duration of hospitalization (days) from randomization to discharge.
- To evaluate clinical status at Day 60.
- To evaluate the safety of different investigational therapeutics relative to the control arm as assessed by:.
- Cumulative incidence of SAEs through Day 29.
- Cumulative incidence of Grade 3 and 4 clinical and/or laboratory AEs through Day 29.
- Discontinuation or temporary suspension of study product administrations (for any reason).
- Changes in white blood cell (WBC) count with differential, hemoglobin, platelets, creatinine, glucose, total bilirubin, alanine aminotransferase (ALT), aspartate aminotransferase (AST), alkaline phosphatase (ALP), prothrombin time (PT)/international normalized ratio (INR), d- dimer, cardiac troponin, and C-reactive protein (CRP) over time (analysis of lab values in addition to AEs noted above).
- To evaluate the impact of different investigational therapeutics relative to the control arm of extrapulmonary manifestations of COVID-19.

Exploratory Objectives:

- To assess the National Early Warning Score (NEWS):).
  - Time to discharge or to a NEWS of ≤ 2 and maintained for 24 hours, whichever occurs first.
- Change from Day 1 to Days 3, 5, 8, 11, 15, and 29 in NEWS.
- To collect blood samples for future research.

CONFIDENTIAL

3.0 STATISTICAL HYPOTHESES AND ENDPOINTS

~~Primary:~~

- The primary null hypothesis being tested is that time-to-recovery does not differ between each test agent (plus SoC) and the pooled control group, consisting of patients receiving SoC plus placebo. The alternative hypothesis is that the test agent and pooled control group differ in time-to-recovery ~~in~~with the hope that the test agent group has a shorter time-to-~~-~~recovery than the pooled control group although a two-tailed test will be used. If Drug A, B, and C denote the initial set of agents to be tested in ACTIV-1 IM plus SoC, and S denotes patients receiving SoC plus placebo, then the primary hypotheses are:

H_{01}: $TR_A = TR_S$ vs H_{A1}: $TR_A \neq TR_S$

H_{02}: $TR_B = TR_S$ vs H_{A2}: $TR_B \neq TR_S$

H_{03}: $TR_C = TR_S$ vs H_{A3}: $TR_C \neq TR_S$

~~There are two key secondary endpoints that will be evaluated as supportive evidence:~~

1. ~~The distribution of the 8-point ordinal scale (see definition below) at Day 15 and Day 29. For this, the parameter of interest is the "common odds ratio," which quantifies the shift in the severity distribution resulting from treatment. The odds ratio will be defined such that a value greater than 1 implies better outcomes with active drug compared to control and a value less than 1 implies worse outcomes with active drug compared to control. In this case, for an efficacious treatment, an odds ratio greater than 1 quantifies an improvement in disease severity; a value of 2 indicates a bigger improvement than a value of 1.25. The null hypothesis to be tested is that the odds of improvement on the ordinal scale is the same for the placebo and experimental treatment arms (i.e., the common odds ratio is 1). It is worth noting that, for large sample sizes, the test based on the proportional odds model is nearly the same as the Wilcoxon rank sum test.~~
2. ~~Mortality will be evaluated with standard survival analysis techniques. Kaplan-Meier curves and associated stratified log-rank statistics will be generated to compare mortality between each test agent and SoC. The stratification factors are based on the combination of geographic region and baseline disease severity Mortality is evaluated at Days 15 and 29 to assess treatment effects in 14-day and 28-day mortality.~~

- ~~8-Point Ordinal Scale~~
  - ~~The 8-point ordinal scale is the basis for the primary and key secondary clinical endpoints in the study, namely, time to recovery, improvement in disease severity, and mortality.~~
  - ~~The scale used in this study is as follows (from worst to best):~~
    - ~~1: Death;~~
    - ~~2: Hospitalized, on invasive mechanical ventilation or ECMO;~~

~~CONFIDENTIAL~~

- 3: Hospitalized, on non-invasive ventilation or high flow oxygen devices;
- 4: Hospitalized, requiring supplemental oxygen;
- 5: Hospitalized, not requiring supplemental oxygen – requiring ongoing medical in-patient care (COVID-19 related or otherwise);
  - 6: Hospitalized, not requiring supplemental oxygen – no longer requires ongoing medical in-patient care;
  - This would include those kept in hospital for quarantine/infection control/social reasons, awaiting bed in rehabilitation facility or homecare, etc.
- 7: Not hospitalized, limitation on activities and/or requiring home oxygen;
- 8: Not hospitalized, no limitations on activities

- **Primary Efficacy Endpoint**
  - - Time to recovery bythrough Day 2928

    - Day of recovery is defined as the first day on which the participant satisfies one of the following three categories from the 8-point ordinal scale: (defined in Section 3.1.2):
      - Hospitalized, not requiring supplemental oxygen – no longer requires ongoing medical in-patient care;
      - Not hospitalized, limitation on activities and/or requiring home oxygen;
      - Not hospitalized, no limitations on activities.

  - There are two key secondary hypotheses that will be evaluated as supportive evidence.

- **Key Secondary** Endpoints
  - **Endpoint 1** - Clinical status (8-point ordinal scale) assessed on Day 15 and Day 29 for the previous day14

    The null hypothesis to be tested is that the odds of improvement on the 8-point ordinal scale is the same for the placebo and experimental treatment arms (i.e., the common odds ratio is 1) on Day 14. For this comparison, the parameter of

  - interest is the "common odds ratio," which quantifies the shift in the severity distribution resulting from treatment. 14-day and 28-day mortality assessed using date and cause of death (if applicable)

    The odds ratio will be defined such that a value greater than 1 implies better outcomes with active drug compared to placebo control.

CONFIDENTIAL

- **Key Secondary Endpoint 2** - Mortality

  The null hypothesis to be tested is that there is no difference in mortality rates between treatment groups. Mortality through Day 28 will be analyzed using a logistic regression model with an indicator variable for the treatment group, 8-point ordinal scale (2, 3, 4, or 5) at baseline, region, sex, and age.

- Additional Secondary Endpoints
  - Clinical severity assessedTime to an improvement of one category and two categories from Day 0 (baseline) using the 8-point ordinal scale-daily while hospitalized and on.
  - Mean change in the 8-point ordinal scale from baseline to Days 15, 222, 4, 7, 10, 14, 21, 28, and 2960.
  - Differences in total severity score (TSS): 8-point ordinal scale summarized as a daily score (for days collected) averaged over time from baseline through Day 28.
  - Days alive and free of supplemental oxygen (if applicable) up to Day 29through 28.
  - Incidence and duration of new oxygen use during the study. This includes new supplemental oxygen, non-invasive ventilation/high flow oxygen use, and invasive mechanical ventilation/extracorporeal membrane oxygenation (ECMO).
  - Days alive and free of non-invasive ventilation/high flow oxygen (if applicable) up touse through Day 2928.
  - Incidence and duration of new non-invasive ventilation or high flow oxygen use during the study. This includes new non-invasive ventilation or high flow oxygen use as well as invasive mechanical ventilation/ECMO.
  - Days alive and free of invasive mechanical ventilation/ECMO (if applicable) up tothrough Day 2928.
  - Incidence and duration of new mechanical ventilation/ECMO use during the study.
- Days alive and out of hospital up to Day 29
  - Clinical status (8-point ordinal scale) athospitalization through Day 6028.

- Key Safety Endpoints
  - Serious Adverse Events (SAEs)
  - Grade 3 and 4 Adverse Events (AEs)
  - Baseline laboratory tests include CBC with differential (including absolute neutrophil count and absolute lymphocyte count), ALT, AST, ALP, total bilirubin, creatinine (with calculated eGFR), glucose, Troponin, PT/INR, d-

CONFIDENTIAL

dimer, and C-reactive protein. *On Days 3, 5, 8, 11, 15, and 29, safety laboratory tests include WBC with differential, hemoglobin, platelets, ALT,- AST, ALP, total bilirubin, creatinine (with calculated*

CONFIDENTIAL

AST, ALP, total bilirubin, creatinine (with calculated eGFR), and glucose. On Day 8 or on day of discharge (if less than 24 hours prior to Day 8), the following ***additional*** *laboratory assessments are performed: Troponin, PT/INR, d-dimer, and C-reactive protein.*
   - Incidence of individual and "any" specified extrapulmonary manifestations atup to Day 29

- Exploratory Endpoints
   - NEWS assessed daily while hospitalized (Day 1 – discharge) and on Days 15 and 29, if feasible
   - Blood draws on Days 1, 3, 8, 15, and 29

CONFIDENTIAL

3.1 STUDY DEFINITIONS AND DERIVED VARIABLES

3.1.1 VISIT DAY

The baseline visit is the date of randomization (Day 1).. Post-baseline study day visits are documenting the clinical information through the completion of the prior day (e.g. Day 2 is collecting the post-randomization data Day 1 and Day 29 is collecting data through of endthat encompasses all of Day 28). However, we will present visit day as actual day that is being assessed.

3.1.2 8-POINT ORDINAL SCALE

- The 8-point ordinal scale is the basis for the primary and key secondary clinical endpoints in the study, namely, time to recovery, improvement in disease severity, and mortality.
- The scale used in this study is as follows (from worst to best):
  - 1: Death;
  - 2: Hospitalized, on invasive mechanical ventilation or ECMO;
  - 3: Hospitalized, on non-invasive ventilation or high flow oxygen devices;
  - 4: Hospitalized, requiring supplemental oxygen;
  - 5: Hospitalized, not requiring supplemental oxygen – requiring ongoing medical in-patient care (COVID-19 related or otherwise);
  - 6: Hospitalized, not requiring supplemental oxygen – no longer requires ongoing medical in-patient care;
    - This would include those kept in hospital for quarantine/infection control/social reasons, awaiting bed in rehabilitation facility or homecare, etc.
  - 7: Not hospitalized, limitation on activities and/or requiring home oxygen;
  - 8: Not hospitalized, no limitations on activities

3.1.23.1.3 MEASURES OF CLINICAL SUPPORT, LIMITATIONS, AND INFECTION CONTROL

The participant's clinical status will be captured on each study day while hospitalized through Day 29., for the previous day. Clinical data status is measured twice on Day 1, once at the time of randomization and once at the Day 2 visit for the post-randomization time period. If a participant is discharged prior to Day 15, clinical status is collected on Day 8, Day 11, Day 15, and Day 29, if the participant returns for an in-person clinic visit or by phone if an in-person visit is not possible. Clinical status will also be captured on Day 22 during a phone visit. Clinical status is largely measured by the 8--point ordinal scale and the NEWS. Unlike the NEWS, the ordinal scale can also be evaluated over the phone if the discharged participant is unable to return for visits on Day 8, 11, 15, or 29 as well as on Day 22.

CONFIDENTIAL

Improvement will be defined as an increase of at least one point on the 8-point scale compared to the baseline value (e.g. from 4 to 5) and the time to improvement will be defined as the elapsed time (in days) from Day 1baseline to the earliest day of observed improvement.

Any subjects that are lost to follow-up or terminated early prior to an observed improvement will be censored at the day of their last observed assessment. Subjects who complete follow-up but do not experience improvement will be censored at the day of their Day 29 visit.

3.1.4 3.1.3 EXTRAPULMONARY MANIFESTATIONS

The presence of the extrapulmonary manifestation of disease during the course of hospitalization will be assessed and reported at discharge. These tables will be reported with stratification by the participant's baseline clinical status. Specifically, clinical organ failure is defined by development of any one or more of the following clinical events:

a. Respiratory dysfunction:

1. Respiratory failure defined as receipt of high flow nasal oxygen, noninvasive ventilation, invasive mechanical ventilation or ECMO

b. Cardiac and vascular dysfunction:

1. Myocardial infarction
2. Myocarditis or pericarditis
3. Congestive heart failure: new onset NYHA class III or IV, or worsening to class III or IV
4. Hypotension requiring institution of vasopressor therapy

c. Renal dysfunction:

1. New requirement for renal replacement therapy

d. Hepatic dysfunction:

1. Hepatic decompensation

CONFIDENTIAL

e. Neurological dysfunction

CONFIDENTIAL

1. Acute delirium
2. Cerebrovascular event (stroke, cerebrovascular accident [CVA])
3. Transient ischemic events (i.e., CVA symptomatology resolving <24 hrs)
4. Encephalitis, meningitis or myelitis

f. Hematological dysfunction:

1. Disseminated intravascular coagulation
2. New arterial or venous thromboembolic events, including pulmonary embolism and deep vein thrombosis
3. Major bleeding events (>2 units of blood within 24 hours, bleeding at a critical site (intracranial, intraspinal, intraocular, pericardial, intraarticular, intramuscular with compartment syndrome, or retroperitoneal), or fatal bleeding).

g. Secondary infection / superinfection:

1. Intercurrent, at least probable, documented serious disease caused by an infection other than SARS-CoV2, requiring antimicrobial administration and care.
2. A complete list of secondary infections / superinfections is provided in the study manual of procedures.

CONFIDENTIAL

3.1.5 3.1.4 NATIONAL EARLY WARNING SCORE (NEWS)

Vital signs and other clinical assessments are collected for the calculation of the NEWS, and include temperature, systolic blood pressure, heart rate, respiratory rate, O_2 saturation and level of consciousness. Vital signs collected per standard of care can be used. NEWS has demonstrated an ability to discriminate subjects at risk of poor outcomes. (Smith, Prytherch et al. 2016). This score is based on 7 clinical parameters (see **Table 12**). The NEWS is being used as an exploratory efficacy measure. The components of NEWS should be evaluated daily while hospitalized. It can be performed concurrently with the Ordinal Scale. This should be evaluated at a consistent time for each study day and prior to administration of study product. The 7 parameters can be obtained from the hospital chart or electronic medical record (EMR) using the last measurement prior to the time of assessment (including parameters collected prior to the time of consent) and a numeric score is given for each parameter (e.g., a respiratory rate [RR] of 9 breaths per minute is one point, oxygen saturation of 92% is two points). This is recorded for the day obtained (i.e., on Day 3, the vital signs and other parameters from Day 3 will be-

CONFIDENTIAL

used to obtain NEWS for Day 3). ECMO and mechanically ventilated participants should be assigned a score of 3 for RR (RR ≤8 breaths per minute) regardless of the ventilator setting. Participants on ECMO should get a score of 3 for heart rate since they are on cardiopulmonary bypass.

Table 12. National Early Warning Score (NEWS)

PHYSIOLOGICAL PARAMETERS	3	2	1	0	1	2	3
Respiration Rate (breaths per minute)	≤8 [a]		9 – 11	12 – 20		21 – 24	≥25
Oxygen Saturation (%)	≤91	92 – 93	94 – 95	≥96			
Any Supplemental Oxygen		Yes		No			
Temperature (°C)	≤35.0		35.1 – 36.0	36.1 – 38.0	38.1 – 39.0	≥39.1	
Systolic Blood Pressure (mm Hg)	≤90	91 – 100	101 – 110	111 – 219			≥220
Heart Rate (beats per minute)	≤40 [b]		41 – 50	51 – 90	91 – 110	111 – 130	≥131
Level of Consciousness				A			V, P, U

Level of consciousness = alert (A), and non-alert and arousable only to voice (V) or pain (P), and unresponsive (U).

[a] *If the participant is on ECMO or invasive mechanical ventilation, they will be given a score of 3 (≤8 breaths per minute) for respiratory rate regardless of ventilator setting.*

[b] *Participants on ECMO will also receive a score of 3 (≤40 beats per minute) for heart rate.*

CONFIDENTIAL

3.2 POWER AND SAMPLE SIZE

3.2

ACTIV-1 is an event-driven trial based on the number of observed recovery events. The effectiveness of each therapeutic agent as add-on therapy to SoC will be evaluated based on the primary endpoint of time to recovery bythrough Day 2928. The Fine-Gray approach will be used to compare each test agent + SoC vs. SoC-alone with respect to the cumulative incidence of recovery, accounting for the semi-competing risk of mortality (Fine and Gray 1999). The approach is similar to using a log-rank test on time to recovery, retaining in the risk set people who die. The two key determinants of power are the total number of events (i.e., recoveries) and the treatment-to-control recovery rate ratio (RRR).

Overall study sample size requirements are based on the number of agents being evaluated and the ability to pool control patients for analysis. Initial sample size estimates are derived assuming three agents are ready for testing at study start and will remain in the study for evaluation at the final analysis stage. If newly emergent therapies are entered into the master protocol after study start, sample size requirements will be adjusted accordingly.

Table 23 provides the numbers of recoveries and of patients required to provide 85% power for a single pairwise comparison of test drug versus control assuming a 73% recovery rate and various RRRs. The assumed 73% recovery rate is based on the Kaplan-Meier recovery rate from ACTT-1. Note that the recovery rate is the analogue of the hazard for each test agent or control treatment, and the RRR is the analogue of the hazard ratio for a test agent relative to control in this setting. Approximately 347 recoveries are required to detect a 40% increase in the rate of recovery ($\theta = 1.40$) from control. An RRR of 1.40 is similar to, but slightly higher than the figure of 1.31 reported in Cao et al. (2020) for a lopinavir/ritonavir trial that used time to improvement by 2 categories as primary endpoint (Cao, Wang et al. 2020). A total of 436 recoveries is needed for an RRR of 1.35 with 85% power.

Table 23. Number of recoveries and number of patients needed for 85% power assuming a type I error rate of 5% for various recovery ratios

Recovery rate ratio (θ)	Number of recoveries needed for 85% power	Sample Size assuming 73% recovery rate (1 test agent vs SoC)
1.25	**788**	**1,080**
1.30	570	781
1.35	436	598
1.40	347	476

The sample size requirements are based on the ability to detect a moderate improvement in time to

29

CONFIDENTIAL

recovery for each agent. After accounting for the proposed interim efficacy and futility monitoring rules,-

CONFIDENTIAL

a total of 788 recoveries are required for each comparison to provide approximately 85% power to detect a RRR of 1.25. Assuming 73% of participants achieve recovery in 28 days, consistent with the ACTT-1 results results (Beigel, Tomashek et al. 2020), the total sample size to evaluate 1, 2, and 3 agents in ACTIV-1 IM is approximately 1080, 1620, and 2160, respectively.

The proposed power and sample size requirements also provide adequate power to detect differences in the key secondary endpoints of clinical improvement and mortality. Based on the formulas from

CONFIDENTIAL

Whitehead (1993), a sample size of 1,080 (540 participants / group)) gives approximately 95% or 85% power to detect a common odds ratio of 1.50 of clinical improvement (the observed odds ratio in the ACTT-1 trial) or 1.40 using a 2-tailed test at level α=0.05. The 28-day mortality probability in the remdesivir arm of ACTT-1 was approximately 12%. The power for comparing 28-day mortality is 90% for a 50% relative reduction (from 12% to 6%) and 80% to detect a difference of 12.0% vs. 6.8%.

3.3 RANDOMIZATION SCHEME

Randomization will be stratified by:

- Geographic Region
  - USA – Northeast
    - Connecticut, Maine, Massachusetts, New Hampshire, New Jersey, New York, Pennsylvania, Rhode Island, Vermont
  - USA - Midwest
    - Illinois, Indiana, Iowa, Kansas, Michigan, Minnesota, Missouri, North Dakota, Nebraska, Ohio, South Dakota, Wisconsin
  - USA – South
    - Alabama, Arkansas, Delaware, District of Columbia, Florida, Georgia, Kentucky, Louisiana, Maryland, Mississippi, North Carolina, Oklahoma, South Carolina, Tennessee, Texas, Virginia, West Virginia
  - USA – West
    - Alaska, Arizona, California, Colorado, Hawaii, Idaho, Montana, New Mexico, Nevada, Oregon, Utah, Wyoming, Washington
  - Argentina
  - Brazil
  - Peru
  - ~~Colombia~~
  - Mexico
  - Additional countries will be treated as separate regions
- Severity of illness at enrollment (by ordinal scale)
  - Severe disease:

CONFIDENTIAL

        - Hospitalized, on invasive mechanical ventilation or ECMO, or
        - Hospitalized, on non-invasive ventilation or high flow oxygen devices.
    - Moderate disease:
        - Hospitalized, requiring supplemental oxygen, or
        - Hospitalized, not requiring supplemental oxygen.

Conceptually, randomization will proceed in two steps. At the first step, each participant will be assigned (open-label) with equal probability to one of the agents available at the time the patient is enrolled and for which the patient is eligible to receive, after applying any agent-specific safety exclusions. At the second step, each participant will be assigned to either the test agent or its matching placebo in an n:1 ratio, where n = the number of agents currently active in the master protocol and for which the patient is eligible to receive.

CONFIDENTIAL

With SoC and three agents active simultaneously, if the participant meets each of the agent-specific criteria, this procedure results in the randomization ratio of 1:1:1:1. If 3 agents are available, but a patient is only eligible to receive 2 of them, the allocation ratio at the first step will be 1:1, and at the second step will be 2:1 (agent vs placebo). Inclusion of a matching placebo for each agent enables masking of study participants and clinical personnel to treatment assignment at the second stage. Data from patients receiving SoC plus placebo will be pooled across agents for comparative analyses and hypothesis testing. Comparative analyses of a particular agent will include the subset of pooled placebo patients who enrolled concurrently with the particular agent and would have been eligible to receive the agent. That is, patients enrolled to control arm prior to a new agent entering the trial will not be included in comparative analyses of that newly added investigation agent.

3.4 INTERIM ANALYSES AND DATA MONITORING

3.4

A complete description of the interim analyses plans including mock tables, listings, and figures is included in a separate DSMB guidance document. Briefly, unblinded interim analyses are planned to (i) assess the futility of each agent, with the goal of discontinuing those with lower probabilities of success to more effectively utilize trial resources for the remaining agents and (ii) review comparative analyses for each test agent to assess early stopping for efficacy.

Alpha spending functions will be used to appropriately control the probability of making an erroneous conclusion across interim and final efficacy analyses at α = 0.05 (two-sided) for each agent. The familywise error rates will be controlled at the two-sided 0.05 level for the primary endpoint and the two key secondary endpoints. Briefly, a gatekeeper approach will be used to test the primary hypothesis. As described in Hung, Wang and O'Neill (2007), the null hypotheses for the two key secondary endpoints are tested only if the null hypothesis is rejected for the primary endpoint. At the second level, the two key secondary endpoints are evaluated using a Pocock-type boundary with a Holm

CONFIDENTIAL

procedure used to control the type I error for the two secondary endpoints (Tamhane, Mehta, Liu 2010; Holm 1979).

3.4.1 INTERIM SAFETY ANALYSES

3.4.1

Safety analyses will evaluate Grade 3 and 4 AE and SAEs by treatment arm. Safety monitoring will be ongoing. The unblinded statistical team will prepare these reports for review by the DSMB. More details will be provided in a separate DSMB guidance document.

3.4.2 INTERIM ANALYSES FOR FUTILITY AND EFFICACY REVIEW

Interim analyses for futility and efficacy arewere planned at three times during the study corresponding to the availability of approximately 25%, 50%, and 75% of total information. The planned randomization algorithm ensures approximately equal allocation to each test agent or control assuming that participants are eligible for all available agents. It is anticipated that the interim analyses for each agent will occur at the same time, and the DSMB will make recommendations for all agents at their scheduled meetings. If recovery rates vary substantially by agent; however, it may be necessary to let the interim analysis times also vary by agent. Because Type I error probabilities are controlled for each agent, independently of the other agents, the need for additional DSMB reviews due to differential information accrual across agents should not pose any issues other than logistical ones.

The Lan-DeMets spending function analog with the O'Brien-Fleming boundaries will be used to monitor the primary endpoint as a guide for the DSMB. This spending function is conservative in that priority is given to preserving power for the final analysis with the use of stringent stopping rules early in the study.

In contrast, moderately aggressive stopping rules for futility will be implemented to promote early discontinuation of agents with low probabilities for success. The futility stopping rules will be considered non-binding by the DSMB in their review of interim data. The futility boundaries are computed based on

CONFIDENTIAL

the gamma family of spending functions (Hwang, Shih, DeCani 1990). **Figure 1** below illustrates the efficacy boundaries based on the Lan-DeMets spending function analog to O'Brien-Fleming and the futility boundaries based on a (Gamma=-2) spending function.

CONFIDENTIAL

Figure 1: Stopping Boundaries for Efficacy (blue shading) and Futility (pink shading).

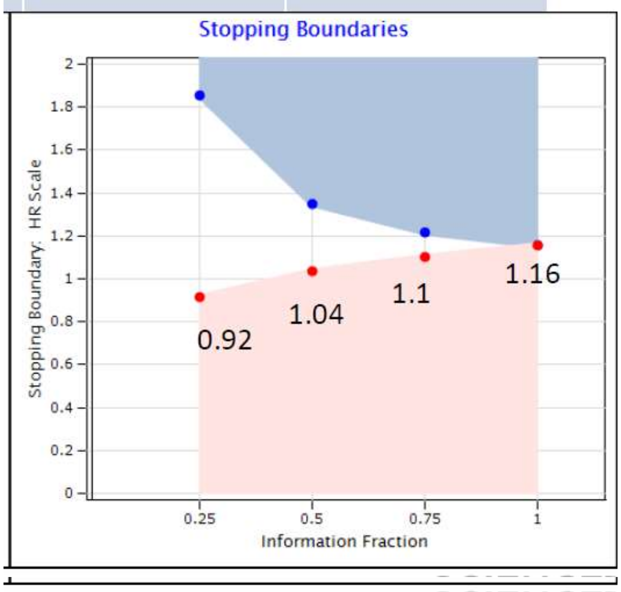

As can be seen from the figure, an RRR showing no more than a small amount of improvement in recovery time (RRR ≤ 1.04) will result in early discontinuation of the agent mid-way through information accrual in the study. (**Table 4**). When the null hypothesis is true (RRR = 1), the probability of discontinuing an agent at this point is 66%, but under the alternative, this probability is 4%.

Table 4. Efficacy and futility boundaries expressed in terms of z-scores and recovery rate ratios (RRRs). Conditional power corresponding to futility boundaries is also shown, and is computed under the originally hypothesized RRR of 1.25. For the interim analyses, conditional power will be calculated under additional scenarios including the current trend and the null hypothesis.

	Efficacy Boundary		Futility Boundary		
Information Fraction	Z-score	RRR	Z-score	RRR	Conditional Power
0.25	4.3326	1.8540	-0.5854	0.9200	0.52
0.50	2.9631	1.3479	0.3791	1.0389	0.40
0.75	2.3590	1.2142	1.2187	1.1055	0.36
1.00	2.0141	1.1543	2.0141	1.1543	NA

If the primary endpoint is declared to be statistically significant in the favorable direction, the two key secondary endpoints will be tested according to a Pocock-like boundary with the Holm procedure used to control for the two key secondary endpoint comparisons (Holm 1979). See **Table 5** for the details of these procedures.

CONFIDENTIAL

Table 5. Efficacy boundaries for the two key secondary endpoints assuming 3 equally spaced interim reviews and a final analysis.

Information Fraction	If the primary endpoint is declared significant, the larger absolute Z-statistic (smaller nominal p-value) is compared against … |Z| (one-sided p-value)	If the primary and other key secondary endpoint is declared statistically significant, the smaller absolute Z-statistic (larger p-value) is compared against … |Z| (one-sided p-value)
0.25	2.62 (0.0043)*	2.36 (0.0091)
0.50	2.62 (0.0043)	2.36 (0.0091)
0.75	2.62 (0.0043)	2.36 (0.0091)
1.00	2.62 (0.0043)	2.36 (0.0091)

*Exact boundaries will be computed using a spending function approach with a Pocock-like bound.

Please note that the DSMB meeting scheduled for the 0.25 information time occurred closer to 0.30 information fraction. Furthermore, the DSMB approval letter dated May 28, 2021 states the following …

"The NIAID DCR Data and Safety Monitoring Board held a teleconference on May 6, 2021. After carefully reviewing the safety data and interim analyses, the DSMB identified no safety concerns and recommended that all sub-studies in ACTIV-1 continue as planned. The DSMB made other recommendations as noted in the summary and recommended that the next DSMB meeting should take placed when ACTIV-1 has reached 60% enrollment."

Following the September 2, 2021 DSMB meeting, the recommendations stated that "the Board recommends that no further interim analysis be done". This meeting was the second DSMB review of the unblinded study data. 1340 participants had been randomized across 70 sites in the U.S. and Latin America at the time of the data cut.

3.4.3 ADAPTATIONS

3.4.3

The master protocol design with common data elements, procedures, etc., and adaptive platform design allows for seamless dropping or replacing of an arm by a new treatment. If the pooled control arm is determined inferior to another arm at an interim analysis, the DSMB will be asked to make a recommendation about possible modifications. Only concurrent data will be used for comparisons. Thus, a new arm will be compared to controls enrolled on or after the time the new arm is added and determined to be eligible to receive the agent. It is anticipated that the DSMB will informally incorporate mortality and adverse event information into the decision making regarding stopping for efficacy and futility.

At the time ofPrior to the second interim analysis, there is a plan to have a blinded sample size re-estimation that allows for sample size increases of as much as 25% but no reduction. This decision would be made by the members of the study team without access to the treatment codes (i.e. active or placebo) or the unblinded results. The formula of Whitehead (1993) was used to determine the expected power for the 8-point scale. That calculation includes a factor that depends on the marginal distribution of the 8-point scale lumped over the active and placebo arms. Additionally, the

CONFIDENTIAL

recommendation for the sample size adjustment will examine the overall recovery rate ~~by day 29.~~through Day 28. It is possible that the observed

~~CONFIDENTIAL~~

recovery rates will be somewhat different from the ACTT-1 results due to changes in SoC, differences in participant mix, and random variability.

Recommendations for the sample size adjustment will be based on trying to maintain ≥ 85% power for the primary endpoint and ≥ 70% power for the key secondary endpoints.

CONFIDENTIAL

Approximately 2 weeks prior to the data cut for the second interim analysis, the blinded statistical team will conduct a review of marginal event rates and endpoint distributions. The goals of the blinded review are to assure that there is adequate power for the primary and two key secondary endpoints under various extreme scenarios. Namely, the team would like to maintain 85% power for the primary endpoint and >70% power for the two key secondary endpoints. The blinded statistics team will have access to limited data including the marginal 28-day mortality rate and the 14-day 8-point ordinal scale lumped across the three sub-studies. As noted in **Table 6** below, the power for these endpoints does depend on these marginal distributions.

Table 6. Outline of the Power Calculations for the Primary and Two Key Secondary Endpoints

**Endpoint**	**Assumptions under the Null Hypothesis** (H_0)	**Assumptions under the Alternative Hypothesis** (H_a)	**Notes**
Time-to-recovery	RRR = 1.0	RRR = 1.25	Each sub-study plans to observe >788 recovery events for the primary endpoint, which provides 85% power under the alternative hypothesis.
8-Point Ordinal Score at 14-days	Common Odds Ratio = 1.00	Common Odds Ratio = 1.50	The power for this statistical comparison is highly dependent on the marginal distribution of the 8-ordered categories. For example, if the placebo group distribution includes 12.5% for each category, a common odds ratio of 1.50 with sample sizes of 540/group will yield approximately 97% power with two-sided Type I error of 0.05. By comparison, a placebo distribution with 10% in category 1, 84% in category 8, and 1% each in categories 2 to 7, would yield only 63% power under the same conditions.
Mortality at 28 days (binary endpoint)	Odds Ratio = 1.00	Odds Ratio < 1.00 (e.g. 50% relative reduction)	12% (placebo) vs. 6% (active) with 540 participants per group will provide 88% power with Type 1 error (two-sided) of 0.025. By comparison, 6% (placebo) vs. 3% (active) will yield the only 55% power under the same conditions.

Tables 7 and 8 provide an example of the power calculations that will be conducted to determine whether there is adequate power to detect 50% reductions in 28-day mortality rates. In the example below there would be adequate power (i.e. >70% power) to detect large relative differences. With a much lower pooled mortality rate, the power would decrease.

CONFIDENTIAL

Table 7. Four scenarios for the treatment mortality rates with marginal mortality rates of 10%.

	**Scenario 1**	**Scenario 2**	**Scenario 3**	**Scenario 4**
	**No effective treatments**	**1 effective treatment**	**2 effective treatments**	**3 effective treatments**
Placebo	10%	11.43%	13.33%	16.00%
Active Drug 1	10%	5.71%	6.67%	8.00%
Active Drug 2	10%	11.43%	6.67%	8.00%
Active Drug 3	10%	11.43%	13.33%	8.00%
Total	10.00%	10.00%	10.00%	10.00%

Table 8. Power calculations to detect 50% relative reductions for the 28-day mortality endpoint.

**Placebo Group Rate**	**Active Group Rate**	**Power with Type I Error of 0.05 (two-sided)**	**Power with Type I Error of 0.025 (two-sided)**
11.43%	5.71%	92%	86%
13.33%	6.67%	95%	92%
16.00%	8.00%	98%	96%

Assuming approximately 540 participants per group

A similar approach will be used with the marginal distribution on the 8-point scale. If these plausible scenarios provide greater than 70% power to detect a common odds ratio of 1.50, the sample size will not be increased.

3.5 DATA SOURCES

3.5

At each study site, data will be entered on the electronic Case Report Forms (eCRFs) stored in Medidata Rave. Prior to database lock, programmed computer edit checks will be run against the database to identify discrepancies and verify reasonableness of the data. Queries to resolve discrepancies will be generated and resolved by the sites. As needed, DCRI Statistics will be able to directly download the eCRF database from Rave using SAS on Demand as Clinical Data Interchange Standards Consortium Study Data Tabulation Model (CDISC SDTM version 3.2) datasets. CDISC Analysis Data Model (ADaM version 2.1) datasets will be created by DCRI Statistics for production of final tables, figures, and listings.

CONFIDENTIAL

All planned reporting will be based off of CDISC datasets, but in the case of emergent safety data, some reporting may occur from the raw eCRF data. All programs written to create analysis datasets and perform analyses will be validated according to SOPs established by the DCRI Statistical Programming group.

3.6 DOCUMENTATION CONVENTION

The statistical analyses described in this SAP, as well as production of tables, listings, and figures will be performed using SAS®, version 9.4 or higher (SAS Institute, Cary, NC). Additional statistical software may be used as needed.

3.7 VERIFICATION OF RESULTS

3.7

All tables, listings, and graphs will be verified and reviewed before considered final. The verification process will ensure that the numbers are produced by a statistically valid method and that the execution of the computations is correct. Qualified statisticians or statistical programmers employed by the DCRI who have not been previously involved in the production of the original programming will perform the verification procedures. Methods of verification include independent programming, prior to issuance of the draft statistical report, of all analysis datasets/ADaM and comparison to data listings. Tables, listings, and figures will be reviewed for accuracy, consistency with this analysis plan, consistency within tables/listings/figures, and consistency with corresponding output. Once verification is complete, all documentation of the verification process will be filed in a statistical programming documentation notebook as required by the Statistical Standard Operations Procedures of the DCRI.

3.8 SUBJECT DISPOSITION

3.8

The disposition of subjects (number randomized, number who received any amount of the randomly assigned treatment, number completing study drug administration, number who withdrew consent or discontinued from study drug early, and number lost to follow-up, and number who completed the trial) will be summarized by treatment group. The number of subjects screened for inclusion and a breakdown of reasons for exclusion will be summarized. The timing and reasons for early discontinuation of study drug and/or withdrawal from the study will be summarized by treatment group. For the calculation of percentages, subjects who die will not be included in the denominators for visits/assessments beyond their death. Treatment compliance (e.g. number of subjects who had their required infusions halted/slowed and the number of subjects with missed doses) will be summarized by-

CONFIDENTIAL

treatment group. A subject listing of analysis population eligibilities will be generated. A listing of all

CONFIDENTIAL

subjects discontinued from the study after enrollment, broken down by site and treatment group will be provided. The listing will include: reason for discontinuation, treatment group, duration of treatment, and whether or not the blind was broken. Also, for subjects who discontinued from the study after enrollment, a listing of adverse events will be provided. Subject-specific protocol deviations will be summarized by the reason for the deviation, the deviation category, treatment group, disease severity and (separately) site for all subjects. All subject-specific protocol deviations and non-subject specific protocol deviations will be included in listings.

3.9 POPULATIONS FOR ANALYSES

3.9

The primary analysis will be based on an intent-to-treat (ITT) population, including all participants randomized. Similarly, safety analyses will be based on a modified intention-to-treat (MITT) population consisting of all participants who received at least one dose or injection of each drug administered in the randomization arm (e.g. investigational agent/placebo plus SoC). Each substudy will define separate ITT and MITT populations due to the shared SoC plus placebo (or control) group. The primary analysis will be based on those participants randomized in order to achieve 788 recoveries for each pairwise comparison. Subsequent analysis will be performed on all randomized participants.

4.0 STATISTICAL ANALYSES

4.1 GENERAL APPROACH

- Statistical significance: Statistical comparisons will be performed using two-sided significance tests. An alpha level of 0.05 will determine significance (unless otherwise noted) for each agent. Analyses will be carried out separately for each test agent.

- Descriptive statistics:
  - Continuous variables will be presented as n, mean, standard deviation, median, Q1, Q3, and minimum and maximum. For comparisons of treatment groups, we will use the non-parametric Wilcoxon/-Mann-Whitney rank-sum test.
  - Categorical variables will be presented as percentage (number). Group comparisons will use the conventional chi-square test.
  - Binary variables will be presented as percentage (number). Group comparisons will use the conventional chi-square test or Fisher's Exact Test.

- When death is not a competing risk (for example, the endpoint includes death in the composite), differences in time-to-event endpoints by treatment will be summarized with Kaplan-Meier curves, log-rank tests, stratified log-rank tests, and hazard ratios from Cox proportional hazards models. When death is a competing risk (for example, time to at least a one-category improvement in ordinal scale, and time to at least a two-category improvement), the same competing risk approach will be used as for the primary analysis and results will be summarized by the hazard ratio from the Fine-Gray model.

- ~~For hazard ratio and odds ratio estimates, Wald confidence intervals will be used. For other efficacy outcomes, Wilson or Score confidence intervals will be used. For safety outcomes, exact (e.g. Clopper-Pearson) confidence intervals will be used.~~

- Covariate adjustment clarification – for some of the models described below, it might be necessary to combine regions and / or disease severity levels to meet modeling assumptions.

- Unblinding: for the final analysis of a study agent:

Unblinding of study data for final analysis will occur for each test agent independently of other

CONFIDENTIAL

agents, consistent with the master protocol design. That is, once the planned number of recoveries required for a particular agent's comparative analysis are observed, study close-out-

CONFIDENTIAL

procedures for that agent are applied for all study visits and data elements associated with the participants receiving the test agent as well as participants receiving SoC plus placebo during the randomization period for that agent (i.e., concurrently controlled participants) subset to those eligible to receive the agent. The

Once the study visits are monitored, data edits are completed, queries are resolved, the database is locked, and treatment assignments are unmasked for that comparative analysis only. Note that, because placebo control participant data are shared across agents, procedures for unmasking data for one agent's final analysis will be established to protect the ability to continue the ongoing study in a double-blinded manner for the remaining agents. It may be that recovery rates are similar enough across all agents in the study to enable a single study close- out with standard procedures. Any necessary modifications or updates to the statistical analysis should be made prior to the study unblinding.

- Handling of Missing Data:

CONFIDENTIAL

Efforts to minimize loss-to-follow-up will be considerable. However, small amounts of missing data may occur. The plans for handling missing data follow the approach taken in the ACTT-1 study (Beigel, Tomashek et al. 2020). All attempts will be made to collect all data per protocol. Any data point that appears to be erroneous or inexplicable based on clinical judgment will be investigated as a possible outlier. If data points are identified as outliers, sensitivity analyses may be performed to examine the impact of including or excluding the outliers. Any substantive differences in these analyses will be reported.

For time to event outcomes, participants who are lost to follow-up or terminate the study prior to Day 2928 and prior to observing/experiencing the event will be censored at the time of their last observed assessment. Participants who die prior to observing/experiencing the event will be censored at Day 29.

For the analyses of the secondary outcomes described in Section 4.3, the following imputation rules will be used for participants who are lost to follow-up, terminate early from the study, or do not have further outcome data available after discharge for any reason:

- Days of Non-invasive ventilation/high-flow oxygen:
  - If the subject's clinical status scale is 3 or 2 at the last observed assessment, then the subject will be considered to be on non-invasive ventilation/high-flow oxygen through-

CONFIDENTIAL

o Day 2928. The endpoint will be total days when assessments are available plus all imputed days following the last observed assessment.

- If the subject is not on non-invasive ventilation/high-flow oxygen at the last observed assessment, then the subject will be considered to not be on non- invasive ventilation/high-flow oxygen for the remainder of follow-up. Thus, no additional imputed days will be added to the number of days recorded on available assessments.

- Days of ventilation/ECMO:
  - If the subject's clinical status scale is 2 at the last observed assessment, then the subject will be considered to be on ventilation/ECMO through Day 2928. The endpoint will be total days when assessments are available plus all imputed days following the last observed assessment.
  - If the subject is not on ventilation/ECMO at the last observed assessment, then the subject will be considered to not be on ventilation/ECMO through Day 2928. Thus, no additional imputed days will be added to the number of days recorded on available assessments.

- Days of Oxygen:
  - If the subject's clinical status score is 4, 3, or 2 at the last observed assessment, then the subject will be considered to be on oxygen through Day 2928. The endpoint will be total days when assessments are available plus all imputed days following the last observed assessment.
  - If the subject is not on oxygen at the last observed assessment, then the subject will be considered to not be on oxygen through Day 2928. Thus, no additional imputed days will be added to the number of days recorded on available assessments.

- Days of Hospitalization

CONFIDENTIAL

- If the subject is discharged and no further hospitalization data are available, then the subject will be assumed to not have been readmitted. Thus, no additional imputed days will be added to the number of days recorded on available assessments. If a subject dies while hospitalized, the number of days of hospitalization will be imputed as 28 days.

- NEVS
  - There will be no imputation for incomplete components of the NEWS score.

CONFIDENTIAL

4.2 ANALYSIS OF THE PRIMARY AND KEY SECONDARY EFFICACY ENDPOINTENDPOINTS

○ Primary Endpoint – Time to Recovery through Day 28

The primary efficacy analysis for the comparison of each test agent plus SoC versus SoC plus placebo is a stratified test based on the Fine-Gray proportional hazards approach, where stratification is according to geographic region and baseline disease severity (i.e. protocol defined moderate vs severe disease).on the 8-point ordinal scale (i.e. 2 or 3 or 4 or 5). Other covariates in the model will include age, and sex. The approach is similar to using a stratified log-rank test on time to recovery, providing an estimate of the cumulative incidence of recovery while accounting for the competing risk of death. The primary quantity of interest, or estimand, is the RRR. With no censoring, the hazard rate in each arm can be thought of as the hazard for recovery, treating deaths as never having recovered (Fine and Gray 1999, Gooley, Leisenring et al. 1999).

The median time to event and 95% confidence interval will be summarized by treatment arm. Cumulative incidence curves for each treatment arm will be presented, supplemented with the RRR estimate, p-value, andalong with the number of subjects at risk in each arm at Days 1, 3, 50 (baseline), 2, 4, 7, 11, 15, 2210, 14, 21, and 2928.

○• Sensitivity Analyses

The primary Gray's test on time to recovery will be calculated to obtain the p-value with fewer parametric assumptions (Gray 1988).

As a sensitivity analysis, the main analysis of the primary and two key secondary endpoints (described below) will be repeated in the MITT analysis population where subjects who are ineligible at baseline will be censored at enrollment. For all.

• Supplemental Analyses

A supplemental and sensitivity analyses, p- values will be reported and analysis will examine the Day 28 binary response of 'recovered' or 'not recovered' which aligns with the 8-point ordinal score at Day 28. Approximately, 95% of study participants are expected to have complete information on this endpoint.

For individuals with a recovery event but missing 8-point ordinal score at Day 28 (~3% of the study population), a multiple imputation approach will be applied to impute those missing values. For each missing observation, 10 imputed observations will be randomly drawn from the subset of individuals with complete data who had a recovery event.

For individuals without a recovery event but missing 8-point ordinal score at Day 28 (~2% of the study population), a multiple imputation approach will be applied to impute those missing values. For each missing observation, 10 imputed observations will be randomly drawn from the subset of individuals

CONFIDENTIAL

with complete data and the same baseline 8-point ordinal score (i.e. 2 or 3 or 4 or 5). Results from the 10 imputed datasets will be combined using Rubin's rule to obtain the point estimate, 95% confidence levels will be used for confidence interval, and p-value.

CONFIDENTIAL

Those individuals in categories 6-8 would be considered as recovered and those in categories 1-5 are 'not recovered'. The point estimate will be based on a difference in the probability estimates. The tabular for active vs. control and graphical summaries described in the previous section95% CI will be replicatedconstructed (and p-value obtained) using the Wald method.

Additionally, for the MITT analysiseach participant, a count of the number of days recovered and out-of-hospital will be computed. The treatment effect will be summarized by the mean difference between groups. This outcome will be analyzed using a linear model with adjustment for geographic region and baseline disease severity on the 8-point ordinal scale (i.e. 2 or 3 or 4 or 5) and an indicator for active vs. placebo treatment. An additional supplemental analysis will analyze this outcome variable using a Wilcoxon- Mann-Whitney test.

The primary analysis will also be repeated using the other subgroups defined in Section 4.7. Each subgroup will be considered separately and the tabular and graphical summaries described in the previous section will be replicated for each subgroup. In addition, a forest plot will be generated to display the overall treatment hazard ratio (or RRR) estimate and CI from each of the within-stratum analyses. These analyses will be performed in the ITT and MITT populations.

Sensitivity analyses will be performed to explore subjects who recover but subsequently report a clinical score < 6. The specific analytic procedures used to explore the subjects will depend on the number of subjects who experience this scenario at the timing of their increase in score. Analyses may include repeating the above analyses, treating such subjects as not recovered or excluding such subjects, in addition to reporting rates of increased score post-recovery.

Analyses that take into account concomitant medication will be performed. The primary analysis will be repeated, where subjects who take prohibited medications will be treated as treatment failures and will be censored at the time of medication use. Other censoring techniques and additional analyses may be performed.

CONFIDENTIAL

A very important sensitivity analysis in the ACTT trials treated recoveries followed by death as a failure to recover. Based on the prior ACTT results we expect fewer than 1% of participants to have this outcome during the 29 days of follow-up. Finally, a set of Bayesian analyses will be conducted for the primary endpoint using optimistic, neutral, and skeptical prior as described in Wijeysundera et al. (2009).

- **Key Secondary Endpoint #1 - Clinical status (8-point ordinal scale) at Day 14**

4.3 ANALYSIS OF THE SECONDARY EFFICACY ENDPOINT(S)

The ordinal scale will be used to estimate a proportional odds model by disease strata and region. We will perform a covariate adjusted test to evaluate whether the common odds ratio for treatment is equal to one. The model covariates will include baseline severity (2, 3, 4, or 5), region, sex, and age (continuous). The distribution of severity results will be summarized by treatment group as percentages. The treatment odds ratio, 95% CI, and p-value estimated from the model will be presented. Efforts to minimize loss-to-follow-up will be considerable.

Supplemental analyses will examine the clinical status using the 8-point ordinal scale at Day 28. This endpoint is expected to be bimodal with individuals more likely to be at the extremes of the scale compared to the Day 14 version.

Analyses of the Day 14 and Day 28 clinical status score will be conducted using multiple imputation. The ~2% of participants who recovered prior to Day 14 but have missing data for the 8-point ordinal scale collected at Day 14 will have 10 observations imputed using randomly selected data from other participants who had complete data for their clinical status (8-point ordinal scale) at Day 14 and a recovery event prior to Day 14.

Participants without a recovery and with missing data for the 8-point ordinal scale at Day 14 (~2% of participants) will have 10 imputed values randomly drawn from the subset of individuals with complete data and the same baseline 8-point ordinal scale. A similar approach will be taken for the Day 28 clinical status score. Results from the 10 imputed datasets will be combined using Rubin's rule.

The number and proportion of subjects along with 95% confidence intervals by category of clinical status will be presented by treatment arm at Days 1, 3, 5, 8, 11, 150 (baseline), 2, 4, 7, 10, 14, 21, and 2928. A figure will present stacked bar charts by day with side by side bars for each treatment arm. Histograms will be generated to display the ordinal scale value distributions over time in each treatment group.

Change in clinical status scale from baseline at specific time points will be summarized by proportions (e.g., proportion who have

CONFIDENTIAL

o **Key Secondary Endpoint #2 - Mortality through Day 28**

Mortality through Day 28 will be analyzed as a 1-, 2-, 3-, or 4-point improvement or 1-, 2-, 3-, 4-point worsening). binary endpoint, using a logistic regression model with an indicator variable for the treatment group. Other variables in the regression modelA table will presentinclude the proportion of people on Days8- point ordinal scale at baseline (2, 3, 4, or 5, 8, 11, 15), region, sex, and 29 within each category of change by treatment armage (continuous). The odds ratio from this model will be supplemented with event rates at 14 and 28-days along with a difference between treatment groups and 95% confidence intervals. The difference in proportions between treatment arms along with 95% confidence interval will also be reported. Additionally, side-by-side histograms (of an agent versus control) of the proportions with various changes will be presented.

(and p-values) obtained using the Wald method. The number and percentage of subjects who die within 14- and 28--days following randomization will be presented by treatment arm (denominator for the percentages will be the number of subjects in the MITT population in each treatment arm). The 14- and 28-day mortality rate, which will take into account the amount of follow-up time for each subject will be calculated and presented. Mortality through Day 29 will also be analyzed as a time to event endpoint, similar to the analysis of the primary endpoint. A table will present median time to event along with 95% confidence intervals overall for each treatment arm along with the hazard ratio estimate and log rank p-values. Differences in time-to-event endpoints by treatment will be summarized with Kaplan-Meier curves.ITT population in each treatment arm).

Approximately, 95% of study participants are expected to have complete information on this endpoint.

For individuals with a recovery event but missing mortality status at Day 28 (~3% of the study population), a multiple imputation approach will be applied to impute those missing values. For each missing observation, 10 imputed observations will be randomly drawn from the subset of individuals with complete data who had a recovery event.

For individuals without a recovery event but missing mortality status at Day 28 (~2% of the study population), a multiple imputation approach will be applied to impute those missing values. For each missing observation, 10 imputed observations will be randomly drawn from the subset of individuals with complete data and the same 8-point ordinal scale baseline score.

Results from the 10 imputed datasets will be combined using Rubin's rule to obtain the point estimate, 95% confidence interval, and p-value.

o **Tipping Point Sensitivity Analyses for the Primary and Key Secondary Endpoints**

Tipping point sensitivity analyses that systematically and comprehensively vary assumptions about the

CONFIDENTIAL

missing outcomes on the two treatment arms will be applied. These analyses will be two-dimensional and will allow assumptions about the missing outcomes on the two arms to vary independently. These scenarios will include those where dropouts on active drug tend to have worse outcomes than dropouts on placebo. The goal is to explore the plausibility of missing data assumptions under which the conclusions change, i.e., under which there is no longer evidence of efficacy.

Among randomized participants, approximately 5% or participants have missing data for the primary or the two key secondary endpoints (**Table 9**). There are two distinct types of missing data for these endpoints:

1) participants with almost no information (n~42) and

CONFIDENTIAL

2) those with missing data following a recovery event (n~60).

Among the 1308 randomized participants with a recovery event prior to Day 14, the vast majority (~98%) had complete data for the ordinal score at Day 14 and approximately 99% of those individuals had a score of 7 or 8. Similarly, among the 1527 randomized participants with a recovery event prior to Day 28, only 8 (<1%) died prior to Day 28.

Table 9. Summary of Missing Data by Type for the Primary and Two Key Secondary Endpoints (data as of March 28, 2022)

Endpoint	Total Participants with Missing Data (N=1971)	Post-Recovery Missing Data (N=1971)	Missing Data Prior to a Recovery (N=1971)
Primary Endpoint – Time to Recovery through Day 28	42* (2.1%)	n/a	42 (2.1%)
Key Secondary Endpoint #1 - Clinical status (8-point ordinal scale) at Day 14	77 (3.9%)	35 (1.8%)	42 (2.1%)
Key Secondary Endpoint #2 - Mortality through Day 28	102 (5.2%)	60** (3.0%)	42 (2.1%)

* 5 (11.4%) participants had > 3 days of follow-up and 11 (25%) received any of their randomized study drug
**of the 1527 participants who recovered prior to Day 28, 8 (<1%) were determined to have died prior by Day 28.

To create an imputation strategy that maintains consistency between the primary and two key secondary endpoints, we will first impute the 'mortality status at day 28' endpoint, then the '8-point ordinal scale at 14 days', and finally, the 'time-to-recovery through day 28' primary endpoint. This structure will help us avoid illogical scenarios such as having the same participant with the following imputed values …

- Death at Day 6
- Recovery at Day 10
- Day 14 8-point ordinal scale of 2 (hospitalized, on invasive mechanical ventilation or ECMO)

Clearly, a participant who dies on Day 6 cannot have a recovery on Day 10.

An ordinal logistic regression model will be fitted to the entire ACTIV1 cohort of randomized participants. The outcome variable will be the participant's status at Day 28 and will have three levels. The worst value of the outcome is death, the best value of the outcome is recovery prior to Day 28, and the intermediate value is for those participants who are alive without a recovery by Day 28. The logistic regression imputation model will include covariates for region and severity of illness ordinal score at the time of randomization. A parameter, ϴ, will vary from 0.1 to 10 and correspond to an odds ratio parameter for the ordinal logistic model. Values of ϴ less than 1.0 will be associated with better than average outcomes and values great than 1.0 will have worse than average outcomes. Specifically, for each level of the ordered response, the value of log(ϴ) will be added to the logit function with the covariates associated with the participants with missing data. Then a random uniform variable will be

CONFIDENTIAL

generated and compared to the imputed probability distribution. With the imputed outcome of either 'death', 'recovery', or 'alive without recovery', the time-to-recovery and the Day 14 8-point ordinal scale response will be imputed by selecting a participant at random with the same severity of illness at randomization, region, and corresponding status at Day 28 (e.g. 'death', 'recovery', or 'alive without recovery').

On average, a value of ϴ = 0.1 will correspond to an expected Day 28 mortality of < 1% whereas a value of ϴ = 10 will correspond to an expected Day 28 mortality of ~ 49% (see **Table 10**). As special cases, values are ϴ = 0 and ϴ = ∞ will be used to denote the special cases of 'best possible outcome' and 'worst possible outcome', respectively. As an example, for a participant who withdrew on Day 3, the best possible outcome (i.e. ϴ = 0) would correspond to: Recovery at 3, Day 14 8-point ordinal scale of 8, and Alive at Day 28. Similarly, for a participant who withdrew on Day 3, the worst possible outcome (i.e. ϴ = ∞) would correspond to: Death at Day 3 and Day 14 8-point ordinal scale of 1.

Table 10. Probability, on average, of recovery and death by Day 28 for various parameter settings

Parameter Setting	Recovery through Day 28	Death through Day 28
ϴ = 0 "Best Case"	100%	0%
ϴ = 0.1	98%*	1%
ϴ = 0.2	95%	2%
ϴ = 0.5	90%	6%
ϴ = 1 "Missing at Random"	82%	10%
ϴ = 2	71%	18%
ϴ = 5	51%	34%
ϴ = 10	35%	49%
ϴ = ∞ "Worst Case"	0%	100%

*the exact values will depend on the participant's covariates

For each parameter setting, 10 imputed datasets will be constructed for each individual with missing data for the primary endpoint. For each imputed dataset, the primary and two key secondary analyses will be re-run and the results of the 10 models will be combined using Rubin's rules. The results of the tipping point analysis will allow for a comparison of point estimates and p-values across the various values of ϴ (see **Table 11** for an example).

Table 11 will be repeated for the two key secondary endpoints, and this analysis will be repeated for the comparison of each study drug vs. the corresponding placebo. Separate models will be constructed for participants with incomplete data following a recovery event (e.g. withdrawal of consent after discharge from the hospital).

CONFIDENTIAL

Table 11. Example summary of the tipping point analysis for the time-to-recovery endpoint

RRR*<br>95% CI<br>p-value						Active				
		ϴ = 0	ϴ = 0.1	ϴ = 0.2	ϴ = 0.5	ϴ = 1.0	ϴ = 2	ϴ = 5	ϴ = 10	ϴ = ∞
	ϴ=0									
	ϴ=0.1									
	ϴ=0.2									
	ϴ=0.5									
Placebo	ϴ=1									
	ϴ=2									
	ϴ=5									
	ϴ=10									
	ϴ=∞									

*the values within each cell will correspond to the estimated RRR, 95% CI, and p-value for the particular parameter setting (e.g. $ϴ_{Placebo}$=1, $ϴ_{Active}$=10).

The above description of the tipping point analysis has focused on the missing data of the subset of participants with no recovery. For the two key secondary endpoints, it is expected that 2-3% of participants will have some missing data following a recovery event.

For individuals with a recovery event but missing mortality status at Day 28 (~3% of the study population) or the Day 14 clinical status score, a multiple imputation approach will be applied to impute those missing values. For each missing observation, 10 imputed observations will be randomly drawn from the subset of individuals with complete data who had a recovery event, and the imputed datasets will be combined as described above.

4.3 ANALYSIS OF THE OTHER SECONDARY EFFICACY ENDPOINTS

- Time to improvement of one and two categories in ordinal scale

Two separate endpoints are considered here. The first endpoint will examine the time to a one category improvement in the 8-point ordinal scale. The second endpoint will examine the time to a two category improvement in the 8-point ordinal scale. These time-to-event endpoints will be computed through Day 28 will also be analyzed using the same Fine-Gray proportional hazards approach as for the primary efficacy endpoint and stratification using the randomization factors. Proportions of one and two category improvements at 14 and 28-days along with 95% confidence intervals overall for each treatment arm along with a difference between treatment arms and 95% confidence intervals obtained

CONFIDENTIAL

using the Miettinen-Nurminen method. Calculations of these endpoints will rely on the 8-point ordinal scale that is collected on a daily basis while the participant is hospitalized.

- Mean change in ordinal scale at specific time points

Change in clinical status scale from baseline at specific time points will be summarized by proportions (e.g., proportion who have a 1-, 2-, 3-, or 4+ point improvement or 1-, 2-, 3-, 4-point worsening). A table will present the proportion of people on Days 2, 4, 7, 10, 14, 21, and 28 within each category of change by treatment arm along with 95% confidence intervals. The mean change in the ordinal scale difference between treatment arms along with 95% confidence interval will also be reported. Additionally, side-by-side histograms (of an agent versus control) of the proportions with various changes will be presented.

- Differences in total severity score (TSS) over time

Differences in total severity score (TSS) over time will assessed by fitting a longitudinal mixedMarkov model to a modified version of the 8-point clinical scale value on each day of assessment (in hospital plus Days 15, 22, 29).a daily basis between days 1 and 28. The average difference across days in study (analogous to the area under the severity curve) will be estimated with 95% confidence limits in the context of the longitudinal model. The longitudinal mixed model will be developed as follows. Differences in total severity score (TSS) over time will be assessed by fitting a longitudinal mixed effects model to the 8-point clinical scale calculated daily from randomization until the day 29 assessment. The average of the 8-point scale over days 1 to 29 (analogous to the area under the severity curve) will be estimated by treatment group with 95% confidence limits in the context of the longitudinal model. The value of the ordinal scale on day t will be assumed to depend on the

CONFIDENTIAL

patient's prior outcome trajectory only through the prior day's value, and treatment group assignment, and an unobserved patient-specific random intercept. The general form of the model will be:

$$\Pr(Y_{it} \le k \mid H_{i,t-1}, Y_{i,t-1} = x, Z_i = z, \boldsymbol{\alpha}, \boldsymbol{\beta}, \boldsymbol{\gamma}, e_i) \equiv \pi_{itk|xz},$$

$$\log \frac{\pi_{itk|xz}}{1-\pi_{itk|xz}} = \alpha_{kx} + t\beta_1 + t^2\beta_2 + z(\gamma_0 + t\gamma_1 + t^2\gamma_2) + e_i,$$

$$e_i \sim N(0, \sigma^2),$$

$$= \alpha_{kx} + t\beta_1 + t^2\beta_2 + z(\gamma_0 + t\gamma_1 + t^2\gamma_2),$$

where Y_{it} is outcome of the i-th patient on day t, $H_{i,t-1} = (Y_{i1}, \dots, Y_{i,t-1})$ is the patient's outcome trajectory through day $t-1$, Z_i is a binary indicator of treatment group assignment (1=active agent +plus SoC, 0=placebo +plus SoC), e_i is an unobserved patient-specific random intercept, and the symbols $\alpha_{kx}, \beta_1, \beta_2, \gamma_0, \gamma_1 \gamma_2, \sigma^2$ represent unknown fixed effect parameters to be estimated from the data. The model assumes that on each day, the odds of an outcome in one of the k lowest categories depends jointly on treatment assignment, days since randomization, and x is the patient's prior day outcome category. Parameters will be estimated by maximum likelihood using 1 record per patient per day from the first day post-randomization until the earlier of day 28, death or loss to follow-up. Days between death and day 28 do not contribute to the likelihood because death is an absorbing state and hence $\alpha_{11} = \infty$ and $\alpha_{21} = \alpha_{31} \cdots = \alpha_{81} = -\infty$. The estimation procedure will assume that loss to follow-up is conditionally ignorable given treatment assignment and the patient's current observed outcome trajectory. A feature of the trial's outcome assessment schedule is that only patients who are hospitalized have their outcome assessed on days-

other than 8, 11, 15, 227, 10, 14, 21, and 2928. If a patient is not hospitalized, the patient's outcome category will be at least 7 but the exact value (7 versus 8) will only be collected on days 8, 11, 15, 227, 10, 14, 21, and 2928. To mitigate potential bias from informatively missing data, categories 7 and 8 will be combined into a single category for this analysis. This modification guarantees that a patient will have non-missing data on all days 1 through 28 if the patient's vital status and hospitalization status is known on each day and the patient's outcome is assessed per protocol on each day that the patient is alive and in-hospital. Additional considerations for this analysis will be detailed in a later version of the Statistical Analysis Plan.

Duration

- Days alive and free of supplemental oxygen

Days alive and free of supplemental oxygen and its components, duration of oxygenation days and total days alive, will be summarized in a table using mean (median) and standard deviations (quartiles) by

CONFIDENTIAL

treatment arm. Point estimates for the difference between active and placebo for days alive and free of supplemental oxygen and associated 95% confidence intervals will be obtained using a linear model with treatment group and baseline severity8-point ordinal score (i.e. 2 or 3 or 4 or 5) as a categorical predictor. Duration of hospitalization days, duration of non-invasive ventilation/high flow oxygen days, and predictors. The duration of invasive Mechanical Ventilation/ECMOoxygenation days will be analyzedcomputed using a similar approachthe 8-point ordinal scale that is collected each day the participant is hospitalized. If a participant is rehospitalized after an initial recovery and the 8-point ordinal scale is not available, the participant will be assumed to have an 8-point ordinal score value of 2 for all days of the rehospitalization.

The incidence of new oxygen use will be analyzed by treatment arm. This will only include subjects in category 4 at enrollment. New use will be identified by a post-enrollment score of at least 5. The number and percent of subjects reporting new use and 95% confidence interval will be reported. Comparisons between treatment arms will be presented as differences in proportions with 95% confidence intervals.

The incidence of new Non-Invasive Ventilation/High-Flow Oxygen use will be analyzed by treatment arm. This will only include subjects in category 4 or 5 at enrollment. The incidence of new oxygen use will be analyzed by treatment arm. This will only include subjects in ordinal scale category 45 at enrollment. (i.e. 5: Hospitalized, not requiring supplemental oxygen – requiring ongoing medical in-patient care). New use will be identified by a post-enrollment score of at least 64 or 3 or 2. The number and percent of subjects reporting new use and the 95% confidence interval will be reported. Comparisons between treatment arms will be presented as differences in proportions with 95% confidence intervals using the Miettinen-Nurminen method.

CONFIDENTIAL

- Days alive and free of non-invasive ventilation/high flow oxygen

Days alive and free of non-invasive ventilation/high flow oxygen and its components, duration of non-invasive ventilation/high flow oxygen days will be summarized in a table using mean (median) and standard deviations (quartiles) by treatment arm. Point estimates and associated 95% confidence intervals will be obtained using a linear model with treatment group and baseline 8-point ordinal score (i.e. 2 or 3 or 4 or 5) as categorical predictors. The duration of non-invasive ventilation/high flow oxygen days will be computed using the 8-point ordinal scale that is collected each day the participant is hospitalized. If a participant is rehospitalized after an initial recovery and the 8-point ordinal scale is not available, the participant will be assumed to have an 8-point ordinal score value of 2 for all days of the rehospitalization.

The incidence of new Non-Invasive Ventilation/High- Flow Oxygen use will be analyzed by treatment arm. This will only include subjects in category 4 or 5 at enrollment. ThisNew use will only include subjects in category 4, 5, or 6 at be identified by a post-

CONFIDENTIAL

enrollment.- score of 3 or 2. Comparisons between treatment arms will be presented as differences in proportions with 95% confidence intervals using the Miettinen-Nurminen method.

- Days alive and free of invasive mechanical ventilation/ECMO

Days alive and free of invasive mechanical ventilation / ECMO and its components, total days alive and duration of invasive mechanical ventilation/ECMO days, will be summarized in a table using mean (median) and standard deviations (quartiles) by treatment arm. Point estimates for differences in the days alive and free of invasive mechanical ventilation / ECMO and associated 95% confidence intervals will be obtained using a linear model with treatment group and baseline 8-point ordinal score (i.e. 2 or 3 or 4 or 5) as categorical predictors. The duration of invasive mechanical ventilation/ECMO days will be computed using the 8-point ordinal scale that is collected each day the participant is hospitalized. If a participant is rehospitalized after an initial recovery and the 8-point ordinal scale is not available, the participant will be assumed to have an 8-point ordinal score value of 2 for all days of the rehospitalization.

The incidence of new oxygenInvasive Mechanical Ventilation/ECMO use will be analyzed by treatment arm. This will only include subjects in category 5, 4, or 3 at enrollment. New use will be identified by a post- enrollment score of 72. The number and percent of subjects reporting new use and the 95% confidence interval will be reported. Comparisons between treatment arms will be presented as differences in proportions with 95% confidence intervals using the Miettinen-Nurminen method.

- Days alive and out of hospitalization

Days alive and out of hospital along with its components, duration of hospitalizations and total days alive, will be summarized in a table using mean (median) and standard deviations (quartiles) by treatment arm. Point estimates for the difference between active and placebo agents for days alive and out of hospital and associated 95% confidence intervals will be obtained using a linear model with treatment group and baseline 8-point ordinal score (i.e. 2 or 3 or 4 or 5) as categorical predictors.

4.4 SAFETY ANALYSES

4.4

Safety endpoints include death through Day 28 (as assessed at Day 29), SAEs, and Grade 3 and 4 AEs. These events will be analyzed individually and as a composite endpoint. The time to this composite endpoint will be defined as the elapsed time (in days) from Day 1baseline to the earliest date of any of the events. Any subjects that are lost to follow-up or terminated early prior to experiencing any of the events will be censored at the day of their last observed assessment. Subjects who complete follow-up-

CONFIDENTIAL

but do not experience any of the events will be censored at the day of their Day 29 visit. Time-to-event methods will be used for death and the composite endpoint. A table will present the hazard ratio estimate with 95% confidence intervals and log rank p-values. Differences in time-to-event endpoints by treatment will be summarized with cumulative incidence curves. In addition, risk differences between treatment groups and 95% CIs using the Miettinen-Nurminen method will be estimated for each type of event.

Similar analyses will be conducted for the following AEs:

- Any Grade 2 suspected drug-related hypersensitivity reactions associated with study product administration will be reported as an AE.
- Any newly diagnosed infection (other than COVID-19) at any time during the study.
- Any AEs leading to dose modification
- Any AEs leading to discontinuation from the study.

AEs will be coded in accordance with the current version of the Medical Dictionary for Regulatory Activities (MedDRA). AEs will be graded with regard to severity according to criteria defined in the Common Terminology Criteria for Adverse Events version 5.0 (CTCAE) and relationship to COVID-19 or study intervention. Each AE will be counted once only for a given participant. AEs will be presented by system organ class, preferred term (PT), duration (in days), start- and stop-date. AEs leading to premature discontinuation from the study intervention and serious AEs will be presented in a listing.

4.4.1 LABORATORY DATA

Clinical safety laboratory adverse events are collected Day 1, 3, 5, 8, 11 and Day 15 and 29 if able to return to clinic or still hospitalized. Parameters evaluated include white blood cell count, differential, Hgb, PLT, creatinine, glucose, total bilirubin, AST, ALT, ALP, PT/INR, d-dimer, cardiac troponin, and C-reactive protein. Laboratory safety parameters will be graded according to the Division of AIDS (DAIDS) Table for Grading the Severity of Adult and Pediatric Adverse Events, version 2.1 (July 2017). The distribution of abnormal chemistry and hematology laboratory results by maximum severity, time point, and treatment group will be presented. In addition, the distribution of Grade 3 and 4 chemistry and hematology laboratory results by maximum severity, time point, disease severity and treatment group will be presented. Descriptive statistics including mean, median, standard deviation, maximum, and minimum values and change from baseline by time point, for all and each chemistry and hematology laboratory parameter will be summarized by disease severity and treatment arm. Changes in chemistry or INR, d-dimer, cardiac troponin, and C- reactive protein.

CONFIDENTIAL

and hematology laboratory values will be presented in line graphs over time with mean and SD plotted by disease severity and treatment arm.

Descriptive statistics for baseline and post-baseline time points will be generated for abnormal laboratory data (hematology and chemistry values).. Additionally, shift-
tables will be presented. The shift tables tabulate the number of lab values determined to be “Normal” and “Abnormal” at baseline and post-baseline time points. The classifications on shift tables will-categorize lab values as “Normal” when they are in acceptable range, using a combination of clinician judgment specific to each lab test, and the reference ranges.. Laboratory values outside the normal range will be graded on the Labs Listing as >1x, >2x, >3x, >5x, or >10x the upper limit (or <0.1x, <0.5x, <1x the lower limit) of normal value based on appropriate increases or decreases. Laboratory data will also be summarized graphically to show the magnitude and changes in individual subject values over time relative to normal ranges. Details of any abnormalities will be listed.

CONFIDENTIAL

4.4.2 VITAL SIGNS

Vital sign measurements include pulse, systolic blood pressure, respiratory rate, SpO2 and oral temperature. Vital signs will be presented in a listing.

4.4.3 PRIOR AND COMCOMITANT MEDICATIONS

Concomitant medications will be coded to the Anatomical Therapeutic Classification using the WHO Drug Dictionary. The use of prior, concomitant medications taken, and changes in concomitant medications during the study will be recorded on the CRFs. The use of concomitant medications during the study will be summarized by ATC1, ATC2 code, disease severity, and treatment group for the MITTITT population. The summaries will be repeated for each test agent.

4.4.4 TREATMENT COMPLIANCE

Treatment compliance will be assessed based upon the administration rules for each of the test agents. Details for each test agent will be provided in an addendumFor treatments that are one-time infusions (e.g. infliximab or abatacept), compliance will be summarized by the percentage of participants starting the infusion, the mean duration of the infusion, and the total amount of drug received (mg). For oral medications (e.g. cenicriviroc), compliance will be summarized by the percentage of participants taking the loading dose and starting the maintenance dose, mean number of maintenance doses, and mean duration (days) of the oral medication.

4.4.5 PREGNANCY TESTS

Pregnancy tests for females of childbearing potential are performed as part of screening laboratory test on Day -1/Day 1. For any female participants in the MITT population who become pregnant during the study, every attempt will be made to follow these participants to completion of pregnancy to document the outcome, including information regarding any complications with pregnancy and/or delivery. A set of listings of pregnancies and outcomes will be presented.

A set of listings of pregnancies and outcomes will be presented.

4.5 EXPLORATORY ANALYSES

An exploratory analysis will compare treatment efficacy estimates for each agent for the primary endpoint and the

two key secondary endpoints. Specifically, the

The probability of falling into category "i" or better will be compared between arms for each baseline severity score category. Exploratory analyses will also compare the difference between each test agent plus SoC and SoC plus placebo in the 8-point ordinal scale at each study day for which the clinical scale is administered (in-hospital and at Days 14, 21, and 28).

CONFIDENTIAL

will also compare the difference between each test agent plus SoC and SoC plus placebo in TSS at each study day for which the clinical scale is administered (in-hospital and at Days 15, 22, and 29).

A set of Bayesian analyses will be conducted for the primary endpoint using informative, enthusiastic, and skeptical priors as described in Wijeysundera et al. (2009) and Zampieri et al. (2021). Three different prior distributions will be assumed for the odds ratio parameter for the treatment effect in the proportional odds model. For the informative prior distribution, the log odds ratio (active drug vs. placebo) will have a mean of 0 and a standard deviation of 10 on the log-odds scale. For the skeptical prior, the log odds ratio will have a mean of 0 and a standard deviation for the log-odds ratio that provides 5% probability for a response that is better than the assumed alternative hypothesis (e.g. OR=1.25). For the enthusiastic prior, the assumed log-odds ratio will use the treatment effect assumed for the estimation of the sample size (e.g. OR~1.25) and a standard deviation that provides 5% probability of no benefit (e.g. OR<=1.00). Similar analyses will be conducted for the 28-day mortality binary endpoint. For these analyses, the enthusiastic prior will correspond to an odds ratio < 1.0 indicating treatment benefit for the active drug.

A separate statistical analysis plan will be created for the analyses of biomarkers.

4.6 BASELINE DESCRIPTIVE STATISTICS

Baseline characteristics will be summarized by test agent versus control for each agent and study population. For continuous measures the mean and standard deviation will be summarized. Categorical variables will be described by the proportion in each category (with the corresponding sample size- numbers).).

Baseline characteristics will include age, sex, race, ethnicity, symptom duration, hypertension, obesity, diabetes, coronary disease, history of heart failure, history of cancer, asthma, COPD, chronic pulmonary disease, tuberculosis, HIV, severe liver disease, severe kidney disease, respiratory support received, days since hospitalization, and baseline 8-point ordinal score.

4.7 SUB-GROUP ANALYSES

The following subgroups have been pre-specified for the primary outcome of time to recovery and the two key secondary outcomes:

- Geographic region
- Duration of symptoms prior to randomization (<= Median; > Median)
- Duration of symptoms prior to randomization (based on quartiles)
- Hospitalization duration prior to randomization (<= Median, > Median)

CONFIDENTIAL

- Baseline disease severity based on the 8-point ordinal scale

CONFIDENTIAL

- Age (<40; 40-64; 65 and older)
- Race (White; Black/African American; Asian; Other)
- Ethnicity (Hispanic, non-Hispanic)
- Sex (Female; Male)
- Comorbidities: Any0, 1, 2, 3+
- Baseline use of dexamethasone
- Co-enrollment in any ACTIV trial prior to randomization in ACTIV-1 IM
- Individual comorbidities: hypertension, obesity, diabetes, coronary disease, history of heart failure, history of cancer, asthma, COPD, chronic pulmonary disease, severe liver disease, severe kidney disease

In addition, Fine-Gray modelsAny subgroup that has a frequency <5% will not be run within each of generated. Subgroups with frequencies <10% may also have issues with model convergence that dictate that they cannot be generated.

The primary analysis will also be repeated for the disease severity strata to obtain stratum- specific estimates ofprimary endpoint and the treatment hazard ratio. A forest plot will display confidence intervals across two key secondary endpoints using the subgroups defined above. Each subgroup will be considered separately and the tabular and graphical summaries described in the previous section will be replicated for each subgroup. In addition, a forest plot will be generated to display the overall treatment hazard ratio (or RRR) estimate and CI from each of the within-stratum analyses. These analyses will be performed in the ITT and MITT populations.

. Interaction tests will be conducted to determine whether the effect of treatment varies by subgroup. These subgroup analyses will be carried out separately for each test agent.

In addition to the subgroup analyses described above, primary and key secondary and safety analyses will be performed within additional subgroups as outlined in **Appendix 2**.

CONFIDENTIAL

4.8 REFERENCES

4.8

Beigel, J. H., K. M. Tomashek, L. E. Dodd, A. K. Mehta, B. S. Zingman, A. C. Kalil, E. Hohmann, H. Y. Chu, A. Luetkemeyer, S. Kline, D. Lopez de Castilla, R. W. Finberg, K. Dierberg, V. Tapson, L. Hsieh, T. F. Patterson, R. Paredes, D. A. Sweeney, W. R. Short, G. Touloumi, D. C. Lye, N. Ohmagari, M. D. Oh, G. M. Ruiz-Palacios, T. Benfield, G. Fätkenheuer, M. G. Kortepeter, R. L. Atmar, C. B. Creech, J. Lundgren, A. G. Babiker, S. Pett, J. D. Neaton, T. H. Burgess, T. Bonnett, M. Green, M. Makowski, A. Osinusi, S. Nayak and H. C. Lane (2020). "Remdesivir for the Treatment of Covid-19 - Preliminary Report." N Engl J Med.

Cao, B., Y. Wang, D. Wen, W. Liu, J. Wang, G. Fan, L. Ruan, B. Song, Y. Cai, M. Wei, X. Li, J. Xia, N. Chen, J. Xiang, T. Yu, T. Bai, X. Xie, L. Zhang, C. Li, Y. Yuan, H. Chen, H. Li, H. Huang, S. Tu, F. Gong, Y. Liu, Y. Wei, C. Dong, F. Zhou, X. Gu, J. Xu, Z. Liu, Y. Zhang, H. Li, L. Shang, K. Wang, K. Li, X. Zhou, X. Dong, Z. Qu, S. Lu, X. Hu, S. Ruan, S. Luo, J. Wu, L. Peng, F. Cheng, L. Pan, J. Zou, C. Jia, J. Wang, X. Liu, S. Wang, X. Wu, Q. Ge, J. He, H. Zhan, F. Qiu, L. Guo, C. Huang, T. Jaki, F. G. Hayden, P. W. Horby, D. Zhang and C. Wang (2020). "A Trial of Lopinavir-Ritonavir in Adults Hospitalized with Severe Covid-19." N Engl J Med 382(19): 1787-1799.

Fine, J. P. and R. J. Gray (1999). "A Proportional Hazards Model for the Subdistribution of a Competing Risk." Journal of the American Statistical Association 94(446): 496-509.

Gooley, T. A., W. Leisenring, J. Crowley and B. E. Storer (1999). "Estimation of failure probabilities in the presence of competing risks: new representations of old estimators." Stat Med 18(6): 695-706.

Gray RJ (1988). “A class of K-sample tests for comparing the cumulative incidence of a competing risk.” Ann Stat 16:1141-1154.

Holm SA. (1979) “A simple sequentially rejective multiple test procedure.” Scandanavian Journal of Statistics; 6: 65-70.

Huang, C., Y. Wang, X. Li, L. Ren, J. Zhao, Y. Hu, L. Zhang, G. Fan, J. Xu, X. Gu, Z. Cheng, T. Yu, J. Xia, Y. Wei, W. Wu, X. Xie, W. Yin, H. Li, M. Liu, Y. Xiao, H. Gao, L. Guo, J. Xie, G. Wang, R. Jiang, Z. Gao, Q. Jin, J. Wang and B. Cao (2020). "Clinical features of patients infected with 2019 novel coronavirus in Wuhan, China." Lancet 395(10223): 497-506.

Hung J, Wang S-J, O'Neill R (2007). “Statistical considerations for testing multiple endpoints in group sequential or adaptive clinical trials.” J Biopharm Stat; 17(6):1201-10.

Hwang IK, Shih WJ, J S De Cani JS (1990). “Group sequential designs using a family of type I error probability spending functions” Stat Med; 9(12):1439-45.

Smith, G. B., D. R. Prytherch, S. Jarvis, C. Kovacs, P. Meredith, P. E. Schmidt and J. Briggs (2016). "A Comparison of the Ability of the Physiologic Components of Medical Emergency Team Criteria and the

U.K. National Early Warning Score to Discriminate Patients at Risk of a Range of Adverse Clinical Outcomes." Crit Care Med 44(12): 2171-2181.

Tamhane AC, Mehta CR, Liu L (2010) "Testing a Primary and a Secondary Endpoint in a Group Sequential Design." Biometrics; 66(4): 1174–1184.

Whitehead J. (1993) "Sample Size Calculations for Ordered Categorical Data." Stat Med; vol. 12, 2257-2271.

Wijeysundera DN, Austin PC, Hux JE, Beattie WS, Laupacis A (2009). "Bayesian statistical inference enhances the interpretation of contemporary randomized controlled trials." J Clin Epidemiol; 62: 13-21 e15.

CONFIDENTIAL

Zampieri FG, Casey JD, Shankar-Hari M; Harrell Jr. FE, and Harhay MO (2021). "Using Bayesian Methods to Augment the Interpretation of Critical Care Trials: An Overview of Theory and Example Reanalysis of the Alveolar Recruitment Trial." Am J Resp Crit Care Med. Am J Respir Crit Care Med; 203(5): 543-552.

CONFIDENTIAL

4.9 APPENDICES

APPENDIX 1. ACTIV-1 Roster of Statisticians

Team	Name	Email	Attends DSMB Meetings
Unblinded Statistical Team	Michael Proschan	[redacted]	X
	Tyler Bonnet	[redacted]	X
	Jyotsna Garg	[redacted]	X
	Eric Yow	[redacted]	X
	Zhen Huang	[redacted]	
	Hwasoon Kim	[redacted]	
	Hussein Al-Khalidi	[redacted]	X
	Sean O'Brien	[redacted]	X
Blinded Statistical Team	Lisa LaVange	[redacted]	X
	Steven McNulty	[redacted]	X
	Adam Silverstein	[redacted]	
	Jun Wen	[redacted]	
	Pearl Zakroysky	[redacted]	X
	Susan Halabi	[redacted]	X
	Kevin Anstrom	[redacted]	X

CONFIDENTIAL

~~Statistical Analysis Plan~~

46

APPENDIX 2. Additional Tables and Listings

Table	Demographic and Baseline Characteristics – US	Intent-to-Treat Population
Table	Demographic and Baseline Characteristics – Latin America	Intent-to-Treat Population
Table	Demographic and Baseline Characteristics by Geographic Region - Argentina	Intent-to-Treat Population
Table	Demographic and Baseline Characteristics by Geographic Region - Brazil	Intent-to-Treat Population
Table	Demographic and Baseline Characteristics by Geographic Region - Mexico	Intent-to-Treat Population
Table	Demographic and Baseline Characteristics by Geographic Region - Peru	Intent-to-Treat Population
Table	Demographic and Baseline Characteristics by Geographic Region – USA – Northeast	Intent-to-Treat Population
Table	Demographic and Baseline Characteristics by Geographic Region – USA – Midwest	Intent-to-Treat Population
Table	Demographic and Baseline Characteristics by Geographic Region – USA – South	Intent-to-Treat Population
Table	Demographic and Baseline Characteristics by Geographic Region – USA – West	Intent-to-Treat Population
Table	Demographic and Baseline Characteristics – Moderate Disease Severity at Baseline	Intent-to-Treat Population
Table	Demographic and Baseline Characteristics – Severe Disease Severity at Baseline	Intent-to-Treat Population
Table	Time to Recovery by US vs. Latin America	Intent-to-Treat Population
Table	Time to Recovery by Disease Severity at Baseline (Moderate vs. Severe)	Intent-to-Treat Population
Table	Time to Recovery by Remdesivir Use at Randomization	Intent-to-Treat Population
Table	Time to Recovery by Steroid Use at Randomization	Intent-to-Treat Population
Table	Time to Recovery by Immune Modulator Use at Randomization	Intent-to-Treat Population
Table	Time to Recovery by Anticoagulant Use at Randomization	Intent-to-Treat Population
Table	Time to Recovery by Baseline CRP (<=75 mg/L; >75 mg/L)	Intent-to-Treat Population
Table	Proportional Odds Model of Clinical Status Score at Day 14 by US vs. Latin America	Intent-to-Treat Population
Table	Proportional Odds Model of Clinical Status Score at Day 14 by Disease Severity at Baseline (Moderate vs. Severe)	Intent-to-Treat Populat4io7n

Table	Proportional Odds Model of Clinical Status Score at Day 28 by US vs. Latin America	Intent-to-Treat Population
Table	Proportional Odds Model of Clinical Status Score at Day 28 by Geographic Region	Intent-to-Treat Population
Table	Proportional Odds Model of Clinical Status Score at Day 28 by Disease Severity at Baseline (Moderate vs. Severe)	Intent-to-Treat Population
Table	28-Day Mortality by US vs. Latin America	Intent-to-Treat Population
Table	28-Day Mortality by Disease Severity at Baseline (Moderate vs. Severe)	Intent-to-Treat Population
Table	28-Day Mortality by Remdesivir Use at Randomization	Intent-to-Treat Population
Table	28-Day Mortality by Steroid Use at Randomization	Intent-to-Treat Population
Table	28-Day Mortality by Immune Modulator Use at Randomization	Intent-to-Treat Population
Table	28-Day Mortality by Anticoagulant Use at Randomization	Intent-to-Treat Population
Table	28-Day Mortality by Baseline CRP (<=75 mg/L; >75 mg/L)	Intent-to-Treat Population
Table	14-Day and 28-Day Mortality Rates by US vs. Latin America	Intent-to-Treat Population
Table	14-Day and 28-Day Mortality Rates by Geographic Region	Intent-to-Treat Population
Table	14-Day and 28-Day Mortality Rates by Disease Severity at Baseline (Moderate vs. Severe)	Intent-to-Treat Population
Table	14-Day and 28-Day Mortality Rates by Baseline CRP (<=75 mg/L; >75 mg/L)	Intent-to-Treat Population
Table	Overview of Safety Composite, Individual Endpoints, and Adverse Events through Day 28 - US	Modified Intent-to-Treat Population
Table	Overview of Safety Composite, Individual Endpoints, and Adverse Events through Day 28 – Latin America	Modified Intent-to-Treat Population
Table	Overview of Safety Composite, Individual Endpoints, and Adverse Events through Day 28 by Geographic Region - Argentina	Modified Intent-to-Treat Population
Table	Overview of Safety Composite, Individual Endpoints, and Adverse Events through Day 28 by Geographic Region – Brazil	Modified Intent-to-Treat Population
Table	Overview of Safety Composite, Individual Endpoints, and Adverse Events through Day 28 by Geographic Region – Mexico	Modified Intent-to-Treat Population
Table	Overview of Safety Composite, Individual Endpoints, and Adverse Events through Day 28 by Geographic Region – Peru	Modified Intent-to-Treat Population
Table	Overview of Safety Composite, Individual Endpoints, and Adverse Events	Modified Intent-to-Treat

	through Day 28 by Geographic Region – USA – Northeast	Population
Table	Overview of Safety Composite, Individual Endpoints, and Adverse Events through Day 28 by Geographic Region – USA – Midwest	Modified Intent-to-Treat Population
Table	Overview of Safety Composite, Individual Endpoints, and Adverse Events through Day 28 by Geographic Region – USA – South	Modified Intent-to-Treat Population
Table	Overview of Safety Composite, Individual Endpoints, and Adverse Events through Day 28 by Geographic Region – USA – West	Modified Intent-to-Treat Population
Table	Overview of Safety Composite, Individual Endpoints, and Adverse Events through Day 28 – Moderate Disease Severity at Baseline	Modified Intent-to-Treat Population
Table	Overview of Safety Composite, Individual Endpoints, and Adverse Events through Day 28 – Severe Disease Severity at Baseline	Modified Intent-to-Treat Population
Table	Most Common Adverse Events through Day 28 Occurring in >10% of Infliximab Subjects	Modified Intent-to-Treat Population
Table	Most Common Adverse Events through Day 60 Occurring in >10% of Infliximab Subjects	Modified Intent-to-Treat Population
Listing	Prior Medications	Intent-to-Treat Population
Listing	Concomitant Medications	Intent-to-Treat Population

04_SAP_ACTIV1_IM_~~25NOV202 0~~11APR2022_clean

Final Audit Report ~~2020~~2022-04-12-~~04~~

~~Created:~~	~~2020-12-02~~
~~By:~~	~~Pearl Zakroysky (pz33@duke.edu)~~
~~Status:~~	~~Signed~~
~~Transaction ID:~~	~~CBJCHBCAABAAvllCGJWy11ZJIO7QbAkWAPOjLqEjAL4b~~

~~"Statistical Analysis Plan~~ "04_SAP_ACTIV1_IM_~~25NOV2020~~11APR2022_clean" ~~Histor y~~History

Document created by ~~Pearl Zakroysky (pz33~~rose beci (beci0001@duke.edu)
~~2020-12-02 - 2:21:36~~2022-04-11 - 8:41:01 PM GMT- IP address: ~~152.16.191.126~~136.226.49.3

Document emailed to ~~danny benjamin~~Danny Benjamin (danny.benjamin@duke.edu) for signature
~~2020-12-02 - 2:23:44~~2022-04-11 - 8:41:56 PM GMT

Email viewed by ~~danny benjamin~~Danny Benjamin (danny.benjamin@duke.edu)
~~2020-12-~~2022-04-11 - 10:17:02 ~~- 5:26:20~~ PM GMT- IP address: ~~99.132.141.156~~104.28.33.33

Document e-signed by ~~danny benjamin~~Danny Benjamin (danny.benjamin@duke.edu)
Signature Date: ~~2020-12-02 - 5:26:55~~2022-04-11 - 10:18:13 PM GMT - Time Source: server- IP address: ~~99.132.141.156~~174.216.2.166

Document emailed to ~~Jane C. Atkinson (jatkinso@mail.~~Soju Chang (soju.chang@nih.gov) for signature
~~2020-12-02 - 5:26:57~~2022-04-11 - 10:18:15 PM GMT

Email viewed by ~~Jane C. Atkinson (jatkinso@mail.~~Soju Chang (soju.chang@nih.gov)
~~2020-12-02 - 6:24:07~~2022-04-11 - 10:37:14 PM GMT- IP address: ~~128.231.234.6~~172.225.29.21

Document e-signed by ~~Jane C. Atkinson (jatkinso@mail.~~Soju Chang (soju.chang@nih.gov)
Signature Date: ~~2020~~2022-04-12-~~02 - 6:26:26 PM~~ - 0:01:29 AM GMT - Time Source: server- IP address: 128.231.234.626

Document emailed to Lisa LaVange (lisa.lavange@unc.edu) for signature
~~2020~~2022-04-12-~~02 - 6:26:28 PM~~ - 0:01:31 AM GMT

Email viewed by Lisa LaVange (lisa.lavange@unc.edu)
~~2020~~2022-04-12-~~04~~ - 1:~~36:39 AM~~27:32 PM GMT- IP address: ~~99.69.18.80~~152.19.206.98

Document e-signed by Lisa LaVange (lisa.lavange@unc.edu)

Signature Date: ~~2020~~2022-04-12~~-04~~ - 1:~~39:40 AM~~28:08 PM GMT - Time Source: server- IP address: ~~99.69.18.80~~152.19.206.98

Adobe Sign

Document emailed to Michael Proschan (proscham@niaid.nih.gov) for signature
2020-12-04 - 1:39:43 AM GMT

Email viewed by Michael Proschan (proscham@niaid.nih.gov)
2020-12-04 - 2:21:38 PM GMT- IP address: 128.231.234.67

Document e-signed by Michael Proschan (proscham@niaid.nih.gov)
Signature Date: 2020-12-04 - 3:44:48 PM GMT - Time Source: server- IP address: 128.231.234.67

Document emailed to William Powderly (wpowderly@wustl.edu) for signature
20202022-04-12-04 - 3:44:50 - 1:28:10 PM GMT

Adobe Acrobat Sign

Email viewed by William Powderly (wpowderly@wustl.edu)

20202022-04-12-04 - 3:51:31 - 1:30:52 PM GMT- IP address: 128.252.34.131108.207.150.118

Document e-signed by William Powderly (wpowderly@wustl.edu)

Signature Date: 20202022-04-12-04 - 3:53:30 - 1:31:22 PM GMT - Time Source: server- IP address: 128.252.34.131108.207.150.118

Agreement completed.

20202022-04-12-04 - 3:53:30 - 1:31:22 PM GMT

Adobe Sign

Adobe Acrobat Sign
