# Supplementary material for: Abatacept Pharmacokinetics and Exposure Response in Patients Hospitalized With COVID-19: A Secondary Analysis of the ACTIV-1 IM Randomized Clinical Trial
Source: JAMA Netw Open. 2024 Apr 25;7(4):e247615. doi: 10.1001/jamanetworkopen.2024.7615 (PMC11046337; doi:10.1001/jamanetworkopen.2024.7615)
Supplement: Supplement 2. — eMethods. eResults. eTable. Pharmacokinetic Parameters for the Base Model eFigure 1. CONSORT Diagram eFigure 2. Diagnostic Plots for the Final Abatacept Model eFigure 3. Prediction-Corrected Visual Prediction Check for the Final Population Pharmacokinetic Model eFigure 4. Abatacept Clearance and Baseline Disease Severity eFigure 5. Abatacept Exposure and Probability of Recovery at 28 Days eFigure 6. Target Abatacept Attainment From Dosage Simulations eFigure 7. Sensitivity Analysis for Maximum Abatacept Concentration Using IPRED eFigure 8. Maximum Abatacept Concentration Using DV eReferences. [file jamanetwopen-e247615-s002.pdf]

Supplementary Online Content

Balevic SJ, Benjamin DK Jr, Powderly WG, et al; ACTIV-1 IM Study Group. Abatacept pharmacokinetics and exposure response in patients hospitalized with COVID-19: a secondary analysis of the ACTIV-1 IM randomized clinical trial. *JAMA Netw Open*. 2024;7(4):e247615. doi:10.1001/jamanetworkopen.2024.7615

eMethods.

eResults.

eTable. Pharmacokinetic Parameters for the Base Model

eFigure 1. CONSORT Diagram

eFigure 2. Diagnostic Plots for the Final Abatacept Model

eFigure 3. Prediction-Corrected Visual Predictive Check for the Final Population Pharmacokinetic Model

eFigure 4. Abatacept Clearance and Baseline Disease Severity

eFigure 5. Abatacept Exposure and Probability of Recovery at 28 Days

eFigure 6. Target Abatacept Attainment From Dosage Simulations

eFigure 7. Sensitivity Analysis for Maximum Abatacept Concentration Using IPRED

eFigure 8. Maximum Abatacept Concentration Using DV

eReferences.

This supplementary material has been provided by the authors to give readers additional information about their work.

© 2024 Balevic SJ et al. *JAMA Network Open.*

eMethods.

PK Sample Collection and Assay

Samples were collected for PK assessment on study intervention period Day 1 (randomization/baseline) within 24 hours post-dose; study intervention period Day 8 (±1 day if hospitalized, ±3 days if after discharge); and follow-up period Day 29 (±3 days).

If participants were discharged prior to PK sample collection times, PK samples could be drawn prior to discharge. Abatacept concentration was measured in human serum by quantitative enzyme-linked immunoassay (ELISA). The assay was developed and validated by ICON Laboratory Services, Inc., with a calibration range of 1.00 (LLOQ) to 30.0 (ULOQ) ng/mL. Samples below the quantification limit (BQL) were counted and then excluded from further analyses.

PK Model Development

For model fitting, the first order conditional estimation with extended least squares (FOCE-ELS) algorithm was used and parameter precision was reported as the relative standard error (RSE), calculated as 100* standard error/parameter value. We explored both one and two-compartment structural models for abatacept, which has linear PK.[1] We estimated inter-individual variability (IIV) for all abatacept PK parameters using the relationship denoted in Equation 1[2-5] and calculated IIV as % coefficient of variation (%CV [100*square root of variance]).

$$paramater_x = \theta_{Population} * \exp(\eta_x) \quad (1)$$

© 2024 Balevic SJ et al. *JAMA Network Open.*

Where $\boldsymbol{\theta}_{\mathbf{Population}}$ denotes the parameter value for the entire population; and $\boldsymbol{\eta}_{\mathbf{x}}$ is the individual's deviation from the average population parameter value with a mean of zero and variance ω^2.

We then removed IIV on any PK parameter with high shrinkage (>30%). Equation 2 denotes the calculation for shrinkage, where $\mathbf{SD}(\boldsymbol{\eta}_{x})$ is the standard deviation of the xth ETA across all number of subjects, and $\boldsymbol{\omega x, x}$ is the population variance of the random effect.

$$Shrinkage = 1 - \frac{\mathrm{SD}(\eta_{x})}{\sqrt{\omega x, x}} \tag{2}$$

To characterize the model's residual variability, we explored several error models including multiplicative, additive, and combined as shown in Equations 3-5, respectively.[2-6]

$$C_{obs,x} = C_{pred,x} * \left(1 + \varepsilon_{prop,x}\right) \tag{3}$$

$$C_{obs,x} = C_{pred,x} + \varepsilon_{add,x} \tag{4}$$

$$C_{obs,x} = (\, C_{pred,x} \,+\, CEps \,*\, \sqrt{(1 \,+\, C_{pred,x}{}^\wedge 2 \,*\, (CMultStdev/sigma^\wedge 2))} \tag{5}$$

where $\mathbf{C}_{\mathbf{obs,x}}$ represents an observed concentration for the theoretical individual 'x'; and $\mathbf{C}_{\mathbf{pred,x}}$ represents the predicted concentration; $\boldsymbol{\varepsilon}_{\mathbf{prop,x}}$ and $\boldsymbol{\varepsilon}_{\mathbf{add,x}}$ denote random effects

© 2024 Balevic SJ et al. *JAMA Network Open.*

with a mean of zero and a respective variance of $\sigma_{prop,x}^2$ and $\sigma_{add,x}^2$. $\boldsymbol{CMultStdev}$ is the multiplicative sigma.

We evaluated several clinical covariates for inclusion into the PK model based on physiological plausibility and diagnostic plots that related the covariate to PK parameter changes from the population-typical value.[2-5] We evaluated the following covariates for their effect on abatacept clearance (CL): (1) weight; (2) concomitant medications, including tocilizumab or baricitinib (categorized as ever used), or dexamethasone (categorized as used concomitantly when the PK sample was collected); (3) baseline disease severity, categorized by both an 8-level ordinal scale (**Table 1** in main text), and dichotomously (ventilated/extracorporeal membrane oxygenation [ECMO] vs. not); (4) sex; (5) body mass index (BMI); and (6) ECMO vs not. For volume of distribution in the central compartment (V1), we evaluated the following covariates: (1) weight; (2) sex; (3) baseline disease severity; (4) BMI; and (5) ECMO vs not. ECMO use during the PK study was defined as receipt of ECMO at the same time of any PK sample collection. To evaluate for covariate inclusion in the PK model, we conducted a forward inclusion (P=0.01) and backward elimination (P=0.001) search. The P-values correspond to a change in the model's objective function value (OFV) by 6.635 and 10.828 for 1 degree of freedom, respectively.

Weight was normalized to a 70 kg adult, and body mass index (BMI) was normalized to the median population value. Equation 6 denotes the parameterization of continuous covariates and equation 7 denotes parameterization of categorical covariates.[2-5] For missing height data, BMI was calculated by imputing a height of 175.4 cm for males and 161.5 cm for females.[7]

© 2024 Balevic SJ et al. *JAMA Network Open.*

$$parameter_x = \theta_{Population} * \left(\frac{covariate_{individual}}{covariate_{median}}\right)^{\theta_{covariate}} \quad (6)$$

$$parameter_x = \theta_{Population} * \exp\left(^{\theta_{covariate}*VARIABLE}\right) \quad (7)$$

where $\boldsymbol{\theta}_{\mathbf{Population}}$ represents the typical population value; $\boldsymbol{\theta}_{\mathbf{covariate}}$ is the estimated covariate effect, and **VARIABLE** represents the presence or absence of each level of a categorical predictor.

Population PK model evaluation and validation

To determine population PK model performance, we evaluated several metrics, including model diagnostic plots, physiologic plausibility of PK parameter estimates and their precision, successful minimization, OFV, Akaike information criterion (AIC), and shrinkage values.[2,8] Additionally, Monte Carlo simulations were conducted using the final population PK model, generating the 95% confidence intervals for PK parameter estimates (1000 replicates of nonparametric bootstrapping).[2,8] To compare the simulated concentrations to those observed, we used prediction-corrected visual predictive checks (pcVPCs). Using the final population PK model we determined the empirical Bayesian estimates (EBEs) for volume of distribution in the central compartment and clearance using each individual patient's actual covariates (e.g., body weight).[2,8]

© 2024 Balevic SJ et al. *JAMA Network Open.*

Dosage Simulations

We tested the following dosage regimens: (1) The actual study dosage in ACTIV-1 (single 10 mg/kg IV infusion, with a maximum of 1000 mg); and (2) a modified rheumatoid arthritis (RA) regimen whereby patients <60 kg receive 1000 mg IV, those 60-100 kg receive 1250 mg IV, and those >100 kg receive 1500 mg IV.

Additionally, we also conducted exploratory analyses to determine whether the maximum predicted C_{max} would exceed that observed in healthy volunteers across various PK model assumptions. We chose C_{max} as this was the exposure parameter most available in the FDA drug label.[6] Using the modified rheumatoid arthritis regimen, we conducted Monte Carlo simulations (100 replicates per patient) to derive (1) individual concentrations (IPRED) which used each patient's total body weight, and the model's IIV for clearance; and (2) predicted concentrations using IPRED plus the model's residual error (DV). For each time point, we then took the average concentration across all patients and replicates and plotted the mean, 5th percentile and 95th percentile of concentrations vs time. We then compared the predicted concentrations to the abatacept C_{max} observed in healthy volunteers given a dosage of 10 mg/kg IV once (mean 292 mg/L [range 175-427]).[6]

Statistical Tests

For the logistic regression modeling, we examined linearity assumptions for exposure variables using cubic polynomial spline functions, and used transformations when necessary for non-linear relationships. We also tested the interaction between disease severity (8-point ordinal scale and as a binary term categorized as moderate vs severe) and abatacept exposure, represented by AUC_{0-28}, C_{max}, and $C_{min,}$ to determine if the impact of abatacept exposure on

© 2024 Balevic SJ et al. *JAMA Network Open.*

outcome differed across disease severity. In a sensitivity analysis, we adjusted for concomitant medication use (any use of dexamethasone, tocilizumab, or baricitinib).

© 2024 Balevic SJ et al. *JAMA Network Open.*

eResults.

Base Model Development

The data was best characterized using a two-compartment structural model. Shrinkage was high (>30%) on V1, V2, Q for the multiplicative and combined error models, and IIV was not estimated for these parameters. Additionally, shrinkage was high for V2 and Q for the additive error models and IIV was not estimated for these parameters in the additive error model.

Across residual error models, there was similar performance of a multiplicative and combined error model with an AIC of 7409 vs 7403, respectively, which was superior to the additive error model (AIC 7666). We selected the multiplicative error model due to having a fewer number of parameters and the advantage of not simulating negative concentrations (as can occur with a combined error model).

Accordingly, the final base model was a two-compartment structural model with multiplicative error and estimates of IIV on CL.

Population PK Model Sensitivity Analyses

In a sensitivity analysis, we compared post-hoc empirical Bayesian estimates for CL and V1 between the full and reduced models and found the estimates to be highly similar across the entire population. The median (range) estimates for the full covariate model were 5.05 L (3.33-7.78) for V1 and 0.04 L/hr (0.01-0.08) for CL; compared to 5.02 L (3.27-8.2) for V1 and 0.04 L/hr (0.01-0.1) for CL for the reduced model.

Exploratory Analyses for PK Model Simulations

Using IPRED concentrations, the 95th percentile of simulated concentrations did not exceed the mean C_{max} observed in healthy volunteers of 292 mg/L (**eFigure 7**).

© 2024 Balevic SJ et al. *JAMA Network Open.*

Using DV concentrations, the 50th percentile of simulated concentrations remained under 292 mg/L, but the 95th percentile exceeded 292 mg/L. However, the 95th percentile of simulated concentrations using DV did not exceed the maximum C_{max} observed in healthy volunteers of 427 mg/L (**eFigure 8**).

© 2024 Balevic SJ et al. *JAMA Network Open.*

eTable 1. Pharmacokinetic Parameters for the Base Model

Parameter	Estimate	RSE (%)
V1 (L)	5.0	3.20
V2 (L)	4.53	8.96
CL (L/hr)	0.037	2.50
Q (L/hr)	0.031	39.20
Inter-Individual Variability (%CV)		
CL	34.24	20.26
Residual Error		
Proportional error (%)	29.63	5.62

V1, volume of distribution in the central compartment; V2, volume of distribution in the peripheral compartment; CL, clearance from the central compartment; Q, intercompartmental clearance; RSE, relative standard error; CV, coefficient of variation;

© 2024 Balevic SJ et al. *JAMA Network Open.*

eFigure 1. CONSORT Diagram

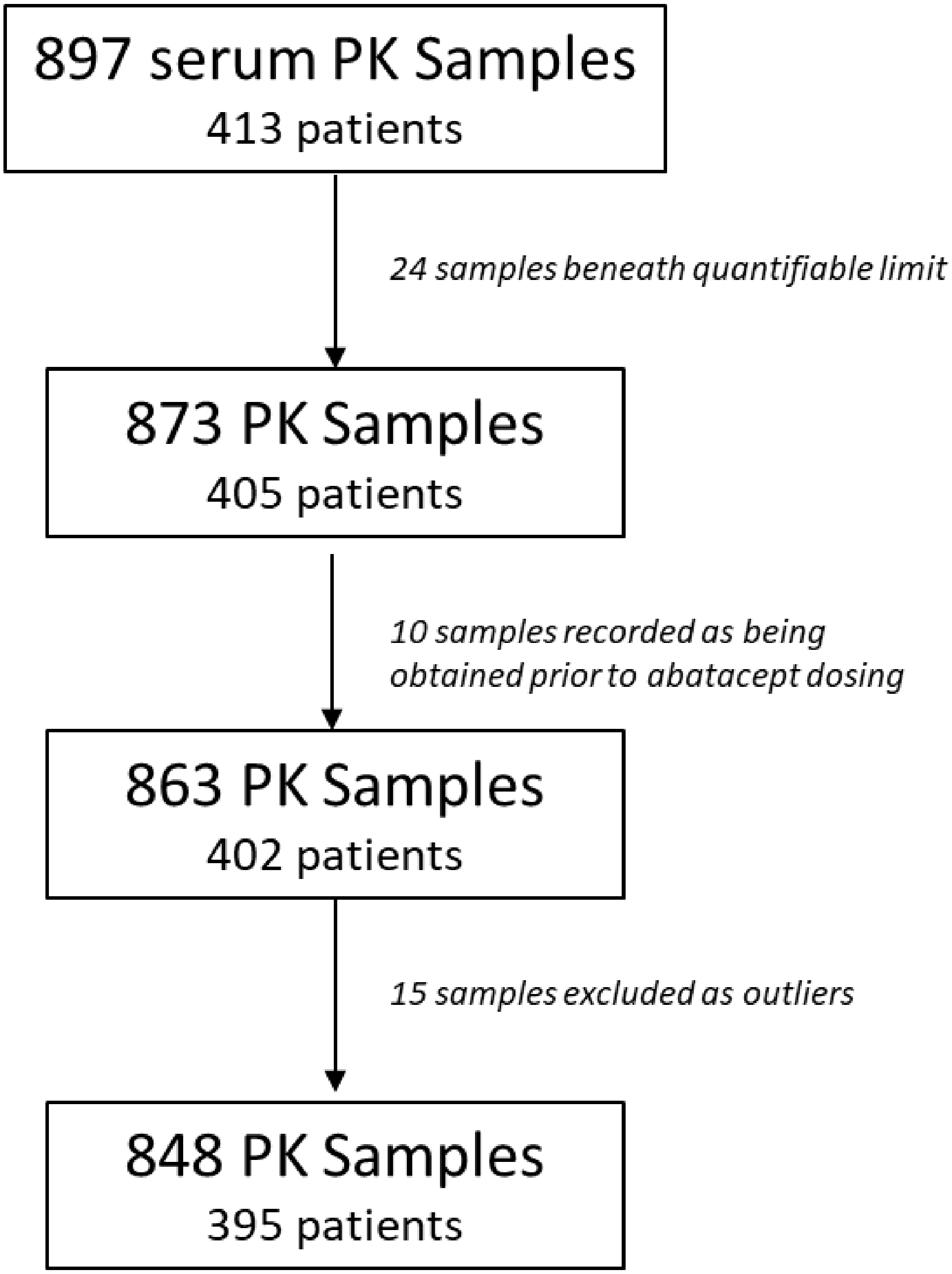

© 2024 Balevic SJ et al. *JAMA Network Open.*

eFigure 2. Diagnostic Plots for the Final Abatacept Model

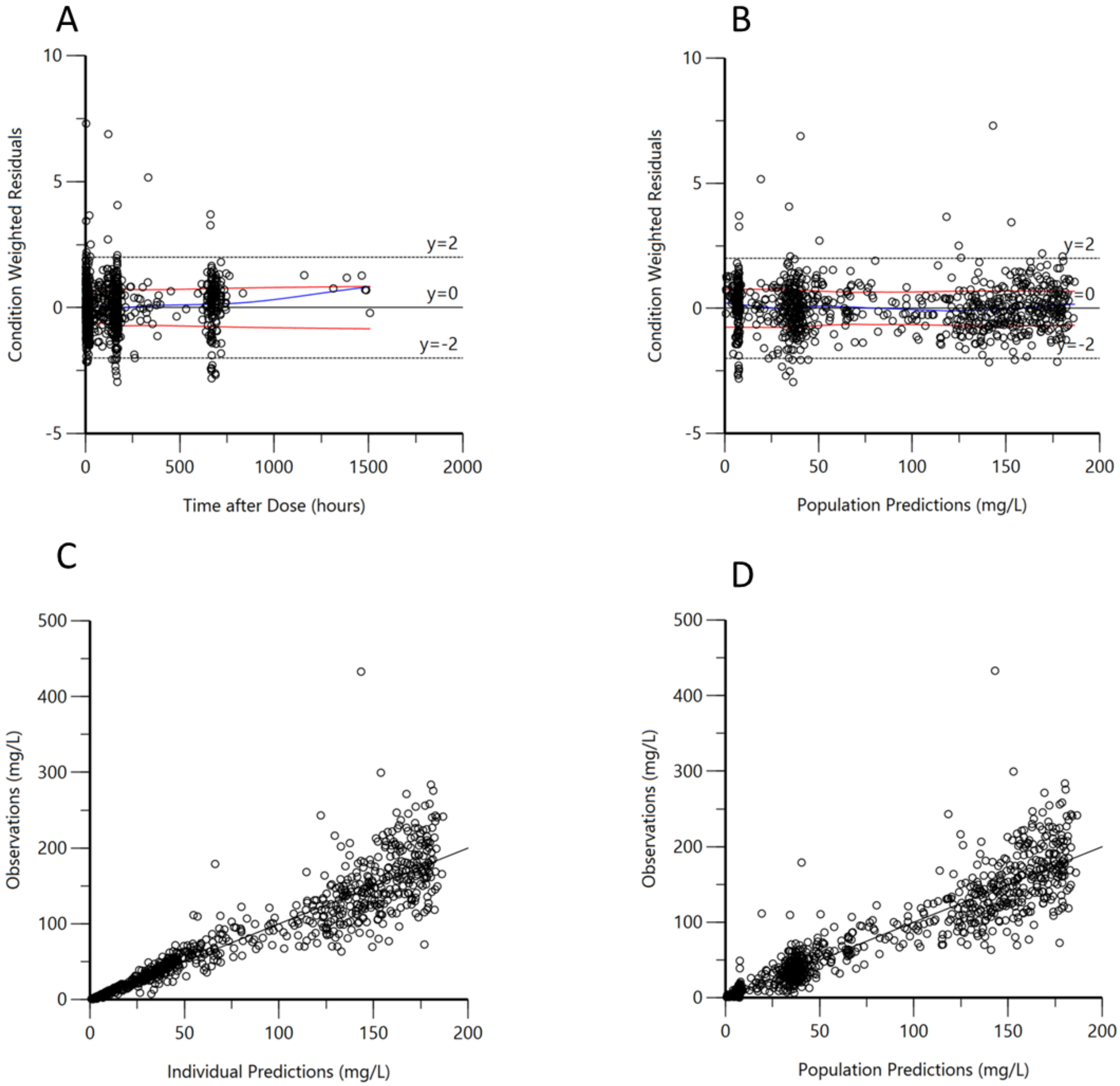

(A) Condition Weighted Residuals (CWRES) vs. Time after First Dose; (B) Condition Weighted Residuals vs. Population Predicted Concentrations; (C) Observed vs Individual Predicted Concentrations; (D) Observed vs Population Predicted Concentrations. The upper and lower dashed lines in A and B represent CWRES values of 2 and -2. The blue line is the LOESS (locally estimated scatterplot smoothing) fit while the red lines are the absolute values of the LOESS fit. The black line in C and D represent the line of unity.

© 2024 Balevic SJ et al. *JAMA Network Open.*

eFigure 3. Prediction-Corrected Visual Predictive Check for the Final Population Pharmacokinetic Model

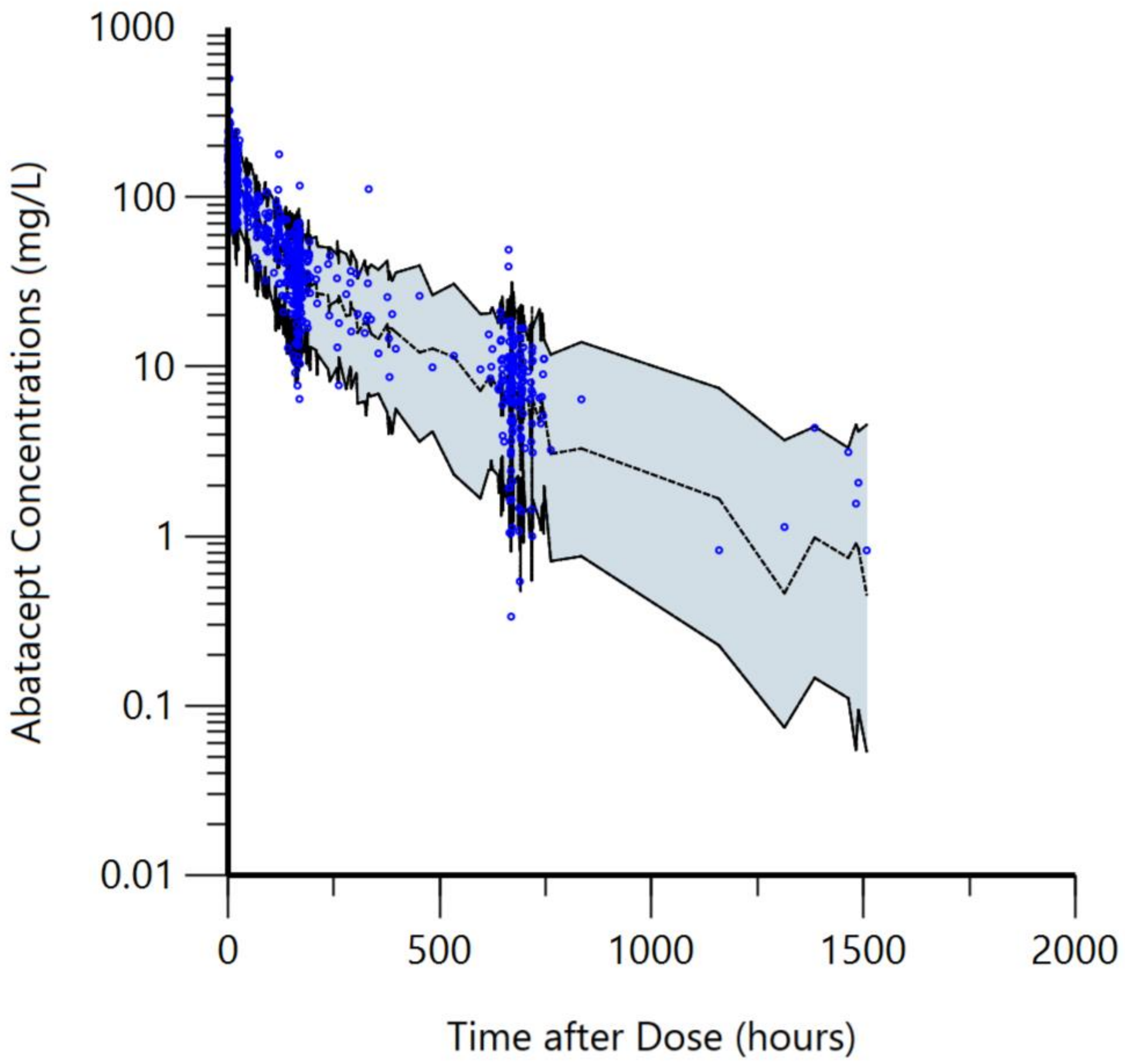

Blue circles represent the observed abatacept concentrations. The gray area depicts the 90% prediction interval with the black lines representing the 95%, 50%, and 5% predicted quantiles.

© 2024 Balevic SJ et al. *JAMA Network Open.*

eFigure 4. Abatacept Clearance and Baseline Disease Severity

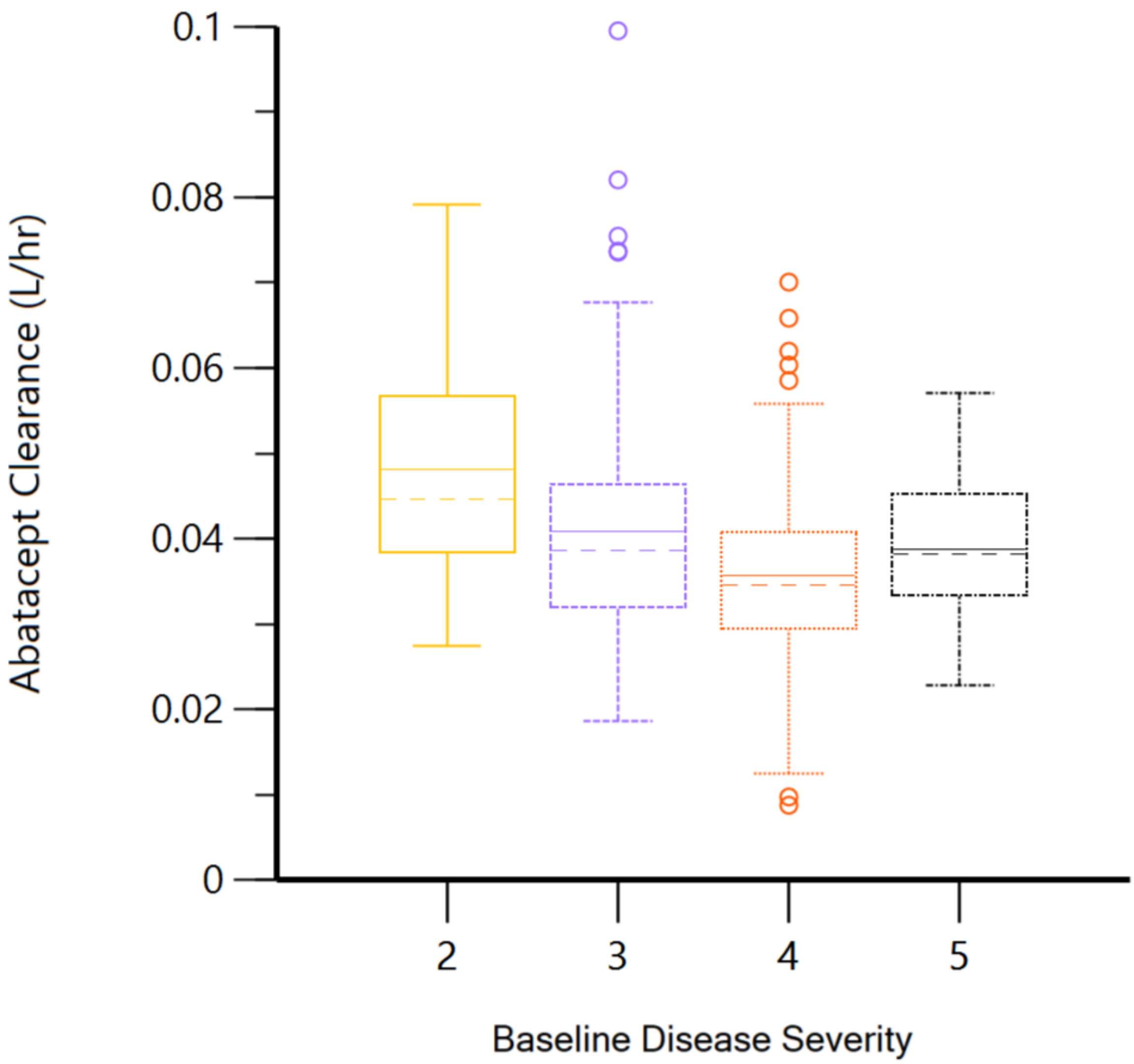

Dotted line represents the median whereas the solid line in each boxplot represents the mean. Boxes represent the interquartile range and circles represent data outside of 1.5x the interquartile range. Disease severity is an ordinal scale ranging from 1 (death) to 8 (not hospitalized, no limitations on activities).

© 2024 Balevic SJ et al. *JAMA Network Open.*

eFigure 5. Abatacept Exposure and Probability of Recovery at 28 Days

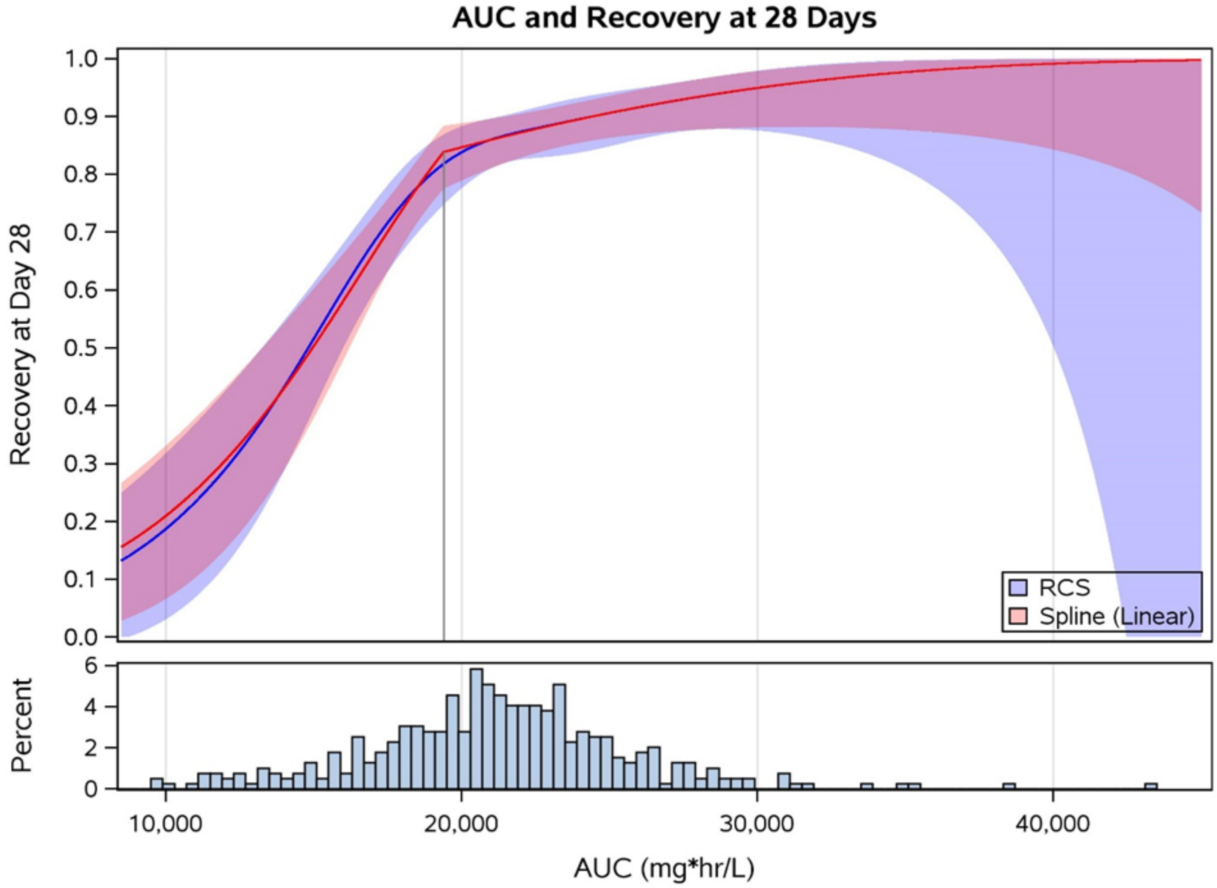

Shaded regions represent the 95% confidence intervals. RCS; restricted cubic spline. Figure generated using Cox regression with knots at the 5th, 35th, 65th, and 95th percentiles of the empirical distribution.

© 2024 Balevic SJ et al. *JAMA Network Open.*

eFigure 6. Target Abatacept Attainment From Dosage Simulations

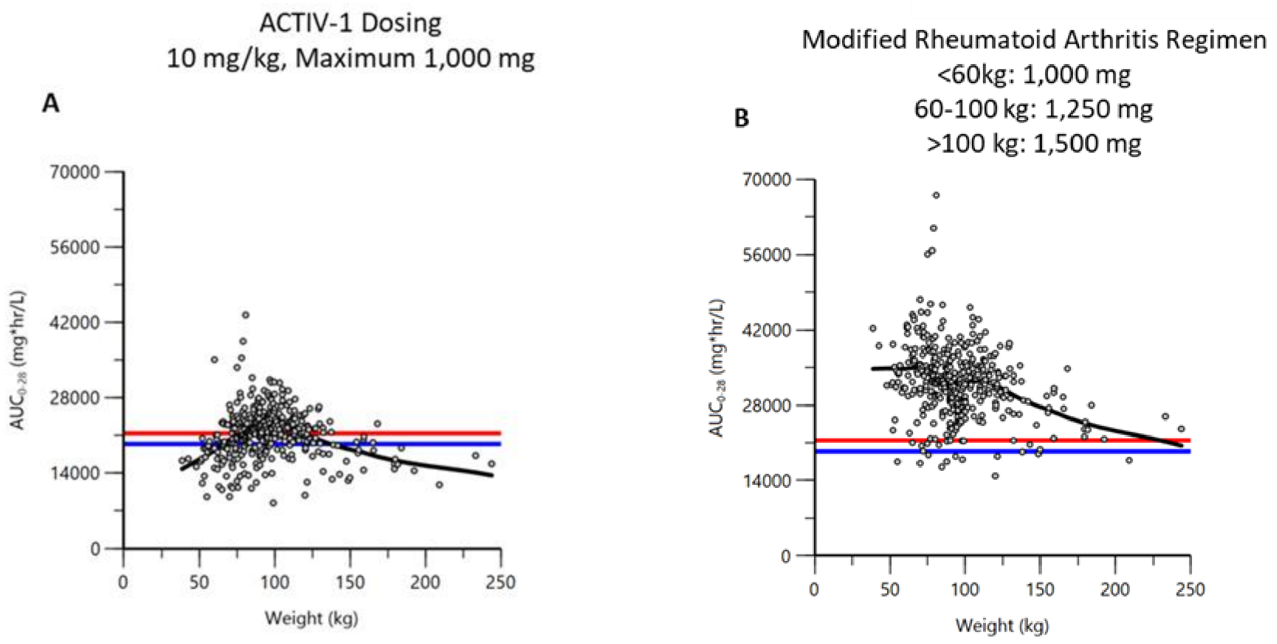

The blue line represents the target abatacept $AUC_{0\text{-}28}$ from the time-to-recovery analysis (19,400 mg*hr/L). The red line represents the median abatacept $AUC_{0\text{-}28}$ in the survival group (21,428 mg*hr/L). The black line represents LOESS (locally estimated scatterplot smoothing) fit while the silver circles represent individual patient predicted $AUC_{0\text{-}28}$. **(A)** ACTIV-1 dosage; (**B)** Modified, higher dose regimen.

© 2024 Balevic SJ et al. *JAMA Network Open.*

eFigure 7. Sensitivity Analysis for Maximum Abatacept Concentration Using IPRED

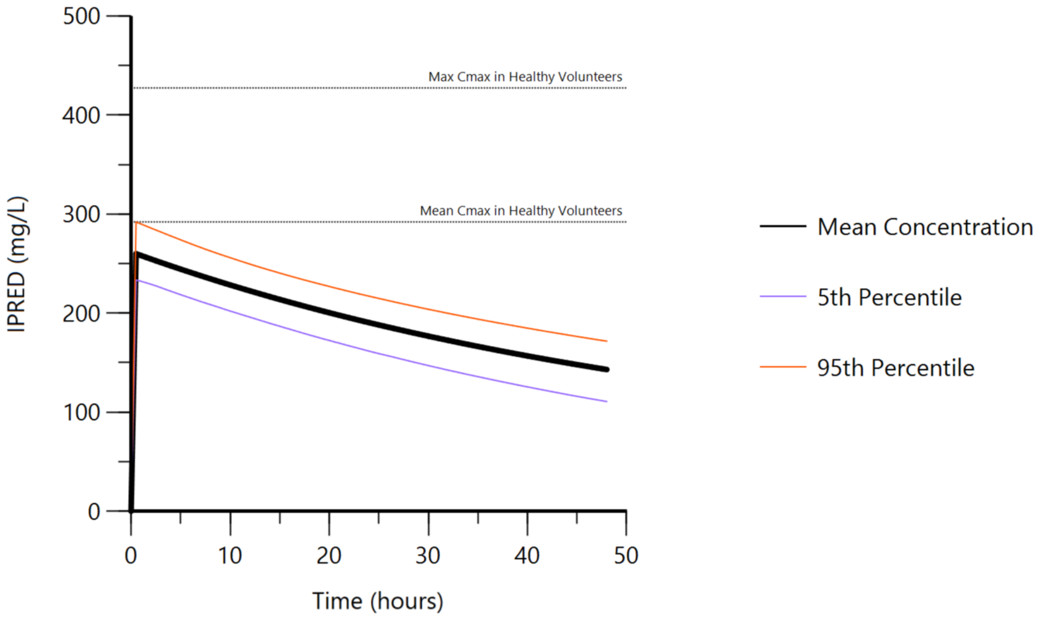

IPRED, Individual Predicted Concentrations.

© 2024 Balevic SJ et al. *JAMA Network Open.*

eFigure 8. Maximum Abatacept Concentration Using DV

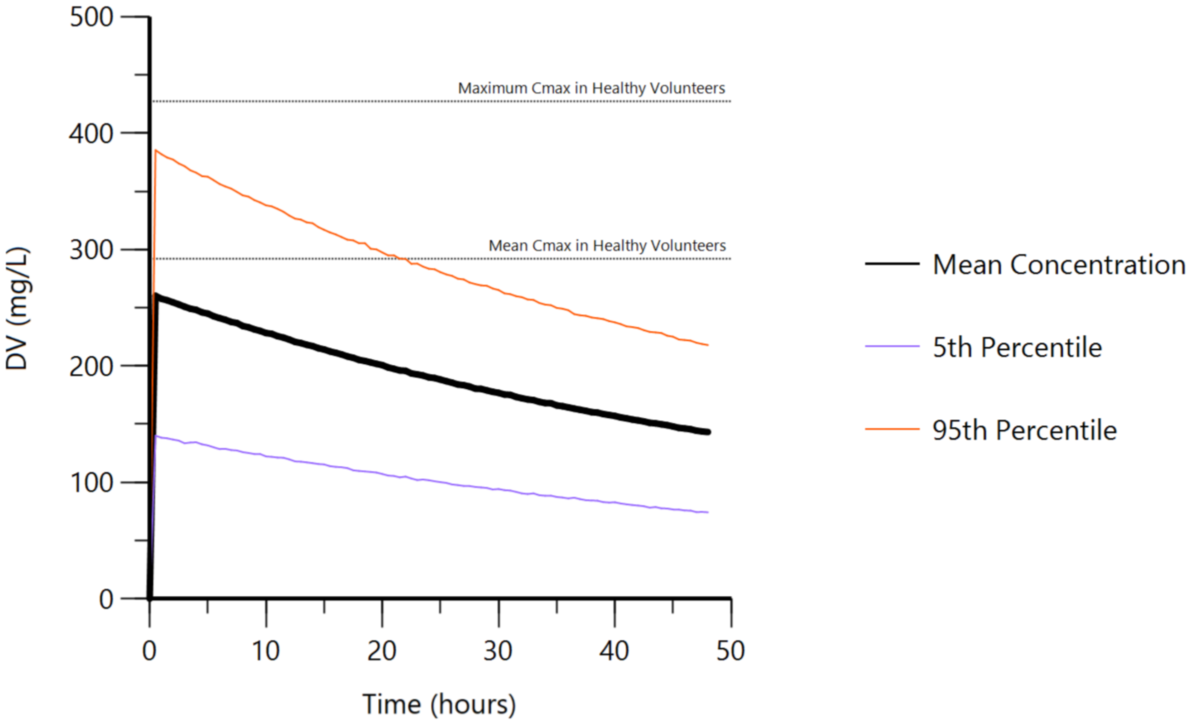

DV, Individual predicted concentrations plus model residual error.

© 2024 Balevic SJ et al. *JAMA Network Open.*

eReferences.

1. Li X, Roy A, Murthy B. Population pharmacokinetics and exposure-response relationship of intravenous and subcutaneous abatacept in patients with rheumatoid arthritis. *J Clin Pharmacol.* 2019;59(2):245-257.
2. Balevic SJ, Randell R, Weiner D, et al. Pharmacokinetics of hydroxychloroquine in paediatric lupus: Data from a novel, direct-to-family clinical trial. *Lupus Sci Med.* 2022;9(1).
3. Dallefeld SH, Atz AM, Yogev R, et al. A pharmacokinetic model for amiodarone in infants developed from an opportunistic sampling trial and published literature data. *J Pharmacokinet Pharmacodyn.* 2018;45(3):419-430.
4. Smith MJ, Gonzalez D, Goldman JL, et al. Pharmacokinetics of clindamycin in obese and nonobese children. *Antimicrob Agents Chemother.* 2017;61(4).
5. Thompson EJ, Wu H, Melloni C, et al. Population pharmacokinetics of doxycycline in children. *Antimicrob Agents Chemother.* 2019;63(12).
6. Orencia (abatacept) - Package Insert. Bristol-myers squibb company. Available at: https://www.accessdata.fda.gov/drugsatfda_docs/label/2013/125118s171lbl.pdf. Published 2013. Accessed July, 2023.
7. Fryar CD, Kruszon-Moran D, Gu Q, Ogden CL. Mean body weight, height, waist circumference, and body mass index among adults: United States, 1999-2000 through 2015-2016. *Natl Health Stat Report.* 2018(122):1-16.
8. Balevic SJ, Weiner D, Clowse MEB, et al. Hydroxychloroquine PK and exposure-response in pregnancies with lupus: The importance of adherence for neonatal outcomes. *Lupus Sci Med.* 2022;9(1).

© 2024 Balevic SJ et al. *JAMA Network Open.*
