# Supplementary material for: Abatacept Pharmacokinetics and Exposure Response in Patients Hospitalized With COVID-19: A Secondary Analysis of the ACTIV-1 IM Randomized Clinical Trial
Source: JAMA Netw Open. 2024 Apr 25;7(4):e247615. doi: 10.1001/jamanetworkopen.2024.7615 (PMC11046337; doi:10.1001/jamanetworkopen.2024.7615)
Supplement: Supplement 3. — Nonauthor Collaborators [file jamanetwopen-e247615-s003.pdf]

Supplement 3. Nonauthor Collaborators
*First name, last name, and suffix (if applicable) are required and will appear in PubMed.

***Group Name(s): ACTIV-1 IM Study Group**

***First Name and Middle Initial(s)**	***Last Name**	***Suffix (eg, Jr, III)**	Academic Degrees	Institution	Location (city, state/province, country)	Role or Contribution, eg, chair, principal investigator	Group (if more than 1 Group listed in the byline) and/or Subgroup (eg, Steering Committee)
Mahendra	Patel						
Arun	Sanyal						
Jason	Green						
Huimin	Wu						
Benjamin	Linas						
Philip	Grant						
Vivek	Iyer						
Otto	Yang						
Bindu	Balani						
Sam	Parnia						
Ryan	Dare						
Caryn G.	Morse						
Estelle S.	Harris						
Glenn	Wortmann						
Nicholas	Hill						
Shama	Patel						
Julia	Garcia-Diaz						
Suman	Thapamager						
Megan	Devine						
Christine M.	Bojanowski						
Barry	Meisenberg						
Gailen	Marshall						
Dima	Dandachi						
Arick	Sabin						
Anthony	Breemo						
Suman	Sinha						
Christopher	Goss						
Rebecca	Reece						
Arlette	Aouad						
Seth	Glassman						

Supplement 3. Nonauthor Collaborators
*First name, last name, and suffix (if applicable) are required and will appear in PubMed.

***First Name and Middle Initial(s)**	***Last Name**	***Suffix (eg, Jr, III)**	Academic Degrees	Institution	Location (city, state/province, country)	Role or Contribution, eg, chair, principal investigator	Group (if more than 1 Group listed in the byline) and/or Subgroup (eg, Steering Committee)
Peter	Morris						
Bela	Patel						
Fatimah	Bello						
Juliana	Cardozo Fernandes						
Oscar	Carbajal						
Lorena	Ravera						
Mozar	Castro						
Miguel	Villegas-Chiroque						
Fernando	Oscar Riera						
Adrian	Camacho						
Claudio	Stadnik						
Jorge	Gave						
Rodrigo	Biondi						
Ronal	Gamarra Velarde						
Jose	Cerbino Neto						
Juan	Ditondo						
Marcelo H.	Losso						
Mariano	Dolz						
Alexandra	O’Sullivan						
Brian	Gavin						
Maria	Beumont-Mauviel						
Huyen	Ca						
Rose	Beci						
Daniel	Molina						
Sandhya	Rao						
Thomas	Stock						
William	Erhardt						
Sarah	Read						
Jessica	Springer						
Rachel	Presti						
Ryley	Thompson						
Kimberly	Gray						
Cathy	Henry						

Supplement 3. Nonauthor Collaborators

*First name, last name, and suffix (if applicable) are required and will appear in PubMed.

***First Name and Middle Initial(s)**	***Last Name**	***Suffix (eg, Jr, III)**	Academic Degrees	Institution	Location (city, state/province, country)	Role or Contribution, eg, chair, principal investigator	Group (if more than 1 Group listed in the byline) and/or Subgroup (eg, Steering Committee)
Alem	Haile						
Michael	Klebert						
Lisa	Kessels						
Kathryn	Vehe						
Kristopher	Bakos						
Teresa	Spitz						
Sara	Hubert						
Raghd	Alyatim						
Brittany	Schneider						
Chapelle	Ayres						
Andrej	Spec						
Laura	Blair						
Anita	Afghanzada						
Natalie	Schodl						
Lana	Wahid						
John J.	Engemann						
Gloria	Pinero						
Beth	McLendon-Arvik						
Lynn	Whitt						
Jenny	Shroba						
Elizabeth	Salsgiver						
Candace	Alleyne						
Anna	Gwak						
Nicholas	Pickell						
Jack	Spagnoletti						
Samson	Goh						
Katharine	Robb						
Michael	Cenname						
Catherine	Small						
Markus	Plate						
Mahendra	Patel						
Rodrigo	Burgos						
Brenna	Lindsey						

Supplement 3. Nonauthor Collaborators

*First name, last name, and suffix (if applicable) are required and will appear in PubMed.

***First Name and Middle Initial(s)**	***Last Name**	***Suffix (eg, Jr, III)**	Academic Degrees	Institution	Location (city, state/province, country)	Role or Contribution, eg, chair, principal investigator	Group (if more than 1 Group listed in the byline) and/or Subgroup (eg, Steering Committee)
Fischer	Herald						
Stephanie	Echeverria						
Dorendra	Lewis						
Mahesh	Patel						
Rodrigo	Burgos						
Fischer	Herald						
Charles D.	Bengtson						
Andreas	Schmid						
Kimberly	Lovell						
Carly	Lovelett						
Daniel	Soule						
Daniel	Jaremczuk						
Jennie	Flanagan						
Cameron	Murray						
Kylie	Sands						
Kyle	Flint						
Sara	Mohaddes						
Caryn	Harrington						
Kylie	Broughal						
David	Sogoian						
Karen	Cox						
Heta	Javeri						
Philip O.	Ponce						
Danielle O.	Dixon						
Jason E.	Bowling						
Jan E.	Patterson						
Barbara S.	Taylor						
Ruth C.	Serrano						
Kaylin	Sallee						
Robin	Tragus						
Gabriel	Catano						
Irma	Scholler						
Rose Ann	Barajas						

Supplement 3. Nonauthor Collaborators
*First name, last name, and suffix (if applicable) are required and will appear in PubMed.

***First Name and Middle Initial(s)**	***Last Name**	***Suffix (eg, Jr, III)**	Academic Degrees	Institution	Location (city, state/province, country)	Role or Contribution, eg, chair, principal investigator	Group (if more than 1 Group listed in the byline) and/or Subgroup (eg, Steering Committee)
Armando	Garcia						
Bridgette	Soileau						
Patricia	Heard						
Manuel	Camilo Endo Carvajal						
Rukevwe	Ehwarieme						
Divya	Chandramohan						
Alejandro	Cabo						
Abdelhameed	Nawwar						
Caroline M.	Quill						
Nayeem	Choudhury						
Ashley	Arrington						
Isaiah	Holyfield						
Arun	Sanyal						
Jason	Green						
Abby	Smith						
Glenda	Brown						
Kyle	Varner						
Joni	Baxter						
Tracy	Roundy						
Mary	Co						
Mireya	Wessolossky						
Juan	Perez-Velazquez						
Huimin	Wu						
Jennifer	Holter-Chakrabarty						
Brittany	Karfonta						
Juvaria	Anjum						
Benjamin	Linas						
Jai	Marathe						
Myriam	Castagne						
Daniel	Mompoint						
Ryan	Schroeder						
Philip	Grant						
Mallika	Rao						

Supplement 3. Nonauthor Collaborators
*First name, last name, and suffix (if applicable) are required and will appear in PubMed.

***First Name and Middle Initial(s)**	***Last Name**	***Suffix (eg, Jr, III)**	Academic Degrees	Institution	Location (city, state/province, country)	Role or Contribution, eg, chair, principal investigator	Group (if more than 1 Group listed in the byline) and/or Subgroup (eg, Steering Committee)
Johnathan	Nguyen						
Jake	Plewa						
Vivek	Iyer						
Sue	Donlinger						
Otto	Yang						
Bindu	Balani						
Marylynn	Breslin						
Sam	Parnia						
Ryan	Dare						
Susan	Dodson						
Mitch	Jenkins						
Caryn G.	Morse						
John	Williamson						
Estelle S.	Harris						
Elizabeth A.	Middleton						
Glenn	Wortmann						
Nicholas	Hill						
Shama	Patel						
Julia	Garcia-Diaz						
Suman	Thapamager						
Megan	Devine						
Christine M.	Bojanowski						
Mai	Tavadze						
Barry	Meisenberg						
Gailen	Marshall						
Dima	Dandachi						
Arick	Sabin						
Anthony	Breemo						
Romai	Sebhatu						
Jessica	Pierobon						
Nate	Miller						
John	Lee						
Suman	Sinha						

Supplement 3. Nonauthor Collaborators
*First name, last name, and suffix (if applicable) are required and will appear in PubMed.

***First Name and Middle Initial(s)**	***Last Name**	***Suffix (eg, Jr, III)**	Academic Degrees	Institution	Location (city, state/province, country)	Role or Contribution, eg, chair, principal investigator	Group (if more than 1 Group listed in the byline) and/or Subgroup (eg, Steering Committee)
Christopher	Goss						
Rebecca	Reece						
Arlette	Aouad						
Seth	Glassman						
Peter	Morris						
Bela	Patel						
Pratik	Doshi						
Fatimah	Bello						
Andrew	Dentino						
Jessica	Martin						
Erik	Hinojosa						
Pablo	Torres						
Ricardo	Sanchez						
Gladys	Murga						
Silvana	de la Gala						
Jhon	Chaiña						
Gladys	Murga						
Jorge	Ramos						
Jenny	Malca						
Kathia	Castillo						
Johana	Calderon Galvez						
Maria Lyda	Icochea Perez						
Claudia Carolina	Becerra Nunez						
Sandra	Betteta Riondato						
Sandra	Delgado Málaga						
Cecilia	Barreda Sánchez						
Sylvia	Sánchez Morales						
Myriam	Yaringano Palacios						
Dora	Galarza Cuba						
Ivan	Hermenegildo						
Mayra	Falla Benites						
Stefania	Neyra						
Josefina	Hernández						

Supplement 3. Nonauthor Collaborators
*First name, last name, and suffix (if applicable) are required and will appear in PubMed.

***First Name and Middle Initial(s)**	***Last Name**	***Suffix (eg, Jr, III)**	Academic Degrees	Institution	Location (city, state/province, country)	Role or Contribution, eg, chair, principal investigator	Group (if more than 1 Group listed in the byline) and/or Subgroup (eg, Steering Committee)
Victoria	García						
Katherine	Palacios						
Miluska	Matos						
Fiorella	Zuloeta						
Fiorella	del Carpio						
Gloria	Chacaltana						
Carmen	de la Cruz						
Juliana	Cardozo Fernandes						
Felipe	Ceriolli Breda						
Mauricio	Mello Roux Leite						
Tobias	Milbradt						
Luz	Rodeles						
Nadia	Benzaquen						
Sebastian	Pezzini						
Lucila	Alberdi						
Priscila	Serravalle						
Giulia	Russo						
Franco	Ferini						
Maria Eugenia	Guala						
Alejandro	Crespo						
Agostina	Benitez						
Maria Elena	Cristaldi						
Paula	Di Renzo						
Corina	Gramagalia						
Antonela	Tessini						
Joana Evelin	Alonso						
Carmen	Pic						
Georgina	Ceraldi						
Azucena	Mondino						
Iliana	Higareda Almaraz						
Víctor Hugo	Madrigal Robles						
María Fernanda	Rosas Ismerio						
Maria Fernanda	Rodarte Rodriguez						

Supplement 3. Nonauthor Collaborators
*First name, last name, and suffix (if applicable) are required and will appear in PubMed.

***First Name and Middle Initial(s)**	***Last Name**	***Suffix (eg, Jr, III)**	Academic Degrees	Institution	Location (city, state/province, country)	Role or Contribution, eg, chair, principal investigator	Group (if more than 1 Group listed in the byline) and/or Subgroup (eg, Steering Committee)
Norma Esther	Olmos Meza						
Norma Esther	de la Cruz Barba						
Ana Maria	Alba Ponce						
Juan Manuel	Calderon						
Eduardo	Borsetta						
Noemí	Sandoval						
Daniela	Vazquez						
Malena	Mansilla						
Marta	Molina						
Yamila	Jara						
Laura	De Bona						
Maria	Eduarda Claus						
Arthur	Pille						
Matías	Lahitte						
Mariángeles	Fenés						
Cecilia	Bianchi						
María Emilia	Miserere						
Maria Fernanda	Alzogaray						
Oscar	Carbajal						
Lorena	Ravera						
Mozar	Castro						
Miguel	Villegas-Chiroque						
Halbert	Christian Sanchez Carrillo						
Fernando Oscar	Riera						
Aldana	Mano						
Adrian	Camacho						
Claudio	Stadnik						
Jorge	Gave						
Rodrigo	Biondi						
Ronal	Gamarra Velarde						
Jose	Cerbino Neto						
Juan	Ditondo						
Myrna	Zuain						

Supplement 3. Nonauthor Collaborators
*First name, last name, and suffix (if applicable) are required and will appear in PubMed.

***First Name and Middle Initial(s)**	***Last Name**	***Suffix (eg, Jr, III)**	Academic Degrees	Institution	Location (city, state/province, country)	Role or Contribution, eg, chair, principal investigator	Group (if more than 1 Group listed in the byline) and/or Subgroup (eg, Steering Committee)
Marcelo H.	Losso						
Javier J.	Toibaro						
Valeria	Pachioli						
Sebastián	Chaio						
Natalia	Malamud						
Mariano	Dolz						
David B.	Bharucha						
Alexandra	O'Sullivan						
Patrick	Dorr						
Brian	Gavin						
Jonathan	Sadeh						
Sheila	Kelly						
Maria	Beumont-Mauviel						
Marita	Stevens						
Huyen	Cao						
Adam	DeZure						
Kavita	Juneja						
Mazin	Abdelghany						
Theresa	Jasion						
Rachel	Olson						
Megan	Roebuck						
Jacqueline	Huvane						
Christopher J.	Lindsell						
Jeff	Leimberger						
Eric	Yow						
Zhen	Huang						
Hwasoon	Kim						
Carla	Anderson						
Carrie	Elliott						
Merri	Swartz						
Rose	Beci						
Jyotsna	Garg						
Daniel	Molina						

Supplement 3. Nonauthor Collaborators
*First name, last name, and suffix (if applicable) are required and will appear in PubMed.

***First Name and Middle Initial(s)**	***Last Name**	***Suffix (eg, Jr, III)**	Academic Degrees	Institution	Location (city, state/province, country)	Role or Contribution, eg, chair, principal investigator	Group (if more than 1 Group listed in the byline) and/or Subgroup (eg, Steering Committee)
Neta	Nelson						
Divya	Kalaria						
Sandhya	Rao						
Ketty	Philogene						
Tim	Schulz						
Averie	Kuek						
Fatou	Bah						
Jarrard	Mitchell						
Elizabeth	Polo						
Michelle	Wong						
Sharon	Baldan						
Sandra	Mendez						
Bradford	Stevens						
Marcela	Toledo						
Talita	Abba						
Emma	Herrejon						
Cristina	Gomez						
Georgeta	Mardari						
Neeraja	Putta						
Thomas	Stock						
William	Erhardt						
Sarah	Read						
Robin	Mason						
Holli	Hamilton						
Derek	Eisnor						
Anna	O’Rourke						
Aditi	Patel						
Betty	Brody						
Anna	Chiang						
Jessica	Springer						
Brian	Lind						
Lilli M.	Portilla						
Ami D.	Gadhia						

Supplement 3. Nonauthor Collaborators
*First name, last name, and suffix (if applicable) are required and will appear in PubMed.

***First Name and Middle Initial(s)**	***Last Name**	***Suffix (eg, Jr, III)**	Academic Degrees	Institution	Location (city, state/province, country)	Role or Contribution, eg, chair, principal investigator	Group (if more than 1 Group listed in the byline) and/or Subgroup (eg, Steering Committee)
Sury	Vepa						
Emily	Carlson Marti						
Bobbi	Gardner						
Joni	Rutter						
Clare	Schmitt						
Michael	Kurilla						
