# Supplementary material for: Abatacept Pharmacokinetics and Exposure Response in Patients Hospitalized With COVID-19: A Secondary Analysis of the ACTIV-1 IM Randomized Clinical Trial
Source: JAMA Netw Open. 2024 Apr 25;7(4):e247615. doi: 10.1001/jamanetworkopen.2024.7615 (PMC11046337; doi:10.1001/jamanetworkopen.2024.7615)
Supplement: Supplement 4. — Data Sharing Statement [file jamanetwopen-e247615-s004.pdf]

Data Sharing Statement

Balevic. Abatacept Pharmacokinetics and Exposure Response in Patients Hospitalized With COVID-19. *JAMA Netw Open*. Published April 25, 2024.
doi:10.1001/jamanetworkopen.2024.7615

Data
Data available: Yes
Data types: Deidentified participant data
How to access data: De-identified participant data from the primary trial is available through the NIAID Clinical Trials Data Repository.
When available: beginning date: 10-01-2023

Supporting Documents
Document types: None

Additional Information
Who can access the data: Researchers looking to access NIAID COVID-19 and other sponsored clinical trials data may submit a data access request, will be reviewed by the NIAID Clinical Trials Data Access Committee.
Types of analyses: Any analysis approved by the NIAID Clinical Trials Data Access Committee
Mechanisms of data availability: Upon approval of the data access request, the primary data requestor and their institution will be notified and required to agree to and sign a NIAID Data Use Agreement (DUA) online via DocuSign that outlines the terms of the use of the data.
